# Supplementary material for: Selective emergence of photoluminescence at telecommunication wavelengths from cyclic perfluoroalkylated carbon nanotubes
Source: Commun Chem. 2023 Jul 31;6:159. doi: 10.1038/s42004-023-00950-1 (PMC10390534; doi:10.1038/s42004-023-00950-1)
Supplement: Supplementary file 2 — Supplementary Information [file 42004_2023_950_MOESM2_ESM.pdf]

Supplementary information

Selective emergence of photoluminescence at telecommunication wavelengths from cyclic perfluoroalkylated carbon nanotubes

Yutaka Maeda[1*], Yasuhiro Suzuki[1], Yui Konno[1], Pei Zhao[2*], Nobuhiro Kikuchi[1], Michio Yamada[1], Masaya Mitsuishi[3], Anh T. N. Dao[4,5], Hitoshi Kasai[4], Masahiro Ehara[2*]

[1]Department of Chemistry, Tokyo Gakugei University, Tokyo 184-8501, Japan

[2]Research Center for Computational Science, Institute for Molecular Science, Okazaki, 444-8585, Japan

[3]Graduate School of Engineering, Tohoku University, Sendai 980-8579, Japan

[4]Institute of Multidisciplinary Research for Advanced Materials (IMRAM), Tohoku University, Sendai 980-8577, Japan

[5]Graduate School of Engineering, Nagasaki University, Nagasaki 852-8521, Japan

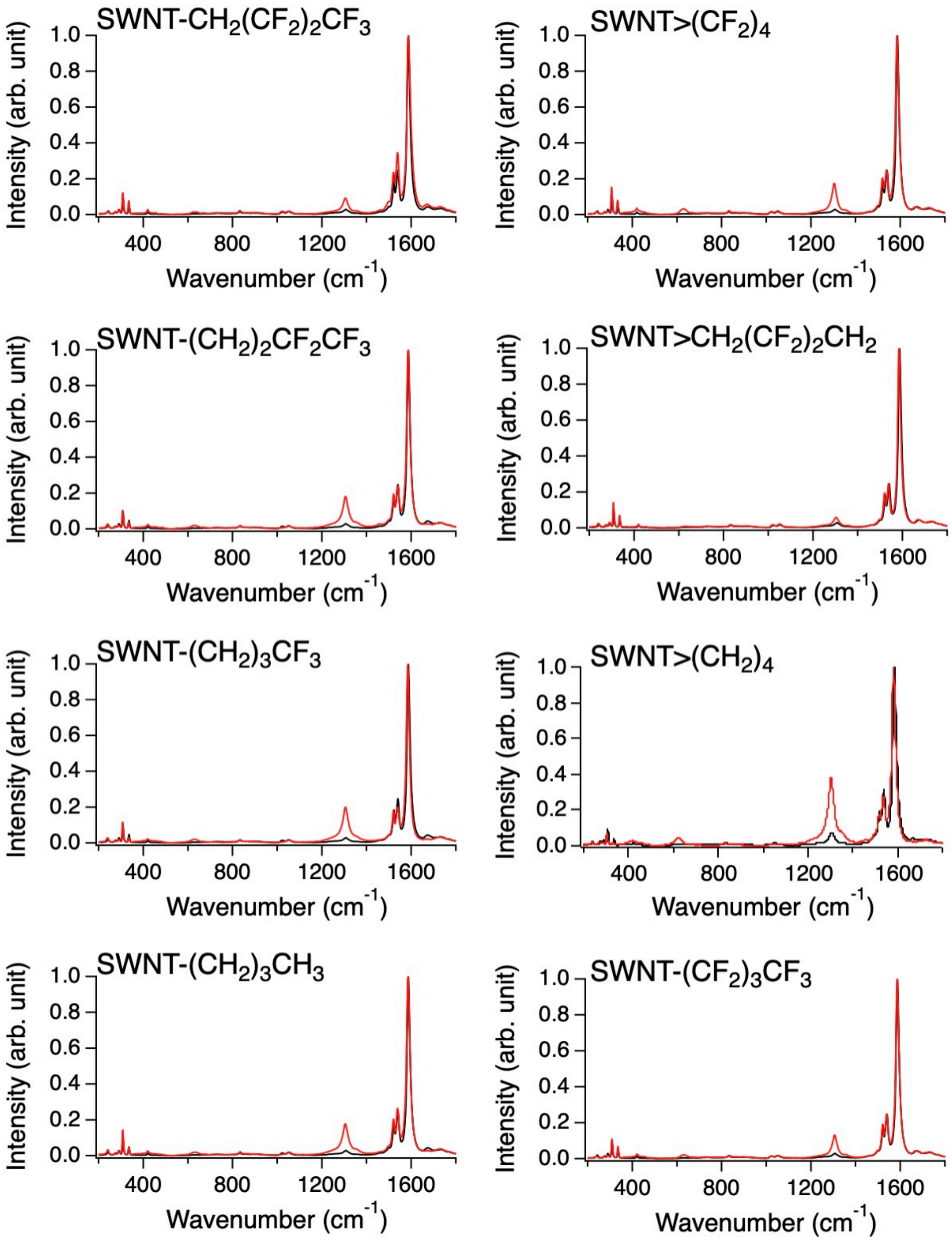

Figure S1. Raman spectra of SWNTs (black) and the functionalised SWNTs (red) at an excitation wavelength of 561 nm.

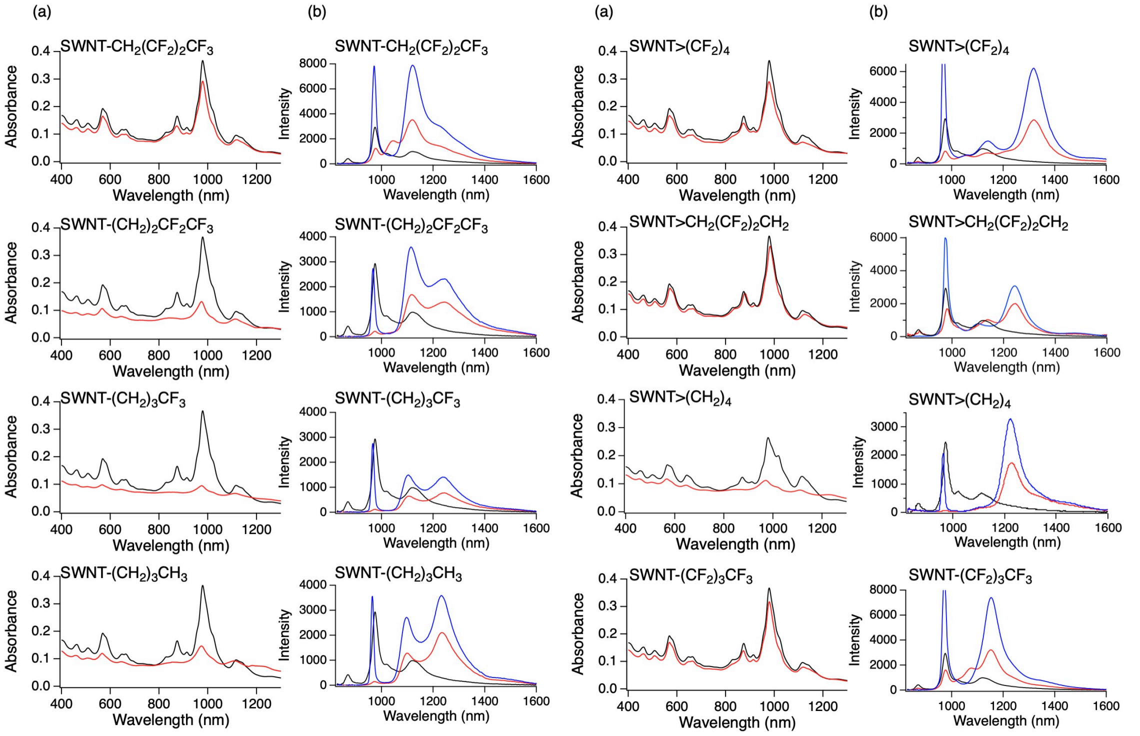

Figure S2. (a) Absorption spectra of SWNTs (black) and the functionalised SWNTs (red) dispersed in D_2O containing 1 wt% SDBS. (b) PL spectra of SWNTs (black) and the functionalised SWNTs excited at the E_{22} (red) and E_{11} wavelengths (blue).

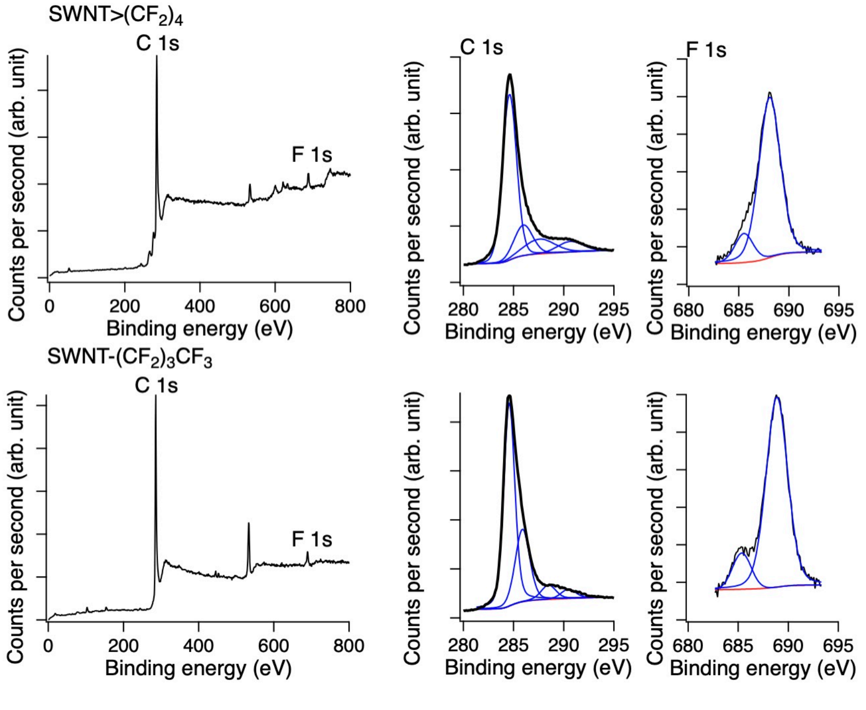

Figure S3. XPS spectra of SWNT>$(CF_2)_4$ and SWNT-$(CF_2)_3CF_3$.

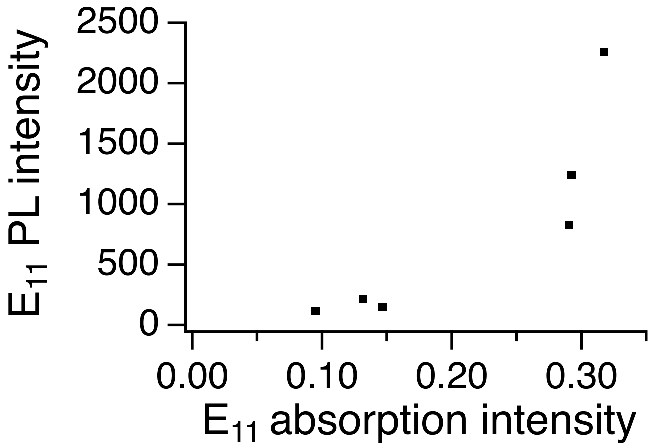

Figure S4. E_{11} PL peak intensity of the functionalised SWNTs toward SWNTs, as a function of E_{11} absorbance of the functionalised SWNTs toward SWNTs.

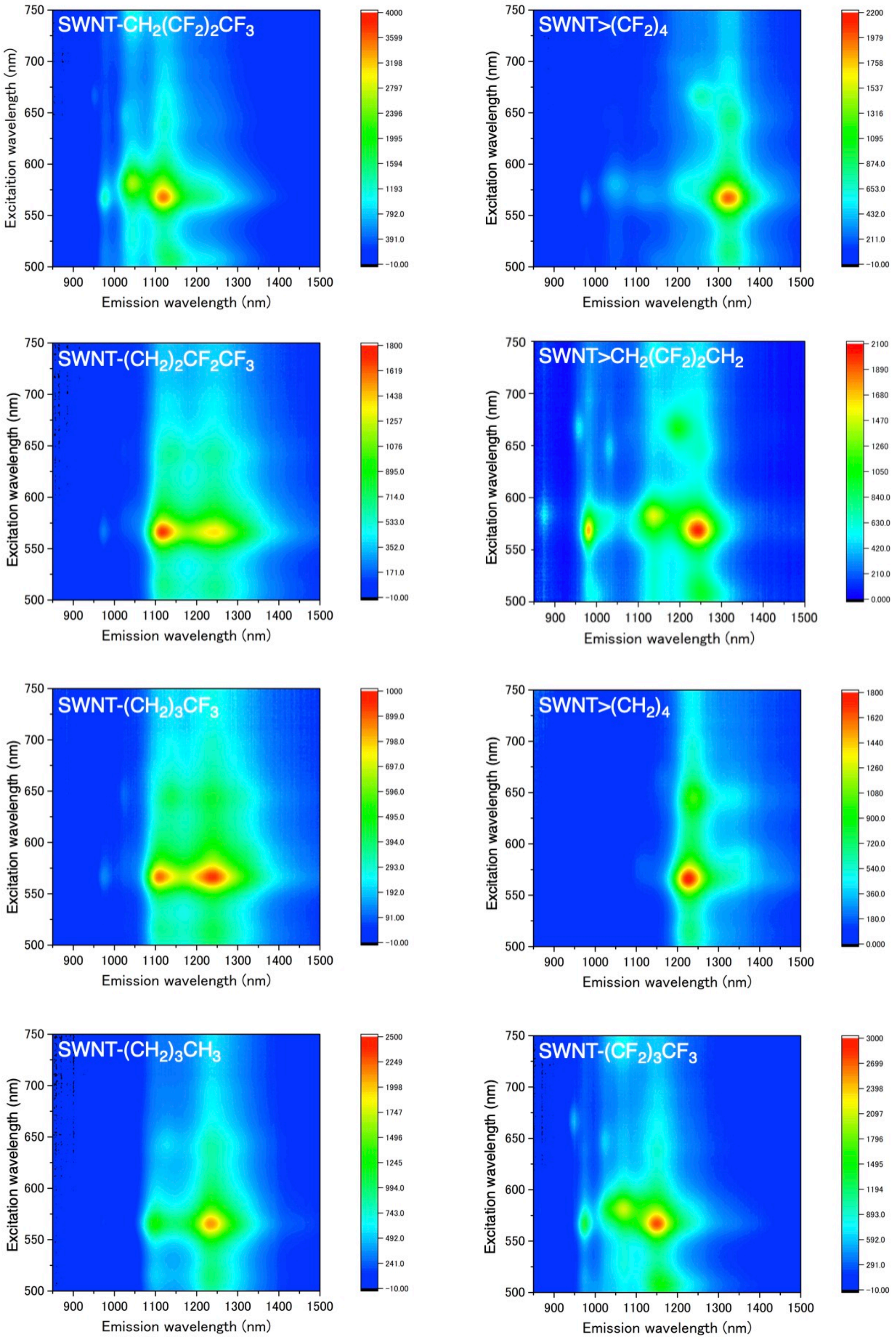

Figure S5. PL contour maps of SWNTs and the functionalised SWNTs dispersed in D_2O containing 1 wt% SDBS.

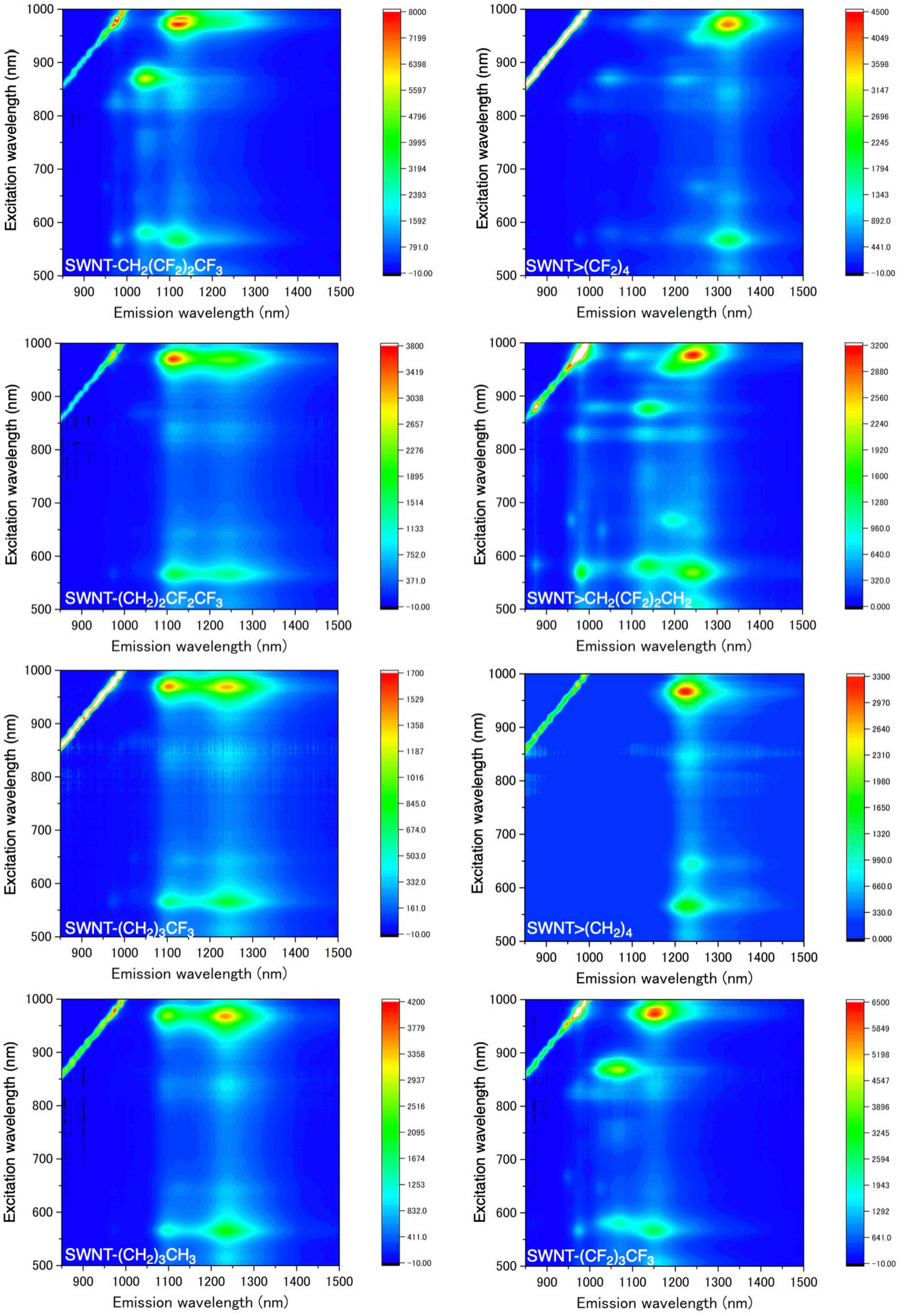

Figure S6. PL contour maps of SWNTs and the functionalised SWNTs dispersed in D_2O containing 1 wt% SDBS.

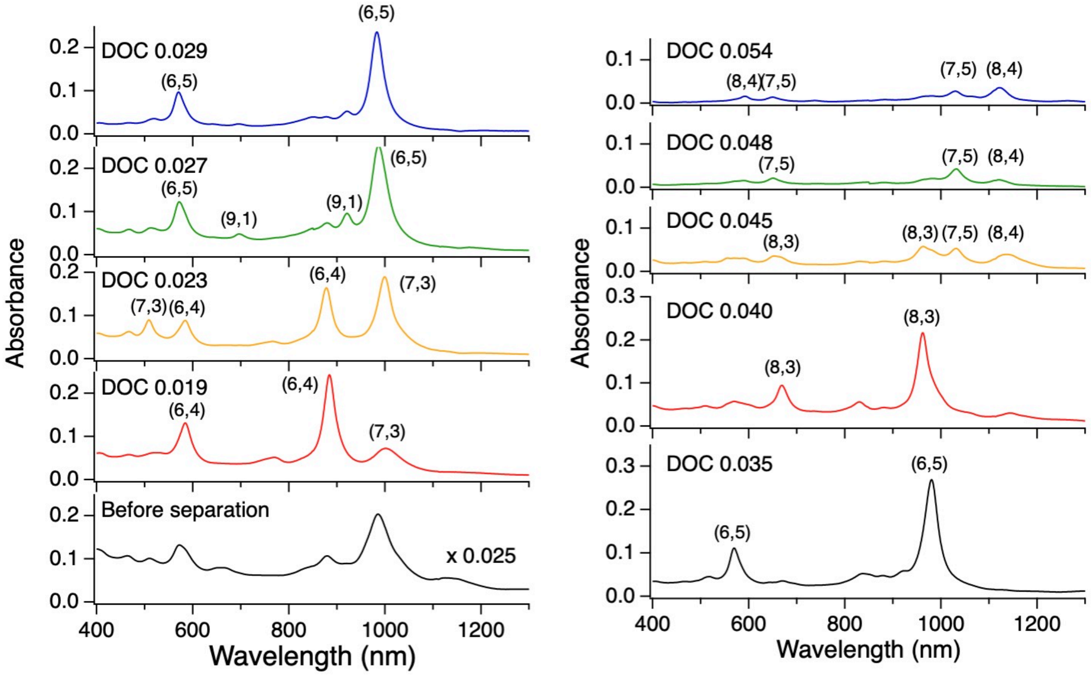

Figure S7. Absorption spectra of SWNT>$(CF_2)_4$ after separation in H_2O containing three surfactants (SDS 0.5, SC 0.5, DOC X wt%).

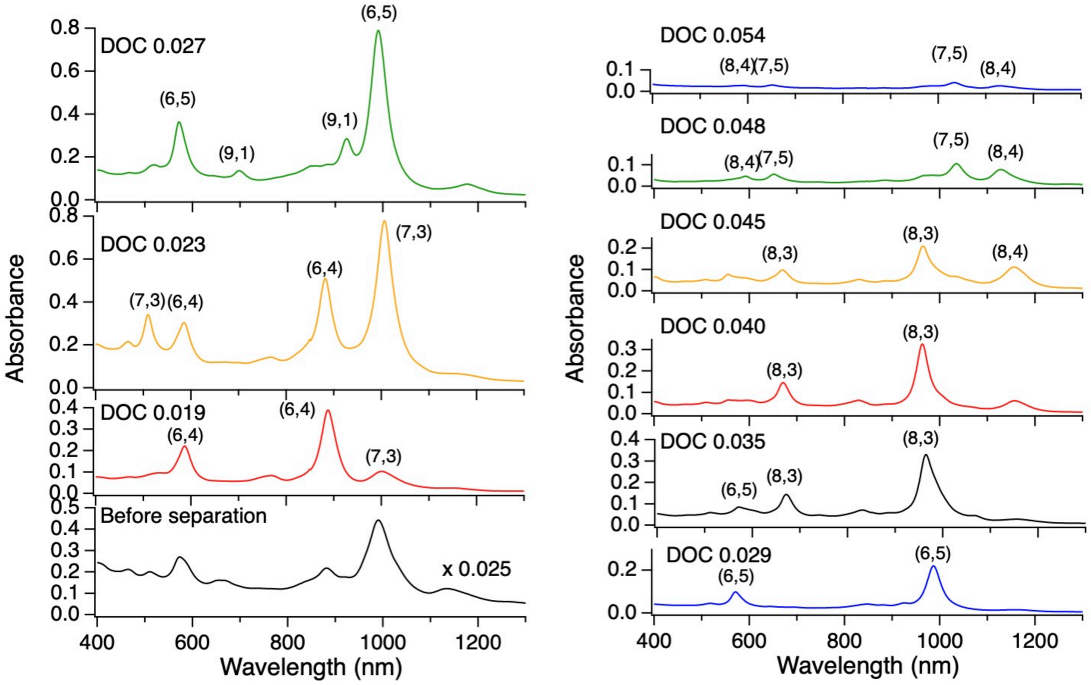

Figure S8. Absorption spectra of SWNT-$(CF_2)_3CF_3$ after separation in H_2O containing three surfactants (SDS 0.5, SC 0.5, DOC X wt%).

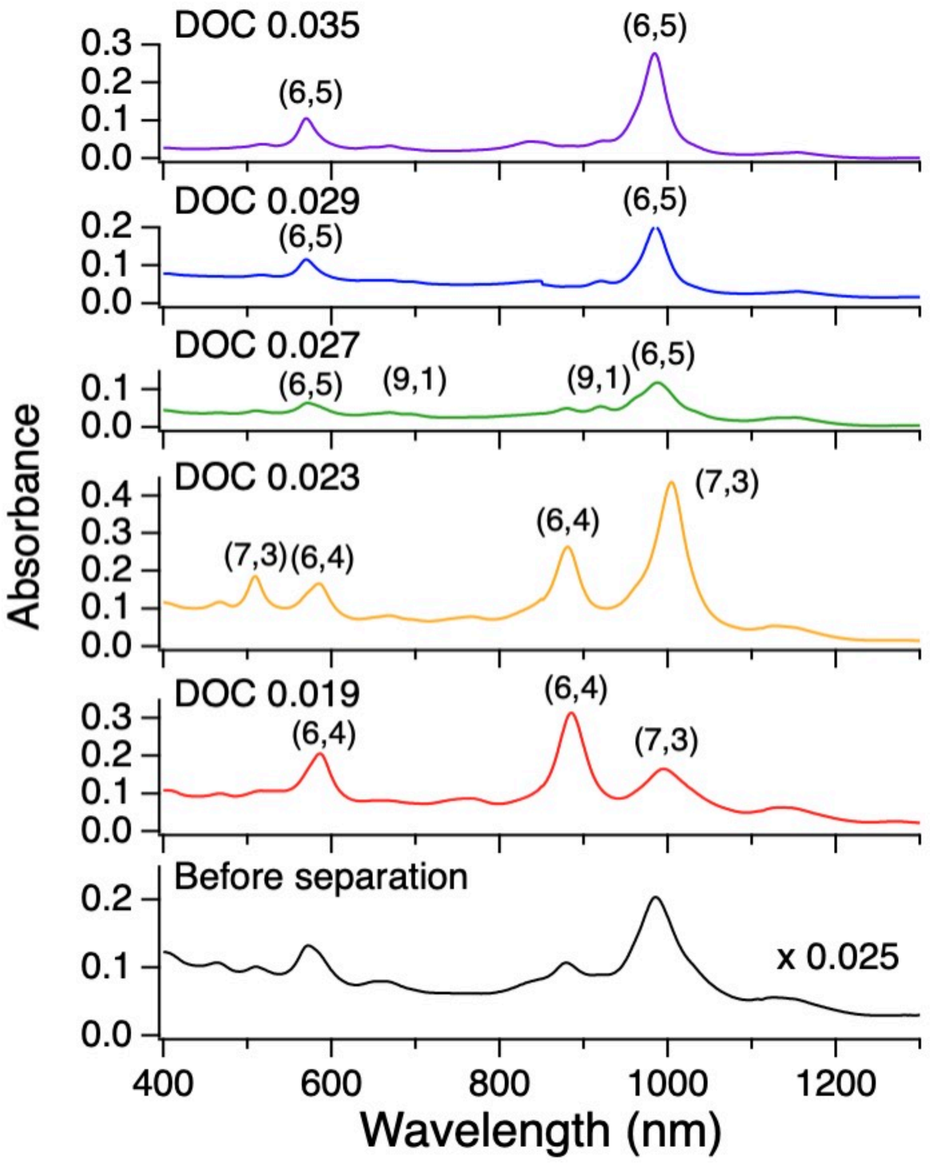

Figure S9. Absorption spectra of SWNT>$CH_2(CF_2)_2CH_2$ after separation in H_2O containing three surfactants (SDS 0.5, SC 0.5, DOC X wt%).

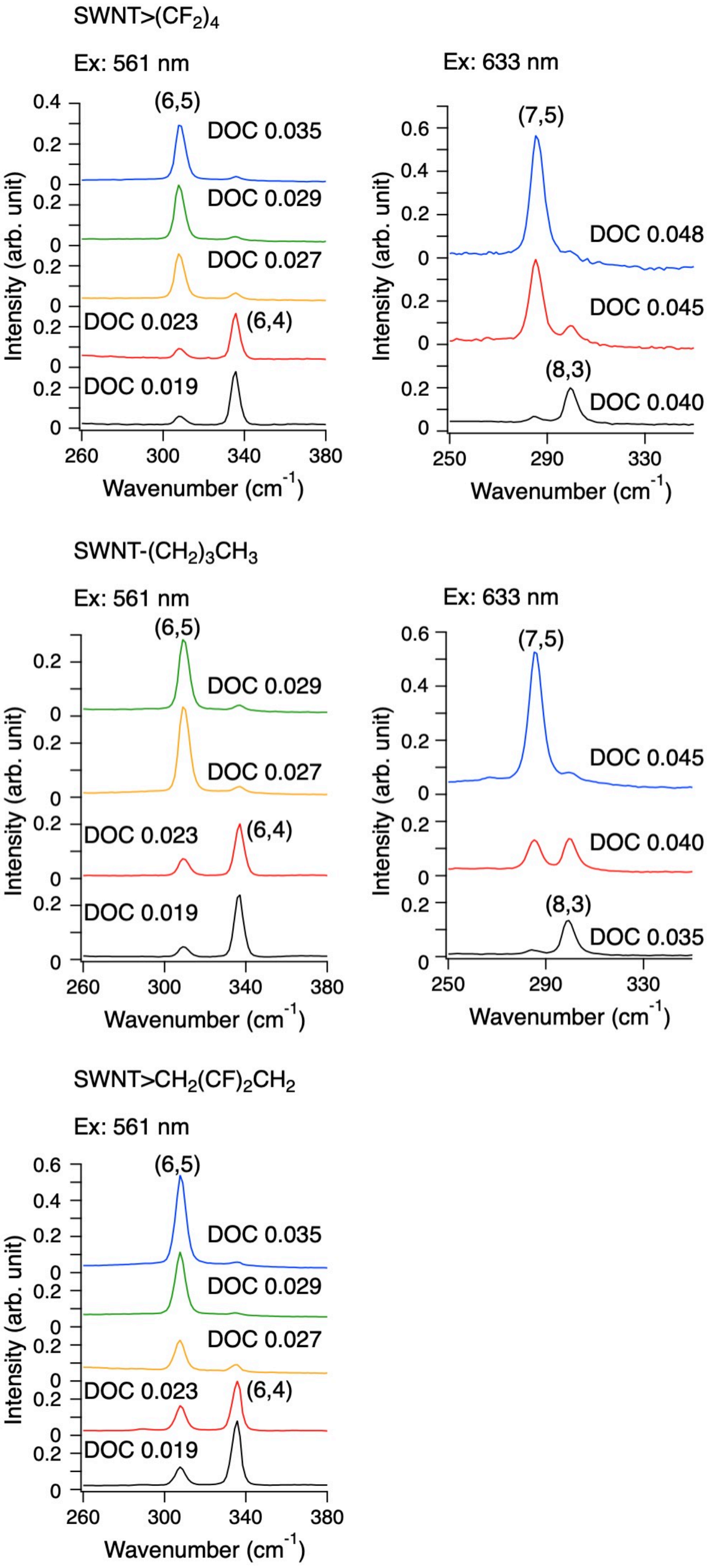

Figure S10. Solution phase Raman spectra of the separated SWNT>$(CF_2)_4$, SWNT-$(CF_2)_3CF_3$, and SWNT>$CH_2(CF_2)_2CH_2$ dispersed in 1 wt% D_2O solution.

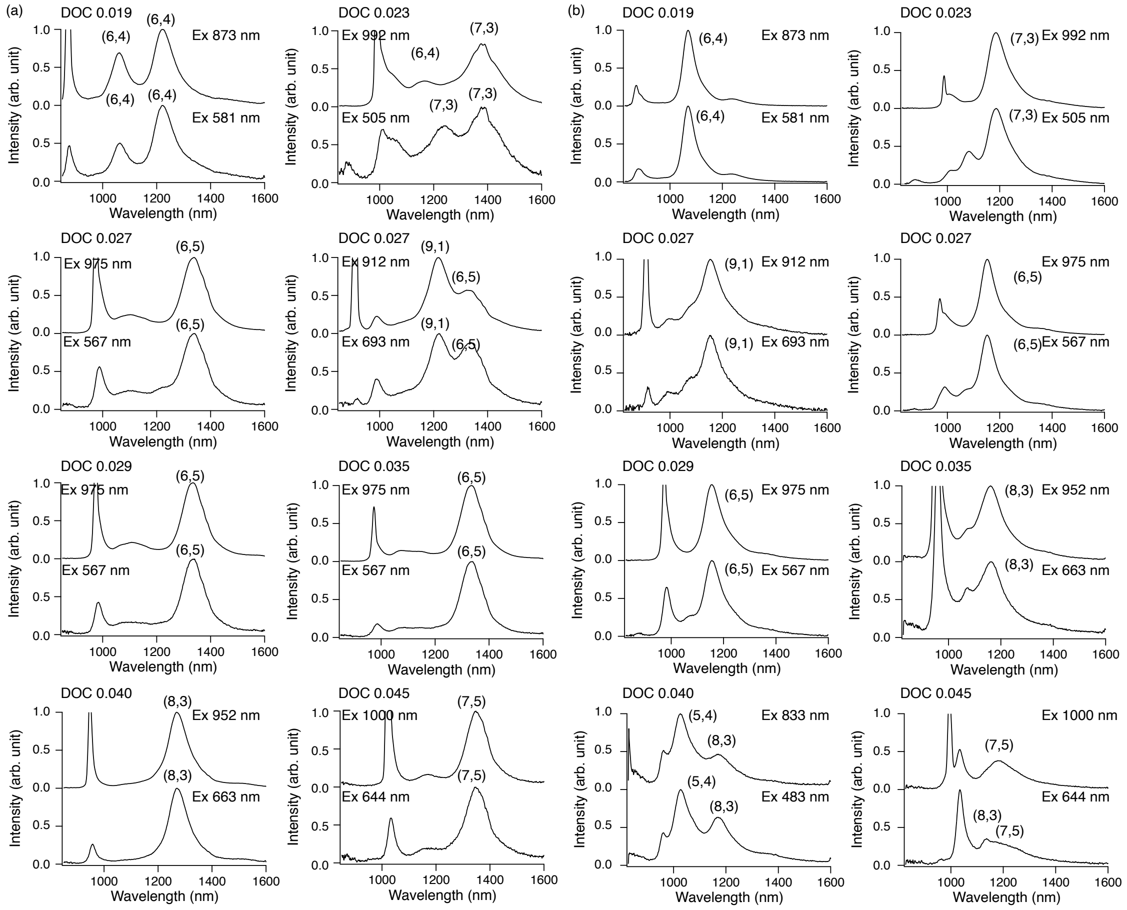

Figure S11. PL spectra of the separated (a) SWNTs>$(CF_2)_4$ and (b) SWNTs-$(CF_2)_3CF_3$ in D_2O containing 1wt% SC.

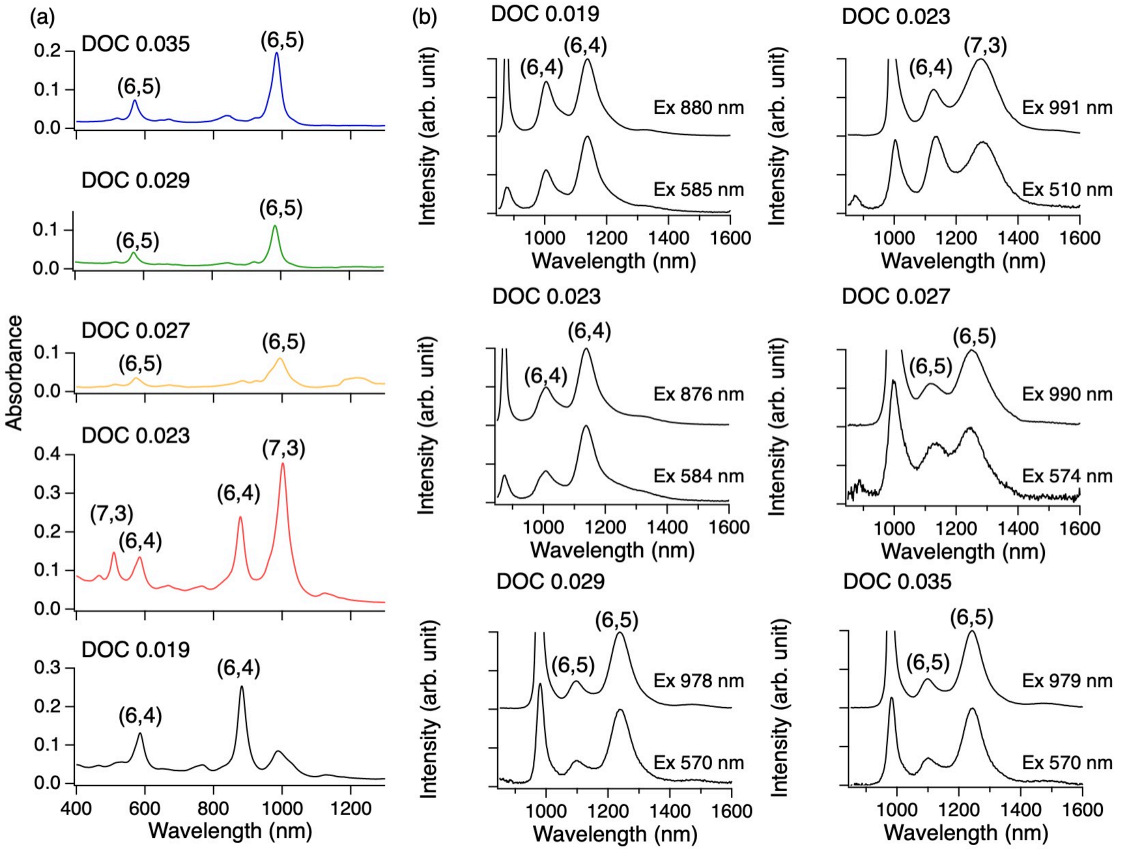

Figure S12. (a) Absorption spectra of the separated SWNT>$CH_2(CF_2)_2CH_2$ dispersed in D_2O containing 1 wt% SC. (b) PL spectra of separated SWNT>$CH_2(CF_2)_2CH_2$ in D_2O containing 1 wt% SC.

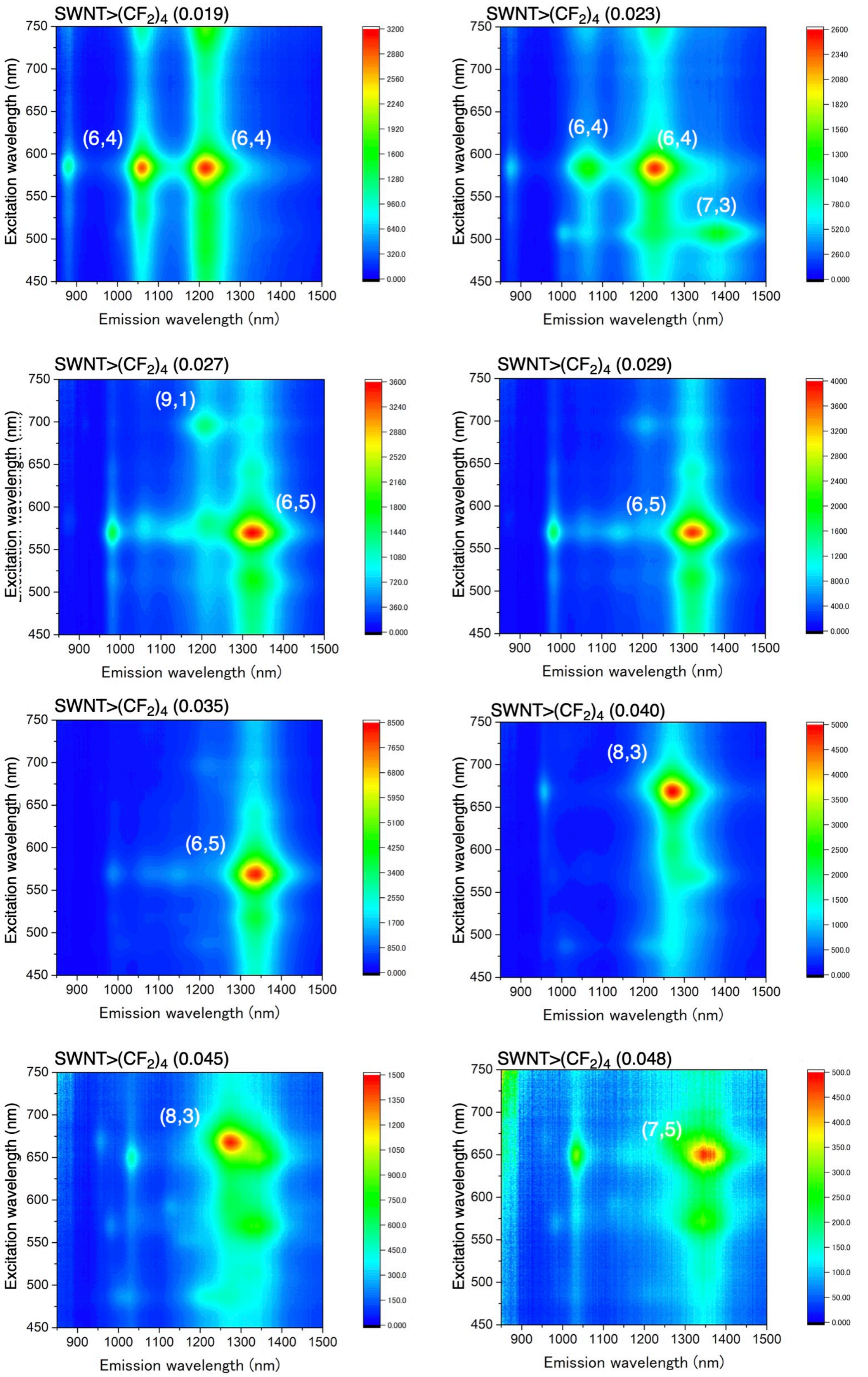

Figure S13. PL contour maps of the separated SWNT>$(CF_2)_4$ dispersed in D_2O containing 1 wt% SC.

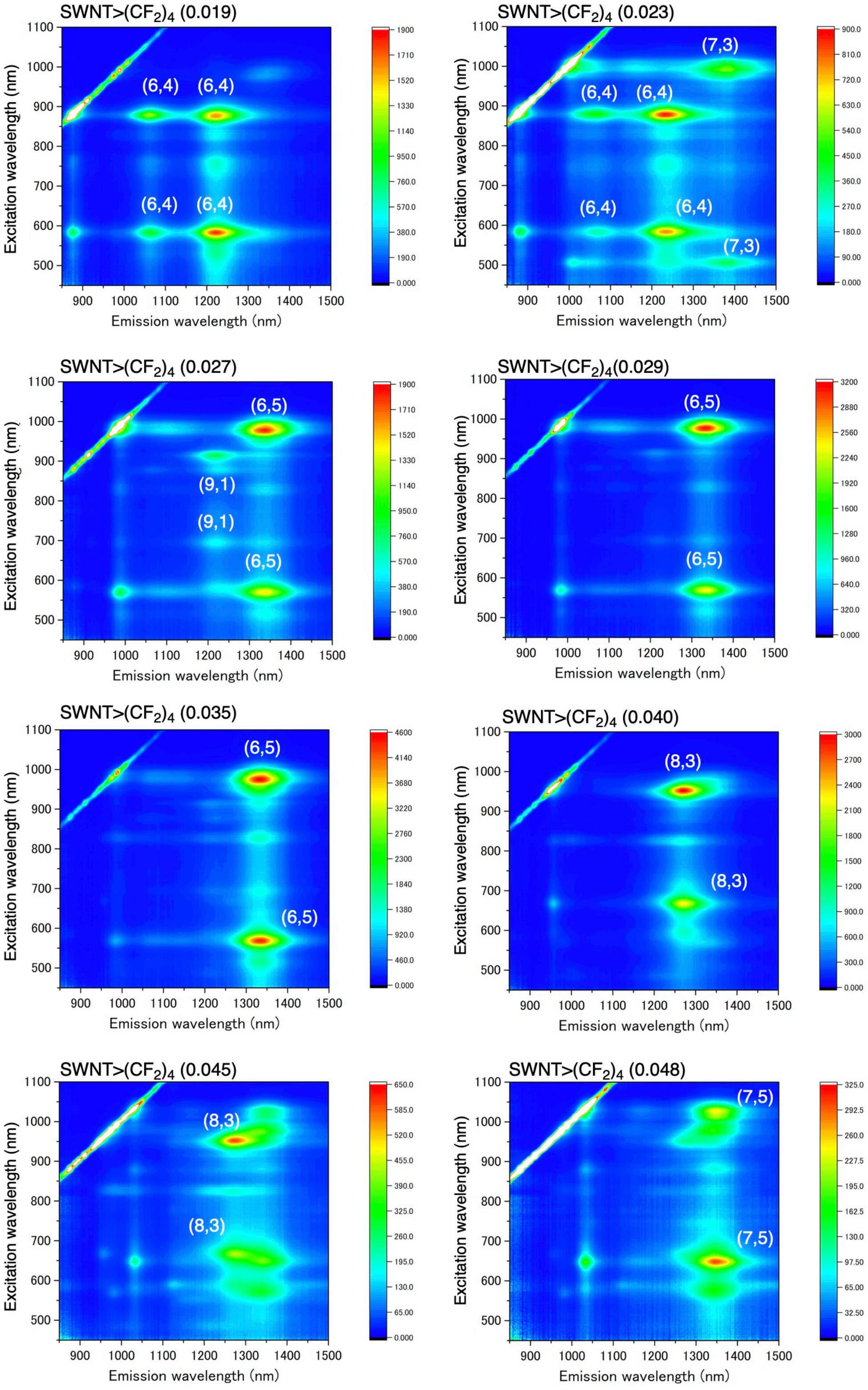

Figure S14. PL contour maps of the separated SWNT>$(CF_2)_4$ dispersed in D_2O containing 1 wt% SC.

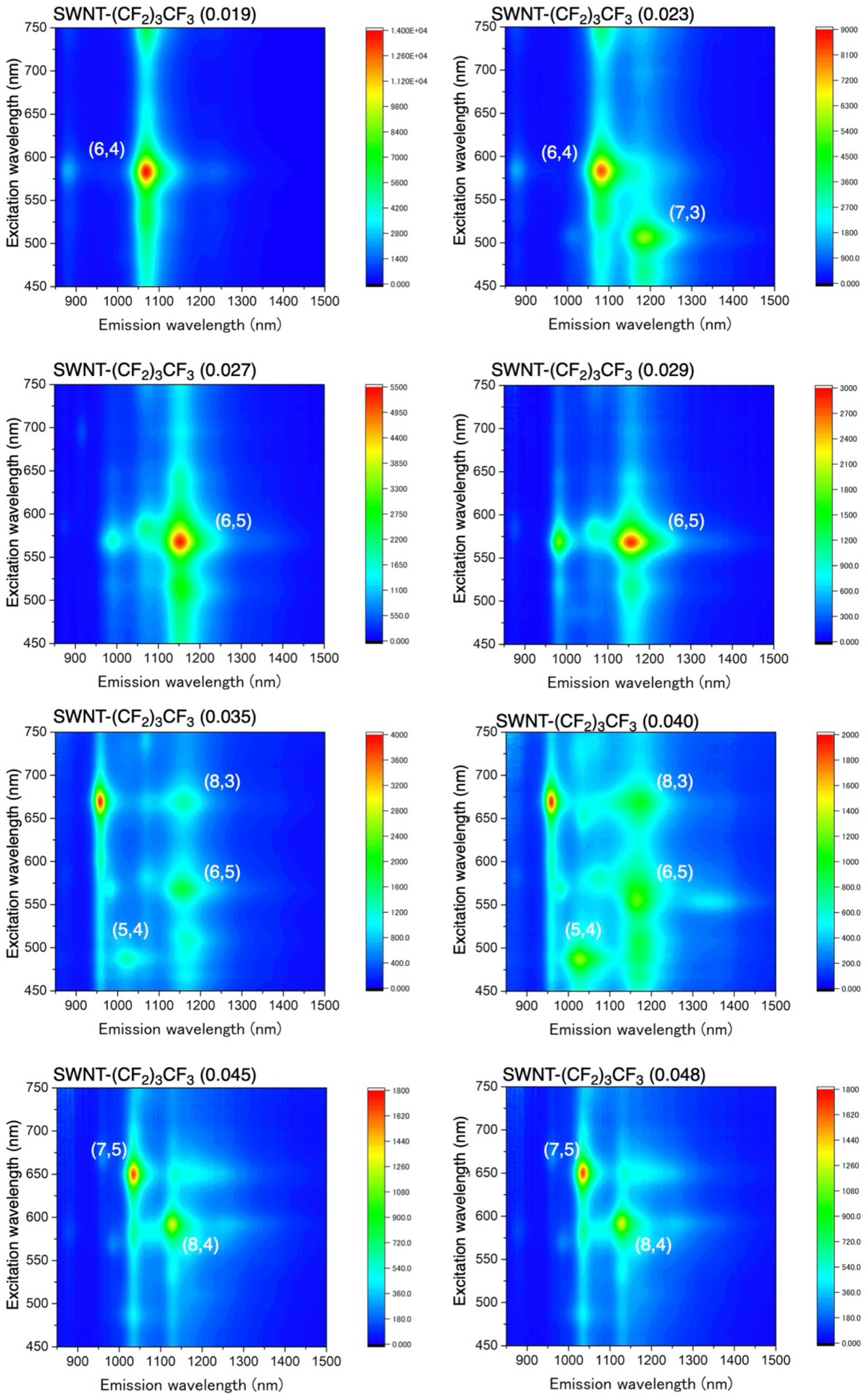

Figure S15. PL contour maps of the separated SWNT-$(CF_2)_3CF_3$ dispersed in D_2O containing 1 wt% SC.

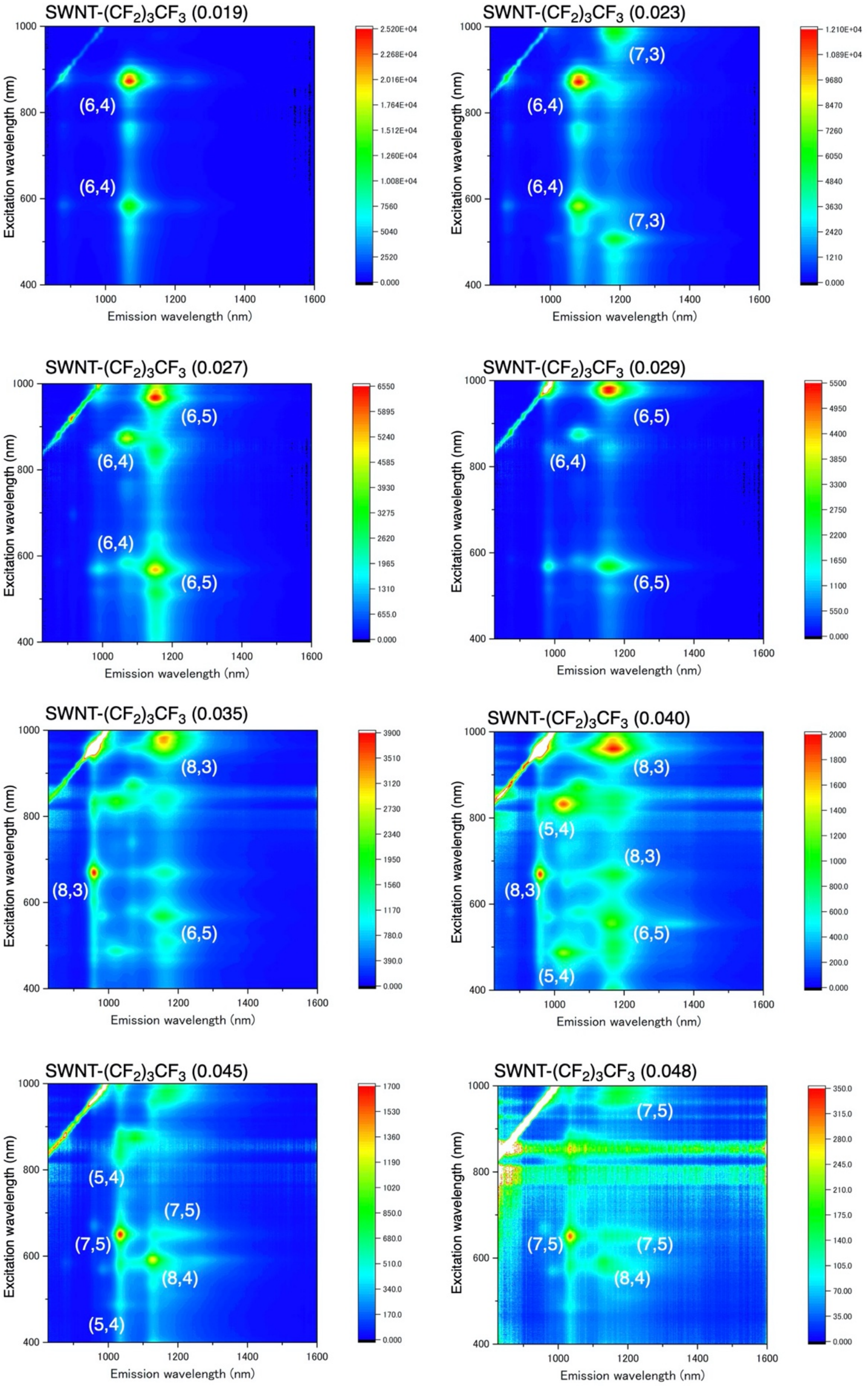

Figure S16. PL contour maps of the separated SWNT-$(CF_2)_3CF_3$ dispersed in D_2O containing 1 wt% SC.

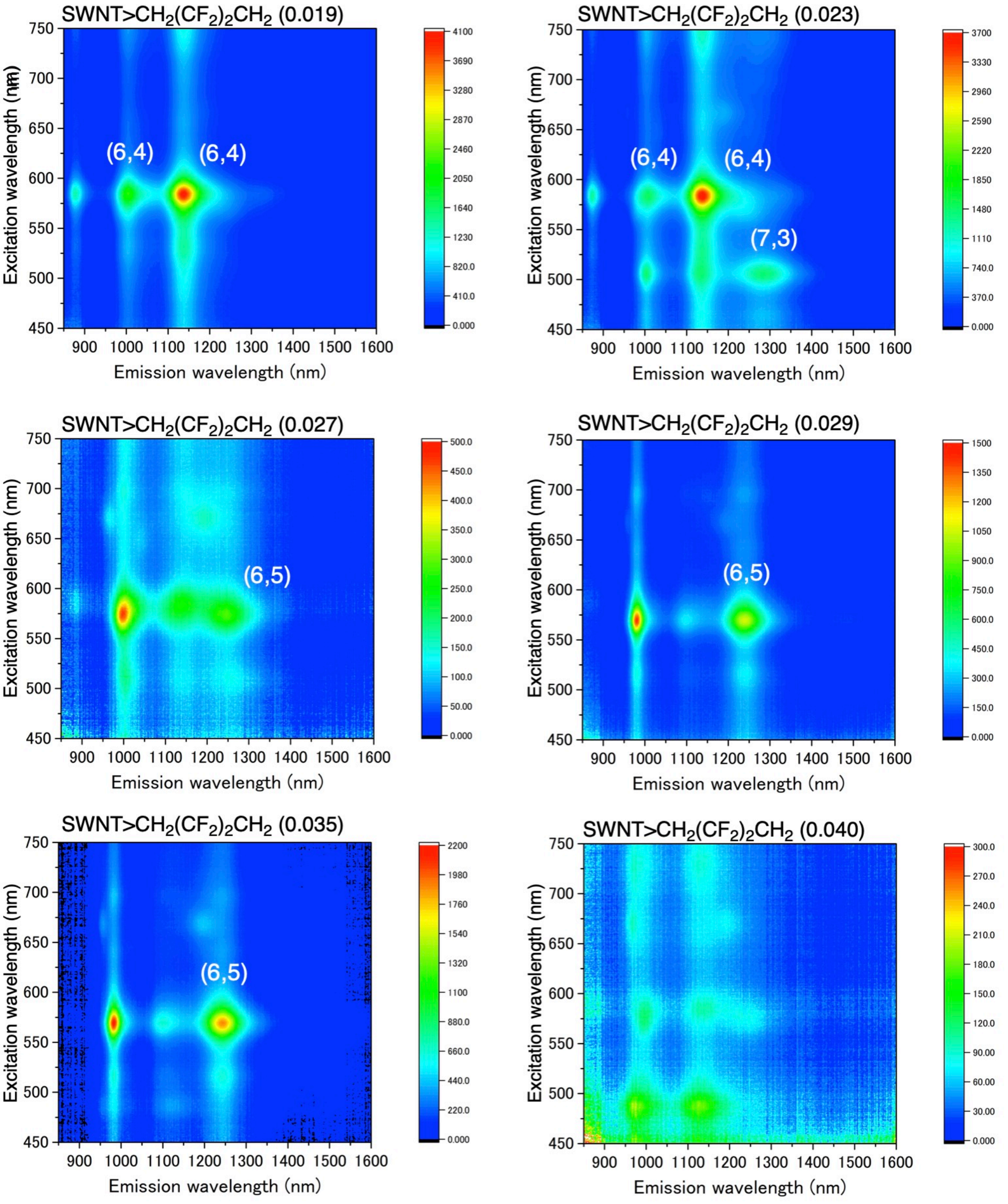

Figure S17. PL contour maps of the separated SWNT>$CH_2(CF_2)_2CH_2$ dispersed in D_2O containing 1 wt% SC.

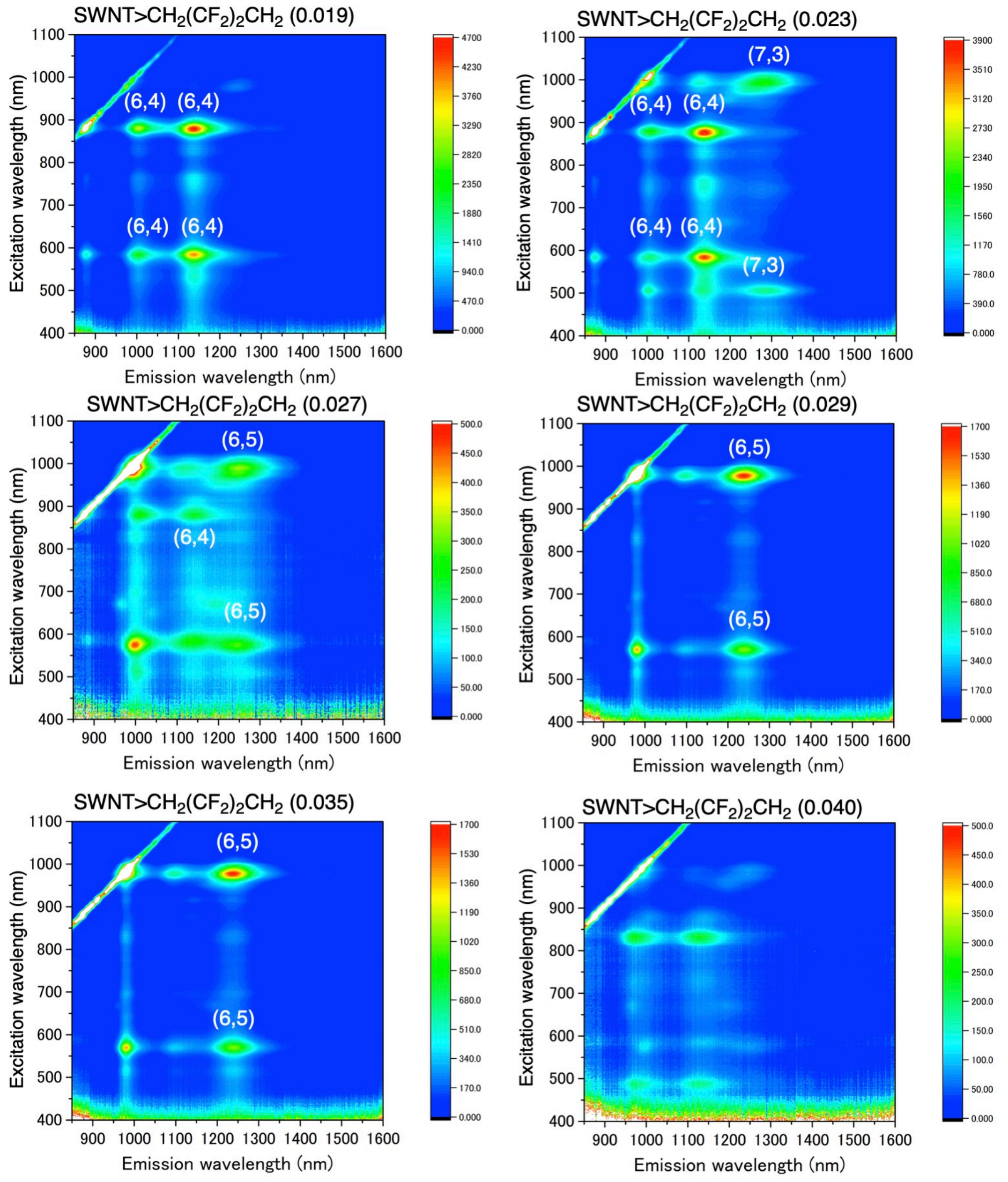

Figure 18. PL contour maps of the separated SWNT>$CH_2(CF_2)_2CH_2$ dispersed in D_2O containing 1 wt% SC.

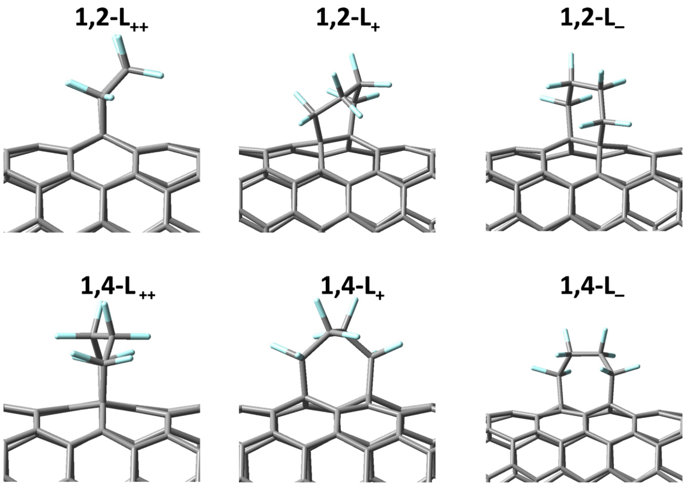

Figure S19. Optimized partial structures of (6,5) SWNT>$(CF_2)_4$ (L_{++}: L_{87}; L_{+}: L_{27}; L_{-}: L_{-33}). Color code: C grey; F blue.

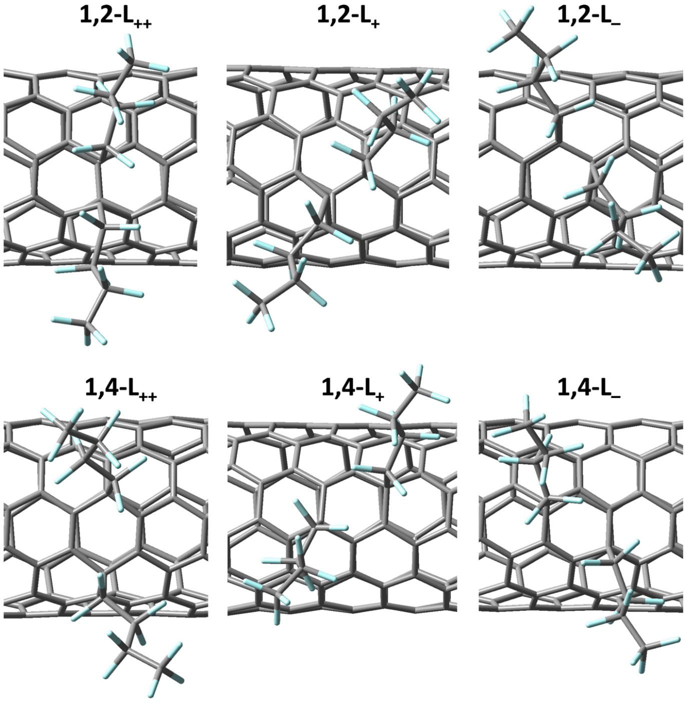

Figure S20. Optimized partial structures of (6,5) SWNT-$[(CF_2)_3CF_3]_2$ (L_{++}: L_{87}; L_{+}: L_{27}; L_{-}: L_{-33}). Color code: C grey; F blue.

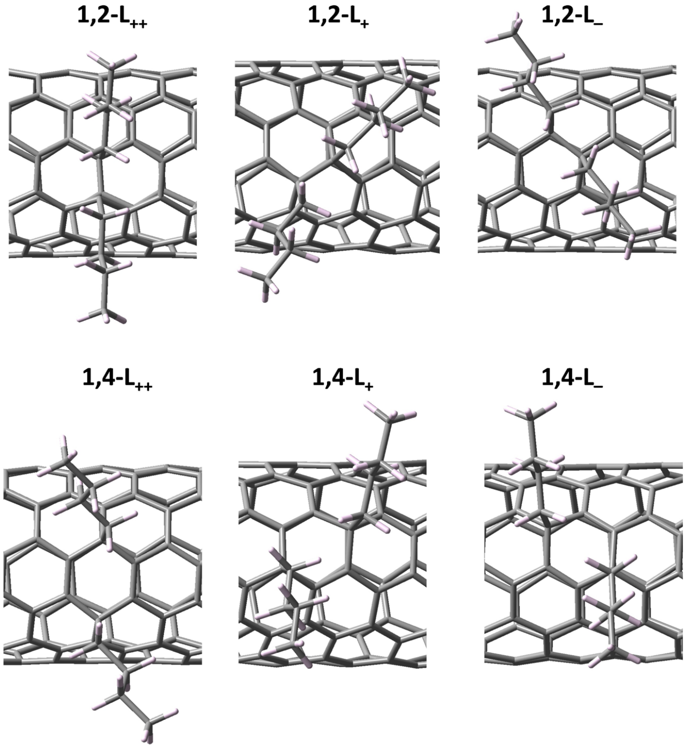

Figure S21. Optimized partial structures of (6,5) SWNT-$[(CH_2)_3CH_3]_2$ (L_{++}: L_{87}; L_{+}: L_{27}; L_{-}:L_{-33}). Color code: C grey; H pink.

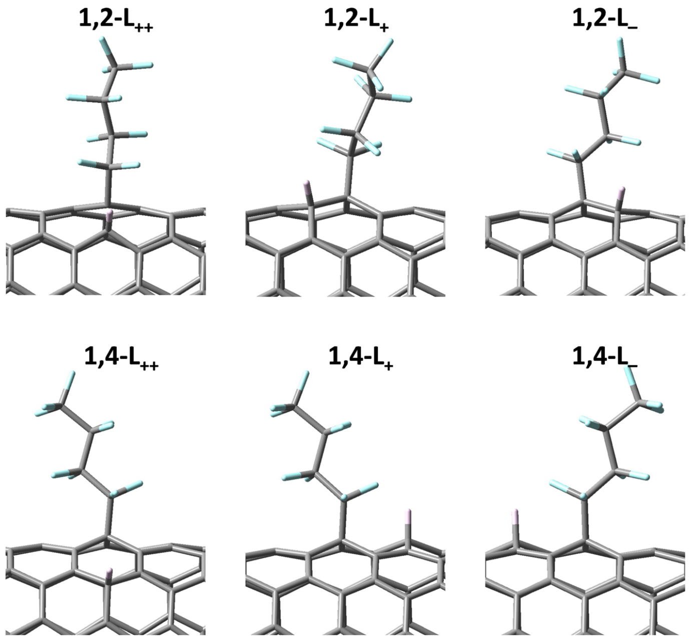

Figure S22. Optimized partial structures of $CF_3(CF_2)_3$-(6,5) SWNT-H (L_{++}: L_{87}; L_{+}: L_{27}; L_{-}: L_{-33}). Color code: C grey; F blue; H pink.

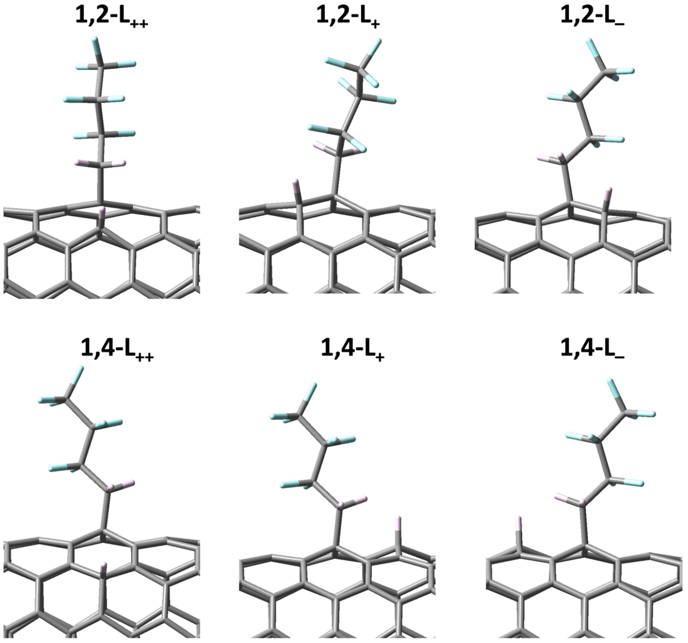

Figure S23. Optimized partial structures of $CF_3(CF_2)_2CH_2$-(6,5) SWNT-H (L_{++}: L_{87}; L_+: L_{27}; $L_–$: $L_{–33}$). Color code: C grey; F blue; H pink.

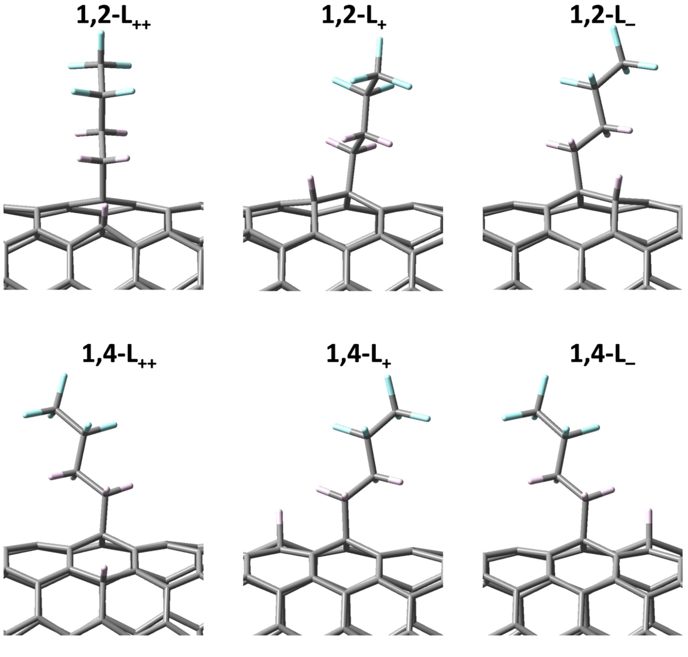

Figure S24. Optimized partial structures of $CF_3CF_2(CH_2)_2$-(6,5) SWNT-H (L_{++}: L_{87}; L_+: L_{27}; $L_–$: $L_{–33}$). Color code: C grey; F blue; H pink.

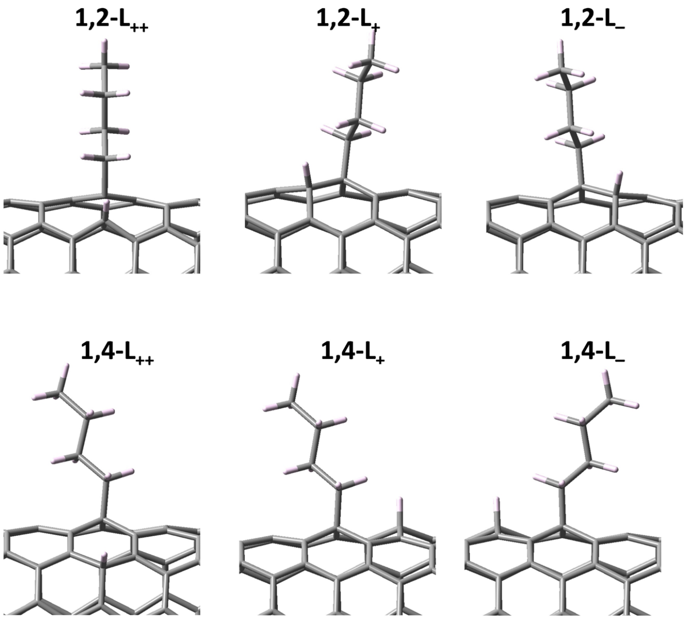

Figure S25. Optimized partial structures of $CH_3(CH_2)_3$-(6,5) SWNT-H (L_{++}: L_{87}; L_+: L_{27}; $L_–$: $L_{–33}$). Color code: C grey; H pink.

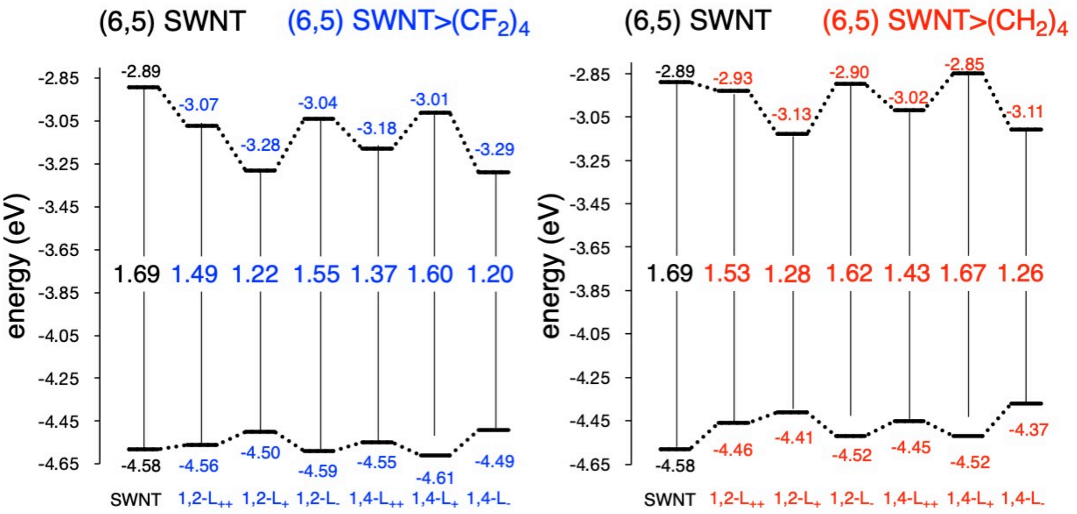

Figure S26. Calculated HOMO and LUMO energy level of (6,5) SWNTs, (6,5) SWNT>$(CF_2)_4$, and (6,5) SWNT>$(CH_2)_4$.

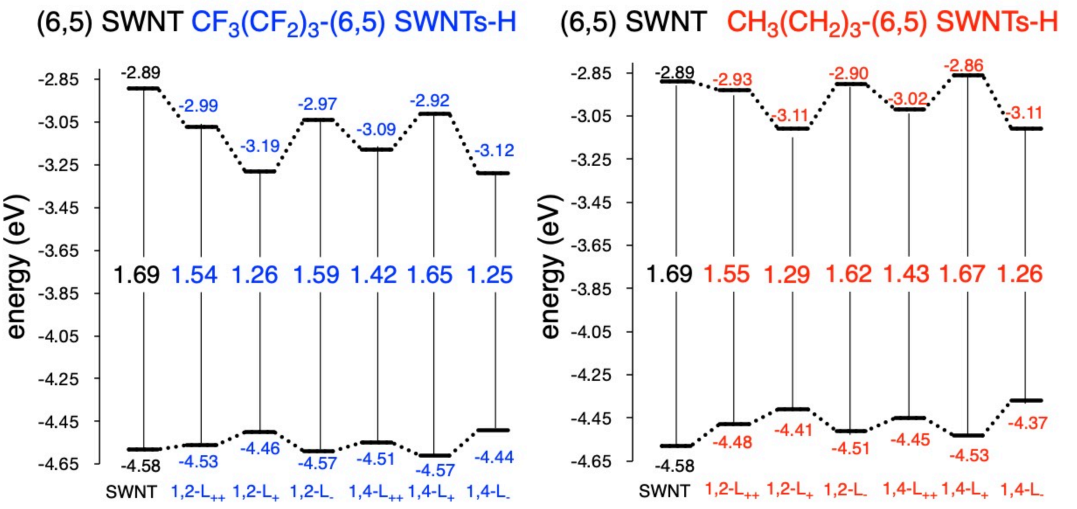

Figure S27. Calculated HOMO and LUMO energy level of (6,5) SWNTs, $CF_3(CF_2)_3$-(6,5) SWNT-H, and $CH_3(CH_2)_3$-(6,5) SWNT-H.

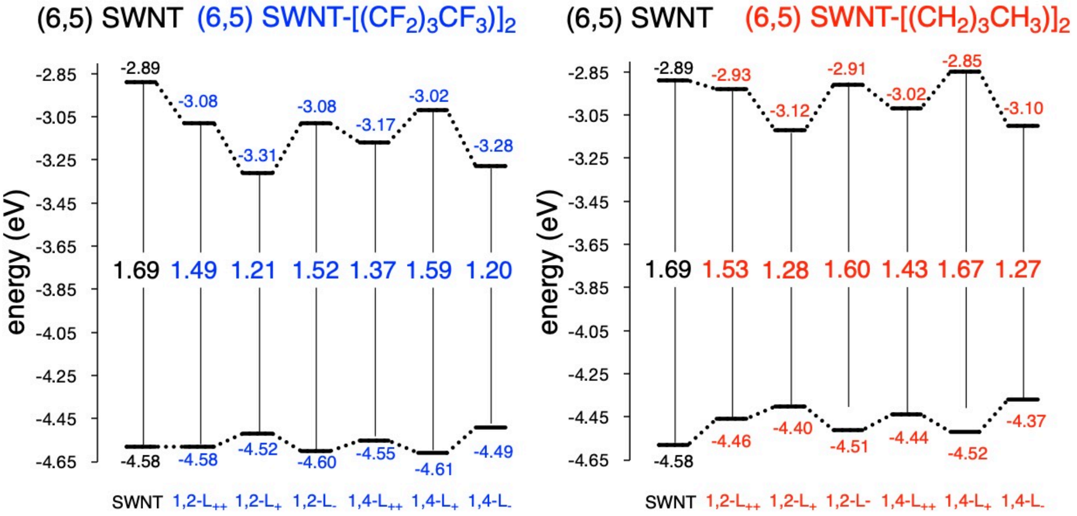

Figure S28. Calculated HOMO and LUMO energy level of (6,5) SWNTs, (6,5) SWNT-$[(CF_2)_3CF_3]_2$, and (6,5) SWNT-$[(CH_2)_3CH_3]_2$.

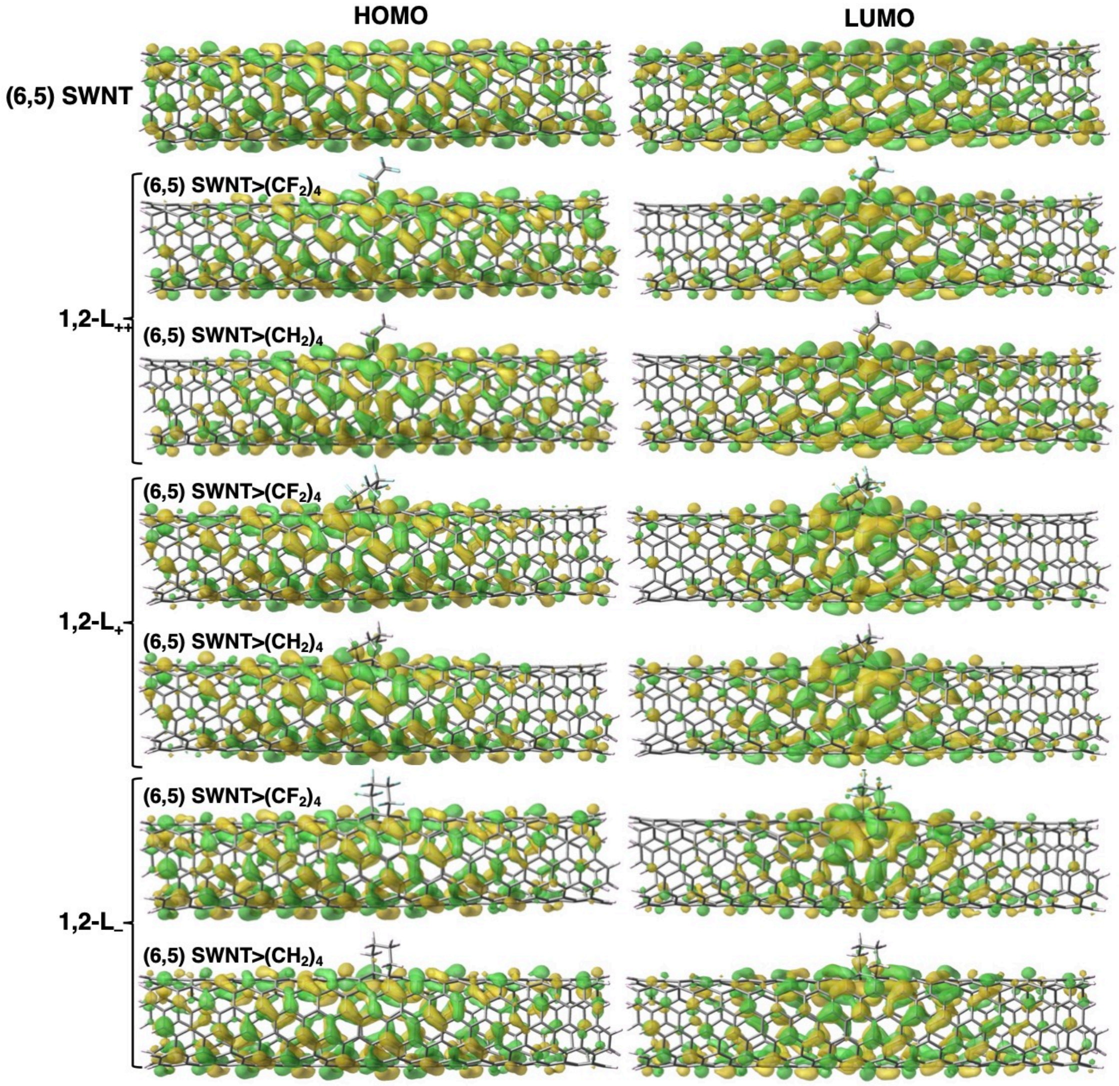

Figure S29. HOMO and LUMO distributions (isovalue=0.01) of (6,5) SWNT, (6,5) SWNT>$(CF_2)_4$, and (6,5) SWNT>$(CH_2)_4$. Color code: C grey; F blue; H pink. (continue)

(continued)

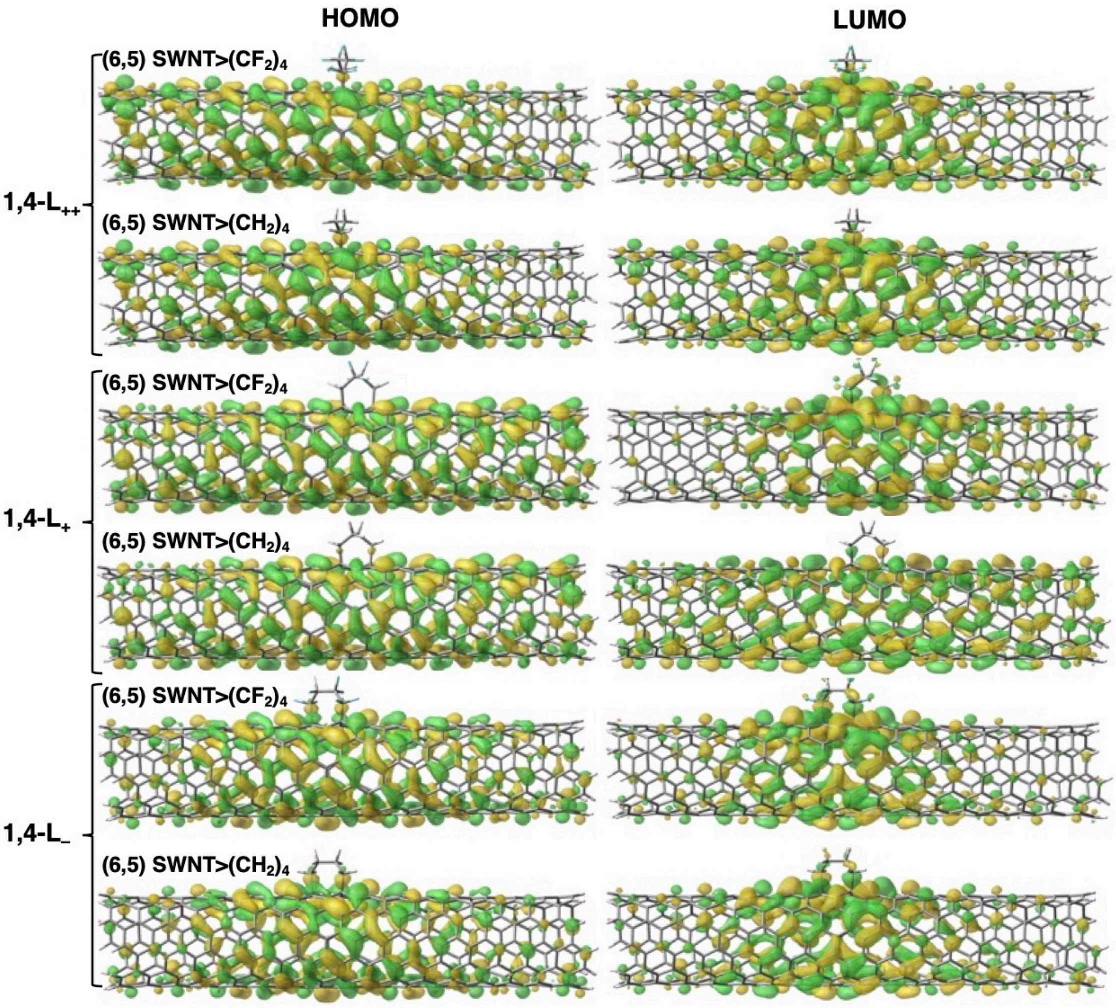

Figure S29. HOMO and LUMO distributions (isovalue = 0.01) of (6,5) SWNT, (6,5) SWNT>(CF_2)$_4$, and (6,5) SWNT>(CH_2)$_4$. Color code: C grey; F blue; H pink.

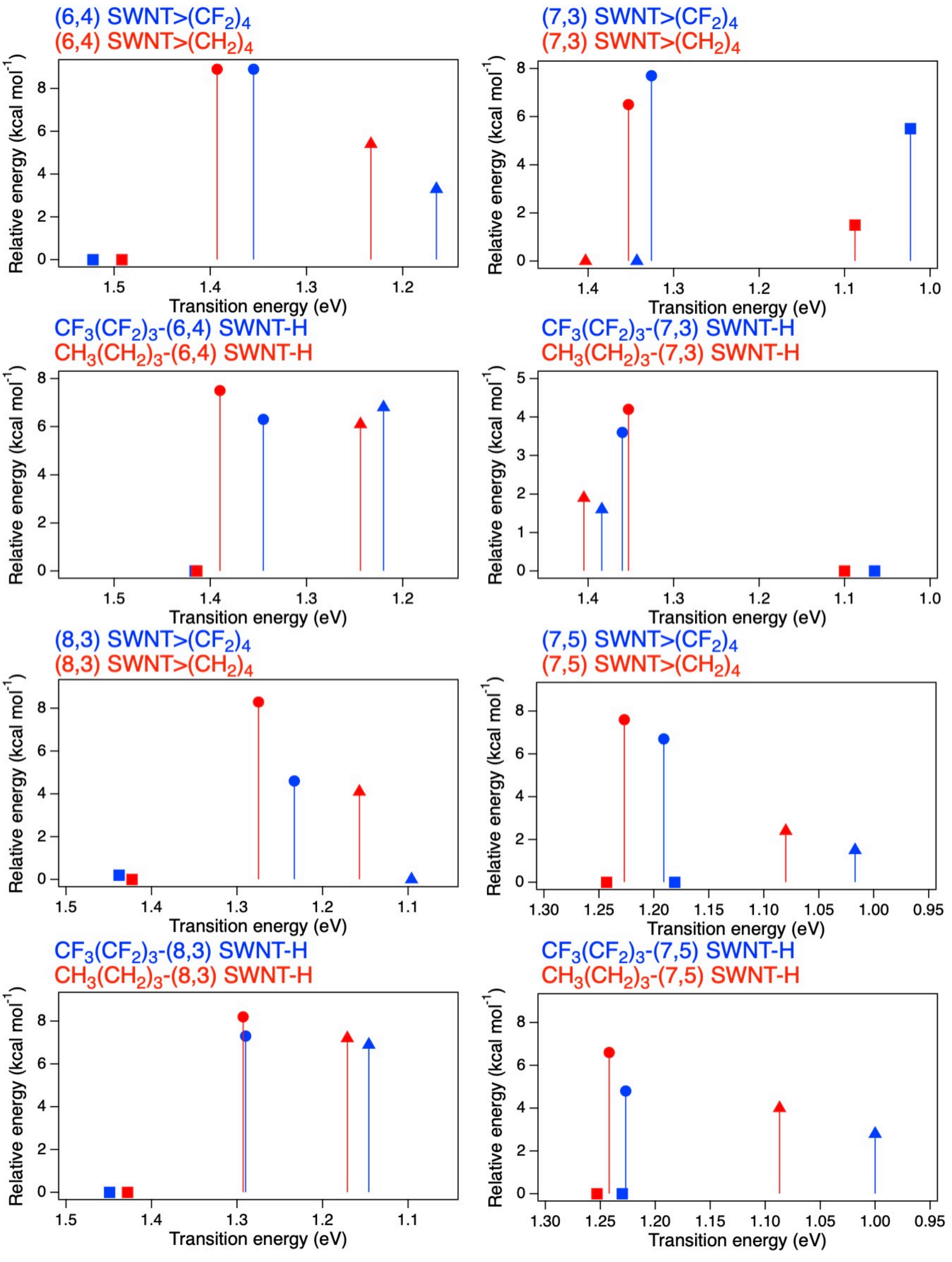

Figure S30. Calculated transition energy (eV) and relative energy (kcal mol^{-1}) of model molecules of SWNT>$(CH_2)_4$, SWNT>$(CF_2)_4$, $CH_3(CH_2)_3$-SWNT-H, and $CF_3(CF_2)_3$-SWNT-H for (7,3), (6,4), (8,3) and (7,5) SWNT.1,2-addition sites are classified according to their bond angles of bonds with respect to the SWNT axis, marked as (●): L++, (■): L+, and (▲): L–.

Table S1. PL wavelength of functionalised (6,5) SWNTs before the separation.

	SWNT-$(CH_2)_3CH_3$	SWNT-$(CH_2)_3CF_3$	SWNT-$(CH_2)_2CF_2CF_3$	SWNT-$CH_2(CF_2)_2CF_3$	SWNT-$(CF_2)_3CF_3$
E_{11}^{*} PL (nm)	1100	1104	1118	1119	1150
E_{11}^{**} PL (nm)	1231	1240	1243	-	-
D/G_{561nm}	0.19 (Br)<br>0.21 (I)	0.20	0.18	0.09	0.13

	SWNT>$(CH_2)_4$[1]	SWNT>$CH_2(CF_2)_2CH_2$	SWNT>$(CF_2)_4$
E11* PL (nm)	-	-	-
E11** PL (nm)	1228	1246	1319
D/G_{561nm}	0.44	0.06	0.17

Table S2. PL wavelength of functionalised SWNTs after the separation.

	(6,4)	(7,3)	(9,1)	(6,5)	(8,3)	(5,4)	(7,5)
SWNT>$(CF_2)_4$	1064<br>1222	1373<br>1389	1207	1320	1269	-	1345
SWNT>$(CH_2)_4$[1]	1115	1265	-	1229	1170	-	1248
SWNT-$CH_2(CF_2)_2CH_2$	1003<br>1139	1288	-	1237	-	-	-
SWNT-$(CF_2)_3CF_3$	1068	1185	1153	1152	1164	1027	1178
SWNT-$(CH_2)_3CH_3$ (300˚C)[2]	1119	1251	-	1213	1160	-	1237
$CH_3(CH_2)_3$-SWNTs-H[2]	1015	1128<br>1213	-	1104<br>1231	1076	-	1136
SWNTs-$(CH_2)_5CH_3$[3]	1049	1150	1086	1096	1124	-	1174
SWNTs-$(CF_2)_5CF_3$[3]	1082	1190	1128	1155	1169	1027	1206

Table S3. Values of spin densities of the $CF_3CF_2CF_2CF_2$ radical, $CF_3CF_2CF_2CH_2$ radical, $CF_3CF_2CH_2CH_2$ radical, $CF_3CH_2CH_2CH_2$ radical, and $CH_3CH_2CH_2CH_2$ radical at the level of UB3LYP/3-21G (Isovalue = 0.004).

$CF_3CF_2CF_2CF_2$ radical	$CF_3CF_2CF_2CH_2$ radical	$CF_3CF_2CH_2CH_2$ radical	$CF_3CH_2CH_2CH_2$ radical	$CH_3CH_2CH_2CH_2$ radical
0.7880	1.0756	1.1109	1.1104	1.1054

Spin density maps

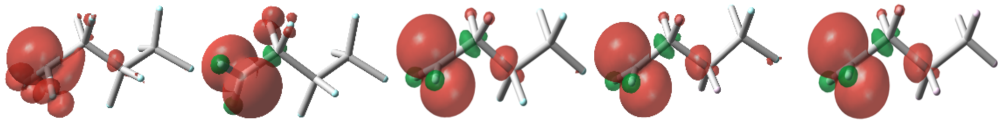

Table S4. HOMO and LUMO energy levels (in eV) of pristine and SWNT>$(CF_2)_4$, SWNT>$(CH_2)_4$, $CH_3(CH_2)_3$-SWNT-H, and $CF_3(CF_2)_3$-SWNT-H for (6,4), (7,3), (8,3), and (7,5) SWNT.

		LUMO	HOMO	HOMO-LUMO Gap
(6,4) SWNT		–2.87	–4.62	1.76
	1,2-L_{++}	–3.03	–4.61	1.58
(6,4) SWNT>$(CF_2)_4$	1,2-L_{+}	–3.08	–4.63	1.55
	1,2-L_{-}	–3.19	–4.55	1.36
	1,2-L_{++}	–2.87	–4.48	1.61
(6,4) SWNT>$(CH_2)_4$	1,2-L_{+}	–2.93	–4.55	1.62
	1,2-L_{-}	–3.01	–4.44	1.43
	1,2-L_{++}	–2.87	–4.50	1.63
$CH_3(CH_2)_3$-(6,4) SWNT-H	1,2-L_{+}	–2.92	–4.56	1.64
	1,2-L_{-}	–3.01	–4.44	1.44
	1,2-L_{++}	–2.93	–4.57	1.64
H-(6,4) SWNT-C_4F_9	1,2-L_{+}	–3.00	–4.61	1.60
	1,2-L_{-}	–3.10	–4.51	1.41
(7,3) SWNT		–2.94	–4.57	1.64
	1,2-L_{++}	–3.05	–4.59	1.53
(7,3) SWNT>$(CF_2)_4$	1,2-L_{+}	–3.31	–4.52	1.21
	1,2-L_{-}	–3.03	–4.60	1.57
	1,2-L_{++}	–2.94	–4.49	1.55
(7,3) SWNT>$(CH_2)_4$	1,2-L_{+}	–3.16	–4.43	1.27
	1,2-L_{-}	–2.91	–4.52	1.61
	1,2-L_{++}	–2.94	–4.50	1.56
$CH_3(CH_2)_3$-(7,3) SWNT-H	1,2-L_{+}	–3.15	–4.43	1.28
	1,2-L_{-}	–2.92	–4.53	1.61
	1,2-L_{++}	–2.99	–4.55	1.56
$CF_3(CF_2)_3$-(7,3) SWNT-H	1,2-L_{+}	–3.23	–4.49	1.25
	1,2-L_{-}	–2.98	–4.58	1.60

(continue)

(continued)

		LUMO	HOMO	HOMO-LUMO Gap
(8,3) SWNT		–3.02	–4.52	1.49
$(8,3)\ SWNT>(CF_2)_4$	1,2-L_{++}	-3.07	-4.41	1.34
	1,2-L_{+}	-3.11	-4.55	1.43
	1,2-L_{-}	-3.16	-4.6	1.44
$(8,3)\ SWNT>(CH_2)_4$	1,2-L_{++}	-2.95	-4.59	1.64
	1,2-L_{+}	-2.98	-4.45	1.47
	1,2-L_{-}	-3.03	-4.53	1.50
$CH_3(CH_2)_3$-(8,3) SWNT-H	1,2-L_{++}	-3.23	-4.5	1.28
	1,2-L_{+}	-2.98	-4.46	1.48
	1,2-L_{-}	-3.02	-4.53	1.51
$CF_3(CF_2)_3$-(8,3) SWNT-H	1,2-L_{++}	-3.06	-4.41	1.35
	1,2-L_{+}	-3.03	-4.52	1.48
	1,2-L_{-}	-3.09	-4.58	1.48
(7,5) SWNT		–3.02	–4.52	1.49
$(7,5)\ SWNT>(CF_2)_4$	1,2-L_{++}	–3.14	–4.52	1.38
	1,2-L_{+}	–3.14	–4.53	1.39
	1,2-L_{-}	–3.28	–4.47	1.19
$(7,5)\ SWNT>(CH_2)_4$	1,2-L_{++}	–3.03	–4.44	1.40
	1,2-L_{+}	–3.05	–4.48	1.43
	1,2-L_{-}	–3.15	–4.39	1.25
$CH_3(CH_2)_3$-(7,5) SWNT-H	1,2-L_{++}	–3.03	–4.45	1.41
	1,2-L_{+}	–3.04	–4.48	1.44
	1,2-L_{-}	–3.14	–4.39	1.25
$CF_3(CF_2)_3$-(7,5) SWNT-H	1,2-L_{++}	–3.08	–4.49	1.41
	1,2-L_{+}	–3.09	–4.52	1.43
	1,2-L_{-}	–3.21	–4.44	1.23

Table S5. Transition energies (in nm) of (6,5) SWNT>$(CF_2)_4$, >$CH_2(CF_2)_2CH_2$, >$(CH_2)_4$, -$[(CF_2)_3CF_3]_2$, -$[(CH_2)_3CH_3]_2$, $CF_3(CF_2)_3$-(6,5)SWNT-H, $CF_3(CF_2)_2CH_2$-(6,5)SWNT-H, $CF_3CF_2(CH_2)_2$-(6,5)SWNT-H, and $CH_3(CH_2)_3$-(6,5)SWNT-H adducts.

Binding configuration	1,2-L_{++}	1,2-L_{+}	1,2-L_{-}	1,4-L_{++}	1,4-L_{+}	1,4-L_{-}
SWNTs	Transition energy (nm)					
(6,5) SWNT>$(CF_2)_4$	958	1188	944	1045	853	1198
(6,5) SWNT >$CH_2(CF_2)_2CH_2$	940	1142	901	-	-	-
(6,5) SWNT>$(CH_2)_4$[1]	934	1120	885	998	852	1120
(6,5) SWNT-$[(CF_2)_3CF_3]_2$	963	1202	967	1052	855	1191
(6,5) SWNT-$[(CH_2)_3CH_3)]_2$	934	1125	892	999	853	1122
$CF_3(CF_2)_3$-(6,5) SWNT-H	928	1138	905	1009	876	1138
$CF_3(CF_2)_2CH_2$-(6,5) SWNT-H	924	1121	894	998	850	1132
$CF_3CF_2(CH_2)_2$-(6,5) SWNT-H	925	1117	891	998	861	1127
$CH_3(CH_2)_3$-(6,5) SWNT-H[2]	922	1106	880	992	855	1121

Table S6. Relative energies (ΔE, in kcal mol^{-1}) of (6,5) SWNT>$(CF_2)_4$, >$CH_2(CF_2)_2CH_2$, >$(CH_2)_4$, -$[(CF_2)_3CF_3]_2$, -$[(CH_2)_3CH_3]_2$, $CF_3(CF_2)_3$-(6,5)SWNT-H, $CF_3(CF_2)_2CH_2$-(6,5)SWNT-H, $CF_3CF_2(CH_2)_2$-(6,5)SWNT-H, and $CH_3(CH_2)_3$-(6,5)SWNT-H adducts.

Binding configuration	1,2-L_{++}	1,2-L_{+}	1,2-L_{-}	1,4-L_{++}	1,4-L_{+}	1,4-L_{-}
SWNTs	Relative energy (ΔE, kcal mol^{-1})					
(6,5) SWNT>$(CF_2)_4$	8.3	4.0	0	13.6	4.0	10.7
(6,5) SWNT >$CH_2(CF_2)_2CH_2$	13.1	5.8	0	-	-	-
(6,5) SWNT>$(CH_2)_4$[1]	7.9	3.5	0	18.2	6.4	11.7
(6,5) SWNT-$[(CF_2)_3CF_3]_2$	18.4	31.3	24.6	0	8.1	8.2
(6,5) SWNT-$[(CH_2)_3CH_3)_2$	7.5	13.7	8.6	0	3.8	5.0
$CF_3(CF_2)_3$-(6,5) SWNT-H	1.8	1.1	0	4.6	5.0	8.3
$CF_3(CF_2)_2CH_2$-(6,5) SWNT-H	5.2	1.1	0	5.8	6.7	10.5
$CF_3CF_2(CH_2)_2$-(6,5) SWNT-H	4.3	1.6	0	5.4	5.9	9.6
$CH_3(CH_2)_3$-(6,5) SWNT-H[2]	4.8	1.9	0	6.1	6.6	10.3

Table S7. Relative energies (ΔE, in kcal mol^{-1}) and transition energies (in eV/nm) of SWNT>$(CF_2)_4$, SWNT>$(CH_2)_4$, $CH_3(CH_2)_3$-SWNT-H, and $CF_3(CF_2)_3$-SWNT-H for (6,4), (7,3), (8,3), and (7,5) SWNT.

Binding configuration	ΔE	Transition energies		Oscillator strength	ΔE	Transition energies		Oscillator strength
		eV	nm	*f*		eV	nm	*f*
	(6,4) SWNT>$(CH_2)_4$				(6,4) SWNT>$(CF_2)_4$			
1,2-L_{++}	8.9	1.393	890	0.445	8.9	1.355	915	0.462
1,2-L_{+}	0	1.371	905	0.036	0	1.283	966	0.028
		1.492	831	0.116		1.522	815	0.113
1,2-L_{-}	5.4	1.233	1006	0.802	3.3	1.165	1064	0.730
	$CH_3(CH_2)_3$-(6,4) SWNT-H				$CF_3(CF_2)_3$-(6,4) SWNT-H			
1,2-L_{++}	7.5	1.39	892	0.034	6.3	1.345	922	0.033
1,2-L_{+}	0	1.414	877	0.433	0	1.416	876	0.398
		1.528	812	0.206		1.522	815	0.218
1,2-L_{-}	6.1	1.244	997	0.813	6.8	1.22	1017	0.784
	(7,3) SWNT>$(CH_2)_4$				(7,3) SWNT>$(CF_2)_4$			
1,2-L_{++}	6.5	1.353	916	0.728	7.7	1.326	935	0.668
1,2-L_{+}	1.5	1.088	1139	0.629	5.5	1.023	1211	0.380
		1.104	1123	0.192		1.04	1192	0.377
1,2-L_{-}	0	1.403	883	0.421	0	1.343	924	0.359
	$CH_3(CH_2)_3$-(7,3) SWNT-H				$CF_3(CF_2)_3$-(7,3) SWNT-H			
1,2-L_{++}	4.2	1.363	909	0.734	3.6	1.36	912	0.591
1,2-L_{+}	0	1.1	1127	0.634	0	1.065	1164	0.426
1,2-L_{-}	1.9	1.405	882	0.375	1.6	1.384	896	0.448

(continue)

(continued)

Binding configuration	ΔE	Transition energies		Oscillator strength	ΔE	Transition energies		Oscillator strength
		eV	nm	*f*		eV	nm	*f*
	$(8,3)$ SWNT>$(CH_2)_4$				$(8,3)$ SWNT>$(CF_2)_4$			
1,2-L_{++}	8.3	1.275	972	0.624	4.6	1.233	1005	0.589
1,2-L_{+}	0	1.423	871	0.226	0.2	1.438	862	0.472
1,2-L_{-}	4.1	1.157	1072	0.753	0	1.096	1131	0.704
	$CH_3(CH_2)_3$-(8,3) SWNT-H				$CF_3(CF_2)_3$-(8,3) SWNT-H			
1,2-L_{++}	8.2	1.293	959	0.625	7.3	1.29	961	0.630
1,2-L_{+}	0	1.428	868	0.226	0	1.449	856	0.288
1,2-L_{-}	7.2	1.171	1059	0.761	6.9	1.146	1082	0.748
	(7,5) SWNT>$(CH_2)_4$				(7,5) SWNT>$(CF_2)_4$			
1,2-L_{++}	7.6	1.227	1010	0.343	6.7	1.191	1041	0.366
		1.234	1005	0.448		1.205	1029	0.381
1,2-L_{+}	0	1.243	997	0.259	0	1.181	1050	0.165
1,2-L_{-}	2.4	1.08	1149	0.845	1.5	1.017	1219	0.759
	$CH_3(CH_2)_3$-(7,5) SWNT-H				$CF_3(CF_2)_3$-(7,5) SWNT-H			
1,2-L_{++}	6.6	1.242	998	0.725	4.8	1.227	1011	0.265
						1.245	996	0.522
1,2-L_{+}	0	1.253	989	0.253	0	1.23	1008	0.234
1,2-L_{-}	4	1.087	1141	0.862	2.8	1.06	1170	0.823

Supplementary Methods

Preparation of 1,4-diodo-2,2,3,3-tetrafluorobutane[3,4]

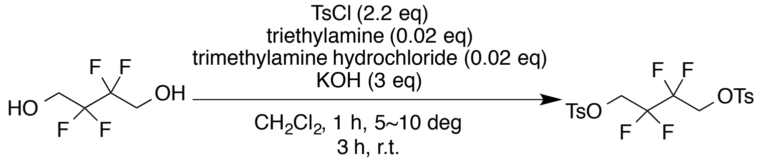

A solution of 2,2,3,3-tatrafluoro-1,4-butanediol (1.71 g, 10 mmol), KOH (1.73 g, 30 mmol), triethylamine (27.4 μL, 0.20 mmol), and trimethylamine hydrochloride (0.20 g, 0.20 mmol) in dichloromethane (20 mL) in a 100 mL round-bottom flask was stirred at 5-10 °C. Tosyl chloride (4.2 g, 22 mmol) in dichloromethane (20 mL) was added dropwise to a stirred mixture and then stirred. After 1h, the reaction mixture was stirred for 3 h at room temperature. 1 M HCl (20 mL) was added to the reaction mixture and the resulting organic layer was washed with water (20 mL×3). The organic layer was dried over Na_2SO_4, filtered, and evaporated. 2,2,3,3-tetrafluoro-1,4-bis(4-methylbenzenesulfonate) was obtained as a white solid (3.90 g, 83%). mp: 88.9-90.5 °C; R_f=0.53 (*n*-hexane/EtOAc 1:1); ^1H NMR (400 MHz, $CDCl_3$): δ 2.47 (s, 6H), 4.36 (tt, J = 12.26 Hz, J = 2.28 Hz, 4H), 7.38 (d, J = 8.48 Hz, 4H), 7.79 (d, J = 8.48 Hz, 4H); $^{13}C\{^1H\}$ NMR (100 MHz, $CDCl_3$): δ 21.89, 63.89 (t, J = 27.6 Hz), 114.373 (tt, J = 254.6, 33.3 Hz), 128.23, 130.29, 131.85, 146.03; $^{19}F\{^1H\}$ NMR (376 MHz, $CDCl_3$): δ -121.55; FT-IR (neat): 3009.4, 2974.7, 1379.8, 1188.9 cm^{-1}; HRMS (*m/z*): [M^+] calcd. for $C_{18}H_{18}F_4O_6S_2$, 470.0481; found 470.0475.

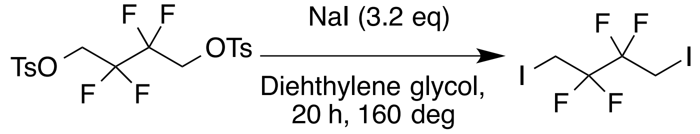

A solution of 2,2,3,3-tetrafluoro-1,4-bis(4-methylbenzenesulfonate) (5.50 g, 11.70 mmol) and NaI (7.89 g, 52.64 mmol) in of diethylene glycol (50 mL) in 100 mL round-bottom flask was stirred at 160°C for 20 h. After cooling, 100 mL of water was added to the reaction mixture. The resulting aqueous layer was extracted with diethyl ether (100 mL×2). The combined organic layer was washed with aq. $NaHSO_3$ (100 mL×2) and activated carbon. Resulted organic layer was dried over $MgSO_4$, filtered, and evaporated to obtain a yellow solid. Column chromatography (SiO_2, hexane) gave 2,2,3,3-tetrafluoro-1,4-diiodobutane (1.18 g, 26%) as white needle crystals. mp 79.5-80.3 °C; ^1H NMR (400 MHz, $CDCl_3$): δ 3.68-3.58 (m, 4H); $^{13}C\{^1H\}$ NMR (100 MHz, $CDCl_3$): δ -3.39 (m), 113.87 (tt, J = 251.1 Hz, J = 37.7 Hz); $^{19}F\{^1H\}$ NMR (376 MHz, $CDCl_3$) δ -107.59; FT-IR: 2979.5, 1160.0 cm^{-1}; HRMS (*m/z*): [M^+] calcd. for $C_4H_4F_4I_2$ 381.8339; found 381.8332.

Column chromatography was carried out using silica gel 60 N (Kanto Chemical CO., INC.). TLC was carried out on silica gel 60 PF254 (Merck KGaA). IR spectra were recorded as neat films between

KBr plates (FT-IR4100; JASCO Corp.). NMR spectra were obtained as dilute solution in $CDCl_3$ (JNM-ECX 400P spectrometer; JEOL Ltd.). The chemical shifts were reported relative to internal TMS or CF_3COOH. High resolution mass spectra were recorded (JMS-700(2) MStation; JEOL Ltd.) using EI ionization mode.

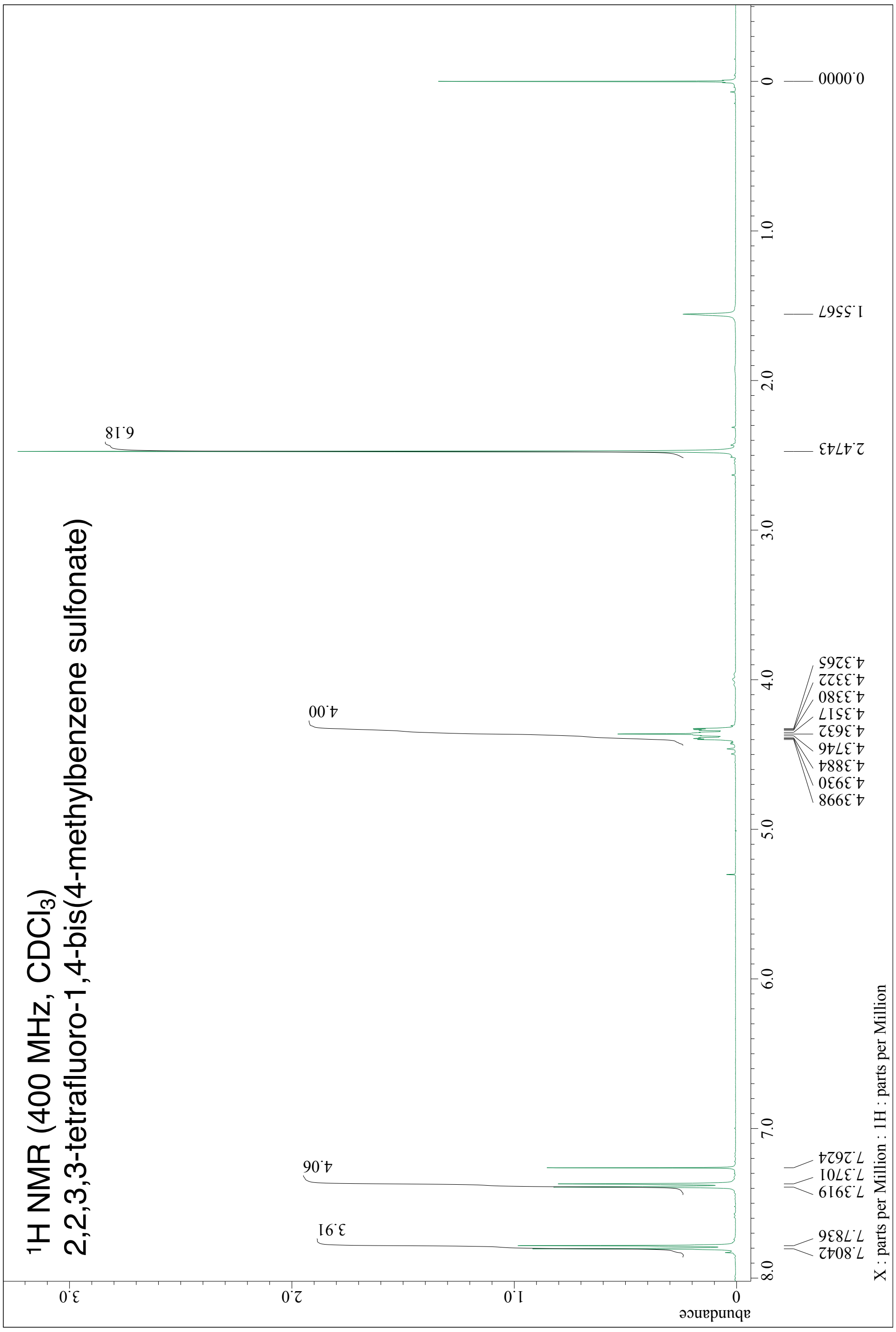

Figure S31. ¹H NMR spectra of 2,2,3,3-tetrafluoro-1,4-bis(4-methylbenzene sulfonate) in $CDCl_3$.

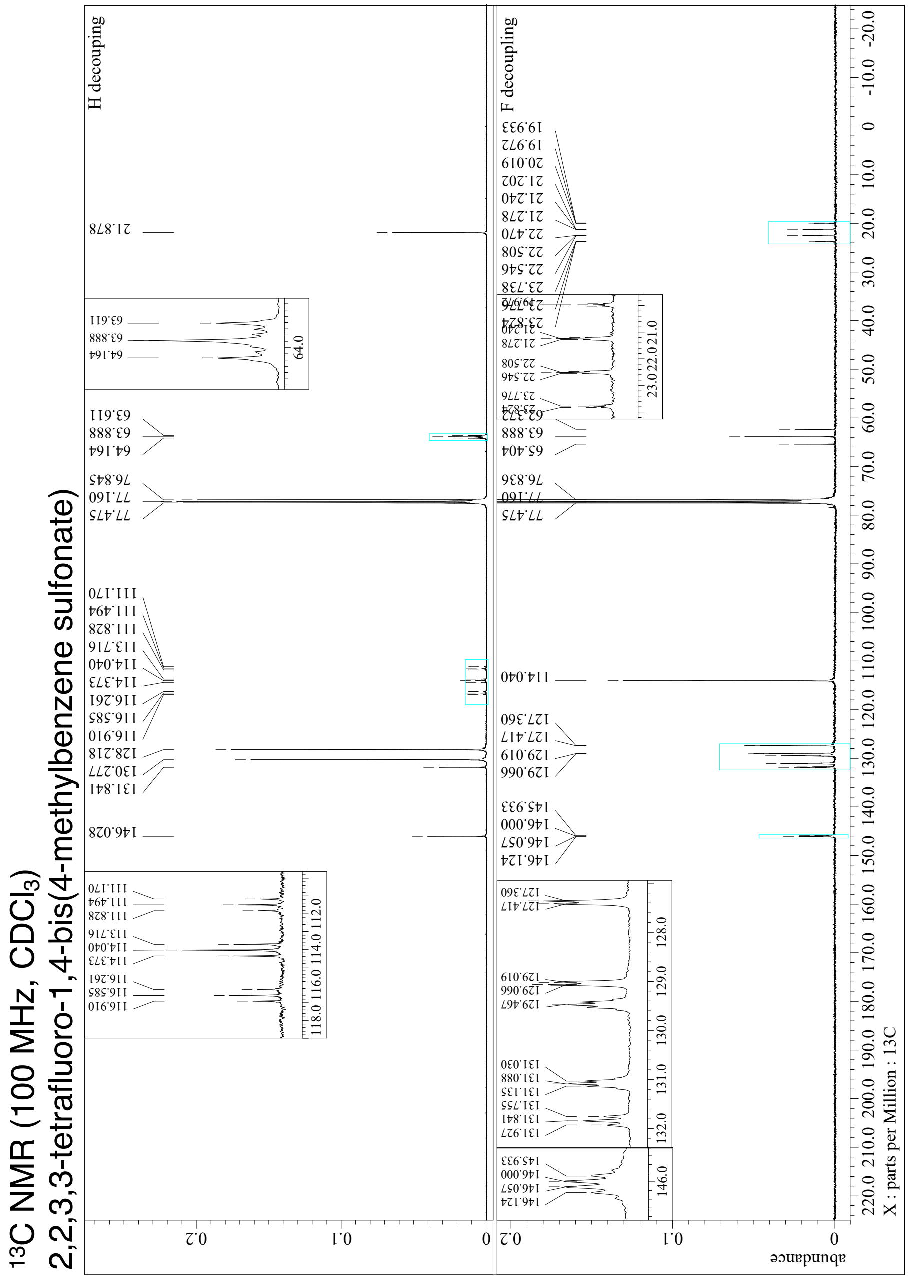

Figure S32. $^{13}C\{^1H\}$ and $^{13}C\{^{19}F\}$ NMR spectra of 2,2,3,3-tetrafluoro-1,4-bis(4-methylbenzene sulfonate) in $CDCl_3$.

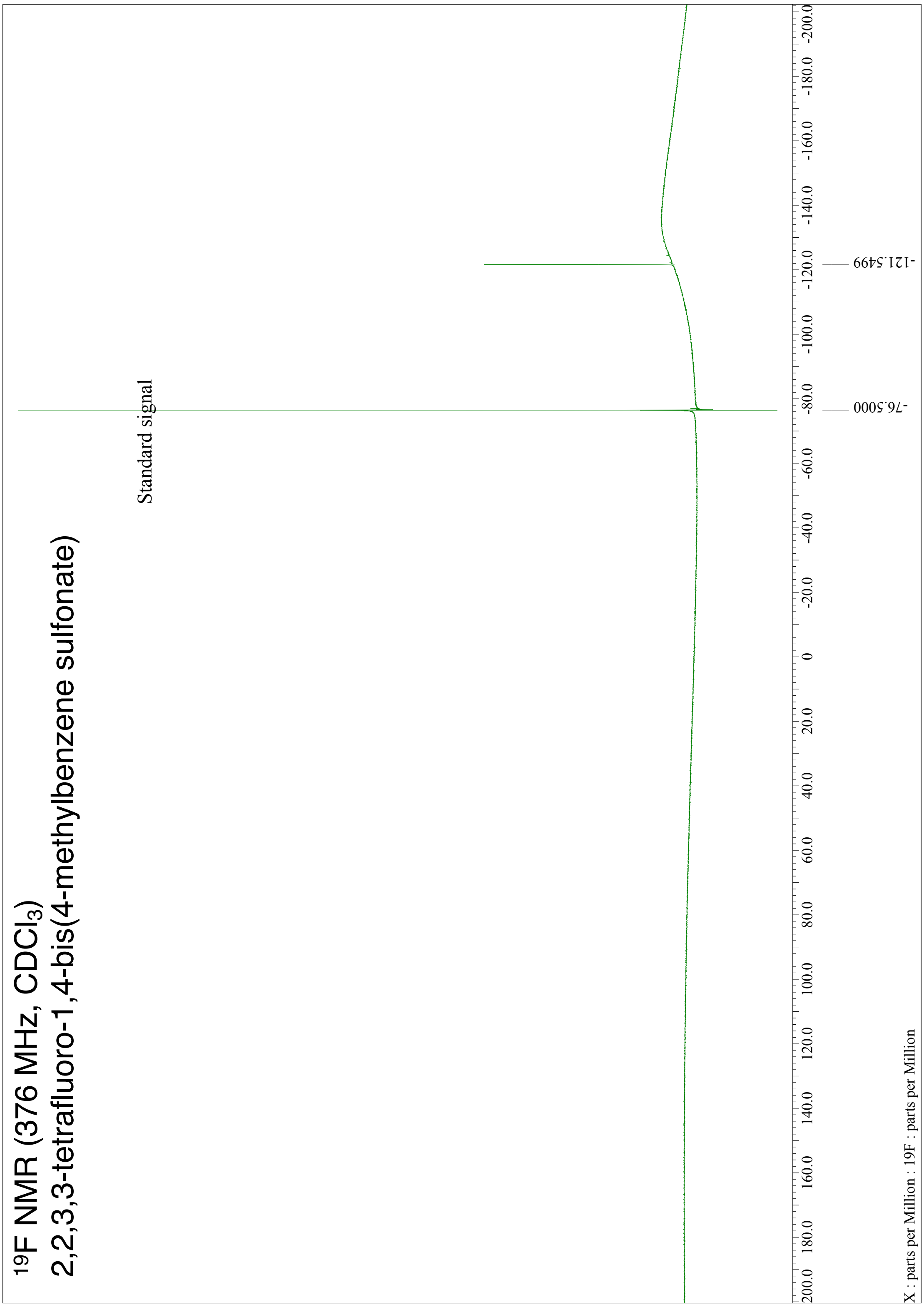

Figure S33. $^{19}F\{^{1}H\}$ NMR spectra of 2,2,3,3-tetrafluoro-1,4-bis(4-methylbenzene sulfonate) in $CDCl_3$.

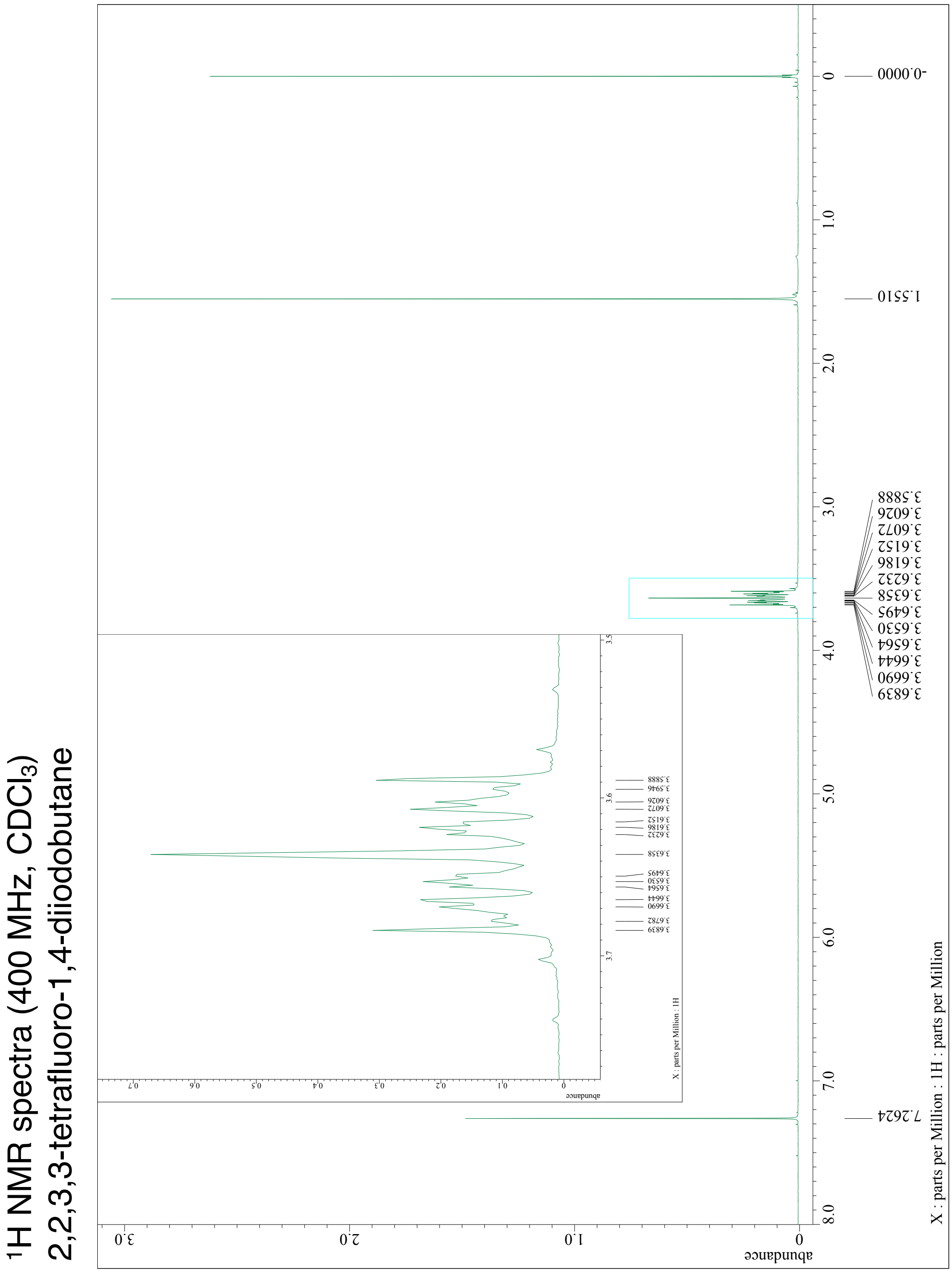

Figure S34. ^{1}H NMR spectra of 2,2,3,3-tetrafluoro-1,4-diiodobutane in $CDCl_3$.

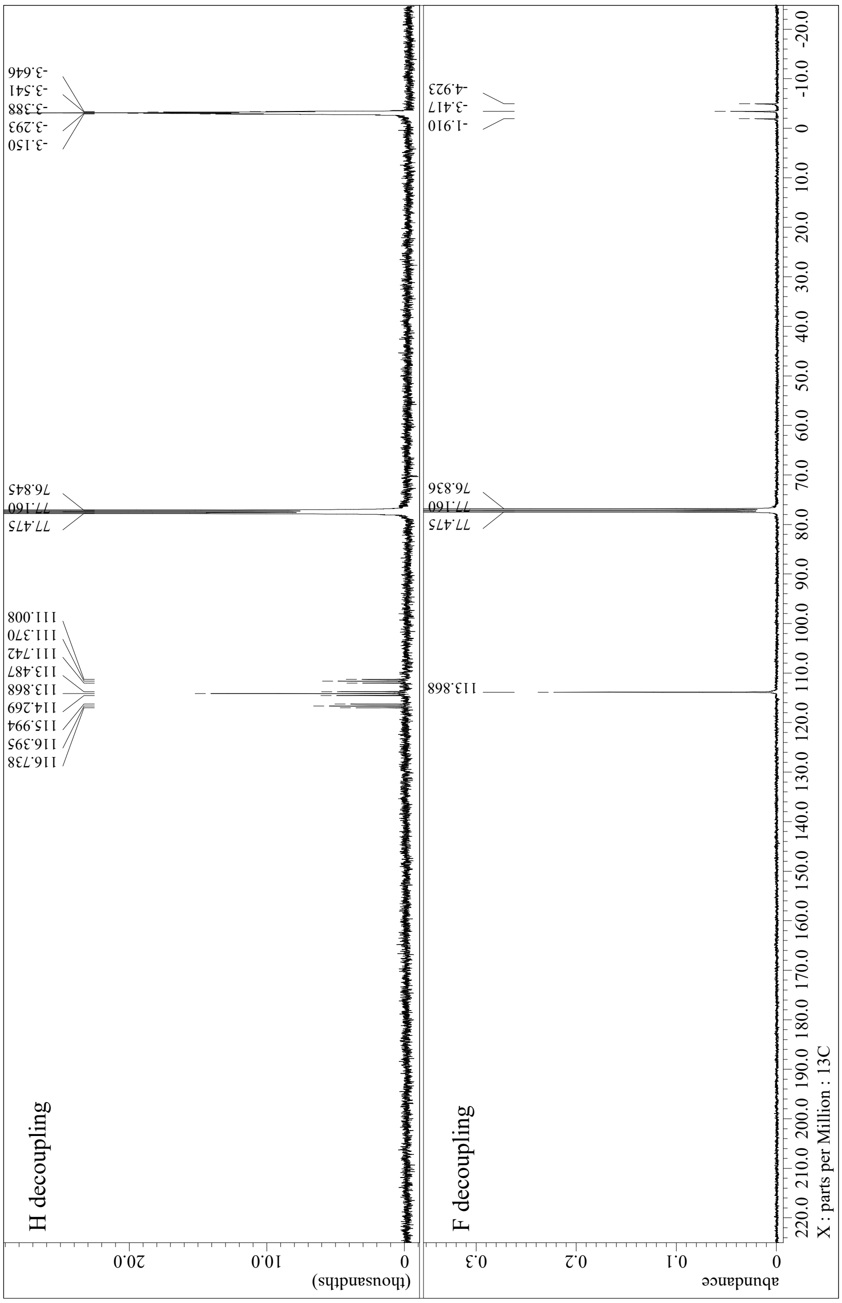

Figure S35. $^{13}C\{^{19}F\}$ and $^{13}C\{^{1}H\}$ NMR spectra of 2,2,3,3-tetrafluoro-1,4-diiodobutane in $CDCl_3$.

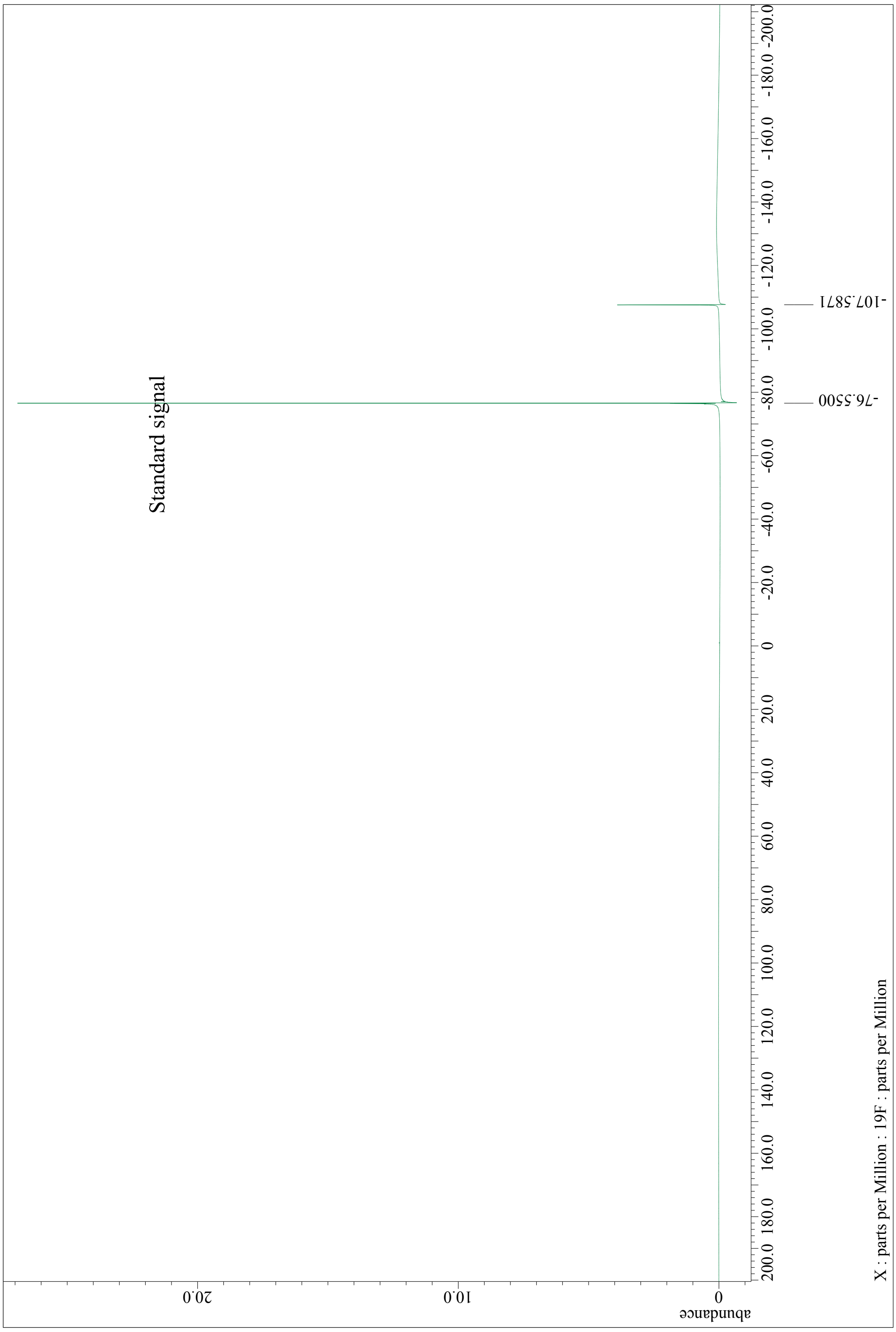

Figure S36. $^{19}F\{^{1}H\}$ NMR spectra of 2,2,3,3-tetrafluoro-1,4-diiodobutane in $CDCl_3$.

Supplementary References

1. Konno, Y., Morooka, R., Morishita, T., Zhao, P., Miyasaka, N., Ono, K., Noda, A., Uchida, D., Iwasaki, R., Yamada, M., Ehara, M. & Maeda, Y. Effect on the tether length of reagents with two reactive sites on the band gap of single-walled carbon nanotubes. *Submitted.*
2. Maeda, Y., Morooka, R., Zhao, P., Uchida, D., Konno, Y., Yamada, M. & Ehara, M. Controlling near-infrared photoluminescence properties of single-walled carbon nanotubes by substituent effect in stepwise chemical functionalization. *J. Phys. Chem. C* **127**, 2360-2370 (2023).
3. Kwon, H., Furmanchuk, A., Kim, M., Meany, B., Guo, Y., Schatz, G. C. & Wang, Y. Molecularly tunable fluorescent quantum defects. *J. Am. Chem. Soc.* **138**, 6878-6885 (2016).
4. Arkhipov, D. E., Lyubeshkin,A. V., Volodin, A. D. & Korlyukov, A. A.Molecular Structures Polymorphism the Role of F…F Interactions in Crystal Packing of Fluorinated Tosylates, *Crystals*, **9**, 242- (2019).
5. Puy, M. V. D., Belter, R. K., Borowski, R. J., Elis, L. A. S., Persichini III, P. J. Poss, A. J., Rygas, T. P. & Tung, H. S. A high-yield synthesis of 3-carboethoxy-4-trifluoromethylfuran and some Diels-Alder reactions of this furoate with acetylenic dienophiles, *J. Fluorine Chem.* **71**, 59-69 (1995).
