# Supplementary material for: Selective emergence of photoluminescence at telecommunication wavelengths from cyclic perfluoroalkylated carbon nanotubes
Source: Commun Chem. 2023 Jul 31;6:159. doi: 10.1038/s42004-023-00950-1 (PMC10390534; doi:10.1038/s42004-023-00950-1)
Supplement: Supplementary file 3 — Description of Additional Supplementary Files [file 42004_2023_950_MOESM3_ESM.pdf]

Description of Additional Supplementary Files

File name: Supplementary Data 1

Description: Text Data of Figure 1 and 2

File name: Supplementary Data 2

Description: Coordinates of the optimized computational models
