# Supplementary material for: Selective emergence of photoluminescence at telecommunication wavelengths from cyclic perfluoroalkylated carbon nanotubes
Source: Commun Chem. 2023 Jul 31;6:159. doi: 10.1038/s42004-023-00950-1 (PMC10390534; doi:10.1038/s42004-023-00950-1)
Supplement: Supplementary file 4 — Supplementary Data 1 [file 42004_2023_950_MOESM4_ESM.pdf]

Figures 1b and 1d

Wavelength	SWNTs	SWNT-$(CH_2)_3CH_3$	SWNT-$(CH_2)_3CF_3$	SWNT-$(CH_2)_2CF_2CF_3$	SWNT-$CH_2(CF_2)_2CF_3$
1300	0.03035	0.05510	0.04002	0.03237	0.02789
1299	0.03049	0.05506	0.04016	0.03248	0.02804
1298	0.03063	0.05520	0.04014	0.03259	0.02811
1297	0.03077	0.05532	0.04019	0.03267	0.02813
1296	0.03081	0.05538	0.04037	0.03265	0.02828
1295	0.03103	0.05552	0.04037	0.03276	0.02844
1294	0.03114	0.05564	0.04046	0.03279	0.02852
1293	0.03131	0.05573	0.04064	0.03281	0.02863
1292	0.03145	0.05586	0.04062	0.03297	0.02870
1291	0.03155	0.05608	0.04075	0.03311	0.02881
1290	0.03170	0.05615	0.04082	0.03312	0.02891
1289	0.03186	0.05632	0.04091	0.03328	0.02906
1288	0.03204	0.05646	0.04101	0.03339	0.02916
1287	0.03221	0.05667	0.04109	0.03351	0.02937
1286	0.03238	0.05693	0.04120	0.03355	0.02945
1285	0.03252	0.05710	0.04130	0.03359	0.02956
1284	0.03271	0.05728	0.04146	0.03374	0.02976
1283	0.03287	0.05751	0.04158	0.03383	0.02993
1282	0.03309	0.05766	0.04169	0.03397	0.03006
1281	0.03317	0.05791	0.04178	0.03409	0.03020
1280	0.03329	0.05802	0.04192	0.03411	0.03034
1279	0.03351	0.05817	0.04214	0.03430	0.03053
1278	0.03362	0.05835	0.04220	0.03446	0.03073
1277	0.03381	0.05847	0.04234	0.03459	0.03096
1276	0.03398	0.05865	0.04248	0.03474	0.03104
1275	0.03418	0.05888	0.04264	0.03486	0.03124
1274	0.03437	0.05906	0.04280	0.03495	0.03144
1273	0.03452	0.05918	0.04299	0.03505	0.03154
1272	0.03464	0.05947	0.04313	0.03521	0.03171

1271	0.03475	0.05970	0.04321	0.03532	0.03193
1270	0.03492	0.06000	0.04338	0.03549	0.03199
1269	0.03504	0.06027	0.04348	0.03564	0.03221
1268	0.03515	0.06050	0.04363	0.03575	0.03234
1267	0.03529	0.06086	0.04380	0.03582	0.03250
1266	0.03535	0.06121	0.04388	0.03598	0.03260
1265	0.03542	0.06161	0.04403	0.03606	0.03289
1264	0.03551	0.06194	0.04414	0.03621	0.03287
1263	0.03559	0.06230	0.04421	0.03635	0.03298
1262	0.03565	0.06266	0.04440	0.03640	0.03306
1261	0.03569	0.06304	0.04450	0.03643	0.03315
1260	0.03574	0.06345	0.04460	0.03654	0.03325
1259	0.03570	0.06388	0.04468	0.03662	0.03344
1258	0.03574	0.06423	0.04479	0.03667	0.03350
1257	0.03578	0.06464	0.04490	0.03684	0.03353
1256	0.03578	0.06501	0.04496	0.03687	0.03359
1255	0.03583	0.06540	0.04516	0.03695	0.03370
1254	0.03579	0.06576	0.04524	0.03700	0.03380
1253	0.03576	0.06624	0.04532	0.03707	0.03386
1252	0.03573	0.06656	0.04537	0.03713	0.03387
1251	0.03571	0.06697	0.04542	0.03714	0.03392
1250	0.03573	0.06739	0.04551	0.03722	0.03397
1249	0.03572	0.06776	0.04558	0.03729	0.03397
1248	0.03565	0.06804	0.04569	0.03732	0.03403
1247	0.03557	0.06838	0.04580	0.03740	0.03408
1246	0.03558	0.06876	0.04587	0.03741	0.03408
1245	0.03561	0.06912	0.04597	0.03737	0.03414
1244	0.03554	0.06937	0.04608	0.03744	0.03423
1243	0.03550	0.06967	0.04612	0.03751	0.03415
1242	0.03549	0.07003	0.04626	0.03752	0.03413
1241	0.03544	0.07028	0.04633	0.03755	0.03418
1240	0.03543	0.07053	0.04649	0.03749	0.03420

1239	0.03543	0.07082	0.04651	0.03761	0.03422
1238	0.03541	0.07110	0.04660	0.03755	0.03415
1237	0.03539	0.07127	0.04665	0.03758	0.03422
1236	0.03536	0.07147	0.04674	0.03753	0.03417
1235	0.03539	0.07165	0.04687	0.03757	0.03423
1234	0.03541	0.07180	0.04693	0.03756	0.03420
1233	0.03537	0.07199	0.04707	0.03760	0.03422
1232	0.03539	0.07212	0.04706	0.03758	0.03427
1231	0.03546	0.07229	0.04709	0.03754	0.03424
1230	0.03550	0.07243	0.04719	0.03746	0.03432
1229	0.03552	0.07250	0.04720	0.03750	0.03438
1228	0.03564	0.07263	0.04724	0.03745	0.03447
1227	0.03569	0.07269	0.04735	0.03738	0.03453
1226	0.03576	0.07276	0.04740	0.03738	0.03458
1225	0.03587	0.07285	0.04747	0.03722	0.03463
1224	0.03597	0.07290	0.04750	0.03724	0.03472
1223	0.03607	0.07294	0.04751	0.03722	0.03482
1222	0.03628	0.07308	0.04759	0.03715	0.03494
1221	0.03646	0.07310	0.04767	0.03704	0.03499
1220	0.03661	0.07313	0.04768	0.03699	0.03519
1219	0.03677	0.07322	0.04776	0.03699	0.03533
1218	0.03694	0.07324	0.04778	0.03691	0.03546
1217	0.03719	0.07339	0.04777	0.03677	0.03562
1216	0.03738	0.07336	0.04782	0.03677	0.03573
1215	0.03759	0.07336	0.04787	0.03671	0.03594
1214	0.03780	0.07344	0.04787	0.03672	0.03618
1213	0.03805	0.07344	0.04798	0.03665	0.03639
1212	0.03829	0.07351	0.04804	0.03670	0.03655
1211	0.03857	0.07357	0.04803	0.03667	0.03678
1210	0.03882	0.07358	0.04811	0.03668	0.03702
1209	0.03921	0.07361	0.04823	0.03669	0.03728
1208	0.03953	0.07361	0.04829	0.03672	0.03743

1207	0.03980	0.07357	0.04845	0.03676	0.03765
1206	0.04010	0.07365	0.04851	0.03671	0.03800
1205	0.04045	0.07379	0.04849	0.03680	0.03823
1204	0.04082	0.07384	0.04867	0.03683	0.03857
1203	0.04113	0.07391	0.04874	0.03676	0.03884
1202	0.04148	0.07391	0.04886	0.03678	0.03914
1201	0.04183	0.07394	0.04896	0.03677	0.03934
1200	0.04182	0.07392	0.04913	0.03675	0.03977
1199	0.04218	0.07391	0.04923	0.03672	0.04010
1198	0.04263	0.07405	0.04933	0.03675	0.04042
1197	0.04308	0.07406	0.04946	0.03683	0.04077
1196	0.04347	0.07424	0.04961	0.03685	0.04104
1195	0.04390	0.07425	0.04974	0.03698	0.04148
1194	0.04439	0.07431	0.04986	0.03708	0.04183
1193	0.04488	0.07445	0.05008	0.03723	0.04219
1192	0.04543	0.07456	0.05025	0.03751	0.04263
1191	0.04593	0.07475	0.05037	0.03776	0.04315
1190	0.04647	0.07492	0.05048	0.03803	0.04350
1189	0.04709	0.07496	0.05069	0.03834	0.04399
1188	0.04770	0.07511	0.05084	0.03868	0.04448
1187	0.04832	0.07531	0.05095	0.03917	0.04503
1186	0.04896	0.07538	0.05111	0.03954	0.04556
1185	0.04958	0.07555	0.05123	0.04002	0.04609
1184	0.05023	0.07562	0.05143	0.04041	0.04664
1183	0.05085	0.07562	0.05157	0.04089	0.04712
1182	0.05164	0.07569	0.05175	0.04133	0.04779
1181	0.05237	0.07561	0.05193	0.04178	0.04839
1180	0.05318	0.07550	0.05209	0.04213	0.04898
1179	0.05391	0.07540	0.05218	0.04254	0.04967
1178	0.05472	0.07517	0.05239	0.04287	0.05027
1177	0.05553	0.07493	0.05250	0.04329	0.05085
1176	0.05637	0.07463	0.05268	0.04371	0.05145

1175	0.05719	0.07427	0.05285	0.04409	0.05212
1174	0.05800	0.07391	0.05301	0.04440	0.05274
1173	0.05891	0.07357	0.05322	0.04474	0.05335
1172	0.05971	0.07320	0.05341	0.04514	0.05395
1171	0.06057	0.07287	0.05355	0.04559	0.05458
1170	0.06151	0.07269	0.05383	0.04591	0.05514
1169	0.06237	0.07250	0.05403	0.04634	0.05582
1168	0.06317	0.07236	0.05415	0.04674	0.05635
1167	0.06404	0.07233	0.05428	0.04717	0.05689
1166	0.06486	0.07234	0.05449	0.04753	0.05744
1165	0.06567	0.07238	0.05476	0.04790	0.05799
1164	0.06638	0.07255	0.05495	0.04834	0.05855
1163	0.06716	0.07269	0.05519	0.04876	0.05905
1162	0.06791	0.07281	0.05540	0.04917	0.05955
1161	0.06858	0.07308	0.05564	0.04953	0.06007
1160	0.06933	0.07334	0.05591	0.04991	0.06054
1159	0.07006	0.07360	0.05604	0.05019	0.06106
1158	0.07080	0.07390	0.05628	0.05065	0.06160
1157	0.07156	0.07422	0.05647	0.05100	0.06216
1156	0.07231	0.07458	0.05670	0.05136	0.06269
1155	0.07311	0.07491	0.05694	0.05176	0.06321
1154	0.07400	0.07531	0.05711	0.05211	0.06375
1153	0.07496	0.07559	0.05732	0.05250	0.06432
1152	0.07597	0.07595	0.05761	0.05282	0.06494
1151	0.07700	0.07628	0.05784	0.05332	0.06553
1150	0.07805	0.07667	0.05806	0.05372	0.06605
1149	0.07916	0.07716	0.05831	0.05409	0.06664
1148	0.08026	0.07748	0.05848	0.05446	0.06721
1147	0.08138	0.07786	0.05871	0.05488	0.06770
1146	0.08237	0.07834	0.05893	0.05532	0.06831
1145	0.08343	0.07871	0.05915	0.05572	0.06884
1144	0.08434	0.07919	0.05938	0.05616	0.06935

1143	0.08515	0.07967	0.05961	0.05653	0.06982
1142	0.08583	0.08006	0.05984	0.05695	0.07020
1141	0.08648	0.08050	0.06005	0.05738	0.07063
1140	0.08700	0.08097	0.06032	0.05776	0.07108
1139	0.08743	0.08138	0.06065	0.05816	0.07145
1138	0.08776	0.08195	0.06094	0.05863	0.07181
1137	0.08808	0.08236	0.06115	0.05908	0.07220
1136	0.08839	0.08280	0.06140	0.05949	0.07269
1135	0.08865	0.08332	0.06171	0.05997	0.07299
1134	0.08893	0.08377	0.06197	0.06045	0.07346
1133	0.08915	0.08428	0.06234	0.06102	0.07393
1132	0.08946	0.08483	0.06267	0.06155	0.07442
1131	0.08981	0.08533	0.06295	0.06205	0.07490
1130	0.09024	0.08581	0.06335	0.06251	0.07543
1129	0.09059	0.08632	0.06359	0.06308	0.07588
1128	0.09100	0.08677	0.06390	0.06370	0.07648
1127	0.09155	0.08731	0.06421	0.06413	0.07696
1126	0.09201	0.08780	0.06453	0.06474	0.07752
1125	0.09253	0.08836	0.06478	0.06528	0.07798
1124	0.09305	0.08878	0.06519	0.06568	0.07842
1123	0.09358	0.08912	0.06541	0.06610	0.07878
1122	0.09416	0.08955	0.06574	0.06655	0.07908
1121	0.09463	0.08992	0.06595	0.06690	0.07938
1120	0.09511	0.09032	0.06611	0.06725	0.07950
1119	0.09542	0.09064	0.06636	0.06757	0.07961
1118	0.09575	0.09095	0.06651	0.06783	0.07959
1117	0.09596	0.09125	0.06662	0.06807	0.07960
1116	0.09600	0.09153	0.06677	0.06825	0.07939
1115	0.09592	0.09174	0.06679	0.06834	0.07909
1114	0.09563	0.09194	0.06688	0.06839	0.07885
1113	0.09512	0.09212	0.06697	0.06847	0.07843
1112	0.09433	0.09225	0.06696	0.06843	0.07788

1111	0.09343	0.09235	0.06700	0.06839	0.07731
1110	0.09237	0.09239	0.06704	0.06831	0.07667
1109	0.09116	0.09242	0.06700	0.06810	0.07596
1108	0.08970	0.09249	0.06694	0.06783	0.07516
1107	0.08819	0.09245	0.06683	0.06758	0.07432
1106	0.08667	0.09237	0.06670	0.06727	0.07349
1105	0.08506	0.09223	0.06673	0.06689	0.07263
1104	0.08342	0.09214	0.06657	0.06655	0.07179
1103	0.08178	0.09191	0.06642	0.06616	0.07082
1102	0.08027	0.09171	0.06628	0.06571	0.06994
1101	0.07872	0.09153	0.06606	0.06525	0.06907
1100	0.07726	0.09129	0.06589	0.06472	0.06821
1099	0.07593	0.09110	0.06578	0.06433	0.06740
1098	0.07473	0.09079	0.06559	0.06385	0.06663
1097	0.07361	0.09050	0.06537	0.06331	0.06591
1096	0.07257	0.09025	0.06526	0.06283	0.06523
1095	0.07163	0.08987	0.06500	0.06234	0.06460
1094	0.07081	0.08958	0.06486	0.06177	0.06407
1093	0.07007	0.08930	0.06472	0.06122	0.06355
1092	0.06940	0.08895	0.06439	0.06071	0.06305
1091	0.06895	0.08862	0.06418	0.06026	0.06271
1090	0.06853	0.08823	0.06397	0.05971	0.06233
1089	0.06821	0.08790	0.06371	0.05928	0.06203
1088	0.06802	0.08760	0.06354	0.05869	0.06178
1087	0.06790	0.08719	0.06322	0.05830	0.06152
1086	0.06784	0.08689	0.06300	0.05783	0.06141
1085	0.06793	0.08653	0.06286	0.05729	0.06137
1084	0.06804	0.08617	0.06260	0.05690	0.06124
1083	0.06821	0.08581	0.06232	0.05646	0.06128
1082	0.06843	0.08547	0.06213	0.05600	0.06136
1081	0.06879	0.08520	0.06189	0.05574	0.06143
1080	0.06917	0.08493	0.06171	0.05531	0.06157

1079	0.06962	0.08459	0.06157	0.05502	0.06163
1078	0.07005	0.08419	0.06139	0.05470	0.06177
1077	0.07049	0.08389	0.06125	0.05446	0.06204
1076	0.07107	0.08361	0.06110	0.05418	0.06227
1075	0.07172	0.08339	0.06093	0.05400	0.06259
1074	0.07236	0.08311	0.06087	0.05384	0.06289
1073	0.07305	0.08283	0.06077	0.05363	0.06328
1072	0.07388	0.08261	0.06065	0.05354	0.06369
1071	0.07468	0.08239	0.06065	0.05335	0.06412
1070	0.07554	0.08217	0.06056	0.05335	0.06463
1069	0.07656	0.08202	0.06050	0.05331	0.06517
1068	0.07755	0.08185	0.06050	0.05322	0.06579
1067	0.07862	0.08169	0.06050	0.05322	0.06646
1066	0.07980	0.08159	0.06051	0.05327	0.06712
1065	0.08104	0.08149	0.06056	0.05326	0.06793
1064	0.08228	0.08144	0.06048	0.05336	0.06869
1063	0.08361	0.08129	0.06054	0.05343	0.06943
1062	0.08492	0.08123	0.06049	0.05349	0.07018
1061	0.08620	0.08125	0.06046	0.05359	0.07099
1060	0.08766	0.08123	0.06050	0.05364	0.07182
1059	0.08898	0.08126	0.06059	0.05377	0.07256
1058	0.09032	0.08125	0.06060	0.05382	0.07341
1057	0.09164	0.08136	0.06067	0.05393	0.07423
1056	0.09301	0.08141	0.06069	0.05407	0.07497
1055	0.09436	0.08155	0.06075	0.05431	0.07580
1054	0.09577	0.08174	0.06093	0.05445	0.07672
1053	0.09717	0.08193	0.06102	0.05464	0.07758
1052	0.09858	0.08212	0.06115	0.05486	0.07854
1051	0.10012	0.08231	0.06123	0.05511	0.07941
1050	0.10171	0.08253	0.06140	0.05526	0.08045
1049	0.10325	0.08281	0.06154	0.05550	0.08154
1048	0.10487	0.08307	0.06168	0.05577	0.08259

1047	0.10666	0.08335	0.06187	0.05610	0.08367
1046	0.10841	0.08370	0.06212	0.05647	0.08481
1045	0.11020	0.08407	0.06240	0.05682	0.08610
1044	0.11207	0.08451	0.06260	0.05718	0.08737
1043	0.11407	0.08484	0.06284	0.05759	0.08865
1042	0.11614	0.08527	0.06307	0.05800	0.09005
1041	0.11828	0.08585	0.06337	0.05837	0.09158
1040	0.12056	0.08633	0.06372	0.05889	0.09309
1039	0.12302	0.08684	0.06394	0.05936	0.09485
1038	0.12576	0.08741	0.06430	0.05995	0.09671
1037	0.12869	0.08806	0.06468	0.06059	0.09869
1036	0.13181	0.08877	0.06499	0.06133	0.10088
1035	0.13533	0.08944	0.06544	0.06197	0.10327
1034	0.13908	0.09021	0.06583	0.06271	0.10566
1033	0.14304	0.09097	0.06624	0.06352	0.10821
1032	0.14710	0.09177	0.06670	0.06433	0.11087
1031	0.15136	0.09261	0.06710	0.06518	0.11364
1030	0.15563	0.09348	0.06758	0.06606	0.11630
1029	0.15993	0.09419	0.06809	0.06681	0.11896
1028	0.16418	0.09509	0.06843	0.06772	0.12152
1027	0.16815	0.09593	0.06882	0.06854	0.12400
1026	0.17189	0.09668	0.06920	0.06932	0.12633
1025	0.17519	0.09751	0.06962	0.07003	0.12847
1024	0.17823	0.09821	0.06995	0.07072	0.13038
1023	0.18096	0.09898	0.07030	0.07144	0.13225
1022	0.18332	0.09969	0.07060	0.07213	0.13389
1021	0.18542	0.10035	0.07099	0.07269	0.13533
1020	0.18718	0.10103	0.07119	0.07321	0.13676
1019	0.18878	0.10164	0.07147	0.07375	0.13815
1018	0.19039	0.10229	0.07169	0.07434	0.13956
1017	0.19199	0.10290	0.07193	0.07476	0.14106
1016	0.19377	0.10339	0.07225	0.07512	0.14259

1015	0.19571	0.10395	0.07251	0.07563	0.14421
1014	0.19799	0.10460	0.07278	0.07611	0.14600
1013	0.20044	0.10522	0.07297	0.07664	0.14802
1012	0.20343	0.10580	0.07323	0.07720	0.15022
1011	0.20670	0.10644	0.07353	0.07779	0.15276
1010	0.21048	0.10714	0.07387	0.07840	0.15533
1009	0.21462	0.10789	0.07416	0.07904	0.15832
1008	0.21927	0.10855	0.07442	0.07984	0.16141
1007	0.22426	0.10938	0.07478	0.08062	0.16487
1006	0.22960	0.11022	0.07517	0.08141	0.16851
1005	0.23527	0.11105	0.07555	0.08225	0.17233
1004	0.24115	0.11189	0.07600	0.08328	0.17637
1003	0.24708	0.11272	0.07639	0.08422	0.18066
1002	0.25319	0.11378	0.07688	0.08527	0.18488
1001	0.25927	0.11478	0.07738	0.08647	0.18930
1000	0.26519	0.11577	0.07787	0.08764	0.19391
999	0.27086	0.11681	0.07837	0.08880	0.19862
998	0.27641	0.11783	0.07887	0.09017	0.20323
997	0.28175	0.11896	0.07944	0.09159	0.20801
996	0.28684	0.12001	0.08007	0.09303	0.21282
995	0.29172	0.12118	0.08073	0.09455	0.21777
994	0.29650	0.12244	0.08136	0.09622	0.22282
993	0.30115	0.12371	0.08213	0.09788	0.22807
992	0.30587	0.12504	0.08287	0.09971	0.23322
991	0.31064	0.12643	0.08368	0.10162	0.23867
990	0.31556	0.12791	0.08455	0.10374	0.24429
989	0.32069	0.12933	0.08541	0.10582	0.25003
988	0.32601	0.13082	0.08628	0.10802	0.25586
987	0.33156	0.13237	0.08728	0.11032	0.26168
986	0.33741	0.13400	0.08815	0.11257	0.26739
985	0.34334	0.13551	0.08913	0.11501	0.27293
984	0.34910	0.13714	0.09000	0.11742	0.27801

983	0.35466	0.13870	0.09087	0.11979	0.28262
982	0.35955	0.14017	0.09170	0.12207	0.28659
981	0.36367	0.14157	0.09257	0.12423	0.28956
980	0.36637	0.14286	0.09318	0.12615	0.29168
979	0.36751	0.14398	0.09368	0.12790	0.29247
978	0.36680	0.14494	0.09423	0.12936	0.29217
977	0.36405	0.14562	0.09460	0.13042	0.29069
976	0.35925	0.14615	0.09485	0.13123	0.28789
975	0.35253	0.14656	0.09494	0.13173	0.28396
974	0.34402	0.14655	0.09481	0.13183	0.27896
973	0.33430	0.14646	0.09475	0.13168	0.27310
972	0.32345	0.14621	0.09457	0.13115	0.26657
971	0.31180	0.14566	0.09413	0.13034	0.25953
970	0.29997	0.14502	0.09363	0.12923	0.25217
969	0.28799	0.14421	0.09317	0.12793	0.24458
968	0.27659	0.14315	0.09256	0.12656	0.23714
967	0.26590	0.14199	0.09190	0.12499	0.22978
966	0.25613	0.14074	0.09125	0.12334	0.22295
965	0.24735	0.13949	0.09060	0.12159	0.21657
964	0.23966	0.13809	0.08995	0.11982	0.21074
963	0.23310	0.13651	0.08925	0.11812	0.20538
962	0.22754	0.13498	0.08855	0.11638	0.20064
961	0.22277	0.13345	0.08794	0.11477	0.19637
960	0.21865	0.13193	0.08729	0.11323	0.19256
959	0.21491	0.13037	0.08664	0.11159	0.18894
958	0.21136	0.12874	0.08609	0.11010	0.18566
957	0.20770	0.12713	0.08549	0.10855	0.18232
956	0.20371	0.12564	0.08481	0.10714	0.17909
955	0.19945	0.12400	0.08410	0.10571	0.17567
954	0.19476	0.12244	0.08353	0.10425	0.17210
953	0.18949	0.12085	0.08304	0.10280	0.16834
952	0.18388	0.11934	0.08249	0.10133	0.16442

951	0.17801	0.11784	0.08188	0.10005	0.16027
950	0.17196	0.11635	0.08126	0.09859	0.15604
949	0.16594	0.11492	0.08069	0.09712	0.15180
948	0.16001	0.11363	0.08021	0.09577	0.14753
947	0.15433	0.11229	0.07966	0.09446	0.14340
946	0.14905	0.11105	0.07920	0.09313	0.13928
945	0.14412	0.10986	0.07872	0.09187	0.13537
944	0.13957	0.10872	0.07820	0.09070	0.13179
943	0.13568	0.10769	0.07778	0.08952	0.12839
942	0.13214	0.10657	0.07747	0.08837	0.12531
941	0.12901	0.10563	0.07695	0.08735	0.12234
940	0.12629	0.10461	0.07648	0.08621	0.11964
939	0.12385	0.10366	0.07607	0.08518	0.11719
938	0.12163	0.10279	0.07568	0.08426	0.11496
937	0.11982	0.10190	0.07531	0.08338	0.11288
936	0.11816	0.10105	0.07490	0.08246	0.11108
935	0.11669	0.10020	0.07452	0.08153	0.10934
934	0.11552	0.09947	0.07422	0.08069	0.10785
933	0.11446	0.09866	0.07385	0.07991	0.10661
932	0.11362	0.09796	0.07354	0.07924	0.10529
931	0.11297	0.09723	0.07316	0.07848	0.10423
930	0.11236	0.09655	0.07292	0.07787	0.10329
929	0.11193	0.09590	0.07268	0.07725	0.10256
928	0.11186	0.09537	0.07252	0.07662	0.10191
927	0.11195	0.09473	0.07223	0.07616	0.10148
926	0.11226	0.09427	0.07198	0.07565	0.10114
925	0.11289	0.09383	0.07175	0.07522	0.10104
924	0.11367	0.09331	0.07160	0.07473	0.10093
923	0.11472	0.09284	0.07143	0.07450	0.10113
922	0.11587	0.09236	0.07136	0.07413	0.10140
921	0.11731	0.09200	0.07117	0.07385	0.10178
920	0.11878	0.09169	0.07108	0.07352	0.10220

919	0.12017	0.09122	0.07099	0.07328	0.10278
918	0.12146	0.09094	0.07083	0.07300	0.10325
917	0.12256	0.09062	0.07068	0.07271	0.10377
916	0.12348	0.09025	0.07053	0.07245	0.10414
915	0.12405	0.08998	0.07041	0.07216	0.10451
914	0.12415	0.08977	0.07036	0.07189	0.10469
913	0.12395	0.08940	0.07017	0.07171	0.10468
912	0.12355	0.08926	0.07003	0.07140	0.10469
911	0.12268	0.08892	0.06991	0.07123	0.10445
910	0.12176	0.08857	0.06983	0.07117	0.10419
909	0.12074	0.08840	0.06975	0.07080	0.10375
908	0.11978	0.08816	0.06970	0.07065	0.10325
907	0.11877	0.08801	0.06966	0.07033	0.10277
906	0.11785	0.08776	0.06953	0.07017	0.10233
905	0.11709	0.08752	0.06947	0.07001	0.10183
904	0.11643	0.08736	0.06946	0.06989	0.10156
903	0.11600	0.08715	0.06954	0.06978	0.10120
902	0.11587	0.08704	0.06941	0.06966	0.10091
901	0.11580	0.08694	0.06939	0.06944	0.10086
900	0.11595	0.08669	0.06942	0.06937	0.10081
899	0.11634	0.08663	0.06944	0.06929	0.10093
898	0.11684	0.08656	0.06936	0.06914	0.10107
897	0.11743	0.08647	0.06937	0.06911	0.10134
896	0.11824	0.08641	0.06939	0.06905	0.10184
895	0.11912	0.08635	0.06938	0.06903	0.10249
894	0.12007	0.08635	0.06935	0.06892	0.10306
893	0.12133	0.08633	0.06938	0.06883	0.10380
892	0.12263	0.08625	0.06937	0.06891	0.10480
891	0.12417	0.08614	0.06943	0.06888	0.10576
890	0.12575	0.08615	0.06949	0.06879	0.10676
889	0.12754	0.08627	0.06958	0.06891	0.10801
888	0.12965	0.08639	0.06965	0.06883	0.10951

887	0.13204	0.08631	0.06964	0.06901	0.11087
886	0.13455	0.08630	0.06971	0.06917	0.11240
885	0.13734	0.08638	0.06988	0.06934	0.11401
884	0.14062	0.08636	0.06991	0.06940	0.11578
883	0.14395	0.08643	0.07010	0.06958	0.11759
882	0.14736	0.08657	0.07017	0.06978	0.11930
881	0.15093	0.08649	0.07030	0.06990	0.12121
880	0.15440	0.08677	0.07047	0.06999	0.12293
879	0.15754	0.08680	0.07062	0.07024	0.12439
878	0.16059	0.08684	0.07069	0.07055	0.12589
877	0.16305	0.08701	0.07073	0.07061	0.12717
876	0.16454	0.08728	0.07083	0.07057	0.12829
875	0.16546	0.08730	0.07070	0.07075	0.12920
874	0.16549	0.08731	0.07087	0.07101	0.12969
873	0.16460	0.08740	0.07090	0.07121	0.12986
872	0.16316	0.08743	0.07098	0.07119	0.12976
871	0.16068	0.08738	0.07105	0.07128	0.12926
870	0.15778	0.08736	0.07093	0.07137	0.12861
869	0.15428	0.08740	0.07104	0.07129	0.12760
868	0.15048	0.08741	0.07108	0.07136	0.12638
867	0.14651	0.08741	0.07109	0.07157	0.12487
866	0.14242	0.08740	0.07117	0.07163	0.12336
865	0.13874	0.08746	0.07127	0.07159	0.12174
864	0.13514	0.08766	0.07129	0.07169	0.12002
863	0.13168	0.08760	0.07133	0.07187	0.11830
862	0.12872	0.08753	0.07143	0.07187	0.11658
861	0.12597	0.08768	0.07148	0.07177	0.11501
860	0.12373	0.08764	0.07163	0.07185	0.11340
859	0.12153	0.08772	0.07147	0.07196	0.11191
858	0.11989	0.08788	0.07157	0.07203	0.11061
857	0.11827	0.08778	0.07154	0.07198	0.10940
856	0.11671	0.08774	0.07151	0.07213	0.10817

855	0.11569	0.08772	0.07154	0.07209	0.10699
854	0.11458	0.08776	0.07167	0.07211	0.10586
853	0.11372	0.08772	0.07161	0.07212	0.10496
852	0.11275	0.08763	0.07165	0.07210	0.10414
851	0.11220	0.08765	0.07159	0.07207	0.10318
850	0.11132	0.08628	0.07102	0.07262	0.10399
849	0.11083	0.08624	0.07116	0.07224	0.10335
848	0.11027	0.08627	0.07113	0.07223	0.10277
847	0.10973	0.08616	0.07129	0.07238	0.10200
846	0.10944	0.08600	0.07136	0.07249	0.10127
845	0.10904	0.08612	0.07154	0.07246	0.10060
844	0.10867	0.08595	0.07164	0.07244	0.09997
843	0.10837	0.08574	0.07160	0.07255	0.09927
842	0.10778	0.08570	0.07141	0.07257	0.09884
841	0.10748	0.08571	0.07159	0.07253	0.09824
840	0.10732	0.08568	0.07167	0.07273	0.09770
839	0.10708	0.08553	0.07147	0.07250	0.09711
838	0.10698	0.08532	0.07147	0.07238	0.09655
837	0.10685	0.08526	0.07150	0.07225	0.09614
836	0.10661	0.08526	0.07132	0.07223	0.09569
835	0.10665	0.08509	0.07120	0.07221	0.09529
834	0.10674	0.08489	0.07126	0.07197	0.09488
833	0.10671	0.08459	0.07125	0.07188	0.09450
832	0.10685	0.08446	0.07115	0.07162	0.09407
831	0.10700	0.08437	0.07108	0.07149	0.09385
830	0.10696	0.08422	0.07107	0.07143	0.09350
829	0.10670	0.08411	0.07114	0.07130	0.09317
828	0.10657	0.08392	0.07113	0.07130	0.09291
827	0.10609	0.08368	0.07111	0.07130	0.09247
826	0.10524	0.08369	0.07105	0.07116	0.09215
825	0.10451	0.08353	0.07096	0.07099	0.09168
824	0.10342	0.08332	0.07106	0.07102	0.09120

823	0.10224	0.08313	0.07105	0.07086	0.09073
822	0.10108	0.08293	0.07092	0.07072	0.09016
821	0.09986	0.08286	0.07086	0.07055	0.08968
820	0.09852	0.08251	0.07075	0.07050	0.08905
819	0.09724	0.08236	0.07061	0.07018	0.08839
818	0.09607	0.08217	0.07062	0.06991	0.08776
817	0.09479	0.08200	0.07051	0.06973	0.08711
816	0.09368	0.08178	0.07054	0.06951	0.08629
815	0.09260	0.08159	0.07046	0.06929	0.08561
814	0.09158	0.08151	0.07026	0.06913	0.08493
813	0.09058	0.08119	0.07029	0.06889	0.08417
812	0.08972	0.08096	0.07029	0.06870	0.08349
811	0.08882	0.08072	0.07032	0.06856	0.08283
810	0.08791	0.08055	0.07026	0.06828	0.08223
809	0.08715	0.08039	0.07030	0.06821	0.08156
808	0.08652	0.08009	0.07032	0.06807	0.08094
807	0.08587	0.07991	0.07022	0.06796	0.08025
806	0.08518	0.07975	0.07017	0.06773	0.07968
805	0.08470	0.07961	0.07015	0.06758	0.07917
804	0.08408	0.07931	0.07010	0.06735	0.07865
803	0.08345	0.07920	0.07001	0.06717	0.07828
802	0.08302	0.07885	0.06995	0.06684	0.07789
801	0.08249	0.07868	0.06982	0.06662	0.07737
800	0.08216	0.07857	0.06972	0.06648	0.07684
799	0.08166	0.07844	0.06959	0.06616	0.07647
798	0.08122	0.07828	0.06954	0.06590	0.07602
797	0.08084	0.07800	0.06943	0.06573	0.07578
796	0.08039	0.07784	0.06938	0.06553	0.07541
795	0.08005	0.07767	0.06937	0.06547	0.07498
794	0.07970	0.07744	0.06929	0.06541	0.07463
793	0.07942	0.07732	0.06925	0.06520	0.07439
792	0.07900	0.07721	0.06936	0.06510	0.07403

791	0.07867	0.07698	0.06927	0.06496	0.07369
790	0.07832	0.07674	0.06921	0.06490	0.07339
789	0.07804	0.07673	0.06916	0.06472	0.07319
788	0.07782	0.07654	0.06910	0.06458	0.07292
787	0.07762	0.07641	0.06902	0.06433	0.07268
786	0.07752	0.07627	0.06879	0.06419	0.07252
785	0.07734	0.07603	0.06868	0.06403	0.07237
784	0.07731	0.07591	0.06865	0.06379	0.07220
783	0.07720	0.07576	0.06869	0.06366	0.07210
782	0.07700	0.07577	0.06876	0.06357	0.07196
781	0.07698	0.07554	0.06874	0.06350	0.07189
780	0.07703	0.07549	0.06879	0.06338	0.07201
779	0.07710	0.07532	0.06882	0.06343	0.07186
778	0.07707	0.07527	0.06887	0.06348	0.07189
777	0.07717	0.07523	0.06899	0.06342	0.07187
776	0.07736	0.07510	0.06911	0.06345	0.07194
775	0.07738	0.07505	0.06898	0.06351	0.07214
774	0.07759	0.07505	0.06897	0.06336	0.07232
773	0.07773	0.07505	0.06904	0.06328	0.07236
772	0.07803	0.07506	0.06909	0.06324	0.07256
771	0.07815	0.07510	0.06904	0.06315	0.07270
770	0.07823	0.07500	0.06905	0.06311	0.07269
769	0.07843	0.07499	0.06897	0.06301	0.07287
768	0.07868	0.07498	0.06896	0.06294	0.07293
767	0.07878	0.07492	0.06900	0.06296	0.07312
766	0.07881	0.07497	0.06903	0.06299	0.07325
765	0.07898	0.07493	0.06914	0.06309	0.07341
764	0.07904	0.07504	0.06912	0.06305	0.07338
763	0.07908	0.07496	0.06919	0.06315	0.07351
762	0.07911	0.07489	0.06929	0.06327	0.07356
761	0.07922	0.07503	0.06925	0.06316	0.07347
760	0.07927	0.07510	0.06934	0.06324	0.07358

759	0.07930	0.07509	0.06936	0.06327	0.07368
758	0.07934	0.07505	0.06938	0.06321	0.07366
757	0.07947	0.07501	0.06936	0.06312	0.07360
756	0.07949	0.07511	0.06931	0.06318	0.07374
755	0.07955	0.07514	0.06929	0.06314	0.07361
754	0.07957	0.07518	0.06927	0.06309	0.07360
753	0.07966	0.07515	0.06932	0.06316	0.07360
752	0.07977	0.07519	0.06939	0.06321	0.07352
751	0.07991	0.07524	0.06952	0.06325	0.07359
750	0.07998	0.07529	0.06966	0.06337	0.07360
749	0.08016	0.07538	0.06968	0.06342	0.07360
748	0.08028	0.07540	0.06993	0.06348	0.07358
747	0.08045	0.07546	0.07002	0.06361	0.07356
746	0.08059	0.07557	0.07000	0.06361	0.07371
745	0.08084	0.07552	0.07001	0.06357	0.07375
744	0.08104	0.07556	0.07007	0.06361	0.07383
743	0.08112	0.07564	0.07017	0.06371	0.07393
742	0.08130	0.07571	0.07021	0.06368	0.07403
741	0.08148	0.07575	0.07027	0.06368	0.07413
740	0.08145	0.07586	0.07040	0.06373	0.07412
739	0.08160	0.07589	0.07042	0.06378	0.07424
738	0.08179	0.07602	0.07055	0.06397	0.07439
737	0.08194	0.07604	0.07070	0.06403	0.07447
736	0.08208	0.07612	0.07079	0.06411	0.07456
735	0.08221	0.07614	0.07086	0.06428	0.07461
734	0.08227	0.07611	0.07103	0.06435	0.07464
733	0.08236	0.07605	0.07113	0.06437	0.07473
732	0.08235	0.07617	0.07107	0.06444	0.07472
731	0.08226	0.07616	0.07106	0.06440	0.07467
730	0.08231	0.07609	0.07118	0.06440	0.07467
729	0.08234	0.07613	0.07114	0.06433	0.07473
728	0.08234	0.07611	0.07111	0.06436	0.07474

727	0.08231	0.07605	0.07115	0.06438	0.07469
726	0.08233	0.07605	0.07118	0.06441	0.07469
725	0.08230	0.07604	0.07127	0.06445	0.07467
724	0.08230	0.07610	0.07143	0.06451	0.07466
723	0.08234	0.07608	0.07151	0.06449	0.07463
722	0.08242	0.07607	0.07157	0.06458	0.07462
721	0.08240	0.07595	0.07168	0.06461	0.07454
720	0.08243	0.07589	0.07175	0.06463	0.07449
719	0.08243	0.07584	0.07169	0.06454	0.07444
718	0.08239	0.07575	0.07168	0.06453	0.07441
717	0.08236	0.07568	0.07175	0.06447	0.07446
716	0.08248	0.07562	0.07164	0.06446	0.07444
715	0.08252	0.07561	0.07163	0.06429	0.07445
714	0.08273	0.07555	0.07177	0.06414	0.07446
713	0.08286	0.07544	0.07174	0.06412	0.07455
712	0.08308	0.07533	0.07176	0.06410	0.07471
711	0.08327	0.07525	0.07175	0.06410	0.07482
710	0.08350	0.07520	0.07183	0.06417	0.07497
709	0.08383	0.07519	0.07195	0.06418	0.07511
708	0.08419	0.07513	0.07194	0.06416	0.07543
707	0.08454	0.07507	0.07193	0.06423	0.07568
706	0.08490	0.07514	0.07191	0.06429	0.07595
705	0.08549	0.07513	0.07186	0.06428	0.07631
704	0.08603	0.07512	0.07181	0.06433	0.07669
703	0.08652	0.07515	0.07175	0.06428	0.07709
702	0.08703	0.07521	0.07185	0.06428	0.07754
701	0.08768	0.07519	0.07185	0.06432	0.07798
700	0.08824	0.07524	0.07188	0.06442	0.07836
699	0.08881	0.07534	0.07200	0.06461	0.07886
698	0.08941	0.07547	0.07204	0.06470	0.07927
697	0.09000	0.07553	0.07218	0.06490	0.07970
696	0.09056	0.07562	0.07236	0.06506	0.08014

695	0.09109	0.07576	0.07240	0.06521	0.08048
694	0.09157	0.07592	0.07254	0.06534	0.08089
693	0.09196	0.07602	0.07261	0.06546	0.08126
692	0.09229	0.07617	0.07267	0.06553	0.08159
691	0.09257	0.07638	0.07276	0.06568	0.08198
690	0.09278	0.07654	0.07288	0.06578	0.08230
689	0.09306	0.07671	0.07301	0.06592	0.08255
688	0.09329	0.07693	0.07321	0.06608	0.08292
687	0.09357	0.07713	0.07343	0.06626	0.08327
686	0.09387	0.07730	0.07362	0.06650	0.08371
685	0.09432	0.07755	0.07390	0.06678	0.08419
684	0.09489	0.07784	0.07404	0.06699	0.08465
683	0.09554	0.07810	0.07426	0.06718	0.08527
682	0.09639	0.07835	0.07439	0.06740	0.08587
681	0.09724	0.07870	0.07435	0.06762	0.08655
680	0.09819	0.07891	0.07449	0.06786	0.08736
679	0.09928	0.07921	0.07463	0.06798	0.08816
678	0.10049	0.07951	0.07475	0.06814	0.08892
677	0.10182	0.07989	0.07502	0.06843	0.08983
676	0.10322	0.08024	0.07521	0.06898	0.09086
675	0.10461	0.08054	0.07550	0.06941	0.09188
674	0.10607	0.08092	0.07573	0.06984	0.09293
673	0.10757	0.08127	0.07599	0.07021	0.09383
672	0.10908	0.08160	0.07619	0.07062	0.09484
671	0.11052	0.08197	0.07644	0.07100	0.09581
670	0.11195	0.08238	0.07656	0.07137	0.09671
669	0.11325	0.08275	0.07675	0.07170	0.09749
668	0.11448	0.08310	0.07701	0.07201	0.09819
667	0.11554	0.08343	0.07728	0.07233	0.09878
666	0.11639	0.08385	0.07749	0.07266	0.09916
665	0.11701	0.08417	0.07776	0.07305	0.09943
664	0.11732	0.08457	0.07801	0.07340	0.09960

663	0.11736	0.08486	0.07828	0.07377	0.09957
662	0.11716	0.08529	0.07858	0.07421	0.09947
661	0.11689	0.08561	0.07883	0.07452	0.09933
660	0.11647	0.08596	0.07904	0.07472	0.09917
659	0.11604	0.08621	0.07921	0.07501	0.09897
658	0.11574	0.08646	0.07927	0.07517	0.09878
657	0.11546	0.08684	0.07946	0.07536	0.09871
656	0.11532	0.08710	0.07961	0.07556	0.09865
655	0.11533	0.08744	0.07987	0.07580	0.09876
654	0.11544	0.08774	0.08014	0.07614	0.09884
653	0.11561	0.08797	0.08042	0.07647	0.09898
652	0.11599	0.08824	0.08072	0.07683	0.09902
651	0.11630	0.08846	0.08086	0.07717	0.09922
650	0.11649	0.08876	0.08107	0.07738	0.09940
649	0.11678	0.08895	0.08116	0.07759	0.09942
648	0.11681	0.08911	0.08123	0.07770	0.09932
647	0.11667	0.08921	0.08125	0.07779	0.09922
646	0.11642	0.08929	0.08128	0.07782	0.09908
645	0.11598	0.08934	0.08137	0.07780	0.09865
644	0.11537	0.08929	0.08148	0.07782	0.09822
643	0.11462	0.08923	0.08158	0.07788	0.09773
642	0.11364	0.08909	0.08164	0.07787	0.09712
641	0.11261	0.08898	0.08167	0.07769	0.09653
640	0.11158	0.08876	0.08154	0.07754	0.09584
639	0.11043	0.08853	0.08145	0.07728	0.09513
638	0.10930	0.08840	0.08131	0.07703	0.09435
637	0.10810	0.08816	0.08119	0.07664	0.09368
636	0.10697	0.08798	0.08101	0.07630	0.09297
635	0.10592	0.08772	0.08088	0.07594	0.09233
634	0.10489	0.08744	0.08078	0.07568	0.09168
633	0.10395	0.08719	0.08071	0.07548	0.09116
632	0.10308	0.08696	0.08065	0.07531	0.09061

631	0.10231	0.08683	0.08063	0.07507	0.09020
630	0.10180	0.08668	0.08049	0.07491	0.08985
629	0.10124	0.08656	0.08029	0.07478	0.08955
628	0.10085	0.08652	0.08022	0.07457	0.08936
627	0.10069	0.08649	0.08017	0.07433	0.08929
626	0.10054	0.08640	0.08006	0.07420	0.08922
625	0.10058	0.08640	0.08010	0.07419	0.08925
624	0.10069	0.08647	0.08015	0.07413	0.08937
623	0.10086	0.08652	0.08026	0.07418	0.08957
622	0.10107	0.08658	0.08033	0.07424	0.08973
621	0.10148	0.08675	0.08044	0.07433	0.09009
620	0.10199	0.08689	0.08048	0.07441	0.09046
619	0.10258	0.08707	0.08053	0.07451	0.09084
618	0.10323	0.08721	0.08056	0.07456	0.09131
617	0.10388	0.08745	0.08066	0.07470	0.09192
616	0.10470	0.08774	0.08077	0.07487	0.09254
615	0.10563	0.08806	0.08092	0.07509	0.09321
614	0.10653	0.08835	0.08122	0.07529	0.09400
613	0.10758	0.08860	0.08149	0.07559	0.09484
612	0.10876	0.08892	0.08169	0.07594	0.09573
611	0.11000	0.08932	0.08185	0.07619	0.09664
610	0.11134	0.08969	0.08193	0.07646	0.09763
609	0.11272	0.08999	0.08204	0.07670	0.09866
608	0.11423	0.09042	0.08221	0.07695	0.09974
607	0.11573	0.09089	0.08248	0.07724	0.10082
606	0.11749	0.09135	0.08275	0.07766	0.10203
605	0.11920	0.09180	0.08305	0.07804	0.10328
604	0.12102	0.09225	0.08332	0.07851	0.10454
603	0.12303	0.09286	0.08367	0.07907	0.10595
602	0.12517	0.09338	0.08400	0.07955	0.10738
601	0.12750	0.09397	0.08424	0.08004	0.10884
600	0.12982	0.09458	0.08457	0.08046	0.11062

599	0.13240	0.09522	0.08477	0.08095	0.11239
598	0.13507	0.09585	0.08515	0.08147	0.11430
597	0.13787	0.09657	0.08564	0.08209	0.11629
596	0.14078	0.09728	0.08614	0.08286	0.11841
595	0.14383	0.09799	0.08669	0.08366	0.12060
594	0.14708	0.09879	0.08717	0.08454	0.12291
593	0.15028	0.09956	0.08757	0.08529	0.12522
592	0.15350	0.10041	0.08797	0.08597	0.12762
591	0.15672	0.10114	0.08834	0.08668	0.12998
590	0.15992	0.10189	0.08868	0.08736	0.13241
589	0.16293	0.10265	0.08907	0.08812	0.13477
588	0.16579	0.10337	0.08953	0.08885	0.13701
587	0.16850	0.10407	0.08999	0.08962	0.13917
586	0.17108	0.10475	0.09042	0.09045	0.14131
585	0.17337	0.10547	0.09084	0.09120	0.14331
584	0.17543	0.10624	0.09115	0.09192	0.14521
583	0.17716	0.10702	0.09145	0.09262	0.14696
582	0.17861	0.10776	0.09178	0.09330	0.14848
581	0.17980	0.10854	0.09211	0.09407	0.14995
580	0.18085	0.10924	0.09257	0.09487	0.15124
579	0.18173	0.11005	0.09302	0.09570	0.15245
578	0.18260	0.11087	0.09359	0.09665	0.15378
577	0.18359	0.11181	0.09409	0.09771	0.15507
576	0.18466	0.11270	0.09454	0.09872	0.15651
575	0.18590	0.11363	0.09503	0.09970	0.15799
574	0.18734	0.11465	0.09547	0.10072	0.15960
573	0.18879	0.11563	0.09589	0.10173	0.16113
572	0.19027	0.11661	0.09647	0.10288	0.16263
571	0.19154	0.11742	0.09704	0.10406	0.16392
570	0.19255	0.11828	0.09754	0.10505	0.16487
569	0.19303	0.11888	0.09786	0.10594	0.16530
568	0.19276	0.11935	0.09819	0.10659	0.16516

567	0.19169	0.11957	0.09829	0.10694	0.16431
566	0.18969	0.11958	0.09821	0.10694	0.16274
565	0.18669	0.11935	0.09804	0.10669	0.16042
564	0.18300	0.11892	0.09790	0.10616	0.15750
563	0.17867	0.11823	0.09766	0.10542	0.15410
562	0.17382	0.11738	0.09731	0.10449	0.15038
561	0.16885	0.11635	0.09698	0.10344	0.14648
560	0.16385	0.11527	0.09647	0.10220	0.14266
559	0.15910	0.11409	0.09578	0.10080	0.13895
558	0.15466	0.11290	0.09514	0.09935	0.13546
557	0.15051	0.11174	0.09460	0.09798	0.13217
556	0.14676	0.11064	0.09414	0.09676	0.12921
555	0.14351	0.10951	0.09366	0.09560	0.12656
554	0.14059	0.10842	0.09322	0.09450	0.12415
553	0.13790	0.10738	0.09277	0.09343	0.12195
552	0.13552	0.10643	0.09221	0.09239	0.11996
551	0.13325	0.10553	0.09173	0.09142	0.11802
550	0.13117	0.10465	0.09127	0.09042	0.11629
549	0.12928	0.10381	0.09095	0.08958	0.11472
548	0.12754	0.10307	0.09059	0.08880	0.11322
547	0.12596	0.10237	0.09033	0.08813	0.11183
546	0.12462	0.10169	0.08998	0.08754	0.11061
545	0.12345	0.10109	0.08970	0.08688	0.10960
544	0.12244	0.10046	0.08941	0.08624	0.10866
543	0.12165	0.10001	0.08914	0.08570	0.10795
542	0.12105	0.09957	0.08888	0.08524	0.10728
541	0.12051	0.09911	0.08883	0.08485	0.10669
540	0.12012	0.09878	0.08864	0.08451	0.10629
539	0.11981	0.09854	0.08853	0.08423	0.10606
538	0.11967	0.09827	0.08845	0.08395	0.10585
537	0.11953	0.09804	0.08822	0.08371	0.10564
536	0.11953	0.09788	0.08808	0.08347	0.10552

535	0.11961	0.09776	0.08803	0.08326	0.10549
534	0.11970	0.09774	0.08805	0.08313	0.10554
533	0.11989	0.09771	0.08812	0.08312	0.10561
532	0.12016	0.09772	0.08814	0.08313	0.10569
531	0.12058	0.09772	0.08808	0.08317	0.10590
530	0.12104	0.09781	0.08802	0.08316	0.10619
529	0.12152	0.09793	0.08807	0.08318	0.10662
528	0.12215	0.09815	0.08819	0.08326	0.10705
527	0.12291	0.09836	0.08843	0.08347	0.10756
526	0.12370	0.09855	0.08861	0.08370	0.10819
525	0.12457	0.09873	0.08886	0.08401	0.10887
524	0.12558	0.09904	0.08898	0.08428	0.10953
523	0.12661	0.09936	0.08908	0.08455	0.11032
522	0.12770	0.09966	0.08918	0.08482	0.11109
521	0.12876	0.09994	0.08937	0.08500	0.11189
520	0.13003	0.10025	0.08957	0.08537	0.11273
519	0.13107	0.10062	0.08987	0.08573	0.11353
518	0.13222	0.10092	0.09005	0.08603	0.11423
517	0.13326	0.10116	0.09013	0.08634	0.11491
516	0.13428	0.10134	0.09025	0.08650	0.11555
515	0.13533	0.10155	0.09043	0.08669	0.11616
514	0.13629	0.10178	0.09064	0.08692	0.11684
513	0.13713	0.10193	0.09085	0.08716	0.11753
512	0.13787	0.10203	0.09098	0.08741	0.11806
511	0.13860	0.10229	0.09118	0.08757	0.11858
510	0.13919	0.10243	0.09124	0.08770	0.11912
509	0.13948	0.10251	0.09122	0.08770	0.11937
508	0.13955	0.10253	0.09140	0.08770	0.11956
507	0.13944	0.10257	0.09163	0.08776	0.11950
506	0.13907	0.10255	0.09174	0.08782	0.11927
505	0.13827	0.10250	0.09176	0.08777	0.11883
504	0.13741	0.10240	0.09159	0.08760	0.11826

503	0.13636	0.10225	0.09154	0.08737	0.11742
502	0.13526	0.10202	0.09145	0.08706	0.11652
501	0.13401	0.10174	0.09148	0.08675	0.11550
500	0.13281	0.10144	0.09145	0.08647	0.11443
499	0.13167	0.10112	0.09133	0.08619	0.11341
498	0.13051	0.10085	0.09121	0.08583	0.11242
497	0.12950	0.10053	0.09094	0.08543	0.11156
496	0.12868	0.10019	0.09079	0.08510	0.11080
495	0.12783	0.09993	0.09079	0.08478	0.11009
494	0.12712	0.09962	0.09082	0.08451	0.10945
493	0.12652	0.09934	0.09068	0.08430	0.10891
492	0.12599	0.09915	0.09048	0.08402	0.10849
491	0.12557	0.09893	0.09040	0.08373	0.10818
490	0.12526	0.09875	0.09029	0.08365	0.10791
489	0.12501	0.09870	0.09038	0.08354	0.10762
488	0.12476	0.09867	0.09052	0.08350	0.10749
487	0.12472	0.09864	0.09049	0.08339	0.10751
486	0.12469	0.09873	0.09044	0.08340	0.10756
485	0.12465	0.09881	0.09053	0.08339	0.10769
484	0.12478	0.09893	0.09071	0.08341	0.10787
483	0.12497	0.09913	0.09094	0.08355	0.10814
482	0.12521	0.09938	0.09110	0.08373	0.10849
481	0.12569	0.09971	0.09120	0.08388	0.10889
480	0.12640	0.10009	0.09139	0.08413	0.10944
479	0.12719	0.10052	0.09159	0.08433	0.11005
478	0.12818	0.10100	0.09188	0.08455	0.11072
477	0.12949	0.10162	0.09227	0.08502	0.11149
476	0.13088	0.10219	0.09274	0.08551	0.11231
475	0.13258	0.10287	0.09302	0.08588	0.11324
474	0.13444	0.10363	0.09328	0.08630	0.11429
473	0.13640	0.10442	0.09381	0.08673	0.11534
472	0.13859	0.10527	0.09429	0.08733	0.11652

471	0.14076	0.10603	0.09480	0.08785	0.11768
470	0.14296	0.10689	0.09517	0.08839	0.11885
469	0.14509	0.10769	0.09550	0.08882	0.11986
468	0.14705	0.10852	0.09596	0.08931	0.12080
467	0.14871	0.10926	0.09633	0.08976	0.12159
466	0.15018	0.10987	0.09682	0.09024	0.12231
465	0.15128	0.11048	0.09721	0.09063	0.12285
464	0.15192	0.11098	0.09744	0.09092	0.12322
463	0.15228	0.11142	0.09764	0.09115	0.12354
462	0.15230	0.11172	0.09802	0.09133	0.12369
461	0.15215	0.11194	0.09830	0.09157	0.12381
460	0.15175	0.11210	0.09849	0.09172	0.12374
459	0.15114	0.11221	0.09860	0.09172	0.12364
458	0.15053	0.11217	0.09865	0.09178	0.12356
457	0.14973	0.11211	0.09882	0.09182	0.12318
456	0.14889	0.11202	0.09894	0.09184	0.12289
455	0.14794	0.11190	0.09893	0.09179	0.12251
454	0.14689	0.11171	0.09891	0.09170	0.12212
453	0.14576	0.11141	0.09889	0.09147	0.12154
452	0.14460	0.11105	0.09888	0.09127	0.12087
451	0.14333	0.11079	0.09883	0.09116	0.12029
450	0.14209	0.11037	0.09867	0.09101	0.11971
449	0.14093	0.10989	0.09858	0.09069	0.11922
448	0.13987	0.10959	0.09847	0.09038	0.11862
447	0.13885	0.10930	0.09832	0.09020	0.11820
446	0.13798	0.10897	0.09841	0.09010	0.11781
445	0.13738	0.10869	0.09841	0.08992	0.11749
444	0.13680	0.10849	0.09830	0.08982	0.11718
443	0.13641	0.10832	0.09822	0.08962	0.11684
442	0.13612	0.10826	0.09825	0.08957	0.11673
441	0.13582	0.10813	0.09840	0.08955	0.11668
440	0.13580	0.10807	0.09849	0.08949	0.11663

439	0.13583	0.10802	0.09850	0.08946	0.11666
438	0.13592	0.10812	0.09865	0.08948	0.11686
437	0.13619	0.10821	0.09890	0.08955	0.11706
436	0.13642	0.10835	0.09904	0.08963	0.11727
435	0.13674	0.10854	0.09909	0.08972	0.11758
434	0.13715	0.10871	0.09924	0.08972	0.11787
433	0.13760	0.10900	0.09942	0.08989	0.11828
432	0.13810	0.10932	0.09976	0.09005	0.11861
431	0.13865	0.10956	0.10007	0.09030	0.11900
430	0.13926	0.10985	0.10023	0.09049	0.11944
429	0.13990	0.11013	0.10043	0.09061	0.11996
428	0.14060	0.11052	0.10071	0.09085	0.12056
427	0.14136	0.11085	0.10109	0.09109	0.12123
426	0.14222	0.11121	0.10138	0.09128	0.12181
425	0.14327	0.11165	0.10174	0.09146	0.12244
424	0.14437	0.11221	0.10207	0.09171	0.12322
423	0.14546	0.11268	0.10238	0.09202	0.12389
422	0.14667	0.11321	0.10263	0.09234	0.12460
421	0.14799	0.11394	0.10309	0.09256	0.12542
420	0.14931	0.11458	0.10357	0.09280	0.12630
419	0.15089	0.11528	0.10411	0.09315	0.12706
418	0.15249	0.11582	0.10449	0.09358	0.12785
417	0.15411	0.11660	0.10487	0.09387	0.12878
416	0.15563	0.11749	0.10547	0.09423	0.12973
415	0.15742	0.11826	0.10599	0.09462	0.13065
414	0.15893	0.11902	0.10651	0.09499	0.13156
413	0.16022	0.11989	0.10695	0.09531	0.13242
412	0.16143	0.12074	0.10749	0.09567	0.13321
411	0.16262	0.12150	0.10806	0.09614	0.13390
410	0.16363	0.12226	0.10854	0.09653	0.13455
409	0.16447	0.12302	0.10887	0.09691	0.13550
408	0.16516	0.12378	0.10930	0.09720	0.13628

407	0.16590	0.12443	0.10975	0.09750	0.13680
406	0.16640	0.12507	0.11019	0.09781	0.13739
405	0.16696	0.12578	0.11055	0.09802	0.13808
404	0.16738	0.12639	0.11096	0.09827	0.13842
403	0.16774	0.12687	0.11143	0.09852	0.13883
402	0.16802	0.12737	0.11173	0.09875	0.13917
401	0.16809	0.12779	0.11200	0.09887	0.13956
400	0.16797	0.12806	0.11230	0.09895	0.13980

Wavelength	SWNT-$(CF_2)_3CF_3$	SWNT>$(CH_2)_4$	SWNT>$CH_2(CF_2)_2CH_2$	SWNT>$(CF_2)_4$
1300	0.02846	0.05180	0.01021	0.03055
1299	0.02850	0.05182	0.01064	0.03052
1298	0.02862	0.05192	0.01087	0.03057
1297	0.02880	0.05212	0.01117	0.03064
1296	0.02891	0.05219	0.01142	0.03066
1295	0.02901	0.05232	0.01168	0.03069
1294	0.02911	0.05245	0.01196	0.03078
1293	0.02922	0.05258	0.01221	0.03081
1292	0.02934	0.05269	0.01245	0.03083
1291	0.02948	0.05290	0.01260	0.03093
1290	0.02962	0.05299	0.01279	0.03093
1289	0.02974	0.05313	0.01295	0.03090
1288	0.02989	0.05323	0.01309	0.03093
1287	0.02997	0.05341	0.01331	0.03098
1286	0.03012	0.05357	0.01353	0.03106
1285	0.03029	0.05380	0.01373	0.03114
1284	0.03048	0.05395	0.01389	0.03109
1283	0.03068	0.05408	0.01409	0.03110
1282	0.03083	0.05434	0.01423	0.03121
1281	0.03096	0.05448	0.01448	0.03125
1280	0.03114	0.05463	0.01462	0.03131

1279	0.03126	0.05480	0.01490	0.03138
1278	0.03143	0.05497	0.01512	0.03143
1277	0.03161	0.05526	0.01522	0.03145
1276	0.03178	0.05529	0.01541	0.03156
1275	0.03201	0.05544	0.01554	0.03157
1274	0.03216	0.05566	0.01564	0.03169
1273	0.03223	0.05581	0.01582	0.03168
1272	0.03249	0.05607	0.01583	0.03166
1271	0.03259	0.05623	0.01591	0.03181
1270	0.03281	0.05640	0.01591	0.03197
1269	0.03293	0.05661	0.01593	0.03200
1268	0.03309	0.05677	0.01588	0.03202
1267	0.03323	0.05702	0.01578	0.03203
1266	0.03328	0.05722	0.01571	0.03215
1265	0.03353	0.05743	0.01560	0.03222
1264	0.03362	0.05768	0.01544	0.03224
1263	0.03373	0.05791	0.01525	0.03229
1262	0.03384	0.05811	0.01509	0.03237
1261	0.03392	0.05832	0.01489	0.03242
1260	0.03410	0.05853	0.01465	0.03247
1259	0.03414	0.05872	0.01442	0.03252
1258	0.03425	0.05901	0.01415	0.03250
1257	0.03430	0.05914	0.01397	0.03252
1256	0.03439	0.05933	0.01374	0.03256
1255	0.03453	0.05954	0.01339	0.03264
1254	0.03456	0.05975	0.01311	0.03267
1253	0.03462	0.05999	0.01289	0.03266
1252	0.03467	0.06017	0.01263	0.03281
1251	0.03475	0.06037	0.01229	0.03283
1250	0.03478	0.06059	0.01204	0.03281
1249	0.03483	0.06075	0.01174	0.03287
1248	0.03487	0.06094	0.01150	0.03295

1247	0.03483	0.06112	0.01126	0.03298
1246	0.03498	0.06123	0.01100	0.03297
1245	0.03503	0.06146	0.01067	0.03302
1244	0.03501	0.06166	0.01044	0.03307
1243	0.03506	0.06173	0.01017	0.03310
1242	0.03506	0.06180	0.00991	0.03325
1241	0.03511	0.06201	0.00963	0.03327
1240	0.03507	0.06214	0.00939	0.03330
1239	0.03516	0.06225	0.00919	0.03328
1238	0.03513	0.06229	0.00903	0.03340
1237	0.03517	0.06237	0.00887	0.03346
1236	0.03521	0.06243	0.00875	0.03350
1235	0.03521	0.06248	0.00868	0.03354
1234	0.03520	0.06249	0.00853	0.03358
1233	0.03530	0.06257	0.00855	0.03364
1232	0.03538	0.06264	0.00851	0.03373
1231	0.03538	0.06268	0.00852	0.03380
1230	0.03547	0.06271	0.00852	0.03393
1229	0.03565	0.06271	0.00849	0.03405
1228	0.03571	0.06276	0.00856	0.03417
1227	0.03580	0.06279	0.00857	0.03421
1226	0.03593	0.06272	0.00858	0.03424
1225	0.03602	0.06266	0.00866	0.03444
1224	0.03619	0.06269	0.00881	0.03465
1223	0.03624	0.06267	0.00890	0.03482
1222	0.03645	0.06261	0.00902	0.03499
1221	0.03665	0.06255	0.00910	0.03512
1220	0.03683	0.06256	0.00927	0.03530
1219	0.03704	0.06257	0.00936	0.03544
1218	0.03724	0.06250	0.00954	0.03560
1217	0.03752	0.06239	0.00974	0.03590
1216	0.03758	0.06225	0.00998	0.03610

1215	0.03789	0.06221	0.01018	0.03626
1214	0.03820	0.06215	0.01043	0.03650
1213	0.03845	0.06206	0.01061	0.03674
1212	0.03878	0.06202	0.01089	0.03702
1211	0.03903	0.06195	0.01115	0.03723
1210	0.03933	0.06190	0.01146	0.03747
1209	0.03960	0.06181	0.01185	0.03776
1208	0.03994	0.06170	0.01211	0.03801
1207	0.04027	0.06156	0.01241	0.03827
1206	0.04060	0.06150	0.01273	0.03855
1205	0.04089	0.06145	0.01312	0.03885
1204	0.04127	0.06133	0.01348	0.03920
1203	0.04158	0.06129	0.01386	0.03952
1202	0.04193	0.06119	0.01423	0.03988
1201	0.04231	0.06113	0.01466	0.04020
1200	0.04275	0.06104	0.01521	0.04049
1199	0.04311	0.06089	0.01563	0.04087
1198	0.04345	0.06087	0.01603	0.04112
1197	0.04389	0.06082	0.01650	0.04146
1196	0.04420	0.06080	0.01692	0.04190
1195	0.04463	0.06070	0.01738	0.04213
1194	0.04510	0.06066	0.01791	0.04249
1193	0.04555	0.06064	0.01843	0.04288
1192	0.04600	0.06056	0.01899	0.04329
1191	0.04653	0.06057	0.01950	0.04367
1190	0.04698	0.06051	0.02007	0.04395
1189	0.04745	0.06049	0.02070	0.04427
1188	0.04804	0.06047	0.02130	0.04465
1187	0.04861	0.06049	0.02195	0.04511
1186	0.04916	0.06043	0.02274	0.04543
1185	0.04978	0.06042	0.02345	0.04584
1184	0.05029	0.06044	0.02426	0.04622

1183	0.05087	0.06043	0.02503	0.04660
1182	0.05154	0.06038	0.02595	0.04711
1181	0.05216	0.06032	0.02689	0.04752
1180	0.05274	0.06029	0.02785	0.04797
1179	0.05332	0.06033	0.02898	0.04843
1178	0.05396	0.06022	0.03016	0.04890
1177	0.05456	0.06031	0.03139	0.04936
1176	0.05513	0.06026	0.03264	0.04980
1175	0.05574	0.06022	0.03404	0.05024
1174	0.05638	0.06020	0.03542	0.05072
1173	0.05694	0.06017	0.03688	0.05120
1172	0.05749	0.06018	0.03826	0.05167
1171	0.05808	0.06024	0.03971	0.05211
1170	0.05865	0.06022	0.04099	0.05248
1169	0.05914	0.06024	0.04228	0.05296
1168	0.05972	0.06024	0.04352	0.05334
1167	0.06020	0.06034	0.04461	0.05374
1166	0.06078	0.06037	0.04567	0.05420
1165	0.06124	0.06040	0.04669	0.05453
1164	0.06167	0.06048	0.04755	0.05488
1163	0.06224	0.06056	0.04831	0.05530
1162	0.06265	0.06064	0.04918	0.05571
1161	0.06308	0.06072	0.04992	0.05610
1160	0.06342	0.06090	0.05054	0.05645
1159	0.06391	0.06097	0.05131	0.05678
1158	0.06430	0.06108	0.05202	0.05720
1157	0.06478	0.06121	0.05273	0.05756
1156	0.06528	0.06140	0.05346	0.05795
1155	0.06574	0.06147	0.05429	0.05832
1154	0.06610	0.06160	0.05500	0.05864
1153	0.06671	0.06178	0.05582	0.05902
1152	0.06724	0.06197	0.05657	0.05943

1151	0.06776	0.06208	0.05740	0.05988
1150	0.06829	0.06226	0.05834	0.06024
1149	0.06882	0.06243	0.05925	0.06062
1148	0.06935	0.06257	0.06018	0.06100
1147	0.06990	0.06278	0.06098	0.06145
1146	0.07036	0.06295	0.06184	0.06188
1145	0.07075	0.06317	0.06272	0.06222
1144	0.07131	0.06340	0.06352	0.06257
1143	0.07167	0.06354	0.06428	0.06299
1142	0.07214	0.06378	0.06505	0.06340
1141	0.07248	0.06395	0.06577	0.06365
1140	0.07276	0.06416	0.06635	0.06407
1139	0.07306	0.06441	0.06692	0.06441
1138	0.07339	0.06460	0.06764	0.06467
1137	0.07371	0.06481	0.06814	0.06498
1136	0.07402	0.06510	0.06866	0.06528
1135	0.07426	0.06537	0.06922	0.06553
1134	0.07457	0.06561	0.06971	0.06591
1133	0.07496	0.06588	0.07021	0.06627
1132	0.07533	0.06614	0.07072	0.06659
1131	0.07564	0.06642	0.07126	0.06691
1130	0.07603	0.06664	0.07180	0.06724
1129	0.07649	0.06689	0.07233	0.06762
1128	0.07684	0.06726	0.07286	0.06792
1127	0.07728	0.06750	0.07341	0.06827
1126	0.07773	0.06782	0.07396	0.06858
1125	0.07809	0.06810	0.07440	0.06894
1124	0.07841	0.06847	0.07493	0.06925
1123	0.07869	0.06862	0.07538	0.06954
1122	0.07897	0.06898	0.07575	0.06989
1121	0.07907	0.06927	0.07606	0.07012
1120	0.07926	0.06947	0.07651	0.07037

1119	0.07926	0.06973	0.07670	0.07044
1118	0.07920	0.06999	0.07687	0.07054
1117	0.07915	0.07028	0.07696	0.07065
1116	0.07899	0.07047	0.07696	0.07068
1115	0.07867	0.07060	0.07688	0.07064
1114	0.07829	0.07077	0.07668	0.07059
1113	0.07781	0.07090	0.07650	0.07037
1112	0.07734	0.07111	0.07612	0.07027
1111	0.07673	0.07119	0.07566	0.06996
1110	0.07598	0.07134	0.07516	0.06972
1109	0.07526	0.07131	0.07457	0.06944
1108	0.07443	0.07139	0.07391	0.06907
1107	0.07358	0.07147	0.07317	0.06867
1106	0.07263	0.07139	0.07243	0.06814
1105	0.07171	0.07137	0.07164	0.06768
1104	0.07085	0.07133	0.07085	0.06721
1103	0.06995	0.07126	0.07006	0.06670
1102	0.06906	0.07118	0.06931	0.06620
1101	0.06809	0.07108	0.06857	0.06578
1100	0.06731	0.07096	0.06782	0.06536
1099	0.06653	0.07084	0.06712	0.06494
1098	0.06581	0.07076	0.06650	0.06454
1097	0.06521	0.07066	0.06601	0.06416
1096	0.06460	0.07054	0.06546	0.06391
1095	0.06411	0.07038	0.06498	0.06373
1094	0.06360	0.07027	0.06457	0.06334
1093	0.06321	0.07015	0.06426	0.06313
1092	0.06286	0.07002	0.06398	0.06298
1091	0.06260	0.06989	0.06380	0.06289
1090	0.06240	0.06980	0.06361	0.06288
1089	0.06221	0.06967	0.06351	0.06288
1088	0.06215	0.06955	0.06345	0.06282

1087	0.06207	0.06943	0.06345	0.06286
1086	0.06200	0.06945	0.06341	0.06304
1085	0.06208	0.06934	0.06357	0.06321
1084	0.06211	0.06923	0.06374	0.06338
1083	0.06231	0.06910	0.06389	0.06366
1082	0.06254	0.06905	0.06406	0.06398
1081	0.06269	0.06897	0.06444	0.06424
1080	0.06295	0.06893	0.06474	0.06449
1079	0.06324	0.06882	0.06503	0.06500
1078	0.06359	0.06883	0.06549	0.06538
1077	0.06395	0.06885	0.06592	0.06573
1076	0.06431	0.06877	0.06630	0.06628
1075	0.06479	0.06879	0.06685	0.06680
1074	0.06529	0.06882	0.06741	0.06744
1073	0.06579	0.06877	0.06798	0.06807
1072	0.06629	0.06880	0.06858	0.06863
1071	0.06692	0.06888	0.06921	0.06928
1070	0.06754	0.06895	0.06989	0.06997
1069	0.06823	0.06895	0.07066	0.07078
1068	0.06889	0.06903	0.07143	0.07154
1067	0.06977	0.06911	0.07222	0.07246
1066	0.07061	0.06921	0.07311	0.07325
1065	0.07131	0.06926	0.07408	0.07410
1064	0.07222	0.06934	0.07507	0.07511
1063	0.07304	0.06949	0.07598	0.07603
1062	0.07387	0.06962	0.07703	0.07699
1061	0.07476	0.06968	0.07802	0.07805
1060	0.07556	0.06983	0.07904	0.07899
1059	0.07642	0.06994	0.08014	0.08002
1058	0.07722	0.07001	0.08111	0.08099
1057	0.07806	0.07014	0.08219	0.08206
1056	0.07894	0.07026	0.08334	0.08312

1055	0.07975	0.07038	0.08441	0.08420
1054	0.08063	0.07058	0.08547	0.08530
1053	0.08157	0.07069	0.08656	0.08647
1052	0.08246	0.07081	0.08773	0.08766
1051	0.08347	0.07100	0.08894	0.08878
1050	0.08456	0.07113	0.09021	0.09004
1049	0.08566	0.07134	0.09148	0.09129
1048	0.08677	0.07148	0.09273	0.09255
1047	0.08785	0.07166	0.09412	0.09396
1046	0.08907	0.07187	0.09559	0.09528
1045	0.09027	0.07200	0.09714	0.09668
1044	0.09161	0.07219	0.09860	0.09820
1043	0.09296	0.07239	0.10015	0.09977
1042	0.09443	0.07265	0.10175	0.10137
1041	0.09605	0.07288	0.10352	0.10310
1040	0.09770	0.07306	0.10543	0.10486
1039	0.09959	0.07332	0.10733	0.10678
1038	0.10159	0.07365	0.10955	0.10873
1037	0.10384	0.07401	0.11181	0.11088
1036	0.10626	0.07428	0.11436	0.11313
1035	0.10890	0.07470	0.11704	0.11548
1034	0.11155	0.07516	0.11992	0.11788
1033	0.11438	0.07548	0.12289	0.12035
1032	0.11737	0.07592	0.12597	0.12293
1031	0.12033	0.07638	0.12913	0.12545
1030	0.12333	0.07676	0.13235	0.12801
1029	0.12615	0.07720	0.13555	0.13058
1028	0.12902	0.07767	0.13864	0.13307
1027	0.13166	0.07812	0.14161	0.13551
1026	0.13410	0.07859	0.14456	0.13787
1025	0.13639	0.07900	0.14735	0.13993
1024	0.13847	0.07931	0.14982	0.14209

1023	0.14042	0.07977	0.15215	0.14419
1022	0.14212	0.08015	0.15434	0.14615
1021	0.14379	0.08045	0.15637	0.14803
1020	0.14536	0.08077	0.15833	0.14991
1019	0.14699	0.08114	0.16025	0.15179
1018	0.14857	0.08132	0.16217	0.15364
1017	0.15032	0.08165	0.16404	0.15565
1016	0.15200	0.08192	0.16606	0.15774
1015	0.15393	0.08218	0.16818	0.15982
1014	0.15607	0.08242	0.17043	0.16207
1013	0.15844	0.08275	0.17284	0.16453
1012	0.16113	0.08299	0.17558	0.16716
1011	0.16414	0.08320	0.17852	0.16980
1010	0.16745	0.08338	0.18175	0.17261
1009	0.17096	0.08363	0.18517	0.17576
1008	0.17490	0.08392	0.18883	0.17902
1007	0.17913	0.08426	0.19266	0.18235
1006	0.18364	0.08454	0.19668	0.18594
1005	0.18835	0.08472	0.20084	0.18960
1004	0.19311	0.08505	0.20516	0.19330
1003	0.19816	0.08541	0.20934	0.19734
1002	0.20333	0.08568	0.21368	0.20121
1001	0.20863	0.08608	0.21796	0.20522
1000	0.21386	0.08644	0.22211	0.20929
999	0.21912	0.08680	0.22606	0.21335
998	0.22443	0.08721	0.22982	0.21735
997	0.22972	0.08764	0.23340	0.22136
996	0.23499	0.08799	0.23685	0.22530
995	0.24040	0.08842	0.24017	0.22930
994	0.24588	0.08889	0.24340	0.23322
993	0.25146	0.08932	0.24654	0.23722
992	0.25715	0.08989	0.24996	0.24128

991	0.26302	0.09039	0.25330	0.24539
990	0.26886	0.09101	0.25681	0.24967
989	0.27488	0.09159	0.26058	0.25390
988	0.28094	0.09217	0.26449	0.25834
987	0.28697	0.09281	0.26865	0.26280
986	0.29287	0.09337	0.27282	0.26728
985	0.29866	0.09401	0.27719	0.27172
984	0.30390	0.09474	0.28149	0.27587
983	0.30863	0.09541	0.28558	0.27981
982	0.31245	0.09599	0.28917	0.28349
981	0.31543	0.09664	0.29212	0.28647
980	0.31701	0.09732	0.29427	0.28874
979	0.31726	0.09783	0.29541	0.29019
978	0.31616	0.09831	0.29539	0.29065
977	0.31343	0.09871	0.29389	0.29009
976	0.30921	0.09902	0.29106	0.28840
975	0.30356	0.09933	0.28683	0.28564
974	0.29674	0.09963	0.28145	0.28184
973	0.28881	0.09977	0.27498	0.27726
972	0.28019	0.09979	0.26775	0.27170
971	0.27089	0.09980	0.25984	0.26554
970	0.26143	0.09982	0.25154	0.25900
969	0.25191	0.09974	0.24316	0.25219
968	0.24267	0.09954	0.23480	0.24525
967	0.23403	0.09935	0.22677	0.23830
966	0.22600	0.09913	0.21920	0.23167
965	0.21860	0.09879	0.21220	0.22539
964	0.21219	0.09850	0.20601	0.21946
963	0.20644	0.09821	0.20057	0.21395
962	0.20160	0.09787	0.19579	0.20886
961	0.19733	0.09744	0.19177	0.20411
960	0.19363	0.09711	0.18834	0.19976

959	0.19019	0.09675	0.18528	0.19560
958	0.18698	0.09629	0.18256	0.19170
957	0.18385	0.09590	0.18013	0.18790
956	0.18054	0.09540	0.17780	0.18408
955	0.17705	0.09497	0.17534	0.18015
954	0.17334	0.09450	0.17267	0.17634
953	0.16927	0.09402	0.16978	0.17232
952	0.16502	0.09349	0.16661	0.16827
951	0.16048	0.09297	0.16321	0.16415
950	0.15579	0.09242	0.15970	0.16002
949	0.15112	0.09190	0.15594	0.15589
948	0.14635	0.09138	0.15205	0.15188
947	0.14181	0.09083	0.14821	0.14801
946	0.13738	0.09032	0.14441	0.14414
945	0.13328	0.08983	0.14076	0.14055
944	0.12931	0.08935	0.13713	0.13720
943	0.12576	0.08887	0.13386	0.13393
942	0.12253	0.08832	0.13081	0.13101
941	0.11950	0.08782	0.12785	0.12827
940	0.11678	0.08731	0.12522	0.12568
939	0.11427	0.08680	0.12273	0.12338
938	0.11206	0.08642	0.12065	0.12126
937	0.11001	0.08607	0.11874	0.11923
936	0.10818	0.08565	0.11696	0.11744
935	0.10659	0.08518	0.11540	0.11586
934	0.10520	0.08489	0.11411	0.11435
933	0.10394	0.08448	0.11287	0.11299
932	0.10281	0.08412	0.11182	0.11183
931	0.10182	0.08376	0.11103	0.11076
930	0.10105	0.08335	0.11031	0.10999
929	0.10041	0.08304	0.10967	0.10921
928	0.09992	0.08266	0.10931	0.10855

927	0.09957	0.08241	0.10903	0.10803
926	0.09955	0.08216	0.10898	0.10774
925	0.09971	0.08185	0.10901	0.10759
924	0.09990	0.08166	0.10931	0.10750
923	0.10046	0.08146	0.10963	0.10759
922	0.10121	0.08123	0.11018	0.10775
921	0.10191	0.08097	0.11076	0.10805
920	0.10286	0.08088	0.11156	0.10835
919	0.10369	0.08069	0.11218	0.10878
918	0.10460	0.08047	0.11295	0.10915
917	0.10542	0.08038	0.11372	0.10951
916	0.10604	0.08009	0.11438	0.10977
915	0.10633	0.07995	0.11501	0.10997
914	0.10662	0.07974	0.11539	0.11005
913	0.10656	0.07957	0.11557	0.11008
912	0.10620	0.07957	0.11565	0.11002
911	0.10566	0.07942	0.11566	0.10993
910	0.10508	0.07913	0.11542	0.10962
909	0.10422	0.07900	0.11506	0.10927
908	0.10338	0.07891	0.11461	0.10897
907	0.10265	0.07882	0.11438	0.10862
906	0.10177	0.07863	0.11405	0.10839
905	0.10120	0.07857	0.11364	0.10825
904	0.10048	0.07844	0.11349	0.10797
903	0.09995	0.07829	0.11331	0.10790
902	0.09962	0.07823	0.11325	0.10792
901	0.09956	0.07811	0.11330	0.10794
900	0.09943	0.07791	0.11345	0.10814
899	0.09939	0.07780	0.11380	0.10854
898	0.09974	0.07796	0.11425	0.10898
897	0.10013	0.07786	0.11465	0.10947
896	0.10052	0.07769	0.11534	0.10993

895	0.10110	0.07760	0.11605	0.11061
894	0.10183	0.07757	0.11696	0.11145
893	0.10281	0.07756	0.11807	0.11238
892	0.10366	0.07767	0.11908	0.11319
891	0.10484	0.07770	0.12023	0.11439
890	0.10606	0.07767	0.12163	0.11547
889	0.10743	0.07761	0.12311	0.11684
888	0.10898	0.07768	0.12473	0.11830
887	0.11070	0.07778	0.12664	0.11974
886	0.11267	0.07777	0.12855	0.12130
885	0.11481	0.07788	0.13101	0.12304
884	0.11696	0.07787	0.13339	0.12478
883	0.11927	0.07785	0.13602	0.12671
882	0.12178	0.07791	0.13867	0.12863
881	0.12449	0.07801	0.14140	0.13058
880	0.12720	0.07803	0.14432	0.13245
879	0.12984	0.07810	0.14697	0.13428
878	0.13228	0.07823	0.14930	0.13608
877	0.13448	0.07831	0.15140	0.13758
876	0.13631	0.07845	0.15338	0.13865
875	0.13770	0.07839	0.15458	0.13965
874	0.13862	0.07835	0.15523	0.13995
873	0.13906	0.07843	0.15552	0.13996
872	0.13883	0.07851	0.15504	0.13975
871	0.13813	0.07852	0.15383	0.13887
870	0.13679	0.07869	0.15211	0.13760
869	0.13515	0.07866	0.15016	0.13607
868	0.13300	0.07849	0.14759	0.13418
867	0.13076	0.07861	0.14485	0.13193
866	0.12819	0.07872	0.14176	0.12965
865	0.12564	0.07864	0.13863	0.12732
864	0.12301	0.07850	0.13572	0.12506

863	0.12042	0.07855	0.13265	0.12282
862	0.11806	0.07849	0.12976	0.12076
861	0.11588	0.07864	0.12700	0.11848
860	0.11373	0.07861	0.12436	0.11670
859	0.11187	0.07850	0.12202	0.11493
858	0.11012	0.07859	0.11980	0.11322
857	0.10856	0.07857	0.11815	0.11173
856	0.10718	0.07859	0.11643	0.11044
855	0.10582	0.07870	0.11500	0.10912
854	0.10472	0.07864	0.11359	0.10789
853	0.10370	0.07854	0.11227	0.10692
852	0.10289	0.07856	0.11115	0.10603
851	0.10181	0.07870	0.11024	0.10518
850	0.10220	0.07780	0.11012	0.10442
849	0.10122	0.07758	0.10928	0.10339
848	0.10049	0.07743	0.10826	0.10247
847	0.09974	0.07745	0.10754	0.10200
846	0.09917	0.07753	0.10717	0.10142
845	0.09855	0.07766	0.10638	0.10074
844	0.09781	0.07742	0.10578	0.10025
843	0.09723	0.07735	0.10514	0.09940
842	0.09662	0.07746	0.10455	0.09901
841	0.09590	0.07738	0.10400	0.09848
840	0.09560	0.07731	0.10343	0.09783
839	0.09490	0.07736	0.10298	0.09726
838	0.09444	0.07738	0.10265	0.09668
837	0.09387	0.07736	0.10235	0.09622
836	0.09327	0.07718	0.10196	0.09584
835	0.09296	0.07717	0.10162	0.09540
834	0.09249	0.07717	0.10126	0.09483
833	0.09220	0.07709	0.10084	0.09437
832	0.09190	0.07694	0.10062	0.09414

831	0.09149	0.07690	0.10052	0.09382
830	0.09123	0.07691	0.10033	0.09331
829	0.09098	0.07698	0.10001	0.09297
828	0.09077	0.07704	0.09962	0.09265
827	0.09043	0.07690	0.09936	0.09220
826	0.09003	0.07675	0.09886	0.09189
825	0.08959	0.07671	0.09833	0.09121
824	0.08894	0.07651	0.09776	0.09048
823	0.08838	0.07650	0.09711	0.08999
822	0.08779	0.07657	0.09643	0.08934
821	0.08719	0.07625	0.09560	0.08867
820	0.08651	0.07628	0.09474	0.08794
819	0.08592	0.07611	0.09390	0.08717
818	0.08517	0.07607	0.09302	0.08634
817	0.08438	0.07611	0.09219	0.08554
816	0.08379	0.07614	0.09141	0.08473
815	0.08300	0.07605	0.09053	0.08403
814	0.08233	0.07586	0.08967	0.08330
813	0.08144	0.07573	0.08887	0.08246
812	0.08084	0.07566	0.08819	0.08161
811	0.08023	0.07561	0.08740	0.08091
810	0.07951	0.07554	0.08662	0.08029
809	0.07881	0.07552	0.08598	0.07953
808	0.07817	0.07561	0.08531	0.07874
807	0.07762	0.07543	0.08468	0.07818
806	0.07712	0.07535	0.08401	0.07764
805	0.07668	0.07532	0.08346	0.07701
804	0.07611	0.07516	0.08292	0.07654
803	0.07550	0.07528	0.08249	0.07601
802	0.07504	0.07510	0.08210	0.07539
801	0.07465	0.07502	0.08162	0.07489
800	0.07409	0.07503	0.08120	0.07454

799	0.07377	0.07499	0.08078	0.07416
798	0.07330	0.07491	0.08032	0.07374
797	0.07301	0.07480	0.07998	0.07327
796	0.07262	0.07485	0.07973	0.07287
795	0.07230	0.07477	0.07939	0.07247
794	0.07197	0.07474	0.07894	0.07206
793	0.07165	0.07471	0.07866	0.07168
792	0.07138	0.07453	0.07847	0.07137
791	0.07111	0.07449	0.07815	0.07104
790	0.07092	0.07443	0.07795	0.07075
789	0.07059	0.07451	0.07775	0.07043
788	0.07036	0.07453	0.07753	0.07023
787	0.07007	0.07444	0.07744	0.07008
786	0.06988	0.07436	0.07722	0.06997
785	0.06973	0.07441	0.07706	0.06975
784	0.06972	0.07440	0.07703	0.06958
783	0.06964	0.07427	0.07690	0.06938
782	0.06950	0.07427	0.07672	0.06932
781	0.06933	0.07427	0.07676	0.06921
780	0.06937	0.07413	0.07680	0.06907
779	0.06943	0.07421	0.07684	0.06901
778	0.06941	0.07421	0.07693	0.06907
777	0.06952	0.07413	0.07688	0.06914
776	0.06967	0.07416	0.07711	0.06928
775	0.06981	0.07419	0.07729	0.06922
774	0.06991	0.07414	0.07726	0.06933
773	0.06998	0.07423	0.07733	0.06940
772	0.07024	0.07413	0.07757	0.06948
771	0.07038	0.07414	0.07760	0.06956
770	0.07045	0.07421	0.07785	0.06967
769	0.07058	0.07420	0.07796	0.06978
768	0.07078	0.07428	0.07813	0.06989

767	0.07094	0.07430	0.07824	0.06997
766	0.07112	0.07435	0.07831	0.06998
765	0.07122	0.07439	0.07847	0.07006
764	0.07137	0.07434	0.07859	0.07022
763	0.07139	0.07431	0.07864	0.07020
762	0.07135	0.07446	0.07859	0.07019
761	0.07148	0.07448	0.07873	0.07011
760	0.07157	0.07448	0.07880	0.07015
759	0.07160	0.07456	0.07869	0.07012
758	0.07157	0.07470	0.07879	0.07012
757	0.07162	0.07470	0.07887	0.07013
756	0.07175	0.07476	0.07873	0.07012
755	0.07174	0.07475	0.07878	0.07015
754	0.07174	0.07483	0.07873	0.07013
753	0.07174	0.07494	0.07872	0.07014
752	0.07181	0.07498	0.07876	0.07009
751	0.07184	0.07502	0.07887	0.07020
750	0.07175	0.07510	0.07882	0.07017
749	0.07192	0.07521	0.07888	0.07016
748	0.07209	0.07533	0.07896	0.07026
747	0.07206	0.07534	0.07898	0.07025
746	0.07211	0.07545	0.07909	0.07025
745	0.07221	0.07550	0.07918	0.07035
744	0.07234	0.07562	0.07918	0.07036
743	0.07237	0.07570	0.07928	0.07041
742	0.07248	0.07578	0.07941	0.07054
741	0.07259	0.07591	0.07950	0.07068
740	0.07269	0.07597	0.07958	0.07067
739	0.07277	0.07620	0.07965	0.07074
738	0.07290	0.07627	0.07969	0.07078
737	0.07291	0.07634	0.07984	0.07083
736	0.07302	0.07640	0.07986	0.07088

735	0.07307	0.07648	0.07988	0.07086
734	0.07308	0.07660	0.07999	0.07084
733	0.07308	0.07669	0.08009	0.07086
732	0.07312	0.07677	0.08014	0.07087
731	0.07315	0.07675	0.08016	0.07097
730	0.07316	0.07687	0.08018	0.07098
729	0.07327	0.07699	0.08023	0.07096
728	0.07321	0.07710	0.08032	0.07097
727	0.07323	0.07719	0.08039	0.07107
726	0.07323	0.07715	0.08035	0.07108
725	0.07329	0.07719	0.08038	0.07101
724	0.07336	0.07732	0.08045	0.07101
723	0.07331	0.07731	0.08040	0.07100
722	0.07335	0.07739	0.08049	0.07112
721	0.07328	0.07747	0.08054	0.07119
720	0.07326	0.07746	0.08055	0.07124
719	0.07331	0.07749	0.08059	0.07120
718	0.07337	0.07747	0.08067	0.07119
717	0.07337	0.07745	0.08064	0.07121
716	0.07333	0.07744	0.08069	0.07134
715	0.07344	0.07748	0.08084	0.07143
714	0.07354	0.07751	0.08092	0.07155
713	0.07367	0.07746	0.08106	0.07174
712	0.07383	0.07745	0.08124	0.07183
711	0.07403	0.07756	0.08139	0.07203
710	0.07424	0.07753	0.08162	0.07225
709	0.07444	0.07747	0.08190	0.07247
708	0.07467	0.07743	0.08229	0.07271
707	0.07502	0.07750	0.08261	0.07299
706	0.07537	0.07758	0.08291	0.07336
705	0.07579	0.07754	0.08324	0.07363
704	0.07618	0.07751	0.08363	0.07398

703	0.07669	0.07759	0.08406	0.07432
702	0.07716	0.07764	0.08460	0.07468
701	0.07773	0.07773	0.08510	0.07504
700	0.07823	0.07769	0.08553	0.07550
699	0.07872	0.07774	0.08609	0.07601
698	0.07924	0.07787	0.08656	0.07641
697	0.07980	0.07792	0.08701	0.07693
696	0.08032	0.07811	0.08755	0.07736
695	0.08081	0.07825	0.08809	0.07788
694	0.08128	0.07834	0.08858	0.07844
693	0.08163	0.07840	0.08901	0.07889
692	0.08205	0.07853	0.08940	0.07940
691	0.08248	0.07872	0.08980	0.07989
690	0.08276	0.07887	0.09023	0.08041
689	0.08313	0.07917	0.09058	0.08095
688	0.08345	0.07933	0.09100	0.08143
687	0.08379	0.07949	0.09143	0.08187
686	0.08418	0.07978	0.09187	0.08241
685	0.08471	0.07998	0.09251	0.08296
684	0.08520	0.08017	0.09298	0.08347
683	0.08574	0.08034	0.09366	0.08399
682	0.08632	0.08055	0.09437	0.08461
681	0.08708	0.08078	0.09511	0.08532
680	0.08788	0.08103	0.09589	0.08598
679	0.08870	0.08123	0.09682	0.08659
678	0.08964	0.08142	0.09774	0.08716
677	0.09063	0.08160	0.09873	0.08783
676	0.09171	0.08178	0.09967	0.08889
675	0.09273	0.08206	0.10065	0.08981
674	0.09378	0.08244	0.10162	0.09068
673	0.09489	0.08274	0.10269	0.09150
672	0.09593	0.08300	0.10361	0.09241

671	0.09690	0.08332	0.10455	0.09323
670	0.09788	0.08366	0.10542	0.09402
669	0.09877	0.08396	0.10620	0.09476
668	0.09961	0.08429	0.10685	0.09542
667	0.10031	0.08467	0.10732	0.09596
666	0.10082	0.08494	0.10770	0.09642
665	0.10115	0.08522	0.10787	0.09676
664	0.10130	0.08560	0.10798	0.09701
663	0.10133	0.08596	0.10792	0.09702
662	0.10119	0.08623	0.10768	0.09703
661	0.10096	0.08664	0.10750	0.09688
660	0.10062	0.08695	0.10719	0.09672
659	0.10032	0.08725	0.10690	0.09646
658	0.10004	0.08767	0.10662	0.09635
657	0.09970	0.08801	0.10643	0.09620
656	0.09946	0.08837	0.10624	0.09603
655	0.09934	0.08874	0.10622	0.09592
654	0.09932	0.08909	0.10625	0.09593
653	0.09930	0.08948	0.10623	0.09594
652	0.09930	0.08981	0.10632	0.09591
651	0.09938	0.09004	0.10644	0.09599
650	0.09934	0.09036	0.10650	0.09593
649	0.09939	0.09057	0.10646	0.09590
648	0.09932	0.09082	0.10634	0.09580
647	0.09908	0.09108	0.10623	0.09566
646	0.09888	0.09120	0.10596	0.09557
645	0.09856	0.09129	0.10560	0.09523
644	0.09811	0.09134	0.10521	0.09479
643	0.09764	0.09129	0.10472	0.09446
642	0.09697	0.09128	0.10425	0.09400
641	0.09629	0.09120	0.10366	0.09351
640	0.09559	0.09106	0.10301	0.09301

639	0.09484	0.09099	0.10235	0.09241
638	0.09413	0.09085	0.10174	0.09179
637	0.09334	0.09069	0.10105	0.09121
636	0.09260	0.09054	0.10031	0.09061
635	0.09183	0.09032	0.09975	0.08997
634	0.09111	0.09018	0.09915	0.08948
633	0.09047	0.09005	0.09862	0.08896
632	0.08990	0.08993	0.09811	0.08854
631	0.08943	0.08972	0.09770	0.08807
630	0.08906	0.08965	0.09746	0.08778
629	0.08876	0.08948	0.09725	0.08756
628	0.08854	0.08937	0.09716	0.08742
627	0.08838	0.08940	0.09705	0.08735
626	0.08836	0.08937	0.09711	0.08742
625	0.08835	0.08936	0.09732	0.08756
624	0.08854	0.08927	0.09753	0.08780
623	0.08880	0.08927	0.09782	0.08806
622	0.08911	0.08942	0.09816	0.08836
621	0.08942	0.08947	0.09861	0.08874
620	0.08983	0.08953	0.09902	0.08926
619	0.09034	0.08963	0.09964	0.08979
618	0.09086	0.08974	0.10028	0.09036
617	0.09144	0.08993	0.10099	0.09105
616	0.09213	0.09010	0.10174	0.09181
615	0.09292	0.09031	0.10256	0.09253
614	0.09369	0.09046	0.10351	0.09342
613	0.09456	0.09069	0.10450	0.09439
612	0.09546	0.09098	0.10547	0.09539
611	0.09645	0.09128	0.10661	0.09645
610	0.09751	0.09152	0.10777	0.09760
609	0.09861	0.09185	0.10899	0.09885
608	0.09978	0.09217	0.11036	0.10018

607	0.10099	0.09253	0.11170	0.10150
606	0.10229	0.09290	0.11317	0.10287
605	0.10363	0.09331	0.11479	0.10437
604	0.10510	0.09373	0.11636	0.10589
603	0.10662	0.09417	0.11803	0.10756
602	0.10819	0.09468	0.11987	0.10929
601	0.10991	0.09515	0.12175	0.11113
600	0.11175	0.09558	0.12373	0.11310
599	0.11368	0.09617	0.12590	0.11513
598	0.11572	0.09673	0.12816	0.11728
597	0.11792	0.09741	0.13052	0.11942
596	0.12016	0.09799	0.13302	0.12178
595	0.12258	0.09861	0.13555	0.12432
594	0.12500	0.09930	0.13816	0.12674
593	0.12754	0.09993	0.14099	0.12935
592	0.13019	0.10062	0.14370	0.13197
591	0.13277	0.10130	0.14641	0.13458
590	0.13539	0.10190	0.14911	0.13716
589	0.13803	0.10248	0.15177	0.13973
588	0.14061	0.10297	0.15430	0.14232
587	0.14305	0.10352	0.15667	0.14471
586	0.14536	0.10403	0.15891	0.14702
585	0.14751	0.10440	0.16088	0.14920
584	0.14960	0.10488	0.16277	0.15119
583	0.15147	0.10542	0.16431	0.15292
582	0.15305	0.10591	0.16566	0.15452
581	0.15447	0.10636	0.16682	0.15586
580	0.15568	0.10688	0.16775	0.15705
579	0.15685	0.10734	0.16850	0.15807
578	0.15801	0.10785	0.16920	0.15912
577	0.15914	0.10841	0.16999	0.16007
576	0.16040	0.10894	0.17086	0.16102

575	0.16178	0.10943	0.17165	0.16211
574	0.16331	0.10994	0.17269	0.16319
573	0.16487	0.11047	0.17375	0.16426
572	0.16643	0.11097	0.17470	0.16534
571	0.16785	0.11139	0.17559	0.16631
570	0.16885	0.11180	0.17617	0.16695
569	0.16936	0.11212	0.17637	0.16722
568	0.16925	0.11218	0.17588	0.16698
567	0.16834	0.11219	0.17482	0.16614
566	0.16663	0.11215	0.17306	0.16462
565	0.16409	0.11196	0.17057	0.16241
564	0.16086	0.11173	0.16752	0.15965
563	0.15708	0.11134	0.16404	0.15643
562	0.15290	0.11079	0.16020	0.15276
561	0.14855	0.11027	0.15625	0.14884
560	0.14424	0.10970	0.15231	0.14492
559	0.14013	0.10912	0.14843	0.14103
558	0.13618	0.10842	0.14475	0.13732
557	0.13253	0.10778	0.14139	0.13383
556	0.12931	0.10718	0.13834	0.13060
555	0.12642	0.10657	0.13567	0.12773
554	0.12377	0.10602	0.13316	0.12511
553	0.12144	0.10553	0.13095	0.12272
552	0.11927	0.10502	0.12895	0.12047
551	0.11737	0.10460	0.12708	0.11845
550	0.11555	0.10407	0.12542	0.11656
549	0.11388	0.10363	0.12386	0.11495
548	0.11238	0.10324	0.12242	0.11336
547	0.11105	0.10288	0.12112	0.11197
546	0.10978	0.10253	0.11999	0.11080
545	0.10873	0.10225	0.11900	0.10975
544	0.10783	0.10195	0.11814	0.10877

543	0.10709	0.10168	0.11751	0.10802
542	0.10645	0.10142	0.11693	0.10738
541	0.10598	0.10123	0.11645	0.10681
540	0.10553	0.10103	0.11613	0.10637
539	0.10522	0.10092	0.11585	0.10609
538	0.10502	0.10080	0.11561	0.10591
537	0.10491	0.10075	0.11558	0.10571
536	0.10483	0.10065	0.11553	0.10551
535	0.10486	0.10057	0.11562	0.10553
534	0.10487	0.10054	0.11571	0.10554
533	0.10500	0.10054	0.11585	0.10576
532	0.10526	0.10059	0.11616	0.10596
531	0.10550	0.10055	0.11653	0.10627
530	0.10586	0.10066	0.11690	0.10667
529	0.10628	0.10084	0.11742	0.10706
528	0.10683	0.10088	0.11791	0.10751
527	0.10742	0.10097	0.11847	0.10810
526	0.10807	0.10113	0.11918	0.10879
525	0.10882	0.10135	0.11999	0.10953
524	0.10956	0.10162	0.12085	0.11034
523	0.11035	0.10176	0.12169	0.11118
522	0.11123	0.10197	0.12264	0.11203
521	0.11211	0.10220	0.12364	0.11291
520	0.11294	0.10236	0.12456	0.11376
519	0.11385	0.10268	0.12551	0.11456
518	0.11468	0.10294	0.12656	0.11549
517	0.11543	0.10317	0.12746	0.11631
516	0.11622	0.10330	0.12841	0.11706
515	0.11704	0.10349	0.12936	0.11787
514	0.11778	0.10378	0.13022	0.11856
513	0.11841	0.10397	0.13115	0.11929
512	0.11905	0.10414	0.13192	0.11986

511	0.11967	0.10427	0.13264	0.12043
510	0.12020	0.10443	0.13315	0.12082
509	0.12057	0.10461	0.13361	0.12112
508	0.12076	0.10469	0.13378	0.12118
507	0.12075	0.10480	0.13369	0.12107
506	0.12039	0.10484	0.13333	0.12075
505	0.11990	0.10493	0.13275	0.12008
504	0.11917	0.10496	0.13196	0.11938
503	0.11824	0.10494	0.13101	0.11848
502	0.11718	0.10498	0.12991	0.11740
501	0.11600	0.10492	0.12863	0.11643
500	0.11487	0.10480	0.12747	0.11543
499	0.11376	0.10467	0.12627	0.11437
498	0.11269	0.10454	0.12514	0.11344
497	0.11170	0.10446	0.12408	0.11260
496	0.11074	0.10434	0.12321	0.11185
495	0.10997	0.10417	0.12241	0.11113
494	0.10932	0.10404	0.12170	0.11055
493	0.10877	0.10393	0.12113	0.11004
492	0.10827	0.10391	0.12060	0.10975
491	0.10785	0.10394	0.12020	0.10952
490	0.10757	0.10393	0.11989	0.10923
489	0.10745	0.10390	0.11969	0.10918
488	0.10735	0.10402	0.11955	0.10923
487	0.10737	0.10410	0.11959	0.10925
486	0.10745	0.10418	0.11962	0.10941
485	0.10757	0.10431	0.11966	0.10970
484	0.10781	0.10447	0.11986	0.11000
483	0.10822	0.10463	0.12024	0.11040
482	0.10860	0.10497	0.12060	0.11096
481	0.10913	0.10524	0.12119	0.11153
480	0.10973	0.10565	0.12190	0.11234

479	0.11052	0.10605	0.12273	0.11325
478	0.11146	0.10644	0.12377	0.11425
477	0.11243	0.10702	0.12485	0.11539
476	0.11355	0.10755	0.12615	0.11676
475	0.11487	0.10813	0.12760	0.11808
474	0.11622	0.10870	0.12925	0.11956
473	0.11766	0.10930	0.13094	0.12103
472	0.11916	0.10997	0.13278	0.12257
471	0.12069	0.11070	0.13452	0.12398
470	0.12204	0.11135	0.13620	0.12516
469	0.12334	0.11200	0.13787	0.12638
468	0.12455	0.11262	0.13938	0.12736
467	0.12555	0.11312	0.14069	0.12812
466	0.12636	0.11371	0.14172	0.12873
465	0.12694	0.11419	0.14253	0.12908
464	0.12736	0.11464	0.14308	0.12925
463	0.12752	0.11497	0.14333	0.12914
462	0.12756	0.11535	0.14344	0.12899
461	0.12750	0.11574	0.14323	0.12872
460	0.12729	0.11603	0.14284	0.12826
459	0.12696	0.11635	0.14229	0.12783
458	0.12660	0.11656	0.14165	0.12730
457	0.12618	0.11678	0.14096	0.12675
456	0.12562	0.11689	0.14019	0.12620
455	0.12503	0.11706	0.13941	0.12561
454	0.12433	0.11714	0.13849	0.12499
453	0.12357	0.11714	0.13745	0.12428
452	0.12268	0.11704	0.13641	0.12363
451	0.12191	0.11692	0.13544	0.12289
450	0.12116	0.11677	0.13439	0.12221
449	0.12034	0.11652	0.13344	0.12157
448	0.11955	0.11634	0.13253	0.12103

447	0.11891	0.11624	0.13173	0.12063
446	0.11841	0.11608	0.13110	0.12021
445	0.11811	0.11606	0.13051	0.11993
444	0.11774	0.11591	0.13011	0.11967
443	0.11743	0.11591	0.12977	0.11952
442	0.11730	0.11586	0.12949	0.11941
441	0.11708	0.11594	0.12940	0.11940
440	0.11715	0.11600	0.12940	0.11928
439	0.11721	0.11606	0.12945	0.11939
438	0.11734	0.11620	0.12956	0.11993
437	0.11747	0.11635	0.12982	0.12024
436	0.11770	0.11651	0.13012	0.12059
435	0.11792	0.11668	0.13045	0.12097
434	0.11824	0.11689	0.13076	0.12152
433	0.11864	0.11717	0.13119	0.12199
432	0.11903	0.11749	0.13160	0.12254
431	0.11949	0.11787	0.13218	0.12316
430	0.11986	0.11828	0.13276	0.12383
429	0.12039	0.11865	0.13342	0.12457
428	0.12095	0.11901	0.13406	0.12532
427	0.12140	0.11944	0.13478	0.12633
426	0.12206	0.11995	0.13564	0.12725
425	0.12278	0.12049	0.13662	0.12809
424	0.12349	0.12100	0.13763	0.12909
423	0.12428	0.12148	0.13874	0.13011
422	0.12507	0.12196	0.13992	0.13116
421	0.12586	0.12252	0.14118	0.13223
420	0.12677	0.12304	0.14265	0.13338
419	0.12756	0.12368	0.14390	0.13459
418	0.12856	0.12429	0.14536	0.13569
417	0.12958	0.12482	0.14672	0.13689
416	0.13056	0.12534	0.14814	0.13807

415	0.13156	0.12588	0.14946	0.13894
414	0.13272	0.12662	0.15081	0.13996
413	0.13354	0.12722	0.15200	0.14089
412	0.13448	0.12775	0.15292	0.14168
411	0.13543	0.12840	0.15383	0.14258
410	0.13641	0.12886	0.15467	0.14337
409	0.13717	0.12939	0.15546	0.14403
408	0.13796	0.12993	0.15611	0.14471
407	0.13870	0.13030	0.15677	0.14526
406	0.13940	0.13066	0.15739	0.14567
405	0.13991	0.13108	0.15784	0.14603
404	0.14044	0.13150	0.15830	0.14651
403	0.14090	0.13184	0.15869	0.14684
402	0.14129	0.13226	0.15896	0.14711
401	0.14162	0.13261	0.15917	0.14726
400	0.14182	0.13311	0.15920	0.14738

Figure 2b

Wavelength	SWNT-$(CF_2)_4$ DOC0.019	SWNT-$(CF_2)_4$ DOC0.023	SWNT-$(CF_2)_4$ DOC0.027	SWNT-$(CF_2)_4$ DOC0.029
1300	0.0283	0.0807	0.0439	0.0425
1299	0.0282	0.0804	0.0437	0.0423
1298	0.0281	0.0801	0.0435	0.0422
1297	0.0282	0.0802	0.0434	0.0422
1296	0.0282	0.0803	0.0433	0.0422
1295	0.0282	0.0803	0.0432	0.0421
1294	0.0282	0.0803	0.0430	0.0421
1293	0.0283	0.0806	0.0429	0.0420
1292	0.0284	0.0806	0.0429	0.0419
1291	0.0283	0.0804	0.0426	0.0417
1290	0.0283	0.0804	0.0426	0.0416
1289	0.0285	0.0806	0.0425	0.0415
1288	0.0287	0.0806	0.0424	0.0414
1287	0.0287	0.0805	0.0423	0.0411
1286	0.0286	0.0803	0.0421	0.0410
1285	0.0286	0.0801	0.0419	0.0407
1284	0.0288	0.0799	0.0418	0.0404
1283	0.0290	0.0799	0.0418	0.0404
1282	0.0289	0.0796	0.0415	0.0401
1281	0.0289	0.0795	0.0415	0.0397
1280	0.0290	0.0795	0.0414	0.0396
1279	0.0289	0.0790	0.0412	0.0393
1278	0.0290	0.0788	0.0410	0.0390
1277	0.0291	0.0790	0.0408	0.0388
1276	0.0292	0.0790	0.0407	0.0385
1275	0.0293	0.0787	0.0406	0.0383
1274	0.0293	0.0785	0.0405	0.0380
1273	0.0293	0.0786	0.0404	0.0379
1272	0.0295	0.0787	0.0403	0.0378

1271	0.0297	0.0791	0.0403	0.0377
1270	0.0299	0.0793	0.0402	0.0376
1269	0.0301	0.0795	0.0402	0.0375
1268	0.0302	0.0797	0.0403	0.0374
1267	0.0305	0.0802	0.0403	0.0375
1266	0.0308	0.0807	0.0403	0.0376
1265	0.0310	0.0811	0.0404	0.0376
1264	0.0313	0.0815	0.0406	0.0375
1263	0.0316	0.0821	0.0406	0.0377
1262	0.0320	0.0829	0.0407	0.0379
1261	0.0323	0.0834	0.0409	0.0379
1260	0.0328	0.0840	0.0411	0.0381
1259	0.0330	0.0848	0.0412	0.0383
1258	0.0333	0.0854	0.0414	0.0384
1257	0.0338	0.0860	0.0417	0.0386
1256	0.0342	0.0869	0.0418	0.0388
1255	0.0345	0.0875	0.0419	0.0390
1254	0.0348	0.0881	0.0423	0.0390
1253	0.0353	0.0890	0.0425	0.0394
1252	0.0357	0.0896	0.0427	0.0397
1251	0.0361	0.0902	0.0429	0.0398
1250	0.0365	0.0912	0.0431	0.0399
1249	0.0369	0.0917	0.0433	0.0401
1248	0.0374	0.0923	0.0435	0.0404
1247	0.0377	0.0932	0.0439	0.0406
1246	0.0382	0.0940	0.0442	0.0408
1245	0.0385	0.0947	0.0444	0.0410
1244	0.0387	0.0951	0.0446	0.0411
1243	0.0393	0.0958	0.0450	0.0414
1242	0.0397	0.0965	0.0454	0.0417
1241	0.0401	0.0972	0.0456	0.0419
1240	0.0405	0.0977	0.0459	0.0421

1239	0.0410	0.0986	0.0463	0.0425
1238	0.0413	0.0992	0.0465	0.0426
1237	0.0416	0.0996	0.0468	0.0428
1236	0.0419	0.0997	0.0469	0.0427
1235	0.0424	0.1002	0.0473	0.0430
1234	0.0428	0.1010	0.0476	0.0431
1233	0.0431	0.1013	0.0480	0.0432
1232	0.0434	0.1016	0.0482	0.0433
1231	0.0437	0.1020	0.0485	0.0434
1230	0.0440	0.1025	0.0489	0.0436
1229	0.0444	0.1028	0.0491	0.0437
1228	0.0446	0.1033	0.0493	0.0436
1227	0.0450	0.1034	0.0496	0.0437
1226	0.0453	0.1036	0.0499	0.0438
1225	0.0456	0.1041	0.0503	0.0439
1224	0.0459	0.1044	0.0506	0.0440
1223	0.0462	0.1046	0.0508	0.0440
1222	0.0464	0.1048	0.0511	0.0440
1221	0.0467	0.1051	0.0515	0.0441
1220	0.0469	0.1053	0.0518	0.0442
1219	0.0472	0.1055	0.0522	0.0442
1218	0.0475	0.1056	0.0524	0.0441
1217	0.0475	0.1056	0.0526	0.0442
1216	0.0479	0.1058	0.0530	0.0443
1215	0.0481	0.1060	0.0533	0.0444
1214	0.0482	0.1058	0.0536	0.0443
1213	0.0485	0.1059	0.0540	0.0445
1212	0.0486	0.1060	0.0541	0.0444
1211	0.0486	0.1058	0.0543	0.0443
1210	0.0487	0.1058	0.0546	0.0443
1209	0.0488	0.1057	0.0548	0.0445
1208	0.0490	0.1057	0.0552	0.0446

1207	0.0491	0.1055	0.0552	0.0445
1206	0.0492	0.1055	0.0555	0.0444
1205	0.0492	0.1053	0.0557	0.0443
1204	0.0492	0.1050	0.0558	0.0442
1203	0.0491	0.1049	0.0559	0.0441
1202	0.0492	0.1048	0.0561	0.0441
1201	0.0491	0.1045	0.0562	0.0441
1200	0.0491	0.1047	0.0566	0.0442
1199	0.0490	0.1043	0.0567	0.0441
1198	0.0489	0.1041	0.0567	0.0440
1197	0.0489	0.1041	0.0569	0.0439
1196	0.0486	0.1037	0.0571	0.0437
1195	0.0486	0.1036	0.0572	0.0437
1194	0.0485	0.1032	0.0573	0.0437
1193	0.0483	0.1032	0.0576	0.0436
1192	0.0482	0.1032	0.0577	0.0437
1191	0.0481	0.1030	0.0579	0.0436
1190	0.0478	0.1028	0.0580	0.0434
1189	0.0477	0.1028	0.0585	0.0435
1188	0.0476	0.1026	0.0587	0.0434
1187	0.0475	0.1027	0.0590	0.0435
1186	0.0473	0.1026	0.0593	0.0436
1185	0.0470	0.1023	0.0594	0.0435
1184	0.0469	0.1025	0.0597	0.0435
1183	0.0467	0.1025	0.0599	0.0435
1182	0.0463	0.1020	0.0601	0.0434
1181	0.0461	0.1021	0.0605	0.0435
1180	0.0456	0.1013	0.0604	0.0434
1179	0.0453	0.1008	0.0604	0.0433
1178	0.0449	0.1005	0.0605	0.0430
1177	0.0446	0.0998	0.0607	0.0428
1176	0.0441	0.0987	0.0605	0.0424

1175	0.0436	0.0979	0.0602	0.0420
1174	0.0430	0.0968	0.0601	0.0415
1173	0.0421	0.0954	0.0596	0.0408
1172	0.0414	0.0944	0.0593	0.0403
1171	0.0410	0.0933	0.0590	0.0400
1170	0.0402	0.0919	0.0584	0.0393
1169	0.0398	0.0911	0.0581	0.0389
1168	0.0393	0.0900	0.0577	0.0386
1167	0.0388	0.0894	0.0572	0.0382
1166	0.0383	0.0887	0.0568	0.0379
1165	0.0380	0.0882	0.0563	0.0377
1164	0.0377	0.0879	0.0560	0.0375
1163	0.0374	0.0872	0.0556	0.0372
1162	0.0372	0.0872	0.0553	0.0371
1161	0.0370	0.0872	0.0550	0.0370
1160	0.0368	0.0869	0.0546	0.0368
1159	0.0367	0.0871	0.0544	0.0367
1158	0.0367	0.0873	0.0542	0.0368
1157	0.0364	0.0870	0.0537	0.0366
1156	0.0365	0.0875	0.0536	0.0367
1155	0.0365	0.0875	0.0534	0.0366
1154	0.0366	0.0879	0.0533	0.0366
1153	0.0365	0.0878	0.0531	0.0364
1152	0.0364	0.0881	0.0529	0.0365
1151	0.0365	0.0883	0.0527	0.0365
1150	0.0363	0.0883	0.0525	0.0364
1149	0.0362	0.0886	0.0524	0.0364
1148	0.0363	0.0890	0.0523	0.0364
1147	0.0363	0.0890	0.0522	0.0364
1146	0.0365	0.0893	0.0523	0.0365
1145	0.0365	0.0895	0.0522	0.0365
1144	0.0364	0.0896	0.0520	0.0364

1143	0.0366	0.0900	0.0520	0.0365
1142	0.0367	0.0904	0.0522	0.0366
1141	0.0367	0.0904	0.0521	0.0367
1140	0.0368	0.0909	0.0521	0.0369
1139	0.0368	0.0909	0.0521	0.0368
1138	0.0368	0.0911	0.0521	0.0368
1137	0.0369	0.0914	0.0522	0.0369
1136	0.0369	0.0918	0.0522	0.0369
1135	0.0370	0.0921	0.0522	0.0371
1134	0.0371	0.0923	0.0523	0.0372
1133	0.0372	0.0926	0.0523	0.0372
1132	0.0372	0.0928	0.0523	0.0372
1131	0.0373	0.0931	0.0523	0.0373
1130	0.0374	0.0936	0.0525	0.0373
1129	0.0374	0.0938	0.0525	0.0373
1128	0.0375	0.0940	0.0526	0.0374
1127	0.0377	0.0947	0.0527	0.0376
1126	0.0377	0.0948	0.0528	0.0376
1125	0.0378	0.0951	0.0528	0.0376
1124	0.0378	0.0954	0.0529	0.0377
1123	0.0378	0.0956	0.0529	0.0376
1122	0.0379	0.0959	0.0529	0.0376
1121	0.0380	0.0964	0.0531	0.0379
1120	0.0382	0.0969	0.0533	0.0378
1119	0.0383	0.0974	0.0534	0.0379
1118	0.0384	0.0978	0.0535	0.0381
1117	0.0386	0.0985	0.0537	0.0382
1116	0.0387	0.0988	0.0537	0.0382
1115	0.0388	0.0992	0.0538	0.0382
1114	0.0390	0.0997	0.0541	0.0382
1113	0.0391	0.1002	0.0542	0.0384
1112	0.0395	0.1010	0.0545	0.0386

1111	0.0394	0.1014	0.0546	0.0387
1110	0.0397	0.1022	0.0548	0.0388
1109	0.0400	0.1029	0.0550	0.0389
1108	0.0402	0.1036	0.0553	0.0390
1107	0.0404	0.1044	0.0556	0.0391
1106	0.0407	0.1054	0.0559	0.0394
1105	0.0412	0.1063	0.0563	0.0397
1104	0.0414	0.1071	0.0565	0.0399
1103	0.0417	0.1082	0.0569	0.0400
1102	0.0420	0.1092	0.0572	0.0401
1101	0.0425	0.1102	0.0577	0.0404
1100	0.0427	0.1114	0.0581	0.0408
1099	0.0431	0.1129	0.0587	0.0412
1098	0.0438	0.1141	0.0591	0.0415
1097	0.0442	0.1155	0.0596	0.0420
1096	0.0447	0.1171	0.0601	0.0422
1095	0.0452	0.1182	0.0606	0.0425
1094	0.0456	0.1196	0.0612	0.0428
1093	0.0463	0.1215	0.0620	0.0434
1092	0.0468	0.1231	0.0626	0.0439
1091	0.0476	0.1250	0.0632	0.0444
1090	0.0482	0.1270	0.0639	0.0449
1089	0.0487	0.1286	0.0646	0.0452
1088	0.0496	0.1308	0.0655	0.0459
1087	0.0503	0.1331	0.0662	0.0465
1086	0.0509	0.1349	0.0669	0.0470
1085	0.0517	0.1372	0.0679	0.0475
1084	0.0524	0.1397	0.0688	0.0481
1083	0.0533	0.1421	0.0697	0.0489
1082	0.0540	0.1445	0.0706	0.0495
1081	0.0549	0.1472	0.0717	0.0503
1080	0.0558	0.1499	0.0727	0.0509

1079	0.0566	0.1527	0.0738	0.0518
1078	0.0575	0.1556	0.0750	0.0525
1077	0.0583	0.1583	0.0761	0.0533
1076	0.0592	0.1614	0.0772	0.0542
1075	0.0603	0.1649	0.0785	0.0552
1074	0.0611	0.1680	0.0797	0.0560
1073	0.0622	0.1714	0.0810	0.0570
1072	0.0631	0.1750	0.0823	0.0579
1071	0.0642	0.1785	0.0837	0.0590
1070	0.0651	0.1821	0.0850	0.0600
1069	0.0661	0.1859	0.0863	0.0610
1068	0.0673	0.1897	0.0879	0.0621
1067	0.0682	0.1937	0.0894	0.0633
1066	0.0693	0.1976	0.0909	0.0645
1065	0.0705	0.2021	0.0926	0.0656
1064	0.0716	0.2065	0.0941	0.0670
1063	0.0729	0.2110	0.0959	0.0683
1062	0.0741	0.2161	0.0977	0.0695
1061	0.0753	0.2206	0.0995	0.0709
1060	0.0767	0.2256	0.1014	0.0724
1059	0.0780	0.2305	0.1033	0.0738
1058	0.0794	0.2358	0.1054	0.0753
1057	0.0806	0.2410	0.1073	0.0767
1056	0.0819	0.2463	0.1094	0.0783
1055	0.0834	0.2518	0.1115	0.0799
1054	0.0849	0.2576	0.1137	0.0816
1053	0.0862	0.2635	0.1160	0.0832
1052	0.0876	0.2692	0.1184	0.0849
1051	0.0891	0.2750	0.1207	0.0866
1050	0.0907	0.2815	0.1232	0.0884
1049	0.0923	0.2879	0.1261	0.0904
1048	0.0938	0.2946	0.1287	0.0923

1047	0.0953	0.3013	0.1314	0.0945
1046	0.0969	0.3080	0.1342	0.0964
1045	0.0984	0.3149	0.1369	0.0984
1044	0.1001	0.3222	0.1400	0.1007
1043	0.1018	0.3298	0.1431	0.1031
1042	0.1033	0.3372	0.1463	0.1052
1041	0.1051	0.3456	0.1497	0.1078
1040	0.1068	0.3540	0.1529	0.1103
1039	0.1087	0.3628	0.1565	0.1130
1038	0.1105	0.3719	0.1601	0.1157
1037	0.1122	0.3813	0.1639	0.1185
1036	0.1143	0.3916	0.1681	0.1216
1035	0.1162	0.4024	0.1721	0.1248
1034	0.1183	0.4138	0.1765	0.1281
1033	0.1204	0.4256	0.1808	0.1314
1032	0.1225	0.4379	0.1854	0.1348
1031	0.1246	0.4508	0.1901	0.1382
1030	0.1269	0.4647	0.1949	0.1419
1029	0.1292	0.4794	0.1999	0.1457
1028	0.1315	0.4949	0.2052	0.1497
1027	0.1341	0.5112	0.2106	0.1538
1026	0.1366	0.5281	0.2161	0.1582
1025	0.1391	0.5459	0.2219	0.1625
1024	0.1417	0.5649	0.2280	0.1670
1023	0.1444	0.5846	0.2343	0.1719
1022	0.1471	0.6055	0.2410	0.1768
1021	0.1502	0.6272	0.2480	0.1824
1020	0.1534	0.6505	0.2556	0.1883
1019	0.1565	0.6741	0.2636	0.1943
1018	0.1599	0.6984	0.2719	0.2009
1017	0.1631	0.7230	0.2807	0.2077
1016	0.1665	0.7485	0.2901	0.2150

1015	0.1698	0.7740	0.2998	0.2230
1014	0.1731	0.7991	0.3103	0.2314
1013	0.1764	0.8240	0.3211	0.2402
1012	0.1797	0.8486	0.3326	0.2498
1011	0.1827	0.8720	0.3448	0.2601
1010	0.1858	0.8950	0.3580	0.2713
1009	0.1887	0.9158	0.3717	0.2835
1008	0.1913	0.9353	0.3861	0.2966
1007	0.1936	0.9524	0.4014	0.3107
1006	0.1957	0.9673	0.4176	0.3262
1005	0.1977	0.9798	0.4348	0.3427
1004	0.1991	0.9891	0.4530	0.3608
1003	0.2004	0.9958	0.4723	0.3806
1002	0.2015	0.9996	0.4929	0.4018
1001	0.2019	1.0000	0.5145	0.4249
1000	0.2020	0.9971	0.5378	0.4502
999	0.2017	0.9915	0.5619	0.4770
998	0.2012	0.9830	0.5880	0.5061
997	0.2003	0.9713	0.6153	0.5373
996	0.1991	0.9576	0.6442	0.5706
995	0.1977	0.9425	0.6743	0.6055
994	0.1961	0.9248	0.7055	0.6420
993	0.1941	0.9059	0.7378	0.6799
992	0.1921	0.8855	0.7713	0.7190
991	0.1898	0.8642	0.8047	0.7583
990	0.1874	0.8424	0.8379	0.7977
989	0.1851	0.8200	0.8705	0.8363
988	0.1825	0.7970	0.9017	0.8731
987	0.1799	0.7740	0.9298	0.9070
986	0.1772	0.7513	0.9547	0.9370
985	0.1745	0.7289	0.9748	0.9621
984	0.1719	0.7069	0.9894	0.9812

983	0.1692	0.6855	0.9981	0.9941
982	0.1663	0.6641	1.0000	1.0000
981	0.1636	0.6440	0.9948	0.9987
980	0.1608	0.6241	0.9827	0.9902
979	0.1578	0.6048	0.9644	0.9750
978	0.1550	0.5866	0.9401	0.9540
977	0.1521	0.5691	0.9109	0.9277
976	0.1493	0.5524	0.8782	0.8975
975	0.1464	0.5361	0.8423	0.8639
974	0.1436	0.5207	0.8042	0.8284
973	0.1410	0.5063	0.7662	0.7915
972	0.1381	0.4920	0.7275	0.7534
971	0.1355	0.4786	0.6893	0.7155
970	0.1330	0.4661	0.6520	0.6779
969	0.1305	0.4542	0.6162	0.6418
968	0.1281	0.4425	0.5818	0.6069
967	0.1260	0.4315	0.5495	0.5736
966	0.1240	0.4215	0.5192	0.5417
965	0.1220	0.4119	0.4912	0.5119
964	0.1201	0.4027	0.4650	0.4835
963	0.1186	0.3943	0.4411	0.4572
962	0.1170	0.3859	0.4188	0.4325
961	0.1155	0.3782	0.3985	0.4098
960	0.1144	0.3711	0.3798	0.3884
959	0.1133	0.3642	0.3627	0.3688
958	0.1123	0.3578	0.3471	0.3504
957	0.1113	0.3516	0.3327	0.3335
956	0.1106	0.3459	0.3195	0.3177
955	0.1100	0.3404	0.3075	0.3031
954	0.1094	0.3352	0.2963	0.2895
953	0.1092	0.3304	0.2864	0.2770
952	0.1088	0.3254	0.2771	0.2652

951	0.1086	0.3213	0.2687	0.2546
950	0.1086	0.3171	0.2610	0.2448
949	0.1089	0.3136	0.2544	0.2359
948	0.1092	0.3104	0.2483	0.2274
947	0.1098	0.3077	0.2431	0.2201
946	0.1105	0.3047	0.2382	0.2133
945	0.1112	0.3022	0.2340	0.2071
944	0.1123	0.3000	0.2305	0.2016
943	0.1135	0.2983	0.2277	0.1968
942	0.1149	0.2968	0.2254	0.1926
941	0.1163	0.2951	0.2235	0.1888
940	0.1179	0.2937	0.2222	0.1854
939	0.1197	0.2927	0.2214	0.1827
938	0.1216	0.2917	0.2212	0.1804
937	0.1235	0.2909	0.2215	0.1787
936	0.1258	0.2902	0.2223	0.1773
935	0.1284	0.2895	0.2237	0.1764
934	0.1309	0.2890	0.2260	0.1760
933	0.1335	0.2885	0.2287	0.1761
932	0.1364	0.2882	0.2323	0.1766
931	0.1397	0.2884	0.2364	0.1777
930	0.1429	0.2887	0.2411	0.1790
929	0.1464	0.2889	0.2464	0.1809
928	0.1500	0.2891	0.2519	0.1829
927	0.1539	0.2897	0.2576	0.1851
926	0.1580	0.2906	0.2632	0.1873
925	0.1624	0.2916	0.2686	0.1895
924	0.1670	0.2928	0.2732	0.1915
923	0.1717	0.2945	0.2769	0.1930
922	0.1768	0.2961	0.2797	0.1940
921	0.1823	0.2984	0.2814	0.1946
920	0.1882	0.3009	0.2817	0.1947

919	0.1943	0.3036	0.2805	0.1941
918	0.2008	0.3066	0.2783	0.1928
917	0.2080	0.3106	0.2751	0.1911
916	0.2155	0.3148	0.2708	0.1888
915	0.2232	0.3194	0.2658	0.1857
914	0.2314	0.3246	0.2606	0.1826
913	0.2403	0.3301	0.2550	0.1793
912	0.2497	0.3365	0.2493	0.1758
911	0.2597	0.3432	0.2437	0.1721
910	0.2703	0.3507	0.2380	0.1684
909	0.2820	0.3595	0.2331	0.1650
908	0.2944	0.3687	0.2284	0.1618
907	0.3077	0.3786	0.2240	0.1588
906	0.3221	0.3894	0.2202	0.1557
905	0.3378	0.4017	0.2170	0.1531
904	0.3548	0.4149	0.2142	0.1506
903	0.3735	0.4292	0.2119	0.1485
902	0.3939	0.4447	0.2100	0.1467
901	0.4156	0.4608	0.2085	0.1447
900	0.4391	0.4784	0.2078	0.1433
899	0.4644	0.4977	0.2073	0.1420
898	0.4920	0.5178	0.2074	0.1410
897	0.5210	0.5396	0.2080	0.1404
896	0.5521	0.5631	0.2091	0.1403
895	0.5853	0.5866	0.2108	0.1400
894	0.6195	0.6112	0.2125	0.1401
893	0.6554	0.6381	0.2151	0.1408
892	0.6922	0.6653	0.2178	0.1412
891	0.7294	0.6922	0.2208	0.1417
890	0.7669	0.7199	0.2243	0.1428
889	0.8046	0.7479	0.2281	0.1443
888	0.8417	0.7763	0.2323	0.1460

887	0.8766	0.8032	0.2366	0.1473
886	0.9097	0.8296	0.2406	0.1492
885	0.9386	0.8547	0.2447	0.1510
884	0.9631	0.8769	0.2489	0.1527
883	0.9825	0.8957	0.2527	0.1543
882	0.9947	0.9119	0.2557	0.1558
881	1.0000	0.9245	0.2583	0.1573
880	0.9978	0.9322	0.2603	0.1584
879	0.9871	0.9359	0.2616	0.1592
878	0.9698	0.9352	0.2621	0.1594
877	0.9459	0.9295	0.2615	0.1596
876	0.9156	0.9194	0.2605	0.1597
875	0.8807	0.9057	0.2585	0.1593
874	0.8427	0.8881	0.2558	0.1581
873	0.8026	0.8671	0.2526	0.1572
872	0.7613	0.8437	0.2490	0.1561
871	0.7197	0.8184	0.2455	0.1550
870	0.6787	0.7916	0.2417	0.1537
869	0.6392	0.7637	0.2374	0.1524
868	0.6010	0.7359	0.2332	0.1514
867	0.5652	0.7084	0.2297	0.1506
866	0.5315	0.6806	0.2260	0.1500
865	0.5004	0.6548	0.2230	0.1500
864	0.4715	0.6298	0.2202	0.1499
863	0.4445	0.6053	0.2175	0.1496
862	0.4200	0.5824	0.2154	0.1503
861	0.3973	0.5608	0.2132	0.1507
860	0.3765	0.5397	0.2115	0.1511
859	0.3576	0.5210	0.2101	0.1521
858	0.3403	0.5031	0.2087	0.1531
857	0.3246	0.4864	0.2077	0.1543
856	0.3100	0.4710	0.2065	0.1552

855	0.2971	0.4569	0.2056	0.1562
854	0.2847	0.4433	0.2048	0.1572
853	0.2736	0.4305	0.2040	0.1580
852	0.2636	0.4197	0.2032	0.1586
851	0.2543	0.4088	0.2021	0.1594
850	0.2511	0.4054	0.2023	0.1586
849	0.2426	0.3950	0.2012	0.1587
848	0.2351	0.3857	0.1999	0.1586
847	0.2280	0.3774	0.1985	0.1585
846	0.2219	0.3702	0.1974	0.1586
845	0.2161	0.3623	0.1957	0.1581
844	0.2107	0.3545	0.1940	0.1571
843	0.2051	0.3475	0.1921	0.1557
842	0.2011	0.3420	0.1904	0.1546
841	0.1975	0.3364	0.1884	0.1534
840	0.1935	0.3308	0.1863	0.1522
839	0.1901	0.3253	0.1844	0.1506
838	0.1871	0.3204	0.1824	0.1491
837	0.1846	0.3160	0.1804	0.1476
836	0.1819	0.3120	0.1784	0.1459
835	0.1795	0.3075	0.1767	0.1446
834	0.1774	0.3039	0.1750	0.1434
833	0.1753	0.2999	0.1729	0.1415
832	0.1735	0.2970	0.1713	0.1405
831	0.1718	0.2930	0.1699	0.1389
830	0.1700	0.2896	0.1683	0.1376
829	0.1683	0.2863	0.1667	0.1360
828	0.1666	0.2832	0.1653	0.1347
827	0.1652	0.2803	0.1639	0.1333
826	0.1633	0.2771	0.1623	0.1321
825	0.1616	0.2745	0.1608	0.1306
824	0.1598	0.2714	0.1595	0.1294

823	0.1580	0.2682	0.1581	0.1283
822	0.1560	0.2657	0.1569	0.1269
821	0.1541	0.2628	0.1554	0.1253
820	0.1527	0.2604	0.1542	0.1242
819	0.1504	0.2573	0.1526	0.1227
818	0.1483	0.2542	0.1512	0.1212
817	0.1465	0.2517	0.1499	0.1199
816	0.1443	0.2490	0.1486	0.1186
815	0.1427	0.2470	0.1474	0.1173
814	0.1406	0.2441	0.1462	0.1162
813	0.1390	0.2417	0.1451	0.1149
812	0.1373	0.2391	0.1439	0.1136
811	0.1354	0.2362	0.1427	0.1122
810	0.1339	0.2341	0.1416	0.1111
809	0.1324	0.2319	0.1407	0.1098
808	0.1307	0.2295	0.1396	0.1085
807	0.1293	0.2274	0.1389	0.1076
806	0.1281	0.2251	0.1377	0.1064
805	0.1271	0.2234	0.1367	0.1054
804	0.1260	0.2215	0.1357	0.1041
803	0.1250	0.2198	0.1348	0.1030
802	0.1244	0.2181	0.1337	0.1017
801	0.1236	0.2163	0.1326	0.1006
800	0.1232	0.2153	0.1315	0.0996
799	0.1229	0.2140	0.1304	0.0986
798	0.1227	0.2128	0.1294	0.0977
797	0.1227	0.2120	0.1283	0.0966
796	0.1229	0.2113	0.1273	0.0955
795	0.1230	0.2103	0.1261	0.0945
794	0.1235	0.2099	0.1251	0.0934
793	0.1243	0.2098	0.1241	0.0924
792	0.1253	0.2097	0.1234	0.0916

791	0.1265	0.2095	0.1226	0.0908
790	0.1278	0.2097	0.1218	0.0901
789	0.1291	0.2099	0.1210	0.0891
788	0.1310	0.2106	0.1206	0.0885
787	0.1334	0.2117	0.1200	0.0881
786	0.1356	0.2123	0.1195	0.0875
785	0.1379	0.2129	0.1191	0.0869
784	0.1406	0.2142	0.1188	0.0867
783	0.1437	0.2153	0.1187	0.0865
782	0.1470	0.2170	0.1184	0.0863
781	0.1501	0.2186	0.1181	0.0859
780	0.1535	0.2205	0.1183	0.0858
779	0.1566	0.2222	0.1181	0.0855
778	0.1597	0.2237	0.1180	0.0853
777	0.1631	0.2259	0.1181	0.0851
776	0.1661	0.2275	0.1180	0.0851
775	0.1687	0.2291	0.1179	0.0846
774	0.1712	0.2305	0.1177	0.0843
773	0.1737	0.2324	0.1175	0.0839
772	0.1757	0.2337	0.1173	0.0839
771	0.1772	0.2345	0.1170	0.0835
770	0.1782	0.2353	0.1165	0.0830
769	0.1791	0.2362	0.1162	0.0827
768	0.1795	0.2370	0.1158	0.0823
767	0.1795	0.2373	0.1154	0.0819
766	0.1794	0.2371	0.1149	0.0815
765	0.1790	0.2372	0.1145	0.0813
764	0.1782	0.2370	0.1140	0.0808
763	0.1771	0.2364	0.1133	0.0803
762	0.1759	0.2352	0.1127	0.0797
761	0.1750	0.2344	0.1122	0.0796
760	0.1736	0.2334	0.1115	0.0791

759	0.1724	0.2325	0.1110	0.0786
758	0.1710	0.2311	0.1103	0.0780
757	0.1696	0.2299	0.1098	0.0778
756	0.1683	0.2289	0.1093	0.0776
755	0.1671	0.2276	0.1087	0.0771
754	0.1657	0.2265	0.1083	0.0768
753	0.1645	0.2253	0.1079	0.0765
752	0.1632	0.2239	0.1073	0.0761
751	0.1619	0.2231	0.1070	0.0760
750	0.1604	0.2217	0.1065	0.0756
749	0.1589	0.2205	0.1060	0.0752
748	0.1573	0.2190	0.1054	0.0749
747	0.1560	0.2176	0.1052	0.0747
746	0.1545	0.2161	0.1048	0.0744
745	0.1528	0.2151	0.1044	0.0743
744	0.1512	0.2138	0.1042	0.0741
743	0.1497	0.2122	0.1038	0.0737
742	0.1481	0.2108	0.1037	0.0736
741	0.1461	0.2094	0.1032	0.0733
740	0.1445	0.2081	0.1031	0.0733
739	0.1429	0.2065	0.1028	0.0731
738	0.1411	0.2051	0.1027	0.0728
737	0.1396	0.2037	0.1025	0.0729
736	0.1380	0.2025	0.1024	0.0728
735	0.1361	0.2009	0.1022	0.0726
734	0.1345	0.1995	0.1023	0.0725
733	0.1329	0.1979	0.1023	0.0725
732	0.1311	0.1965	0.1021	0.0724
731	0.1296	0.1948	0.1022	0.0724
730	0.1283	0.1938	0.1024	0.0725
729	0.1265	0.1924	0.1025	0.0726
728	0.1249	0.1909	0.1026	0.0725

727	0.1234	0.1900	0.1029	0.0726
726	0.1219	0.1886	0.1031	0.0727
725	0.1205	0.1875	0.1034	0.0728
724	0.1191	0.1863	0.1037	0.0730
723	0.1178	0.1853	0.1041	0.0731
722	0.1165	0.1839	0.1044	0.0732
721	0.1153	0.1829	0.1049	0.0733
720	0.1144	0.1822	0.1056	0.0738
719	0.1135	0.1815	0.1062	0.0739
718	0.1127	0.1809	0.1071	0.0744
717	0.1120	0.1805	0.1080	0.0748
716	0.1113	0.1799	0.1087	0.0752
715	0.1108	0.1797	0.1099	0.0756
714	0.1102	0.1794	0.1108	0.0761
713	0.1098	0.1792	0.1120	0.0767
712	0.1093	0.1791	0.1131	0.0772
711	0.1089	0.1790	0.1144	0.0778
710	0.1088	0.1792	0.1160	0.0785
709	0.1084	0.1790	0.1174	0.0792
708	0.1082	0.1791	0.1189	0.0800
707	0.1079	0.1794	0.1205	0.0809
706	0.1076	0.1796	0.1219	0.0816
705	0.1072	0.1794	0.1235	0.0823
704	0.1068	0.1796	0.1250	0.0831
703	0.1066	0.1798	0.1266	0.0839
702	0.1062	0.1799	0.1280	0.0846
701	0.1059	0.1799	0.1295	0.0855
700	0.1057	0.1805	0.1309	0.0864
699	0.1055	0.1805	0.1321	0.0872
698	0.1052	0.1800	0.1330	0.0877
697	0.1049	0.1800	0.1337	0.0883
696	0.1046	0.1798	0.1341	0.0888

695	0.1044	0.1795	0.1341	0.0890
694	0.1041	0.1791	0.1339	0.0889
693	0.1038	0.1789	0.1332	0.0889
692	0.1036	0.1786	0.1322	0.0886
691	0.1032	0.1778	0.1306	0.0879
690	0.1031	0.1777	0.1292	0.0873
689	0.1029	0.1775	0.1274	0.0865
688	0.1027	0.1774	0.1256	0.0857
687	0.1025	0.1772	0.1237	0.0849
686	0.1025	0.1774	0.1220	0.0841
685	0.1023	0.1774	0.1203	0.0832
684	0.1022	0.1770	0.1187	0.0824
683	0.1021	0.1767	0.1173	0.0817
682	0.1021	0.1769	0.1161	0.0809
681	0.1021	0.1769	0.1152	0.0805
680	0.1020	0.1767	0.1142	0.0800
679	0.1020	0.1765	0.1135	0.0795
678	0.1020	0.1767	0.1129	0.0792
677	0.1022	0.1766	0.1125	0.0790
676	0.1023	0.1765	0.1120	0.0789
675	0.1023	0.1764	0.1118	0.0790
674	0.1023	0.1764	0.1116	0.0789
673	0.1024	0.1763	0.1114	0.0789
672	0.1026	0.1764	0.1113	0.0789
671	0.1028	0.1763	0.1112	0.0790
670	0.1029	0.1761	0.1113	0.0791
669	0.1030	0.1759	0.1112	0.0794
668	0.1034	0.1761	0.1113	0.0796
667	0.1035	0.1760	0.1113	0.0799
666	0.1037	0.1757	0.1114	0.0800
665	0.1039	0.1754	0.1115	0.0803
664	0.1042	0.1755	0.1116	0.0807

663	0.1045	0.1755	0.1119	0.0811
662	0.1048	0.1755	0.1122	0.0815
661	0.1051	0.1755	0.1124	0.0819
660	0.1054	0.1752	0.1127	0.0823
659	0.1056	0.1753	0.1131	0.0827
658	0.1060	0.1753	0.1135	0.0833
657	0.1064	0.1753	0.1140	0.0839
656	0.1068	0.1753	0.1145	0.0845
655	0.1072	0.1757	0.1152	0.0853
654	0.1075	0.1757	0.1158	0.0860
653	0.1077	0.1754	0.1163	0.0867
652	0.1081	0.1753	0.1170	0.0874
651	0.1085	0.1758	0.1176	0.0883
650	0.1088	0.1758	0.1184	0.0893
649	0.1091	0.1757	0.1191	0.0901
648	0.1095	0.1760	0.1199	0.0910
647	0.1099	0.1760	0.1205	0.0918
646	0.1104	0.1761	0.1213	0.0926
645	0.1108	0.1762	0.1217	0.0933
644	0.1112	0.1764	0.1221	0.0938
643	0.1118	0.1768	0.1226	0.0943
642	0.1123	0.1771	0.1228	0.0945
641	0.1128	0.1773	0.1228	0.0946
640	0.1136	0.1776	0.1228	0.0946
639	0.1145	0.1779	0.1227	0.0945
638	0.1151	0.1782	0.1225	0.0941
637	0.1160	0.1790	0.1225	0.0939
636	0.1170	0.1795	0.1222	0.0935
635	0.1181	0.1802	0.1221	0.0931
634	0.1193	0.1811	0.1222	0.0929
633	0.1206	0.1820	0.1224	0.0929
632	0.1221	0.1831	0.1226	0.0929

631	0.1236	0.1841	0.1230	0.0930
630	0.1252	0.1855	0.1234	0.0933
629	0.1271	0.1867	0.1239	0.0936
628	0.1289	0.1883	0.1246	0.0939
627	0.1310	0.1901	0.1254	0.0947
626	0.1332	0.1919	0.1263	0.0954
625	0.1353	0.1936	0.1272	0.0960
624	0.1380	0.1956	0.1281	0.0968
623	0.1406	0.1978	0.1291	0.0975
622	0.1433	0.2001	0.1302	0.0984
621	0.1463	0.2028	0.1313	0.0994
620	0.1496	0.2056	0.1327	0.1005
619	0.1531	0.2088	0.1341	0.1017
618	0.1566	0.2119	0.1356	0.1027
617	0.1603	0.2149	0.1371	0.1040
616	0.1644	0.2187	0.1387	0.1054
615	0.1687	0.2229	0.1405	0.1068
614	0.1734	0.2269	0.1424	0.1083
613	0.1783	0.2314	0.1445	0.1100
612	0.1835	0.2362	0.1465	0.1117
611	0.1892	0.2414	0.1488	0.1135
610	0.1952	0.2472	0.1513	0.1155
609	0.2016	0.2531	0.1538	0.1175
608	0.2085	0.2598	0.1567	0.1198
607	0.2157	0.2667	0.1596	0.1221
606	0.2236	0.2740	0.1627	0.1245
605	0.2318	0.2817	0.1660	0.1270
604	0.2408	0.2904	0.1696	0.1299
603	0.2505	0.2994	0.1734	0.1330
602	0.2609	0.3094	0.1776	0.1364
601	0.2724	0.3199	0.1823	0.1399
600	0.2847	0.3311	0.1871	0.1436

599	0.2980	0.3433	0.1924	0.1477
598	0.3125	0.3564	0.1982	0.1523
597	0.3278	0.3698	0.2043	0.1573
596	0.3439	0.3841	0.2110	0.1626
595	0.3610	0.3992	0.2181	0.1685
594	0.3787	0.4145	0.2257	0.1747
593	0.3965	0.4301	0.2338	0.1814
592	0.4144	0.4454	0.2422	0.1886
591	0.4318	0.4604	0.2512	0.1963
590	0.4481	0.4743	0.2605	0.2044
589	0.4625	0.4870	0.2700	0.2128
588	0.4743	0.4979	0.2796	0.2217
587	0.4830	0.5067	0.2894	0.2309
586	0.4879	0.5126	0.2989	0.2402
585	0.4888	0.5159	0.3082	0.2495
584	0.4857	0.5165	0.3173	0.2592
583	0.4790	0.5142	0.3262	0.2691
582	0.4692	0.5094	0.3352	0.2791
581	0.4572	0.5027	0.3444	0.2898
580	0.4436	0.4941	0.3536	0.3009
579	0.4289	0.4846	0.3634	0.3128
578	0.4137	0.4742	0.3736	0.3253
577	0.3986	0.4633	0.3840	0.3384
576	0.3839	0.4525	0.3942	0.3518
575	0.3700	0.4418	0.4040	0.3649
574	0.3565	0.4309	0.4126	0.3770
573	0.3438	0.4206	0.4194	0.3876
572	0.3318	0.4106	0.4239	0.3957
571	0.3202	0.4005	0.4253	0.4007
570	0.3094	0.3912	0.4235	0.4023
569	0.2992	0.3822	0.4183	0.4000
568	0.2894	0.3729	0.4097	0.3936

567	0.2799	0.3641	0.3981	0.3835
566	0.2710	0.3559	0.3841	0.3702
565	0.2625	0.3478	0.3683	0.3544
564	0.2547	0.3401	0.3517	0.3371
563	0.2474	0.3327	0.3348	0.3193
562	0.2404	0.3259	0.3180	0.3011
561	0.2339	0.3195	0.3017	0.2832
560	0.2280	0.3136	0.2865	0.2665
559	0.2226	0.3082	0.2724	0.2507
558	0.2176	0.3033	0.2595	0.2363
557	0.2132	0.2992	0.2480	0.2234
556	0.2091	0.2950	0.2376	0.2116
555	0.2052	0.2915	0.2283	0.2009
554	0.2019	0.2886	0.2203	0.1917
553	0.1992	0.2859	0.2131	0.1836
552	0.1968	0.2838	0.2069	0.1763
551	0.1947	0.2821	0.2013	0.1700
550	0.1929	0.2807	0.1965	0.1642
549	0.1916	0.2798	0.1923	0.1593
548	0.1907	0.2793	0.1886	0.1550
547	0.1898	0.2789	0.1853	0.1510
546	0.1895	0.2789	0.1826	0.1475
545	0.1894	0.2795	0.1804	0.1446
544	0.1894	0.2800	0.1783	0.1419
543	0.1896	0.2809	0.1766	0.1395
542	0.1900	0.2821	0.1753	0.1376
541	0.1906	0.2838	0.1742	0.1358
540	0.1912	0.2854	0.1732	0.1342
539	0.1917	0.2871	0.1724	0.1329
538	0.1922	0.2890	0.1718	0.1318
537	0.1929	0.2914	0.1714	0.1311
536	0.1934	0.2940	0.1712	0.1306

535	0.1940	0.2966	0.1712	0.1304
534	0.1943	0.2994	0.1715	0.1305
533	0.1943	0.3023	0.1719	0.1307
532	0.1942	0.3054	0.1725	0.1312
531	0.1941	0.3092	0.1735	0.1323
530	0.1939	0.3131	0.1748	0.1334
529	0.1936	0.3172	0.1762	0.1347
528	0.1931	0.3217	0.1778	0.1362
527	0.1926	0.3270	0.1795	0.1377
526	0.1920	0.3325	0.1812	0.1394
525	0.1915	0.3390	0.1831	0.1411
524	0.1908	0.3462	0.1848	0.1426
523	0.1902	0.3540	0.1864	0.1439
522	0.1896	0.3631	0.1878	0.1451
521	0.1890	0.3729	0.1893	0.1458
520	0.1884	0.3833	0.1903	0.1463
519	0.1879	0.3951	0.1912	0.1464
518	0.1873	0.4078	0.1920	0.1463
517	0.1868	0.4215	0.1927	0.1459
516	0.1863	0.4356	0.1932	0.1451
515	0.1858	0.4499	0.1936	0.1442
514	0.1853	0.4646	0.1939	0.1432
513	0.1847	0.4781	0.1940	0.1420
512	0.1840	0.4904	0.1939	0.1405
511	0.1832	0.5005	0.1934	0.1392
510	0.1821	0.5076	0.1926	0.1374
509	0.1809	0.5113	0.1913	0.1358
508	0.1794	0.5109	0.1895	0.1339
507	0.1776	0.5068	0.1873	0.1318
506	0.1758	0.4995	0.1848	0.1298
505	0.1736	0.4891	0.1820	0.1276
504	0.1714	0.4761	0.1789	0.1254

503	0.1693	0.4618	0.1757	0.1232
502	0.1671	0.4464	0.1724	0.1211
501	0.1650	0.4308	0.1692	0.1192
500	0.1628	0.4154	0.1660	0.1172
499	0.1609	0.4007	0.1631	0.1153
498	0.1591	0.3871	0.1604	0.1137
497	0.1573	0.3743	0.1579	0.1120
496	0.1558	0.3629	0.1556	0.1106
495	0.1542	0.3526	0.1536	0.1094
494	0.1527	0.3432	0.1517	0.1083
493	0.1516	0.3349	0.1500	0.1074
492	0.1503	0.3277	0.1486	0.1064
491	0.1493	0.3214	0.1472	0.1057
490	0.1483	0.3158	0.1459	0.1048
489	0.1472	0.3106	0.1448	0.1039
488	0.1464	0.3062	0.1437	0.1033
487	0.1456	0.3026	0.1428	0.1026
486	0.1449	0.2996	0.1420	0.1020
485	0.1442	0.2972	0.1414	0.1015
484	0.1439	0.2953	0.1408	0.1010
483	0.1436	0.2945	0.1403	0.1006
482	0.1434	0.2939	0.1400	0.1002
481	0.1433	0.2940	0.1398	0.1000
480	0.1435	0.2947	0.1399	0.0998
479	0.1438	0.2959	0.1399	0.0997
478	0.1442	0.2978	0.1401	0.0997
477	0.1448	0.3004	0.1405	0.0996
476	0.1455	0.3031	0.1411	0.0997
475	0.1463	0.3065	0.1418	0.0999
474	0.1470	0.3097	0.1424	0.1000
473	0.1480	0.3131	0.1433	0.1003
472	0.1490	0.3162	0.1441	0.1006

471	0.1499	0.3190	0.1448	0.1008
470	0.1507	0.3212	0.1455	0.1010
469	0.1513	0.3230	0.1460	0.1012
468	0.1517	0.3242	0.1465	0.1014
467	0.1520	0.3245	0.1467	0.1015
466	0.1523	0.3243	0.1469	0.1016
465	0.1522	0.3232	0.1469	0.1016
464	0.1522	0.3221	0.1469	0.1016
463	0.1517	0.3202	0.1467	0.1016
462	0.1513	0.3179	0.1464	0.1015
461	0.1506	0.3152	0.1459	0.1013
460	0.1498	0.3123	0.1455	0.1012
459	0.1490	0.3091	0.1448	0.1009
458	0.1481	0.3059	0.1442	0.1007
457	0.1470	0.3026	0.1435	0.1004
456	0.1460	0.2996	0.1429	0.1002
455	0.1447	0.2962	0.1421	0.0996
454	0.1436	0.2935	0.1414	0.0994
453	0.1424	0.2906	0.1406	0.0991
452	0.1411	0.2876	0.1397	0.0985
451	0.1400	0.2851	0.1391	0.0983
450	0.1389	0.2830	0.1384	0.0981
449	0.1379	0.2810	0.1377	0.0977
448	0.1370	0.2793	0.1374	0.0974
447	0.1362	0.2779	0.1369	0.0973
446	0.1354	0.2765	0.1365	0.0971
445	0.1346	0.2753	0.1361	0.0969
444	0.1339	0.2744	0.1358	0.0967
443	0.1333	0.2733	0.1357	0.0965
442	0.1327	0.2725	0.1353	0.0963
441	0.1323	0.2718	0.1352	0.0961
440	0.1319	0.2714	0.1350	0.0960

439	0.1315	0.2706	0.1347	0.0958
438	0.1313	0.2704	0.1348	0.0958
437	0.1310	0.2705	0.1348	0.0956
436	0.1308	0.2704	0.1347	0.0956
435	0.1308	0.2703	0.1348	0.0956
434	0.1306	0.2700	0.1348	0.0955
433	0.1304	0.2696	0.1348	0.0953
432	0.1303	0.2697	0.1350	0.0953
431	0.1302	0.2697	0.1352	0.0955
430	0.1303	0.2697	0.1353	0.0955
429	0.1304	0.2702	0.1355	0.0955
428	0.1304	0.2703	0.1356	0.0955
427	0.1305	0.2706	0.1359	0.0956
426	0.1307	0.2711	0.1361	0.0958
425	0.1308	0.2716	0.1366	0.0958
424	0.1311	0.2722	0.1370	0.0958
423	0.1315	0.2729	0.1374	0.0961
422	0.1319	0.2737	0.1380	0.0964
421	0.1325	0.2746	0.1387	0.0967
420	0.1326	0.2753	0.1391	0.0968
419	0.1334	0.2762	0.1398	0.0971
418	0.1342	0.2775	0.1407	0.0977
417	0.1348	0.2785	0.1415	0.0980
416	0.1358	0.2800	0.1426	0.0985
415	0.1366	0.2812	0.1433	0.0990
414	0.1373	0.2826	0.1442	0.0994
413	0.1382	0.2837	0.1453	0.0999
412	0.1391	0.2852	0.1463	0.1005
411	0.1398	0.2862	0.1472	0.1008
410	0.1408	0.2878	0.1481	0.1013
409	0.1414	0.2892	0.1489	0.1018
408	0.1422	0.2905	0.1498	0.1023

407	0.1428	0.2915	0.1504	0.1025
406	0.1432	0.2926	0.1509	0.1026
405	0.1437	0.2937	0.1515	0.1028
404	0.1443	0.2952	0.1521	0.1032
403	0.1447	0.2964	0.1525	0.1034
402	0.1450	0.2975	0.1530	0.1035
401	0.1452	0.2985	0.1534	0.1036
400	0.1454	0.3000	0.1539	0.1038

Wavelength	SWNT-$(CF_2)_4$ DOC0.035	SWNT-$(CF_2)_4$ DOC0.040	SWNT-$(CF_2)_4$ DOC0.045	SWNT-$(CF_2)_4$ DOC0.048
1300	0.0505	0.0704	0.1650	0.2393
1299	0.0503	0.0704	0.1650	0.2386
1298	0.0499	0.0705	0.1656	0.2378
1297	0.0497	0.0708	0.1660	0.2396
1296	0.0493	0.0710	0.1665	0.2386
1295	0.0490	0.0712	0.1667	0.2388
1294	0.0487	0.0715	0.1668	0.2389
1293	0.0484	0.0717	0.1671	0.2386
1292	0.0482	0.0721	0.1676	0.2392
1291	0.0478	0.0721	0.1671	0.2373
1290	0.0476	0.0724	0.1673	0.2373
1289	0.0473	0.0726	0.1680	0.2382
1288	0.0471	0.0728	0.1680	0.2386
1287	0.0469	0.0729	0.1673	0.2377
1286	0.0466	0.0729	0.1672	0.2366
1285	0.0463	0.0730	0.1664	0.2350
1284	0.0460	0.0731	0.1659	0.2361
1283	0.0459	0.0732	0.1663	0.2358
1282	0.0456	0.0732	0.1658	0.2344
1281	0.0453	0.0733	0.1655	0.2346

1280	0.0451	0.0733	0.1655	0.2346
1279	0.0448	0.0733	0.1650	0.2332
1278	0.0444	0.0732	0.1643	0.2320
1277	0.0442	0.0733	0.1649	0.2324
1276	0.0439	0.0736	0.1646	0.2319
1275	0.0435	0.0735	0.1642	0.2319
1274	0.0432	0.0736	0.1640	0.2306
1273	0.0429	0.0736	0.1644	0.2311
1272	0.0427	0.0737	0.1643	0.2319
1271	0.0425	0.0740	0.1649	0.2331
1270	0.0422	0.0742	0.1650	0.2345
1269	0.0420	0.0744	0.1658	0.2369
1268	0.0418	0.0746	0.1664	0.2390
1267	0.0416	0.0748	0.1667	0.2405
1266	0.0415	0.0751	0.1679	0.2431
1265	0.0414	0.0754	0.1692	0.2456
1264	0.0413	0.0758	0.1700	0.2480
1263	0.0412	0.0760	0.1709	0.2502
1262	0.0412	0.0765	0.1723	0.2543
1261	0.0411	0.0767	0.1729	0.2579
1260	0.0410	0.0771	0.1746	0.2619
1259	0.0410	0.0773	0.1756	0.2652
1258	0.0410	0.0777	0.1762	0.2669
1257	0.0410	0.0782	0.1777	0.2715
1256	0.0410	0.0786	0.1793	0.2759
1255	0.0409	0.0789	0.1800	0.2790
1254	0.0410	0.0792	0.1807	0.2834
1253	0.0411	0.0796	0.1827	0.2879
1252	0.0411	0.0801	0.1841	0.2923
1251	0.0411	0.0803	0.1850	0.2956
1250	0.0411	0.0804	0.1866	0.2994
1249	0.0412	0.0806	0.1879	0.3020

1248	0.0413	0.0811	0.1889	0.3056
1247	0.0413	0.0814	0.1904	0.3094
1246	0.0414	0.0817	0.1923	0.3129
1245	0.0414	0.0821	0.1938	0.3160
1244	0.0415	0.0824	0.1946	0.3186
1243	0.0416	0.0828	0.1964	0.3230
1242	0.0417	0.0830	0.1983	0.3257
1241	0.0418	0.0832	0.1999	0.3287
1240	0.0418	0.0835	0.2013	0.3315
1239	0.0420	0.0839	0.2038	0.3350
1238	0.0420	0.0841	0.2049	0.3365
1237	0.0420	0.0843	0.2061	0.3394
1236	0.0420	0.0844	0.2066	0.3386
1235	0.0422	0.0848	0.2087	0.3421
1234	0.0423	0.0853	0.2109	0.3453
1233	0.0424	0.0854	0.2121	0.3458
1232	0.0425	0.0856	0.2135	0.3476
1231	0.0425	0.0858	0.2149	0.3475
1230	0.0426	0.0861	0.2165	0.3492
1229	0.0426	0.0865	0.2174	0.3498
1228	0.0427	0.0867	0.2191	0.3504
1227	0.0429	0.0869	0.2203	0.3512
1226	0.0429	0.0872	0.2215	0.3515
1225	0.0430	0.0875	0.2236	0.3524
1224	0.0431	0.0879	0.2252	0.3540
1223	0.0432	0.0880	0.2268	0.3542
1222	0.0432	0.0884	0.2285	0.3543
1221	0.0433	0.0888	0.2300	0.3534
1220	0.0435	0.0892	0.2318	0.3542
1219	0.0435	0.0895	0.2335	0.3554
1218	0.0436	0.0900	0.2349	0.3548
1217	0.0437	0.0903	0.2369	0.3538

1216	0.0438	0.0908	0.2393	0.3544
1215	0.0439	0.0912	0.2408	0.3550
1214	0.0439	0.0917	0.2430	0.3544
1213	0.0441	0.0923	0.2458	0.3554
1212	0.0441	0.0926	0.2471	0.3536
1211	0.0441	0.0930	0.2495	0.3518
1210	0.0442	0.0937	0.2518	0.3510
1209	0.0443	0.0943	0.2539	0.3507
1208	0.0444	0.0950	0.2567	0.3507
1207	0.0444	0.0954	0.2593	0.3496
1206	0.0444	0.0960	0.2617	0.3482
1205	0.0444	0.0964	0.2643	0.3468
1204	0.0445	0.0972	0.2670	0.3463
1203	0.0446	0.0980	0.2701	0.3456
1202	0.0446	0.0988	0.2730	0.3443
1201	0.0446	0.0995	0.2751	0.3420
1200	0.0447	0.1006	0.2793	0.3418
1199	0.0448	0.1012	0.2825	0.3411
1198	0.0447	0.1021	0.2853	0.3398
1197	0.0448	0.1030	0.2894	0.3398
1196	0.0448	0.1038	0.2924	0.3367
1195	0.0449	0.1047	0.2958	0.3361
1194	0.0449	0.1056	0.2997	0.3364
1193	0.0451	0.1067	0.3042	0.3368
1192	0.0452	0.1077	0.3083	0.3350
1191	0.0453	0.1087	0.3121	0.3352
1190	0.0453	0.1100	0.3173	0.3357
1189	0.0454	0.1112	0.3216	0.3376
1188	0.0455	0.1125	0.3262	0.3363
1187	0.0456	0.1138	0.3311	0.3365
1186	0.0458	0.1150	0.3363	0.3374
1185	0.0459	0.1162	0.3412	0.3369

1184	0.0461	0.1178	0.3462	0.3368
1183	0.0462	0.1190	0.3510	0.3372
1182	0.0463	0.1202	0.3559	0.3356
1181	0.0465	0.1216	0.3605	0.3365
1180	0.0465	0.1225	0.3643	0.3335
1179	0.0465	0.1237	0.3688	0.3303
1178	0.0466	0.1247	0.3726	0.3276
1177	0.0467	0.1257	0.3761	0.3262
1176	0.0466	0.1265	0.3791	0.3200
1175	0.0465	0.1271	0.3821	0.3146
1174	0.0465	0.1278	0.3845	0.3083
1173	0.0464	0.1280	0.3863	0.3005
1172	0.0463	0.1285	0.3882	0.2945
1171	0.0462	0.1291	0.3907	0.2884
1170	0.0461	0.1294	0.3929	0.2826
1169	0.0461	0.1301	0.3959	0.2779
1168	0.0460	0.1307	0.3986	0.2724
1167	0.0459	0.1315	0.4019	0.2680
1166	0.0459	0.1323	0.4061	0.2658
1165	0.0460	0.1334	0.4109	0.2637
1164	0.0460	0.1343	0.4151	0.2622
1163	0.0460	0.1354	0.4196	0.2610
1162	0.0461	0.1366	0.4250	0.2623
1161	0.0463	0.1379	0.4311	0.2628
1160	0.0464	0.1391	0.4370	0.2637
1159	0.0465	0.1402	0.4429	0.2665
1158	0.0467	0.1417	0.4493	0.2691
1157	0.0467	0.1426	0.4546	0.2701
1156	0.0470	0.1439	0.4611	0.2749
1155	0.0471	0.1449	0.4670	0.2776
1154	0.0473	0.1459	0.4731	0.2815
1153	0.0474	0.1464	0.4780	0.2841

1152	0.0476	0.1469	0.4835	0.2889
1151	0.0478	0.1474	0.4882	0.2933
1150	0.0479	0.1475	0.4921	0.2955
1149	0.0480	0.1475	0.4960	0.2987
1148	0.0483	0.1474	0.4996	0.3041
1147	0.0484	0.1469	0.5022	0.3076
1146	0.0487	0.1463	0.5051	0.3125
1145	0.0488	0.1454	0.5070	0.3161
1144	0.0490	0.1443	0.5084	0.3197
1143	0.0492	0.1432	0.5094	0.3259
1142	0.0494	0.1421	0.5102	0.3296
1141	0.0496	0.1405	0.5106	0.3334
1140	0.0498	0.1390	0.5107	0.3383
1139	0.0499	0.1373	0.5097	0.3423
1138	0.0500	0.1354	0.5085	0.3472
1137	0.0502	0.1337	0.5074	0.3501
1136	0.0503	0.1318	0.5060	0.3543
1135	0.0505	0.1302	0.5045	0.3587
1134	0.0506	0.1285	0.5023	0.3624
1133	0.0507	0.1266	0.5006	0.3655
1132	0.0508	0.1247	0.4974	0.3681
1131	0.0509	0.1230	0.4946	0.3704
1130	0.0510	0.1213	0.4911	0.3746
1129	0.0511	0.1197	0.4870	0.3768
1128	0.0513	0.1181	0.4830	0.3786
1127	0.0514	0.1165	0.4787	0.3804
1126	0.0514	0.1150	0.4731	0.3808
1125	0.0515	0.1136	0.4680	0.3811
1124	0.0516	0.1120	0.4619	0.3795
1123	0.0516	0.1105	0.4544	0.3780
1122	0.0516	0.1091	0.4474	0.3762
1121	0.0517	0.1079	0.4411	0.3748

1120	0.0519	0.1068	0.4333	0.3722
1119	0.0519	0.1054	0.4248	0.3689
1118	0.0520	0.1044	0.4167	0.3644
1117	0.0521	0.1035	0.4090	0.3613
1116	0.0521	0.1022	0.4000	0.3564
1115	0.0522	0.1012	0.3914	0.3500
1114	0.0523	0.1001	0.3828	0.3443
1113	0.0524	0.0993	0.3741	0.3391
1112	0.0526	0.0986	0.3661	0.3351
1111	0.0527	0.0976	0.3572	0.3284
1110	0.0528	0.0971	0.3494	0.3223
1109	0.0530	0.0964	0.3417	0.3158
1108	0.0531	0.0955	0.3339	0.3110
1107	0.0533	0.0948	0.3270	0.3050
1106	0.0536	0.0944	0.3204	0.2999
1105	0.0538	0.0938	0.3141	0.2954
1104	0.0540	0.0933	0.3077	0.2899
1103	0.0543	0.0929	0.3020	0.2846
1102	0.0546	0.0926	0.2963	0.2793
1101	0.0548	0.0923	0.2917	0.2755
1100	0.0552	0.0920	0.2868	0.2719
1099	0.0556	0.0920	0.2830	0.2697
1098	0.0559	0.0918	0.2791	0.2669
1097	0.0563	0.0916	0.2756	0.2640
1096	0.0567	0.0918	0.2733	0.2609
1095	0.0571	0.0918	0.2701	0.2578
1094	0.0576	0.0917	0.2673	0.2551
1093	0.0582	0.0920	0.2655	0.2562
1092	0.0587	0.0924	0.2638	0.2541
1091	0.0592	0.0929	0.2626	0.2544
1090	0.0598	0.0931	0.2616	0.2540
1089	0.0603	0.0933	0.2598	0.2525

1088	0.0610	0.0941	0.2600	0.2537
1087	0.0617	0.0947	0.2596	0.2523
1086	0.0623	0.0952	0.2591	0.2522
1085	0.0630	0.0956	0.2597	0.2550
1084	0.0637	0.0962	0.2600	0.2555
1083	0.0645	0.0973	0.2611	0.2569
1082	0.0653	0.0980	0.2614	0.2585
1081	0.0661	0.0988	0.2628	0.2614
1080	0.0669	0.0998	0.2643	0.2634
1079	0.0678	0.1010	0.2664	0.2677
1078	0.0687	0.1021	0.2683	0.2714
1077	0.0695	0.1030	0.2704	0.2741
1076	0.0704	0.1043	0.2731	0.2784
1075	0.0715	0.1057	0.2765	0.2836
1074	0.0724	0.1070	0.2788	0.2886
1073	0.0733	0.1082	0.2825	0.2941
1072	0.0744	0.1095	0.2864	0.3004
1071	0.0755	0.1108	0.2901	0.3075
1070	0.0764	0.1120	0.2939	0.3131
1069	0.0774	0.1133	0.2983	0.3204
1068	0.0785	0.1146	0.3029	0.3299
1067	0.0795	0.1159	0.3073	0.3382
1066	0.0806	0.1171	0.3130	0.3493
1065	0.0817	0.1184	0.3193	0.3615
1064	0.0828	0.1196	0.3247	0.3701
1063	0.0840	0.1209	0.3311	0.3822
1062	0.0852	0.1222	0.3385	0.3956
1061	0.0864	0.1232	0.3457	0.4088
1060	0.0875	0.1246	0.3536	0.4248
1059	0.0887	0.1256	0.3624	0.4405
1058	0.0900	0.1268	0.3713	0.4583
1057	0.0914	0.1279	0.3805	0.4755

1056	0.0926	0.1291	0.3909	0.4954
1055	0.0940	0.1303	0.4018	0.5153
1054	0.0955	0.1316	0.4142	0.5384
1053	0.0969	0.1328	0.4263	0.5623
1052	0.0985	0.1343	0.4392	0.5857
1051	0.1000	0.1355	0.4537	0.6111
1050	0.1016	0.1369	0.4691	0.6384
1049	0.1034	0.1386	0.4857	0.6684
1048	0.1051	0.1402	0.5029	0.6961
1047	0.1069	0.1419	0.5212	0.7261
1046	0.1088	0.1436	0.5393	0.7569
1045	0.1107	0.1452	0.5582	0.7852
1044	0.1128	0.1470	0.5783	0.8159
1043	0.1150	0.1490	0.5981	0.8453
1042	0.1171	0.1506	0.6174	0.8702
1041	0.1195	0.1527	0.6372	0.8985
1040	0.1220	0.1547	0.6557	0.9225
1039	0.1246	0.1569	0.6737	0.9445
1038	0.1273	0.1589	0.6901	0.9625
1037	0.1301	0.1610	0.7042	0.9767
1036	0.1331	0.1633	0.7174	0.9882
1035	0.1363	0.1655	0.7271	0.9952
1034	0.1396	0.1681	0.7346	1.0000
1033	0.1430	0.1703	0.7390	0.9978
1032	0.1466	0.1728	0.7410	0.9928
1031	0.1504	0.1753	0.7395	0.9835
1030	0.1544	0.1778	0.7358	0.9717
1029	0.1584	0.1805	0.7299	0.9568
1028	0.1628	0.1834	0.7222	0.9392
1027	0.1675	0.1863	0.7129	0.9211
1026	0.1723	0.1893	0.7013	0.8992
1025	0.1773	0.1924	0.6888	0.8761

1024	0.1827	0.1958	0.6764	0.8534
1023	0.1884	0.1994	0.6640	0.8323
1022	0.1945	0.2031	0.6509	0.8097
1021	0.2011	0.2073	0.6388	0.7883
1020	0.2081	0.2120	0.6285	0.7678
1019	0.2155	0.2166	0.6174	0.7479
1018	0.2237	0.2218	0.6082	0.7300
1017	0.2322	0.2272	0.5997	0.7112
1016	0.2415	0.2332	0.5923	0.6945
1015	0.2513	0.2393	0.5863	0.6798
1014	0.2619	0.2457	0.5815	0.6663
1013	0.2732	0.2526	0.5773	0.6532
1012	0.2853	0.2600	0.5748	0.6426
1011	0.2982	0.2676	0.5735	0.6324
1010	0.3123	0.2758	0.5741	0.6250
1009	0.3273	0.2844	0.5753	0.6184
1008	0.3435	0.2934	0.5775	0.6133
1007	0.3608	0.3025	0.5805	0.6061
1006	0.3793	0.3118	0.5851	0.6033
1005	0.3991	0.3216	0.5901	0.6022
1004	0.4202	0.3319	0.5963	0.6008
1003	0.4428	0.3423	0.6039	0.6015
1002	0.4670	0.3529	0.6118	0.6015
1001	0.4924	0.3634	0.6202	0.6030
1000	0.5197	0.3744	0.6297	0.6053
999	0.5480	0.3855	0.6393	0.6071
998	0.5782	0.3971	0.6503	0.6114
997	0.6092	0.4086	0.6611	0.6143
996	0.6417	0.4203	0.6721	0.6185
995	0.6746	0.4323	0.6843	0.6234
994	0.7087	0.4448	0.6968	0.6287
993	0.7428	0.4572	0.7092	0.6334

992	0.7773	0.4703	0.7223	0.6376
991	0.8112	0.4839	0.7356	0.6424
990	0.8445	0.4978	0.7486	0.6469
989	0.8764	0.5119	0.7621	0.6505
988	0.9061	0.5264	0.7749	0.6537
987	0.9327	0.5412	0.7872	0.6575
986	0.9559	0.5563	0.7999	0.6599
985	0.9746	0.5711	0.8111	0.6606
984	0.9882	0.5863	0.8219	0.6614
983	0.9971	0.6016	0.8323	0.6641
982	1.0000	0.6164	0.8413	0.6631
981	0.9975	0.6316	0.8503	0.6628
980	0.9894	0.6470	0.8588	0.6598
979	0.9765	0.6626	0.8661	0.6577
978	0.9590	0.6787	0.8738	0.6558
977	0.9378	0.6956	0.8808	0.6526
976	0.9131	0.7134	0.8880	0.6518
975	0.8866	0.7320	0.8952	0.6473
974	0.8583	0.7520	0.9032	0.6440
973	0.8287	0.7733	0.9115	0.6427
972	0.7982	0.7960	0.9198	0.6401
971	0.7677	0.8198	0.9298	0.6367
970	0.7375	0.8449	0.9400	0.6346
969	0.7072	0.8706	0.9501	0.6322
968	0.6778	0.8962	0.9604	0.6290
967	0.6494	0.9207	0.9706	0.6264
966	0.6218	0.9438	0.9805	0.6245
965	0.5954	0.9639	0.9884	0.6207
964	0.5702	0.9805	0.9944	0.6158
963	0.5459	0.9923	0.9987	0.6111
962	0.5225	0.9991	1.0000	0.6045
961	0.5006	1.0000	0.9981	0.5976

960	0.4797	0.9947	0.9941	0.5906
959	0.4596	0.9831	0.9856	0.5808
958	0.4408	0.9664	0.9740	0.5707
957	0.4230	0.9446	0.9594	0.5595
956	0.4059	0.9184	0.9420	0.5460
955	0.3897	0.8886	0.9215	0.5330
954	0.3745	0.8560	0.8988	0.5196
953	0.3602	0.8217	0.8746	0.5051
952	0.3464	0.7859	0.8485	0.4890
951	0.3337	0.7504	0.8228	0.4753
950	0.3218	0.7148	0.7957	0.4600
949	0.3107	0.6801	0.7689	0.4453
948	0.3004	0.6466	0.7425	0.4303
947	0.2911	0.6148	0.7176	0.4197
946	0.2822	0.5842	0.6925	0.4067
945	0.2741	0.5554	0.6685	0.3940
944	0.2668	0.5287	0.6463	0.3838
943	0.2600	0.5039	0.6249	0.3749
942	0.2539	0.4809	0.6056	0.3664
941	0.2484	0.4594	0.5869	0.3574
940	0.2434	0.4397	0.5694	0.3490
939	0.2389	0.4216	0.5530	0.3437
938	0.2349	0.4047	0.5374	0.3389
937	0.2315	0.3890	0.5235	0.3317
936	0.2283	0.3743	0.5098	0.3257
935	0.2256	0.3610	0.4969	0.3194
934	0.2234	0.3486	0.4849	0.3160
933	0.2215	0.3371	0.4739	0.3108
932	0.2200	0.3268	0.4635	0.3056
931	0.2189	0.3173	0.4542	0.3025
930	0.2182	0.3087	0.4459	0.3002
929	0.2177	0.3007	0.4379	0.2953

928	0.2174	0.2932	0.4298	0.2922
927	0.2174	0.2866	0.4232	0.2905
926	0.2174	0.2804	0.4172	0.2875
925	0.2177	0.2748	0.4113	0.2849
924	0.2178	0.2696	0.4056	0.2830
923	0.2178	0.2644	0.3999	0.2805
922	0.2177	0.2594	0.3947	0.2767
921	0.2175	0.2551	0.3903	0.2754
920	0.2170	0.2508	0.3858	0.2747
919	0.2162	0.2466	0.3814	0.2721
918	0.2150	0.2427	0.3767	0.2705
917	0.2136	0.2389	0.3734	0.2720
916	0.2119	0.2354	0.3705	0.2704
915	0.2099	0.2316	0.3659	0.2668
914	0.2077	0.2282	0.3626	0.2680
913	0.2052	0.2250	0.3587	0.2670
912	0.2026	0.2219	0.3553	0.2671
911	0.1998	0.2189	0.3526	0.2668
910	0.1970	0.2161	0.3496	0.2653
909	0.1943	0.2139	0.3478	0.2657
908	0.1915	0.2117	0.3454	0.2660
907	0.1887	0.2095	0.3446	0.2674
906	0.1862	0.2078	0.3423	0.2684
905	0.1838	0.2063	0.3413	0.2694
904	0.1815	0.2049	0.3406	0.2721
903	0.1793	0.2038	0.3407	0.2766
902	0.1773	0.2031	0.3414	0.2781
901	0.1754	0.2021	0.3403	0.2775
900	0.1738	0.2013	0.3411	0.2788
899	0.1725	0.2009	0.3413	0.2827
898	0.1714	0.2007	0.3425	0.2848
897	0.1704	0.2003	0.3439	0.2875

896	0.1698	0.2005	0.3463	0.2939
895	0.1694	0.2006	0.3476	0.2945
894	0.1691	0.2010	0.3489	0.2968
893	0.1691	0.2016	0.3520	0.3030
892	0.1692	0.2019	0.3534	0.3038
891	0.1696	0.2022	0.3543	0.3041
890	0.1702	0.2029	0.3562	0.3074
889	0.1710	0.2037	0.3591	0.3111
888	0.1720	0.2047	0.3617	0.3151
887	0.1731	0.2055	0.3639	0.3158
886	0.1742	0.2065	0.3644	0.3163
885	0.1754	0.2072	0.3666	0.3192
884	0.1767	0.2083	0.3690	0.3223
883	0.1780	0.2089	0.3705	0.3227
882	0.1791	0.2092	0.3703	0.3221
881	0.1802	0.2098	0.3711	0.3231
880	0.1810	0.2099	0.3704	0.3198
879	0.1817	0.2100	0.3699	0.3187
878	0.1821	0.2095	0.3694	0.3190
877	0.1821	0.2091	0.3674	0.3159
876	0.1822	0.2085	0.3661	0.3134
875	0.1821	0.2077	0.3644	0.3130
874	0.1817	0.2061	0.3617	0.3082
873	0.1812	0.2046	0.3580	0.3047
872	0.1805	0.2036	0.3549	0.3048
871	0.1799	0.2025	0.3530	0.3036
870	0.1794	0.2008	0.3497	0.3026
869	0.1787	0.1992	0.3451	0.2967
868	0.1783	0.1976	0.3418	0.2929
867	0.1781	0.1963	0.3391	0.2905
866	0.1780	0.1952	0.3365	0.2884
865	0.1783	0.1943	0.3344	0.2868

864	0.1787	0.1938	0.3330	0.2855
863	0.1792	0.1932	0.3301	0.2817
862	0.1800	0.1934	0.3298	0.2825
861	0.1808	0.1930	0.3274	0.2782
860	0.1817	0.1931	0.3255	0.2743
859	0.1828	0.1932	0.3255	0.2728
858	0.1840	0.1938	0.3254	0.2716
857	0.1851	0.1947	0.3237	0.2695
856	0.1862	0.1953	0.3237	0.2681
855	0.1874	0.1964	0.3242	0.2671
854	0.1884	0.1973	0.3244	0.2655
853	0.1895	0.1986	0.3248	0.2645
852	0.1906	0.2007	0.3266	0.2666
851	0.1914	0.2024	0.3280	0.2672
850	0.1916	0.2035	0.3344	0.2664
849	0.1923	0.2051	0.3352	0.2632
848	0.1927	0.2073	0.3356	0.2656
847	0.1931	0.2094	0.3377	0.2660
846	0.1936	0.2122	0.3403	0.2674
845	0.1936	0.2146	0.3419	0.2657
844	0.1936	0.2179	0.3423	0.2625
843	0.1933	0.2205	0.3426	0.2585
842	0.1931	0.2240	0.3456	0.2598
841	0.1928	0.2276	0.3476	0.2630
840	0.1924	0.2309	0.3489	0.2609
839	0.1919	0.2348	0.3529	0.2603
838	0.1914	0.2379	0.3563	0.2603
837	0.1908	0.2416	0.3577	0.2577
836	0.1900	0.2444	0.3593	0.2530
835	0.1891	0.2472	0.3611	0.2516
834	0.1881	0.2496	0.3634	0.2520
833	0.1871	0.2515	0.3637	0.2497

832	0.1859	0.2532	0.3656	0.2521
831	0.1843	0.2532	0.3645	0.2524
830	0.1827	0.2529	0.3633	0.2498
829	0.1811	0.2524	0.3635	0.2492
828	0.1794	0.2513	0.3617	0.2454
827	0.1775	0.2496	0.3607	0.2444
826	0.1755	0.2472	0.3574	0.2441
825	0.1736	0.2449	0.3537	0.2411
824	0.1716	0.2423	0.3503	0.2389
823	0.1695	0.2391	0.3463	0.2360
822	0.1674	0.2359	0.3434	0.2360
821	0.1653	0.2327	0.3395	0.2325
820	0.1634	0.2301	0.3367	0.2322
819	0.1612	0.2263	0.3314	0.2275
818	0.1591	0.2228	0.3270	0.2260
817	0.1570	0.2197	0.3228	0.2237
816	0.1550	0.2166	0.3196	0.2217
815	0.1531	0.2139	0.3165	0.2220
814	0.1512	0.2108	0.3115	0.2203
813	0.1495	0.2080	0.3084	0.2205
812	0.1477	0.2053	0.3048	0.2161
811	0.1457	0.2025	0.3012	0.2152
810	0.1441	0.2000	0.2982	0.2144
809	0.1424	0.1978	0.2954	0.2121
808	0.1407	0.1957	0.2925	0.2101
807	0.1392	0.1932	0.2898	0.2087
806	0.1376	0.1908	0.2854	0.2070
805	0.1361	0.1889	0.2835	0.2076
804	0.1346	0.1868	0.2805	0.2048
803	0.1331	0.1850	0.2778	0.2035
802	0.1317	0.1827	0.2758	0.2024
801	0.1303	0.1806	0.2724	0.2003

800	0.1289	0.1791	0.2703	0.2000
799	0.1276	0.1770	0.2681	0.1974
798	0.1263	0.1750	0.2659	0.1943
797	0.1250	0.1735	0.2645	0.1956
796	0.1237	0.1719	0.2620	0.1953
795	0.1225	0.1700	0.2597	0.1939
794	0.1213	0.1681	0.2574	0.1921
793	0.1202	0.1667	0.2556	0.1903
792	0.1193	0.1652	0.2543	0.1910
791	0.1183	0.1637	0.2523	0.1913
790	0.1173	0.1625	0.2510	0.1907
789	0.1165	0.1610	0.2494	0.1893
788	0.1156	0.1597	0.2484	0.1884
787	0.1149	0.1587	0.2473	0.1891
786	0.1142	0.1572	0.2455	0.1887
785	0.1135	0.1559	0.2439	0.1870
784	0.1130	0.1548	0.2438	0.1861
783	0.1125	0.1538	0.2428	0.1874
782	0.1121	0.1531	0.2419	0.1872
781	0.1116	0.1519	0.2400	0.1866
780	0.1112	0.1512	0.2394	0.1869
779	0.1108	0.1502	0.2387	0.1842
778	0.1104	0.1490	0.2380	0.1831
777	0.1101	0.1483	0.2376	0.1849
776	0.1097	0.1478	0.2361	0.1867
775	0.1093	0.1469	0.2351	0.1860
774	0.1088	0.1463	0.2344	0.1839
773	0.1085	0.1456	0.2343	0.1839
772	0.1081	0.1453	0.2337	0.1843
771	0.1076	0.1446	0.2324	0.1832
770	0.1071	0.1440	0.2312	0.1808
769	0.1067	0.1437	0.2314	0.1821

768	0.1063	0.1436	0.2310	0.1828
767	0.1060	0.1431	0.2311	0.1831
766	0.1054	0.1429	0.2304	0.1830
765	0.1050	0.1429	0.2302	0.1844
764	0.1046	0.1427	0.2296	0.1850
763	0.1040	0.1421	0.2294	0.1836
762	0.1036	0.1419	0.2286	0.1828
761	0.1032	0.1420	0.2292	0.1855
760	0.1027	0.1419	0.2281	0.1849
759	0.1024	0.1420	0.2284	0.1857
758	0.1019	0.1415	0.2269	0.1839
757	0.1015	0.1415	0.2269	0.1830
756	0.1012	0.1415	0.2280	0.1857
755	0.1008	0.1414	0.2276	0.1851
754	0.1005	0.1413	0.2270	0.1834
753	0.1000	0.1413	0.2272	0.1849
752	0.0998	0.1413	0.2273	0.1852
751	0.0995	0.1416	0.2281	0.1872
750	0.0993	0.1416	0.2282	0.1875
749	0.0990	0.1417	0.2286	0.1855
748	0.0987	0.1419	0.2278	0.1856
747	0.0984	0.1422	0.2283	0.1855
746	0.0983	0.1424	0.2284	0.1861
745	0.0981	0.1427	0.2283	0.1876
744	0.0979	0.1432	0.2292	0.1886
743	0.0977	0.1435	0.2298	0.1861
742	0.0975	0.1438	0.2297	0.1865
741	0.0973	0.1442	0.2301	0.1862
740	0.0972	0.1446	0.2310	0.1873
739	0.0970	0.1447	0.2311	0.1887
738	0.0968	0.1447	0.2314	0.1875
737	0.0968	0.1452	0.2316	0.1893

736	0.0966	0.1452	0.2322	0.1884
735	0.0965	0.1452	0.2320	0.1869
734	0.0965	0.1454	0.2321	0.1874
733	0.0964	0.1456	0.2324	0.1884
732	0.0962	0.1455	0.2329	0.1871
731	0.0961	0.1454	0.2330	0.1858
730	0.0962	0.1455	0.2337	0.1878
729	0.0961	0.1457	0.2336	0.1869
728	0.0960	0.1457	0.2339	0.1864
727	0.0961	0.1458	0.2340	0.1873
726	0.0962	0.1460	0.2342	0.1881
725	0.0962	0.1464	0.2347	0.1881
724	0.0963	0.1466	0.2346	0.1874
723	0.0964	0.1470	0.2349	0.1871
722	0.0965	0.1473	0.2346	0.1854
721	0.0966	0.1475	0.2353	0.1853
720	0.0968	0.1480	0.2365	0.1866
719	0.0971	0.1487	0.2366	0.1861
718	0.0974	0.1496	0.2378	0.1879
717	0.0978	0.1505	0.2390	0.1907
716	0.0981	0.1512	0.2399	0.1901
715	0.0985	0.1522	0.2414	0.1905
714	0.0990	0.1533	0.2422	0.1915
713	0.0995	0.1545	0.2436	0.1912
712	0.1000	0.1557	0.2451	0.1915
711	0.1006	0.1571	0.2468	0.1936
710	0.1013	0.1588	0.2489	0.1971
709	0.1019	0.1602	0.2507	0.1966
708	0.1026	0.1623	0.2535	0.1977
707	0.1033	0.1644	0.2562	0.2011
706	0.1040	0.1664	0.2581	0.2017
705	0.1047	0.1687	0.2606	0.2022

704	0.1055	0.1711	0.2632	0.2033
703	0.1063	0.1740	0.2668	0.2056
702	0.1071	0.1770	0.2698	0.2076
701	0.1079	0.1801	0.2732	0.2106
700	0.1087	0.1836	0.2771	0.2112
699	0.1093	0.1873	0.2806	0.2136
698	0.1098	0.1913	0.2848	0.2160
697	0.1104	0.1955	0.2889	0.2173
696	0.1109	0.1999	0.2932	0.2206
695	0.1112	0.2048	0.2977	0.2221
694	0.1114	0.2100	0.3023	0.2240
693	0.1116	0.2156	0.3076	0.2267
692	0.1116	0.2214	0.3124	0.2295
691	0.1115	0.2276	0.3172	0.2300
690	0.1114	0.2344	0.3237	0.2341
689	0.1112	0.2416	0.3296	0.2376
688	0.1110	0.2492	0.3365	0.2409
687	0.1109	0.2573	0.3433	0.2434
686	0.1109	0.2661	0.3514	0.2486
685	0.1109	0.2755	0.3600	0.2520
684	0.1108	0.2849	0.3677	0.2554
683	0.1109	0.2947	0.3769	0.2608
682	0.1111	0.3053	0.3868	0.2657
681	0.1114	0.3164	0.3970	0.2724
680	0.1117	0.3276	0.4075	0.2776
679	0.1120	0.3390	0.4179	0.2828
678	0.1124	0.3507	0.4286	0.2890
677	0.1129	0.3626	0.4401	0.2968
676	0.1133	0.3742	0.4517	0.3042
675	0.1138	0.3853	0.4629	0.3116
674	0.1143	0.3957	0.4730	0.3180
673	0.1147	0.4048	0.4833	0.3251

672	0.1151	0.4129	0.4930	0.3328
671	0.1153	0.4190	0.5017	0.3408
670	0.1155	0.4231	0.5095	0.3483
669	0.1156	0.4248	0.5155	0.3565
668	0.1157	0.4242	0.5215	0.3663
667	0.1156	0.4209	0.5262	0.3750
666	0.1155	0.4150	0.5285	0.3822
665	0.1152	0.4065	0.5298	0.3904
664	0.1150	0.3965	0.5313	0.4012
663	0.1148	0.3850	0.5312	0.4109
662	0.1146	0.3724	0.5313	0.4221
661	0.1145	0.3591	0.5308	0.4334
660	0.1143	0.3456	0.5297	0.4436
659	0.1142	0.3326	0.5291	0.4545
658	0.1142	0.3203	0.5288	0.4662
657	0.1143	0.3087	0.5295	0.4776
656	0.1145	0.2979	0.5292	0.4867
655	0.1147	0.2883	0.5301	0.4974
654	0.1150	0.2793	0.5294	0.5053
653	0.1153	0.2711	0.5273	0.5089
652	0.1157	0.2638	0.5255	0.5130
651	0.1162	0.2575	0.5223	0.5148
650	0.1167	0.2516	0.5178	0.5140
649	0.1171	0.2461	0.5121	0.5103
648	0.1176	0.2411	0.5055	0.5033
647	0.1181	0.2366	0.4968	0.4947
646	0.1186	0.2324	0.4874	0.4853
645	0.1190	0.2283	0.4768	0.4728
644	0.1193	0.2246	0.4658	0.4604
643	0.1196	0.2211	0.4551	0.4473
642	0.1198	0.2178	0.4439	0.4337
641	0.1198	0.2144	0.4326	0.4179

640	0.1197	0.2114	0.4219	0.4052
639	0.1196	0.2085	0.4115	0.3917
638	0.1194	0.2055	0.4012	0.3783
637	0.1191	0.2028	0.3919	0.3672
636	0.1188	0.2001	0.3827	0.3557
635	0.1185	0.1977	0.3745	0.3443
634	0.1183	0.1955	0.3674	0.3361
633	0.1181	0.1936	0.3607	0.3278
632	0.1180	0.1917	0.3549	0.3201
631	0.1181	0.1900	0.3491	0.3131
630	0.1183	0.1885	0.3443	0.3063
629	0.1185	0.1872	0.3402	0.3006
628	0.1188	0.1860	0.3361	0.2945
627	0.1194	0.1854	0.3335	0.2910
626	0.1199	0.1847	0.3310	0.2874
625	0.1205	0.1841	0.3286	0.2831
624	0.1212	0.1839	0.3264	0.2807
623	0.1220	0.1837	0.3249	0.2763
622	0.1229	0.1838	0.3239	0.2752
621	0.1238	0.1841	0.3232	0.2719
620	0.1249	0.1848	0.3231	0.2722
619	0.1260	0.1856	0.3232	0.2712
618	0.1272	0.1864	0.3233	0.2691
617	0.1285	0.1876	0.3238	0.2693
616	0.1299	0.1887	0.3249	0.2695
615	0.1315	0.1903	0.3265	0.2698
614	0.1331	0.1919	0.3281	0.2691
613	0.1349	0.1938	0.3303	0.2708
612	0.1367	0.1956	0.3327	0.2716
611	0.1387	0.1976	0.3355	0.2734
610	0.1408	0.1998	0.3389	0.2753
609	0.1430	0.2019	0.3425	0.2773

608	0.1454	0.2040	0.3468	0.2818
607	0.1479	0.2062	0.3507	0.2846
606	0.1505	0.2082	0.3557	0.2877
605	0.1533	0.2102	0.3603	0.2905
604	0.1562	0.2122	0.3654	0.2966
603	0.1594	0.2141	0.3707	0.3025
602	0.1628	0.2157	0.3769	0.3072
601	0.1666	0.2176	0.3837	0.3141
600	0.1706	0.2192	0.3894	0.3198
599	0.1748	0.2206	0.3964	0.3258
598	0.1795	0.2224	0.4031	0.3337
597	0.1845	0.2240	0.4097	0.3398
596	0.1898	0.2257	0.4161	0.3459
595	0.1957	0.2275	0.4220	0.3524
594	0.2019	0.2293	0.4274	0.3580
593	0.2086	0.2310	0.4320	0.3620
592	0.2158	0.2328	0.4351	0.3654
591	0.2235	0.2348	0.4386	0.3687
590	0.2317	0.2368	0.4414	0.3704
589	0.2404	0.2388	0.4429	0.3716
588	0.2495	0.2409	0.4444	0.3727
587	0.2591	0.2431	0.4458	0.3732
586	0.2690	0.2450	0.4463	0.3724
585	0.2793	0.2469	0.4469	0.3718
584	0.2898	0.2488	0.4478	0.3713
583	0.3007	0.2508	0.4485	0.3709
582	0.3120	0.2527	0.4489	0.3698
581	0.3237	0.2547	0.4500	0.3693
580	0.3358	0.2565	0.4504	0.3675
579	0.3485	0.2588	0.4523	0.3677
578	0.3618	0.2611	0.4546	0.3679
577	0.3753	0.2637	0.4570	0.3676

576	0.3887	0.2661	0.4592	0.3670
575	0.4018	0.2686	0.4616	0.3681
574	0.4141	0.2709	0.4640	0.3663
573	0.4247	0.2729	0.4659	0.3652
572	0.4331	0.2747	0.4678	0.3648
571	0.4387	0.2756	0.4684	0.3613
570	0.4411	0.2759	0.4692	0.3591
569	0.4400	0.2756	0.4691	0.3565
568	0.4353	0.2744	0.4681	0.3514
567	0.4269	0.2723	0.4659	0.3472
566	0.4155	0.2698	0.4638	0.3424
565	0.4015	0.2664	0.4608	0.3352
564	0.3857	0.2628	0.4582	0.3289
563	0.3689	0.2592	0.4559	0.3232
562	0.3515	0.2556	0.4536	0.3185
561	0.3339	0.2518	0.4517	0.3120
560	0.3169	0.2483	0.4514	0.3061
559	0.3007	0.2450	0.4511	0.3010
558	0.2854	0.2420	0.4509	0.2968
557	0.2714	0.2394	0.4508	0.2920
556	0.2585	0.2365	0.4496	0.2867
555	0.2469	0.2333	0.4470	0.2820
554	0.2364	0.2302	0.4439	0.2783
553	0.2270	0.2267	0.4382	0.2745
552	0.2186	0.2231	0.4318	0.2712
551	0.2111	0.2192	0.4240	0.2671
550	0.2044	0.2150	0.4150	0.2642
549	0.1984	0.2109	0.4055	0.2605
548	0.1931	0.2069	0.3958	0.2577
547	0.1884	0.2031	0.3860	0.2538
546	0.1842	0.1997	0.3771	0.2517
545	0.1805	0.1966	0.3689	0.2502

544	0.1772	0.1934	0.3610	0.2468
543	0.1743	0.1908	0.3539	0.2439
542	0.1717	0.1886	0.3479	0.2429
541	0.1696	0.1868	0.3426	0.2430
540	0.1676	0.1850	0.3380	0.2402
539	0.1660	0.1832	0.3332	0.2383
538	0.1646	0.1819	0.3291	0.2367
537	0.1636	0.1808	0.3253	0.2356
536	0.1629	0.1800	0.3225	0.2355
535	0.1625	0.1795	0.3207	0.2355
534	0.1623	0.1790	0.3183	0.2347
533	0.1625	0.1787	0.3158	0.2329
532	0.1629	0.1785	0.3142	0.2307
531	0.1637	0.1788	0.3135	0.2326
530	0.1648	0.1793	0.3133	0.2324
529	0.1660	0.1797	0.3126	0.2318
528	0.1674	0.1803	0.3122	0.2310
527	0.1690	0.1809	0.3120	0.2305
526	0.1707	0.1818	0.3123	0.2305
525	0.1724	0.1830	0.3133	0.2320
524	0.1740	0.1842	0.3135	0.2320
523	0.1754	0.1854	0.3146	0.2327
522	0.1767	0.1867	0.3158	0.2338
521	0.1777	0.1881	0.3170	0.2340
520	0.1783	0.1894	0.3176	0.2342
519	0.1788	0.1909	0.3187	0.2352
518	0.1789	0.1925	0.3208	0.2360
517	0.1788	0.1940	0.3223	0.2361
516	0.1783	0.1956	0.3239	0.2363
515	0.1776	0.1970	0.3251	0.2359
514	0.1768	0.1986	0.3275	0.2373
513	0.1758	0.2001	0.3295	0.2372

512	0.1746	0.2011	0.3307	0.2361
511	0.1733	0.2023	0.3319	0.2382
510	0.1718	0.2027	0.3329	0.2367
509	0.1701	0.2030	0.3339	0.2365
508	0.1682	0.2026	0.3332	0.2358
507	0.1662	0.2017	0.3329	0.2338
506	0.1641	0.2007	0.3327	0.2331
505	0.1619	0.1991	0.3311	0.2315
504	0.1597	0.1972	0.3293	0.2294
503	0.1574	0.1952	0.3274	0.2278
502	0.1553	0.1931	0.3252	0.2275
501	0.1532	0.1910	0.3229	0.2252
500	0.1513	0.1889	0.3211	0.2216
499	0.1495	0.1871	0.3191	0.2203
498	0.1478	0.1854	0.3169	0.2198
497	0.1463	0.1837	0.3148	0.2171
496	0.1449	0.1824	0.3131	0.2163
495	0.1438	0.1813	0.3118	0.2149
494	0.1427	0.1802	0.3108	0.2133
493	0.1418	0.1795	0.3092	0.2124
492	0.1410	0.1789	0.3082	0.2117
491	0.1403	0.1783	0.3076	0.2112
490	0.1395	0.1777	0.3066	0.2102
489	0.1389	0.1769	0.3048	0.2094
488	0.1382	0.1758	0.3041	0.2087
487	0.1374	0.1750	0.3029	0.2085
486	0.1366	0.1740	0.3009	0.2066
485	0.1358	0.1728	0.2992	0.2056
484	0.1350	0.1717	0.2974	0.2035
483	0.1343	0.1707	0.2958	0.2030
482	0.1335	0.1694	0.2939	0.2029
481	0.1328	0.1685	0.2929	0.2033

480	0.1323	0.1675	0.2917	0.2032
479	0.1318	0.1667	0.2901	0.2014
478	0.1314	0.1659	0.2892	0.1998
477	0.1311	0.1654	0.2889	0.2016
476	0.1308	0.1651	0.2880	0.2013
475	0.1307	0.1650	0.2875	0.2023
474	0.1306	0.1648	0.2877	0.2015
473	0.1307	0.1649	0.2879	0.2013
472	0.1308	0.1650	0.2879	0.2015
471	0.1308	0.1650	0.2878	0.2006
470	0.1309	0.1651	0.2882	0.2016
469	0.1309	0.1651	0.2882	0.2013
468	0.1309	0.1652	0.2889	0.2005
467	0.1308	0.1651	0.2882	0.1998
466	0.1309	0.1651	0.2885	0.2005
465	0.1308	0.1648	0.2880	0.1990
464	0.1307	0.1646	0.2881	0.2002
463	0.1305	0.1642	0.2879	0.1999
462	0.1303	0.1639	0.2877	0.1990
461	0.1301	0.1636	0.2868	0.1985
460	0.1298	0.1631	0.2864	0.1987
459	0.1295	0.1624	0.2856	0.1968
458	0.1292	0.1620	0.2855	0.1974
457	0.1289	0.1615	0.2850	0.1972
456	0.1285	0.1607	0.2837	0.1961
455	0.1280	0.1599	0.2822	0.1941
454	0.1277	0.1594	0.2818	0.1957
453	0.1273	0.1587	0.2808	0.1941
452	0.1269	0.1577	0.2790	0.1916
451	0.1266	0.1571	0.2782	0.1913
450	0.1264	0.1565	0.2767	0.1906
449	0.1261	0.1557	0.2757	0.1899

448	0.1260	0.1552	0.2753	0.1894
447	0.1259	0.1547	0.2749	0.1887
446	0.1257	0.1539	0.2736	0.1879
445	0.1258	0.1535	0.2732	0.1883
444	0.1256	0.1531	0.2728	0.1891
443	0.1255	0.1527	0.2722	0.1865
442	0.1255	0.1523	0.2715	0.1854
441	0.1254	0.1521	0.2710	0.1870
440	0.1253	0.1517	0.2706	0.1870
439	0.1253	0.1513	0.2697	0.1856
438	0.1252	0.1513	0.2704	0.1863
437	0.1252	0.1512	0.2709	0.1868
436	0.1251	0.1514	0.2715	0.1871
435	0.1252	0.1515	0.2716	0.1888
434	0.1252	0.1516	0.2718	0.1880
433	0.1251	0.1514	0.2721	0.1851
432	0.1252	0.1518	0.2728	0.1851
431	0.1253	0.1521	0.2741	0.1877
430	0.1254	0.1524	0.2751	0.1872
429	0.1256	0.1527	0.2758	0.1878
428	0.1257	0.1531	0.2766	0.1875
427	0.1258	0.1537	0.2778	0.1876
426	0.1259	0.1543	0.2791	0.1884
425	0.1261	0.1548	0.2803	0.1887
424	0.1264	0.1556	0.2821	0.1891
423	0.1267	0.1565	0.2843	0.1912
422	0.1270	0.1576	0.2872	0.1908
421	0.1274	0.1586	0.2900	0.1908
420	0.1275	0.1593	0.2915	0.1934
419	0.1279	0.1605	0.2939	0.1926
418	0.1283	0.1619	0.2975	0.1940
417	0.1288	0.1630	0.3005	0.1944

416	0.1294	0.1650	0.3063	0.1978
415	0.1298	0.1667	0.3093	0.1977
414	0.1301	0.1682	0.3125	0.1981
413	0.1306	0.1701	0.3185	0.2026
412	0.1311	0.1721	0.3242	0.2028
411	0.1314	0.1737	0.3287	0.2035
410	0.1319	0.1760	0.3346	0.2061
409	0.1322	0.1780	0.3405	0.2076
408	0.1326	0.1799	0.3462	0.2107
407	0.1329	0.1818	0.3516	0.2124
406	0.1333	0.1833	0.3566	0.2144
405	0.1337	0.1849	0.3609	0.2158
404	0.1340	0.1863	0.3649	0.2199
403	0.1343	0.1874	0.3686	0.2218
402	0.1346	0.1884	0.3706	0.2227
401	0.1349	0.1889	0.3714	0.2242
400	0.1353	0.1897	0.3722	0.2262

Wavelength	SWNT-$(CF_2)_3CF_3$ DOC0.019	SWNT-$(CF_2)_3CF_3$ DOC0.023	SWNT-$(CF_2)_3CF_3$ DOC0.027	SWNT-$(CF_2)_3CF_3$ DOC0.029
1300	0.0234	0.0340	0.0222	0.0257
1299	0.0234	0.0342	0.0223	0.0257
1298	0.0233	0.0342	0.0224	0.0255
1297	0.0233	0.0344	0.0225	0.0255
1296	0.0234	0.0345	0.0226	0.0259
1295	0.0235	0.0347	0.0227	0.0259
1294	0.0235	0.0348	0.0228	0.0258
1293	0.0238	0.0350	0.0231	0.0262
1292	0.0238	0.0351	0.0232	0.0263
1291	0.0239	0.0353	0.0233	0.0263
1290	0.0241	0.0355	0.0235	0.0267

1289	0.0242	0.0356	0.0237	0.0269
1288	0.0243	0.0358	0.0238	0.0271
1287	0.0244	0.0360	0.0240	0.0272
1286	0.0244	0.0361	0.0242	0.0275
1285	0.0247	0.0363	0.0244	0.0276
1284	0.0248	0.0365	0.0246	0.0277
1283	0.0248	0.0366	0.0247	0.0277
1282	0.0249	0.0368	0.0249	0.0282
1281	0.0251	0.0370	0.0250	0.0283
1280	0.0251	0.0371	0.0251	0.0284
1279	0.0252	0.0372	0.0253	0.0284
1278	0.0253	0.0374	0.0254	0.0285
1277	0.0254	0.0376	0.0255	0.0288
1276	0.0255	0.0377	0.0256	0.0287
1275	0.0255	0.0379	0.0257	0.0288
1274	0.0257	0.0381	0.0259	0.0289
1273	0.0257	0.0383	0.0261	0.0292
1272	0.0258	0.0385	0.0262	0.0294
1271	0.0259	0.0387	0.0264	0.0296
1270	0.0260	0.0388	0.0265	0.0298
1269	0.0261	0.0389	0.0266	0.0301
1268	0.0263	0.0391	0.0267	0.0304
1267	0.0265	0.0393	0.0269	0.0308
1266	0.0268	0.0395	0.0272	0.0314
1265	0.0269	0.0397	0.0274	0.0316
1264	0.0272	0.0399	0.0276	0.0321
1263	0.0273	0.0401	0.0278	0.0323
1262	0.0275	0.0403	0.0280	0.0328
1261	0.0279	0.0405	0.0282	0.0333
1260	0.0281	0.0407	0.0285	0.0338
1259	0.0282	0.0409	0.0287	0.0342
1258	0.0285	0.0412	0.0289	0.0347

1257	0.0288	0.0414	0.0291	0.0352
1256	0.0290	0.0416	0.0294	0.0357
1255	0.0294	0.0419	0.0296	0.0362
1254	0.0295	0.0420	0.0298	0.0365
1253	0.0298	0.0423	0.0301	0.0372
1252	0.0301	0.0426	0.0304	0.0377
1251	0.0303	0.0429	0.0307	0.0383
1250	0.0306	0.0432	0.0310	0.0388
1249	0.0309	0.0434	0.0313	0.0394
1248	0.0312	0.0437	0.0316	0.0400
1247	0.0316	0.0440	0.0319	0.0406
1246	0.0317	0.0443	0.0322	0.0409
1245	0.0319	0.0445	0.0325	0.0414
1244	0.0321	0.0448	0.0328	0.0418
1243	0.0323	0.0451	0.0331	0.0424
1242	0.0326	0.0454	0.0335	0.0427
1241	0.0329	0.0457	0.0338	0.0432
1240	0.0331	0.0460	0.0341	0.0435
1239	0.0332	0.0463	0.0345	0.0438
1238	0.0334	0.0466	0.0349	0.0443
1237	0.0336	0.0469	0.0353	0.0447
1236	0.0338	0.0472	0.0356	0.0448
1235	0.0340	0.0475	0.0360	0.0452
1234	0.0342	0.0478	0.0364	0.0458
1233	0.0344	0.0481	0.0368	0.0460
1232	0.0345	0.0485	0.0372	0.0463
1231	0.0347	0.0488	0.0377	0.0467
1230	0.0349	0.0491	0.0382	0.0469
1229	0.0349	0.0495	0.0386	0.0471
1228	0.0350	0.0498	0.0391	0.0473
1227	0.0351	0.0501	0.0396	0.0476
1226	0.0351	0.0505	0.0402	0.0477

1225	0.0354	0.0509	0.0407	0.0481
1224	0.0355	0.0512	0.0413	0.0481
1223	0.0356	0.0515	0.0419	0.0484
1222	0.0357	0.0519	0.0425	0.0485
1221	0.0359	0.0524	0.0432	0.0489
1220	0.0359	0.0528	0.0440	0.0491
1219	0.0360	0.0532	0.0447	0.0495
1218	0.0362	0.0536	0.0455	0.0498
1217	0.0362	0.0540	0.0462	0.0497
1216	0.0364	0.0544	0.0470	0.0499
1215	0.0365	0.0548	0.0478	0.0500
1214	0.0365	0.0552	0.0485	0.0500
1213	0.0365	0.0557	0.0493	0.0499
1212	0.0366	0.0561	0.0500	0.0501
1211	0.0367	0.0566	0.0508	0.0504
1210	0.0369	0.0570	0.0515	0.0505
1209	0.0370	0.0575	0.0522	0.0507
1208	0.0371	0.0579	0.0528	0.0510
1207	0.0371	0.0583	0.0534	0.0510
1206	0.0372	0.0588	0.0540	0.0512
1205	0.0372	0.0593	0.0546	0.0511
1204	0.0373	0.0597	0.0551	0.0511
1203	0.0372	0.0601	0.0557	0.0511
1202	0.0375	0.0606	0.0563	0.0512
1201	0.0375	0.0611	0.0569	0.0515
1200	0.0377	0.0616	0.0575	0.0516
1199	0.0377	0.0621	0.0581	0.0517
1198	0.0378	0.0626	0.0587	0.0520
1197	0.0379	0.0630	0.0594	0.0523
1196	0.0379	0.0634	0.0600	0.0522
1195	0.0380	0.0639	0.0608	0.0525
1194	0.0381	0.0643	0.0615	0.0529

1193	0.0382	0.0648	0.0623	0.0528
1192	0.0383	0.0652	0.0631	0.0529
1191	0.0384	0.0657	0.0640	0.0533
1190	0.0386	0.0662	0.0649	0.0536
1189	0.0387	0.0665	0.0658	0.0537
1188	0.0387	0.0670	0.0667	0.0540
1187	0.0389	0.0675	0.0676	0.0546
1186	0.0391	0.0679	0.0686	0.0548
1185	0.0393	0.0683	0.0695	0.0554
1184	0.0395	0.0687	0.0703	0.0556
1183	0.0396	0.0691	0.0712	0.0557
1182	0.0396	0.0694	0.0720	0.0560
1181	0.0396	0.0697	0.0726	0.0559
1180	0.0397	0.0700	0.0733	0.0561
1179	0.0397	0.0702	0.0737	0.0561
1178	0.0396	0.0705	0.0741	0.0560
1177	0.0397	0.0707	0.0744	0.0559
1176	0.0394	0.0707	0.0745	0.0553
1175	0.0392	0.0708	0.0744	0.0549
1174	0.0390	0.0708	0.0743	0.0543
1173	0.0387	0.0709	0.0740	0.0536
1172	0.0384	0.0709	0.0735	0.0530
1171	0.0383	0.0709	0.0731	0.0525
1170	0.0381	0.0709	0.0724	0.0519
1169	0.0380	0.0709	0.0717	0.0515
1168	0.0378	0.0709	0.0709	0.0509
1167	0.0377	0.0709	0.0701	0.0503
1166	0.0376	0.0710	0.0692	0.0499
1165	0.0376	0.0711	0.0682	0.0496
1164	0.0377	0.0711	0.0674	0.0494
1163	0.0377	0.0712	0.0664	0.0491
1162	0.0379	0.0713	0.0655	0.0491

1161	0.0380	0.0713	0.0646	0.0489
1160	0.0381	0.0714	0.0637	0.0485
1159	0.0381	0.0715	0.0629	0.0485
1158	0.0385	0.0716	0.0622	0.0488
1157	0.0386	0.0718	0.0614	0.0487
1156	0.0387	0.0718	0.0608	0.0484
1155	0.0390	0.0719	0.0602	0.0486
1154	0.0392	0.0720	0.0595	0.0486
1153	0.0393	0.0721	0.0590	0.0484
1152	0.0395	0.0722	0.0585	0.0484
1151	0.0396	0.0723	0.0580	0.0482
1150	0.0399	0.0724	0.0576	0.0484
1149	0.0401	0.0724	0.0572	0.0484
1148	0.0402	0.0725	0.0568	0.0481
1147	0.0404	0.0726	0.0565	0.0481
1146	0.0406	0.0727	0.0563	0.0481
1145	0.0408	0.0728	0.0559	0.0481
1144	0.0409	0.0728	0.0557	0.0480
1143	0.0410	0.0729	0.0554	0.0480
1142	0.0412	0.0730	0.0552	0.0479
1141	0.0413	0.0730	0.0550	0.0479
1140	0.0414	0.0732	0.0548	0.0478
1139	0.0415	0.0732	0.0545	0.0476
1138	0.0415	0.0733	0.0543	0.0473
1137	0.0416	0.0733	0.0541	0.0471
1136	0.0418	0.0734	0.0539	0.0469
1135	0.0418	0.0735	0.0537	0.0470
1134	0.0418	0.0736	0.0535	0.0467
1133	0.0420	0.0737	0.0533	0.0465
1132	0.0421	0.0737	0.0531	0.0464
1131	0.0420	0.0738	0.0528	0.0463
1130	0.0419	0.0738	0.0526	0.0459

1129	0.0420	0.0739	0.0524	0.0458
1128	0.0420	0.0741	0.0522	0.0457
1127	0.0419	0.0741	0.0519	0.0454
1126	0.0420	0.0743	0.0517	0.0452
1125	0.0421	0.0744	0.0515	0.0448
1124	0.0421	0.0746	0.0513	0.0447
1123	0.0422	0.0747	0.0512	0.0445
1122	0.0421	0.0749	0.0509	0.0442
1121	0.0421	0.0751	0.0508	0.0442
1120	0.0423	0.0754	0.0507	0.0441
1119	0.0424	0.0756	0.0506	0.0440
1118	0.0424	0.0759	0.0504	0.0437
1117	0.0425	0.0761	0.0503	0.0435
1116	0.0427	0.0765	0.0503	0.0432
1115	0.0426	0.0768	0.0502	0.0432
1114	0.0427	0.0771	0.0501	0.0431
1113	0.0428	0.0775	0.0501	0.0428
1112	0.0430	0.0779	0.0501	0.0427
1111	0.0431	0.0784	0.0501	0.0428
1110	0.0432	0.0789	0.0501	0.0427
1109	0.0434	0.0795	0.0501	0.0426
1108	0.0438	0.0800	0.0503	0.0427
1107	0.0439	0.0806	0.0504	0.0426
1106	0.0441	0.0813	0.0505	0.0426
1105	0.0444	0.0820	0.0506	0.0429
1104	0.0447	0.0828	0.0508	0.0428
1103	0.0451	0.0836	0.0510	0.0430
1102	0.0453	0.0844	0.0512	0.0431
1101	0.0457	0.0853	0.0515	0.0433
1100	0.0460	0.0863	0.0518	0.0434
1099	0.0465	0.0873	0.0521	0.0436
1098	0.0470	0.0884	0.0525	0.0439

1097	0.0473	0.0895	0.0528	0.0439
1096	0.0477	0.0906	0.0532	0.0443
1095	0.0481	0.0919	0.0536	0.0445
1094	0.0486	0.0932	0.0540	0.0450
1093	0.0491	0.0944	0.0545	0.0451
1092	0.0497	0.0958	0.0550	0.0455
1091	0.0501	0.0972	0.0555	0.0457
1090	0.0507	0.0987	0.0561	0.0461
1089	0.0512	0.1002	0.0566	0.0463
1088	0.0518	0.1017	0.0572	0.0467
1087	0.0523	0.1033	0.0578	0.0473
1086	0.0529	0.1050	0.0585	0.0477
1085	0.0537	0.1067	0.0591	0.0482
1084	0.0544	0.1084	0.0598	0.0488
1083	0.0550	0.1101	0.0606	0.0493
1082	0.0558	0.1118	0.0613	0.0496
1081	0.0565	0.1136	0.0621	0.0503
1080	0.0572	0.1154	0.0629	0.0511
1079	0.0580	0.1173	0.0638	0.0517
1078	0.0588	0.1192	0.0646	0.0523
1077	0.0597	0.1211	0.0655	0.0531
1076	0.0606	0.1230	0.0665	0.0540
1075	0.0615	0.1250	0.0675	0.0549
1074	0.0624	0.1270	0.0685	0.0556
1073	0.0635	0.1291	0.0695	0.0568
1072	0.0644	0.1312	0.0706	0.0577
1071	0.0652	0.1332	0.0716	0.0585
1070	0.0662	0.1355	0.0728	0.0596
1069	0.0672	0.1379	0.0740	0.0607
1068	0.0682	0.1403	0.0752	0.0616
1067	0.0692	0.1427	0.0765	0.0627
1066	0.0703	0.1454	0.0778	0.0638

1065	0.0713	0.1481	0.0792	0.0650
1064	0.0724	0.1509	0.0806	0.0663
1063	0.0734	0.1539	0.0822	0.0675
1062	0.0742	0.1570	0.0837	0.0686
1061	0.0754	0.1603	0.0853	0.0701
1060	0.0767	0.1638	0.0871	0.0718
1059	0.0779	0.1674	0.0890	0.0735
1058	0.0789	0.1712	0.0908	0.0750
1057	0.0801	0.1751	0.0928	0.0767
1056	0.0812	0.1792	0.0947	0.0783
1055	0.0824	0.1835	0.0968	0.0804
1054	0.0835	0.1879	0.0990	0.0820
1053	0.0846	0.1926	0.1012	0.0840
1052	0.0858	0.1977	0.1036	0.0861
1051	0.0872	0.2029	0.1062	0.0883
1050	0.0884	0.2084	0.1087	0.0907
1049	0.0897	0.2141	0.1113	0.0929
1048	0.0910	0.2202	0.1141	0.0953
1047	0.0925	0.2267	0.1171	0.0979
1046	0.0938	0.2333	0.1200	0.1005
1045	0.0953	0.2403	0.1231	0.1033
1044	0.0967	0.2476	0.1263	0.1059
1043	0.0982	0.2553	0.1297	0.1090
1042	0.0997	0.2634	0.1332	0.1118
1041	0.1014	0.2719	0.1368	0.1149
1040	0.1031	0.2809	0.1405	0.1184
1039	0.1049	0.2903	0.1444	0.1220
1038	0.1066	0.3002	0.1485	0.1254
1037	0.1085	0.3106	0.1527	0.1289
1036	0.1103	0.3215	0.1570	0.1327
1035	0.1123	0.3330	0.1616	0.1368
1034	0.1141	0.3451	0.1663	0.1410

1033	0.1160	0.3577	0.1710	0.1452
1032	0.1179	0.3707	0.1759	0.1495
1031	0.1198	0.3843	0.1809	0.1539
1030	0.1218	0.3985	0.1860	0.1586
1029	0.1236	0.4134	0.1913	0.1636
1028	0.1256	0.4291	0.1969	0.1688
1027	0.1275	0.4453	0.2026	0.1741
1026	0.1294	0.4624	0.2085	0.1802
1025	0.1313	0.4804	0.2147	0.1859
1024	0.1332	0.4990	0.2212	0.1922
1023	0.1350	0.5188	0.2280	0.1987
1022	0.1369	0.5396	0.2350	0.2057
1021	0.1388	0.5614	0.2424	0.2129
1020	0.1407	0.5842	0.2503	0.2209
1019	0.1426	0.6082	0.2586	0.2294
1018	0.1445	0.6334	0.2674	0.2387
1017	0.1464	0.6594	0.2767	0.2480
1016	0.1483	0.6865	0.2865	0.2581
1015	0.1503	0.7144	0.2971	0.2691
1014	0.1522	0.7432	0.3082	0.2809
1013	0.1540	0.7727	0.3199	0.2932
1012	0.1560	0.8025	0.3325	0.3068
1011	0.1580	0.8316	0.3460	0.3213
1010	0.1600	0.8613	0.3603	0.3370
1009	0.1621	0.8893	0.3754	0.3539
1008	0.1640	0.9159	0.3917	0.3718
1007	0.1660	0.9397	0.4089	0.3910
1006	0.1680	0.9602	0.4271	0.4117
1005	0.1698	0.9779	0.4461	0.4335
1004	0.1716	0.9900	0.4669	0.4571
1003	0.1733	0.9975	0.4886	0.4821
1002	0.1749	1.0000	0.5119	0.5088

1001	0.1764	0.9966	0.5363	0.5367
1000	0.1779	0.9880	0.5624	0.5664
999	0.1794	0.9746	0.5897	0.5976
998	0.1805	0.9567	0.6184	0.6300
997	0.1815	0.9355	0.6487	0.6636
996	0.1825	0.9113	0.6799	0.6980
995	0.1833	0.8852	0.7119	0.7331
994	0.1839	0.8577	0.7448	0.7681
993	0.1844	0.8292	0.7786	0.8031
992	0.1849	0.8000	0.8128	0.8374
991	0.1851	0.7708	0.8466	0.8704
990	0.1850	0.7424	0.8789	0.9011
989	0.1849	0.7143	0.9095	0.9289
988	0.1847	0.6873	0.9373	0.9530
987	0.1843	0.6605	0.9612	0.9730
986	0.1834	0.6351	0.9802	0.9876
985	0.1824	0.6106	0.9941	0.9966
984	0.1811	0.5875	1.0000	1.0000
983	0.1795	0.5651	0.9990	0.9971
982	0.1776	0.5439	0.9914	0.9883
981	0.1755	0.5236	0.9759	0.9739
980	0.1731	0.5043	0.9543	0.9544
979	0.1703	0.4859	0.9265	0.9297
978	0.1673	0.4686	0.8943	0.9014
977	0.1642	0.4520	0.8593	0.8702
976	0.1608	0.4362	0.8218	0.8365
975	0.1574	0.4213	0.7832	0.8017
974	0.1537	0.4072	0.7445	0.7657
973	0.1502	0.3940	0.7063	0.7298
972	0.1465	0.3814	0.6689	0.6942
971	0.1431	0.3695	0.6326	0.6599
970	0.1396	0.3581	0.5977	0.6258

969	0.1363	0.3475	0.5647	0.5933
968	0.1332	0.3375	0.5332	0.5622
967	0.1302	0.3281	0.5038	0.5326
966	0.1273	0.3191	0.4763	0.5045
965	0.1246	0.3108	0.4507	0.4783
964	0.1222	0.3031	0.4268	0.4538
963	0.1199	0.2958	0.4049	0.4310
962	0.1179	0.2889	0.3845	0.4094
961	0.1160	0.2825	0.3660	0.3894
960	0.1142	0.2764	0.3487	0.3705
959	0.1127	0.2710	0.3329	0.3530
958	0.1114	0.2658	0.3185	0.3368
957	0.1102	0.2610	0.3054	0.3215
956	0.1091	0.2565	0.2932	0.3071
955	0.1081	0.2522	0.2820	0.2935
954	0.1073	0.2484	0.2719	0.2808
953	0.1067	0.2448	0.2628	0.2692
952	0.1062	0.2415	0.2545	0.2584
951	0.1060	0.2385	0.2472	0.2485
950	0.1060	0.2357	0.2407	0.2393
949	0.1062	0.2333	0.2350	0.2309
948	0.1065	0.2311	0.2300	0.2232
947	0.1068	0.2291	0.2257	0.2161
946	0.1076	0.2274	0.2222	0.2099
945	0.1086	0.2258	0.2194	0.2041
944	0.1096	0.2244	0.2173	0.1989
943	0.1108	0.2232	0.2157	0.1944
942	0.1124	0.2221	0.2150	0.1906
941	0.1141	0.2211	0.2149	0.1874
940	0.1159	0.2204	0.2154	0.1842
939	0.1179	0.2196	0.2166	0.1816
938	0.1200	0.2188	0.2183	0.1793

937	0.1224	0.2182	0.2210	0.1776
936	0.1249	0.2175	0.2245	0.1762
935	0.1276	0.2170	0.2289	0.1753
934	0.1305	0.2164	0.2340	0.1745
933	0.1338	0.2161	0.2402	0.1746
932	0.1374	0.2157	0.2472	0.1748
931	0.1411	0.2152	0.2551	0.1753
930	0.1452	0.2150	0.2637	0.1761
929	0.1495	0.2149	0.2730	0.1772
928	0.1541	0.2148	0.2824	0.1784
927	0.1590	0.2148	0.2916	0.1795
926	0.1642	0.2150	0.3005	0.1808
925	0.1697	0.2153	0.3086	0.1819
924	0.1756	0.2157	0.3149	0.1824
923	0.1820	0.2164	0.3197	0.1829
922	0.1888	0.2172	0.3224	0.1828
921	0.1959	0.2182	0.3226	0.1824
920	0.2038	0.2195	0.3207	0.1818
919	0.2119	0.2211	0.3166	0.1808
918	0.2204	0.2227	0.3104	0.1790
917	0.2295	0.2247	0.3027	0.1768
916	0.2395	0.2270	0.2938	0.1749
915	0.2499	0.2297	0.2841	0.1721
914	0.2608	0.2327	0.2740	0.1697
913	0.2727	0.2361	0.2639	0.1672
912	0.2850	0.2399	0.2539	0.1645
911	0.2979	0.2441	0.2441	0.1618
910	0.3117	0.2486	0.2349	0.1591
909	0.3263	0.2536	0.2262	0.1567
908	0.3416	0.2592	0.2183	0.1543
907	0.3578	0.2653	0.2110	0.1525
906	0.3747	0.2720	0.2043	0.1509

905	0.3923	0.2791	0.1982	0.1491
904	0.4109	0.2867	0.1928	0.1477
903	0.4304	0.2951	0.1880	0.1467
902	0.4511	0.3039	0.1834	0.1456
901	0.4726	0.3136	0.1795	0.1449
900	0.4948	0.3240	0.1762	0.1442
899	0.5180	0.3350	0.1732	0.1438
898	0.5425	0.3467	0.1706	0.1435
897	0.5682	0.3592	0.1684	0.1435
896	0.5954	0.3721	0.1664	0.1438
895	0.6240	0.3862	0.1649	0.1444
894	0.6540	0.4015	0.1636	0.1455
893	0.6854	0.4171	0.1626	0.1466
892	0.7177	0.4337	0.1618	0.1475
891	0.7511	0.4513	0.1614	0.1490
890	0.7855	0.4695	0.1611	0.1506
889	0.8208	0.4884	0.1611	0.1523
888	0.8560	0.5088	0.1614	0.1546
887	0.8903	0.5291	0.1618	0.1568
886	0.9216	0.5497	0.1623	0.1587
885	0.9499	0.5696	0.1629	0.1610
884	0.9735	0.5890	0.1636	0.1632
883	0.9902	0.6071	0.1641	0.1646
882	0.9992	0.6228	0.1647	0.1658
881	1.0000	0.6361	0.1654	0.1675
880	0.9921	0.6460	0.1658	0.1687
879	0.9761	0.6521	0.1660	0.1688
878	0.9518	0.6547	0.1660	0.1691
877	0.9219	0.6526	0.1658	0.1686
876	0.8866	0.6459	0.1654	0.1680
875	0.8476	0.6362	0.1650	0.1673
874	0.8065	0.6231	0.1643	0.1656

873	0.7646	0.6077	0.1637	0.1642
872	0.7220	0.5899	0.1629	0.1626
871	0.6799	0.5707	0.1624	0.1615
870	0.6389	0.5509	0.1619	0.1601
869	0.5999	0.5307	0.1614	0.1585
868	0.5629	0.5102	0.1609	0.1572
867	0.5280	0.4903	0.1607	0.1564
866	0.4954	0.4706	0.1609	0.1555
865	0.4657	0.4521	0.1612	0.1553
864	0.4383	0.4345	0.1616	0.1557
863	0.4128	0.4177	0.1622	0.1557
862	0.3894	0.4019	0.1629	0.1560
861	0.3681	0.3871	0.1637	0.1567
860	0.3487	0.3734	0.1647	0.1576
859	0.3311	0.3608	0.1656	0.1580
858	0.3150	0.3484	0.1666	0.1589
857	0.3007	0.3376	0.1677	0.1601
856	0.2873	0.3275	0.1685	0.1610
855	0.2750	0.3179	0.1694	0.1621
854	0.2638	0.3092	0.1700	0.1624
853	0.2537	0.3009	0.1706	0.1631
852	0.2443	0.2930	0.1709	0.1636
851	0.2361	0.2859	0.1710	0.1643
850	0.2344	0.2845	0.1718	0.1633
849	0.2268	0.2781	0.1719	0.1645
848	0.2203	0.2724	0.1717	0.1647
847	0.2141	0.2667	0.1711	0.1642
846	0.2082	0.2615	0.1704	0.1636
845	0.2028	0.2566	0.1695	0.1632
844	0.1981	0.2521	0.1684	0.1618
843	0.1940	0.2479	0.1674	0.1614
842	0.1899	0.2439	0.1660	0.1609

841	0.1862	0.2401	0.1644	0.1600
840	0.1832	0.2365	0.1628	0.1586
839	0.1803	0.2333	0.1613	0.1573
838	0.1778	0.2300	0.1597	0.1565
837	0.1756	0.2270	0.1583	0.1555
836	0.1732	0.2241	0.1566	0.1540
835	0.1710	0.2214	0.1549	0.1525
834	0.1691	0.2187	0.1535	0.1510
833	0.1672	0.2161	0.1520	0.1496
832	0.1657	0.2136	0.1506	0.1481
831	0.1640	0.2111	0.1492	0.1468
830	0.1625	0.2088	0.1479	0.1453
829	0.1607	0.2064	0.1466	0.1436
828	0.1591	0.2042	0.1453	0.1421
827	0.1577	0.2019	0.1439	0.1402
826	0.1559	0.1996	0.1427	0.1382
825	0.1541	0.1973	0.1415	0.1366
824	0.1525	0.1950	0.1403	0.1351
823	0.1506	0.1928	0.1392	0.1335
822	0.1486	0.1906	0.1380	0.1319
821	0.1468	0.1884	0.1369	0.1303
820	0.1449	0.1861	0.1357	0.1286
819	0.1430	0.1838	0.1346	0.1267
818	0.1407	0.1816	0.1335	0.1247
817	0.1389	0.1795	0.1326	0.1233
816	0.1370	0.1773	0.1317	0.1219
815	0.1350	0.1753	0.1308	0.1203
814	0.1331	0.1732	0.1300	0.1190
813	0.1311	0.1711	0.1292	0.1172
812	0.1292	0.1690	0.1284	0.1155
811	0.1276	0.1670	0.1277	0.1140
810	0.1260	0.1651	0.1271	0.1127

809	0.1245	0.1633	0.1265	0.1114
808	0.1232	0.1614	0.1259	0.1099
807	0.1218	0.1596	0.1253	0.1087
806	0.1207	0.1579	0.1247	0.1075
805	0.1197	0.1564	0.1241	0.1063
804	0.1187	0.1549	0.1236	0.1051
803	0.1180	0.1534	0.1229	0.1035
802	0.1175	0.1522	0.1223	0.1026
801	0.1171	0.1510	0.1215	0.1014
800	0.1166	0.1499	0.1207	0.1004
799	0.1165	0.1489	0.1200	0.0993
798	0.1165	0.1480	0.1191	0.0981
797	0.1167	0.1474	0.1182	0.0975
796	0.1169	0.1468	0.1173	0.0964
795	0.1173	0.1462	0.1163	0.0952
794	0.1179	0.1457	0.1154	0.0941
793	0.1188	0.1454	0.1145	0.0935
792	0.1198	0.1452	0.1136	0.0928
791	0.1210	0.1451	0.1128	0.0921
790	0.1225	0.1452	0.1120	0.0913
789	0.1243	0.1452	0.1113	0.0906
788	0.1264	0.1455	0.1106	0.0900
787	0.1286	0.1459	0.1099	0.0896
786	0.1312	0.1464	0.1095	0.0894
785	0.1337	0.1469	0.1089	0.0883
784	0.1366	0.1476	0.1085	0.0882
783	0.1399	0.1486	0.1080	0.0881
782	0.1433	0.1496	0.1076	0.0880
781	0.1467	0.1507	0.1072	0.0876
780	0.1504	0.1519	0.1069	0.0875
779	0.1539	0.1532	0.1065	0.0872
778	0.1577	0.1547	0.1061	0.0872

777	0.1613	0.1561	0.1058	0.0872
776	0.1646	0.1576	0.1054	0.0871
775	0.1677	0.1590	0.1050	0.0868
774	0.1706	0.1604	0.1045	0.0864
773	0.1733	0.1617	0.1040	0.0863
772	0.1756	0.1630	0.1036	0.0860
771	0.1775	0.1640	0.1031	0.0858
770	0.1788	0.1650	0.1026	0.0855
769	0.1799	0.1658	0.1021	0.0854
768	0.1806	0.1664	0.1016	0.0849
767	0.1807	0.1667	0.1010	0.0842
766	0.1808	0.1670	0.1006	0.0843
765	0.1805	0.1669	0.1000	0.0838
764	0.1798	0.1668	0.0995	0.0833
763	0.1791	0.1665	0.0991	0.0831
762	0.1781	0.1661	0.0984	0.0826
761	0.1771	0.1656	0.0979	0.0823
760	0.1759	0.1648	0.0973	0.0819
759	0.1748	0.1641	0.0969	0.0813
758	0.1735	0.1634	0.0964	0.0811
757	0.1722	0.1627	0.0960	0.0809
756	0.1710	0.1619	0.0956	0.0804
755	0.1698	0.1612	0.0953	0.0803
754	0.1686	0.1604	0.0949	0.0799
753	0.1672	0.1596	0.0944	0.0797
752	0.1660	0.1588	0.0940	0.0794
751	0.1646	0.1580	0.0937	0.0788
750	0.1632	0.1572	0.0934	0.0789
749	0.1617	0.1564	0.0932	0.0789
748	0.1603	0.1555	0.0930	0.0785
747	0.1589	0.1547	0.0927	0.0781
746	0.1571	0.1538	0.0925	0.0776

745	0.1554	0.1529	0.0924	0.0774
744	0.1539	0.1520	0.0922	0.0775
743	0.1522	0.1511	0.0921	0.0773
742	0.1504	0.1502	0.0920	0.0766
741	0.1487	0.1493	0.0921	0.0764
740	0.1475	0.1485	0.0921	0.0768
739	0.1461	0.1475	0.0922	0.0768
738	0.1444	0.1466	0.0923	0.0767
737	0.1427	0.1457	0.0924	0.0766
736	0.1410	0.1448	0.0925	0.0761
735	0.1391	0.1438	0.0926	0.0758
734	0.1375	0.1429	0.0928	0.0758
733	0.1360	0.1421	0.0932	0.0760
732	0.1344	0.1412	0.0934	0.0761
731	0.1327	0.1402	0.0938	0.0758
730	0.1311	0.1394	0.0942	0.0757
729	0.1294	0.1385	0.0948	0.0756
728	0.1276	0.1376	0.0952	0.0755
727	0.1262	0.1368	0.0959	0.0757
726	0.1247	0.1360	0.0965	0.0757
725	0.1231	0.1351	0.0972	0.0755
724	0.1215	0.1343	0.0980	0.0757
723	0.1203	0.1336	0.0988	0.0758
722	0.1192	0.1330	0.0997	0.0759
721	0.1180	0.1324	0.1008	0.0760
720	0.1172	0.1318	0.1020	0.0764
719	0.1162	0.1313	0.1032	0.0766
718	0.1153	0.1309	0.1045	0.0768
717	0.1146	0.1306	0.1058	0.0772
716	0.1140	0.1303	0.1073	0.0775
715	0.1134	0.1302	0.1089	0.0779
714	0.1130	0.1300	0.1107	0.0783

713	0.1124	0.1300	0.1126	0.0788
712	0.1122	0.1301	0.1146	0.0794
711	0.1118	0.1302	0.1167	0.0800
710	0.1115	0.1303	0.1188	0.0806
709	0.1112	0.1305	0.1211	0.0813
708	0.1108	0.1307	0.1234	0.0818
707	0.1105	0.1309	0.1257	0.0824
706	0.1102	0.1312	0.1281	0.0832
705	0.1099	0.1316	0.1306	0.0838
704	0.1098	0.1320	0.1330	0.0848
703	0.1095	0.1323	0.1352	0.0855
702	0.1091	0.1326	0.1373	0.0858
701	0.1089	0.1330	0.1394	0.0867
700	0.1088	0.1334	0.1412	0.0873
699	0.1085	0.1337	0.1426	0.0880
698	0.1081	0.1338	0.1436	0.0882
697	0.1080	0.1338	0.1443	0.0888
696	0.1078	0.1338	0.1445	0.0892
695	0.1077	0.1335	0.1441	0.0894
694	0.1075	0.1333	0.1432	0.0894
693	0.1072	0.1330	0.1418	0.0892
692	0.1070	0.1328	0.1400	0.0891
691	0.1069	0.1326	0.1377	0.0888
690	0.1068	0.1326	0.1352	0.0885
689	0.1065	0.1325	0.1325	0.0880
688	0.1064	0.1324	0.1297	0.0874
687	0.1062	0.1324	0.1269	0.0869
686	0.1061	0.1324	0.1244	0.0865
685	0.1061	0.1326	0.1220	0.0863
684	0.1061	0.1327	0.1198	0.0859
683	0.1060	0.1328	0.1179	0.0854
682	0.1061	0.1330	0.1162	0.0853

681	0.1062	0.1332	0.1148	0.0854
680	0.1062	0.1333	0.1136	0.0855
679	0.1065	0.1335	0.1125	0.0854
678	0.1066	0.1336	0.1116	0.0852
677	0.1067	0.1337	0.1110	0.0854
676	0.1068	0.1338	0.1103	0.0853
675	0.1071	0.1340	0.1099	0.0856
674	0.1071	0.1341	0.1095	0.0857
673	0.1073	0.1342	0.1093	0.0862
672	0.1077	0.1344	0.1091	0.0866
671	0.1079	0.1345	0.1089	0.0868
670	0.1082	0.1345	0.1088	0.0869
669	0.1084	0.1346	0.1087	0.0870
668	0.1087	0.1346	0.1086	0.0871
667	0.1090	0.1346	0.1087	0.0875
666	0.1094	0.1346	0.1087	0.0877
665	0.1096	0.1345	0.1087	0.0879
664	0.1101	0.1345	0.1088	0.0882
663	0.1105	0.1345	0.1090	0.0885
662	0.1109	0.1344	0.1092	0.0887
661	0.1113	0.1344	0.1094	0.0889
660	0.1117	0.1343	0.1097	0.0893
659	0.1121	0.1343	0.1101	0.0898
658	0.1125	0.1343	0.1104	0.0902
657	0.1131	0.1343	0.1109	0.0910
656	0.1136	0.1343	0.1115	0.0917
655	0.1140	0.1344	0.1121	0.0923
654	0.1143	0.1344	0.1127	0.0928
653	0.1147	0.1344	0.1134	0.0936
652	0.1152	0.1344	0.1141	0.0945
651	0.1155	0.1344	0.1148	0.0950
650	0.1159	0.1343	0.1154	0.0957

649	0.1163	0.1343	0.1162	0.0966
648	0.1167	0.1343	0.1169	0.0972
647	0.1171	0.1343	0.1176	0.0979
646	0.1174	0.1342	0.1182	0.0984
645	0.1178	0.1342	0.1188	0.0990
644	0.1182	0.1342	0.1193	0.0994
643	0.1186	0.1342	0.1196	0.0997
642	0.1191	0.1342	0.1198	0.0998
641	0.1196	0.1342	0.1199	0.0997
640	0.1203	0.1343	0.1198	0.0996
639	0.1210	0.1345	0.1196	0.0994
638	0.1218	0.1347	0.1194	0.0992
637	0.1226	0.1349	0.1191	0.0988
636	0.1236	0.1352	0.1189	0.0984
635	0.1246	0.1356	0.1187	0.0979
634	0.1258	0.1361	0.1186	0.0977
633	0.1270	0.1366	0.1186	0.0974
632	0.1285	0.1372	0.1188	0.0974
631	0.1300	0.1380	0.1191	0.0973
630	0.1317	0.1387	0.1194	0.0975
629	0.1334	0.1397	0.1199	0.0978
628	0.1354	0.1407	0.1205	0.0983
627	0.1377	0.1418	0.1212	0.0989
626	0.1400	0.1431	0.1219	0.0996
625	0.1422	0.1444	0.1226	0.1003
624	0.1449	0.1458	0.1234	0.1011
623	0.1476	0.1474	0.1242	0.1016
622	0.1506	0.1491	0.1251	0.1025
621	0.1536	0.1509	0.1261	0.1034
620	0.1571	0.1529	0.1272	0.1046
619	0.1608	0.1551	0.1283	0.1060
618	0.1647	0.1574	0.1295	0.1072

617	0.1689	0.1599	0.1308	0.1086
616	0.1734	0.1626	0.1321	0.1103
615	0.1782	0.1655	0.1335	0.1120
614	0.1832	0.1684	0.1350	0.1135
613	0.1885	0.1717	0.1366	0.1153
612	0.1944	0.1752	0.1383	0.1169
611	0.2007	0.1790	0.1401	0.1189
610	0.2075	0.1830	0.1421	0.1213
609	0.2146	0.1874	0.1442	0.1235
608	0.2220	0.1920	0.1463	0.1256
607	0.2302	0.1971	0.1487	0.1285
606	0.2389	0.2023	0.1512	0.1312
605	0.2479	0.2078	0.1539	0.1339
604	0.2577	0.2137	0.1567	0.1367
603	0.2682	0.2201	0.1598	0.1398
602	0.2796	0.2269	0.1631	0.1432
601	0.2919	0.2342	0.1668	0.1469
600	0.3052	0.2421	0.1708	0.1514
599	0.3192	0.2504	0.1751	0.1557
598	0.3340	0.2593	0.1798	0.1603
597	0.3495	0.2685	0.1848	0.1655
596	0.3657	0.2782	0.1901	0.1711
595	0.3823	0.2882	0.1959	0.1771
594	0.3994	0.2985	0.2022	0.1836
593	0.4165	0.3089	0.2088	0.1903
592	0.4336	0.3194	0.2161	0.1976
591	0.4499	0.3297	0.2237	0.2054
590	0.4650	0.3394	0.2317	0.2133
589	0.4781	0.3483	0.2400	0.2216
588	0.4887	0.3561	0.2486	0.2303
587	0.4961	0.3623	0.2575	0.2392
586	0.4995	0.3667	0.2664	0.2481

585	0.4991	0.3691	0.2755	0.2571
584	0.4948	0.3695	0.2848	0.2665
583	0.4868	0.3680	0.2942	0.2757
582	0.4757	0.3647	0.3040	0.2852
581	0.4627	0.3600	0.3141	0.2953
580	0.4479	0.3543	0.3247	0.3056
579	0.4325	0.3478	0.3359	0.3169
578	0.4168	0.3408	0.3477	0.3288
577	0.4015	0.3337	0.3599	0.3412
576	0.3868	0.3268	0.3720	0.3538
575	0.3732	0.3201	0.3838	0.3663
574	0.3603	0.3135	0.3947	0.3784
573	0.3479	0.3071	0.4039	0.3892
572	0.3363	0.3009	0.4106	0.3979
571	0.3252	0.2947	0.4144	0.4037
570	0.3145	0.2884	0.4143	0.4059
569	0.3044	0.2822	0.4105	0.4042
568	0.2947	0.2761	0.4029	0.3988
567	0.2850	0.2699	0.3916	0.3892
566	0.2758	0.2636	0.3774	0.3763
565	0.2671	0.2577	0.3613	0.3608
564	0.2589	0.2519	0.3440	0.3440
563	0.2512	0.2464	0.3263	0.3261
562	0.2437	0.2411	0.3085	0.3075
561	0.2369	0.2362	0.2914	0.2894
560	0.2307	0.2318	0.2754	0.2725
559	0.2249	0.2276	0.2608	0.2564
558	0.2197	0.2239	0.2474	0.2418
557	0.2150	0.2205	0.2355	0.2286
556	0.2107	0.2175	0.2248	0.2164
555	0.2070	0.2150	0.2155	0.2059
554	0.2036	0.2128	0.2074	0.1964

553	0.2009	0.2110	0.2002	0.1882
552	0.1986	0.2096	0.1940	0.1811
551	0.1966	0.2083	0.1886	0.1742
550	0.1948	0.2073	0.1839	0.1685
549	0.1937	0.2068	0.1800	0.1636
548	0.1928	0.2065	0.1766	0.1592
547	0.1922	0.2064	0.1737	0.1553
546	0.1919	0.2065	0.1713	0.1516
545	0.1918	0.2070	0.1692	0.1487
544	0.1921	0.2076	0.1676	0.1460
543	0.1924	0.2084	0.1661	0.1433
542	0.1927	0.2094	0.1649	0.1413
541	0.1932	0.2106	0.1638	0.1396
540	0.1939	0.2121	0.1628	0.1382
539	0.1943	0.2137	0.1618	0.1369
538	0.1949	0.2154	0.1609	0.1358
537	0.1954	0.2173	0.1602	0.1351
536	0.1958	0.2195	0.1595	0.1347
535	0.1961	0.2218	0.1590	0.1344
534	0.1964	0.2244	0.1587	0.1345
533	0.1965	0.2271	0.1586	0.1350
532	0.1962	0.2300	0.1587	0.1354
531	0.1958	0.2333	0.1592	0.1363
530	0.1954	0.2369	0.1600	0.1377
529	0.1949	0.2409	0.1610	0.1391
528	0.1941	0.2453	0.1622	0.1406
527	0.1931	0.2501	0.1636	0.1423
526	0.1923	0.2555	0.1650	0.1443
525	0.1913	0.2614	0.1664	0.1462
524	0.1904	0.2680	0.1678	0.1480
523	0.1893	0.2753	0.1689	0.1495
522	0.1883	0.2834	0.1698	0.1508

521	0.1870	0.2923	0.1704	0.1517
520	0.1857	0.3021	0.1708	0.1524
519	0.1845	0.3127	0.1708	0.1527
518	0.1832	0.3242	0.1707	0.1528
517	0.1817	0.3366	0.1702	0.1526
516	0.1804	0.3495	0.1696	0.1522
515	0.1789	0.3627	0.1688	0.1514
514	0.1776	0.3756	0.1678	0.1506
513	0.1758	0.3879	0.1666	0.1494
512	0.1744	0.3986	0.1654	0.1481
511	0.1729	0.4072	0.1640	0.1466
510	0.1714	0.4128	0.1624	0.1450
509	0.1699	0.4149	0.1607	0.1435
508	0.1685	0.4131	0.1588	0.1416
507	0.1670	0.4078	0.1568	0.1396
506	0.1655	0.3994	0.1547	0.1373
505	0.1641	0.3883	0.1524	0.1352
504	0.1627	0.3753	0.1502	0.1327
503	0.1614	0.3613	0.1480	0.1305
502	0.1601	0.3471	0.1459	0.1283
501	0.1589	0.3329	0.1439	0.1266
500	0.1577	0.3194	0.1419	0.1246
499	0.1565	0.3067	0.1401	0.1226
498	0.1554	0.2951	0.1385	0.1210
497	0.1544	0.2847	0.1370	0.1198
496	0.1533	0.2753	0.1357	0.1181
495	0.1523	0.2668	0.1344	0.1167
494	0.1514	0.2594	0.1333	0.1157
493	0.1506	0.2528	0.1323	0.1148
492	0.1497	0.2470	0.1313	0.1141
491	0.1491	0.2419	0.1305	0.1135
490	0.1484	0.2374	0.1298	0.1125

489	0.1477	0.2335	0.1290	0.1121
488	0.1471	0.2301	0.1283	0.1115
487	0.1464	0.2272	0.1277	0.1107
486	0.1457	0.2248	0.1270	0.1098
485	0.1452	0.2230	0.1266	0.1095
484	0.1450	0.2217	0.1262	0.1090
483	0.1446	0.2208	0.1258	0.1083
482	0.1443	0.2203	0.1254	0.1076
481	0.1441	0.2204	0.1252	0.1070
480	0.1440	0.2210	0.1250	0.1065
479	0.1441	0.2221	0.1250	0.1064
478	0.1443	0.2237	0.1251	0.1063
477	0.1446	0.2256	0.1252	0.1060
476	0.1451	0.2280	0.1255	0.1059
475	0.1455	0.2306	0.1257	0.1057
474	0.1460	0.2334	0.1261	0.1061
473	0.1466	0.2363	0.1266	0.1061
472	0.1471	0.2390	0.1270	0.1062
471	0.1477	0.2414	0.1274	0.1064
470	0.1483	0.2435	0.1278	0.1067
469	0.1488	0.2450	0.1281	0.1065
468	0.1492	0.2461	0.1284	0.1066
467	0.1493	0.2466	0.1284	0.1065
466	0.1496	0.2466	0.1285	0.1065
465	0.1496	0.2459	0.1284	0.1063
464	0.1496	0.2448	0.1283	0.1062
463	0.1494	0.2434	0.1281	0.1060
462	0.1491	0.2415	0.1279	0.1058
461	0.1487	0.2394	0.1276	0.1056
460	0.1482	0.2372	0.1272	0.1054
459	0.1477	0.2348	0.1269	0.1051
458	0.1469	0.2322	0.1264	0.1045

457	0.1462	0.2296	0.1261	0.1041
456	0.1453	0.2270	0.1257	0.1038
455	0.1444	0.2245	0.1253	0.1033
454	0.1437	0.2222	0.1249	0.1030
453	0.1427	0.2199	0.1244	0.1029
452	0.1416	0.2176	0.1241	0.1026
451	0.1408	0.2156	0.1237	0.1022
450	0.1399	0.2137	0.1234	0.1020
449	0.1389	0.2120	0.1231	0.1018
448	0.1381	0.2105	0.1229	0.1017
447	0.1373	0.2092	0.1227	0.1015
446	0.1365	0.2081	0.1226	0.1015
445	0.1358	0.2072	0.1224	0.1014
444	0.1353	0.2063	0.1224	0.1014
443	0.1347	0.2056	0.1223	0.1013
442	0.1342	0.2051	0.1223	0.1011
441	0.1338	0.2047	0.1222	0.1009
440	0.1333	0.2043	0.1221	0.1008
439	0.1328	0.2041	0.1220	0.1003
438	0.1326	0.2040	0.1221	0.1005
437	0.1324	0.2040	0.1221	0.1005
436	0.1322	0.2040	0.1222	0.1003
435	0.1319	0.2040	0.1222	0.1001
434	0.1319	0.2041	0.1223	0.1000
433	0.1320	0.2043	0.1225	0.1003
432	0.1318	0.2045	0.1226	0.1002
431	0.1318	0.2047	0.1228	0.1000
430	0.1318	0.2049	0.1231	0.1002
429	0.1318	0.2050	0.1233	0.1001
428	0.1321	0.2053	0.1237	0.1004
427	0.1320	0.2055	0.1240	0.1003
426	0.1322	0.2059	0.1243	0.1003

425	0.1324	0.2062	0.1248	0.1004
424	0.1327	0.2066	0.1252	0.1004
423	0.1330	0.2071	0.1257	0.1006
422	0.1334	0.2077	0.1263	0.1008
421	0.1340	0.2084	0.1270	0.1012
420	0.1346	0.2092	0.1278	0.1013
419	0.1352	0.2099	0.1285	0.1017
418	0.1357	0.2108	0.1292	0.1020
417	0.1365	0.2119	0.1301	0.1023
416	0.1371	0.2128	0.1309	0.1027
415	0.1381	0.2139	0.1318	0.1032
414	0.1391	0.2152	0.1327	0.1039
413	0.1399	0.2164	0.1336	0.1044
412	0.1407	0.2176	0.1344	0.1048
411	0.1415	0.2188	0.1352	0.1052
410	0.1420	0.2199	0.1358	0.1055
409	0.1428	0.2210	0.1364	0.1058
408	0.1434	0.2222	0.1369	0.1062
407	0.1439	0.2233	0.1374	0.1065
406	0.1442	0.2244	0.1378	0.1068
405	0.1446	0.2257	0.1383	0.1072
404	0.1452	0.2269	0.1388	0.1074
403	0.1454	0.2281	0.1392	0.1074
402	0.1459	0.2293	0.1396	0.1079
401	0.1460	0.2304	0.1399	0.1079
400	0.1462	0.2317	0.1403	0.1084

Wavelength	SWNT-$(CF_2)_3CF_3$ DOC0.035	SWNT-$(CF_2)_3CF_3$ DOC0.040	SWNT-$(CF_2)_3CF_3$ DOC0.045
1300	0.0202	0.0582	0.0870
1299	0.0202	0.0586	0.0878

1298	0.0203	0.0587	0.0887
1297	0.0203	0.0590	0.0894
1296	0.0205	0.0598	0.0895
1295	0.0205	0.0602	0.0902
1294	0.0205	0.0605	0.0913
1293	0.0207	0.0613	0.0924
1292	0.0209	0.0617	0.0929
1291	0.0210	0.0621	0.0936
1290	0.0210	0.0626	0.0944
1289	0.0212	0.0632	0.0960
1288	0.0213	0.0639	0.0967
1287	0.0215	0.0643	0.0974
1286	0.0216	0.0648	0.0982
1285	0.0218	0.0650	0.0992
1284	0.0218	0.0655	0.1002
1283	0.0219	0.0660	0.1012
1282	0.0220	0.0666	0.1013
1281	0.0222	0.0668	0.1021
1280	0.0222	0.0672	0.1031
1279	0.0222	0.0675	0.1035
1278	0.0223	0.0678	0.1040
1277	0.0224	0.0683	0.1049
1276	0.0224	0.0683	0.1052
1275	0.0224	0.0685	0.1055
1274	0.0226	0.0690	0.1054
1273	0.0227	0.0695	0.1063
1272	0.0229	0.0697	0.1070
1271	0.0230	0.0702	0.1071
1270	0.0233	0.0709	0.1073
1269	0.0233	0.0711	0.1080
1268	0.0236	0.0714	0.1081
1267	0.0238	0.0720	0.1086

1266	0.0242	0.0730	0.1091
1265	0.0243	0.0734	0.1098
1264	0.0246	0.0740	0.1102
1263	0.0248	0.0745	0.1115
1262	0.0251	0.0751	0.1119
1261	0.0253	0.0758	0.1122
1260	0.0256	0.0766	0.1126
1259	0.0259	0.0774	0.1130
1258	0.0262	0.0779	0.1136
1257	0.0265	0.0784	0.1148
1256	0.0268	0.0793	0.1150
1255	0.0271	0.0803	0.1155
1254	0.0274	0.0808	0.1164
1253	0.0277	0.0816	0.1171
1252	0.0281	0.0826	0.1181
1251	0.0285	0.0836	0.1179
1250	0.0287	0.0845	0.1190
1249	0.0290	0.0854	0.1194
1248	0.0294	0.0864	0.1197
1247	0.0298	0.0878	0.1209
1246	0.0302	0.0887	0.1216
1245	0.0305	0.0897	0.1222
1244	0.0306	0.0904	0.1235
1243	0.0308	0.0915	0.1234
1242	0.0313	0.0927	0.1242
1241	0.0316	0.0938	0.1248
1240	0.0319	0.0950	0.1257
1239	0.0321	0.0959	0.1270
1238	0.0324	0.0973	0.1273
1237	0.0327	0.0985	0.1280
1236	0.0329	0.0996	0.1283
1235	0.0332	0.1009	0.1289

1234	0.0336	0.1025	0.1302
1233	0.0337	0.1039	0.1307
1232	0.0340	0.1054	0.1316
1231	0.0344	0.1070	0.1324
1230	0.0347	0.1086	0.1330
1229	0.0348	0.1098	0.1338
1228	0.0350	0.1116	0.1346
1227	0.0353	0.1134	0.1354
1226	0.0355	0.1149	0.1364
1225	0.0360	0.1169	0.1376
1224	0.0361	0.1187	0.1384
1223	0.0363	0.1207	0.1390
1222	0.0365	0.1226	0.1397
1221	0.0368	0.1250	0.1411
1220	0.0371	0.1269	0.1424
1219	0.0374	0.1292	0.1428
1218	0.0377	0.1319	0.1442
1217	0.0379	0.1340	0.1452
1216	0.0383	0.1369	0.1469
1215	0.0384	0.1395	0.1481
1214	0.0386	0.1423	0.1492
1213	0.0388	0.1449	0.1505
1212	0.0391	0.1481	0.1520
1211	0.0394	0.1512	0.1526
1210	0.0398	0.1546	0.1538
1209	0.0401	0.1578	0.1552
1208	0.0404	0.1615	0.1570
1207	0.0407	0.1652	0.1585
1206	0.0408	0.1688	0.1601
1205	0.0412	0.1729	0.1619
1204	0.0414	0.1772	0.1638
1203	0.0416	0.1814	0.1654

1202	0.0420	0.1863	0.1673
1201	0.0424	0.1914	0.1699
1200	0.0427	0.1969	0.1720
1199	0.0430	0.2022	0.1740
1198	0.0435	0.2079	0.1772
1197	0.0439	0.2140	0.1797
1196	0.0441	0.2200	0.1821
1195	0.0446	0.2266	0.1847
1194	0.0450	0.2335	0.1877
1193	0.0454	0.2407	0.1907
1192	0.0458	0.2484	0.1938
1191	0.0463	0.2565	0.1971
1190	0.0467	0.2647	0.2005
1189	0.0471	0.2729	0.2039
1188	0.0475	0.2816	0.2079
1187	0.0481	0.2910	0.2123
1186	0.0487	0.3006	0.2161
1185	0.0492	0.3104	0.2207
1184	0.0497	0.3201	0.2250
1183	0.0502	0.3297	0.2291
1182	0.0506	0.3395	0.2333
1181	0.0509	0.3492	0.2377
1180	0.0514	0.3591	0.2422
1179	0.0518	0.3685	0.2466
1178	0.0521	0.3774	0.2509
1177	0.0524	0.3867	0.2553
1176	0.0524	0.3950	0.2593
1175	0.0524	0.4033	0.2636
1174	0.0525	0.4114	0.2678
1173	0.0525	0.4192	0.2726
1172	0.0525	0.4266	0.2766
1171	0.0525	0.4343	0.2808

1170	0.0524	0.4415	0.2860
1169	0.0524	0.4492	0.2906
1168	0.0523	0.4566	0.2962
1167	0.0523	0.4639	0.3021
1166	0.0524	0.4712	0.3081
1165	0.0526	0.4783	0.3153
1164	0.0527	0.4851	0.3226
1163	0.0528	0.4917	0.3297
1162	0.0530	0.4982	0.3375
1161	0.0531	0.5039	0.3459
1160	0.0532	0.5090	0.3543
1159	0.0532	0.5135	0.3636
1158	0.0535	0.5177	0.3732
1157	0.0536	0.5203	0.3822
1156	0.0538	0.5221	0.3924
1155	0.0539	0.5231	0.4025
1154	0.0540	0.5229	0.4134
1153	0.0542	0.5213	0.4244
1152	0.0541	0.5189	0.4352
1151	0.0540	0.5152	0.4469
1150	0.0541	0.5107	0.4594
1149	0.0540	0.5048	0.4723
1148	0.0539	0.4979	0.4847
1147	0.0537	0.4901	0.4975
1146	0.0537	0.4818	0.5114
1145	0.0536	0.4726	0.5256
1144	0.0534	0.4630	0.5399
1143	0.0532	0.4528	0.5549
1142	0.0531	0.4424	0.5696
1141	0.0528	0.4318	0.5847
1140	0.0526	0.4210	0.6001
1139	0.0523	0.4102	0.6148

1138	0.0521	0.3994	0.6297
1137	0.0518	0.3887	0.6437
1136	0.0516	0.3786	0.6575
1135	0.0513	0.3687	0.6710
1134	0.0510	0.3589	0.6839
1133	0.0508	0.3492	0.6955
1132	0.0504	0.3397	0.7051
1131	0.0502	0.3309	0.7141
1130	0.0497	0.3221	0.7211
1129	0.0494	0.3138	0.7266
1128	0.0491	0.3058	0.7299
1127	0.0487	0.2979	0.7313
1126	0.0485	0.2904	0.7304
1125	0.0482	0.2829	0.7274
1124	0.0478	0.2758	0.7219
1123	0.0476	0.2691	0.7144
1122	0.0473	0.2624	0.7052
1121	0.0471	0.2562	0.6943
1120	0.0469	0.2503	0.6808
1119	0.0467	0.2443	0.6661
1118	0.0464	0.2386	0.6499
1117	0.0463	0.2330	0.6327
1116	0.0460	0.2277	0.6142
1115	0.0458	0.2224	0.5954
1114	0.0456	0.2174	0.5763
1113	0.0455	0.2129	0.5560
1112	0.0455	0.2083	0.5368
1111	0.0453	0.2040	0.5175
1110	0.0452	0.2000	0.4989
1109	0.0453	0.1960	0.4806
1108	0.0453	0.1925	0.4628
1107	0.0453	0.1893	0.4455

1106	0.0454	0.1859	0.4297
1105	0.0456	0.1828	0.4148
1104	0.0456	0.1802	0.4008
1103	0.0458	0.1779	0.3878
1102	0.0460	0.1753	0.3754
1101	0.0463	0.1732	0.3639
1100	0.0466	0.1714	0.3528
1099	0.0470	0.1696	0.3432
1098	0.0475	0.1682	0.3347
1097	0.0480	0.1670	0.3266
1096	0.0485	0.1657	0.3193
1095	0.0490	0.1649	0.3129
1094	0.0495	0.1639	0.3072
1093	0.0500	0.1630	0.3027
1092	0.0509	0.1630	0.2980
1091	0.0516	0.1625	0.2937
1090	0.0526	0.1627	0.2905
1089	0.0533	0.1627	0.2880
1088	0.0544	0.1628	0.2863
1087	0.0556	0.1632	0.2847
1086	0.0567	0.1636	0.2838
1085	0.0581	0.1644	0.2835
1084	0.0596	0.1653	0.2840
1083	0.0612	0.1663	0.2849
1082	0.0628	0.1674	0.2858
1081	0.0645	0.1687	0.2874
1080	0.0664	0.1703	0.2895
1079	0.0684	0.1718	0.2924
1078	0.0704	0.1736	0.2953
1077	0.0726	0.1755	0.2992
1076	0.0747	0.1774	0.3029
1075	0.0768	0.1795	0.3078

1074	0.0789	0.1817	0.3126
1073	0.0810	0.1841	0.3185
1072	0.0826	0.1862	0.3244
1071	0.0842	0.1882	0.3312
1070	0.0857	0.1906	0.3384
1069	0.0869	0.1929	0.3461
1068	0.0878	0.1951	0.3544
1067	0.0888	0.1975	0.3634
1066	0.0893	0.1997	0.3731
1065	0.0897	0.2020	0.3842
1064	0.0899	0.2044	0.3954
1063	0.0901	0.2072	0.4073
1062	0.0899	0.2094	0.4207
1061	0.0900	0.2117	0.4346
1060	0.0900	0.2147	0.4498
1059	0.0901	0.2175	0.4665
1058	0.0901	0.2202	0.4836
1057	0.0903	0.2232	0.5019
1056	0.0903	0.2264	0.5212
1055	0.0909	0.2299	0.5412
1054	0.0912	0.2333	0.5624
1053	0.0916	0.2365	0.5845
1052	0.0924	0.2403	0.6074
1051	0.0932	0.2443	0.6314
1050	0.0941	0.2485	0.6566
1049	0.0952	0.2526	0.6825
1048	0.0962	0.2569	0.7090
1047	0.0975	0.2616	0.7360
1046	0.0988	0.2660	0.7635
1045	0.1002	0.2703	0.7912
1044	0.1018	0.2745	0.8191
1043	0.1035	0.2791	0.8465

1042	0.1051	0.2832	0.8740
1041	0.1070	0.2870	0.8995
1040	0.1089	0.2912	0.9238
1039	0.1112	0.2949	0.9457
1038	0.1132	0.2981	0.9649
1037	0.1154	0.3010	0.9804
1036	0.1176	0.3032	0.9917
1035	0.1202	0.3053	0.9986
1034	0.1228	0.3070	1.0000
1033	0.1255	0.3085	0.9974
1032	0.1283	0.3093	0.9894
1031	0.1312	0.3098	0.9768
1030	0.1342	0.3100	0.9604
1029	0.1374	0.3099	0.9406
1028	0.1408	0.3099	0.9174
1027	0.1443	0.3098	0.8916
1026	0.1480	0.3097	0.8646
1025	0.1520	0.3099	0.8365
1024	0.1560	0.3103	0.8075
1023	0.1604	0.3108	0.7792
1022	0.1651	0.3114	0.7510
1021	0.1701	0.3124	0.7234
1020	0.1753	0.3144	0.6975
1019	0.1808	0.3162	0.6724
1018	0.1867	0.3186	0.6484
1017	0.1928	0.3217	0.6261
1016	0.1995	0.3253	0.6061
1015	0.2065	0.3294	0.5876
1014	0.2138	0.3340	0.5710
1013	0.2213	0.3390	0.5553
1012	0.2293	0.3444	0.5419
1011	0.2378	0.3507	0.5299

1010	0.2464	0.3576	0.5189
1009	0.2555	0.3647	0.5099
1008	0.2648	0.3724	0.5020
1007	0.2743	0.3804	0.4948
1006	0.2840	0.3886	0.4892
1005	0.2940	0.3971	0.4843
1004	0.3040	0.4062	0.4799
1003	0.3141	0.4153	0.4760
1002	0.3246	0.4246	0.4732
1001	0.3351	0.4343	0.4713
1000	0.3461	0.4446	0.4700
999	0.3570	0.4550	0.4683
998	0.3680	0.4650	0.4678
997	0.3793	0.4758	0.4667
996	0.3909	0.4867	0.4666
995	0.4030	0.4981	0.4673
994	0.4150	0.5095	0.4679
993	0.4274	0.5209	0.4683
992	0.4402	0.5328	0.4691
991	0.4533	0.5453	0.4696
990	0.4666	0.5578	0.4701
989	0.4802	0.5704	0.4703
988	0.4939	0.5834	0.4708
987	0.5078	0.5962	0.4711
986	0.5215	0.6088	0.4709
985	0.5354	0.6216	0.4705
984	0.5494	0.6347	0.4701
983	0.5631	0.6477	0.4691
982	0.5772	0.6609	0.4678
981	0.5918	0.6743	0.4666
980	0.6069	0.6884	0.4653
979	0.6225	0.7027	0.4636

978	0.6392	0.7180	0.4616
977	0.6571	0.7338	0.4597
976	0.6761	0.7511	0.4575
975	0.6971	0.7697	0.4560
974	0.7200	0.7892	0.4541
973	0.7447	0.8101	0.4522
972	0.7715	0.8320	0.4508
971	0.8003	0.8548	0.4499
970	0.8301	0.8784	0.4488
969	0.8611	0.9016	0.4482
968	0.8918	0.9247	0.4470
967	0.9213	0.9460	0.4463
966	0.9484	0.9648	0.4452
965	0.9710	0.9802	0.4434
964	0.9880	0.9920	0.4418
963	0.9981	0.9986	0.4399
962	1.0000	1.0000	0.4366
961	0.9933	0.9959	0.4328
960	0.9777	0.9861	0.4287
959	0.9546	0.9710	0.4239
958	0.9243	0.9511	0.4178
957	0.8885	0.9272	0.4109
956	0.8487	0.8993	0.4039
955	0.8061	0.8683	0.3963
954	0.7624	0.8357	0.3880
953	0.7183	0.8018	0.3796
952	0.6746	0.7672	0.3709
951	0.6325	0.7328	0.3620
950	0.5924	0.6991	0.3532
949	0.5544	0.6663	0.3449
948	0.5191	0.6346	0.3370
947	0.4860	0.6039	0.3288

946	0.4556	0.5758	0.3220
945	0.4278	0.5491	0.3148
944	0.4024	0.5236	0.3079
943	0.3792	0.5002	0.3020
942	0.3583	0.4786	0.2963
941	0.3394	0.4587	0.2911
940	0.3224	0.4406	0.2856
939	0.3067	0.4233	0.2811
938	0.2925	0.4073	0.2773
937	0.2798	0.3927	0.2727
936	0.2679	0.3792	0.2688
935	0.2574	0.3666	0.2652
934	0.2477	0.3550	0.2620
933	0.2392	0.3448	0.2586
932	0.2315	0.3353	0.2559
931	0.2244	0.3263	0.2538
930	0.2182	0.3186	0.2515
929	0.2126	0.3114	0.2490
928	0.2073	0.3047	0.2471
927	0.2028	0.2982	0.2458
926	0.1984	0.2926	0.2444
925	0.1944	0.2874	0.2429
924	0.1906	0.2822	0.2414
923	0.1871	0.2774	0.2410
922	0.1837	0.2724	0.2399
921	0.1804	0.2678	0.2387
920	0.1770	0.2635	0.2381
919	0.1737	0.2590	0.2362
918	0.1704	0.2548	0.2360
917	0.1673	0.2504	0.2352
916	0.1642	0.2467	0.2347
915	0.1610	0.2425	0.2335

914	0.1582	0.2387	0.2333
913	0.1556	0.2355	0.2330
912	0.1529	0.2325	0.2334
911	0.1506	0.2292	0.2335
910	0.1484	0.2263	0.2344
909	0.1465	0.2241	0.2344
908	0.1446	0.2217	0.2352
907	0.1430	0.2198	0.2365
906	0.1418	0.2185	0.2380
905	0.1405	0.2170	0.2398
904	0.1395	0.2159	0.2419
903	0.1387	0.2150	0.2440
902	0.1379	0.2141	0.2467
901	0.1374	0.2132	0.2486
900	0.1370	0.2132	0.2516
899	0.1367	0.2127	0.2540
898	0.1363	0.2123	0.2574
897	0.1362	0.2123	0.2607
896	0.1358	0.2123	0.2643
895	0.1362	0.2126	0.2670
894	0.1364	0.2129	0.2703
893	0.1363	0.2130	0.2737
892	0.1364	0.2131	0.2772
891	0.1365	0.2133	0.2804
890	0.1367	0.2137	0.2834
889	0.1368	0.2136	0.2869
888	0.1372	0.2147	0.2896
887	0.1376	0.2150	0.2929
886	0.1377	0.2151	0.2956
885	0.1382	0.2153	0.2976
884	0.1384	0.2154	0.2995
883	0.1385	0.2154	0.3004

882	0.1384	0.2157	0.3011
881	0.1387	0.2154	0.3015
880	0.1387	0.2153	0.3007
879	0.1383	0.2146	0.2993
878	0.1381	0.2140	0.2975
877	0.1376	0.2129	0.2954
876	0.1371	0.2123	0.2924
875	0.1365	0.2112	0.2886
874	0.1354	0.2095	0.2845
873	0.1345	0.2079	0.2807
872	0.1335	0.2062	0.2766
871	0.1328	0.2050	0.2724
870	0.1320	0.2036	0.2681
869	0.1310	0.2016	0.2631
868	0.1304	0.2002	0.2589
867	0.1298	0.1992	0.2542
866	0.1297	0.1981	0.2507
865	0.1297	0.1975	0.2473
864	0.1297	0.1970	0.2447
863	0.1299	0.1960	0.2407
862	0.1301	0.1961	0.2373
861	0.1307	0.1961	0.2346
860	0.1316	0.1966	0.2330
859	0.1321	0.1965	0.2304
858	0.1328	0.1964	0.2272
857	0.1339	0.1971	0.2253
856	0.1351	0.1984	0.2236
855	0.1361	0.1984	0.2213
854	0.1374	0.1990	0.2208
853	0.1391	0.2004	0.2190
852	0.1404	0.2020	0.2165
851	0.1423	0.2031	0.2151

850	0.1392	0.1981	0.2179
849	0.1414	0.1999	0.2168
848	0.1437	0.2028	0.2157
847	0.1461	0.2039	0.2149
846	0.1488	0.2060	0.2132
845	0.1510	0.2081	0.2113
844	0.1535	0.2103	0.2106
843	0.1567	0.2126	0.2099
842	0.1596	0.2151	0.2087
841	0.1626	0.2183	0.2069
840	0.1659	0.2207	0.2068
839	0.1686	0.2234	0.2054
838	0.1716	0.2262	0.2050
837	0.1746	0.2285	0.2048
836	0.1766	0.2314	0.2035
835	0.1782	0.2330	0.2030
834	0.1799	0.2345	0.2023
833	0.1812	0.2362	0.2017
832	0.1819	0.2370	0.2003
831	0.1819	0.2376	0.1999
830	0.1821	0.2382	0.1985
829	0.1815	0.2378	0.1975
828	0.1803	0.2369	0.1964
827	0.1789	0.2355	0.1945
826	0.1775	0.2344	0.1940
825	0.1757	0.2326	0.1924
824	0.1737	0.2307	0.1916
823	0.1715	0.2284	0.1906
822	0.1692	0.2259	0.1886
821	0.1671	0.2238	0.1872
820	0.1646	0.2211	0.1859
819	0.1622	0.2184	0.1854

818	0.1598	0.2155	0.1834
817	0.1577	0.2133	0.1820
816	0.1556	0.2105	0.1808
815	0.1533	0.2083	0.1795
814	0.1514	0.2062	0.1781
813	0.1494	0.2037	0.1761
812	0.1474	0.2013	0.1752
811	0.1455	0.1988	0.1750
810	0.1437	0.1969	0.1733
809	0.1421	0.1950	0.1722
808	0.1402	0.1928	0.1719
807	0.1385	0.1905	0.1716
806	0.1369	0.1889	0.1692
805	0.1354	0.1871	0.1675
804	0.1336	0.1853	0.1679
803	0.1319	0.1837	0.1673
802	0.1305	0.1820	0.1662
801	0.1290	0.1804	0.1655
800	0.1273	0.1787	0.1646
799	0.1257	0.1770	0.1644
798	0.1243	0.1753	0.1632
797	0.1229	0.1741	0.1622
796	0.1216	0.1728	0.1621
795	0.1199	0.1709	0.1619
794	0.1183	0.1692	0.1611
793	0.1170	0.1678	0.1601
792	0.1159	0.1666	0.1600
791	0.1146	0.1652	0.1605
790	0.1133	0.1641	0.1596
789	0.1122	0.1629	0.1589
788	0.1110	0.1616	0.1577
787	0.1098	0.1606	0.1581

786	0.1088	0.1595	0.1578
785	0.1075	0.1580	0.1574
784	0.1065	0.1572	0.1568
783	0.1059	0.1566	0.1570
782	0.1049	0.1558	0.1572
781	0.1039	0.1549	0.1575
780	0.1031	0.1541	0.1574
779	0.1024	0.1534	0.1570
778	0.1018	0.1529	0.1570
777	0.1011	0.1525	0.1570
776	0.1007	0.1519	0.1574
775	0.1003	0.1512	0.1579
774	0.0997	0.1505	0.1590
773	0.0995	0.1500	0.1591
772	0.0993	0.1495	0.1589
771	0.0989	0.1490	0.1596
770	0.0990	0.1488	0.1607
769	0.0989	0.1490	0.1613
768	0.0988	0.1485	0.1614
767	0.0988	0.1480	0.1620
766	0.0990	0.1481	0.1632
765	0.0989	0.1479	0.1645
764	0.0990	0.1480	0.1657
763	0.0993	0.1480	0.1670
762	0.0995	0.1479	0.1671
761	0.0996	0.1482	0.1684
760	0.0995	0.1482	0.1694
759	0.0998	0.1480	0.1710
758	0.1002	0.1480	0.1719
757	0.1005	0.1485	0.1733
756	0.1006	0.1486	0.1752
755	0.1011	0.1489	0.1769

754	0.1014	0.1491	0.1783
753	0.1017	0.1494	0.1793
752	0.1021	0.1497	0.1805
751	0.1025	0.1494	0.1820
750	0.1030	0.1499	0.1829
749	0.1036	0.1506	0.1839
748	0.1041	0.1511	0.1845
747	0.1046	0.1517	0.1845
746	0.1051	0.1519	0.1847
745	0.1058	0.1526	0.1848
744	0.1065	0.1532	0.1846
743	0.1070	0.1538	0.1844
742	0.1072	0.1540	0.1840
741	0.1076	0.1545	0.1840
740	0.1080	0.1549	0.1838
739	0.1082	0.1554	0.1834
738	0.1083	0.1557	0.1832
737	0.1081	0.1560	0.1824
736	0.1081	0.1560	0.1820
735	0.1078	0.1561	0.1819
734	0.1077	0.1564	0.1808
733	0.1076	0.1566	0.1808
732	0.1073	0.1567	0.1805
731	0.1071	0.1568	0.1806
730	0.1070	0.1569	0.1803
729	0.1069	0.1569	0.1797
728	0.1068	0.1567	0.1794
727	0.1070	0.1574	0.1792
726	0.1072	0.1576	0.1786
725	0.1075	0.1578	0.1786
724	0.1077	0.1579	0.1784
723	0.1081	0.1583	0.1776

722	0.1087	0.1590	0.1770
721	0.1091	0.1596	0.1764
720	0.1100	0.1602	0.1765
719	0.1107	0.1610	0.1768
718	0.1115	0.1621	0.1769
717	0.1125	0.1630	0.1774
716	0.1135	0.1638	0.1777
715	0.1146	0.1649	0.1777
714	0.1159	0.1664	0.1782
713	0.1173	0.1680	0.1790
712	0.1191	0.1699	0.1795
711	0.1209	0.1718	0.1802
710	0.1227	0.1739	0.1813
709	0.1248	0.1762	0.1830
708	0.1269	0.1782	0.1843
707	0.1293	0.1808	0.1850
706	0.1320	0.1837	0.1872
705	0.1347	0.1867	0.1889
704	0.1377	0.1902	0.1910
703	0.1409	0.1935	0.1923
702	0.1444	0.1969	0.1942
701	0.1480	0.2010	0.1970
700	0.1521	0.2053	0.1989
699	0.1563	0.2099	0.2009
698	0.1606	0.2142	0.2032
697	0.1654	0.2196	0.2053
696	0.1706	0.2250	0.2074
695	0.1761	0.2306	0.2099
694	0.1820	0.2367	0.2121
693	0.1882	0.2433	0.2140
692	0.1947	0.2502	0.2161
691	0.2020	0.2578	0.2191

690	0.2095	0.2654	0.2222
689	0.2173	0.2734	0.2251
688	0.2258	0.2821	0.2281
687	0.2349	0.2914	0.2315
686	0.2444	0.3014	0.2357
685	0.2546	0.3114	0.2394
684	0.2651	0.3216	0.2441
683	0.2758	0.3323	0.2482
682	0.2872	0.3433	0.2530
681	0.2989	0.3548	0.2585
680	0.3110	0.3662	0.2637
679	0.3232	0.3776	0.2690
678	0.3351	0.3889	0.2748
677	0.3468	0.3998	0.2810
676	0.3582	0.4107	0.2875
675	0.3688	0.4212	0.2951
674	0.3779	0.4302	0.3025
673	0.3855	0.4383	0.3097
672	0.3915	0.4452	0.3171
671	0.3949	0.4504	0.3252
670	0.3957	0.4534	0.3330
669	0.3937	0.4543	0.3414
668	0.3890	0.4529	0.3503
667	0.3817	0.4495	0.3600
666	0.3718	0.4439	0.3695
665	0.3599	0.4364	0.3798
664	0.3465	0.4276	0.3909
663	0.3319	0.4175	0.4022
662	0.3168	0.4069	0.4136
661	0.3018	0.3960	0.4259
660	0.2871	0.3853	0.4388
659	0.2733	0.3750	0.4514

658	0.2603	0.3653	0.4640
657	0.2487	0.3564	0.4760
656	0.2382	0.3483	0.4872
655	0.2286	0.3405	0.4975
654	0.2200	0.3335	0.5057
653	0.2127	0.3270	0.5115
652	0.2061	0.3208	0.5155
651	0.2003	0.3144	0.5159
650	0.1948	0.3085	0.5139
649	0.1902	0.3026	0.5090
648	0.1859	0.2967	0.5016
647	0.1820	0.2910	0.4915
646	0.1783	0.2851	0.4797
645	0.1750	0.2795	0.4661
644	0.1718	0.2741	0.4514
643	0.1688	0.2683	0.4365
642	0.1660	0.2629	0.4216
641	0.1634	0.2579	0.4067
640	0.1608	0.2529	0.3921
639	0.1582	0.2480	0.3782
638	0.1556	0.2434	0.3651
637	0.1532	0.2389	0.3521
636	0.1511	0.2349	0.3405
635	0.1492	0.2313	0.3300
634	0.1474	0.2280	0.3205
633	0.1456	0.2248	0.3115
632	0.1442	0.2221	0.3039
631	0.1429	0.2197	0.2974
630	0.1418	0.2175	0.2915
629	0.1407	0.2158	0.2860
628	0.1400	0.2145	0.2817
627	0.1394	0.2134	0.2779

626	0.1390	0.2125	0.2745
625	0.1387	0.2119	0.2711
624	0.1386	0.2115	0.2690
623	0.1387	0.2114	0.2672
622	0.1389	0.2113	0.2662
621	0.1394	0.2115	0.2656
620	0.1403	0.2123	0.2650
619	0.1411	0.2133	0.2647
618	0.1422	0.2145	0.2649
617	0.1434	0.2159	0.2658
616	0.1448	0.2175	0.2670
615	0.1464	0.2197	0.2681
614	0.1480	0.2215	0.2700
613	0.1499	0.2234	0.2719
612	0.1517	0.2256	0.2750
611	0.1536	0.2280	0.2778
610	0.1557	0.2306	0.2811
609	0.1576	0.2332	0.2854
608	0.1595	0.2357	0.2902
607	0.1614	0.2385	0.2957
606	0.1630	0.2412	0.3026
605	0.1644	0.2438	0.3099
604	0.1658	0.2463	0.3187
603	0.1670	0.2487	0.3280
602	0.1680	0.2512	0.3377
601	0.1692	0.2540	0.3481
600	0.1701	0.2566	0.3598
599	0.1710	0.2591	0.3712
598	0.1717	0.2612	0.3831
597	0.1727	0.2635	0.3950
596	0.1735	0.2657	0.4054
595	0.1744	0.2678	0.4145

594	0.1754	0.2695	0.4217
593	0.1763	0.2710	0.4260
592	0.1773	0.2726	0.4280
591	0.1784	0.2739	0.4281
590	0.1795	0.2747	0.4257
589	0.1807	0.2758	0.4213
588	0.1820	0.2768	0.4149
587	0.1833	0.2779	0.4076
586	0.1846	0.2788	0.4002
585	0.1859	0.2798	0.3922
584	0.1873	0.2810	0.3839
583	0.1884	0.2820	0.3758
582	0.1897	0.2830	0.3685
581	0.1912	0.2845	0.3617
580	0.1926	0.2860	0.3553
579	0.1944	0.2878	0.3492
578	0.1961	0.2899	0.3445
577	0.1979	0.2923	0.3398
576	0.1999	0.2948	0.3353
575	0.2021	0.2978	0.3314
574	0.2040	0.3009	0.3282
573	0.2056	0.3042	0.3253
572	0.2069	0.3074	0.3225
571	0.2075	0.3104	0.3189
570	0.2073	0.3128	0.3153
569	0.2063	0.3153	0.3121
568	0.2043	0.3177	0.3085
567	0.2014	0.3195	0.3046
566	0.1976	0.3213	0.3008
565	0.1935	0.3236	0.2966
564	0.1890	0.3264	0.2922
563	0.1841	0.3294	0.2885

562	0.1791	0.3330	0.2850
561	0.1744	0.3374	0.2819
560	0.1698	0.3422	0.2794
559	0.1653	0.3473	0.2770
558	0.1613	0.3525	0.2746
557	0.1575	0.3566	0.2727
556	0.1539	0.3590	0.2709
555	0.1508	0.3598	0.2687
554	0.1477	0.3578	0.2667
553	0.1451	0.3536	0.2640
552	0.1429	0.3469	0.2612
551	0.1405	0.3378	0.2582
550	0.1384	0.3277	0.2551
549	0.1366	0.3171	0.2519
548	0.1348	0.3059	0.2488
547	0.1332	0.2948	0.2459
546	0.1317	0.2844	0.2433
545	0.1305	0.2751	0.2408
544	0.1295	0.2664	0.2384
543	0.1284	0.2585	0.2367
542	0.1276	0.2518	0.2352
541	0.1268	0.2456	0.2331
540	0.1263	0.2402	0.2314
539	0.1257	0.2353	0.2301
538	0.1253	0.2312	0.2286
537	0.1250	0.2275	0.2274
536	0.1248	0.2242	0.2257
535	0.1248	0.2215	0.2240
534	0.1249	0.2196	0.2230
533	0.1250	0.2178	0.2214
532	0.1251	0.2159	0.2198
531	0.1256	0.2149	0.2189

530	0.1261	0.2143	0.2182
529	0.1268	0.2140	0.2173
528	0.1275	0.2137	0.2167
527	0.1283	0.2136	0.2154
526	0.1292	0.2141	0.2148
525	0.1302	0.2145	0.2141
524	0.1313	0.2152	0.2136
523	0.1323	0.2160	0.2133
522	0.1333	0.2171	0.2130
521	0.1343	0.2181	0.2134
520	0.1356	0.2193	0.2133
519	0.1365	0.2207	0.2131
518	0.1374	0.2225	0.2128
517	0.1384	0.2242	0.2128
516	0.1394	0.2261	0.2135
515	0.1403	0.2278	0.2135
514	0.1411	0.2298	0.2140
513	0.1417	0.2318	0.2142
512	0.1422	0.2337	0.2139
511	0.1425	0.2353	0.2146
510	0.1425	0.2363	0.2143
509	0.1422	0.2373	0.2142
508	0.1415	0.2380	0.2140
507	0.1403	0.2379	0.2131
506	0.1389	0.2374	0.2117
505	0.1374	0.2363	0.2108
504	0.1356	0.2348	0.2096
503	0.1337	0.2331	0.2082
502	0.1319	0.2310	0.2067
501	0.1300	0.2290	0.2049
500	0.1283	0.2271	0.2030
499	0.1267	0.2252	0.2017

498	0.1253	0.2230	0.2003
497	0.1242	0.2212	0.1985
496	0.1229	0.2192	0.1976
495	0.1216	0.2169	0.1964
494	0.1208	0.2152	0.1952
493	0.1200	0.2134	0.1938
492	0.1194	0.2118	0.1928
491	0.1188	0.2108	0.1921
490	0.1182	0.2096	0.1911
489	0.1176	0.2082	0.1897
488	0.1170	0.2070	0.1891
487	0.1164	0.2054	0.1884
486	0.1156	0.2039	0.1878
485	0.1149	0.2029	0.1868
484	0.1145	0.2020	0.1863
483	0.1137	0.2008	0.1855
482	0.1130	0.1997	0.1851
481	0.1125	0.1990	0.1845
480	0.1119	0.1980	0.1842
479	0.1116	0.1976	0.1837
478	0.1113	0.1974	0.1836
477	0.1112	0.1973	0.1835
476	0.1110	0.1972	0.1837
475	0.1107	0.1970	0.1834
474	0.1109	0.1973	0.1840
473	0.1110	0.1974	0.1845
472	0.1110	0.1977	0.1845
471	0.1112	0.1982	0.1843
470	0.1112	0.1983	0.1848
469	0.1111	0.1984	0.1850
468	0.1112	0.1984	0.1854
467	0.1111	0.1984	0.1847

466	0.1109	0.1984	0.1847
465	0.1107	0.1984	0.1847
464	0.1103	0.1980	0.1841
463	0.1101	0.1979	0.1839
462	0.1098	0.1976	0.1834
461	0.1095	0.1971	0.1828
460	0.1092	0.1968	0.1827
459	0.1088	0.1964	0.1820
458	0.1083	0.1958	0.1815
457	0.1077	0.1952	0.1810
456	0.1073	0.1946	0.1809
455	0.1069	0.1941	0.1803
454	0.1064	0.1934	0.1797
453	0.1060	0.1928	0.1788
452	0.1055	0.1919	0.1779
451	0.1050	0.1915	0.1769
450	0.1046	0.1908	0.1765
449	0.1041	0.1901	0.1763
448	0.1036	0.1897	0.1755
447	0.1033	0.1891	0.1750
446	0.1031	0.1889	0.1750
445	0.1027	0.1885	0.1744
444	0.1025	0.1885	0.1735
443	0.1024	0.1884	0.1732
442	0.1023	0.1883	0.1735
441	0.1021	0.1883	0.1731
440	0.1018	0.1883	0.1730
439	0.1016	0.1884	0.1728
438	0.1015	0.1891	0.1729
437	0.1014	0.1893	0.1736
436	0.1015	0.1896	0.1739
435	0.1015	0.1901	0.1742

434	0.1015	0.1909	0.1747
433	0.1015	0.1917	0.1753
432	0.1016	0.1926	0.1759
431	0.1019	0.1938	0.1763
430	0.1022	0.1951	0.1770
429	0.1023	0.1961	0.1775
428	0.1028	0.1979	0.1776
427	0.1032	0.1996	0.1781
426	0.1035	0.2011	0.1794
425	0.1039	0.2032	0.1797
424	0.1044	0.2052	0.1806
423	0.1047	0.2075	0.1817
422	0.1053	0.2101	0.1827
421	0.1060	0.2130	0.1842
420	0.1067	0.2164	0.1855
419	0.1071	0.2196	0.1867
418	0.1076	0.2229	0.1885
417	0.1084	0.2269	0.1900
416	0.1091	0.2309	0.1927
415	0.1099	0.2358	0.1948
414	0.1109	0.2411	0.1976
413	0.1116	0.2464	0.1998
412	0.1126	0.2522	0.2026
411	0.1135	0.2583	0.2059
410	0.1143	0.2636	0.2084
409	0.1152	0.2694	0.2107
408	0.1162	0.2752	0.2145
407	0.1171	0.2800	0.2176
406	0.1179	0.2843	0.2207
405	0.1186	0.2883	0.2244
404	0.1195	0.2908	0.2282
403	0.1204	0.2919	0.2313

402	0.1212	0.2926	0.2349
401	0.1221	0.2921	0.2382
400	0.1230	0.2909	0.2417

Figure 2c

Wavelength	SWNT-$(CF_2)_4$ DOC 0027	SWNT-$(CF_2)_4$ DOC 0035	SWNT-$(CF_2)_3CF_3$ DOC 0027	SWNT-$(CF_2)_3CF_3$ DOC 0029
700	7.036	-0.412	1.829	-3.174
699.5	8.384	0.233	2.581	-0.612
699	7.637	-0.932	2.560	0.175
698.5	5.515	-1.881	2.226	-1.549
698	5.300	-1.632	2.857	-3.008
697.5	5.440	-2.133	2.487	-3.180
697	7.837	-1.179	2.762	-1.659
696.5	8.406	-0.437	2.872	-2.418
696	8.253	-0.554	2.646	-3.440
695.5	7.332	-1.073	2.911	-2.925
695	6.577	-1.759	2.735	-2.844
694.5	7.447	-1.166	2.393	-1.485
694	6.857	-1.947	2.794	-2.901
693.5	7.921	-1.822	2.563	-4.183
693	7.387	-1.510	2.512	-3.911
692.5	6.181	-1.871	1.975	-4.502
692	6.828	-1.806	2.504	-4.440
691.5	7.088	-1.468	3.093	-4.315
691	7.279	-0.822	3.246	-0.444
690.5	7.584	-1.940	3.498	-3.551
690	8.253	-1.451	3.723	-4.930
689.5	8.550	-1.110	4.088	-2.549
689	7.321	-1.716	3.898	-4.156
688.5	6.322	-2.026	3.923	-3.739
688	7.754	-2.305	3.913	-1.756
687.5	9.644	-1.230	3.344	-2.080
687	8.095	-1.262	4.103	-4.083
686.5	7.334	-0.982	4.840	-3.146
686	7.138	-1.568	4.172	-4.497

685.5	8.491	-1.827	4.244	-5.847
685	8.245	-2.542	4.393	-5.226
684.5	7.955	-2.056	4.828	-4.891
684	7.808	-0.646	5.001	-5.203
683.5	8.257	-0.696	5.020	-5.582
683	8.903	-0.720	5.272	-4.953
682.5	10.247	-1.511	5.873	-1.762
682	9.822	-1.626	5.892	-2.286
681.5	10.429	-0.780	6.102	-2.852
681	11.639	-0.022	6.996	-1.300
680.5	10.962	-0.373	6.648	-2.150
680	10.008	-1.301	6.238	-3.866
679.5	10.480	-1.190	7.090	-1.865
679	9.471	-2.367	7.058	-3.666
678.5	8.697	-2.380	5.925	-6.492
678	9.112	-1.412	6.570	-6.740
677.5	11.254	-0.570	7.392	-4.215
677	10.882	-1.598	6.331	-6.176
676.5	10.138	-1.952	6.709	-8.273
676	10.355	-1.928	7.529	-5.561
675.5	10.875	-2.200	7.144	-4.966
675	10.862	-2.298	7.049	-4.907
674.5	11.201	-2.657	7.159	-5.440
674	10.538	-2.696	7.112	-6.650
673.5	10.271	-2.477	7.957	-7.233
673	10.036	-2.224	8.815	-7.645
672.5	10.926	-3.101	7.911	-7.674
672	13.163	-2.677	8.371	-6.955
671.5	12.697	-2.862	8.609	-9.956
671	14.149	-2.859	8.885	-6.662
670.5	13.743	-3.273	9.304	-5.750
670	11.070	-4.129	8.237	-10.771

669.5	12.142	-4.330	8.661	-12.355
669	14.109	-3.771	8.894	-10.470
668.5	14.009	-3.777	8.884	-12.688
668	14.612	-4.053	9.318	-11.001
667.5	13.764	-4.108	9.962	-8.850
667	14.352	-3.983	10.305	-9.978
666.5	16.424	-5.073	10.810	-9.024
666	17.353	-5.183	10.914	-10.027
665.5	15.858	-5.658	10.739	-12.294
665	15.451	-6.018	10.953	-12.484
664.5	14.673	-6.664	11.161	-13.104
664	16.264	-6.709	11.797	-13.503
663.5	17.527	-6.866	12.076	-11.565
663	17.392	-7.589	10.864	-13.260
662.5	18.298	-7.726	10.827	-12.274
662	18.446	-8.280	10.645	-13.777
661.5	18.893	-8.429	11.276	-13.166
661	20.392	-8.373	11.881	-13.667
660.5	20.688	-8.207	12.693	-14.262
660	20.001	-8.229	12.753	-13.030
659.5	19.351	-9.217	12.139	-16.590
659	20.961	-9.442	12.565	-15.823
658.5	22.659	-9.370	12.902	-15.206
658	24.235	-9.845	12.864	-14.185
657.5	23.907	-9.967	13.277	-15.453
657	24.089	-10.333	13.140	-14.156
656.5	25.439	-11.283	12.651	-15.843
656	25.844	-11.804	12.623	-14.416
655.5	24.178	-13.122	11.896	-16.831
655	25.193	-12.821	11.872	-17.196
654.5	24.990	-12.967	11.998	-16.750
654	25.846	-13.427	12.294	-16.338

653.5	27.890	-13.419	11.856	-17.839
653	27.859	-14.211	11.113	-19.655
652.5	27.411	-14.066	12.061	-18.295
652	26.839	-15.174	11.932	-16.904
651.5	27.838	-15.344	11.472	-15.128
651	28.910	-14.987	10.797	-15.660
650.5	29.894	-15.520	11.130	-18.358
650	30.246	-16.494	11.788	-18.335
649.5	29.599	-17.199	10.704	-16.386
649	29.180	-17.568	9.830	-16.881
648.5	27.669	-18.225	9.576	-17.888
648	27.439	-17.726	9.069	-18.149
647.5	30.358	-17.186	9.346	-15.213
647	30.545	-18.331	8.854	-14.730
646.5	28.842	-19.068	8.484	-12.776
646	27.803	-18.069	7.673	-12.908
645.5	29.937	-16.529	7.404	-12.444
645	29.027	-16.778	6.977	-13.600
644.5	27.653	-17.097	6.461	-14.879
644	26.747	-16.625	5.841	-12.786
643.5	26.725	-16.106	5.835	-11.064
643	27.029	-15.966	6.222	-8.611
642.5	25.231	-15.936	5.047	-8.626
642	24.356	-15.575	4.481	-8.886
641.5	24.561	-14.628	4.833	-7.887
641	23.371	-13.801	4.665	-6.988
640.5	20.348	-13.585	3.869	-6.983
640	19.751	-13.018	3.685	-5.635
639.5	18.542	-13.008	3.084	-7.172
639	16.802	-12.436	3.111	-8.667
638.5	16.834	-10.935	2.881	-5.790
638	16.543	-9.969	2.693	-4.431

637.5	14.116	-9.975	2.064	-5.361
637	12.994	-9.238	1.955	-6.216
636.5	12.280	-7.673	2.576	-5.872
636	12.492	-6.326	2.440	-3.550
635.5	11.372	-5.598	2.487	-0.907
635	11.089	-6.726	2.008	-5.886
634.5	10.341	-6.497	2.304	-5.592
634	9.747	-5.988	2.047	-4.356
633.5	10.456	-5.498	2.181	-2.484
633	11.435	-3.814	2.431	-0.350
632.5	10.897	-4.330	2.147	-0.899
632	9.725	-4.656	1.660	-3.335
631.5	11.276	-3.799	2.742	-2.052
631	11.427	-3.480	3.620	-3.932
630.5	11.358	-3.909	3.029	-4.424
630	9.591	-4.789	2.886	-3.782
629.5	9.264	-4.447	2.569	-4.493
629	8.813	-4.550	1.601	-5.602
628.5	8.620	-5.182	1.476	-3.694
628	9.959	-5.515	1.700	-3.685
627.5	8.777	-5.533	1.497	-5.670
627	8.515	-4.932	1.323	-5.167
626.5	9.444	-4.436	1.477	-3.053
626	8.822	-4.538	1.073	-5.378
625.5	9.981	-4.519	1.627	-6.562
625	11.219	-3.659	2.643	-3.856
624.5	11.246	-3.783	2.268	-2.471
624	11.345	-3.958	1.698	-2.899
623.5	10.722	-4.386	1.971	-2.334
623	10.366	-4.699	2.043	-3.567
622.5	11.186	-4.377	1.285	-4.668
622	11.254	-4.425	1.644	-3.723

621.5	10.486	-4.110	2.282	-2.604
621	10.959	-3.353	2.217	-0.592
620.5	11.218	-3.150	1.280	-2.538
620	10.121	-3.438	1.170	-3.785
619.5	9.454	-3.343	1.322	-1.702
619	9.575	-4.084	0.583	-3.176
618.5	9.248	-5.228	0.463	-2.693
618	9.668	-4.085	0.935	0.204
617.5	8.398	-3.298	0.555	-1.388
617	9.142	-3.611	-0.347	-1.100
616.5	8.565	-3.571	-0.402	-0.136
616	8.980	-3.472	0.063	1.786
615.5	7.391	-3.090	-0.970	-1.455
615	8.234	-3.122	-1.230	0.190
614.5	8.895	-3.163	-0.769	0.558
614	7.466	-3.802	-0.655	0.087
613.5	8.173	-2.569	-0.375	1.304
613	6.646	-3.141	-2.083	-0.828
612.5	7.455	-2.855	-1.979	-0.162
612	5.646	-2.564	-1.964	0.775
611.5	5.450	-2.111	-1.900	3.845
611	7.148	-0.859	-1.822	4.716
610.5	4.618	-1.070	-2.430	3.973
610	3.824	-1.100	-3.052	3.442
609.5	3.790	-0.922	-3.234	3.438
609	2.511	-1.557	-4.014	2.766
608.5	0.465	-1.657	-4.202	3.700
608	0.306	-1.129	-4.947	2.756
607.5	1.230	-0.102	-5.151	2.376
607	2.237	1.369	-5.351	3.408
606.5	1.529	1.188	-5.740	6.509
606	0.472	1.444	-5.507	7.711

605.5	-0.654	1.873	-6.058	6.928
605	-1.822	1.283	-7.439	7.411
604.5	-4.253	1.110	-7.962	8.175
604	-3.843	2.016	-7.592	8.115
603.5	-2.049	3.089	-7.057	10.535
603	-3.585	2.790	-8.080	9.220
602.5	-5.865	3.022	-7.966	9.632
602	-7.170	3.498	-8.420	9.699
601.5	-7.419	3.771	-8.325	11.419
601	-7.525	4.968	-7.980	11.216
600.5	-7.772	4.545	-8.586	10.798
600	-8.684	4.636	-9.475	9.994
599.5	-8.740	5.580	-10.050	12.265
599	-8.855	5.953	-10.131	13.397
598.5	-11.681	6.277	-10.381	12.649
598	-12.722	6.270	-10.340	13.363
597.5	-11.237	7.337	-10.830	15.149
597	-11.324	7.991	-11.154	16.503
596.5	-12.815	8.323	-11.471	17.258
596	-13.745	8.721	-12.206	16.429
595.5	-15.285	8.865	-13.074	17.616
595	-15.814	9.325	-13.432	16.346
594.5	-16.612	9.871	-13.359	16.552
594	-18.809	9.900	-14.616	16.697
593.5	-20.630	9.616	-14.798	17.534
593	-20.631	10.540	-15.165	18.788
592.5	-21.265	12.224	-15.433	18.316
592	-21.223	13.335	-15.259	19.933
591.5	-21.533	12.926	-15.244	21.103
591	-23.331	13.306	-16.086	21.555
590.5	-24.251	14.330	-16.208	24.965
590	-26.303	14.902	-16.892	25.821

589.5	-27.362	15.696	-17.933	24.979
589	-27.160	16.823	-18.181	24.057
588.5	-28.188	17.254	-18.113	26.101
588	-28.915	17.188	-18.499	27.461
587.5	-30.092	17.903	-19.040	28.810
587	-30.565	18.636	-19.100	30.998
586.5	-32.874	19.648	-19.776	30.915
586	-34.536	20.283	-20.952	31.459
585.5	-34.104	21.345	-20.723	32.896
585	-33.624	22.153	-21.098	33.629
584.5	-36.961	21.611	-22.694	31.461
584	-39.338	21.887	-22.826	32.578
583.5	-39.455	22.964	-23.728	33.516
583	-40.276	23.946	-24.329	34.711
582.5	-41.840	25.464	-25.326	36.893
582	-43.610	26.372	-25.017	36.522
581.5	-44.486	26.860	-25.738	35.860
581	-44.258	28.624	-25.508	36.301
580.5	-46.752	29.743	-27.272	36.172
580	-49.498	31.066	-28.332	38.924
579.5	-49.889	31.802	-27.720	40.928
579	-50.865	32.988	-28.560	42.137
578.5	-54.205	33.590	-29.903	41.372
578	-56.184	34.973	-29.794	43.993
577.5	-58.037	36.019	-29.833	46.351
577	-60.434	36.829	-29.856	45.122
576.5	-62.809	38.387	-30.363	46.652
576	-64.051	39.994	-30.917	46.606
575.5	-65.047	40.777	-31.506	46.520
575	-66.325	43.051	-32.072	47.146
574.5	-67.289	45.104	-31.438	49.231
574	-68.912	46.355	-30.979	52.720

573.5	-70.463	45.645	-30.945	52.350
573	-72.472	46.533	-30.763	49.599
572.5	-71.719	47.395	-31.030	49.071
572	-71.552	47.845	-30.150	49.804
571.5	-72.911	48.034	-30.359	50.648
571	-72.248	49.564	-30.515	49.987
570.5	-71.909	50.469	-30.354	48.530
570	-71.802	50.107	-30.076	48.258
569.5	-71.682	50.486	-29.413	47.549
569	-73.069	50.757	-28.564	45.853
568.5	-71.093	50.679	-27.155	47.035
568	-69.997	48.591	-27.147	45.035
567.5	-69.113	47.681	-27.101	43.993
567	-66.498	48.293	-25.860	44.531
566.5	-64.728	46.564	-25.510	42.836
566	-62.885	45.746	-24.624	40.383
565.5	-61.270	45.232	-23.084	39.169
565	-59.481	43.892	-22.619	38.391
564.5	-57.567	42.624	-22.355	36.654
564	-55.745	42.080	-21.446	36.220
563.5	-53.660	41.275	-19.821	36.303
563	-51.085	39.106	-19.131	32.515
562.5	-49.618	36.675	-18.824	28.713
562	-47.189	34.906	-18.502	29.191
561.5	-44.336	34.850	-17.452	30.742
561	-41.211	33.411	-16.035	29.515
560.5	-39.032	31.909	-14.413	26.071
560	-37.080	30.571	-14.183	25.873
559.5	-36.876	28.562	-14.401	24.579
559	-34.792	26.843	-13.269	25.353
558.5	-32.587	25.737	-12.847	23.119
558	-30.537	23.911	-12.256	19.830

557.5	-28.888	23.020	-11.406	18.350
557	-26.485	21.650	-11.038	18.317
556.5	-24.801	19.639	-10.723	16.919
556	-24.138	19.156	-10.156	16.581
555.5	-22.767	18.468	-10.468	14.781
555	-21.005	17.635	-9.936	14.772
554.5	-20.632	16.734	-9.764	14.801
554	-19.795	16.585	-8.416	15.417
553.5	-19.517	16.392	-8.620	13.845
553	-19.450	15.258	-9.160	10.378
552.5	-19.839	13.687	-8.336	9.420
552	-18.169	12.878	-7.847	10.939
551.5	-16.366	13.840	-7.062	12.547
551	-16.828	13.393	-7.242	9.913
550.5	-14.773	12.375	-7.236	12.284
550	-13.370	11.885	-7.090	13.784
549.5	-13.048	11.609	-7.598	11.878
549	-12.980	10.756	-7.099	8.681
548.5	-12.793	10.662	-6.680	10.184
548	-12.230	10.699	-6.800	10.938
547.5	-12.535	9.805	-6.180	10.879
547	-12.723	10.098	-6.151	11.007
546.5	-12.873	9.980	-6.880	10.753
546	-12.220	9.806	-6.659	9.728
545.5	-12.047	9.545	-6.458	8.406
545	-11.916	9.139	-7.313	8.325
544.5	-11.642	9.011	-7.559	10.426
544	-11.556	9.224	-7.286	9.985
543.5	-11.371	9.236	-6.317	11.204
543	-12.261	8.585	-6.704	8.636
542.5	-10.909	8.965	-6.842	8.589
542	-11.132	8.675	-6.913	10.225

541.5	-11.820	8.678	-6.767	10.987
541	-11.402	9.152	-6.614	9.750
540.5	-11.560	9.588	-6.820	8.953
540	-12.573	9.134	-7.002	11.210
539.5	-12.125	8.474	-7.393	9.443
539	-10.946	9.343	-7.278	10.473
538.5	-12.004	8.745	-7.454	10.345
538	-13.229	8.038	-7.961	8.815
537.5	-11.610	9.348	-7.546	10.630
537	-12.254	9.167	-7.730	10.883
536.5	-11.650	9.496	-7.070	11.460
536	-12.411	9.220	-7.629	10.060
535.5	-13.548	9.492	-7.978	11.792
535	-13.657	9.896	-8.357	11.598
534.5	-13.146	9.814	-8.518	12.908
534	-13.300	9.531	-8.053	12.958
533.5	-13.648	9.841	-7.978	12.992
533	-14.232	9.777	-8.862	11.989
532.5	-14.341	10.223	-8.980	12.608
532	-14.708	10.365	-9.332	12.273
531.5	-15.808	9.777	-10.090	12.338
531	-15.558	10.242	-9.989	12.781
530.5	-15.778	11.118	-9.761	13.574
530	-14.857	12.218	-9.905	15.482
529.5	-15.414	12.176	-9.732	15.462
529	-16.469	11.822	-10.189	15.976
528.5	-16.140	12.036	-10.455	17.332
528	-16.950	12.199	-10.610	16.157
527.5	-17.549	12.879	-10.630	15.897
527	-17.848	12.720	-10.882	14.221
526.5	-18.447	13.041	-10.815	14.608
526	-18.164	13.297	-10.589	16.379

525.5	-17.689	14.112	-10.817	18.178
525	-19.458	13.610	-11.367	16.528
524.5	-19.604	13.661	-11.721	14.400
524	-20.112	14.031	-11.731	15.476
523.5	-21.405	13.399	-12.089	15.014
523	-20.577	14.189	-12.273	14.869
522.5	-20.625	14.543	-12.206	16.167
522	-19.666	15.010	-11.760	17.540
521.5	-21.084	15.305	-11.268	17.233
521	-21.209	15.320	-10.995	16.818
520.5	-20.834	14.329	-12.199	14.873
520	-21.474	13.931	-11.699	15.100
519.5	-22.230	14.184	-11.929	16.678
519	-22.513	14.684	-12.483	16.073
518.5	-21.681	15.231	-11.794	17.537
518	-21.222	15.469	-11.469	18.500
517.5	-21.852	14.606	-11.917	16.462
517	-21.790	14.636	-11.820	16.672
516.5	-21.662	15.188	-12.209	17.365
516	-20.756	14.482	-12.435	17.140
515.5	-21.025	14.449	-12.211	18.337
515	-20.998	15.139	-12.313	18.231
514.5	-21.421	14.913	-12.949	16.994
514	-20.767	15.377	-12.514	16.135
513.5	-19.572	15.170	-11.912	16.084
513	-20.207	14.950	-12.573	16.333
512.5	-20.852	15.364	-11.997	18.525
512	-21.580	14.855	-12.545	15.498
511.5	-21.837	14.423	-12.973	15.234
511	-21.448	14.366	-13.327	17.423
510.5	-21.189	14.757	-13.282	18.044
510	-22.449	14.236	-13.148	15.729

509.5	-22.077	14.604	-12.824	17.696
509	-21.459	15.296	-12.423	18.734
508.5	-22.248	14.587	-12.629	16.806
508	-22.089	14.469	-12.756	17.586
507.5	-21.051	14.790	-12.596	18.967
507	-21.404	14.952	-12.849	19.665
506.5	-22.773	14.741	-13.104	17.251
506	-22.692	14.596	-12.657	16.773
505.5	-22.930	14.840	-12.934	17.028
505	-22.161	14.912	-12.994	18.436
504.5	-22.461	14.468	-12.959	19.154
504	-23.036	15.009	-13.862	19.360
503.5	-22.251	15.467	-13.873	20.270
503	-22.699	15.378	-13.518	19.417
502.5	-23.012	15.076	-13.783	18.424
502	-23.598	14.607	-14.150	17.938
501.5	-22.618	15.139	-13.654	18.817
501	-22.793	15.430	-13.389	19.176
500.5	-23.920	14.830	-13.940	17.638
500	-23.889	15.329	-14.334	19.926
499.5	-24.106	15.297	-14.207	20.227
499	-23.768	15.598	-13.867	20.534
498.5	-23.189	15.969	-14.042	20.697
498	-24.286	15.674	-14.607	19.694
497.5	-24.035	15.471	-14.094	20.361
497	-25.437	15.115	-14.538	18.577
496.5	-25.428	15.850	-14.319	19.574
496	-24.715	16.656	-14.283	20.288
495.5	-25.431	15.949	-14.374	18.313
495	-26.153	15.856	-14.125	18.804
494.5	-25.619	15.928	-14.404	19.374
494	-24.571	16.265	-14.238	19.790

493.5	-24.962	15.809	-14.085	18.230
493	-25.608	15.460	-14.644	18.638
492.5	-25.413	15.394	-13.867	18.426
492	-24.493	15.699	-13.395	18.206
491.5	-24.349	15.810	-13.017	19.761
491	-25.079	15.375	-13.344	19.367
490.5	-24.087	15.203	-13.774	19.194
490	-24.625	15.402	-13.299	18.081
489.5	-24.471	15.305	-12.843	19.040
489	-24.636	14.987	-12.828	19.462
488.5	-24.975	15.048	-13.258	18.702
488	-24.495	15.238	-12.372	18.394
487.5	-24.465	14.688	-12.389	17.801
487	-24.514	14.660	-13.149	17.744
486.5	-24.112	14.516	-12.841	17.648
486	-24.033	14.114	-12.379	16.469
485.5	-23.420	14.266	-12.600	16.111
485	-24.200	13.914	-13.048	15.234
484.5	-24.204	13.980	-12.834	14.567
484	-23.381	14.107	-12.207	16.648
483.5	-22.841	14.139	-11.750	17.067
483	-21.866	13.736	-11.862	15.323
482.5	-22.412	13.235	-11.783	14.443
482	-22.274	13.795	-11.617	16.014
481.5	-20.808	14.146	-11.077	17.710
481	-21.057	13.735	-11.048	15.828
480.5	-21.400	13.326	-11.191	15.006
480	-21.437	12.779	-11.484	14.693
479.5	-20.943	12.813	-11.182	14.435
479	-20.325	13.138	-10.599	14.304
478.5	-19.278	13.379	-10.525	16.660
478	-19.420	13.061	-10.605	16.184

477.5	-20.361	12.410	-10.351	13.293
477	-20.145	12.340	-10.487	12.941
476.5	-19.612	12.159	-10.611	12.756
476	-19.352	11.897	-11.072	13.000
475.5	-19.765	11.496	-10.935	12.446
475	-19.855	11.541	-10.454	11.179
474.5	-19.462	12.017	-10.598	11.645
474	-18.164	12.685	-10.098	14.028
473.5	-17.038	12.768	-9.497	15.729
473	-17.001	12.042	-9.587	15.401
472.5	-18.398	11.587	-10.050	14.016
472	-19.222	11.530	-10.398	13.188
471.5	-18.408	11.585	-10.590	12.369
471	-18.217	11.127	-10.714	12.163
470.5	-18.016	11.612	-10.231	12.637
470	-17.727	12.030	-10.048	12.872
469.5	-17.268	12.010	-9.869	14.643
469	-17.356	11.747	-10.407	13.489
468.5	-18.308	11.522	-10.654	12.616
468	-17.290	12.383	-10.148	14.345
467.5	-17.439	11.913	-9.908	14.123
467	-17.781	11.349	-10.499	14.603
466.5	-16.726	11.893	-10.220	15.058
466	-17.632	11.536	-10.996	12.607
465.5	-18.243	11.126	-11.337	12.170
465	-18.042	11.603	-11.003	14.501
464.5	-16.924	11.582	-11.430	15.470
464	-16.856	11.875	-11.400	15.064
463.5	-18.202	12.224	-11.190	15.181
463	-17.777	12.262	-11.064	14.889
462.5	-16.079	12.937	-10.805	16.894
462	-17.149	12.493	-11.601	17.389

461.5	-18.198	12.201	-12.231	17.717
461	-17.887	12.562	-12.640	16.772
460.5	-18.070	13.395	-13.094	15.982
460	-19.059	13.644	-13.288	17.113
459.5	-20.630	13.545	-13.975	17.674
459	-20.683	13.775	-14.491	18.124
458.5	-20.215	13.843	-15.186	18.971
458	-21.572	13.956	-15.555	18.529
457.5	-22.781	14.320	-15.853	20.045
457	-23.175	14.815	-16.608	20.671
456.5	-23.383	15.454	-16.989	21.181
456	-24.353	16.265	-17.548	22.944
455.5	-25.673	16.845	-18.588	22.512
455	-27.076	17.158	-19.080	23.532
454.5	-27.559	17.920	-19.383	25.233
454	-29.218	18.417	-20.204	26.055
453.5	-30.644	19.333	-21.057	26.419
453	-30.429	20.864	-20.826	28.805
452.5	-31.867	21.488	-21.086	30.859
452	-34.623	21.813	-21.998	29.811
451.5	-37.769	22.271	-22.666	28.462
451	-37.181	23.540	-22.684	30.318
450.5	-36.994	24.956	-22.718	32.798
450	-38.527	26.049	-22.719	35.630
449.5	-39.774	26.910	-23.100	35.487
449	-41.091	27.602	-23.338	33.981
448.5	-42.634	28.587	-23.679	33.318
448	-43.562	29.229	-23.926	34.453
447.5	-44.746	29.377	-24.226	34.669
447	-45.388	29.506	-24.224	34.268
446.5	-45.724	30.656	-23.940	34.655
446	-44.876	31.805	-23.360	35.120

445.5	-45.498	31.918	-22.616	34.707
445	-46.208	31.781	-22.563	34.149
444.5	-45.999	31.504	-22.345	34.685
444	-45.126	31.832	-22.019	34.849
443.5	-44.911	31.421	-21.485	34.098
443	-44.570	31.201	-21.127	34.063
442.5	-43.577	31.215	-20.443	34.646
442	-43.585	30.153	-21.286	30.670
441.5	-43.492	29.849	-20.993	30.579
441	-42.785	29.520	-19.783	29.930
440.5	-41.839	28.857	-19.087	30.378
440	-40.369	28.891	-18.670	30.486
439.5	-39.300	28.497	-17.582	29.548
439	-38.851	27.591	-17.829	28.620
438.5	-38.962	26.650	-17.559	28.300
438	-37.549	26.334	-17.282	27.782
437.5	-36.585	25.719	-17.390	26.796
437	-35.769	25.592	-16.886	25.599
436.5	-35.710	25.405	-15.997	25.389
436	-35.252	25.440	-15.560	26.894
435.5	-35.054	24.734	-15.816	26.770
435	-33.910	24.512	-15.558	25.574
434.5	-32.261	24.676	-14.971	26.397
434	-32.831	24.325	-14.840	26.261
433.5	-32.835	24.071	-14.327	24.757
433	-32.290	23.686	-13.857	24.633
432.5	-32.231	23.583	-13.670	24.422
432	-32.080	23.328	-13.905	22.978
431.5	-31.586	23.149	-14.095	23.343
431	-31.553	23.182	-14.082	22.634
430.5	-31.289	22.866	-13.796	22.775
430	-31.244	23.035	-13.246	22.974

429.5	-31.157	23.026	-13.015	23.066
429	-30.605	22.411	-12.860	22.219
428.5	-30.302	22.743	-12.479	21.138
428	-30.261	22.356	-12.644	21.749
427.5	-29.627	22.065	-12.258	21.337
427	-29.674	21.895	-12.179	20.445
426.5	-29.025	21.961	-11.496	21.354
426	-29.141	21.585	-11.355	20.718
425.5	-29.573	21.275	-11.987	18.260
425	-28.898	21.320	-11.790	18.238
424.5	-28.306	20.895	-11.226	19.181
424	-27.338	20.822	-10.857	20.395
423.5	-26.556	20.746	-10.683	19.745
423	-25.918	20.556	-10.228	19.170
422.5	-25.740	20.373	-9.947	17.984
422	-26.794	19.286	-10.301	16.260
421.5	-26.399	18.996	-9.909	16.806
421	-25.376	18.986	-9.589	16.087
420.5	-24.757	18.718	-9.839	16.339
420	-24.682	18.379	-9.994	16.043
419.5	-24.723	17.897	-9.773	14.499
419	-23.642	18.301	-9.136	14.455
418.5	-23.033	17.941	-8.831	13.364
418	-22.927	17.456	-8.920	14.228
417.5	-22.690	16.930	-9.049	14.100
417	-22.468	16.633	-9.082	14.735
416.5	-22.212	16.370	-8.832	14.962
416	-21.222	16.092	-9.147	13.572
415.5	-19.940	16.258	-8.783	14.343
415	-19.749	16.039	-8.084	15.527
414.5	-20.540	15.313	-8.221	14.176
414	-21.239	14.726	-8.102	12.928

413.5	-20.815	15.060	-8.299	13.788
413	-19.363	14.738	-8.573	14.856
412.5	-20.080	14.214	-8.252	13.151
412	-20.306	14.354	-8.385	12.334
411.5	-19.675	13.825	-8.034	12.471
411	-19.650	13.820	-7.790	12.188
410.5	-18.249	13.946	-7.623	13.448
410	-18.605	13.659	-7.539	14.575
409.5	-19.916	13.143	-8.071	12.521
409	-19.070	12.791	-8.089	12.137
408.5	-17.849	12.937	-8.066	12.732
408	-17.889	13.224	-8.007	12.935
407.5	-18.362	12.854	-7.827	11.499
407	-18.064	13.059	-7.373	11.742
406.5	-17.688	13.364	-7.320	13.353
406	-17.926	13.158	-7.160	13.189
405.5	-18.311	12.989	-7.308	13.222
405	-18.245	13.034	-7.367	13.391
404.5	-18.221	12.896	-6.800	13.089
404	-18.022	13.287	-6.864	12.963
403.5	-18.187	13.165	-7.145	11.939
403	-18.188	12.710	-6.643	11.737
402.5	-18.611	12.547	-6.721	10.956
402	-18.418	12.607	-6.123	11.912
401.5	-18.848	12.784	-5.777	12.518
401	-18.959	12.861	-5.958	12.175
400.5	-18.439	13.218	-5.829	10.088
400	-17.878	12.685	-5.909	9.785
399.5	-19.248	12.268	-5.777	9.852
399	-18.966	12.179	-5.581	9.548
398.5	-18.243	12.066	-5.333	9.327
398	-18.057	11.852	-5.033	9.178

397.5	-17.278	12.200	-4.402	9.668
397	-17.309	11.502	-4.167	8.790
396.5	-17.371	11.317	-3.658	7.340
396	-16.971	11.575	-2.970	7.384
395.5	-16.675	10.811	-2.337	7.464
395	-17.090	9.841	-2.836	5.795
394.5	-16.498	10.047	-2.950	5.913
394	-15.018	10.048	-2.924	7.303
393.5	-15.179	9.202	-2.323	4.756
393	-15.156	8.881	-2.006	2.929
392.5	-14.046	8.884	-1.726	4.152
392	-13.478	8.156	-1.378	4.110
391.5	-13.690	7.527	-0.952	2.879
391	-13.116	7.470	-0.912	2.542
390.5	-12.237	6.991	-0.403	2.633
390	-11.184	6.807	-0.283	3.203
389.5	-10.244	6.728	-0.014	4.247
389	-10.074	6.373	0.489	2.563
388.5	-9.819	5.493	1.131	0.922
388	-8.712	4.551	1.534	0.467
387.5	-7.996	4.383	2.179	0.889
387	-8.190	3.858	2.595	-0.275
386.5	-7.171	3.365	2.335	-0.930
386	-6.705	2.932	2.966	-2.687
385.5	-5.523	2.391	3.617	-3.092
385	-4.645	1.498	3.936	-3.022
384.5	-4.921	0.969	4.312	-3.602
384	-4.440	0.723	4.469	-5.791
383.5	-2.474	-0.121	4.724	-5.432
383	-0.960	-0.338	5.529	-5.138
382.5	-0.040	-0.423	5.974	-6.072
382	0.380	-0.833	6.239	-5.760

381.5	0.056	-1.719	6.389	-7.105
381	0.245	-2.761	7.014	-9.126
380.5	1.476	-3.396	6.936	-9.579
380	2.984	-3.622	7.826	-8.990
379.5	3.605	-4.673	8.844	-10.778
379	4.288	-5.630	9.698	-13.450
378.5	5.621	-5.804	10.515	-13.183
378	6.477	-6.209	10.525	-12.650
377.5	8.136	-6.906	11.129	-12.663
377	8.676	-7.873	11.447	-14.520
376.5	9.322	-8.584	11.833	-17.002
376	11.086	-9.020	12.529	-16.944
375.5	12.254	-9.843	13.078	-16.945
375	13.240	-10.195	13.629	-16.188
374.5	14.418	-10.633	14.425	-17.687
374	15.318	-11.528	14.180	-20.089
373.5	15.960	-12.646	14.786	-19.836
373	17.349	-13.425	15.869	-20.249
372.5	18.591	-13.733	16.154	-22.258
372	19.891	-14.448	16.944	-20.910
371.5	20.836	-15.515	17.535	-21.834
371	21.407	-16.447	18.558	-24.845
370.5	23.208	-16.661	19.207	-25.315
370	25.513	-17.402	20.025	-24.948
369.5	26.431	-18.701	20.482	-26.320
369	27.346	-19.504	20.865	-27.153
368.5	28.066	-20.744	21.395	-29.536
368	29.438	-21.132	22.492	-31.270
367.5	30.856	-21.600	22.975	-31.814
367	32.408	-22.625	23.792	-32.592
366.5	33.825	-23.506	24.788	-32.499
366	34.681	-24.530	25.237	-35.256

365.5	35.837	-25.742	25.376	-36.003
365	37.757	-26.520	26.847	-35.181
364.5	39.365	-27.111	27.553	-36.071
364	41.230	-28.389	27.935	-38.890
363.5	42.789	-29.176	29.576	-40.143
363	43.498	-30.447	30.751	-40.762
362.5	45.891	-31.396	30.854	-41.024
362	48.181	-31.958	32.037	-41.536
361.5	50.326	-33.051	32.938	-43.630
361	52.718	-34.369	34.088	-45.426
360.5	53.796	-35.861	35.242	-47.337
360	56.687	-36.766	36.793	-49.017
359.5	59.893	-37.981	38.565	-49.168
359	62.261	-40.125	39.512	-52.532
358.5	64.533	-42.013	40.166	-55.087
358	67.765	-44.047	42.315	-55.852
357.5	70.117	-46.306	44.106	-59.469
357	73.497	-48.771	45.110	-62.245
356.5	77.429	-51.058	47.040	-64.200
356	81.324	-53.557	49.546	-67.218
355.5	85.942	-56.023	51.301	-70.044
355	90.787	-59.151	52.816	-73.910
354.5	95.915	-62.140	55.182	-76.008
354	100.430	-65.516	56.929	-77.625
353.5	105.634	-69.141	59.209	-81.538
353	110.013	-72.405	61.324	-85.148
352.5	115.470	-75.567	63.015	-89.023
352	120.422	-78.907	64.140	-91.856
351.5	124.948	-82.242	65.252	-93.574
351	128.636	-85.523	66.394	-95.563
350.5	132.915	-89.088	67.532	-97.769
350	136.644	-92.056	68.214	-99.941

349.5	139.409	-94.501	68.385	-102.800
349	142.337	-95.979	68.860	-104.627
348.5	144.149	-97.771	69.242	-104.567
348	144.889	-99.417	69.015	-104.799
347.5	145.542	-100.690	68.229	-104.278
347	145.643	-100.837	67.181	-104.100
346.5	144.855	-100.982	65.873	-104.024
346	143.165	-101.176	64.390	-102.518
345.5	140.672	-100.328	62.206	-100.640
345	138.327	-98.645	60.117	-97.726
344.5	135.865	-96.978	59.240	-94.342
344	133.725	-95.156	57.846	-92.220
343.5	129.775	-93.101	55.786	-91.562
343	126.618	-90.596	53.718	-88.259
342.5	123.264	-88.449	51.994	-85.815
342	120.087	-85.689	50.412	-82.528
341.5	116.299	-83.536	48.720	-79.012
341	113.383	-81.721	47.323	-77.170
340.5	110.496	-79.674	45.903	-75.311
340	106.923	-77.538	44.335	-73.945
339.5	104.542	-74.983	42.421	-71.515
339	101.403	-73.166	40.900	-68.703
338.5	98.154	-71.322	39.433	-67.802
338	95.636	-69.404	38.240	-64.522
337.5	93.450	-67.797	36.702	-62.383
337	91.073	-65.825	35.319	-61.355
336.5	89.256	-64.060	34.938	-58.869
336	86.305	-62.687	33.615	-58.322
335.5	83.832	-61.333	31.662	-56.852
335	82.293	-59.842	30.207	-53.711
334.5	80.534	-57.914	29.901	-50.954
334	77.863	-56.816	28.451	-49.149

333.5	74.739	-55.690	25.962	-48.026
333	72.778	-53.833	23.977	-48.371
332.5	70.170	-52.340	23.738	-48.097
332	68.012	-50.633	23.863	-44.716
331.5	66.153	-48.684	22.635	-42.680
331	63.971	-47.298	20.843	-41.636
330.5	61.334	-45.957	19.932	-40.467
330	59.677	-44.528	19.348	-37.469
329.5	57.426	-42.981	18.282	-36.170
329	55.505	-41.199	16.907	-33.105
328.5	53.406	-39.327	15.499	-31.058
328	51.558	-37.515	13.924	-29.383
327.5	49.044	-35.565	13.595	-28.787
327	46.471	-34.386	12.644	-26.962
326.5	44.780	-33.371	10.912	-24.928
326	42.700	-31.520	10.197	-23.014
325.5	40.739	-29.500	8.729	-21.863
325	38.808	-27.577	6.997	-19.671
324.5	36.776	-26.546	5.757	-19.012
324	34.189	-25.096	4.726	-16.892
323.5	32.103	-22.972	2.584	-14.923
323	29.253	-21.562	1.796	-13.924
322.5	27.647	-19.745	0.467	-10.563
322	25.480	-18.133	-1.233	-8.694
321.5	23.325	-16.669	-1.764	-5.536
321	20.342	-14.581	-3.548	-4.139
320.5	17.122	-12.250	-4.877	-3.026
320	14.576	-10.245	-5.967	-0.282
319.5	11.793	-8.463	-8.727	1.558
319	8.700	-6.110	-10.332	3.727
318.5	5.750	-3.798	-11.591	6.772
318	2.345	-1.590	-14.018	9.985

317.5	-0.554	1.162	-15.100	12.711
317	-4.267	3.791	-16.709	15.903
316.5	-8.582	5.693	-19.262	18.409
316	-11.709	8.255	-20.989	21.714
315.5	-15.464	11.273	-21.864	24.759
315	-20.117	14.083	-24.403	27.436
314.5	-24.027	17.441	-27.091	30.329
314	-28.724	20.206	-29.020	32.527
313.5	-33.112	23.105	-30.466	35.810
313	-37.539	26.258	-32.753	40.101
312.5	-42.210	29.374	-34.643	43.421
312	-46.575	33.062	-36.603	46.274
311.5	-50.921	36.735	-38.755	50.391
311	-56.347	39.979	-39.474	51.946
310.5	-61.514	43.333	-41.351	55.273
310	-65.601	46.543	-43.432	59.407
309.5	-69.340	49.899	-45.328	61.548
309	-74.038	52.940	-46.323	64.540
308.5	-78.326	55.920	-47.006	65.492
308	-82.563	59.057	-47.088	66.716
307.5	-86.155	61.754	-47.826	69.111
307	-88.688	64.415	-47.756	71.155
306.5	-91.695	66.447	-48.346	71.756
306	-93.927	68.331	-48.432	73.596
305.5	-95.497	69.791	-48.960	73.010
305	-97.111	70.481	-47.717	72.290
304.5	-97.604	70.717	-45.776	71.360
304	-97.376	71.240	-45.847	71.415
303.5	-95.920	70.649	-45.034	69.013
303	-94.338	68.665	-43.327	64.638
302.5	-92.136	67.536	-40.665	63.940
302	-89.560	65.557	-38.920	60.593

301.5	-85.531	62.927	-36.234	57.110
301	-81.615	60.013	-32.984	54.781
300.5	-77.162	56.896	-30.525	50.812
300	-72.310	53.658	-28.469	48.167
299.5	-67.503	50.411	-25.424	44.068
299	-62.433	46.536	-23.391	39.077
298.5	-57.586	42.888	-22.102	35.177
298	-52.047	38.900	-18.522	31.835
297.5	-47.292	34.817	-15.657	27.367
297	-42.117	32.098	-14.318	23.232
296.5	-36.676	28.602	-11.196	21.086
296	-31.924	23.944	-8.460	18.710
295.5	-27.236	20.287	-6.445	15.191
295	-22.278	17.415	-5.059	12.076
294.5	-17.938	14.558	-2.100	10.609
294	-13.805	11.789	0.396	9.332
293.5	-9.372	8.578	2.021	5.397
293	-5.926	5.891	2.744	1.445
292.5	-2.032	3.815	3.973	0.854
292	0.947	1.502	6.297	-1.911
291.5	3.496	-1.036	7.797	-4.021
291	6.280	-3.173	9.602	-5.796
290.5	8.843	-5.318	9.670	-8.066
290	11.731	-6.971	9.671	-8.056
289.5	14.108	-8.000	10.502	-8.817
289	15.413	-9.242	11.116	-9.081
288.5	16.064	-10.423	11.741	-10.199
288	16.616	-10.800	11.704	-10.305
287.5	17.068	-11.092	11.016	-7.917
287	16.302	-11.773	10.298	-8.117
286.5	16.287	-11.304	10.039	-9.908
286	15.282	-10.222	9.500	-8.690

285.5	13.377	-8.587	8.711	-5.270
285	10.404	-7.178	7.395	-3.448
284.5	7.819	-5.543	5.150	-2.605
284	5.140	-3.881	1.814	-0.267
283.5	1.243	-1.597	-1.636	1.961
283	-1.742	1.308	-2.583	3.243
282.5	-5.238	3.758	-4.354	6.934
282	-9.442	6.278	-5.060	9.830
281.5	-12.617	9.313	-6.944	11.931
281	-15.814	11.705	-9.082	15.923
280.5	-19.941	14.189	-10.337	16.344
280	-23.045	16.293	-11.934	19.678
279.5	-25.352	19.218	-13.842	22.914
279	-27.987	21.701	-15.280	24.872
278.5	-30.189	23.374	-15.801	26.274
278	-32.328	24.716	-16.168	25.961
277.5	-34.515	25.867	-18.538	26.396
277	-35.198	27.230	-17.534	26.145
276.5	-35.906	28.382	-17.021	29.138
276	-37.331	29.169	-16.555	29.306
275.5	-38.259	29.691	-17.391	26.966
275	-38.944	30.063	-18.404	28.943
274.5	-38.639	30.689	-15.894	28.694
274	-39.222	30.322	-15.706	26.555
273.5	-38.877	30.093	-15.906	26.608
273	-38.046	30.235	-15.609	27.202
272.5	-38.092	29.548	-15.797	26.198
272	-37.584	29.174	-15.803	26.181
271.5	-37.342	28.519	-14.815	25.301
271	-36.313	28.137	-14.051	23.918
270.5	-35.343	27.617	-14.772	23.165
270	-34.554	27.052	-12.952	22.915

269.5	-32.456	26.158	-11.686	23.494
269	-31.986	24.906	-13.011	21.584
268.5	-31.472	23.994	-11.655	21.026
268	-30.265	23.468	-12.403	20.266
267.5	-29.471	23.184	-9.528	18.859
267	-28.006	22.001	-6.924	20.203
266.5	-28.051	20.324	-7.565	17.764
266	-27.622	19.298	-8.692	16.502
265.5	-25.522	18.727	-8.989	15.206
265	-23.669	18.161	-8.869	14.691
264.5	-23.369	17.126	-7.744	15.635
264	-22.563	16.144	-5.191	15.102
263.5	-22.378	15.228	-3.074	13.085
263	-22.202	13.961	-4.915	10.033
262.5	-22.471	13.290	-5.703	9.059
262	-21.149	13.227	-2.707	10.443
261.5	-20.496	12.260	-0.474	9.739
261	-20.341	10.888	0.529	8.533
260.5	-18.738	10.023	3.617	7.630
260	-17.460	9.227	1.559	7.842
259.5	-16.941	7.906	4.333	7.476
259	-15.628	6.629	3.866	3.663
258.5	-13.994	5.986	2.175	1.810
258	-12.624	4.487	6.440	0.312
257.5	-10.411	3.195	7.189	-1.034
257	-8.707	1.779	5.706	-2.389
256.5	-6.665	0.181	9.131	-4.635
256	-3.897	-1.534	10.234	-7.798
255.5	-1.287	-3.059	8.128	-8.899
255	1.504	-4.480	9.557	-10.420
254.5	3.798	-6.541	10.174	-11.794
254	5.888	-8.072	13.150	-12.193

253.5	8.677	-9.334	12.370	-12.335
253	11.265	-11.105	19.109	-14.982
252.5	14.698	-12.830	22.251	-18.262
252	16.700	-14.761	16.237	-21.763
251.5	18.679	-15.985	16.382	-22.283
251	21.253	-17.414	20.537	-21.542
250.5	23.557	-18.717	16.729	-22.682
250	24.882	-19.398	16.688	-24.967
