# Supplementary material for: Selective emergence of photoluminescence at telecommunication wavelengths from cyclic perfluoroalkylated carbon nanotubes
Source: Commun Chem. 2023 Jul 31;6:159. doi: 10.1038/s42004-023-00950-1 (PMC10390534; doi:10.1038/s42004-023-00950-1)
Supplement: Supplementary file 5 — Supplementary Data 2 [file 42004_2023_950_MOESM5_ESM.docx]

**List of compounds**

(6,5) SWNT>(CF_2_)_4_ (1,2-L_++_: L_87_)

(6,5) SWNT>(CF_2_)_4_ (1,2-L_+_: L_27_)

(6,5) SWNT>(CF_2_)_4_ (1,2- L–: L_–33_)

(6,5) SWNT>(CF_2_)_4_ (1,4-L_++_: L_87_)

(6,5) SWNT>(CF_2_)_4_ (1,4-L_+_: L_27_)

(6,5) SWNT>(CF_2_)_4_ (1,4- L–: L_–33_)

(6,5) SWNT-[(CF_2_)_3_CF_3_]_2_ (1,2-L_++_: L_87_)

(6,5) SWNT-[(CF_2_)_3_CF_3_]_2_ (1,2-L_+_: L_27_)

(6,5) SWNT-[(CF_2_)_3_CF_3_]_2_ (1,2-L–: L_–33_)

(6,5) SWNT-[(CF_2_)_3_CF_3_]_2_ (1,4-L_++_: L_87_)

(6,5) SWNT-[(CF_2_)_3_CF_3_]_2_ (1,4-L_+_: L_27_)

(6,5) SWNT-[(CF_2_)_3_CF_3_]_2_ (1,4-L–: L_–33_)

(6,5) SWNT-[(CH_2_)_3_CH_3_]_2_ (1,2-L_++_: L_87_)

(6,5) SWNT-[(CH_2_)_3_CH_3_]_2_ (1,2-L_+_: L_27_)

(6,5) SWNT-[(CH_2_)_3_CH_3_]_2_ (1,2-L–: L_–33_)

(6,5) SWNT-[(CH_2_)_3_CH_3_]_2_ (1,4-L_++_: L_87_)

(6,5) SWNT-[(CH_2_)_3_CH_3_]_2_ (1,4-L_+_: L_27_)

(6,5) SWNT-[(CH_2_)_3_CH_3_]_2_ (1,4-L–: L_–33_)

CF_3_(CF_2_)_3_-(6,5) SWNT-H (1,2-L_++_: L_87_)

CF_3_(CF_2_)_3_-(6,5) SWNT-H (1,2-L_+_: L_27_)

CF_3_(CF_2_)_3_-(6,5) SWNT-H (1,2-L–: L_–33_)

CF_3_(CF_2_)_3_-(6,5) SWNT-H (1,4-L_++_: L_87_)

CF_3_(CF_2_)_3_-(6,5) SWNT-H (1,4-L_+_: L_27_)

CF_3_(CF_2_)_3_-(6,5) SWNT-H (1,4-L–: L_–33_)

CF_3_(CF_2_)_2_CH_2_-(6,5) SWNT-H (1,2-L_++_: L_87_)

CF_3_(CF_2_)_2_CH_2_-(6,5) SWNT-H (1,2-L_+_: L_27_)

CF_3_(CF_2_)_2_CH_2_-(6,5) SWNT-H (1,2-L–: L_–33_)

CF_3_(CF_2_)_2_CH_2_-(6,5) SWNT-H (1,4-L_++_: L_87_)

CF_3_(CF_2_)_2_CH_2_-(6,5) SWNT-H (1,4-L_+_: L_27_)

CF_3_(CF_2_)_2_CH_2_-(6,5) SWNT-H (1,4-L–: L_–33_)

CF_3_CF_2_(CH_2_)_2_-(6,5) SWNT-H (1,2-L_++_: L_87_)

CF_3_CF_2_(CH_2_)_2_-(6,5) SWNT-H (1,2-L_+_: L_27_)

CF_3_CF_2_(CH_2_)_2_-(6,5) SWNT-H (1,2-L–: L_–33_)

CF_3_CF_2_(CH_2_)_2_-(6,5) SWNT-H (1,4-L_++_: L_87_)

CF_3_CF_2_(CH_2_)_2_-(6,5) SWNT-H (1,4-L_+_: L_27_)

CF_3_CF_2_(CH_2_)_2_-(6,5) SWNT-H (1,4-L–: L_–33_

CH_3_(CH_2_)_3_-(6,5) SWNT-H (1,2-L_++_: L_87_)

CH_3_(CH_2_)_3_-(6,5) SWNT-H (1,2-L_+_: L_27_)

CH_3_(CH_2_)_3_-(6,5) SWNT-H (1,2-L–: L_–33_)

CH_3_(CH_2_)_3_-(6,5) SWNT-H (1,4-L_++_: L_87_)

CH_3_(CH_2_)_3_-(6,5) SWNT-H (1,4-L_+_: L_27_)

CH_3_(CH_2_)_3_-(6,5) SWNT-H (1,4-L–: L_–33_)

**(6,5) SWNT>(CF_2_)_4_ (1,2-L_++_: L_87_)**

C -20.31163700 -1.45412300 -3.46433800

C -19.12574600 -0.66979200 -3.68976800

C -19.16900400 0.72598700 -3.81192900

C -18.01314700 1.50299500 -3.59399500

C -17.86865200 -1.30734500 -3.45711700

C -16.67456000 -0.57175800 -3.68441500

C -15.40565600 -1.21672700 -3.50973600

C -14.20688800 -0.48207200 -3.72803300

C -19.28520000 3.31773100 -2.37033600

C -18.06501200 2.75682900 -2.84979100

C -16.87347600 3.20625100 -2.22500900

C -16.74187900 0.84971000 -3.71290500

C -15.53164400 1.58192300 -3.49237900

C -14.26850300 0.94551300 -3.69149200

C -13.06929800 1.67262000 -3.44172300

C -12.94417500 -1.12905700 -3.56623100

C -11.74037600 -0.37936800 -3.74338600

C -10.47714800 -1.03206700 -3.60501100

C -9.27579800 -0.27645300 -3.74875200

C -19.51678200 3.57921800 1.83522300

C -19.35306900 3.91031400 -1.13069800

C -18.20390900 3.96529400 -0.28679100

C -16.94303600 3.80283500 -0.92340800

C -15.73968700 3.88649500 -0.14996100

C -15.59487400 2.76444600 -2.71028900

C -14.40035500 3.25960300 -2.11580700

C -13.13504700 2.83698300 -2.62083200

C -11.93677800 3.30686700 -2.00041100

C -11.80181800 1.04172700 -3.65410400

C -10.60271200 1.76414400 -3.38097900

C -9.33780800 1.14188800 -3.61090200

C -8.13778200 1.85564100 -3.30993500

C -8.00999500 -0.93299500 -3.62751500

C -6.80845500 -0.17209600 -3.74273500

C -5.54468400 -0.83118300 -3.63490800

C -4.34167100 -0.06616300 -3.71375100

C -3.01759000 -2.04789200 -3.08306500

C -0.54945400 -1.96712700 -3.12243000

C -19.59611500 2.71267100 2.89772900

C -18.44756900 1.97963400 3.33058300

C -18.28157200 3.78444200 1.14711400

C -17.09811600 3.41076800 1.83869400

C -15.81738200 3.66484000 1.24759100

C -14.62618200 3.33227800 1.96020500

C -14.47084100 3.81273000 -0.79882000

C -13.28159000 3.88613000 -0.02217600

C -12.00939900 3.81813200 -0.67269000

C -10.81735500 3.87074500 0.10911200

C -10.66977300 2.90281400 -2.52135800

C -9.47557500 3.35390000 -1.88737500

C -8.20651800 2.96535600 -2.41788500

C -7.01204500 3.40266900 -1.77138500

C -6.87143600 1.24259800 -3.55955400

C -5.67469600 1.94765900 -3.23582100

C -4.40656100 1.34041700 -3.49583500

C -3.21028500 2.03947400 -3.15482700

C -3.07709700 -0.72639000 -3.61293800

C -1.87946000 0.03707900 -3.67185100

C -0.61483800 -0.63228900 -3.58981300

C 0.58264700 0.15073900 -3.66032900

C 1.91526000 -1.85509100 -3.19298800

C 4.38121800 -1.75525800 -3.31626200

C -19.77040100 -0.09526400 3.89243700

C -18.53108100 0.61173600 3.78509200

C -17.34893400 -0.17674700 3.75330300

C -17.18061500 2.48586400 2.93015300

C -15.98212000 1.81984700 3.34621300

C -14.70750300 2.37202700 3.01382200

C -13.52141500 1.69590400 3.41750400

C -13.35856800 3.61929900 1.37902100

C -12.16583900 3.24820200 2.07336800

C -10.89437300 3.55695800 1.50057500

C -9.70498200 3.16433600 2.18082200

C -9.54911800 3.82519000 -0.54252300

C -8.35588000 3.85081500 0.24082300

C -7.08604400 3.83202900 -0.41224300

C -5.89444200 3.83587900 0.37091100

C -5.74428600 3.03329700 -2.31302500

C -4.54979300 3.45237400 -1.65208300

C -3.28012600 3.10230300 -2.20522300

C -2.08693800 3.50248800 -1.53388400

C -1.94472700 1.43870000 -3.42762500

C -0.74917300 2.13196900 -3.07163900

C 0.51679800 1.54155900 -3.37392800

C 1.71485500 2.22598700 -3.01008600

C 1.84555600 -0.50423100 -3.63969400

C 3.04263400 0.27145800 -3.69242900

C 4.30987000 -0.38701100 -3.70201000

C 5.50857700 0.39030800 -3.71280700

C 6.84337900 -1.65321600 -3.39627600

C 9.30891200 -1.54657500 -3.45909700

C -20.02688700 -3.48477100 1.41358000

C -19.85290900 -1.42315900 3.55899900

C -18.70326900 -2.12716100 3.07713900

C -17.43507800 -1.55698700 3.37762200

C -16.23817600 -2.21802600 2.95302800

C -16.06639800 0.46138600 3.73906100

C -14.87642900 -0.32939400 3.74525900

C -13.60525900 0.31315500 3.76688100

C -12.41508100 -0.47692300 3.72786200

C -12.24642700 2.25346900 3.08985700

C -11.05586500 1.56035900 3.46888600

C -9.78470600 2.13060700 3.16282000

C -8.59353600 1.42273600 3.51019100

C -8.43310600 3.49058800 1.61767900

C -7.24112900 3.07622600 2.28513400

C -5.97142700 3.42666500 1.73578600

C -4.77874500 2.98601800 2.38499700

C -4.62393300 3.83879500 -0.28230500

C -3.42997300 3.81891800 0.50131300

C -2.16016500 3.84549100 -0.15125200

C -0.96535100 3.79112400 0.62977100

C -0.81751600 3.16424800 -2.09667300

C 0.38101300 3.54098000 -1.41373800

C 1.64807700 3.22452900 -1.99289100

C 2.84520300 3.56967500 -1.29795000

C 2.97792000 1.65151200 -3.34174700

C 4.17873800 2.31550500 -2.94467600

C 5.44329800 1.75500000 -3.30062700

C 6.64231100 2.39967500 -2.87348000

C 6.77366500 -0.26943800 -3.73954900

C 7.97312800 0.50646700 -3.71203400

C 9.24006900 -0.15260300 -3.75933700

C 10.43809800 0.62038900 -3.70317500

C 10.57675900 -2.13043400 -3.15702500

C 11.77241700 -1.43891500 -3.51401700

C 13.04223100 -2.03432300 -3.22904000

C 14.23781200 -1.33586300 -3.56407600

C -20.10396900 -3.71554100 0.06581300

C -18.94713800 -3.56436700 -0.76868800

C -18.78611100 -3.10434700 2.02201600

C -17.60178700 -3.40905700 1.29697700

C -16.32177100 -3.14058700 1.88098100

C -15.12911800 -3.47828100 1.16987400

C -14.96206500 -1.68836800 3.32010100

C -13.77646500 -2.34439800 2.88290600

C -12.50074800 -1.81831700 3.25640100

C -11.31134600 -2.46055000 2.79348600

C -11.13925300 0.16680800 3.76810700

C -9.95168400 -0.62056600 3.70232600

C -8.67657500 0.02147100 3.75782000

C -7.48600400 -0.76451500 3.66243700

C -7.32035600 2.00619400 3.22617400

C -6.13036300 1.28764100 3.54643400

C -4.85826600 1.88086400 3.28116700

C -3.66579900 1.14827800 3.56908300

C -3.50702800 3.35971800 1.84963700

C -2.31497100 2.89524100 2.48056100

C -1.04339700 3.28369400 1.95759000

C 0.15016200 2.78992800 2.57125200

C 0.30789300 3.83529800 -0.02259100

C 1.50260200 3.75245800 0.75608100

C 2.77279600 3.81362600 0.10581100

C 3.96636200 3.69650400 0.88147900

C 4.11234200 3.26897000 -1.88721400

C 5.30979900 3.58736600 -1.17809400

C 6.57580400 3.31106700 -1.77742400

C 7.77208600 3.60162400 -1.05558400

C 7.90761800 1.85259000 -3.24756800

C 9.10732600 2.48100900 -2.79353100

C 10.37271700 1.95071600 -3.18831600

C 11.57041300 2.55981900 -2.70793200

C 11.70416100 -0.03784300 -3.76798000

C 12.90406700 0.73325300 -3.68108800

C 14.16943200 0.07730500 -3.76859000

C 15.36498900 0.83840000 -3.64055000

C 15.50376900 -1.93836500 -3.29121400

C 16.71011800 -1.23213700 -3.59958000

C 17.98438200 -1.86055300 -3.40433600

C 19.13621600 -1.11037300 -3.71760600

C -20.25763700 -2.57066700 -2.68000500

C -19.01717400 -2.99777300 -2.08554200

C -17.81683900 -2.49373700 -2.66138100

C -17.68069800 -3.62765100 -0.11755700

C -16.47838800 -3.52439300 -0.88508000

C -15.20645300 -3.64988600 -0.24262300

C -14.01486300 -3.53206700 -1.01395300

C -13.85997200 -3.23940300 1.77257900

C -12.66902800 -3.54063600 1.04544200

C -11.39573000 -3.31781600 1.65609900

C -10.20715800 -3.60037800 0.92190400

C -10.03751000 -1.94966700 3.18546800

C -8.84967700 -2.57449300 2.69970700

C -7.57238500 -2.07547900 3.10511500

C -6.38592900 -2.68804000 2.60224000

C -6.21298600 -0.12459500 3.73980700

C -5.02366900 -0.90530100 3.60598900

C -3.74789800 -0.26874200 3.70011600

C -2.56037100 -1.04124500 3.52595600

C -2.39518900 1.75134200 3.32962200

C -1.20548500 1.00392800 3.58030900

C 0.06830700 1.61859300 3.37224300

C 1.25540200 0.86444300 3.61384400

C 1.42415300 3.19773600 2.06777800

C 2.61238600 2.68254600 2.66605600

C 3.88674700 3.10050900 2.17392900

C 5.07574100 2.56769400 2.75874000

C 5.23717900 3.77980900 0.23368500

C 6.42882400 3.63908100 1.00533200

C 7.69888600 3.74410600 0.36123400

C 8.89131400 3.57994000 1.12896800

C 9.03981500 3.34917000 -1.66406500

C 10.23513200 3.61683100 -0.93271600

C 11.50208000 3.38633400 -1.54837100

C 12.69837100 3.62860600 -0.80586900

C 12.83850400 2.04209200 -3.11912200

C 14.03555100 2.63463700 -2.61803600

C 15.30084900 2.12583900 -3.03822400

C 16.50003900 2.69811600 -2.50946300

C 16.63625700 0.18254000 -3.74109100

C 17.82723600 0.94524700 -3.60476900

C 19.08668100 0.28973500 -3.76338400

C 20.26936000 1.10045000 -3.63528800

C -16.54647800 -2.92726400 -2.16824900

C -15.34593100 -2.42548900 -2.76167300

C -14.08205800 -2.88849500 -2.28774200

C -12.88359400 -2.36382500 -2.85615200

C -12.74581800 -3.66625600 -0.37169600

C -11.55021400 -3.52012900 -1.13964100

C -10.28349200 -3.68056600 -0.50231200

C -9.08940700 -3.50984900 -1.26240400

C -8.93529700 -3.39656200 1.53780700

C -7.74542700 -3.66008100 0.79416600

C -6.47309900 -3.48244500 1.41616600

C -5.28879800 -3.72879100 0.66581500

C -5.11123000 -2.19449700 3.00875600

C -3.92133500 -2.78773700 2.47389900

C -2.64654400 -2.30408100 2.87223100

C -1.46101200 -2.89723400 2.32047400

C -1.28848000 -0.41334100 3.64621900

C -0.09965900 -1.18971800 3.45172600

C 1.17288400 -0.55707200 3.63536200

C 2.36095900 -1.31622700 3.45475700

C 2.52878400 1.48448300 3.43628500

C 3.71642600 0.72647000 3.66174000

C 4.99218500 1.35118000 3.49847500

C 6.18077500 0.58965300 3.70591700

C 6.34900900 3.00252100 2.28039400

C 7.53770700 2.45495100 2.84874600

C 8.81157100 2.90513400 2.38262200

C 10.00145100 2.34473000 2.93639800

C 10.16157700 3.71082200 0.48993800

C 11.35256200 3.52352700 1.25135200

C 12.62412300 3.67462900 0.61612600

C 13.81707900 3.46619500 1.37212000

C 13.96717800 3.42331200 -1.42917800

C 15.15976500 3.63608200 -0.67945900

C 16.43132400 3.44040900 -1.30436700

C 17.63444100 3.63666900 -0.55651600

C 17.77143000 2.21617300 -2.95253500

C 18.97057700 2.78814800 -2.44127000

C 20.21173900 2.30061900 -2.98607800

C -11.61661700 -2.83719900 -2.38984400

C -10.41748200 -2.29540900 -2.94026200

C -9.15386300 -2.78316000 -2.48805400

C -7.95122800 -2.21894500 -3.01375800

C -7.82173000 -3.69450400 -0.62936800

C -6.62666600 -3.50560000 -1.38299500

C -5.36481400 -3.71881400 -0.75668900

C -4.16398400 -3.49603800 -1.49521600

C -4.01092500 -3.56254900 1.28435500

C -2.82307100 -3.84003200 0.54751200

C -1.56500900 -3.70002500 1.17145100

C -0.34529400 -4.31554500 0.50038600

C -0.17987500 -2.43379400 2.78004400

C 1.01771100 -3.00657600 2.23854500

C 2.28501600 -2.53812700 2.72479600

C 3.48333000 -3.08591300 2.19327600

C 3.63393400 -0.69592200 3.65583500

C 4.82760200 -1.45310400 3.45681700

C 6.09997800 -0.83495800 3.66424900

C 7.29205500 -1.58291100 3.43013300

C 7.45472300 1.21844500 3.55489500

C 8.64549800 0.45112400 3.73677200

C 9.91951600 1.08580900 3.60554200

C 11.10901100 0.31467000 3.76160500

C 11.27304500 2.80955400 2.48395100

C 12.46431500 2.23025500 3.01844300

C 13.73790000 2.71150700 2.58235900

C 14.92576400 2.11614700 3.09389800

C 15.08489400 3.63413500 0.74313300

C 16.27952300 3.38737300 1.48757300

C 17.55807200 3.58698900 0.87377000

C 18.74427300 3.37459600 1.62820000

C 18.90036200 3.50173600 -1.19794000

C 20.05799300 3.75141200 -0.38882800

C -6.68831200 -2.72663000 -2.58041700

C -5.48745600 -2.13779000 -3.07029500

C -4.22229000 -2.65765300 -2.64207400

C -2.89895100 -3.78230800 -0.89932400

C -1.71685100 -3.53957400 -1.62681800

C -0.41847300 -4.18680100 -1.16569800

C 0.81507400 -3.49661600 -1.73556800

C 0.96715600 -3.77462800 1.05536000

C 2.14626700 -3.90346200 0.29712200

C 3.41550700 -3.73134900 0.92889100

C 4.61299400 -3.85303100 0.16337500

C 4.75441500 -2.65458400 2.69536600

C 5.94823300 -3.17500100 2.11785000

C 7.21727400 -2.75668300 2.62250400

C 8.41210600 -3.24693000 2.01348600

C 8.56543100 -0.96953600 3.64990100

C 9.75836300 -1.70896700 3.38776200

C 11.02917900 -1.10408400 3.62667300

C 12.22204400 -1.83271300 3.33157800

C 12.38299100 0.95382700 3.64583300

C 13.57532500 0.17762400 3.77712700

C 14.84461900 0.81788800 3.67931200

C 16.03699400 0.03575900 3.76698400

C 16.19992100 2.60136100 2.66365400

C 17.39909900 2.00051800 3.16422900

C 18.66534800 2.53330400 2.79456500

C 19.81770000 1.89645400 3.35692100

C 19.98336800 3.68135500 0.97679900

C -1.75624600 -2.60072100 -2.67627900

C 0.72292600 -2.51545600 -2.74186600

C 2.07047600 -3.73477700 -1.14001700

C 3.26325300 -3.36210300 -1.82630400

C 4.53762800 -3.63981400 -1.24343600

C 5.72528800 -3.29460700 -1.94837000

C 5.87726500 -3.75638600 0.81381400

C 7.07017700 -3.84159800 0.03784200

C 8.33893700 -3.77316700 0.69005000

C 9.53176000 -3.83778600 -0.08976800

C 9.68240900 -2.85108200 2.53359500

C 10.87444000 -3.31796700 1.90598600

C 12.14504600 -2.93936200 2.43803600

C 13.33950800 -3.38574600 1.79289100

C 13.49559400 -1.23680600 3.59221700

C 14.68417800 -1.95142700 3.27024300

C 15.95702800 -1.35990800 3.53640200

C 17.15770900 -2.06621300 3.20118800

C 17.31747200 0.67562200 3.70452600

C 18.50208700 -0.09977300 3.82924300

C 19.73953000 0.61847300 3.84845300

C 3.18438000 -2.41033200 -2.87947200

C 5.64908900 -2.32281300 -2.99465400

C 6.99502400 -3.58410600 -1.36370500

C 8.18750000 -3.22237300 -2.05877000

C 9.45688700 -3.53500700 -1.48334900

C 10.64921600 -3.15755900 -2.16700700

C 10.79998000 -3.79676000 0.56249600

C 11.99215800 -3.83421000 -0.21994800

C 13.26419500 -3.81717300 0.43327100

C 14.45349500 -3.83180700 -0.34684800

C 14.60643300 -3.02772600 2.33548000

C 15.79915400 -3.43707800 1.66668000

C 17.07885400 -3.11119700 2.22367200

C 18.26401000 -3.55817400 1.58011600

C 18.42287500 -1.51223500 3.53978600

C 19.57392200 -2.28726900 3.19575000

C 8.11318600 -2.22648800 -3.07734000

C 11.91777400 -3.48822700 -1.60024800

C 13.11346600 -3.09134700 -2.27350500

C 14.38067400 -3.44232800 -1.72086600

C 15.57279300 -3.01722200 -2.37171600

C 15.72265400 -3.82194600 0.30503000

C 16.92572600 -3.82526800 -0.47328400

C 18.18750200 -3.90481200 0.17707000

C 19.33662100 -3.94373800 -0.66761300

C 19.49799200 -3.27133500 2.24069600

C 16.85338500 -3.39014200 -1.83713200

C 18.04275100 -3.01382600 -2.51269900

C 19.26587500 -3.50542600 -1.96960800

H 21.03102100 3.86192700 -0.85671400

H 20.89890100 3.73875400 1.55735000

H 20.79294200 2.36408500 3.26605400

H 20.65472900 0.11009600 4.13577600

H 20.54954400 -2.00649400 3.57961400

H 20.41552900 -3.74077700 1.89964300

H 20.31017800 -4.17879400 -0.24915200

H 20.18646300 -3.41101800 -2.53730300

H 21.22892700 0.68748000 -3.93623900

H 21.13358500 2.83591900 -2.78102900

H 20.11568000 -1.57617100 -3.66932600

H -20.32496800 4.20109700 -0.74468000

H -20.20643700 3.16172000 -2.92323900

H -20.14602400 1.19936500 -3.81875800

H -21.27017800 -1.07435000 -3.80911100

H -21.18138500 -3.07351500 -2.41135900

H -21.07792100 -3.87908800 -0.38429400

H -20.94154400 -3.47161200 1.99822700

H -20.82955400 -1.89557100 3.52654400

H -20.68375600 0.44626900 4.11828600

H -20.57284500 2.48100600 3.31047600

H -20.43284900 4.00818500 1.44101200

C -0.39876400 -5.83922700 0.95306100

C 0.53545400 -6.84933800 0.23226800

C 0.51529600 -6.71715600 -1.33031500

C -0.46397300 -5.62790400 -1.84502900

F -0.22919600 -5.47899800 -3.17627600

F -1.70411600 -6.17550900 -1.72452100

F 1.76634300 -6.44525800 -1.77054800

F 0.13888800 -7.89874800 -1.86877200

F 1.80396600 -6.68402100 0.67588000

F 0.13833900 -8.09501400 0.57554800

F -1.65587100 -6.33935000 0.81113700

F -0.10071500 -5.90195600 2.27877700

**(6,5) SWNT>(CF_2_)_4_ (1,2-L_+_: L_27_)**

C -20.40744400 -1.48355400 -3.50942000

C -19.21945300 -0.70251700 -3.73443700

C -19.25853800 0.69378800 -3.85284100

C -18.10054800 1.46673700 -3.63219200

C -17.96411300 -1.34420000 -3.50452900

C -16.76781600 -0.61117200 -3.73062000

C -15.50106400 -1.26021000 -3.55971200

C -14.29988700 -0.52763500 -3.77484100

C -19.36858200 3.27767800 -2.39949100

C -18.14955900 2.71694300 -2.88188900

C -16.95704700 3.15970400 -2.25411000

C -16.83078000 0.81073800 -3.75365600

C -15.61853700 1.53852700 -3.52956500

C -14.35686300 0.89955300 -3.73065500

C -13.15462200 1.62156100 -3.47615500

C -13.03897600 -1.17939700 -3.61841400

C -11.83263600 -0.43288600 -3.79084400

C -10.57197400 -1.09150500 -3.65788400

C -9.36440100 -0.34015200 -3.79425700

C -19.60030200 3.51129600 1.80891800

C -19.43593400 3.86187200 -1.15578700

C -18.28713100 3.90889000 -0.31117900

C -17.02625600 3.74845700 -0.94886700

C -15.82312900 3.82660000 -0.17522700

C -15.67925000 2.71751200 -2.74136400

C -14.48384600 3.20623700 -2.14364900

C -13.21846000 2.78251100 -2.64930100

C -12.02009100 3.24701700 -2.02643100

C -11.88910000 0.98832500 -3.69047200

C -10.68675800 1.70362800 -3.40829200

C -9.42232400 1.07886500 -3.63881500

C -8.22147600 1.78365400 -3.32636200

C -8.10469500 -1.00356200 -3.68256500

C -6.89450400 -0.24685900 -3.77109900

C -5.63358400 -0.91240500 -3.67160600

C -4.43130400 -0.15431200 -3.70542900

C -3.10339200 -2.16892400 -3.14120100

C -0.66686800 -2.09539600 -3.15449400

C -19.67971000 2.64079900 2.86834900

C -18.53144400 1.90596200 3.29875000

C -18.36519400 3.71946600 1.12165800

C -17.18188000 3.34172000 1.81136700

C -15.90125600 3.59830100 1.22145800

C -14.71073900 3.26196000 1.93252300

C -14.55445800 3.75402600 -0.82454500

C -13.36546300 3.82398200 -0.04746400

C -12.09342000 3.75720700 -0.69773100

C -10.90269200 3.80883700 0.08497100

C -10.75284300 2.83928300 -2.54514400

C -9.55846400 3.28839000 -1.90939900

C -8.28977500 2.89568400 -2.43523800

C -7.09660400 3.33455600 -1.78716600

C -6.95515700 1.16465300 -3.56837600

C -5.75774100 1.86580500 -3.23749300

C -4.49325300 1.25375700 -3.48516600

C -3.29798300 1.95296200 -3.14219400

C -3.16730300 -0.82123600 -3.59069400

C -1.97009700 -0.05921500 -3.64336400

C -0.71775400 -0.72454200 -3.57801200

C 0.47835200 0.04630900 -3.64358600

C 1.82201200 -1.98072900 -3.28145500

C 4.29200400 -1.85876600 -3.41063000

C -19.85504100 -0.16892100 3.85816300

C -18.61532800 0.53699200 3.74990400

C -17.43398700 -0.25288400 3.71319000

C -17.26438500 2.41243400 2.89906400

C -16.06616800 1.74376500 3.31189000

C -14.79190200 2.29650600 2.98118800

C -13.60577000 1.61699500 3.38011400

C -13.44308000 3.55201100 1.35279300

C -12.25127400 3.17893900 2.04611000

C -10.98062400 3.49193400 1.47538700

C -9.79146000 3.09833200 2.15530000

C -9.63342400 3.76417100 -0.56541400

C -8.44242900 3.79433300 0.21900200

C -7.17208300 3.77427700 -0.43192500

C -5.98108000 3.78619200 0.35358200

C -5.82745500 2.96123700 -2.32250100

C -4.63635000 3.38685800 -1.66405600

C -3.36684900 3.02746000 -2.20885500

C -2.17245800 3.43645400 -1.54307800

C -2.03418800 1.35195000 -3.41151400

C -0.84282400 2.03805800 -3.06448900

C 0.41834900 1.43700600 -3.35833600

C 1.61898100 2.12252700 -3.00754200

C 1.74776300 -0.61168100 -3.66329400

C 2.93997800 0.16839100 -3.71876900

C 4.21323900 -0.48211500 -3.75695200

C 5.41133400 0.30109500 -3.75997000

C 6.75630800 -1.74077900 -3.47928400

C 9.22184900 -1.62378400 -3.53310700

C -20.12168100 -3.54174600 1.36170800

C -19.93974400 -1.49545300 3.51986800

C -18.79215100 -2.19832500 3.03168900

C -17.52240300 -1.63133000 3.33208500

C -16.32701100 -2.29182500 2.90174100

C -16.15070100 0.38355100 3.69927800

C -14.96154600 -0.40860000 3.69923200

C -13.68938100 0.23251200 3.72090500

C -12.49917000 -0.55811300 3.67415200

C -12.33122700 2.17709600 3.05641800

C -11.14124900 1.48079900 3.42832600

C -9.87011900 2.05562200 3.12755700

C -8.67881300 1.34655900 3.46707100

C -8.52043000 3.43275000 1.59666300

C -7.32977200 3.01817200 2.26331400

C -6.05883100 3.37766500 1.71905200

C -4.86715000 2.94141500 2.36926900

C -4.71043100 3.78916800 -0.29653500

C -3.51776600 3.77400500 0.48812000

C -2.24500900 3.79618600 -0.16281200

C -1.05122500 3.74936900 0.61615900

C -0.90695200 3.09235000 -2.10194900

C 0.28992700 3.46622300 -1.42637400

C 1.55589100 3.13302400 -2.00360300

C 2.75392000 3.48528900 -1.31882300

C 2.87811000 1.54783400 -3.35003400

C 4.07844500 2.21315100 -2.96211500

C 5.34496500 1.65873100 -3.32896000

C 6.54414500 2.30749300 -2.90834000

C 6.68040500 -0.35234900 -3.80444900

C 7.87791100 0.42626800 -3.76854200

C 9.14865100 -0.22881700 -3.82329700

C 10.34530000 0.54599700 -3.76254400

C 10.48954700 -2.20694200 -3.23136600

C 11.68396900 -1.51165700 -3.58275900

C 12.95417400 -2.10671400 -3.29761200

C 14.14893700 -1.40615800 -3.62913700

C -20.20185600 -3.76282400 0.01237200

C -19.04621500 -3.60956200 -0.82310500

C -18.87902100 -3.16859700 1.97059700

C -17.69658600 -3.47082300 1.24138500

C -16.41474300 -3.20860700 1.82481400

C -15.22438000 -3.54473400 1.10985400

C -15.04930400 -1.76545200 3.26796100

C -13.86479800 -2.42047900 2.82412600

C -12.58652600 -1.89750900 3.19593200

C -11.39889100 -2.53781100 2.72590800

C -11.22358000 0.08481500 3.71431200

C -10.03487000 -0.70213800 3.63532800

C -8.75918800 -0.05983900 3.69443400

C -7.56975900 -0.84137700 3.58054100

C -7.40813300 1.93700800 3.19096000

C -6.21616800 1.21658900 3.50023700

C -4.94604900 1.82253400 3.25282700

C -3.75550300 1.09172400 3.53186600

C -3.59557400 3.31910600 1.83751400

C -2.40202300 2.85585300 2.47055100

C -1.12839000 3.24764900 1.95322700

C 0.06227300 2.75548500 2.56455500

C 0.21952200 3.78066600 -0.03699300

C 1.41589700 3.69755200 0.73884100

C 2.68503300 3.74866200 0.08357900

C 3.87866700 3.63429500 0.85408200

C 4.01658500 3.17443800 -1.91005400

C 5.21668600 3.49898400 -1.20911500

C 6.48067500 3.22064500 -1.81231600

C 7.67804700 3.51563500 -1.09637500

C 7.81044200 1.76754000 -3.29104100

C 9.01123600 2.39752900 -2.83833700

C 10.27795000 1.87326600 -3.23932800

C 11.47571200 2.48215500 -2.75827300

C 11.61296400 -0.10989000 -3.83062200

C 12.81245800 0.66212200 -3.73997400

C 14.07875400 0.00762400 -3.82976500

C 15.27368200 0.76899300 -3.69884700

C 15.41490500 -2.00831800 -3.35580800

C 16.62099200 -1.30061000 -3.66137500

C 17.89540000 -1.92864200 -3.46602600

C 19.04683400 -1.17724300 -3.77764900

C -20.35619900 -2.60258600 -2.72815100

C -19.11654500 -3.03598300 -2.13711000

C -17.91538100 -2.53318700 -2.71274500

C -17.77891700 -3.68010700 -0.17420500

C -16.57765100 -3.57649300 -0.94312000

C -15.30563200 -3.70910100 -0.30333500

C -14.11540100 -3.59126500 -1.07659100

C -13.95300100 -3.31046700 1.71086500

C -12.76399600 -3.61234800 0.98023300

C -11.48875400 -3.39313400 1.58743500

C -10.30259000 -3.67698900 0.84812200

C -10.12177500 -2.02654400 3.11138400

C -8.93355700 -2.64925900 2.61755800

C -7.65552000 -2.14616900 3.01110200

C -6.46785200 -2.74842000 2.48762300

C -6.29512700 -0.19891300 3.66266600

C -5.10451100 -0.97070600 3.51161700

C -3.83594000 -0.32872100 3.62517800

C -2.64547800 -1.09108400 3.43377100

C -2.48265800 1.70602400 3.31017900

C -1.29294400 0.96533000 3.56710600

C -0.02067300 1.57677100 3.36698900

C 1.16624800 0.81675700 3.59296300

C 1.33778300 3.15264900 2.05429600

C 2.52761900 2.63343100 2.64905300

C 3.80048500 3.04452600 2.15155300

C 4.98882500 2.50316200 2.72826600

C 5.14776800 3.70548400 0.20035100

C 6.34093000 3.56684100 0.96900600

C 7.60832300 3.66514100 0.32061300

C 8.80148500 3.49960600 1.08592900

C 8.94522300 3.26307200 -1.70730800

C 10.14177000 3.53321000 -0.97851300

C 11.40809700 3.30488500 -1.59583100

C 12.60519700 3.54637600 -0.85362800

C 12.74520300 1.96824100 -3.17261500

C 13.94224100 2.56030900 -2.67011600

C 15.20821200 2.05439800 -3.09239700

C 16.40733300 2.62555800 -2.56157000

C 16.54576500 0.11415300 -3.80054000

C 17.73612700 0.87726100 -3.66238500

C 18.99609200 0.22284900 -3.82177600

C 20.17818800 1.03426400 -3.69221500

C -16.64585000 -2.97296700 -2.22358500

C -15.44502000 -2.47258400 -2.81689200

C -14.18250600 -2.94195300 -2.34688200

C -12.98305900 -2.41918400 -2.91574300

C -12.84587600 -3.73336900 -0.43749300

C -11.65321900 -3.58981500 -1.20798200

C -10.38646500 -3.75662700 -0.57388300

C -9.19464800 -3.59303800 -1.33975700

C -9.02555100 -3.47576700 1.45831600

C -7.84119400 -3.74551300 0.71050300

C -6.56225500 -3.55243900 1.31829100

C -5.37672900 -3.80513700 0.55689400

C -5.18581500 -2.25099900 2.88234400

C -4.00270900 -2.81787800 2.32186900

C -2.72447100 -2.32634700 2.73565800

C -1.53712000 -2.87816700 2.17296500

C -1.37454200 -0.46080300 3.59977100

C -0.19276700 -1.21992000 3.40437400

C 1.07992800 -0.60528400 3.58844700

C 2.26554100 -1.37134600 3.38004600

C 2.44298200 1.43046400 3.41341700

C 3.62600900 0.66270500 3.62199900

C 4.90262400 1.28060600 3.45737100

C 6.08860900 0.50944300 3.64989000

C 6.26235000 2.93300100 2.24592600

C 7.44887700 2.37846400 2.80819100

C 8.72232600 2.82450800 2.33925900

C 9.91098300 2.25809100 2.88872400

C 10.07054900 3.62774800 0.44448500

C 11.26129000 3.43842400 1.20438800

C 12.53217000 3.58884200 0.56838700

C 13.72502100 3.37761000 1.32365400

C 13.87401500 3.34426000 -1.47801000

C 15.06668000 3.55471900 -0.72766800

C 16.33843700 3.36234600 -1.35322800

C 17.54153800 3.55547600 -0.60423100

C 17.67918900 2.14634200 -3.00657600

C 18.87793500 2.71702800 -2.49309600

C 20.11954600 2.23258100 -3.03985600

C -11.71900400 -2.90064300 -2.45524400

C -10.51875300 -2.35966200 -3.00525600

C -9.25713100 -2.86013000 -2.56282900

C -8.05420400 -2.30164800 -3.09005300

C -7.92988600 -3.78634000 -0.71319900

C -6.74164200 -3.60404900 -1.47777200

C -5.47580400 -3.83311700 -0.86216200

C -4.29397900 -3.67822600 -1.63000100

C -4.09283900 -3.59252500 1.13164200

C -2.91101800 -3.83120400 0.33823200

C -1.60346600 -3.62612900 0.94984900

C -0.43122000 -3.89459000 0.24792200

C -0.27562000 -2.43511200 2.65153600

C 0.91176700 -2.98694300 2.10161500

C 2.18419100 -2.57544000 2.61875100

C 3.37649900 -3.13677300 2.08640600

C 3.53867200 -0.76328100 3.58848100

C 4.72629500 -1.52280900 3.36775500

C 6.00247500 -0.91416900 3.58641500

C 7.19190000 -1.66422400 3.34977300

C 7.36315700 1.13529200 3.50493600

C 8.55086300 0.36249800 3.67703000

C 9.82655000 0.99547200 3.55071200

C 11.01387400 0.22162900 3.70258800

C 11.18199500 2.72174300 2.43598500

C 12.37194900 2.13820500 2.96711300

C 13.64566100 2.61934000 2.53170700

C 14.83245600 2.02140900 3.04126400

C 14.99239500 3.54694100 0.69506200

C 16.18679000 3.29662300 1.43861300

C 17.46536400 3.49840500 0.82582700

C 18.65135400 3.28224200 1.57946000

C 18.80747800 3.42430700 -1.24618400

C 19.96511500 3.66935900 -0.43563200

C -6.79683700 -2.82680100 -2.66727100

C -5.58022900 -2.24856200 -3.15568800

C -4.33778400 -2.80366600 -2.76182900

C -3.03080900 -3.96580500 -1.04103000

C -1.84556200 -4.07273700 -2.00791600

C -0.45838600 -4.36700900 -1.22699200

C 0.74536300 -3.69115900 -1.89620200

C 0.83260900 -3.70199800 0.87280200

C 2.06076400 -3.94420600 0.16292400

C 3.30743100 -3.80247500 0.81853000

C 4.51875000 -3.93583600 0.06455500

C 4.64590900 -2.71674000 2.59148800

C 5.84707500 -3.24513800 2.02153000

C 7.11406500 -2.83497900 2.53373300

C 8.31244800 -3.32676200 1.92929200

C 8.46635200 -1.05727200 3.57725800

C 9.65988800 -1.79778900 3.31392400

C 10.93121300 -1.19709200 3.55974200

C 12.12418300 -1.92541500 3.26336900

C 12.28855500 0.85953900 3.58973600

C 13.48006600 0.08169700 3.71850800

C 14.74975000 0.72143200 3.62343700

C 15.94140400 -0.06145800 3.70961000

C 16.10675600 2.50668400 2.61202200

C 17.30536600 1.90370600 3.11109400

C 18.57193200 2.43667400 2.74291800

C 19.72367600 1.79769900 3.30383900

C 19.89051700 3.59181400 0.92962100

C -1.84681300 -2.75013400 -2.80931600

C 0.63512200 -2.66799000 -2.83586700

C 2.00949800 -3.86998100 -1.26536300

C 3.19262800 -3.49570000 -1.95154700

C 4.45852200 -3.74151000 -1.34186700

C 5.64796500 -3.39183100 -2.04493300

C 5.78141800 -3.83463100 0.72202100

C 6.97799600 -3.92667000 -0.04823500

C 8.24404000 -3.85392500 0.60711800

C 9.44068200 -3.92028400 -0.16990900

C 9.58340900 -2.93587800 2.45487700

C 10.77714800 -3.40392100 1.83031200

C 12.04744100 -3.02855000 2.36567400

C 13.24373200 -3.47399100 1.72129200

C 13.39864300 -1.33197900 3.52861300

C 14.58723700 -2.04634800 3.20628900

C 15.86032400 -1.45652200 3.47571900

C 17.06121000 -2.16255000 3.14007900

C 17.22244500 0.57791800 3.64905900

C 18.40638900 -0.19822900 3.77343700

C 19.64434400 0.51901200 3.79358800

C 3.10051700 -2.53659900 -2.99853400

C 5.56626000 -2.42150700 -3.08880100

C 6.91115800 -3.67481600 -1.45249900

C 8.10351300 -3.30766500 -2.14295000

C 9.37081200 -3.61772300 -1.56319600

C 10.56262100 -3.23709700 -2.24397000

C 10.70627800 -3.88016100 0.48512700

C 11.90038500 -3.91548700 -0.29523700

C 13.17122700 -3.90035600 0.36007200

C 14.36170400 -3.91235900 -0.41851400

C 14.51005100 -3.11939200 2.26723400

C 15.70385100 -3.52644600 1.59843800

C 16.98307700 -3.20384600 2.15863700

C 18.16896800 -3.64857200 1.51482800

C 18.32627700 -1.61036400 3.48180900

C 19.47721900 -2.38527200 3.13745700

C 8.02722200 -2.31018800 -3.15825100

C 11.82931300 -3.56641200 -1.67463900

C 13.02549500 -3.16613800 -2.34506900

C 14.29133300 -3.51782200 -1.79140700

C 15.48382700 -3.08940800 -2.43908100

C 15.62970700 -3.90499100 0.23491700

C 16.83403600 -3.90453600 -0.54199800

C 18.09474500 -3.98743100 0.10960800

C 19.24521100 -4.02094000 -0.73344600

C 19.40203700 -3.36575800 2.17865300

C 16.76377600 -3.46330300 -1.90395800

C 17.95372300 -3.08386800 -2.57669600

C 19.17634800 -3.57634400 -2.03349700

H 20.93817400 3.78214100 -0.90289500

H 20.80609700 3.64593500 1.51041400

H 20.69938500 2.26448400 3.21359900

H 20.55918300 0.00944800 4.07992600

H 20.45240500 -2.10667300 3.52400000

H 20.31969500 -3.83478100 1.83734300

H 20.21832400 -4.25699400 -0.31452500

H 20.09790100 -3.47840000 -2.59902200

H 21.13800400 0.62292300 -3.99459100

H 21.04082900 2.76840200 -2.83363600

H 20.02665500 -1.64235300 -3.72976600

H -20.40744700 4.15202600 -0.76829500

H -20.28989300 3.12728200 -2.95381500

H -20.23417400 1.17000700 -3.85751300

H -21.36526000 -1.09951600 -3.85147100

H -21.28140900 -3.10271100 -2.45943400

H -21.17712800 -3.92048800 -0.43699300

H -21.03535400 -3.52991000 1.94790400

H -20.91691900 -1.96681300 3.48810800

H -20.76717600 0.37277900 4.08853200

H -20.65656400 2.40767900 3.27998000

H -20.51645100 3.94160600 1.41634900

C -0.26090800 -5.92979600 -1.16241900

C -0.10175500 -6.64328200 -2.53275200

C -0.97295300 -5.99382500 -3.63524400

C -2.18606600 -5.25438500 -3.04446300

F -1.34249400 -6.46297000 -0.52723700

F 0.82129600 -6.28731300 -0.42543000

F -0.45224100 -7.94020700 -2.38929600

F 1.19768500 -6.58565300 -2.91049600

F -1.40602600 -6.94416600 -4.49157200

F -0.22575100 -5.10161900 -4.33435500

F -2.96803400 -6.20804800 -2.46493800

F -2.88155400 -4.78113900 -4.11249600

**(6,5) SWNT>(CF_2_)_4_ (1,2- L–: L_–33_)**

C -21.13965200 -1.97588900 -1.93460200

C -19.95258600 -1.19255000 -2.15677300

C -19.99343500 0.20408800 -2.27118900

C -18.83648400 0.97781800 -2.04823700

C -18.69654900 -1.83326300 -1.92833600

C -17.50122700 -1.09797300 -2.15173300

C -16.23345800 -1.74589300 -1.98213900

C -15.03352500 -1.01140700 -2.19533800

C -20.10693000 2.78398600 -0.81103500

C -18.88714100 2.22601500 -1.29434300

C -17.69548400 2.66801100 -0.66447200

C -17.56597700 0.32369700 -2.17094000

C -16.35446400 1.05250100 -1.94442500

C -15.09219200 0.41592300 -2.14738300

C -13.89108300 1.13852600 -1.88961800

C -13.77197200 -1.66187200 -2.03922300

C -12.56611000 -0.91315200 -2.21030900

C -11.30521500 -1.57034500 -2.07923900

C -10.09981600 -0.81636400 -2.21227700

C -20.34168100 3.00479200 3.39764400

C -20.17554900 3.36452400 0.43439000

C -19.02733500 3.41020400 1.27969700

C -17.76603000 3.25254700 0.64243400

C -16.56323400 3.32918800 1.41718500

C -16.41677400 2.22883500 -1.15248500

C -15.22225200 2.71686800 -0.55271800

C -13.95632400 2.29571200 -1.05887300

C -12.75787100 2.75905700 -0.43340000

C -12.62404600 0.50750400 -2.10615600

C -11.42314000 1.22324800 -1.82198300

C -10.15806000 0.59943500 -2.05323900

C -8.95539300 1.30481700 -1.73845700

C -8.83642700 -1.47927600 -2.10224800

C -7.63018900 -0.72180300 -2.19600600

C -6.36821800 -1.38968000 -2.09716400

C -5.15628600 -0.63066600 -2.13981400

C -3.84323200 -2.64818100 -1.58130400

C -1.35551300 -2.55610700 -1.55831200

C -20.42116000 2.13088300 4.45436900

C -19.27257800 1.39616200 4.88371900

C -19.10626600 3.21628700 2.71214900

C -17.92325600 2.83742000 3.40149800

C -16.64226000 3.09661800 2.81318900

C -15.45211400 2.75984800 3.52431300

C -15.29421900 3.25986700 0.76864400

C -14.10585400 3.32753500 1.54685300

C -12.83287100 3.26365000 0.89741000

C -11.64308700 3.31359800 1.68106400

C -11.49048000 2.35505400 -0.95311500

C -10.29645400 2.80012500 -0.31307700

C -9.02594100 2.41057400 -0.83965700

C -7.83432600 2.84493500 -0.18780500

C -7.69029100 0.68898400 -1.98487700

C -6.49099800 1.38455300 -1.64237700

C -5.22258400 0.77546200 -1.89562100

C -4.03157600 1.47205000 -1.54041400

C -3.89770600 -1.29812400 -2.03857300

C -2.69649800 -0.52765400 -2.04784000

C -1.43367400 -1.18851900 -1.96766500

C -0.24477100 -0.41391600 -2.02029300

C 1.07983300 -2.44367500 -1.64456300

C 3.54943800 -2.33328000 -1.80552700

C -20.59437900 -0.68208300 5.43544300

C -19.35537700 0.02557300 5.33090000

C -18.17319600 -0.76267600 5.29331400

C -18.00580300 1.90508400 4.48657700

C -16.80722600 1.23665800 4.89889200

C -15.53320800 1.79174700 4.57122000

C -14.34720600 1.11296100 4.97012800

C -14.18455900 3.05178600 2.94606000

C -12.99272000 2.67825200 3.63994400

C -11.72222400 2.99360900 3.07142400

C -10.53392100 2.59976600 3.75212700

C -10.37356600 3.26974800 1.03184300

C -9.18263100 3.29867200 1.81835500

C -7.91255800 3.28103700 1.16958200

C -6.72341700 3.29164800 1.95780300

C -6.56427600 2.47006500 -0.72051400

C -5.37303900 2.89719400 -0.05726700

C -4.10445400 2.54398000 -0.60017400

C -2.91334900 2.95500900 0.07289200

C -2.76518400 0.86879900 -1.79760100

C -1.57089600 1.57172100 -1.44727700

C -0.31149300 0.98990300 -1.74500000

C 0.88311300 1.67060600 -1.38223900

C 1.01448000 -1.06616600 -2.00589700

C 2.21204900 -0.28612400 -2.06736900

C 3.47594900 -0.93713500 -2.12407600

C 4.67053300 -0.15737600 -2.12473100

C 6.02300100 -2.20082300 -1.88432100

C 8.48757000 -2.07648600 -1.93602100

C -20.85403300 -4.04878800 2.92989800

C -20.67703000 -2.00780000 5.09335000

C -19.52795900 -2.70793100 4.60478700

C -18.25939100 -2.14019500 4.90819100

C -17.06265900 -2.79766100 4.47687300

C -16.89059500 -0.12458000 5.28264700

C -15.70046500 -0.91515000 5.28244000

C -14.42946700 -0.27251200 5.30787400

C -13.23820400 -1.06156000 5.26043400

C -13.07264900 1.67495700 4.64880200

C -11.88240300 0.98039500 5.02232600

C -10.61259200 1.55664100 4.72354900

C -9.42005700 0.84813000 5.06430100

C -9.26222400 2.93581700 3.19550900

C -8.07251300 2.52371300 3.86459600

C -6.80280100 2.88336000 3.32188600

C -5.60997600 2.44814200 3.97522100

C -5.45108700 3.29800400 1.31022900

C -4.26120000 3.28839600 2.09765300

C -2.98947700 3.31265900 1.44965500

C -1.79361900 3.27209800 2.23470900

C -1.64267300 2.62114000 -0.48464500

C -0.44904700 3.00842600 0.19173200

C 0.81621900 2.68257300 -0.37949400

C 2.01601600 3.03462500 0.31023600

C 2.14614000 1.09280000 -1.71338400

C 3.34415700 1.76147200 -1.33081700

C 4.60564500 1.20167700 -1.69249600

C 5.80693700 1.85104500 -1.27332000

C 5.94495800 -0.80709500 -2.18587400

C 7.13828000 -0.02765400 -2.14773900

C 8.41130000 -0.67911300 -2.20976900

C 9.60757400 0.09750400 -2.14435400

C 9.75779100 -2.66089500 -1.64112800

C 10.94991400 -1.96012300 -1.98510400

C 12.22142900 -2.55505900 -1.70434400

C 13.41522500 -1.84969600 -2.02942600

C -20.93280400 -4.26594200 1.57989300

C -19.77672500 -4.10827300 0.74579000

C -19.61237500 -3.67546000 3.54083400

C -18.42901200 -3.97359200 2.81177700

C -17.14796600 -3.71086200 3.39699200

C -15.95627100 -4.04292000 2.68208500

C -15.78587800 -2.27099700 4.84654000

C -14.60039800 -2.92257700 4.40193400

C -13.32319500 -2.39878000 4.77705900

C -12.13370300 -3.03564500 4.30665500

C -11.96292600 -0.41739600 5.30580700

C -10.77431800 -1.20193500 5.22739600

C -9.49869400 -0.55834300 5.28935300

C -8.30750100 -1.33773300 5.17919900

C -8.15030300 1.44090400 4.79261700

C -6.95865400 0.72184500 5.10274900

C -5.68812600 1.32904000 4.85725500

C -4.49603100 0.60145800 5.14015100

C -4.33995700 2.83142300 3.44858000

C -3.14704700 2.36914900 4.08192900

C -1.87270600 2.76718400 3.56747000

C -0.68019900 2.27932600 4.18184200

C -0.52318200 3.31869300 1.58644800

C 0.67157800 3.23581400 2.36324600

C 1.94381400 3.29193600 1.71114900

C 3.13865200 3.17763400 2.48379300

C 3.27939000 2.72902000 -0.28047000

C 4.47743900 3.04904100 0.42158400

C 5.74244100 2.76605200 -0.18027300

C 6.93987900 3.06086200 0.53548300

C 7.07134200 1.31397900 -1.66116900

C 8.27086900 1.94206900 -1.20803000

C 9.53918100 1.42112700 -1.61258700

C 10.73592900 2.02983100 -1.13084900

C 10.87623700 -0.55541500 -2.22011400

C 12.07458600 0.21726200 -2.12369000

C 13.34260900 -0.43459400 -2.21906300

C 14.53625900 0.32741200 -2.08347900

C 14.68196100 -2.45202600 -1.76031700

C 15.88689200 -1.74030700 -2.05978500

C 17.16245000 -2.36781500 -1.86821300

C 18.31249000 -1.61298100 -2.17522300

C -21.08718300 -3.09726700 -1.15687400

C -19.84711000 -3.53078200 -0.56654600

C -18.64650200 -3.02455500 -1.13985500

C -18.50985000 -4.17877000 1.39540300

C -17.30818200 -4.07051700 0.62778800

C -16.03601500 -4.20295100 1.26818800

C -14.84570200 -4.08091600 0.49648800

C -14.68600800 -3.80872600 3.28503100

C -13.49591400 -4.10497600 2.55413600

C -12.22081900 -3.88595100 3.16366900

C -11.03432000 -4.16413000 2.42534400

C -10.85853300 -2.52502500 4.69731200

C -9.66928800 -3.14280600 4.20159200

C -8.38979200 -2.64348200 4.60354800

C -7.20429600 -3.24397300 4.08666700

C -7.03633200 -0.69396400 5.26396900

C -5.84271300 -1.46524100 5.11399900

C -4.57424100 -0.82299100 5.23369200

C -3.38402900 -1.58238400 5.04379200

C -3.22713300 1.21735000 4.91859500

C -2.03306700 0.47515200 5.16989200

C -0.76238200 1.09284300 4.97697800

C 0.42544600 0.33459900 5.19354100

C 0.59238300 2.68194600 3.67640000

C 1.78583700 2.15809900 4.26744400

C 3.05962500 2.57687600 3.77714800

C 4.24726200 2.02906800 4.34840200

C 4.40694300 3.25217500 1.83259900

C 5.60142900 3.10562000 2.60082300

C 6.86934900 3.20706800 1.95379900

C 8.06138600 3.03393300 2.71764400

C 8.20519000 2.80672000 -0.07535300

C 9.40263700 3.07391400 0.65431000

C 10.66841200 2.84853100 0.03578800

C 11.86482700 3.08601000 0.77902900

C 12.00589700 1.51960900 -1.54795300

C 13.20282400 2.11040400 -1.04246500

C 14.46929200 1.60907700 -1.46832800

C 15.66766300 2.17784800 -0.93360900

C 15.80940200 -0.32484500 -2.18956000

C 16.99885400 0.43895600 -2.04644500

C 18.25965200 -0.21251800 -2.21048200

C 19.44057300 0.59936700 -2.07538000

C -17.37655600 -3.46358700 -0.65078000

C -16.17595700 -2.95995100 -1.24217000

C -14.91315900 -3.42860600 -0.77262600

C -13.71448000 -2.90225100 -1.33918700

C -13.57616300 -4.22141800 1.13658700

C -12.38251300 -4.07390300 0.36738000

C -11.11606200 -4.23990900 1.00195000

C -9.92442900 -4.07000100 0.23849100

C -9.75858500 -3.96040400 3.03777400

C -8.57125400 -4.22463800 2.29098200

C -7.29509100 -4.04074200 2.90534200

C -6.11198800 -4.28983100 2.14727100

C -5.92262700 -2.74565500 4.48752900

C -4.73570100 -3.32027100 3.94326000

C -3.46478500 -2.82340600 4.34756400

C -2.27437700 -3.35655100 3.75926900

C -2.11356200 -0.94828700 5.19747600

C -0.92674500 -1.70234400 4.98510100

C 0.33995000 -1.08812000 5.16518500

C 1.52648200 -1.84929100 4.92805200

C 1.70157800 0.94912700 5.01625800

C 2.88663000 0.17617000 5.21337400

C 4.16093400 0.79484100 5.05974800

C 5.34612700 0.01706900 5.23848300

C 5.52169600 2.46073800 3.87065900

C 6.70828000 1.89723500 4.42776100

C 7.98119600 2.34685500 3.96513900

C 9.16936600 1.77180400 4.50818600

C 9.33093300 3.16274900 2.07705200

C 10.52132700 2.96708700 2.83626200

C 11.79155200 3.12031600 2.20162700

C 12.98415400 2.90233500 2.95575900

C 13.13412900 2.88736400 0.15403200

C 14.32624900 3.09394300 0.90601400

C 15.59805500 2.90619000 0.27991500

C 16.80086800 3.09425800 1.03104300

C 16.94033700 1.70329400 -1.38177300

C 18.13829600 2.27167700 -0.86417300

C 19.38039600 1.79297800 -1.41438500

C -12.44898700 -3.38278500 -0.87870700

C -11.25025900 -2.83998300 -1.42746700

C -9.98820400 -3.33706200 -0.98230500

C -8.78474300 -2.77587700 -1.50926300

C -8.65661800 -4.26123800 0.86691900

C -7.46851500 -4.07792600 0.10672600

C -6.20438400 -4.29558700 0.72930800

C -5.01470000 -4.10972900 -0.03407700

C -4.82178800 -4.10744900 2.74771100

C -3.65514700 -4.35077100 1.98279000

C -2.35186700 -4.10038200 2.54536900

C -1.16825300 -4.33870300 1.80583200

C -1.00843300 -2.90727000 4.21524800

C 0.17318500 -3.44233400 3.63692000

C 1.44277800 -3.03167900 4.14537300

C 2.63624800 -3.59363800 3.60108800

C 2.79994300 -1.24735400 5.15194200

C 3.98336300 -2.00702800 4.92200700

C 5.25899500 -1.40401100 5.15343800

C 6.45017600 -2.15416100 4.91444100

C 6.62159700 0.64429300 5.10653700

C 7.80772600 -0.13161600 5.27118800

C 9.08369300 0.50197200 5.15467800

C 10.27120600 -0.27403200 5.30128500

C 10.44097200 2.23958800 4.06171600

C 11.62971000 1.65021100 4.58783700

C 12.90381800 2.13452700 4.15738200

C 14.09020400 1.53237800 4.66315100

C 14.25154300 3.07624300 2.32905200

C 15.44514300 2.82000400 3.07113800

C 16.72408600 3.02617900 2.46053200

C 17.90959300 2.80475800 3.21334600

C 18.06703900 2.96902600 0.38866000

C 19.22405400 3.20746700 1.20169600

C -7.52659700 -3.29855500 -1.08789400

C -6.32014900 -2.71302700 -1.57805500

C -5.06089700 -3.26044700 -1.17376100

C -3.75695000 -4.39988400 0.55693100

C -2.56973300 -4.24748500 -0.21635900

C -1.28133400 -4.84708800 0.35238200

C -0.00687700 -4.48712400 -0.56834900

C 0.08312200 -4.14644700 2.39572100

C 1.31042200 -4.39315400 1.65066400

C 2.56967100 -4.25447500 2.33836100

C 3.78248900 -4.42922300 1.61546600

C 3.90174000 -3.19181300 4.12827200

C 5.10612600 -3.72346200 3.56857100

C 6.37454600 -3.31960000 4.09364200

C 7.57351800 -3.81540700 3.49775800

C 7.72300600 -1.55093200 5.15506900

C 8.91757900 -2.28985800 4.89005300

C 10.18907000 -1.69118100 5.14591300

C 11.38278100 -2.41765200 4.84957800

C 11.54547300 0.36519700 5.19801800

C 12.73722100 -0.41343900 5.32196900

C 14.00701000 0.22773500 5.23489900

C 15.19878000 -0.55494900 5.31756000

C 15.36436400 2.02112900 4.23866200

C 16.56278400 1.41478700 4.73419000

C 17.82945500 1.95094900 4.37108200

C 18.98066300 1.30899000 4.92913500

C 19.14884600 3.11911700 2.56646600

C -2.58850700 -3.23619100 -1.18423800

C -0.10890500 -3.11561100 -1.26994000

C 1.28502600 -4.45758000 0.25227600

C 2.47343000 -4.04696800 -0.41789000

C 3.73178300 -4.25747600 0.20466000

C 4.92215000 -3.87820400 -0.48223100

C 5.04601000 -4.32391500 2.28008100

C 6.24753300 -4.42272400 1.51580300

C 7.50982900 -4.34594300 2.17534900

C 8.70902100 -4.40359600 1.40109700

C 8.84374400 -3.42422900 4.02777400

C 10.04053000 -3.89107000 3.40617800

C 11.30885500 -3.51687400 3.94619800

C 12.50733100 -3.95731300 3.30185000

C 12.65669600 -1.82540500 5.12169100

C 13.84686000 -2.53734200 4.79802400

C 15.11889600 -1.94862500 5.07411400

C 16.32112200 -2.65148000 4.73598400

C 16.47966000 0.08556400 5.26324600

C 17.66395400 -0.69056500 5.38476500

C 18.90129800 0.02717600 5.41105300

C 2.37769100 -3.02681200 -1.41636000

C 4.83526700 -2.89327600 -1.50367900

C 6.18559900 -4.16296600 0.11316900

C 7.37431700 -3.78300500 -0.56963400

C 8.64176100 -4.09212100 0.01087200

C 9.83308700 -3.70116300 -0.66480100

C 9.97372500 -4.36269800 2.05946300

C 11.16844300 -4.39085700 1.28111800

C 12.43815300 -4.37596000 1.93845000

C 13.62980000 -4.38045000 1.16128500

C 13.77251700 -3.60464400 3.85228600

C 14.96778700 -4.00607800 3.18288000

C 16.24576600 -3.68583000 3.74714000

C 17.43327100 -4.12455000 3.10188000

C 17.58520500 -2.10091500 5.08401100

C 18.73726500 -2.87258500 4.73652800

C 7.29480100 -2.77170700 -1.57335400

C 11.09896500 -4.03210400 -0.09643300

C 12.29452500 -3.62234500 -0.76121300

C 13.56059400 -3.97590100 -0.20869200

C 14.75238100 -3.54009800 -0.85153300

C 14.89650500 -4.37529600 1.81647100

C 16.10180200 -4.36648900 1.04112300

C 17.36180700 -4.45221900 1.69378500

C 18.51333100 -4.47659000 0.85204000

C 18.66473600 -3.84591000 3.77015500

C 16.03242700 -3.91518400 -0.31750100

C 17.22235700 -3.52871900 -0.98621500

C 18.44521100 -4.02231900 -0.44485000

H 20.19747700 3.32338500 0.73597300

H 20.06434300 3.16812600 3.14784200

H 19.95634400 1.77658200 4.84278300

H 19.81611400 -0.48368100 5.69513000

H 19.71150500 -2.59613900 5.12697500

H 19.58343200 -4.31160200 3.42705300

H 19.48651100 -4.71330800 1.27043800

H 19.36743500 -3.91786000 -1.00810900

H 20.40094600 0.19134800 -2.38047500

H 20.30102300 2.32835800 -1.20412600

H 19.29305000 -2.07682800 -2.13020700

H -21.14759100 3.65244100 0.82221300

H -21.02781000 2.63412900 -1.36621900

H -20.96968700 0.67905100 -2.27458700

H -22.09783500 -1.59214800 -2.27596200

H -22.01178700 -3.59964200 -0.89028100

H -21.90743200 -4.42401300 1.12927800

H -21.76812200 -4.04038200 3.51550500

H -21.65361700 -2.48018200 5.05887800

H -21.50749800 -0.14212100 5.66599300

H -21.39818500 1.89540100 4.86424700

H -21.25796500 3.43521900 3.00553200

C -1.37687300 -6.42514000 0.54374100

C -1.29119400 -7.31880400 -0.70416700

C -0.02312900 -7.00683800 -1.50310100

C 0.07549800 -5.50038800 -1.79376600

F 1.22399700 -5.32293300 -2.49581600

F -0.95469400 -5.22613700 -2.64840700

F 1.06725700 -7.39600600 -0.79680100

F -0.04219800 -7.68695800 -2.67243900

F -1.28212400 -8.61697200 -0.32346200

F -2.38279800 -7.10553500 -1.48009100

F -0.34577700 -6.82305800 1.34727700

F -2.52498900 -6.77016600 1.18065900

**(6,5) SWNT>(CF_2_)_4_ (1,4-L_++_: L_87_)**

C -20.33238600 -1.42141200 -3.45303500

C -19.14626600 -0.63753400 -3.67755800

C -19.18786900 0.75913100 -3.79335900

C -18.03033200 1.53356900 -3.57647400

C -17.88953700 -1.27783100 -3.45115900

C -16.69513300 -0.54282300 -3.67890600

C -15.42688100 -1.19008900 -3.51049500

C -14.22848100 -0.45609800 -3.73058400

C -19.29420300 3.34697800 -2.34261400

C -18.07713100 2.78506300 -2.82764200

C -16.88249700 3.22948200 -2.20454100

C -16.76045700 0.87883400 -3.70212000

C -15.54851800 1.60868000 -3.48410000

C -14.28779600 0.97131000 -3.69035200

C -13.08621300 1.69642800 -3.44329200

C -12.96597600 -1.10479600 -3.57417400

C -11.76285700 -0.35629800 -3.75720900

C -10.50116500 -1.00977800 -3.62346900

C -9.29932000 -0.25455400 -3.77765400

C -19.51445900 3.58963300 1.86352700

C -19.35689700 3.93497200 -1.10028500

C -18.20551900 3.98308200 -0.25942000

C -16.94667800 3.81983700 -0.89997500

C -15.74082600 3.89558600 -0.12931700

C -15.60647500 2.78822900 -2.69629200

C -14.40916800 3.27742800 -2.10295200

C -13.14616700 2.85581000 -2.61518000

C -11.94420900 3.31824100 -1.99598500

C -11.82141000 1.06565800 -3.66570600

C -10.62035300 1.78525400 -3.39533200

C -9.35734100 1.16314200 -3.63775400

C -8.15331500 1.87331700 -3.34230100

C -8.03537900 -0.91145700 -3.66658600

C -6.83566700 -0.15156900 -3.79803900

C -5.57133800 -0.81305000 -3.70471200

C -4.36282800 -0.05450000 -3.81880200

C -3.05950200 -2.01456900 -3.17160800

C -0.56063100 -2.15666800 -3.54481500

C -19.59452500 2.71685600 2.92108200

C -18.44769700 1.97783100 3.34737200

C -18.28029700 3.79467200 1.17388600

C -17.09650000 3.41272600 1.86026800

C -15.81599900 3.66571200 1.26752600

C -14.62472400 3.32514100 1.97512900

C -14.47418300 3.82141000 -0.78179500

C -13.28194900 3.88444900 -0.00786400

C -12.01147100 3.81713500 -0.66210700

C -10.81754900 3.85652600 0.11640700

C -10.68079700 2.91574800 -2.52504300

C -9.48092000 3.35474500 -1.88978500

C -8.21537000 2.97106500 -2.43079900

C -7.01711300 3.38699500 -1.77923000

C -6.89247700 1.26097300 -3.60813500

C -5.68488700 1.95623800 -3.28052000

C -4.42305300 1.35509900 -3.56703800

C -3.22075000 2.02267600 -3.19387900

C -3.11150100 -0.72024200 -3.74659900

C -1.87828000 0.03079800 -3.80712100

C -0.62868600 -0.62936400 -3.83653300

C 0.55303100 0.13820600 -3.79340900

C 1.93407500 -1.82640900 -3.30201500

C 4.38545000 -1.73075800 -3.37994400

C -19.77512400 -0.09681000 3.90013900

C -18.53436700 0.60771100 3.79533200

C -17.35420500 -0.18347000 3.75938600

C -17.17994000 2.48236000 2.94690800

C -15.98269800 1.81140500 3.35824200

C -14.70733500 2.36116300 3.02560700

C -13.52293000 1.68039900 3.42574900

C -13.35691400 3.60927200 1.39174500

C -12.16412300 3.23128400 2.08177800

C -10.89325600 3.53538000 1.50643900

C -9.70405600 3.13570100 2.18345500

C -9.54994700 3.80924400 -0.53882300

C -8.35519100 3.82212900 0.24120100

C -7.08768300 3.79814600 -0.41442700

C -5.89439600 3.78914100 0.36775400

C -5.75000300 3.01877500 -2.32968800

C -4.55341900 3.41616200 -1.66338300

C -3.28798200 3.06116600 -2.21898400

C -2.09204700 3.44732000 -1.53909300

C -1.95159400 1.42206600 -3.48482200

C -0.75985800 2.10287300 -3.11338000

C 0.49717400 1.52325300 -3.43169700

C 1.69873700 2.19962200 -3.04548400

C 1.85282000 -0.49707200 -3.77814800

C 3.03854700 0.28311000 -3.80300200

C 4.31107600 -0.37139500 -3.78670800

C 5.51004500 0.40789700 -3.77723200

C 6.84540500 -1.63519900 -3.43965100

C 9.30907500 -1.52929300 -3.48838500

C -20.04029400 -3.47916400 1.41133000

C -19.86074800 -1.42349700 3.56204000

C -18.71301400 -2.12860400 3.07765000

C -17.44352300 -1.56210000 3.37899800

C -16.24821400 -2.22426100 2.95104900

C -16.07013100 0.45167800 3.74673900

C -14.88255800 -0.34191300 3.75011700

C -13.61011700 0.29768100 3.77278500

C -12.42187700 -0.49547500 3.73260800

C -12.24637300 2.23488500 3.09773700

C -11.05893800 1.53868800 3.47581600

C -9.78596800 2.10426300 3.16715500

C -8.59724200 1.39510900 3.51828800

C -8.43194100 3.45742000 1.61888800

C -7.24303600 3.03882800 2.28489900

C -5.97203500 3.38137800 1.73273500

C -4.78215800 2.94379800 2.38655000

C -4.62566000 3.78999300 -0.28566500

C -3.43541200 3.75986300 0.49723800

C -2.16528500 3.78107800 -0.15493200

C -0.97283300 3.73064200 0.62583200

C -0.82732800 3.12536200 -2.10949000

C 0.36454800 3.48322400 -1.42326500

C 1.63196400 3.17111300 -2.00616100

C 2.82846800 3.51919700 -1.30972700

C 2.96930800 1.65853200 -3.40928700

C 4.16246600 2.31158400 -2.98873900

C 5.43764700 1.76462800 -3.34939000

C 6.63256600 2.40553500 -2.90610400

C 6.77567900 -0.25242400 -3.79285000

C 7.97220400 0.52257600 -3.75232700

C 9.24072900 -0.13712300 -3.79078700

C 10.43701900 0.63612100 -3.72752000

C 10.57406400 -2.11493600 -3.18212600

C 11.77034600 -1.42349400 -3.53427400

C 13.03907600 -2.02001600 -3.24652500

C 14.23530800 -1.32080700 -3.57676600

C -20.11962600 -3.70387800 0.06258600

C -18.96417900 -3.54914300 -0.77293800

C -18.79840900 -3.10227700 2.01923600

C -17.61547400 -3.40509900 1.29101900

C -16.33414400 -3.14143100 1.87450400

C -15.14281500 -3.47656000 1.16001200

C -14.97114900 -1.69934900 3.31993100

C -13.78725200 -2.35559500 2.87837800

C -12.51011100 -1.83477000 3.25525700

C -11.32237000 -2.47628800 2.78870300

C -11.14541100 0.14498100 3.77559300

C -9.96024600 -0.64554900 3.70867200

C -8.68230200 -0.00704200 3.76946200

C -7.49594500 -0.79433800 3.67732700

C -7.32451000 1.97356300 3.23200100

C -6.13654900 1.25334000 3.55759700

C -4.86307800 1.84473700 3.29505200

C -3.67575000 1.11714000 3.59620600

C -3.51281700 3.30823700 1.84877600

C -2.32195900 2.84763500 2.48661900

C -1.05017200 3.23312200 1.96431200

C 0.13835000 2.75146400 2.58256300

C 0.29353000 3.77256300 -0.02548200

C 1.48750000 3.68758000 0.75069900

C 2.75645600 3.75401000 0.09791300

C 3.94757300 3.64424900 0.87125000

C 4.09404600 3.23968100 -1.90458200

C 5.29069500 3.55277300 -1.18977500

C 6.56093700 3.29750500 -1.79239900

C 7.75354500 3.58349600 -1.06544200

C 7.90147700 1.86560400 -3.27954800

C 9.09909300 2.48949000 -2.81182600

C 10.36757100 1.96433300 -3.20645200

C 11.56212000 2.56987000 -2.71655800

C 11.70270700 -0.02225200 -3.78689400

C 12.90189800 0.74890400 -3.69254200

C 14.16734800 0.09314200 -3.77751000

C 15.36171900 0.85396100 -3.64262700

C 15.50009500 -1.92422500 -3.30258000

C 16.70732900 -1.21642600 -3.60463600

C 17.98100500 -1.84557000 -3.40902300

C 19.13360800 -1.09348200 -3.71458600

C -20.27763800 -2.54275800 -2.67540000

C -19.03638600 -2.97475300 -2.08652300

C -17.83686800 -2.46826100 -2.66163600

C -17.69670100 -3.61639700 -0.12433200

C -16.49527500 -3.50942700 -0.89323500

C -15.22232800 -3.63910200 -0.25370000

C -14.03172100 -3.51594200 -1.02588800

C -13.87263000 -3.24269400 1.76246500

C -12.68187200 -3.53920300 1.03157000

C -11.40783200 -3.32296200 1.64278300

C -10.21962800 -3.59558800 0.90400200

C -10.04811500 -1.97010500 3.18494000

C -8.85930100 -2.59282300 2.69454000

C -7.58245500 -2.10252000 3.10984800

C -6.39645900 -2.70806200 2.59886700

C -6.22034600 -0.15672000 3.75687000

C -5.03072700 -0.93862000 3.63368300

C -3.75854700 -0.30216600 3.73896200

C -2.56949700 -1.07533600 3.57748600

C -2.40339900 1.71896000 3.35345800

C -1.21337000 0.98200300 3.63032800

C 0.05688500 1.58938900 3.41221700

C 1.24490900 0.83935600 3.65673000

C 1.40997100 3.14592100 2.06696100

C 2.60093500 2.63983400 2.66983600

C 3.87032400 3.05508500 2.17054100

C 5.05968700 2.52782800 2.75511300

C 5.21735100 3.73156600 0.22295900

C 6.40981700 3.59695900 0.99585000

C 7.67875000 3.71197100 0.35433400

C 8.87084500 3.54865000 1.12281000

C 9.02578600 3.34326800 -1.67289900

C 10.21907700 3.60778400 -0.93598900

C 11.48860600 3.38672800 -1.55003400

C 12.68297000 3.62474200 -0.80241700

C 12.83288500 2.05502600 -3.12580600

C 14.02762200 2.64561200 -2.61758500

C 15.29454500 2.13930200 -3.03597700

C 16.49170200 2.70974600 -2.50093000

C 16.63353200 0.19863900 -3.74094000

C 17.82358900 0.96134700 -3.59831900

C 19.08375100 0.30687300 -3.75492500

C 20.26560000 1.11771300 -3.62010600

C -16.56562800 -2.90524100 -2.17305700

C -15.36611700 -2.40172000 -2.76645600

C -14.10161100 -2.86650200 -2.29614000

C -12.90388700 -2.34026000 -2.86563100

C -12.76077600 -3.65342400 -0.38633500

C -11.56627400 -3.50154600 -1.15463600

C -10.29827300 -3.66187000 -0.51952900

C -9.10393200 -3.48204300 -1.28020900

C -8.94531400 -3.39810200 1.52168900

C -7.75505700 -3.64890800 0.77536400

C -6.48359500 -3.47483600 1.40080300

C -5.29156600 -3.69730400 0.64329800

C -5.11414400 -2.22769700 3.02342000

C -3.92886300 -2.81460700 2.49485200

C -2.65124700 -2.33292300 2.91339700

C -1.45770800 -2.89286500 2.35362500

C -1.29541300 -0.44182800 3.71721300

C -0.11044800 -1.20731300 3.54330000

C 1.16059700 -0.58314000 3.69638600

C 2.34975100 -1.34947500 3.49271800

C 2.51926400 1.45328700 3.45829900

C 3.70526100 0.69626200 3.68394200

C 4.97773100 1.31697600 3.50443600

C 6.16757200 0.55474800 3.70839400

C 6.33165000 2.96251500 2.27238500

C 7.51988700 2.42054200 2.84270900

C 8.79183400 2.87140800 2.37512000

C 9.98257100 2.31329700 2.93035600

C 10.14266800 3.68894600 0.48768900

C 11.33225600 3.50156000 1.25039900

C 12.60532900 3.65966900 0.61948600

C 13.79740100 3.44956200 1.37722500

C 13.95448400 3.42678700 -1.42387300

C 15.14466100 3.63624000 -0.66987900

C 16.41850900 3.44595800 -1.29253000

C 17.61948600 3.63976500 -0.54056100

C 17.76483200 2.23024800 -2.94218300

C 18.96184600 2.80111800 -2.42535900

C 20.20514400 2.31593700 -2.96758800

C -11.63620700 -2.81410400 -2.40301200

C -10.43955200 -2.27031300 -2.95545000

C -9.17345400 -2.75499700 -2.50451200

C -7.97463200 -2.19301000 -3.03940200

C -7.83456900 -3.66541400 -0.65038700

C -6.64347900 -3.45884400 -1.40274000

C -5.37496000 -3.65689600 -0.77451000

C -4.18077900 -3.41645600 -1.51579500

C -4.01270000 -3.55384700 1.27100200

C -2.83353700 -3.74473000 0.50395600

C -1.52800900 -3.60461300 1.11223400

C -0.35292000 -3.85755200 0.37444500

C -0.19314400 -2.44359100 2.82027900

C 0.99040800 -2.99030500 2.25282100

C 2.26729000 -2.56132300 2.74621900

C 3.46187600 -3.10642600 2.19229300

C 3.62190100 -0.73095300 3.68197500

C 4.81127500 -1.48656400 3.46312200

C 6.08583300 -0.86913100 3.66572900

C 7.27694300 -1.61552200 3.42596000

C 7.43877100 1.18452100 3.55361400

C 8.62894500 0.41841000 3.73277200

C 9.90211200 1.05453000 3.59973700

C 11.09144500 0.28473700 3.75558200

C 11.25279100 2.78180800 2.48068500

C 12.44396300 2.20313000 3.01520300

C 13.71754100 2.68816100 2.58326000

C 14.90509300 2.09278800 3.09483000

C 15.06637900 3.62445300 0.75278700

C 16.25981000 3.37500600 1.49842900

C 17.53968300 3.58109800 0.88922800

C 18.72437000 3.36603600 1.64507600

C 18.88721900 3.50987300 -1.17941600

C 20.04238300 3.75741500 -0.36620900

C -6.71223200 -2.69071700 -2.60329500

C -5.51427400 -2.10436100 -3.11497400

C -4.24870800 -2.60936400 -2.68907600

C -2.92311700 -3.63052100 -0.90339200

C -1.72729300 -3.34289800 -1.64620800

C -0.43291200 -3.97330700 -1.17626700

C 0.79947300 -3.31219800 -1.76336000

C 0.90263300 -3.66585700 0.99526700

C 2.13017100 -3.81437000 0.24615700

C 3.38722400 -3.71945900 0.89865600

C 4.58927500 -3.81783600 0.12816400

C 4.73081300 -2.68124500 2.68684800

C 5.93112300 -3.18955000 2.09337700

C 7.19936300 -2.78245600 2.60439100

C 8.39447900 -3.26259300 1.98706900

C 8.54857300 -1.00249900 3.64392900

C 9.74297000 -1.73950700 3.37524400

C 11.01234900 -1.13478700 3.61707200

C 12.20534400 -1.86028100 3.31738600

C 12.36377000 0.92525600 3.64005700

C 13.55691900 0.14978300 3.77009700

C 14.82494400 0.79181100 3.67550400

C 16.01775500 0.01063500 3.76134300

C 16.17918900 2.58167700 2.66951200

C 17.37836200 1.97968900 3.16932100

C 18.64445100 2.51649800 2.80565800

C 19.79661100 1.87750500 3.36562600

C 19.96445800 3.67940400 0.99880200

C -1.79137000 -2.54113000 -2.74547900

C 0.73583300 -2.45819100 -2.82250700

C 2.06990300 -3.59768300 -1.15300700

C 3.25669400 -3.29787100 -1.86210500

C 4.52373600 -3.58696300 -1.27205300

C 5.71952600 -3.25688300 -1.98134000

C 5.85973400 -3.74376400 0.78156300

C 7.05296000 -3.82788800 0.00515500

C 8.32165200 -3.77126000 0.65792400

C 9.51803400 -3.83235200 -0.12143900

C 9.66656700 -2.87377400 2.51219300

C 10.85870500 -3.33590100 1.88156900

C 12.12887600 -2.96146700 2.41720200

C 13.32475200 -3.40297400 1.77005900

C 13.47841200 -1.26400100 3.58089700

C 14.66739100 -1.97630300 3.25705500

C 15.93918900 -1.38438300 3.52608800

C 17.14085100 -2.08837700 3.18947600

C 17.29762300 0.65201800 3.70281200

C 18.48274000 -0.12270600 3.82604900

C 19.71926100 0.59658200 3.84999000

C 3.18934700 -2.37368400 -2.94595300

C 5.65156600 -2.29730200 -3.03471000

C 6.98357100 -3.55885600 -1.39658000

C 8.17908400 -3.20054100 -2.08835300

C 9.44747600 -3.51983400 -1.51191300

C 10.64190700 -3.14424500 -2.19316700

C 10.78529700 -3.80203200 0.53274000

C 11.97931000 -3.83441300 -0.24782800

C 13.25112100 -3.82415000 0.40744700

C 14.44201700 -3.83472600 -0.37062400

C 14.59082000 -3.04850400 2.31682700

C 15.78454500 -3.45332300 1.64744400

C 17.06354700 -3.12972000 2.20803300

C 18.24970300 -3.57357900 1.56447700

C 18.40505600 -1.53453500 3.53145600

C 19.55692300 -2.30748500 3.18609600

C 8.11093900 -2.20751800 -3.10883100

C 11.90850500 -3.47915400 -1.62572400

C 13.10681500 -3.08038200 -2.29508600

C 14.37244400 -3.43596200 -1.74244400

C 15.56624900 -3.00768000 -2.38834300

C 15.71008600 -3.82992400 0.28335800

C 16.91493600 -3.82882900 -0.49275400

C 18.17539700 -3.91326300 0.15940800

C 19.32609200 -3.94771700 -0.68316400

C 19.48262500 -3.28851300 2.22763200

C 16.84571000 -3.38455400 -1.85367400

C 18.03669200 -3.00456200 -2.52443800

C 19.25829100 -3.50066700 -1.98238800

H 21.01630000 3.87263200 -0.83110300

H 20.87835700 3.73548200 1.58204200

H 20.77154400 2.34643700 3.27832500

H 20.63477900 0.08765000 4.13530400

H 20.53199900 -2.02728000 3.57176600

H 20.40090900 -3.75594700 1.88585600

H 20.29861200 -4.18631400 -0.26429900

H 20.18003200 -3.40330600 -2.54770900

H 21.22639100 0.70619700 -3.91913000

H 21.12601100 2.85107300 -2.75780300

H 20.11307200 -1.55914200 -3.66503700

H -20.32704400 4.22616700 -0.71021300

H -20.21756700 3.19487800 -2.89306900

H -20.16429200 1.23380500 -3.79474300

H -21.29144300 -1.03852600 -3.79288100

H -21.20136600 -3.04587800 -2.40722800

H -21.09439100 -3.86477600 -0.38669400

H -20.95401500 -3.46787700 1.99749000

H -20.83861700 -1.89322700 3.52764900

H -20.68734000 0.44596100 4.12760300

H -20.57123800 2.48596700 3.33431900

H -20.42998200 4.02380000 1.47378700

C -0.54199600 -3.12783700 -4.77521700

C -0.17558800 -4.65490500 -4.48464800

C -0.84514500 -5.52443800 -3.34390100

C -0.46837800 -5.41343400 -1.79570900

F -1.40313400 -6.17277900 -1.15943800

F 0.72735400 -6.03734000 -1.63250300

F -2.19120400 -5.39801700 -3.47948700

F -0.52408400 -6.80482700 -3.66685300

F -0.51061200 -5.29646800 -5.63482400

F 1.17071200 -4.76420500 -4.33774200

F 0.38865900 -2.71659400 -5.68056800

F -1.74229200 -3.12272500 -5.41129700

**(6,5) SWNT>(CF_2_)_4_ (1,4-L_+_: L_27_)**

C -21.14738600 -1.98262100 -1.93799200

C -19.96037300 -1.19824200 -2.15755000

C -20.00210700 0.19812800 -2.27168800

C -18.84567900 0.97261100 -2.04738500

C -18.70416200 -1.83844500 -1.92696900

C -17.50896200 -1.10302900 -2.14874500

C -16.24102000 -1.75040600 -1.97616400

C -15.04077300 -1.01579800 -2.18837500

C -20.11773500 2.78106400 -0.81434500

C -18.89737900 2.22189500 -1.29574900

C -17.70613800 2.66712500 -0.66750300

C -17.57488900 0.31878000 -2.16866700

C -16.36432800 1.04831000 -1.94198900

C -15.10132400 0.41167000 -2.14301200

C -13.90199200 1.13622000 -1.88793700

C -13.77902000 -1.66505700 -2.02914700

C -12.57414300 -0.91538200 -2.19845700

C -11.31103100 -1.57001000 -2.06189100

C -10.10950600 -0.81467600 -2.19814200

C -20.35251800 3.02437800 3.39278100

C -20.18634200 3.36745100 0.42810300

C -19.03776000 3.41791400 1.27327600

C -17.77642100 3.25756500 0.63692300

C -16.57382900 3.33693300 1.41177200

C -16.42753300 2.22661300 -1.15371500

C -15.23323300 2.71796500 -0.55560700

C -13.96781400 2.29700800 -1.06159100

C -12.77029700 2.76356300 -0.43811400

C -12.63492600 0.50506600 -2.10144300

C -11.43573100 1.22571800 -1.82375100

C -10.17119800 0.60410100 -2.05506100

C -8.97221000 1.31700600 -1.75088300

C -8.84428800 -1.47215800 -2.07553400

C -7.64263800 -0.71012500 -2.18674200

C -6.38034400 -1.36795300 -2.07809700

C -5.17867800 -0.60378700 -2.15486700

C -3.85256000 -2.59140700 -1.53073700

C -1.40063200 -2.51600100 -1.58776900

C -20.43332800 2.15324200 4.45129400

C -19.28572800 1.41669600 4.88118600

C -19.11660500 3.23114700 2.70615500

C -17.93388500 2.85363100 3.39717400

C -16.65266500 3.10920800 2.80821900

C -15.46212500 2.77200600 3.52020300

C -15.30441400 3.26506600 0.76365300

C -14.11573200 3.33466900 1.54158200

C -12.84346900 3.26916800 0.89168300

C -11.65183900 3.31734200 1.67464000

C -11.50300100 2.36117000 -0.95986500

C -10.30940900 2.81043900 -0.32403200

C -9.04080800 2.42425900 -0.85583300

C -7.84641000 2.85962700 -0.20744000

C -7.70561100 0.70483800 -2.00163300

C -6.50977400 1.40939800 -1.67739300

C -5.24287100 0.80234400 -1.93823900

C -4.04593600 1.50051400 -1.59723000

C -3.91421100 -1.26550200 -2.06096100

C -2.71951300 -0.50494300 -2.12503700

C -1.45891600 -1.17536900 -2.05103700

C -0.25897800 -0.39457500 -2.11954200

C 1.07984500 -2.40367300 -1.68311700

C 3.54682600 -2.29727700 -1.79454100

C -20.61133100 -0.65956500 5.43041300

C -19.37104800 0.04661200 5.32786000

C -18.18965800 -0.74311000 5.29224000

C -18.01791700 1.92371900 4.48427500

C -16.82043800 1.25422900 4.89749700

C -15.54496500 1.80663200 4.56843800

C -14.35932900 1.12690700 4.96912800

C -14.19364300 3.06116300 2.94152800

C -13.00187100 2.68536600 3.63449500

C -11.72957700 2.99584100 3.06377100

C -10.54022200 2.59847200 3.74216900

C -10.38312100 3.27586500 1.02329700

C -9.19074600 3.29709900 1.80682300

C -7.92038900 3.28184800 1.15351400

C -6.72841000 3.28174500 1.93634100

C -6.57851900 2.49400700 -0.75139700

C -5.38479200 2.90908800 -0.08928200

C -4.11469300 2.55958500 -0.64390900

C -2.92053300 2.95464700 0.02777700

C -2.78210700 0.90193700 -1.87733500

C -1.58773700 1.58635800 -1.51861300

C -0.32232600 0.99277300 -1.82273200

C 0.87739800 1.67563300 -1.45674000

C 1.00831600 -1.04922200 -2.11322100

C 2.20448900 -0.27205700 -2.16127000

C 3.47418200 -0.92855600 -2.17409400

C 4.67367100 -0.14995900 -2.17697900

C 6.00970700 -2.19350100 -1.86465900

C 8.47548500 -2.08596600 -1.92179600

C -20.87024500 -4.03449300 2.93135300

C -20.69524400 -1.98522700 5.08886700

C -19.54613200 -2.68771200 4.60330300

C -18.27733500 -2.12092800 4.90818200

C -17.08103600 -2.78092500 4.48072300

C -16.90647600 -0.10640600 5.28255300

C -15.71730500 -0.89864200 5.28436200

C -14.44499700 -0.25766500 5.31047200

C -13.25580500 -1.04876000 5.26759900

C -13.08382600 1.68490900 4.64530400

C -11.89379500 0.98744600 5.01966500

C -10.62089000 1.55843700 4.71760800

C -9.43101400 0.84705700 5.05960700

C -9.26842600 2.92779200 3.18151100

C -8.07652200 2.50608400 3.84498000

C -6.80552300 2.86174700 3.29855200

C -5.61427200 2.41461100 3.94229100

C -5.45846800 3.28871100 1.28294100

C -4.26446200 3.26013200 2.06616700

C -2.99268000 3.29020900 1.41428400

C -1.80002100 3.23023600 2.19298400

C -1.65332700 2.61747700 -0.53687600

C -0.45567200 2.98377300 0.14818700

C 0.81162300 2.66639900 -0.43260500

C 2.00786100 3.00751500 0.26395100

C 2.14027700 1.10535900 -1.79552200

C 3.34068500 1.76590800 -1.39237100

C 4.60731300 1.21022600 -1.75292000

C 5.80558900 1.85275400 -1.32003700

C 5.93949400 -0.80874600 -2.20534900

C 7.13859500 -0.03295500 -2.17036000

C 8.40651100 -0.69163800 -2.21784800

C 9.60412700 0.08124000 -2.15613800

C 9.74217800 -2.67130300 -1.61992500

C 10.93837000 -1.97858700 -1.97262700

C 12.20777400 -2.57538300 -1.68831300

C 13.40368500 -1.87570300 -2.01909400

C -20.94632800 -4.25906300 1.58249300

C -19.78847400 -4.10600800 0.74965800

C -19.62951200 -3.65872600 3.54284300

C -18.44484100 -3.96160400 2.81729500

C -17.16515600 -3.69779300 3.40368000

C -15.97247900 -4.03360100 2.69177500

C -15.80456700 -2.25459600 4.85132700

C -14.61915700 -2.90996200 4.41130100

C -13.34330700 -2.38780200 4.78852100

C -12.15478300 -3.02906000 4.32215000

C -11.97926500 -0.40663000 5.31056400

C -10.79131400 -1.19579600 5.24050800

C -9.51599400 -0.55589000 5.29903700

C -8.32658200 -1.34348900 5.19260900

C -8.15655300 1.42938200 4.77632800

C -6.96567200 0.70522700 5.08714100

C -5.69440500 1.29824800 4.82575600

C -4.50390300 0.55720700 5.09727700

C -4.34216200 2.79042900 3.40882300

C -3.14988400 2.31997600 4.03725900

C -1.87769700 2.71349900 3.52219600

C -0.68904400 2.21615500 4.13327400

C -0.52817200 3.27139000 1.54158500

C 0.66571900 3.17991500 2.32039100

C 1.93566400 3.24365700 1.67016200

C 3.12744600 3.12314400 2.44476700

C 3.27444100 2.71043000 -0.32689400

C 4.47167900 3.02486800 0.38494300

C 5.73823500 2.75506000 -0.21609400

C 6.93346900 3.04169600 0.50828000

C 7.07191900 1.30973100 -1.69692600

C 8.27129900 1.93540700 -1.23659600

C 9.53761300 1.40871300 -1.63368800

C 10.73445400 2.01476100 -1.14761200

C 10.87027200 -0.57676200 -2.22122300

C 12.07019000 0.19403700 -2.12881300

C 13.33553300 -0.46156900 -2.21755000

C 14.53085600 0.29886200 -2.08407500

C 14.66917500 -2.47941400 -1.74716400

C 15.87602200 -1.77181900 -2.05085300

C 17.14992100 -2.40120500 -1.85707100

C 18.30222900 -1.64949400 -2.16502900

C -21.09560300 -3.10298200 -1.15901000

C -19.85634700 -3.53407700 -0.56483500

C -18.65465300 -3.02894000 -1.13725000

C -18.52269800 -4.17388200 1.40175200

C -17.31959800 -4.06930400 0.63573900

C -16.04866100 -4.19911700 1.27881100

C -14.85586300 -4.07992800 0.50873100

C -14.70342600 -3.79942300 3.29669600

C -13.51273000 -4.09985000 2.56941100

C -12.24043900 -3.88084800 3.18165800

C -11.05134900 -4.16308100 2.44614800

C -10.87962600 -2.52184200 4.71744200

C -9.69307000 -3.14682700 4.22980900

C -8.41691000 -2.64799000 4.63252600

C -7.22823400 -3.25770100 4.11789000

C -7.04844200 -0.70797100 5.26665500

C -5.86211200 -1.48766500 5.11430000

C -4.58585200 -0.85631300 5.20718500

C -3.39609700 -1.63666200 5.03404500

C -3.23013200 1.16844600 4.87294700

C -2.04483000 0.42708600 5.13618900

C -0.77265000 1.04031000 4.93623300

C 0.41517000 0.28764700 5.17981400

C 0.58623000 2.62075800 3.62970500

C 1.77404100 2.10489100 4.22837700

C 3.04730700 2.52336800 3.73661400

C 4.23611200 1.98947900 4.31962500

C 4.39851100 3.20893800 1.79737800

C 5.58964700 3.06669300 2.56905200

C 6.85949300 3.17579100 1.92614900

C 8.05147800 3.00891500 2.69386900

C 8.20232000 2.79468400 -0.10081200

C 9.39685100 3.05886600 0.63327400

C 10.66456400 2.83346800 0.01741500

C 11.86015900 3.07175200 0.76264900

C 12.00353600 1.49973900 -1.56013700

C 13.19979700 2.08965600 -1.05417900

C 14.46571500 1.58323900 -1.47546300

C 15.66424800 2.15286900 -0.94220200

C 15.80231700 -0.35655100 -2.18592800

C 16.99302900 0.40552100 -2.04449300

C 18.25271200 -0.24921600 -2.20433800

C 19.43519400 0.56100800 -2.07096700

C -17.38544200 -3.46642300 -0.64504200

C -16.18360900 -2.96314600 -1.23475400

C -14.92060500 -3.43041000 -0.76212400

C -13.72065300 -2.90499300 -1.32674900

C -13.58810700 -4.21946300 1.15106700

C -12.39177100 -4.07175300 0.38452600

C -11.12556500 -4.23868200 1.02192000

C -9.93018400 -4.07122600 0.26287700

C -9.78121600 -3.96447700 3.06290900

C -8.59439700 -4.22523100 2.31630600

C -7.32167900 -4.04764900 2.93800600

C -6.13736200 -4.32071300 2.20276600

C -5.95099400 -2.77076400 4.50603200

C -4.76447600 -3.37718800 3.95871900

C -3.47162700 -2.90227900 4.39464800

C -2.28278800 -3.49135800 3.87330100

C -2.12628900 -0.99908300 5.19503800

C -0.94154600 -1.75984100 5.02849600

C 0.33203500 -1.13371100 5.20739600

C 1.52346600 -1.89623600 5.02777400

C 1.69028700 0.90677600 5.00057900

C 2.87682100 0.14960200 5.22594700

C 4.15223000 0.77256000 5.05805700

C 5.34117400 0.00979600 5.26247700

C 5.50953400 2.42607600 3.84236300

C 6.69743000 1.87808300 4.40965300

C 7.97123600 2.32950000 3.94484000

C 9.16100800 1.76771100 4.49751300

C 9.32221800 3.14427300 2.05653400

C 10.51246300 2.95421000 2.81793700

C 11.78460200 3.10930700 2.18472000

C 12.97701600 2.89771400 2.94082600

C 13.12984200 2.87119500 0.13937100

C 14.32153100 3.08001500 0.89152800

C 15.59395800 2.88844800 0.26685300

C 16.79622800 3.08100800 1.01704300

C 16.93623200 1.67336400 -1.38627500

C 18.13465300 2.24290400 -0.87071600

C 19.37661400 1.75813700 -1.41632700

C -12.45522200 -3.38283500 -0.86177200

C -11.25284100 -2.83950000 -1.40729400

C -9.99156500 -3.33306100 -0.95868100

C -8.78604400 -2.76195600 -1.47113300

C -8.66650000 -4.26284600 0.89637500

C -7.46100200 -4.07403500 0.14841500

C -6.20878700 -4.31033900 0.76920000

C -4.99830900 -4.04979100 0.05180700

C -4.87408700 -4.18901700 2.83024400

C -3.66381900 -4.78657800 2.10799100

C -2.32831900 -4.29558700 2.68544700

C -1.16510500 -4.47747900 1.95569900

C -1.02050400 -3.00230100 4.32599300

C 0.17790400 -3.55329700 3.79313200

C 1.44894900 -3.11552900 4.29097200

C 2.64028400 -3.66116200 3.74171100

C 2.79459900 -1.27583500 5.22042400

C 3.98644100 -2.03211400 5.00734200

C 5.26010100 -1.41480700 5.21484900

C 6.45127600 -2.16128200 4.97690400

C 6.61430800 0.63895800 5.11291600

C 7.80479400 -0.12895300 5.29111900

C 9.07896500 0.50642600 5.16232100

C 10.26806600 -0.26497500 5.31595300

C 10.43239200 2.23468400 4.04757800

C 11.62337500 1.65349600 4.58035400

C 12.89716500 2.13705600 4.14730400

C 14.08465800 1.53982900 4.65712300

C 14.24530800 3.06978400 2.31407700

C 15.43936500 2.81946900 3.05832900

C 16.71847200 3.02314800 2.44697600

C 17.90399100 2.80703900 3.20136900

C 18.06285400 2.95002600 0.37618800

C 19.21958300 3.19578100 1.18783300

C -7.52261300 -3.27254100 -1.03778900

C -6.32321000 -2.67496700 -1.51022500

C -5.05882900 -3.19203900 -1.07548300

C -3.73053500 -4.37482200 0.61889700

C -2.56417500 -4.11294900 -0.08183400

C -1.23548600 -4.69212700 0.42548700

C -0.01710200 -4.02040200 -0.21230000

C 0.10670300 -4.25059200 2.56020600

C 1.31393200 -4.43150100 1.81387600

C 2.56855900 -4.29686900 2.45957300

C 3.77220400 -4.40149000 1.69138000

C 3.91069800 -3.22922100 4.23682700

C 5.10825100 -3.74446000 3.65280300

C 6.37540400 -3.33088100 4.16012500

C 7.57145700 -3.81480800 3.54664700

C 7.72430000 -1.54918500 5.19820200

C 8.91798300 -2.28724000 4.93220400

C 10.18813800 -1.68371000 5.17463900

C 11.38122000 -2.41031400 4.87543400

C 11.54185400 0.37463300 5.20269000

C 12.73430400 -0.40201600 5.33122000

C 14.00334300 0.23882600 5.23683700

C 15.19571700 -0.54352600 5.32139700

C 15.35900700 2.02728700 4.23018100

C 16.55795300 1.42425000 4.72880500

C 17.82433800 1.95922600 4.36301200

C 18.97638900 1.31961000 4.92277600

C 19.14360800 3.11809100 2.55295700

C -2.59763900 -3.15421600 -1.14938800

C -0.11470000 -3.06245000 -1.22130800

C 1.24359800 -4.25068500 0.39105400

C 2.43165400 -3.89770900 -0.30249200

C 3.70159300 -4.17370300 0.28892100

C 4.89183800 -3.83211600 -0.41797200

C 5.03753100 -4.31347900 2.34344000

C 6.23048300 -4.39335200 1.56717300

C 7.49904500 -4.32994600 2.21975500

C 8.69330400 -4.39128700 1.43995700

C 8.84204400 -3.42359900 4.07047900

C 10.03415100 -3.88651800 3.44045500

C 11.30455300 -3.51150800 3.97535500

C 12.49963800 -3.95416900 3.32817200

C 12.65470600 -1.81559800 5.13969000

C 13.84333700 -2.52844100 4.81468600

C 15.11594800 -1.93808300 5.08425500

C 16.31690700 -2.64266400 4.74629600

C 16.47616800 0.09674400 5.26261500

C 17.66073100 -0.67908500 5.38447700

C 18.89806300 0.03913100 5.40783500

C 2.34976700 -2.95530900 -1.36555100

C 4.81564100 -2.86330500 -1.46529100

C 6.15807700 -4.12707300 0.16604800

C 7.35178500 -3.76413800 -0.52678500

C 8.62037500 -4.08103900 0.04816000

C 9.81324700 -3.70215800 -0.63325300

C 9.96052600 -4.35609700 2.09321000

C 11.15365100 -4.38920800 1.31150400

C 12.42530100 -4.37708200 1.96585300

C 13.61527400 -4.38786100 1.18670700

C 13.76608600 -3.59977100 3.87384200

C 14.95930200 -4.00525600 3.20355400

C 16.23866400 -3.68267300 3.76342400

C 17.42423900 -4.12639400 3.11851500

C 17.58176800 -2.09030200 5.08841100

C 18.73303300 -2.86363300 4.74143100

C 7.27863400 -2.76664800 -1.54274700

C 11.08092100 -4.03579100 -0.06676600

C 12.27768500 -3.63640700 -0.73729200

C 13.54399100 -3.99073600 -0.18534300

C 14.73696300 -3.56257900 -0.83280100

C 14.88387300 -4.38222000 1.83966500

C 16.08777300 -4.38159600 1.06239700

C 17.34886600 -4.46536100 1.71344100

C 18.49879500 -4.49986400 0.86968300

C 18.65773100 -3.84299500 3.78146300

C 16.01694500 -3.93876600 -0.29902400

C 17.20707200 -3.55917800 -0.97140700

C 18.42949800 -4.05412700 -0.42987300

H 20.19299700 3.30951400 0.72152500

H 20.05849400 3.17287800 3.13477600

H 19.95165000 1.78775200 4.83481900

H 19.81316200 -0.47057600 5.69310900

H 19.70840200 -2.58476500 5.12732200

H 19.57550300 -4.31074400 3.43872500

H 19.47184100 -4.73771800 1.28774900

H 19.35066100 -3.95690300 -0.99615700

H 20.39516600 0.14954200 -2.37268000

H 20.29811600 2.29262500 -1.20765300

H 19.28161800 -2.11553500 -2.11761800

H -21.15837000 3.65703000 0.81470000

H -21.03854000 2.62857900 -1.36893200

H -20.97861700 0.67255800 -2.27712600

H -22.10502800 -1.60021600 -2.28234100

H -22.02019000 -3.60630000 -0.89413200

H -21.92007600 -4.41923600 1.13073900

H -21.78546600 -4.02277100 3.51514200

H -21.67238300 -2.45638900 5.05328000

H -21.52408900 -0.11828700 5.65925200

H -21.41046100 1.92092500 4.86271400

H -21.26782600 3.45637800 3.00011800

C -1.15338400 -6.21820500 -0.01599800

C -1.71416300 -7.32865500 0.91755400

C -3.21273500 -7.34539300 1.28775300

C -3.76344500 -6.35569900 2.35314300

F -5.06941500 -6.73681500 2.45376800

F -3.17770300 -6.65259800 3.54485600

F -3.94010700 -7.19689800 0.14989900

F -3.45437000 -8.58516800 1.78667100

F -1.48313000 -8.49758800 0.26606700

F -0.98671300 -7.33151800 2.06528200

F -1.74259600 -6.35700600 -1.23452100

F 0.14850600 -6.59810200 -0.16493900

**(6,5) SWNT>(CF_2_)_4_ (1,4- L–: L_–33_)**

C -20.38855600 -1.52426000 -3.51124900

C -19.20262300 -0.73960600 -3.73505700

C -19.24529700 0.65677900 -3.85127900

C -18.08872400 1.43218900 -3.63189000

C -17.94559100 -1.37875000 -3.50685100

C -16.75146100 -0.64281500 -3.73296200

C -15.48263400 -1.28904900 -3.56274200

C -14.28402400 -0.55411600 -3.78035000

C -19.35755100 3.24217600 -2.39803500

C -18.13859200 2.68278800 -2.88192200

C -16.94577300 3.12828700 -2.25667000

C -16.81802000 0.77874500 -3.75559800

C -15.60695900 1.50944100 -3.53417900

C -14.34470900 0.87336000 -3.73841900

C -13.14449800 1.59876600 -3.48750100

C -13.02133400 -1.20216600 -3.62258800

C -11.81743400 -0.45249800 -3.80025000

C -10.55470800 -1.10615500 -3.66623800

C -9.35275900 -0.35065100 -3.81088000

C -19.58327500 3.48430900 1.80905000

C -19.42318500 3.82897000 -1.15543000

C -18.27288800 3.87904200 -0.31297200

C -17.01304000 3.71832600 -0.95203900

C -15.80844900 3.79696900 -0.17983100

C -15.66817200 2.68828100 -2.74620800

C -14.47246600 3.17937600 -2.15101700

C -13.20817200 2.75851500 -2.66019800

C -12.00831100 3.22383200 -2.03894900

C -11.87771100 0.96844700 -3.70566800

C -10.67761600 1.68863800 -3.43117100

C -9.41331900 1.06667900 -3.66660300

C -8.21135100 1.77762800 -3.36370500

C -8.08714100 -1.00836700 -3.69552400

C -6.88495700 -0.24832700 -3.81157300

C -5.62117500 -0.90944800 -3.70958400

C -4.41444800 -0.14621800 -3.78684700

C -3.09409400 -2.13410600 -3.17205800

C -0.61245200 -2.05480400 -3.19593000

C -19.66215100 2.61306200 2.86786100

C -18.51385700 1.87721900 3.29617800

C -18.34883600 3.69147500 1.12035600

C -17.16490700 3.31329900 1.80856400

C -15.88457700 3.56865300 1.21689300

C -14.69301300 3.23185900 1.92641200

C -14.54072100 3.72525200 -0.83073100

C -13.35030700 3.79293500 -0.05530900

C -12.07890600 3.72718600 -0.70785900

C -10.88616000 3.77408700 0.07253600

C -10.74261700 2.82197000 -2.56418100

C -9.54681800 3.26709100 -1.92845300

C -8.27842900 2.88105100 -2.46322400

C -7.08318400 3.31177800 -1.81478400

C -6.94623500 1.16512600 -3.61921400

C -5.74713900 1.86543900 -3.28994200

C -4.47877200 1.25889800 -3.55552300

C -3.28208300 1.95291300 -3.20780700

C -3.15133600 -0.80890400 -3.69139200

C -1.94968300 -0.04609500 -3.73519300

C -0.68233000 -0.71611600 -3.65438100

C 0.51362500 0.06713200 -3.71832000

C 1.84129100 -1.94250500 -3.24851200

C 4.30680900 -1.83433600 -3.35549100

C -19.83775100 -0.19913500 3.85031300

C -18.59803000 0.50732300 3.74506900

C -17.41658900 -0.28189500 3.70890900

C -17.24695600 2.38389500 2.89620600

C -16.04854900 1.71521500 3.30835900

C -14.77393100 2.26754100 2.97676800

C -13.58822300 1.58894700 3.37678300

C -13.42590100 3.51949700 1.34457400

C -12.23262400 3.14451600 2.03610700

C -10.96202100 3.45407200 1.46304300

C -9.77250400 3.05748900 2.14054400

C -9.61878300 3.72970000 -0.58081000

C -8.42455200 3.75031100 0.20153400

C -7.15620300 3.73326800 -0.45257200

C -5.96437800 3.73175800 0.33018400

C -5.81605000 2.94302500 -2.35882600

C -4.62031400 3.35750000 -1.69560400

C -3.35231900 3.01018500 -2.25046800

C -2.15964800 3.40655500 -1.57527700

C -2.01634300 1.35094000 -3.47976400

C -0.81917000 2.04568800 -3.11936100

C 0.44513800 1.46328800 -3.42685800

C 1.63976800 2.14473900 -3.05623600

C 1.77223700 -0.58456700 -3.69342700

C 2.96912200 0.19244500 -3.73457800

C 4.23536000 -0.46557800 -3.74487500

C 5.43177700 0.31041800 -3.75769700

C 6.76675400 -1.73467600 -3.45274500

C 9.23209100 -1.62748200 -3.52594900

C -20.09879900 -3.57763900 1.35705500

C -19.92151900 -1.52558600 3.51124300

C -18.77282200 -2.22834000 3.02552500

C -17.50402200 -1.66041800 3.32742100

C -16.30788400 -2.32032900 2.89871500

C -16.13350200 0.35531100 3.69623200

C -14.94426000 -0.43630200 3.69864000

C -13.67270400 0.20522000 3.72165900

C -12.48298900 -0.58568600 3.67902800

C -12.31284800 2.14651600 3.04980000

C -11.12296500 1.45182800 3.42610600

C -9.85193000 2.02150900 3.11977500

C -8.66061200 1.31220200 3.46524300

C -8.50087500 3.38487400 1.57750500

C -7.30957800 2.96839800 2.24301000

C -6.04075200 3.31849500 1.69320600

C -4.84750400 2.87709000 2.34220300

C -4.69421800 3.73747400 -0.32342200

C -3.50149000 3.71581300 0.45985400

C -2.23317000 3.74360200 -0.19259100

C -1.03702000 3.69091700 0.58967000

C -0.88949600 3.07420100 -2.14011600

C 0.30722800 3.45412600 -1.45776400

C 1.57178700 3.14192100 -2.03530700

C 2.77012600 3.48463700 -1.33756300

C 2.90363600 1.57076000 -3.38463400

C 4.10429700 2.23620200 -2.98810000

C 5.36656100 1.67666600 -3.34525600

C 6.56608300 2.31965200 -2.91685800

C 6.69677000 -0.34950800 -3.78672500

C 7.89617200 0.42743100 -3.76158500

C 9.16218300 -0.23014100 -3.81577700

C 10.36022900 0.54369300 -3.75765700

C 10.50339600 -2.21085500 -3.22988900

C 11.69725400 -1.51559200 -3.58466900

C 12.96855400 -2.11014800 -3.30427800

C 14.16302700 -1.40807700 -3.63682600

C -20.17746000 -3.80197700 0.00826000

C -19.02155500 -3.64731100 -0.82679000

C -18.85723600 -3.20064400 1.96588700

C -17.67387300 -3.50227100 1.23808200

C -16.39302200 -3.23733700 1.82208000

C -15.20125200 -3.57188800 1.10812700

C -15.03113000 -1.79352800 3.26746000

C -13.84645100 -2.44802600 2.82597100

C -12.56982000 -1.92476900 3.20145700

C -11.38117200 -2.56524400 2.73477600

C -11.20669200 0.05676400 3.72152100

C -10.02017600 -0.73099000 3.65196200

C -8.74353900 -0.09014200 3.70951500

C -7.55418600 -0.87555500 3.61275300

C -7.38836500 1.89564000 3.18246200

C -6.19882900 1.17578800 3.50042100

C -4.92625700 1.77045100 3.23764700

C -3.73494700 1.04063500 3.52892600

C -3.57788800 3.25266500 1.80854100

C -2.38616900 2.78718700 2.43852400

C -1.11438500 3.17980000 1.91689500

C 0.07912200 2.69101900 2.53070200

C 0.23337300 3.74480000 -0.06126300

C 1.42674000 3.65984800 0.71539600

C 2.69730500 3.72269900 0.06520500

C 3.89298600 3.60499400 0.84080000

C 4.03743000 3.18895200 -1.92890500

C 5.23418800 3.50538200 -1.21992500

C 6.49982200 3.22877000 -1.81973100

C 7.69696300 3.51728300 -1.09747300

C 7.83074900 1.77319100 -3.29381400

C 9.02979100 2.40053400 -2.83982900

C 10.29478000 1.87143500 -3.23712200

C 11.49274400 2.48037000 -2.75657800

C 11.62713900 -0.11236600 -3.82991600

C 12.82587800 0.65946500 -3.74070800

C 14.09248900 0.00587300 -3.83358600

C 15.28721400 0.76795000 -3.70351300

C 15.43029300 -2.01018700 -3.36871700

C 16.63513400 -1.30082800 -3.67488200

C 17.91073100 -1.92835600 -3.48412500

C 19.06096700 -1.17526900 -3.79568500

C -20.33400800 -2.64432000 -2.73192500

C -19.09297500 -3.07462900 -2.14103700

C -17.89322400 -2.56842600 -2.71605300

C -17.75442800 -3.71390400 -0.17737900

C -16.55288900 -3.60715800 -0.94573100

C -15.28022500 -3.73595900 -0.30530400

C -14.08958700 -3.61427500 -1.07724200

C -13.93143200 -3.33668000 1.71082700

C -12.74111100 -3.63383800 0.98070900

C -11.46694800 -3.41525800 1.59156000

C -10.27944900 -3.69230200 0.85443700

C -10.10687900 -2.05717700 3.12883100

C -8.91907800 -2.67952400 2.63840800

C -7.64038900 -2.18548000 3.04778700

C -6.45602500 -2.79432700 2.54102800

C -6.28121700 -0.23636800 3.69076300

C -5.09037900 -1.01709200 3.55812800

C -3.81696500 -0.37991100 3.66140700

C -2.62992400 -1.15180600 3.49275500

C -2.46577000 1.64442000 3.28826800

C -1.27304100 0.89930600 3.54288900

C -0.00182800 1.51184800 3.33317800

C 1.18464700 0.75677100 3.55899500

C 1.34798300 3.09799400 2.02608700

C 2.53989500 2.57581700 2.61704400

C 3.81429300 3.00022900 2.12869100

C 5.00224000 2.46282900 2.70900200

C 5.16202800 3.69278300 0.19366000

C 6.35451600 3.54561600 0.96407100

C 7.62453900 3.65417500 0.32000300

C 8.81682000 3.48550900 1.08609300

C 8.96308800 3.26642500 -1.70764600

C 10.15895300 3.53067100 -0.97604100

C 11.42524300 3.30288000 -1.59392900

C 12.62165300 3.54276800 -0.85175800

C 12.76006400 1.96576800 -3.17183700

C 13.95730600 2.55723400 -2.66947800

C 15.22257500 2.05230200 -3.09415500

C 16.42157600 2.62324300 -2.56400000

C 16.55917500 0.11445600 -3.80920300

C 17.74945900 0.87799300 -3.67037300

C 19.00951000 0.22506300 -3.83434900

C 20.19127600 1.03645300 -3.70342400

C -16.62234500 -3.00432900 -2.22613800

C -15.42239800 -2.50061000 -2.81906600

C -14.15818800 -2.96552100 -2.34823200

C -12.96029600 -2.43886800 -2.91627300

C -12.81963600 -3.75132500 -0.43685200

C -11.62494900 -3.60148700 -1.20526300

C -10.35779400 -3.76397200 -0.56977000

C -9.16457600 -3.58791900 -1.33020700

C -9.00610700 -3.49209000 1.47084900

C -7.81662600 -3.75028000 0.72530300

C -6.54400900 -3.57766000 1.34861500

C -5.35867600 -3.81464700 0.59425700

C -5.17634000 -2.30562100 2.95337400

C -3.98855100 -2.89861500 2.42529800

C -2.71696700 -2.41427900 2.83688600

C -1.52723900 -2.98164400 2.27148100

C -1.35517100 -0.51999500 3.61215300

C -0.16721100 -1.28718800 3.41510300

C 1.09944700 -0.66837400 3.56701500

C 2.28662500 -1.43670200 3.34571100

C 2.45648600 1.37350500 3.37143400

C 3.64526500 0.60749300 3.58396500

C 4.91870100 1.23659300 3.43484200

C 6.10754400 0.47387700 3.63552800

C 6.27518100 2.90042800 2.23387800

C 7.46498700 2.34779800 2.79850400

C 8.73788000 2.80253400 2.33612400

C 9.92841700 2.23866400 2.88527300

C 10.08637800 3.61871400 0.44670800

C 11.27827300 3.42782900 1.20644900

C 12.54869100 3.58275800 0.57081300

C 13.74241200 3.37117300 1.32454100

C 13.88972300 3.34046200 -1.47715400

C 15.08296600 3.55108600 -0.72769700

C 16.35365600 3.35949300 -1.35493100

C 17.55743000 3.55316700 -0.60739600

C 17.69304700 2.14517600 -3.01109200

C 18.89216800 2.71591400 -2.49830800

C 20.13308600 2.23297300 -3.04741200

C -11.69280500 -2.91374800 -2.45302900

C -10.49485700 -2.37022700 -3.00351900

C -9.23042400 -2.85836800 -2.55328000

C -8.02876100 -2.29462000 -3.08194300

C -7.89592400 -3.77401600 -0.69918700

C -6.70411100 -3.57809600 -1.45260300

C -5.44014300 -3.78727200 -0.82547100

C -4.24466500 -3.56500500 -1.57070600

C -4.07504300 -3.66296900 1.21571100

C -2.90111900 -3.90074100 0.46218800

C -1.61321600 -3.72914200 1.06703400

C -0.42049300 -4.02577400 0.34693900

C -0.25171900 -2.52806200 2.70756600

C 0.92825100 -3.11392900 2.16127100

C 2.20141300 -2.65639800 2.62938400

C 3.41430900 -3.20269400 2.07288900

C 3.56193600 -0.81131400 3.55550100

C 4.75492300 -1.56361500 3.35772500

C 6.02639600 -0.94857500 3.58101200

C 7.22344800 -1.69430800 3.35097100

C 7.38255500 1.10610500 3.49449500

C 8.57394600 0.33950800 3.67387500

C 9.84742800 0.97601500 3.54664200

C 11.03876800 0.20503700 3.69847700

C 11.19987600 2.70737700 2.43514300

C 12.39182100 2.12651600 2.96596400

C 13.66465200 2.61090600 2.53120700

C 14.85375100 2.01425200 3.03901000

C 15.00950000 3.54292800 0.69496600

C 16.20487800 3.29395700 1.43706000

C 17.48257900 3.49704600 0.82268700

C 18.66978600 3.28260900 1.57508500

C 18.82274200 3.42278500 -1.25093500

C 19.98090500 3.66935200 -0.44186000

C -6.76625900 -2.80238100 -2.65075100

C -5.56555200 -2.21587900 -3.14827000

C -4.30037500 -2.73796800 -2.72550000

C -2.98325700 -3.83364300 -0.96964100

C -1.79840600 -3.60165400 -1.70320200

C -0.50278500 -4.23041900 -1.18657200

C 0.75383400 -3.61170400 -1.81113900

C 0.81699500 -3.88871100 0.96422700

C 2.06703800 -4.38750400 0.22817700

C 3.37000900 -3.88957800 0.85692800

C 4.55245700 -3.98642800 0.09009300

C 4.68220700 -2.75815800 2.58581800

C 5.88187300 -3.27670600 2.02686500

C 7.15250900 -2.86291600 2.54140300

C 8.34647100 -3.35313900 1.93485700

C 8.49543700 -1.08138400 3.57912200

C 9.68964000 -1.81787000 3.31370500

C 10.96076700 -1.21251600 3.55616600

C 12.15437800 -1.93851600 3.25798300

C 12.31194600 0.84642100 3.58668400

C 13.50540400 0.07096000 3.71307000

C 14.77426900 0.71323000 3.61809200

C 15.96759900 -0.06788000 3.70133600

C 16.12695300 2.50283100 2.60998100

C 17.32719800 1.90102100 3.10671800

C 18.59264300 2.43670800 2.73823700

C 19.74603000 1.79888200 3.29723500

C 19.90781100 3.59305800 0.92363100

C -1.83274000 -2.68588500 -2.75757500

C 0.65386000 -2.61205400 -2.82964900

C 1.98868800 -3.88375900 -1.23515900

C 3.18582300 -3.46445300 -1.88358200

C 4.47061100 -3.76781600 -1.32636900

C 5.64826100 -3.39913500 -2.02001500

C 5.81712500 -3.85790300 0.73076000

C 7.00973900 -3.94320500 -0.04566300

C 8.27559800 -3.87563000 0.60833500

C 9.46647900 -3.93426300 -0.17177100

C 9.61599300 -2.95627300 2.45563600

C 10.80911800 -3.41921800 1.82544200

C 12.07891600 -3.04130100 2.35892800

C 13.27348000 -3.48261300 1.71081400

C 13.42734200 -1.34238000 3.52081100

C 14.61703100 -2.05401300 3.19455300

C 15.88922900 -1.46250500 3.46338500

C 17.09061800 -2.16546100 3.12355800

C 17.24732300 0.57370200 3.64110800

C 18.43300900 -0.20091600 3.76132300

C 19.66958800 0.51869300 3.78329100

C 3.11032800 -2.48961000 -2.91390100

C 5.57209200 -2.40714300 -3.05212900

C 6.92891400 -3.68719900 -1.44350900

C 8.11678500 -3.31819600 -2.13783100

C 9.38811400 -3.62736000 -1.56379200

C 10.57942100 -3.24294700 -2.24627800

C 10.73540600 -3.89295700 0.48065500

C 11.92596600 -3.92577500 -0.30276500

C 13.19814300 -3.90841300 0.34956900

C 14.38675800 -3.91771700 -0.43169700

C 14.54043000 -3.12539300 2.25437400

C 15.73309700 -3.53002100 1.58277000

C 17.01266000 -3.20515600 2.14038600

C 18.19804900 -3.64709900 1.49354000

C 18.35536100 -1.61183500 3.46452000

C 19.50716800 -2.38352700 3.11557100

C 8.03924300 -2.31182800 -3.14903400

C 11.84907900 -3.57419500 -1.68210700

C 13.04296500 -3.17164600 -2.35397500

C 14.31173700 -3.52284700 -1.80385700

C 15.50223200 -3.09319700 -2.45402800

C 15.65622900 -3.90872000 0.21926500

C 16.85844900 -3.90655700 -0.56005500

C 18.12100800 -3.98689100 0.08889400

C 19.26931800 -4.02019100 -0.75705200

C 19.43200800 -3.36227700 2.15498500

C 16.78394600 -3.46606200 -1.92205400

C 17.97203900 -3.08512500 -2.59727000

C 19.19649600 -3.57672600 -2.05723700

H 20.95342900 3.78247700 -0.91016400

H 20.82403600 3.64822100 1.50330200

H 20.72067700 2.26803900 3.20783400

H 20.58551200 0.01010600 4.06790800

H 20.48264200 -2.10365700 3.50046300

H 20.34996300 -3.82865500 1.81085700

H 20.24372900 -4.25521500 -0.34056800

H 20.11640900 -3.47833100 -2.62534400

H 21.15096100 0.62627200 -4.00779000

H 21.05460200 2.76813000 -2.84051200

H 20.04113100 -1.63994800 -3.75097600

H -20.39435700 4.11842100 -0.76659500

H -20.27976000 3.08922200 -2.95017400

H -20.22208700 1.13066900 -3.85438200

H -21.34741300 -1.14251000 -3.85294600

H -21.25764400 -3.14787800 -2.46423100

H -21.15197800 -3.96305800 -0.44150400

H -21.01280400 -3.56688100 1.94276800

H -20.89858600 -1.99701500 3.47730000

H -20.75056200 0.34213300 4.07897100

H -20.63864200 2.38041100 3.28062000

H -20.49946700 3.91576200 1.41785900

C -0.64368800 -5.73412900 -1.65598300

C 0.48894000 -6.77921200 -1.44867400

C 1.04309200 -6.92543300 -0.01214200

C 2.18907100 -5.95581800 0.39346600

F -0.85398700 -5.72511500 -3.00526200

F -1.74414700 -6.28319700 -1.07052100

F 1.51515900 -6.51679000 -2.29805800

F -0.03932700 -7.97660800 -1.80208100

F 1.55509200 -8.17596000 0.09810400

F 0.02029600 -6.82181300 0.87470100

F 3.28281100 -6.39360900 -0.28966900

F 2.39828700 -6.21340800 1.71821000

**(6,5) SWNT-[(CF_2_)_3_CF_3_]_2_ (1,2-L_++_: L_87_)**

C -20.54629000 -1.79543300 -3.71608200

C -19.36062800 -1.01082300 -3.94183300

C -19.40494300 0.38460200 -4.06727100

C -18.25028700 1.16311300 -3.84849600

C -18.10335500 -1.64663100 -3.70556800

C -16.90941600 -0.91043100 -3.93237400

C -15.64016700 -1.55383500 -3.75429700

C -14.44138300 -0.81840300 -3.97105700

C -19.52696900 2.97736300 -2.62895800

C -18.30517100 2.41791400 -3.10617000

C -17.11525100 2.87006300 -2.48046500

C -16.97804200 0.51091100 -3.96371900

C -15.76892800 1.24474300 -3.74220000

C -14.50450500 0.60917700 -3.93704000

C -13.30648400 1.33803400 -3.68623400

C -13.17827000 -1.46401700 -3.80586400

C -11.97430100 -0.71341100 -3.97986700

C -10.71061000 -1.36488600 -3.83748400

C -9.50900500 -0.60816700 -3.97559300

C -19.76355900 3.24552700 1.57683100

C -19.59753200 3.57133300 -1.39012400

C -18.44949600 3.62977000 -0.54493300

C -17.18764400 3.46876600 -1.17996200

C -15.98544900 3.55656300 -0.40539900

C -15.83524200 2.42902500 -2.96299700

C -14.64247700 2.92763700 -2.36811800

C -13.37565200 2.50528800 -2.86970700

C -12.17941400 2.98017900 -2.24943700

C -12.03752200 0.70778800 -3.89305300

C -10.83987000 1.43237800 -3.61885700

C -9.57327500 0.81045900 -3.84120100

C -8.37530300 1.52765300 -3.54012900

C -8.24271300 -1.26342100 -3.84875600

C -7.04132900 -0.50094300 -3.95551800

C -5.77700800 -1.15804500 -3.83905000

C -4.57619200 -0.38953600 -3.91065400

C -3.25158500 -2.36796100 -3.27028800

C -0.78681800 -2.27431500 -3.29049100

C -19.84197000 2.38170200 2.64163000

C -18.69238800 1.65186500 3.07723400

C -18.52820700 3.45152700 0.88926600

C -17.34464700 3.08168300 1.58279400

C -16.06401800 3.33735500 0.99247800

C -14.87283600 3.00843200 1.70677900

C -14.71587700 3.48439100 -1.05281000

C -13.52790500 3.56287300 -0.27498800

C -12.25504100 3.49687300 -0.92398400

C -11.06431100 3.55585300 -0.14091100

C -10.91074600 2.57593700 -2.76604500

C -9.71887300 3.03431900 -2.13301400

C -8.44803300 2.64524600 -2.65825200

C -7.25603100 3.09262400 -2.01376500

C -7.10716200 0.91469300 -3.77985100

C -5.91329700 1.62609600 -3.45917300

C -4.64363900 1.01918900 -3.70810900

C -3.45011300 1.72675900 -3.37425200

C -3.31143700 -1.04531700 -3.79639500

C -2.11663600 -0.27882300 -3.85711000

C -0.85238500 -0.94353000 -3.76263400

C 0.34422300 -0.16210500 -3.84469200

C 1.67674200 -2.16611200 -3.37006400

C 4.13934300 -2.07557700 -3.51337900

C -20.01242700 -0.42320600 3.64433500

C -18.77396300 0.28503000 3.53516300

C -17.59082300 -0.50206000 3.50480400

C -17.42606200 2.15900900 2.67620300

C -16.22665000 1.49546400 3.09394800

C -14.95278400 2.04899900 2.76122100

C -13.76558800 1.37487500 3.16543700

C -13.60556800 3.29799400 1.12653400

C -12.41266200 2.93013300 1.82216200

C -11.14178400 3.24291100 1.25069200

C -9.95192500 2.85306000 1.93149400

C -9.79540600 3.51312200 -0.79099300

C -8.60331000 3.54628600 -0.00628400

C -7.33270800 3.53114900 -0.65775600

C -6.14173800 3.54277800 0.12666500

C -5.98643800 2.72274400 -2.54983500

C -4.79391600 3.15352400 -1.89190700

C -3.52244700 2.80248300 -2.43985500

C -2.32994500 3.21298200 -1.77199100

C -2.18331000 1.12591200 -3.63623000

C -0.98894800 1.82638800 -3.29048000

C 0.27763500 1.23250800 -3.58251500

C 1.47578300 1.92161200 -3.22958700

C 1.60615900 -0.81705100 -3.81874300

C 2.80269600 -0.04411800 -3.88486300

C 4.06808800 -0.70548700 -3.89458500

C 5.26698900 0.07096000 -3.91456600

C 6.60181100 -1.97424400 -3.60494800

C 9.06803700 -1.86694300 -3.67347100

C -20.26538500 -3.81451300 1.16862800

C -20.09351400 -1.75166800 3.31295700

C -18.94316900 -2.45494100 2.83174500

C -17.67552300 -1.88304100 3.13154900

C -16.47790600 -2.54360100 2.70835800

C -16.30897000 0.13754400 3.48907500

C -15.11786900 -0.65173800 3.49627200

C -13.84726600 -0.00769200 3.51602600

C -12.65583800 -0.79624400 3.47727000

C -12.49150100 1.93444900 2.83785600

C -11.29956200 1.24287500 3.21571300

C -10.02956000 1.81602000 2.91015700

C -8.83675700 1.10924300 3.25402400

C -8.68079700 3.18529000 1.37032600

C -7.48801000 2.77304800 2.03732200

C -6.21896700 3.13113700 1.49077700

C -5.02521700 2.69177300 2.13864200

C -4.87016000 3.54936000 -0.52498200

C -3.67608800 3.53569200 0.25962500

C -2.40477700 3.56450300 -0.39152600

C -1.20931300 3.51332700 0.39029000

C -1.05881300 2.87189800 -2.33022200

C 0.14001200 3.25566100 -1.65043600

C 1.40827900 2.93324700 -2.22563600

C 2.60601600 3.28229200 -1.53325800

C 2.73824500 1.34053100 -3.55297000

C 3.93962800 2.00729000 -3.16506200

C 5.20289400 1.43960800 -3.51496800

C 6.40281100 2.08712500 -3.09590200

C 6.53169400 -0.58938500 -3.94437900

C 7.73152200 0.18636200 -3.92310600

C 8.99863500 -0.47270500 -3.97242000

C 10.19707000 0.30034700 -3.92033300

C 10.33672600 -2.45094700 -3.37412400

C 11.53209500 -1.75875700 -3.73111400

C 12.80244100 -2.35388300 -3.44675600

C 13.99789900 -1.65541400 -3.78225400

C -20.34144700 -4.04724300 -0.17888700

C -19.18397600 -3.89764300 -1.01278200

C -19.02509900 -3.43323800 1.77758400

C -17.84023900 -3.73850700 1.05377200

C -16.56071900 -3.46844400 1.63820400

C -15.36766700 -3.80740300 0.92864900

C -15.20218200 -2.01176700 3.07421600

C -14.01592600 -2.66785400 2.63888300

C -12.74036400 -2.13918700 3.01000200

C -11.55051600 -2.78233200 2.54931900

C -11.38051000 -0.15090300 3.51465700

C -10.19162700 -0.93663500 3.44788800

C -8.91707300 -0.29273600 3.49906200

C -7.72528400 -1.07678900 3.40078100

C -7.56513500 1.69695100 2.97161100

C -6.37341400 0.97921000 3.28602100

C -5.10322400 1.57858600 3.02503100

C -3.90940400 0.84671400 3.30559300

C -3.75362000 3.07304600 1.60679400

C -2.56046700 2.60807700 2.23573600

C -1.28816400 3.00185300 1.71665400

C -0.09398700 2.50441700 2.32747100

C 0.06560000 3.55733800 -0.26088600

C 1.26091400 3.47426700 0.51845800

C 2.53258700 3.53307200 -0.13068600

C 3.72612400 3.41299300 0.64551000

C 3.87348100 2.97341900 -2.11907100

C 5.07147400 3.29394800 -1.41205600

C 6.33717300 3.00926500 -2.00873200

C 7.53371400 3.30121800 -1.28827300

C 7.66715000 1.53530800 -3.46650600

C 8.86768500 2.16611900 -3.01822500

C 10.13251300 1.63274700 -3.41069000

C 11.33076100 2.24357500 -2.93371100

C 11.46339400 -0.35780800 -3.98568600

C 12.66364200 0.41343100 -3.90142700

C 13.92921600 -0.24250500 -3.98849100

C 15.12497600 0.51881300 -3.86218200

C 15.26414400 -2.25741400 -3.50874800

C 16.47042000 -1.55152100 -3.81804400

C 17.74482000 -2.17945200 -3.62155800

C 18.89652300 -1.42984500 -3.93650800

C -20.49270700 -2.90961400 -2.92836400

C -19.25279100 -3.33426200 -2.33110300

C -18.05201300 -2.83103700 -2.90690100

C -17.91811500 -3.95950600 -0.36045900

C -16.71528100 -3.85766400 -1.12724900

C -15.44406000 -3.98208900 -0.48351600

C -14.25205000 -3.86594700 -1.25425000

C -14.09900100 -3.56662100 1.53155800

C -12.90794800 -3.87050000 0.80609500

C -11.63502900 -3.64566500 1.41656000

C -10.44673400 -3.93308000 0.68421900

C -10.27648700 -2.26813900 2.93707000

C -9.08856400 -2.89530400 2.45407200

C -7.81066600 -2.39156500 2.85209200

C -6.62462800 -3.00817700 2.35230900

C -6.45322900 -0.43427300 3.47242500

C -5.26294300 -1.21218400 3.33293200

C -3.98949300 -0.57147500 3.42311700

C -2.80152900 -1.33896700 3.23906100

C -2.64012700 1.45558000 3.07296200

C -1.44973100 0.70762600 3.31515100

C -0.17616600 1.32461800 3.11546300

C 1.01043300 0.56689000 3.34861000

C 1.18125000 2.91447200 1.82791400

C 2.36909400 2.39286100 2.42298800

C 3.64475300 2.81137900 1.93522000

C 4.83221200 2.27111400 2.51725100

C 4.99803100 3.49243600 -0.00121200

C 6.18894000 3.34747100 0.77111000

C 7.45973500 3.44849100 0.12799600

C 8.65095600 3.28045200 0.89662800

C 8.80103400 3.04197300 -1.89466800

C 9.99628500 3.31052200 -1.16381900

C 11.26296400 3.07520700 -1.77778800

C 12.45892700 3.31799800 -1.03511400

C 12.59859100 1.72382800 -3.34304800

C 13.79590900 2.31751800 -2.84367000

C 15.06112000 1.80740000 -3.26229500

C 16.26036900 2.38031800 -2.73410900

C 16.39636900 -0.13709100 -3.96187600

C 17.58742600 0.62582900 -3.82673300

C 18.84688400 -0.02984400 -3.98452200

C 20.02963000 0.78091400 -3.85726900

C -16.78207500 -3.26292900 -2.41158300

C -15.58103000 -2.76156500 -3.00454300

C -14.31767900 -3.22368200 -2.52876000

C -13.11853200 -2.69884400 -3.09582700

C -12.98391700 -3.99988700 -0.61074100

C -11.78794600 -3.85595900 -1.37817500

C -10.52238200 -4.01785500 -0.73972300

C -9.32782400 -3.85010000 -1.49928100

C -9.17501300 -3.72719900 1.29929500

C -7.98591900 -3.99927400 0.55777600

C -6.71318200 -3.81967300 1.17798500

C -5.53028000 -4.08195400 0.43041300

C -5.34909400 -2.50652000 2.74564900

C -4.16055300 -3.10416200 2.21240900

C -2.88652400 -2.60334800 2.58960900

C -1.70308200 -3.19658900 2.03966400

C -1.53194800 -0.70969500 3.36267400

C -0.34376300 -1.48255600 3.16089100

C 0.92806100 -0.85397200 3.35319200

C 2.11481900 -1.61489100 3.17502700

C 2.28427600 1.18677700 3.18018100

C 3.47004400 0.42426800 3.40066600

C 4.74644500 1.04827800 3.24612300

C 5.93309400 0.28346900 3.45271700

C 6.10684800 2.70591100 2.04345400

C 7.29354800 2.15236000 2.61040700

C 8.56872300 2.60245500 2.14835900

C 9.75683900 2.03855100 2.70224200

C 9.92182700 3.40802500 0.25853900

C 11.11149600 3.21819100 1.02104700

C 12.38362600 3.36671600 0.38672300

C 13.57537000 3.15712800 1.14398100

C 13.72770000 3.10943400 -1.65697100

C 14.91981900 3.32253600 -0.90669300

C 16.19153700 3.12457400 -1.53024200

C 17.39418500 3.32093900 -0.78167800

C 17.53176900 1.89748300 -3.17595700

C 18.73091500 2.46962000 -2.66471700

C 19.97210000 1.98155300 -3.20893500

C -11.85229700 -3.17247300 -2.62814100

C -10.65205400 -2.62976300 -3.17557000

C -9.38946500 -3.11949100 -2.72267100

C -8.18501400 -2.55281700 -3.24195300

C -8.06164000 -4.03939000 -0.86548800

C -6.86554500 -3.85461500 -1.61826400

C -5.60566900 -4.07942300 -0.99213600

C -4.40178300 -3.86043100 -1.72776400

C -4.25068400 -3.91307100 1.04531800

C -3.06412400 -4.21936100 0.31484900

C -1.80449400 -4.06332300 0.93625400

C -0.58105100 -4.71422100 0.31228300

C -0.42366900 -2.72570800 2.49188300

C 0.77497000 -3.31027300 1.96692900

C 2.04054900 -2.84218400 2.45477300

C 3.23889100 -3.41138600 1.94745400

C 3.38685400 -0.99806900 3.38558200

C 4.57928500 -1.75882900 3.19566800

C 5.85131600 -1.14116300 3.40887200

C 7.04423700 -1.89105300 3.18605300

C 7.20797300 0.91199000 3.30953500

C 8.39767600 0.14363500 3.49407600

C 9.67256900 0.77812900 3.36829600

C 10.86159500 0.00689800 3.52806100

C 11.02958000 2.50310100 2.25283900

C 12.21945400 1.92234300 2.78856300

C 13.49401000 2.40302800 2.35444400

C 14.68079500 1.80747900 2.86796300

C 14.84378100 3.32295700 0.51583400

C 16.03735100 3.07601300 1.26167300

C 17.31657200 3.27356000 0.64863000

C 18.50185900 3.06159900 1.40453800

C 18.66044800 3.18420700 -1.42195100

C 19.81765700 3.43360900 -0.61214900

C -6.92347800 -3.06454600 -2.80861500

C -5.71997800 -2.46961600 -3.28474100

C -4.45663800 -2.99481200 -2.85566600

C -3.13944200 -4.17125200 -1.13433600

C -1.95428300 -3.90738800 -1.85375500

C -0.65434000 -4.57658200 -1.44409800

C 0.57672900 -3.85032000 -1.96015000

C 0.72654400 -4.13185000 0.82021300

C 1.90912300 -4.29355900 0.06693600

C 3.17493800 -4.09263200 0.69935200

C 4.37617600 -4.20924500 -0.06304800

C 4.50778500 -2.97011500 2.44862700

C 5.70506500 -3.49879600 1.88556400

C 6.97208600 -3.07261000 2.38932300

C 8.16947600 -3.56664300 1.78811900

C 8.31721900 -1.27715400 3.40895500

C 9.51144500 -2.01784200 3.15525000

C 10.78184600 -1.41215700 3.39582600

C 11.97588200 -2.14162700 3.10650800

C 12.13625000 0.64584200 3.41559700

C 13.32840200 -0.13017100 3.55055800

C 14.59815700 0.50988900 3.45471200

C 15.79044700 -0.27203900 3.54594600

C 15.95577900 2.29179200 2.43882200

C 17.15404100 1.69150200 2.94198400

C 18.42099200 2.22315700 2.57284700

C 19.57231200 1.58749100 3.13859100

C 19.74182400 3.36575600 0.75355100

C -1.99225600 -2.91877200 -2.86019000

C 0.48638400 -2.82308700 -2.91659700

C 1.83391900 -4.11593900 -1.37205600

C 3.02427500 -3.71523000 -2.04872100

C 4.30124500 -3.99187000 -1.46919200

C 5.48726700 -3.63117200 -2.16971900

C 5.63857000 -4.09683100 0.58871000

C 6.83264500 -4.17839400 -0.18543800

C 8.09987100 -4.10158500 0.46811300

C 9.29330000 -4.16417600 -0.31046000

C 9.43818600 -3.16519800 2.30788100

C 10.63194800 -3.63402100 1.68485900

C 11.90125600 -3.25167300 2.21702700

C 13.09701900 -3.69913400 1.57488100

C 13.24900800 -1.54496100 3.36826800

C 14.43853300 -2.26018300 3.05038300

C 15.71093500 -1.66810900 3.31763500

C 16.91237400 -2.37483000 2.98552200

C 17.07117600 0.36769500 3.48478600

C 18.25555100 -0.40745600 3.61298000

C 19.49305800 0.31070100 3.63297100

C 2.94428300 -2.73454200 -3.07710700

C 5.40864700 -2.64710100 -3.20446500

C 6.75777100 -3.91917600 -1.58653900

C 7.94915300 -3.54983000 -2.27918000

C 9.21878800 -3.86119500 -1.70397700

C 10.41045200 -3.47998600 -2.38618800

C 10.56025300 -4.11806400 0.34312800

C 11.75290100 -4.15437600 -0.43826300

C 13.02394200 -4.13395000 0.21623100

C 14.21373000 -4.14816500 -0.56287500

C 14.36286400 -3.33865700 2.11798400

C 15.55666400 -3.74851700 1.45128900

C 16.83543600 -3.42100300 2.00915100

C 18.02161400 -3.86804600 1.36740400

C 18.17691900 -1.82030100 3.32539800

C 19.32872100 -2.59531500 2.98376700

C 7.87290400 -2.54866600 -3.29234000

C 11.67900400 -3.80927100 -1.81879000

C 12.87441200 -3.41083900 -2.49119400

C 14.14150000 -3.76039800 -1.93740600

C 15.33358300 -3.33506500 -2.58785900

C 15.48208300 -4.13571600 0.09021200

C 16.68575100 -4.13899900 -0.68703000

C 17.94694200 -4.21633200 -0.03536000

C 19.09683600 -4.25543700 -0.87897200

C 19.25457800 -3.58010700 2.02933200

C 16.61405700 -3.70634300 -2.05171800

C 17.80356400 -3.33059400 -2.72718000

C 19.02671900 -3.81984400 -2.18193800

H -21.50456600 -1.41730000 -4.06337700

H -20.38237600 0.85705900 -4.07702200

H -20.44723400 2.81904100 -3.18280700

H -20.68017000 3.67163400 1.18077800

H -20.57046200 3.86082700 -1.00573100

H -20.81856300 2.14925900 3.05424600

H -20.92626500 0.11765800 3.86982800

H -21.18053500 -3.80040800 1.75247100

H -21.06957000 -2.22537700 3.28172100

H -21.31509400 -4.21133900 -0.62945000

H 19.87601500 -1.89551700 -3.88763500

H -21.41649000 -3.41232700 -2.65957500

H 20.98914400 0.36757900 -4.15787700

H 20.89395800 2.51697600 -3.00425700

H 20.79123200 3.54218200 -1.07934700

H 20.54775100 2.05485600 3.04843900

H 20.65699200 3.42293900 1.33468900

H 20.40763900 -0.19716000 3.92314600

H 20.30366900 -2.31384500 3.36880900

H 20.07030700 -4.48857800 -0.45923600

H 20.17277200 -4.04947100 1.68993800

H 19.94773500 -3.72564800 -2.74895800

C -0.59250800 -6.22395400 0.85497300

C -0.66058500 -5.98216400 -2.21875400

C -1.05356100 -5.98830900 -3.74607100

C -0.69868200 -7.33266700 -4.46665500

C -1.43519300 -7.53049600 -5.82211900

C -0.20019900 -6.47574600 2.36075500

C -0.58411300 -7.90766100 2.86553600

C 0.16294200 -8.33252400 4.16185800

F 0.29785200 -6.98962800 0.17696300

F -1.83514400 -6.76889200 0.73952700

F 1.14338800 -6.34058600 2.50167800

F -0.81383800 -5.60614100 3.19639700

F -0.30936400 -8.84024300 1.92628800

F -1.90768400 -7.92956900 3.14032000

F 1.44793400 -8.59893700 3.90413800

F -0.41315800 -9.43931800 4.64971500

F 0.09265300 -7.36746000 5.08976600

F -1.56038400 -6.83398300 -1.66814100

F 0.57531800 -6.55281000 -2.19269900

F -2.39310700 -5.80271400 -3.86659400

F -0.41667800 -5.01335700 -4.43548700

F -1.01150000 -8.39315600 -3.68876600

F 0.62783800 -7.34797200 -4.72765200

F -2.73433700 -7.78092100 -5.62689100

F -0.89003300 -8.57583100 -6.45856100

F -1.30956600 -6.44324000 -6.59620400

**(6,5) SWNT-[(CF_2_)_3_CF_3_]_2_ (1,2-L_+_: L_27_)**

C -20.53989800 -1.55536600 -3.52043600

C -19.35254500 -0.77445200 -3.74922900

C -19.39331500 0.62102500 -3.87633700

C -18.23675300 1.39683000 -3.65850400

C -18.09665700 -1.41299300 -3.51380600

C -16.90090600 -0.67969400 -3.74266800

C -15.63344700 -1.32595700 -3.56638500

C -14.43265900 -0.59290100 -3.78320700

C -19.50950600 3.21133600 -2.43595800

C -18.28891300 2.65092900 -2.91495100

C -17.09781600 3.09942400 -2.28877800

C -16.96578200 0.74195000 -3.77428600

C -15.75485200 1.47284300 -3.55294800

C -14.49172300 0.83444600 -3.74764700

C -13.29086100 1.55991400 -3.49598000

C -13.17101200 -1.24207600 -3.62120800

C -11.96487600 -0.49477000 -3.79375300

C -10.70349300 -1.15097300 -3.65436100

C -9.49618300 -0.39837400 -3.78816300

C -19.74336900 3.46470700 1.77193000

C -19.57898600 3.80109000 -1.19498200

C -18.43073300 3.85482700 -0.35004500

C -17.16925100 3.69465500 -0.98657100

C -15.96675200 3.78000200 -0.21295000

C -15.81869700 2.65687900 -2.77254300

C -14.62489600 3.15210000 -2.17722400

C -13.35808200 2.72742700 -2.67870300

C -12.16146500 3.20026400 -2.05884500

C -12.02365400 0.92698900 -3.70243500

C -10.82300400 1.64656900 -3.42354200

C -9.55670800 1.02170900 -3.64410200

C -8.35799100 1.73291200 -3.33701800

C -8.23559900 -1.05925600 -3.66751200

C -7.02689200 -0.30022200 -3.75138300

C -5.76542300 -0.96173300 -3.63448700

C -4.56517900 -0.20206900 -3.66793900

C -3.23765600 -2.19983300 -3.06008000

C -0.80063300 -2.15026500 -3.12157200

C -19.82070300 2.60023600 2.83645200

C -18.67079700 1.87048100 3.27114500

C -18.50872500 3.67211900 1.08369500

C -17.32462600 3.30067300 1.77553600

C -16.04466900 3.55783900 1.18472300

C -14.85349100 3.22751000 1.89738800

C -14.69777600 3.70794300 -0.86158700

C -13.50949800 3.78594100 -0.08447000

C -12.23713200 3.72017700 -0.73403000

C -11.04710800 3.78085800 0.04880400

C -10.89265300 2.79122400 -2.57262200

C -9.70013900 3.25090300 -1.94068600

C -8.42968100 2.85678000 -2.46100800

C -7.23821900 3.30783900 -1.81739000

C -7.08997500 1.11359000 -3.56697600

C -5.89471600 1.82282000 -3.24415500

C -4.62881900 1.20925000 -3.47747000

C -3.43429900 1.91324300 -3.14429000

C -3.30229600 -0.86376100 -3.53229700

C -2.10438300 -0.10817500 -3.60214600

C -0.85356100 -0.77690300 -3.54056400

C 0.34074300 -0.00891700 -3.62019500

C 1.68361300 -2.04775600 -3.28789900

C 4.15763000 -1.91297800 -3.42977100

C -19.99043000 -0.20283500 3.84498400

C -18.75189400 0.50427300 3.73097600

C -17.56934100 -0.28399300 3.69781700

C -17.40486000 2.37688200 2.86803700

C -16.20516400 1.71242800 3.28372300

C -14.93213800 2.26576500 2.94961700

C -13.74439000 1.58986900 3.35013700

C -13.58676900 3.51924200 1.31681400

C -12.39414800 3.15156000 2.01130600

C -11.12465000 3.46780600 1.44011200

C -9.93455300 3.07900600 2.12056800

C -9.77742500 3.73789900 -0.60087400

C -8.58691500 3.77766100 0.18384300

C -7.31587300 3.75955900 -0.46622300

C -6.12504400 3.78041200 0.31982000

C -5.96712100 2.93210100 -2.34665600

C -4.77680400 3.36860500 -1.69260800

C -3.50536300 3.00344700 -2.23027900

C -2.31124500 3.42018400 -1.56855800

C -2.17011100 1.30623500 -3.39978800

C -0.97897200 1.99513300 -3.06440400

C 0.28020300 1.38532900 -3.34895300

C 1.48010500 2.07379400 -3.00823700

C 1.60804600 -0.67116600 -3.64889300

C 2.79930600 0.11228000 -3.71146000

C 4.07472200 -0.53310000 -3.76238300

C 5.27124000 0.25393300 -3.76739400

C 6.62296400 -1.78385100 -3.49524500

C 9.08845000 -1.65878300 -3.54591500

C -20.25448900 -3.58636000 1.36430000

C -20.07358400 -1.53127800 3.51402100

C -18.92547000 -2.23497800 3.02831100

C -17.65618900 -1.66476400 3.32489100

C -16.46023100 -2.32629800 2.89783000

C -16.28693700 0.35421300 3.67862700

C -15.09641500 -0.43613400 3.68176600

C -13.82501400 0.20686100 3.69755800

C -12.63336600 -0.58215600 3.65331500

C -12.47132200 2.15133200 3.02342700

C -11.27958100 1.45808700 3.39527400

C -10.01032700 2.03549200 3.09221200

C -8.81722800 1.32909800 3.42982100

C -8.66472100 3.41836900 1.56213100

C -7.47311100 3.00812300 2.22893900

C -6.20313600 3.37360500 1.68583100

C -5.01076800 2.94157300 2.33691900

C -4.85308100 3.78368200 -0.32897000

C -3.66049900 3.77581400 0.45682300

C -2.38598000 3.79482300 -0.19235700

C -1.19248100 3.75422200 0.58823700

C -1.04480200 3.06589100 -2.12039600

C 0.15140700 3.44599700 -1.44889700

C 1.41672500 3.10020500 -2.02052500

C 2.61440200 3.46059000 -1.34139200

C 2.73737000 1.49358100 -3.34715500

C 3.93712500 2.16531500 -2.96992100

C 5.20351400 1.61176500 -3.33727400

C 6.40216100 2.26698800 -2.92465200

C 6.54280400 -0.39498600 -3.81730300

C 7.73870000 0.38696900 -3.78296300

C 9.01155800 -0.26456000 -3.83841200

C 10.20674300 0.51310700 -3.77994300

C 10.35738400 -2.23757700 -3.24071000

C 11.55027800 -1.54081400 -3.59358000

C 12.82148300 -2.13194000 -3.30464100

C 14.01494100 -1.43063700 -3.63862300

C -20.33445800 -3.81360500 0.01595200

C -19.17871500 -3.66421600 -0.82009100

C -19.01197600 -3.21020000 1.97172400

C -17.82942500 -3.51528000 1.24404800

C -16.54775200 -3.24943400 1.82632300

C -15.35737400 -3.58916100 1.11339100

C -15.18272400 -1.79598800 3.25972000

C -13.99770100 -2.45284000 2.81983400

C -12.71931900 -1.92543400 3.18589900

C -11.53126800 -2.56867500 2.72027700

C -11.35855900 0.06292600 3.68640200

C -10.16836600 -0.72232500 3.60858800

C -8.89383100 -0.07719000 3.65938900

C -7.70289200 -0.85646500 3.54448900

C -7.54888200 1.92387600 3.15332900

C -6.35535100 1.20624400 3.45977300

C -5.08796300 1.81873700 3.21563800

C -3.89626800 1.09149200 3.49354800

C -3.73932600 3.32440200 1.80746900

C -2.54547600 2.86522600 2.44291800

C -1.27102400 3.25919100 1.92788700

C -0.08011700 2.77018100 2.54229100

C 0.07953700 3.77874700 -0.06379800

C 1.27565700 3.70154700 0.71309500

C 2.54515600 3.74395700 0.05706200

C 3.73886900 3.63651900 0.82782400

C 3.87569800 3.13996600 -1.92984300

C 5.07582200 3.47432700 -1.23488300

C 6.33889600 3.19002200 -1.83686800

C 7.53646300 3.49449200 -1.12575700

C 7.66909800 1.72904300 -3.30788900

C 8.86933200 2.36474000 -2.86086100

C 10.13702500 1.84197300 -3.26113000

C 11.33417700 2.45603700 -2.78446000

C 11.47610700 -0.14011800 -3.84646500

C 12.67423800 0.63450900 -3.75887600

C 13.94195100 -0.01796800 -3.84596100

C 15.13558300 0.74605200 -3.71865300

C 15.28181500 -2.02909700 -3.36125900

C 16.48671100 -1.32097200 -3.67001700

C 17.76210400 -1.94574200 -3.47042300

C 18.91225700 -1.19426400 -3.78647000

C -20.48827700 -2.66926100 -2.73187900

C -19.24872100 -3.09779800 -2.13724600

C -18.04757100 -2.59721400 -2.71501900

C -17.91160500 -3.73123600 -0.17055800

C -16.71029800 -3.63098400 -0.93970800

C -15.43862500 -3.76062200 -0.29895900

C -14.24849000 -3.64627600 -1.07254200

C -14.08610300 -3.35099500 1.71315200

C -12.89736500 -3.65795700 0.98457700

C -11.62195100 -3.43489700 1.59017700

C -10.43644000 -3.72643100 0.85316800

C -10.25361700 -2.05191200 3.09757500

C -9.06511900 -2.67880000 2.60827500

C -7.78610300 -2.16760400 2.98927600

C -6.59824000 -2.77427300 2.46937200

C -6.43033300 -0.21002900 3.61920800

C -5.23821900 -0.97813400 3.46488100

C -3.97344700 -0.32980100 3.57873500

C -2.78180800 -1.08658300 3.38633600

C -2.62551300 1.71262700 3.27895000

C -1.43496800 0.97537500 3.53714700

C -0.16314200 1.59079400 3.34371300

C 1.02485500 0.83243400 3.57034800

C 1.19659300 3.16639700 2.03250900

C 2.38687300 2.64958500 2.62969200

C 3.66043500 3.05771900 2.13024700

C 4.84912300 2.51806300 2.70829700

C 5.00744000 3.69921000 0.17185200

C 6.20097700 3.56784600 0.94055500

C 7.46756900 3.65910900 0.28964400

C 8.66107400 3.50035100 1.05533000

C 8.80313100 3.23822900 -1.73593500

C 9.99984800 3.51662300 -1.01054500

C 11.26603000 3.28578500 -1.62704000

C 12.46315200 3.53436400 -0.88707400

C 12.60466000 1.94292500 -3.19715700

C 13.80101500 2.53983600 -2.69828700

C 15.06790600 2.03426500 -3.11844700

C 16.26627300 2.61014800 -2.59078600

C 16.40888900 0.09286300 -3.81701700

C 17.59796200 0.85865900 -3.68295100

C 18.85902900 0.20545500 -3.83886100

C 20.03976400 1.01958700 -3.71401300

C -16.77806600 -3.03341400 -2.22295200

C -15.57719800 -2.53476700 -2.81781900

C -14.31485700 -3.00142000 -2.34512700

C -13.11519300 -2.47952400 -2.91448900

C -12.97955900 -3.78647200 -0.43243800

C -11.78708100 -3.64673000 -1.20333200

C -10.52126400 -3.81385700 -0.56831900

C -9.32973700 -3.65467000 -1.33457900

C -9.15864400 -3.52146500 1.46097200

C -7.97533000 -3.80362800 0.71580000

C -6.69419200 -3.60461700 1.31858300

C -5.51060100 -3.87398300 0.55869700

C -5.31558500 -2.26404200 2.84532800

C -4.13165700 -2.83166700 2.28438900

C -2.85749600 -2.32313200 2.68841800

C -1.67039800 -2.86760800 2.12413500

C -1.51479600 -0.45095800 3.55953300

C -0.33156500 -1.20426600 3.36298200

C 0.93980500 -0.58909100 3.55643900

C 2.12683500 -1.35201100 3.34790500

C 2.30218000 1.44717100 3.39489500

C 3.48589900 0.67931800 3.60290600

C 4.76296600 1.29665700 3.43927400

C 5.94891400 0.52455000 3.63065300

C 6.12272800 2.94433600 2.22254400

C 7.30936600 2.39137700 2.78599200

C 8.58253300 2.83433100 2.31352400

C 9.77123900 2.27006300 2.86464900

C 9.92938700 3.62318100 0.41165600

C 11.12038800 3.44007000 1.17236000

C 12.39067700 3.58663000 0.53464900

C 13.58373300 3.38115500 1.29102000

C 13.73203300 3.33026700 -1.51053600

C 14.92460700 3.54695400 -0.76165800

C 16.19653300 3.35300100 -1.38623800

C 17.39947200 3.55172900 -0.63831300

C 17.53892000 2.13095700 -3.03364800

C 18.73683700 2.70611400 -2.52320200

C 19.97916500 2.22115900 -3.06788400

C -11.85156500 -2.95959100 -2.45176100

C -10.65069600 -2.41863700 -3.00050100

C -9.39000900 -2.91983700 -2.55660700

C -8.18553600 -2.35921000 -3.07839800

C -8.06635400 -3.85321600 -0.70744000

C -6.87847000 -3.67774700 -1.47276000

C -5.61543600 -3.92224900 -0.85833500

C -4.43331600 -3.77477000 -1.62793500

C -4.22012100 -3.64122900 1.11486900

C -3.04181200 -3.89992500 0.31698800

C -1.73602100 -3.63795200 0.91281300

C -0.56261100 -3.89066000 0.21010700

C -0.41258000 -2.41177800 2.60007800

C 0.77785900 -2.95273500 2.05112900

C 2.04776800 -2.54895500 2.57743700

C 3.24024300 -3.11620600 2.05387900

C 3.39950400 -0.74638200 3.56324800

C 4.58717200 -1.50622700 3.34207000

C 5.86316600 -0.89901300 3.56452600

C 7.05281700 -1.64979400 3.33001600

C 7.22369800 1.14977800 3.48549700

C 8.41134200 0.37683500 3.65801700

C 9.68703000 1.00964900 3.53081500

C 10.87450200 0.23655400 3.68490800

C 11.04180300 2.73137400 2.40861800

C 12.23188300 2.15068400 2.94208700

C 13.50510500 2.63009100 2.50362500

C 14.69215900 2.03553000 3.01614000

C 14.85068900 3.54756200 0.66110900

C 16.04530200 3.30255900 1.40601600

C 17.32356600 3.50200300 0.79201800

C 18.50978300 3.29100100 1.54678100

C 18.66553200 3.41915000 -1.27964200

C 19.82294700 3.66938200 -0.47034600

C -6.93006400 -2.88716600 -2.65405000

C -5.71170200 -2.29680000 -3.12054200

C -4.47134400 -2.84897500 -2.71594500

C -3.16878900 -4.09839000 -1.06313000

C -1.98262900 -4.15997000 -2.03947300

C -0.57127300 -4.47169400 -1.22160800

C 0.61922600 -3.80114800 -1.92717600

C 0.70094400 -3.66153400 0.82426100

C 1.92965500 -3.94244400 0.13517300

C 3.17409400 -3.79766400 0.79595200

C 4.38758200 -3.95495400 0.05209500

C 4.50843100 -2.69982900 2.56520000

C 5.71027400 -3.23549000 2.00427600

C 6.97647000 -2.82344400 2.51803200

C 8.17641400 -3.32079000 1.92102400

C 8.32719800 -1.04303700 3.55938600

C 9.52136000 -1.78422800 3.30027200

C 10.79247400 -1.18259400 3.54621500

C 11.98634100 -1.91154000 3.25455600

C 12.14897500 0.87471700 3.57028300

C 13.34086500 0.09821200 3.70258400

C 14.61017100 0.73852200 3.60502900

C 15.80239000 -0.04287000 3.69560600

C 15.96599700 2.51933900 2.58398500

C 17.16498500 1.92010400 3.08640100

C 18.43111400 2.45194100 2.71504300

C 19.58331500 1.81715300 3.27973000

C 19.74858300 3.59837900 0.89528500

C -1.97897800 -2.78649400 -2.74708600

C 0.49964900 -2.75109400 -2.84491800

C 1.88560200 -3.93660300 -1.29332200

C 3.06823300 -3.56108200 -1.97939600

C 4.33147300 -3.78755100 -1.35862800

C 5.52043900 -3.43622700 -2.06039500

C 5.64786700 -3.84198700 0.71229300

C 6.84665800 -3.94317100 -0.05348100

C 8.11101200 -3.86077100 0.60374100

C 9.30908700 -3.93265600 -0.17060700

C 9.44652500 -2.92649100 2.44663400

C 10.64184700 -3.39849100 1.82755800

C 11.91134700 -3.01928600 2.36239000

C 13.10898300 -3.46777200 1.72223300

C 13.26038500 -1.31631200 3.51874800

C 14.44995700 -2.03127700 3.20105800

C 15.72247300 -1.43918400 3.46881200

C 16.92429400 -2.14586900 3.13782400

C 17.08294500 0.59738500 3.63205700

C 18.26748600 -0.17696600 3.76126000

C 19.50488900 0.54140600 3.77725400

C 2.96899600 -2.60046700 -3.02365500

C 5.43503900 -2.47043000 -3.10808900

C 6.78251000 -3.70729300 -1.46074200

C 7.97427900 -3.34053200 -2.15123400

C 9.24095500 -3.64206200 -1.56649500

C 10.43227200 -3.26248000 -2.24801500

C 10.57353500 -3.88491300 0.48590500

C 11.76860700 -3.92432300 -0.29282300

C 13.03860200 -3.90273700 0.36365200

C 14.22980400 -3.91787900 -0.41386300

C 14.37455400 -3.10913900 2.26741800

C 15.56949000 -3.51877100 1.60199500

C 16.84795100 -3.19212300 2.16157800

C 18.03488000 -3.63889300 1.52100800

C 18.18865200 -1.59080600 3.47765600

C 19.34054200 -2.36657500 3.13850300

C 7.89560800 -2.34811800 -3.17130300

C 11.69863700 -3.58494600 -1.67471200

C 12.89436200 -3.18604400 -2.34631700

C 14.16013700 -3.53180400 -1.78919100

C 15.35221300 -3.10485200 -2.43836900

C 15.49716100 -3.90481700 0.24048700

C 16.70210100 -3.90683900 -0.53557100

C 17.96238200 -3.98448700 0.11735300

C 19.11361400 -4.02019300 -0.72457600

C 19.26707000 -3.35193500 2.18461600

C 16.63230600 -3.47323200 -1.90000300

C 17.82204600 -3.09544900 -2.57402300

C 19.04511500 -3.58255900 -2.02703500

H -21.49776700 -1.17452500 -3.86588500

H -20.36956300 1.09591100 -3.88542400

H -20.43016700 3.05624400 -2.99005400

H -20.66054200 3.89051600 1.37684700

H -20.55141400 4.09074100 -0.80940700

H -20.79701900 2.36726700 3.24941600

H -20.90302200 0.33879300 4.07368800

H -21.16828900 -3.57185000 1.95022700

H -21.05004200 -2.00436600 3.48613200

H -21.30967900 -3.97336200 -0.43278200

H 19.89289900 -1.65737200 -3.73595300

H -21.41321900 -3.16861500 -2.46081900

H 21.00020000 0.60834400 -4.01453700

H 20.89951600 2.75975000 -2.86476400

H 20.79585700 3.78084000 -0.93822400

H 20.55869600 2.28407700 3.18670600

H 20.66417200 3.65621700 1.47570600

H 20.42011100 0.03425500 4.06662100

H 20.31509200 -2.08528000 3.52468700

H 20.08681600 -4.25212400 -0.30357800

H 20.18538000 -3.82206400 1.84661300

H 19.96702600 -3.48582700 -2.59218100

C -2.17816900 -5.22354600 -3.24743800

C -0.39318000 -6.06766500 -1.00016000

C -3.18375700 -5.11369900 -4.46472300

C -2.87089100 -6.16431400 -5.59972800

C -4.08076200 -6.40807700 -6.54991600

C 0.60698300 -6.78839800 -0.00806200

C 0.28214900 -8.32253600 0.16713100

C 1.48514800 -9.13728700 0.72757000

F -2.49816100 -6.44937600 -2.75969000

F -0.97249600 -5.26176000 -3.90202000

F -4.45117700 -5.35148500 -4.04142500

F -3.13750600 -3.91888700 -5.09540200

F -1.85851900 -5.68906100 -6.35938300

F -2.51809700 -7.36963500 -5.10571700

F -4.62081100 -5.24928600 -6.95268100

F -3.64984000 -7.07658900 -7.62825300

F -5.02079100 -7.14105700 -5.94269900

F -1.60252500 -6.51042500 -0.52472300

F -0.08382200 -6.68118400 -2.17093400

F 0.56624400 -6.29217300 1.24896200

F 1.87450200 -6.70351900 -0.48532000

F -0.07486800 -8.91068700 -0.99402600

F -0.73089200 -8.45048700 1.05318400

F 2.02055300 -8.53447800 1.79838800

F 2.43063900 -9.29149700 -0.20611800

F 1.04667700 -10.34945400 1.09350000

**(6,5) SWNT-[(CF_2_)_3_CF_3_]_2_ (1,2-L–: L_–33_)**

C -20.53971000 -1.75610600 -3.70820500

C -19.35243300 -0.97457100 -3.93562600

C -19.39306000 0.42116300 -4.06076500

C -18.23625200 1.19652000 -3.84261800

C -18.09659900 -1.61356200 -3.70144600

C -16.90106200 -0.87997300 -3.92949900

C -15.63349800 -1.52666800 -3.75434900

C -14.43329200 -0.79378500 -3.97183200

C -19.50809400 3.01155600 -2.61999100

C -18.28770800 2.45008400 -3.09778700

C -17.09683400 2.89625000 -2.46940000

C -16.96562400 0.54151700 -3.95922700

C -15.75424900 1.27184200 -3.73647000

C -14.49171800 0.63382400 -3.93348600

C -13.29067900 1.35783600 -3.67883900

C -13.17199600 -1.44334100 -3.81055700

C -11.96574000 -0.69600900 -3.98544300

C -10.70516500 -1.35284100 -3.84977700

C -9.49907600 -0.60022400 -3.98559400

C -19.74837700 3.25872900 1.58680400

C -19.57839800 3.60020700 -1.37848300

C -18.43134900 3.65114900 -0.53190900

C -17.16919100 3.48947600 -1.16649900

C -15.96745100 3.57160600 -0.39077900

C -15.81746300 2.45359600 -2.95268800

C -14.62366500 2.94551800 -2.35460800

C -13.35696200 2.52053200 -2.85588600

C -12.15932000 2.98790100 -2.23180500

C -12.02335100 0.72520700 -3.88956100

C -10.82242100 1.44198800 -3.60756100

C -9.55698300 0.81626300 -3.83286400

C -8.35432000 1.52215500 -3.51886200

C -8.23609900 -1.26348900 -3.87177100

C -7.02870600 -0.50744800 -3.96575500

C -5.76711600 -1.17641900 -3.86352400

C -4.55356700 -0.41927400 -3.90078500

C -3.24219300 -2.44050000 -3.34177300

C -0.75299000 -2.35007800 -3.29966100

C -19.82867600 2.39229700 2.64962000

C -18.68013000 1.66170400 3.08605400

C -18.51215200 3.46645600 0.90163800

C -17.32985100 3.09307700 1.59515900

C -16.04826800 3.34892100 1.00673000

C -14.85894700 3.01807700 1.72190200

C -14.69756200 3.49789300 -1.03721900

C -13.51033700 3.57165500 -0.25792400

C -12.23644300 3.50291000 -0.90508800

C -11.04797200 3.55978700 -0.12002700

C -10.89104600 2.57943800 -2.74619100

C -9.69802200 3.02885000 -2.10733000

C -8.42656800 2.63410200 -2.62785700

C -7.23636100 3.07379200 -1.97721500

C -7.08877100 0.90388500 -3.75845800

C -5.88978900 1.59971300 -3.41549200

C -4.62082500 0.98769000 -3.65993100

C -3.43139200 1.68663200 -3.30490200

C -3.29509400 -1.08804500 -3.79303000

C -2.09371000 -0.31717600 -3.79313400

C -0.83123200 -0.97728300 -3.70213200

C 0.35416000 -0.19782000 -3.74750000

C 1.68024900 -2.21210600 -3.33585200

C 4.15020200 -2.10573800 -3.52108200

C -20.00109300 -0.41307100 3.65284000

C -18.76246500 0.29454900 3.54371100

C -17.57977200 -0.49329500 3.51309400

C -17.41321100 2.16851000 2.68677100

C -16.21473700 1.50392100 3.10555500

C -14.94074900 2.05749300 2.77559800

C -13.75481400 1.38236800 3.18090300

C -13.59088000 3.30666800 1.14326500

C -12.39992300 2.93941500 1.84183400

C -11.12896200 3.25156400 1.27288200

C -9.94154000 2.86456300 1.95892400

C -9.77749500 3.51055300 -0.76681500

C -8.58809400 3.54770400 0.02156200

C -7.31707600 3.52432800 -0.62469200

C -6.12957700 3.54495200 0.16582500

C -5.96532700 2.69306900 -2.50302200

C -4.77615500 3.12829000 -1.84168700

C -3.50669600 2.76957100 -2.37754100

C -2.31787000 3.19269900 -1.70784300

C -2.16424700 1.08122800 -3.55152600

C -0.97304700 1.79192400 -3.20652200

C 0.28693000 1.20940100 -3.49391300

C 1.48012000 1.89867200 -3.14123500

C 1.61479800 -0.84447300 -3.71565200

C 2.81148300 -0.06411600 -3.79266100

C 4.07554200 -0.71318500 -3.84787600

C 5.26965800 0.06698500 -3.86212700

C 6.62286100 -1.97542600 -3.61193700

C 9.08773300 -1.84966100 -3.66658900

C -20.25720100 -3.79689500 1.17116000

C -20.08264700 -1.74135300 3.32059200

C -18.93282200 -2.44426900 2.83780500

C -17.66478600 -1.87363300 3.13795000

C -16.46734400 -2.53338200 2.71199400

C -16.29755800 0.14541200 3.49894600

C -15.10690700 -0.64445500 3.50532400

C -13.83635200 -0.00091100 3.52760700

C -12.64441200 -0.78939400 3.48607300

C -12.48040500 1.94325500 2.85766600

C -11.29010400 1.25219400 3.23746400

C -10.02054700 1.82805400 2.93736500

C -8.82775100 1.12288000 3.28405900

C -8.66943200 3.19774200 1.40194400

C -7.48046300 2.79298300 2.07687400

C -6.21035500 3.15021000 1.53377800

C -5.01784900 2.72221800 2.19285700

C -4.85645000 3.54601400 -0.47938900

C -3.66793300 3.54768100 0.31034200

C -2.39556800 3.56766100 -0.33597100

C -1.20011800 3.53775800 0.45073700

C -1.04650500 2.85525800 -2.25959500

C 0.14587400 3.25495300 -1.58784100

C 1.41207500 2.92500300 -2.15420500

C 2.61190300 3.28740100 -1.46890400

C 2.74430700 1.31962700 -3.46383800

C 3.94201400 1.99546400 -3.09316900

C 5.20383800 1.43133500 -3.44763900

C 6.40503100 2.08594900 -3.03688400

C 6.54378900 -0.58310300 -3.92029900

C 7.73700800 0.19726200 -3.89058400

C 9.01041600 -0.45392100 -3.94884100

C 10.20647900 0.32376200 -3.89027500

C 10.35867900 -2.43151300 -3.36899100

C 11.55037300 -1.73188900 -3.71688200

C 12.82238500 -2.32422500 -3.43224000

C 14.01585600 -1.62026100 -3.76149900

C -20.33509400 -4.02262400 -0.17748000

C -19.17854800 -3.86987200 -1.01184000

C -19.01605800 -3.41905500 1.78040200

C -17.83210300 -3.72139200 1.05408300

C -16.55151300 -3.45403500 1.63833100

C -15.35921700 -3.79033900 0.92644000

C -15.19105600 -2.00330100 3.07861200

C -14.00477900 -2.65704200 2.63907000

C -12.72801500 -2.12969900 3.01103000

C -11.53765900 -2.76850100 2.54516300

C -11.36968300 -0.14402300 3.52864200

C -10.18017300 -0.92785800 3.45514100

C -8.90526300 -0.28268700 3.51500700

C -7.71299100 -1.06107500 3.40852300

C -7.55831000 1.71603700 3.01167400

C -6.36608500 1.00013800 3.32718400

C -5.09579400 1.60806500 3.08117300

C -3.90282200 0.88290700 3.36761100

C -3.74748100 3.10342600 1.66540200

C -2.55419600 2.64697900 2.30311100

C -1.27952100 3.04305300 1.78725500

C -0.08646800 2.55854500 2.40402000

C 0.07086800 3.58075100 -0.19681600

C 1.26586600 3.50566400 0.58108300

C 2.53880600 3.55751900 -0.07059800

C 3.73366800 3.44783000 0.70338400

C 3.87616000 2.97659600 -2.05573000

C 5.07421000 3.30351100 -1.35649700

C 6.33971900 3.01355100 -1.95450300

C 7.53683400 3.31379600 -1.24039300

C 7.66949100 1.54388400 -3.41806700

C 8.86896100 2.17684800 -2.97148400

C 10.13745800 1.65204600 -3.37037200

C 11.33396000 2.26538000 -2.89367700

C 11.47571100 -0.32886500 -3.96143300

C 12.67376800 0.44514400 -3.87091500

C 13.94232400 -0.20658800 -3.96117900

C 15.13559000 0.55707100 -3.83077500

C 15.28296900 -2.21993800 -3.48771200

C 16.48758000 -1.50960100 -3.79161800

C 17.76348400 -2.13489800 -3.59481000

C 18.91314600 -1.38162400 -3.90696900

C -20.48785000 -2.87167600 -2.92212100

C -19.24818500 -3.30108700 -2.32800600

C -18.04720800 -2.79897800 -2.90416900

C -17.91207500 -3.93587300 -0.36096300

C -16.70998900 -3.83262700 -1.12850100

C -15.43823200 -3.96038700 -0.48634800

C -14.24760800 -3.84387700 -1.25828100

C -14.08932400 -3.55108400 1.52838200

C -12.89870100 -3.85197600 0.80024900

C -11.62384800 -3.62728200 1.40845500

C -10.43695800 -3.91063900 0.67266900

C -10.26287500 -2.25393000 2.93234600

C -9.07272400 -2.87330700 2.44046800

C -7.79375300 -2.36933600 2.83843700

C -6.60728000 -2.97077900 2.32424700

C -6.44264800 -0.41528700 3.49233400

C -5.24799900 -1.18522400 3.34436400

C -3.98009500 -0.54133400 3.46281500

C -2.78908800 -1.29907800 3.27109800

C -2.63387900 1.49867100 3.14439200

C -1.43896000 0.75707700 3.39603000

C -0.16824900 1.37370700 3.20149300

C 1.01917900 0.61443200 3.41747900

C 1.18632500 2.95865900 1.89703400

C 2.37967100 2.43552400 2.48959300

C 3.65379300 2.85180100 1.99879500

C 4.84039900 2.30374200 2.57214000

C 5.00264100 3.51760500 0.05285100

C 6.19645300 3.37385100 0.82297300

C 7.46504800 3.47038500 0.17673400

C 8.65603600 3.30009300 0.94288100

C 8.80252500 3.05291900 -1.84767300

C 9.99935100 3.32544700 -1.11902200

C 11.26553100 3.09421900 -1.73433900

C 12.46115900 3.33703400 -0.99159700

C 12.60429400 1.75203200 -3.30566600

C 13.80074800 2.34722700 -2.80408200

C 15.06769900 1.84317500 -3.22510700

C 16.26545600 2.41621200 -2.69345200

C 16.40921900 -0.09514400 -3.93160600

C 17.59819400 0.67035900 -3.79326100

C 18.85945200 0.01851000 -3.95218300

C 20.03987500 0.83190900 -3.82183400

C -16.77763000 -3.23463400 -2.41129500

C -15.57671700 -2.73537100 -3.00565600

C -14.31435600 -3.20092100 -2.53218600

C -13.11541700 -2.67895000 -3.10209700

C -12.97847200 -3.97943400 -0.61641600

C -11.78471500 -3.83816800 -1.38638500

C -10.51865200 -3.99905800 -0.74999700

C -9.32719000 -3.83663200 -1.51501600

C -9.16122700 -3.70044700 1.28320900

C -7.97379900 -3.97089000 0.53876800

C -6.69734700 -3.77860400 1.15028800

C -5.51451900 -4.03484400 0.39393500

C -5.32671700 -2.46685400 2.72071200

C -4.13917100 -3.03942900 2.17562600

C -2.86888100 -2.53745700 2.57265100

C -1.67829800 -3.06832900 1.98189300

C -1.51852400 -0.66582100 3.42278900

C -0.33199900 -1.42073900 3.20919600

C 0.93413700 -0.80825400 3.38878300

C 2.11888600 -1.57302500 3.15484900

C 2.29509900 1.22721600 3.23910700

C 3.47883200 0.45255700 3.43740500

C 4.75312900 1.07039400 3.28490600

C 5.93732000 0.29267400 3.46880000

C 6.11532300 2.73326400 2.09484400

C 7.30067700 2.17041700 2.65520800

C 8.57419900 2.61824000 2.19312200

C 9.76131600 2.04537600 2.74056100

C 9.92622100 3.42429900 0.30297800

C 11.11548500 3.23230000 1.06477700

C 12.38635500 3.38124100 0.43064000

C 13.57788900 3.16785400 1.18759000

C 13.73094500 3.13337100 -1.61370000

C 14.92220400 3.34531700 -0.86186800

C 16.19460100 3.15311100 -1.48520600

C 17.39655700 3.34651800 -0.73409800

C 17.53866600 1.93911100 -3.13720600

C 18.73599800 2.51137600 -2.62235400

C 19.97870000 2.02973200 -3.16858900

C -11.85047300 -3.15676800 -2.63784700

C -10.65148100 -2.61853200 -3.19035200

C -9.39009100 -3.11372200 -2.74187200

C -8.18625900 -2.55754000 -3.27315500

C -8.05976600 -4.02269100 -0.88491500

C -6.87242900 -3.84905800 -1.64817900

C -5.60856500 -4.06162400 -1.02417400

C -4.42116400 -3.88832900 -1.79210000

C -4.22464900 -3.83575100 0.98800900

C -3.05742700 -4.08006400 0.22356800

C -1.75397600 -3.81198800 0.77302400

C -0.56889900 -4.09657400 0.04688800

C -0.41195800 -2.62576000 2.44033100

C 0.76732400 -3.17599100 1.87277200

C 2.03562900 -2.76312100 2.38284700

C 3.23060400 -3.33741000 1.85403100

C 3.39195000 -0.97116600 3.37976500

C 4.57519400 -1.73375200 3.16038300

C 5.85022300 -1.12898000 3.39211500

C 7.04250700 -1.88099700 3.16512200

C 7.21289600 0.91968100 3.33789200

C 8.39873900 0.14516000 3.50998800

C 9.67475500 0.77878600 3.39326900

C 10.86241600 0.00458400 3.54786400

C 11.03349500 2.51132700 2.29397000

C 12.22146600 1.92522900 2.82525400

C 13.49605100 2.40757600 2.39390400

C 14.68189500 1.80929200 2.90533500

C 14.84592300 3.33760100 0.56115900

C 16.03859900 3.08655300 1.30636500

C 17.31818500 3.28858500 0.69573900

C 18.50282800 3.07286400 1.45150200

C 18.66340800 3.21698400 -1.37422400

C 19.81953100 3.46099000 -0.56158100

C -6.92899600 -3.08011400 -2.84962800

C -5.72152400 -2.49973600 -3.34412200

C -4.46366100 -3.04895200 -2.93849300

C -3.16351700 -4.16844900 -1.19755100

C -1.97505700 -4.04228700 -1.96925400

C -0.70143000 -4.66369100 -1.38415100

C 0.61333500 -4.29628900 -2.33453600

C 0.67879200 -3.90736800 0.64366600

C 1.90972000 -4.17467400 -0.08909300

C 3.16729500 -4.02227000 0.60364600

C 4.38460400 -4.20457600 -0.11058100

C 4.49502100 -2.92885600 2.38146500

C 5.70225000 -3.46862600 1.83531400

C 6.96930900 -3.05630500 2.35827200

C 8.17056700 -3.55730000 1.77091400

C 8.31464900 -1.27501900 3.40434900

C 9.51059400 -2.01552300 3.14982100

C 10.78132800 -1.41376600 3.40340500

C 11.97640700 -2.14170500 3.11581500

C 12.13665800 0.64397400 3.44325700

C 13.32875200 -0.13285400 3.57515100

C 14.59839700 0.50858400 3.48599000

C 15.79061800 -0.27259400 3.57644100

C 15.95647300 2.29587500 2.47938100

C 17.15444700 1.69379700 2.98098700

C 18.42138500 2.22794100 2.61567500

C 19.57208600 1.59094600 3.18038600

C 19.74281400 3.38239700 0.80370900

C -1.98870400 -3.03432100 -2.94109500

C 0.49052700 -2.90508700 -2.99400000

C 1.89014800 -4.25171200 -1.48726700

C 3.07964100 -3.82906700 -2.14278300

C 4.33798700 -4.03978700 -1.52172900

C 5.52573500 -3.65510600 -2.20799900

C 5.64625400 -4.08723000 0.55522300

C 6.84932400 -4.18754500 -0.20685800

C 8.11027700 -4.10090800 0.45368900

C 9.31030900 -4.16059500 -0.31897400

C 9.43942800 -3.15858400 2.29886000

C 10.63798000 -3.62935500 1.68341800

C 11.90506400 -3.24864600 2.22176000

C 13.10496900 -3.69270900 1.58245900

C 13.24950500 -1.54634500 3.38546500

C 14.44091300 -2.25975200 3.06929300

C 15.71212300 -1.66807600 3.34304900

C 16.91547100 -2.37237600 3.01170500

C 17.07121200 0.36844900 3.51971200

C 18.25583400 -0.40594500 3.64875800

C 19.49270000 0.31277600 3.67183500

C 2.97881600 -2.79500900 -3.12249500

C 5.43619700 -2.66826400 -3.22812500

C 6.78913300 -3.93455700 -1.61082000

C 7.97683800 -3.55271300 -2.29386600

C 9.24410300 -3.85685700 -1.71100600

C 10.43501300 -3.46686000 -2.38753300

C 10.57392700 -4.11204200 0.34053600

C 11.76934700 -4.14318000 -0.43646000

C 13.03812300 -4.12155000 0.22213200

C 14.23045000 -4.12953300 -0.55382600

C 14.36901600 -3.33422400 2.13158600

C 15.56555800 -3.73914000 1.46648200

C 16.84248000 -3.41349200 2.02985800

C 18.03122500 -3.85528200 1.38892300

C 18.17860800 -1.81840600 3.35770400

C 19.33186000 -2.59128300 3.01687300

C 7.89566200 -2.54439300 -3.30063700

C 11.70072600 -3.79294000 -1.81624500

C 12.89612500 -3.38560200 -2.48246000

C 14.16204600 -3.73424100 -1.92654200

C 15.35383900 -3.30160500 -2.57131200

C 15.49635200 -4.11802200 0.10264100

C 16.70236400 -4.11306600 -0.67152600

C 17.96175700 -4.19262500 -0.01698500

C 19.11416100 -4.22163200 -0.85736400

C 19.26156700 -3.57100100 2.05682900

C 16.63376800 -3.67165700 -2.03344900

C 17.82387300 -3.28898100 -2.70393100

C 19.04677200 -3.77713300 -2.15768600

H -21.49765300 -1.37481500 -4.05295900

H -20.36925700 0.89617700 -4.06868900

H -20.42817800 2.85829500 -3.17556200

H -20.66446400 3.68551400 1.19028900

H -20.55091400 3.89096300 -0.99398900

H -20.80611800 2.15893200 3.05970600

H -20.91468700 0.12800200 3.87885100

H -21.17162300 -3.78514600 1.75618300

H -21.05888100 -2.21466000 3.28910800

H -21.30938300 -4.18397400 -0.62766300

H 19.89397900 -1.84452500 -3.85835400

H -21.41268700 -3.37190100 -2.65232900

H 21.00062200 0.42239500 -4.12372200

H 20.89891000 2.56697000 -2.96127300

H 20.79349600 3.57340900 -1.02702700

H 20.54760500 2.05852800 3.09218700

H 20.65769600 3.43541900 1.38569100

H 20.40734100 -0.19539100 3.96124300

H 20.30530400 -2.31128700 3.40675700

H 20.08722200 -4.45399000 -0.43625400

H 20.18115500 -4.03802600 1.71796800

H 19.96944700 -3.67578500 -2.72076100

C 0.72681100 -5.32670300 -3.57476000

C -0.82818400 -6.26224400 -1.18196600

C 1.64145800 -5.14096400 -4.85070400

C 1.28550000 -6.15638900 -6.00233800

C 2.43124200 -6.32061800 -7.04409400

C -1.74601500 -6.98858300 -0.11941700

C -1.40414100 -8.51975700 0.02886400

C -2.55462100 -9.34119300 0.68132900

F 0.41818100 -6.70702300 -0.82390700

F -1.22947000 -6.85854900 -2.33417000

F -1.61433200 -6.48796300 1.12964000

F -3.04597600 -6.90419900 -0.50045300

F -1.13839700 -9.10204200 -1.15970900

F -0.32244900 -8.64385400 0.83048500

F -2.08307500 -10.55180400 1.00929600

F -3.57054600 -9.49820400 -0.17465500

F -3.00566100 -8.74195100 1.79227100

F -0.52206500 -5.39442900 -4.13590500

F 1.11790300 -6.55328500 -3.14293400

F 1.51796900 -3.92249600 -5.42321000

F 2.94115900 -5.35143300 -4.52045300

F 1.01014000 -7.39075800 -5.53035300

F 0.20547900 -5.69036900 -6.66892200

F 2.90045100 -5.12843300 -7.43810100

F 3.43709400 -7.03953300 -6.53350300

F 1.94750300 -6.96488800 -8.11476200

**(6,5) SWNT-[(CF_2_)_3_CF_3_]_2_ (1,4-L_++_: L_87_)**

C -20.28942100 3.02274900 1.87413500

C -19.10319500 2.53521900 2.52765800

C -19.14440300 1.49390400 3.46556300

C -17.98692400 0.74532400 3.76055000

C -17.84657400 2.90841600 1.96036600

C -16.65204200 2.46057200 2.58639100

C -15.38407400 2.87580200 2.06211500

C -14.18537100 2.42300500 2.68025600

C -19.25292000 -1.44335900 3.87169800

C -18.03472600 -0.70436400 3.91942900

C -16.84081000 -1.43582800 3.69024400

C -16.71691800 1.34205100 3.46414600

C -15.50488800 0.62769200 3.72996200

C -14.24380800 1.25959400 3.50834400

C -13.04159500 0.53154600 3.74580200

C -12.92347500 2.84840900 2.16528300

C -11.71940000 2.36063000 2.76049300

C -10.45874100 2.80604000 2.26157200

C -9.25487800 2.29364600 2.83377500

C -19.47752200 -4.17009900 0.66190900

C -19.31735800 -2.66113600 3.23487600

C -18.16672600 -3.20828000 2.59355700

C -16.90695500 -2.69403400 3.00645200

C -15.70208100 -3.22161200 2.43817100

C -15.56373400 -0.78819500 3.81452900

C -14.36693000 -1.53702700 3.63518900

C -13.10251700 -0.89260200 3.78595600

C -11.90122700 -1.63598200 3.56986800

C -11.77635100 1.16817200 3.54061800

C -10.57416500 0.43028900 3.75262100

C -9.31067100 1.07204400 3.56696700

C -8.10546100 0.32590300 3.74857300

C -7.99253500 2.75848700 2.35146600

C -6.78918300 2.22403400 2.90002700

C -5.52735600 2.70509500 2.42791200

C -4.31359600 2.15144700 2.94529000

C -3.01519900 3.41434300 1.29026600

C -0.52291000 3.76505700 1.56516700

C -19.55629700 -4.11259300 -0.70812200

C -18.40770600 -3.78548200 -1.49348000

C -18.24291400 -3.92188700 1.33631900

C -17.05897900 -4.03464900 0.55955700

C -15.77860200 -3.88199100 1.18602600

C -14.58748900 -4.04106700 0.41725200

C -14.43439000 -2.76968200 2.91269800

C -13.24338100 -3.29101900 2.33530900

C -11.97156600 -2.84274400 2.81376000

C -10.77936800 -3.35115900 2.21978800

C -10.63601400 -0.99636300 3.74252600

C -9.43742600 -1.73191200 3.50029900

C -8.16977900 -1.09963200 3.68965700

C -6.97381900 -1.82944000 3.42381500

C -6.84402800 0.97314000 3.58357700

C -5.63660700 0.21854500 3.72755600

C -4.37414800 0.86697400 3.57846700

C -3.17560600 0.10527700 3.67996900

C -3.06535300 2.64890400 2.48600600

C -1.83543400 2.06018800 2.95904300

C -0.58578700 2.59902100 2.58648700

C 0.59546200 1.96042500 3.00107100

C 1.97389100 3.33988600 1.50606300

C 4.43103900 3.26346400 1.63038600

C -19.73018300 -2.46692000 -3.19154700

C -18.49097700 -2.96583000 -2.67954500

C -17.30915000 -2.31569000 -3.12758800

C -17.14100100 -3.94906500 -0.86843500

C -15.94254700 -3.66531400 -1.60035700

C -14.66862200 -3.90620800 -1.00208000

C -13.48294100 -3.60762900 -1.73070000

C -13.31993800 -3.91960200 1.05495600

C -12.12753300 -4.04016600 0.27747000

C -10.85694500 -3.94068900 0.92072800

C -9.66823300 -4.03845400 0.14041800

C -9.51014700 -2.91737300 2.70913700

C -8.31754900 -3.40982000 2.09970400

C -7.04817200 -2.99371400 2.60175500

C -5.85710100 -3.47406200 1.97915700

C -5.70470700 -1.20352800 3.62592500

C -4.51135400 -1.93174700 3.34329800

C -3.24464500 -1.31312900 3.55611300

C -2.05089200 -2.04082500 3.26170800

C -1.90809700 0.75419500 3.53255500

C -0.71771600 -0.01163000 3.65308100

C 0.53860800 0.63917500 3.54502700

C 1.73805500 -0.12834100 3.66280000

C 1.89115300 2.48345300 2.63152700

C 3.07343600 1.86537000 3.12011000

C 4.35047600 2.39094700 2.74794700

C 5.54635400 1.75554200 3.20976200

C 6.89131500 3.20143500 1.73866800

C 9.35439000 3.13299900 1.84239500

C -19.99217600 1.72971800 -3.25039400

C -19.81324100 -1.20615300 -3.72549000

C -18.66456800 -0.35267100 -3.76311400

C -17.39576700 -0.98735000 -3.65803900

C -16.19951300 -0.20201100 -3.71331900

C -16.02650100 -2.81549200 -2.73084600

C -14.83703500 -2.18624700 -3.21022900

C -13.56616900 -2.71179900 -2.83982800

C -12.37577200 -2.05547600 -3.28160800

C -12.20820500 -3.85723700 -1.13369900

C -11.01927500 -3.53263300 -1.85336500

C -9.74848900 -3.80573500 -1.26579600

C -8.55794900 -3.45368400 -1.97055500

C -8.39623700 -3.96064500 0.78544200

C -7.20757800 -4.03867000 0.00241100

C -5.93631300 -3.98536000 0.64966100

C -4.74594000 -4.04088600 -0.13511900

C -4.58641800 -3.07834000 2.49363400

C -3.39727900 -3.54310400 1.85894800

C -2.12541200 -3.16190400 2.38468600

C -0.93258300 -3.60604700 1.73928300

C -0.78572100 -1.43564500 3.50530000

C 0.40563600 -2.14335100 3.19280000

C 1.67199500 -1.53494400 3.45463400

C 2.86942700 -2.23715100 3.12800400

C 3.00466900 0.52701300 3.62493000

C 4.19816200 -0.24449100 3.70461800

C 5.47237700 0.41052800 3.67304900

C 6.66780600 -0.36727900 3.71907200

C 6.81579700 2.29604800 2.84123000

C 8.01029100 1.64736700 3.27489100

C 9.28162000 2.19753600 2.91648900

C 10.47645700 1.53891600 3.33174700

C 10.62042900 3.41222000 1.24576800

C 11.81507800 3.06782300 1.94323300

C 13.08455200 3.36716900 1.35413000

C 14.27960700 3.00601600 2.03942700

C -20.07308400 2.72149500 -2.30926100

C -18.91881300 3.10111000 -1.54733600

C -18.74991800 1.06239300 -3.50638500

C -17.56749300 1.74245100 -3.10585300

C -16.28560700 1.17919300 -3.40891900

C -15.09482900 1.87767000 -3.03993300

C -14.92276600 -0.84392000 -3.68598800

C -13.73781900 -0.05514500 -3.72644400

C -12.46086500 -0.69847900 -3.70674200

C -11.27198800 0.09358800 -3.71716800

C -11.10126600 -2.59542400 -2.92783800

C -9.91360100 -1.92386600 -3.34304800

C -8.63829600 -2.47544400 -3.00641500

C -7.44919100 -1.79022400 -3.39518700

C -7.28747200 -3.75406200 -1.39440500

C -6.09735400 -3.37672700 -2.08415000

C -4.82571800 -3.70416100 -1.52090300

C -3.63621600 -3.30412200 -2.19478100

C -3.47562200 -4.01189700 0.51325100

C -2.28343000 -4.03631700 -0.27294100

C -1.01020500 -4.02966800 0.37550900

C 0.17992900 -4.02030900 -0.40797600

C 0.33484600 -3.23820800 2.27754600

C 1.53026500 -3.64690100 1.61396900

C 2.79917600 -3.29392500 2.16913800

C 3.99198400 -3.67664600 1.49182300

C 4.13224200 -1.64397800 3.42362700

C 5.33077800 -2.32916600 3.05958600

C 6.59858200 -1.75391300 3.38136100

C 7.79316500 -2.42431000 2.98685900

C 7.93731800 0.28833900 3.69959800

C 9.13540200 -0.49156700 3.71113600

C 10.40482000 0.16430700 3.71366900

C 11.59973500 -0.61469300 3.69374400

C 11.74415700 2.09943200 2.98680500

C 12.94224000 1.42536100 3.37682400

C 14.20910400 1.99881100 3.05177400

C 15.40253900 1.30935200 3.40381200

C 15.54500000 3.31911000 1.45656000

C 16.75137300 2.93551800 2.12491300

C 18.02584300 3.31720400 1.58951600

C 19.17737600 2.90218400 2.28909200

C -20.23441900 3.44502700 0.57642600

C -18.99280000 3.43493600 -0.15312800

C -17.79377400 3.37967900 0.61206200

C -17.65058500 2.76427800 -2.10408300

C -16.45031000 3.14334800 -1.42516100

C -15.17671400 2.86197800 -2.01229200

C -13.98765000 3.23199600 -1.32144700

C -13.82370100 1.32645100 -3.37477900

C -12.63380600 2.00519300 -2.97028200

C -11.35831000 1.46207300 -3.32038200

C -10.17141300 2.12827200 -2.89603600

C -9.99785600 -0.54985600 -3.71943700

C -8.80790300 0.24133700 -3.69713100

C -7.53158100 -0.40171500 -3.72106400

C -6.34449300 0.38738000 -3.66723000

C -6.17694400 -2.35600900 -3.07778900

C -4.98489500 -1.65574300 -3.43459100

C -3.71633300 -2.23923400 -3.14390800

C -2.52600600 -1.52247400 -3.46381900

C -2.36428800 -3.65005400 -1.64295600

C -1.17257500 -3.22315400 -2.30232300

C 0.09835900 -3.58411300 -1.76775500

C 1.28670900 -3.12194400 -2.40846500

C 1.45291500 -4.02080300 0.24046100

C 2.64501800 -3.97735100 -0.54551400

C 3.91524200 -4.00111800 0.10259600

C 5.10466400 -3.92623300 -0.68157700

C 5.26032700 -3.34164900 2.05778200

C 6.45420700 -3.70246100 1.36462800

C 7.72135700 -3.39599600 1.94339100

C 8.91424000 -3.73051800 1.23468700

C 9.06376600 -1.86183500 3.32479400

C 10.25852900 -2.52020000 2.90465100

C 11.52716900 -1.97139300 3.26051800

C 12.72240200 -2.61416100 2.81201900

C 12.87132800 0.04165400 3.71185700

C 14.06620500 -0.73667600 3.66643000

C 15.33375100 -0.08157600 3.69567300

C 16.53083200 -0.86011000 3.61536000

C 16.67536800 1.89023600 3.08828700

C 17.86454500 1.19514000 3.43608600

C 19.12547500 1.81077100 3.16748500

C 20.30619500 1.08206900 3.55164900

C -16.52237300 3.43419100 -0.04024100

C -15.32360800 3.39298600 0.73783200

C -14.05932400 3.48186100 0.08305600

C -12.86245900 3.40852200 0.85590800

C -12.71596500 2.95643600 -1.91272900

C -11.52357200 3.30416900 -1.20812600

C -10.25456700 3.04942500 -1.80891700

C -9.06309800 3.37528800 -1.09434100

C -8.89493700 1.59479200 -3.25838100

C -7.70664600 2.25059200 -2.81569500

C -6.43208200 1.72746000 -3.19018600

C -5.24314600 2.36948900 -2.72086200

C -5.06430400 -0.25475700 -3.70314300

C -3.87821500 0.52795400 -3.61571300

C -2.60570000 -0.11453200 -3.65918400

C -1.41385300 0.66365500 -3.53355000

C -1.25376200 -2.12055100 -3.20697400

C -0.06916200 -1.39831500 -3.51277100

C 1.20158200 -1.99314700 -3.27448600

C 2.38862400 -1.25086600 -3.56142300

C 2.56237100 -3.49592700 -1.88632500

C 3.74703300 -3.01373500 -2.51632600

C 5.02093300 -3.40217300 -2.00533200

C 6.20826000 -2.90385400 -2.62275200

C 6.37674200 -3.97462600 -0.03462800

C 7.56410300 -3.87914500 -0.81666300

C 8.83579300 -3.95098300 -0.17183300

C 10.02530800 -3.83541900 -0.95175400

C 10.18445700 -3.45162600 1.82466100

C 11.37430500 -3.76315500 1.10456400

C 12.64622900 -3.50419400 1.70246000

C 13.83823000 -3.79458900 0.97182900

C 13.99345200 -2.08068600 3.18818100

C 15.18402600 -2.70384200 2.71496200

C 16.45768800 -2.17659500 3.09696700

C 17.65882100 -2.78548600 2.61479500

C 17.80439100 -0.21246100 3.67874200

C 19.00111700 -0.98040800 3.61262600

C 20.24454600 -0.26706800 3.75448100

C -11.59541800 3.51159200 0.20172200

C -10.39951100 3.41500900 0.97144600

C -9.13462200 3.53806100 0.32003800

C -7.93598300 3.41758500 1.08602100

C -7.79208700 3.14206900 -1.70344500

C -6.60476900 3.45034000 -0.98071600

C -5.33292000 3.22936800 -1.59419000

C -4.14314200 3.52479000 -0.86450100

C -3.96050900 1.86053500 -3.09799800

C -2.78475800 2.48572600 -2.60334100

C -1.48410000 1.97904400 -2.97741700

C -0.30904400 2.62546300 -2.55641300

C -0.15107400 0.02637100 -3.64731600

C 1.03152800 0.80266100 -3.51923400

C 2.30568100 0.16768500 -3.67189200

C 3.49682800 0.94015300 -3.56338000

C 3.66128200 -1.85905600 -3.35460200

C 4.84749900 -1.11633600 -3.62893500

C 6.12327400 -1.73170300 -3.43209400

C 7.31237500 -0.98811200 -3.69015800

C 7.48077900 -3.31418100 -2.12560600

C 8.66858400 -2.80223500 -2.72762000

C 9.94280000 -3.23062800 -2.24260800

C 11.13040700 -2.70610700 -2.83003200

C 11.29518400 -3.93438200 -0.31047600

C 12.48524000 -3.79221600 -1.08627900

C 13.75854400 -3.91694600 -0.44919900

C 14.94530800 -3.74943000 -1.21666000

C 15.10653500 -3.55502000 1.57493000

C 16.29985800 -3.80665500 0.82996800

C 17.57943200 -3.60225100 1.43982200

C 18.76419800 -3.88719500 0.70743100

C 18.92647300 -2.29717800 3.04650700

C 20.08175600 -2.98536700 2.54760800

C -6.67548200 3.56791300 0.43888800

C -5.47573100 3.41353100 1.19806500

C -4.21264900 3.59329300 0.55633100

C -2.87630100 3.33012200 -1.46899900

C -1.67967800 3.65686900 -0.72735100

C -0.38401800 3.78076800 -1.52380900

C 0.83932200 3.67798100 -0.60680600

C 0.94561000 2.10262400 -2.93374700

C 2.16990300 2.69907400 -2.45478900

C 3.42315400 2.21857000 -2.91857300

C 4.63082800 2.77952800 -2.39580000

C 4.76544600 0.30561800 -3.71715000

C 5.96607800 1.07091800 -3.56540400

C 7.23405400 0.43827400 -3.73563400

C 8.43106500 1.19433700 -3.54502200

C 8.58529000 -1.60931200 -3.50438900

C 9.77895100 -0.85877200 -3.73670300

C 11.04927200 -1.48766200 -3.57116500

C 12.24240700 -0.72897100 -3.77226100

C 12.40349400 -3.14816500 -2.35448300

C 13.59561600 -2.60673500 -2.92598600

C 14.86412800 -3.06213800 -2.46460200

C 16.05646000 -2.49141300 -3.00520600

C 16.21929000 -3.88216200 -0.58250900

C 17.41796100 -3.70354200 -1.34447600

C 18.68420600 -3.91139400 -0.73065800

C 19.83587400 -3.74117900 -1.56368500

C 20.00415100 -3.74682600 1.41193500

C -1.74541400 3.65903900 0.64728700

C 0.77370100 3.66184400 0.76750600

C 2.11180700 3.45237900 -1.25210800

C 3.30731100 3.63737100 -0.51656400

C 4.57169800 3.47646800 -1.15996300

C 5.76997000 3.63636900 -0.39895700

C 5.89815300 2.31499500 -2.87145400

C 7.09568800 2.85819900 -2.31918000

C 8.36218500 2.40956000 -2.80365200

C 9.56143600 2.93141300 -2.22694600

C 9.70281800 0.56659500 -3.73326700

C 10.89697300 1.31532000 -3.51662200

C 12.16677700 0.69242000 -3.71995400

C 13.36433800 1.43430100 -3.47464200

C 13.51601100 -1.36422000 -3.62639900

C 14.70547200 -0.60185700 -3.79978900

C 15.97750300 -1.23701400 -3.65939400

C 17.17964800 -0.47352100 -3.81720000

C 17.33655700 -2.96780600 -2.57177800

C 18.52138600 -2.42538100 -3.13863000

C 19.75798200 -3.01397800 -2.72411000

C 3.23865300 3.55416700 0.90379900

C 5.70063500 3.50389200 1.01895000

C 7.03183800 3.50671000 -1.04759000

C 8.22795500 3.63297500 -0.28057700

C 9.49480700 3.52995000 -0.93335500

C 10.68936700 3.63736700 -0.16367900

C 10.82708100 2.50552800 -2.72887400

C 12.02303000 3.00332300 -2.13007500

C 13.29362000 2.59491400 -2.64558100

C 14.48587300 3.07277900 -2.03365800

C 14.62994000 0.82055400 -3.69798200

C 15.82499200 1.54663700 -3.41122900

C 17.10343400 0.94922900 -3.66389400

C 18.29049600 1.69066700 -3.41991400

C 18.44369000 -1.12229400 -3.75645500

C 19.59592400 -0.29851700 -3.94893100

C 8.15855200 3.45504800 1.13192300

C 11.95495600 3.55602900 -0.81871400

C 13.15323700 3.63899200 -0.04435100

C 14.41824600 3.58476600 -0.69988700

C 15.61199000 3.63102000 0.07364700

C 15.75296800 2.67084200 -2.55171700

C 16.95887600 3.13727600 -1.93309900

C 18.21848400 2.80933100 -2.50449900

C 19.37031200 3.34370200 -1.85412200

C 19.52266600 1.06221900 -3.77759700

C 16.89115800 3.60509300 -0.58002900

C 18.08243500 3.70518400 0.18404700

C 19.30382900 3.77096800 -0.54810000

H -21.24865700 2.92262900 2.37585900

H -20.12074300 1.11728700 3.75460800

H -20.17571400 -0.98811300 4.21805900

H -20.39388900 -4.27689700 1.23412900

H -20.28823900 -3.12674000 3.09785400

H -20.53288400 -4.17415200 -1.17771200

H -20.64326400 -3.03572600 -3.04565500

H -20.90512000 1.36768200 -3.71306500

H -20.79007500 -0.81056400 -3.98493700

H -21.04829800 3.12043300 -2.04896500

H 20.15749100 3.24322300 1.97002400

H -21.15806300 3.68300600 0.05816000

H 21.26732500 1.59005900 3.54345500

H 21.16482700 -0.82084600 3.91180800

H 21.05567800 -2.79616900 2.98761200

H 20.81087000 -4.06284500 -1.21166500

H 20.91829800 -4.14260900 0.98035000

H 20.67329300 -2.78190200 -3.25973800

H 20.57047200 -0.75547600 -4.08842100

H 20.34252600 3.27985900 -2.33252800

H 20.44126600 1.64065300 -3.78857100

H 20.22624000 4.03344900 -0.03926200

C -0.65245800 5.19305000 2.26262900

C -0.26893900 5.21677300 -2.20707000

C -1.42391900 5.76349300 -3.09664100

C -1.04772200 7.03550000 -3.92343500

C -2.27570000 7.80281800 -4.48915300

C 0.49670400 5.74222100 3.15822400

C 0.10731900 7.00146700 3.99829100

C 1.32721000 7.77505000 4.57287300

F 0.83809500 5.23568400 -3.00859800

F -0.08667600 6.13884000 -1.22575700

F -1.80480000 4.82129500 -3.99418400

F -2.48220600 6.07979700 -2.30709100

F -0.34880200 7.90657700 -3.16095800

F -0.28888700 6.65884200 -4.97697700

F -1.84675200 8.71391400 -5.37263500

F -2.93443600 8.43339700 -3.51098100

F -3.11389000 6.96179100 -5.11164000

F -1.75989200 5.19300400 3.06381400

F -0.84357100 6.12312100 1.29058400

F 0.88720200 4.79453500 4.04582700

F 1.55174100 6.07778900 2.37225800

F -0.59993700 7.87346500 3.24459200

F -0.64821300 6.60610000 5.04732800

F 2.17344300 6.93598200 5.18706200

F 1.98015200 8.42249600 3.60188300

F 0.88860900 8.67243400 5.46561200

**(6,5) SWNT-[(CF_2_)_3_CF_3_]_2_ (1,4-L_+_: L_27_)**

C 20.24960700 1.43455600 -3.37484500

C 19.06125400 0.66937900 -3.64864700

C 19.10065500 -0.71582600 -3.85988700

C 17.94323200 -1.50237000 -3.68809800

C 17.80618400 1.29410500 -3.37342300

C 16.60966500 0.57761200 -3.64528300

C 15.34282900 1.21363400 -3.42814800

C 14.14116400 0.49715100 -3.68920100

C 19.21344200 -3.39149700 -2.58049900

C 17.99358900 -2.79983900 -3.02297800

C 16.80192000 -3.28514400 -2.42597700

C 16.67335900 -0.83964900 -3.76325600

C 15.46176000 -1.58125800 -3.58642700

C 14.19952600 -0.93016000 -3.74183500

C 12.99915200 -1.66850400 -3.53589300

C 12.88048000 1.13621200 -3.48584700

C 11.67397300 0.40182300 -3.70365800

C 10.41191700 1.04787900 -3.52284600

C 9.20872300 0.30536900 -3.70579500

C 19.44781800 -3.91353100 1.60080400

C 19.28154400 -4.05924700 -1.37979300

C 18.13298100 -4.16431400 -0.53977500

C 16.87172600 -3.96154900 -1.16380800

C 15.66913800 -4.09229700 -0.39618600

C 15.52365300 -2.81120800 -2.88101000

C 14.32901500 -3.34066200 -2.31711300

C 13.06383600 -2.88426800 -2.79241900

C 11.86615800 -3.39195500 -2.20231300

C 11.73276500 -1.02197300 -3.70331900

C 10.53275800 -1.75797100 -3.47442100

C 9.26883100 -1.11970700 -3.65927400

C 8.06943300 -1.85062600 -3.40464500

C 7.94439800 0.95504500 -3.53812000

C 6.74165700 0.20360500 -3.69507000

C 5.47990300 0.85351700 -3.53914800

C 4.27935400 0.09493700 -3.66808700

C 2.95663700 2.03608600 -2.91256900

C 0.50250300 1.96045100 -2.97304700

C 19.52866900 -3.11685800 2.71643100

C 18.38100900 -2.41217800 3.19646200

C 18.21183400 -4.07342800 0.90216100

C 17.02907400 -3.74341900 1.61696900

C 15.74786900 -3.95900500 1.01231000

C 14.55721700 -3.67042400 1.74507700

C 14.39979100 -3.97661900 -1.03813000

C 13.21112800 -4.09892000 -0.26676500

C 11.93891700 -3.98930200 -0.91092800

C 10.74729800 -4.09158500 -0.13308000

C 10.59909900 -2.95231500 -2.69371600

C 9.40548800 -3.44517100 -2.09111000

C 8.13715900 -3.02059300 -2.59310100

C 6.94276800 -3.50217700 -1.97829900

C 6.80353200 -1.22087000 -3.60922400

C 5.60799200 -1.94719600 -3.33697800

C 4.34193900 -1.32307400 -3.55371200

C 3.14468800 -2.04441100 -3.26691000

C 3.01606700 0.74764500 -3.52815800

C 1.82114100 -0.00642900 -3.64795700

C 0.56021200 0.65649800 -3.52773100

C -0.64063000 -0.11614500 -3.65644200

C -1.97826100 1.85700400 -3.08136900

C -4.44387400 1.76826300 -3.20828200

C 19.70710600 -0.38030500 3.89227200

C 18.46655200 -1.07689600 3.73837400

C 17.28578800 -0.28559100 3.75589700

C 17.11313900 -2.89007300 2.76495600

C 15.91564400 -2.25015300 3.22269200

C 14.64008400 -2.77785100 2.85565400

C 13.45438500 -2.12625600 3.30051700

C 13.28881000 -3.92062900 1.14845500

C 12.09688400 -3.59214000 1.86481400

C 10.82469200 -3.86463500 1.27470400

C 9.63510100 -3.51325200 1.97771800

C 9.47863000 -4.00555600 -0.78034600

C 8.28603200 -4.08159600 -0.00020100

C 7.01574400 -4.02118300 -0.65122400

C 5.82308300 -4.07498200 0.12928700

C 5.67537100 -3.09733100 -2.49423400

C 4.48087300 -3.55997600 -1.86553300

C 3.21146700 -3.17063000 -2.39428100

C 2.01587500 -3.61240700 -1.75446400

C 1.88151300 -1.42702800 -3.50436200

C 0.68566900 -2.13570500 -3.19947900

C -0.57921300 -1.52148600 -3.46241900

C -1.78074000 -2.22719700 -3.14828300

C -1.90758100 0.53710500 -3.60590000

C -3.10506700 -0.23170200 -3.71144700

C -4.37302800 0.42783800 -3.67933300

C -5.57403000 -0.34611500 -3.73826100

C -6.90711900 1.67463300 -3.29063600

C -9.37331700 1.57575500 -3.35793700

C 19.97324100 3.15228300 1.62597700

C 19.79253600 0.96566700 3.64281400

C 18.64492100 1.70052000 3.20372900

C 17.37515900 1.11534100 3.46725700

C 16.18029800 1.80425700 3.08384000

C 16.00213500 -0.91918900 3.70024300

C 14.81356100 -0.12780100 3.75406600

C 13.54068200 -0.76782900 3.73398800

C 12.35219300 0.02546100 3.74229900

C 12.17883400 -2.66047500 2.93910700

C 10.98877000 -1.98865200 3.35749800

C 9.71584600 -2.53802700 3.01791700

C 8.52597600 -1.84950100 3.40370100

C 8.36328900 -3.80535900 1.39628200

C 7.17107600 -3.42817500 2.08567600

C 5.89977100 -3.74658100 1.51650000

C 4.70804200 -3.34146500 2.18738800

C 4.55314100 -4.03599100 -0.52369900

C 3.35812100 -4.05976500 0.25864600

C 2.08643400 -4.04330300 -0.39475200

C 0.89257000 -4.03369600 0.38513800

C 0.74912400 -3.23456600 -2.29485100

C -0.45030000 -3.64700500 -1.63897000

C -1.71709700 -3.28791200 -2.19679900

C -2.91494700 -3.67383700 -1.52618500

C -3.04300600 -1.63197600 -3.44626100

C -4.24510100 -2.31700800 -3.09204200

C -5.51038700 -1.73333100 -3.41198200

C -6.71037100 -2.40244600 -3.02640400

C -6.83877900 0.31557000 -3.72251000

C -8.03944800 -0.45837800 -3.74240800

C -9.30630100 0.20417600 -3.74577600

C -10.50551800 -0.56875900 -3.73758700

C -10.63946100 2.14214500 -3.01956500

C -11.83662400 1.47635600 -3.41784400

C -13.10531700 2.05543500 -3.09603300

C -14.30226700 1.38116200 -3.47276800

C 20.05114900 3.46526000 0.29499300

C 18.89367200 3.37085700 -0.54697600

C 18.73105400 2.73984800 2.21014700

C 17.54774300 3.09228800 1.50513200

C 16.26702100 2.79203900 2.07137100

C 15.07579700 3.17745300 1.38269400

C 14.90274200 1.25464000 3.41501000

C 13.71886200 1.94042100 3.01962200

C 12.44183700 1.39428400 3.35687000

C 11.25494300 2.06826700 2.93465100

C 11.07508000 -0.61676900 3.73915100

C 9.88797900 0.17629100 3.71933300

C 8.61210500 -0.46484000 3.73240800

C 7.42371600 0.32889300 3.67558100

C 7.25151000 -2.41254100 3.08350500

C 6.06095300 -1.70870900 3.43831500

C 4.78889700 -2.28279600 3.13943800

C 3.59893000 -1.55810600 3.45476600

C 3.43546800 -3.67976700 1.62956200

C 2.24291500 -3.24834500 2.28571800

C 0.97009900 -3.60488000 1.74507000

C -0.21824700 -3.14403500 2.38527100

C -0.37918600 -4.02880700 -0.26834700

C -1.57377000 -3.98610200 0.51391900

C -2.84374000 -4.00390900 -0.13915500

C -4.03564300 -3.93059800 0.64141500

C -4.18101400 -3.33418600 -2.09534200

C -5.37933200 -3.69327200 -1.40670100

C -6.64521700 -3.37983700 -1.98791300

C -7.84111400 -3.71171900 -1.28414400

C -7.97548000 -1.83109000 -3.36395600

C -9.17634200 -2.48502600 -2.94881400

C -10.44157600 -1.92915000 -3.30784100

C -11.63972300 -2.56474900 -2.86440300

C -11.77059100 0.09411500 -3.75884000

C -12.97204400 -0.67881300 -3.71882100

C -14.23629200 -0.01681800 -3.76413900

C -15.43307600 -0.78223600 -3.68151500

C -15.56693600 1.96817700 -3.16285000

C -16.77482600 1.28408000 -3.51287900

C -18.04775600 1.90187600 -3.27902500

C -19.20108700 1.17381000 -3.63638300

C 20.19976800 2.49784500 -2.51943300

C 18.96128400 2.88913500 -1.89709700

C 17.75877000 2.42685300 -2.50312000

C 17.62749400 3.39816900 0.10702700

C 16.42481000 3.34820000 -0.66502400

C 15.15370700 3.43814200 -0.01578900

C 13.96140100 3.37432500 -0.79313800

C 13.80581000 2.90503400 1.96924800

C 12.61667800 3.25738300 1.26307300

C 11.34344900 2.99892000 1.85748200

C 10.15616600 3.33543100 1.14231000

C 9.97862300 1.53529700 3.28974100

C 8.79380700 2.19488500 2.84519000

C 7.51649300 1.66900400 3.20666400

C 6.33015500 2.31559300 2.73380500

C 6.14567500 -0.30989300 3.70603300

C 4.96058600 0.47845000 3.60151500

C 3.68313200 -0.15421400 3.65082000

C 2.49504700 0.63798000 3.52348100

C 2.32421900 -2.15126900 3.19130100

C 1.13999900 -1.42363100 3.49707200

C -0.13314300 -2.01996000 3.25890200

C -1.31864600 -1.27956100 3.54683600

C -1.49391100 -3.51206700 1.85626000

C -2.68064400 -3.03041300 2.48512600

C -3.95446900 -3.41350500 1.96824700

C -5.14143500 -2.91314300 2.58357400

C -5.30671200 -3.97089300 -0.00967100

C -6.49733400 -3.87514800 0.76996300

C -7.76728100 -3.93927400 0.12187400

C -8.95838400 -3.81950100 0.89956100

C -9.10930900 -3.42079600 -1.87486400

C -10.30415500 -3.73091000 -1.15940700

C -11.57138000 -3.46137400 -1.75814900

C -12.76702500 -3.74671400 -1.02970200

C -12.90778600 -2.02004800 -3.23997500

C -14.10518300 -2.64044800 -2.77467100

C -15.37017200 -2.10427700 -3.15994600

C -16.56966400 -2.70592800 -2.66544500

C -16.70339400 -0.11954200 -3.74017600

C -17.89551000 -0.88728700 -3.64896700

C -19.15399400 -0.22122500 -3.76622000

C -20.33792600 -1.03631800 -3.68514100

C 16.49023600 2.83262300 -1.98318000

C 15.28759500 2.37318000 -2.60611400

C 14.02543400 2.81090400 -2.10430500

C 12.82445400 2.32688500 -2.70293400

C 12.69358600 3.47401300 -0.14376700

C 11.49761000 3.38222800 -0.91973600

C 10.23155400 3.51020100 -0.27329600

C 9.03623200 3.39913700 -1.04328900

C 8.88505700 3.09607500 1.74183300

C 7.70018300 3.41561500 1.01494100

C 6.42673700 3.19489200 1.61904700

C 5.24467600 3.53129000 0.90397500

C 5.05281800 1.79761300 3.07928200

C 3.86943200 2.43953600 2.57422700

C 2.57398800 1.94177600 2.97064500

C 1.38411500 2.57111700 2.49301200

C 1.22348600 -0.00438900 3.64254000

C 0.04103000 0.76932600 3.52393000

C -1.23287000 0.13724500 3.66307600

C -2.42071200 0.91550100 3.53552900

C -2.59461400 -1.88240900 3.32928800

C -3.77829500 -1.13669000 3.60222700

C -5.05473100 -1.74510000 3.39670800

C -6.24169100 -0.99508000 3.65219000

C -6.41560400 -3.31613900 2.08087600

C -7.60175600 -2.80233200 2.68242600

C -8.87633800 -3.22148300 2.19128500

C -10.06458600 -2.69443800 2.77990500

C -10.22932300 -3.91036000 0.25522800

C -11.41859700 -3.76826800 1.02843300

C -12.69101900 -3.87921600 0.38680800

C -13.88251000 -3.71652400 1.15625400

C -14.03639000 -3.50154700 -1.63728600

C -15.22801500 -3.75852600 -0.90012000

C -16.50022300 -3.52225300 -1.50954600

C -17.70245200 -3.76360400 -0.77365700

C -17.84089400 -2.19556200 -3.07560700

C -19.04024700 -2.79605200 -2.59866900

C -20.28139600 -2.27371400 -3.10998400

C 11.55990300 2.77679300 -2.20869800

C 10.35617300 2.27289900 -2.78829100

C 9.09584700 2.74090200 -2.30988100

C 7.88827300 2.20566800 -2.85577000

C 7.77270200 3.55387100 -0.39924700

C 6.56677800 3.42295900 -1.15980800

C 5.31499800 3.62453700 -0.52569700

C 4.09861200 3.42021900 -1.25308700

C 3.98200900 3.33595600 1.51291800

C 2.77010500 4.00635700 0.87235200

C 1.43208900 3.47625400 1.38477600

C 0.27331000 3.74185200 0.66192600

C 0.12154600 2.05745000 2.91017200

C -1.07172000 2.65764200 2.42206200

C -2.34363600 2.18618800 2.89012400

C -3.53625300 2.77437900 2.38976200

C -3.69297300 0.28613700 3.69021600

C -4.88359200 1.05783900 3.53496000

C -6.15813300 0.42930000 3.70181700

C -7.34885300 1.19205300 3.51935300

C -7.51591600 -1.61130700 3.46382300

C -8.70502600 -0.85569500 3.69467100

C -9.98018200 -1.47959400 3.52568600

C -11.16836700 -0.71931100 3.73191900

C -11.33678700 -3.13029900 2.30226900

C -12.52651300 -2.58366400 2.87272400

C -13.80107800 -3.03716800 2.41036100

C -14.98755800 -2.47356300 2.95909300

C -15.15118900 -3.84475800 0.51988600

C -16.34443200 -3.64367500 1.28003500

C -17.62399800 -3.80415600 0.65680300

C -18.80886600 -3.63838300 1.42463100

C -18.96910600 -3.58688300 -1.40317500

C -20.12592400 -3.88638600 -0.60987100

C 6.62601100 2.69221700 -2.39125400

C 5.42371600 2.12420300 -2.89225800

C 4.15952000 2.61600500 -2.42375500

C 2.83257500 3.72071800 -0.66294900

C 1.66945400 3.47450600 -1.38581100

C 0.34050200 4.02683400 -0.87327500

C -0.88243600 3.37680800 -1.51412200

C -0.99762500 3.46181300 1.25198700

C -2.21034900 3.68683700 0.52499800

C -3.46497500 3.50502300 1.15905300

C -4.66897900 3.65625200 0.39865600

C -4.80664300 2.30843200 2.85414600

C -6.00482900 2.86459900 2.30982100

C -7.27225900 2.41671400 2.78740900

C -8.46835800 2.94234500 2.20908300

C -8.62261900 0.56742700 3.69897000

C -9.81600200 1.32294200 3.48557300

C -11.08687900 0.70547800 3.68660300

C -12.27960600 1.45145200 3.43780700

C -12.44304300 -1.34858800 3.57770300

C -13.63474700 -0.58137900 3.75843800

C -14.90462200 -1.21333300 3.62279700

C -16.09630000 -0.43730900 3.75931400

C -16.26262300 -2.93142100 2.50221600

C -17.46064000 -2.36144500 3.04023000

C -18.72770100 -2.87016300 2.64050100

C -19.87891000 -2.26741500 3.24134100

C -20.04928200 -3.90268700 0.75728800

C 1.70257300 2.56968900 -2.49465400

C -0.78462900 2.47888300 -2.57587200

C -2.14159500 3.59271200 -0.90521400

C -3.32888300 3.27527500 -1.61991100

C -4.59885200 3.51680300 -1.01557100

C -5.78863000 3.21738600 -1.74226000

C -5.93440700 3.52296300 1.04286200

C -7.12733500 3.65278800 0.27312600

C -8.39590200 3.54634900 0.92000600

C -9.58989000 3.65918700 0.14551300

C -9.73932800 2.51633400 2.70461300

C -10.93120500 3.02179100 2.10747900

C -12.20201200 2.61149800 2.61505300

C -13.39671400 3.09791500 1.99926800

C -13.55377000 0.84156300 3.66149300

C -14.74197700 1.57536100 3.38509000

C -16.01527400 0.96975300 3.61484000

C -17.21568500 1.69615600 3.32426000

C -17.37744400 -1.07143300 3.65940200

C -18.56126500 -0.30415000 3.83188300

C -19.79928900 -1.02137100 3.80886500

C -3.24684100 2.39508500 -2.73492000

C -5.71236900 2.31474800 -2.84666900

C -7.05496900 3.47653800 -1.14209700

C -8.24841200 3.15881300 -1.85694600

C -9.51691500 3.43994500 -1.26354900

C -10.70980700 3.10725800 -1.96853600

C -10.85722800 3.58146400 0.79501000

C -12.05001300 3.66723900 0.01678500

C -13.32181200 3.61229200 0.66879400

C -14.51148400 3.67633600 -0.10827700

C -14.66359900 2.70761100 2.51891300

C -15.85637800 3.15880900 1.87770300

C -17.13622500 2.79959700 2.41362900

C -18.32123200 3.28705300 1.79993400

C -18.48116000 1.12332900 3.62846700

C -19.63185200 1.91837100 3.33258100

C -8.17559400 2.22785400 -2.93400900

C -11.97728200 3.40515600 -1.38189400

C -13.17417900 3.05233400 -2.07775600

C -14.44023700 3.37125000 -1.50381800

C -15.63343200 2.98908300 -2.17902300

C -15.78023200 3.62757300 0.54265300

C -16.98379700 3.68071100 -0.23324400

C -18.24497400 3.72123100 0.42142400

C -19.39451700 3.81491800 -0.41829800

C -19.55536600 2.96002000 2.44086800

C -16.91308400 3.33057600 -1.62152000

C -18.10347400 2.99856800 -2.31823700

C -19.32538900 3.45797500 -1.74503500

H 21.20662400 1.07542400 -3.74505000

H 20.07643600 -1.19013200 -3.89994900

H 20.13437300 -3.20331000 -3.12380300

H 20.36319600 -4.31691300 1.17899800

H 20.25329000 -4.37489800 -1.01345300

H 20.50585600 -2.91271900 3.14239300

H 20.61905300 -0.93677200 4.08515400

H 20.88788200 3.09916900 2.20836700

H 20.77000100 1.43751300 3.64199200

H 21.02579900 3.65176100 -0.14456100

H -20.17971700 1.63763700 -3.55985800

H 21.12523600 2.97973900 -2.22006200

H -21.29725600 -0.60423400 -3.95877800

H -21.20387700 -2.81881600 -2.93605700

H -21.09969100 -3.96617600 -1.08246500

H -20.85465200 -2.72784700 3.12320800

H -20.96394900 -3.99544500 1.33465500

H -20.71391300 -0.53037200 4.12662900

H -20.60763700 1.61432900 3.69783900

H -20.36741000 4.02497700 0.01472700

H -20.47270400 3.44976000 2.12915700

H -20.24652700 3.40043600 -2.31676100

C 2.87775100 5.55710600 1.26445800

C 0.25933500 5.57917400 -1.26530400

C -0.25192900 5.93740500 -2.70225300

C 0.07868400 7.39951400 -3.14262200

C -0.75929000 7.89478400 -4.35510400

C 3.39380600 5.90621400 2.70190400

C 3.08839100 7.37391200 3.14191900

C 3.93489700 7.85497600 4.35419300

F 1.49308100 6.15572800 -1.21573600

F -0.55821800 6.21870300 -0.39081300

F 0.32932800 5.11032600 -3.60800500

F -1.59894600 5.79263600 -2.77281300

F 1.37930100 7.47047700 -3.50123400

F -0.15236700 8.25367800 -2.11872600

F -0.22895800 9.03710200 -4.81139100

F -2.02682900 8.12652500 -3.99740700

F -0.74037000 6.98750700 -5.34246500

F 3.70729600 6.18229400 0.39086200

F 1.65428700 6.15506700 1.21363000

F 2.79712800 5.08955700 3.60705300

F 4.73797000 5.73757600 2.77382100

F 1.78918800 7.46705300 3.50058500

F 3.33380100 8.22386500 2.11786800

F 3.90207200 6.94761400 5.34112000

F 5.20576800 8.06659200 3.99578600

F 3.42320000 9.00536300 4.81144300

**(6,5) SWNT-[(CF_2_)_3_CF_3_]_2_ (1,4-L–: L_–33_)**

C -20.55863300 -1.37831900 -3.46069000

C -19.37300100 -0.59354700 -3.68565900

C -19.41698200 0.80217700 -3.80908000

C -18.26186200 1.57998200 -3.59061300

C -18.11581500 -1.23006700 -3.45102600

C -16.92190700 -0.49395100 -3.67796100

C -15.65272300 -1.13778800 -3.50125200

C -14.45430200 -0.40258000 -3.71943800

C -19.53578000 3.39372600 -2.36747500

C -18.31505100 2.83395500 -2.84653400

C -17.12414200 3.28396100 -2.22095500

C -16.99007000 0.92739400 -3.70810700

C -15.78039100 1.66064900 -3.48767300

C -14.51680700 1.02501200 -3.68547000

C -13.31806600 1.75332200 -3.43583300

C -13.19116000 -1.04821800 -3.55494500

C -11.98753600 -0.29802300 -3.73293400

C -10.72425300 -0.94923700 -3.59153400

C -9.52265700 -0.19294300 -3.73622900

C -19.76989600 3.65490700 1.83838300

C -19.60471000 3.98586000 -1.12757000

C -18.45613500 4.04128700 -0.28309700

C -17.19485700 3.87963900 -0.91905000

C -15.99191900 3.96343700 -0.14489600

C -15.84494200 2.84344700 -2.70591400

C -14.65120700 3.33924800 -2.11079700

C -13.38526200 2.91773000 -2.61536300

C -12.18747700 3.38847100 -1.99434600

C -12.04982500 1.12337600 -3.64697200

C -10.85133000 1.84695100 -3.37410500

C -9.58540500 1.22526100 -3.60179500

C -8.38522500 1.94038000 -3.30124400

C -8.25646400 -0.84809100 -3.61255200

C -7.05485800 -0.08692100 -3.72863400

C -5.79042500 -0.74534500 -3.61743200

C -4.58510800 0.01996700 -3.69624800

C -3.26615500 -1.95865200 -3.05831100

C -0.78264200 -1.88126500 -3.07080100

C -19.84959900 2.78893800 2.90145000

C -18.70123600 2.05656900 3.33549600

C -18.53448700 3.86015000 1.15091900

C -17.35134300 3.48662700 1.84306600

C -16.07029700 3.74093300 1.25263500

C -14.87959900 3.40859300 1.96571800

C -14.72292700 3.89103200 -0.79314700

C -13.53418000 3.96394200 -0.01576700

C -12.26157300 3.89796100 -0.66578200

C -11.07048000 3.95023700 0.11661200

C -10.92003700 2.98591400 -2.51486100

C -9.72635000 3.43732800 -1.87962300

C -8.45608400 3.05061300 -2.40950200

C -7.26291900 3.48836700 -1.76170400

C -7.11833100 1.32800300 -3.54832800

C -5.92108400 2.03363700 -3.22249400

C -4.65099400 1.42704200 -3.47915100

C -3.45539900 2.12521300 -3.13440000

C -3.32173900 -0.63942400 -3.58875400

C -2.11942200 0.12192500 -3.63426400

C -0.85190800 -0.54741300 -3.53901100

C 0.34440500 0.23331400 -3.60621500

C 1.67088500 -1.77657200 -3.11980800

C 4.13339800 -1.67565600 -3.23092500

C -20.02407200 -0.01811800 3.89806700

C -18.78480400 0.68894400 3.79131500

C -17.60257700 -0.09934100 3.76103200

C -17.43417300 2.56242300 2.93503900

C -16.23572800 1.89694300 3.35242700

C -14.96109900 2.44877600 3.02011000

C -13.77524800 1.77312100 3.42495000

C -13.61190800 3.69561500 1.38498900

C -12.41937100 3.32463600 2.07988900

C -11.14837200 3.63420600 1.50790300

C -9.95947100 3.24109400 2.18833500

C -9.80190500 3.90627100 -0.53437600

C -8.60928300 3.93236500 0.25001700

C -7.33966800 3.91632200 -0.40178200

C -6.14936400 3.92029800 0.38314800

C -5.99366800 3.11882800 -2.30065900

C -4.79977400 3.54024700 -1.63796800

C -3.52933200 3.19092000 -2.18696000

C -2.33842600 3.59386700 -1.51158600

C -2.18768100 1.52130000 -3.39528200

C -0.99159600 2.21942100 -3.03771900

C 0.27409000 1.63242700 -3.33275200

C 1.46747800 2.31697900 -2.96575400

C 1.60194000 -0.42039100 -3.56968100

C 2.79819100 0.35454800 -3.61569500

C 4.06273900 -0.30621600 -3.61990600

C 5.25881700 0.47035100 -3.63907500

C 6.59456500 -1.57394500 -3.33462900

C 9.06089600 -1.46238600 -3.40660600

C -20.27780100 -3.40747600 1.41912200

C -20.10615900 -1.34619900 3.56504700

C -18.95603900 -2.05016300 3.08451100

C -17.68824400 -1.47978600 3.38614900

C -16.49081100 -2.14084000 2.96299000

C -16.31998500 0.53894700 3.74708400

C -15.12999300 -0.25156200 3.75544600

C -13.85890400 0.39111000 3.77677800

C -12.66834200 -0.39900100 3.74020400

C -12.49991900 2.33028300 3.09714400

C -11.30978300 1.63818200 3.47768800

C -10.03894900 2.20767100 3.17027500

C -8.84725500 1.50058900 3.51912500

C -8.68764700 3.56953100 1.62656100

C -7.49693700 3.15607300 2.29452700

C -6.22802500 3.50854500 1.74649600

C -5.03545000 3.07016100 2.39796200

C -4.87767800 3.92712200 -0.26792700

C -3.68660400 3.91096600 0.51805700

C -2.41623300 3.93900400 -0.13117800

C -1.22183600 3.89191800 0.65463500

C -1.06568400 3.25779900 -2.06949700

C 0.12917700 3.64382600 -1.38656900

C 1.39597900 3.32590100 -1.95688900

C 2.59236700 3.67447700 -1.25887000

C 2.73151100 1.73690900 -3.28243600

C 3.93082600 2.40568900 -2.89069400

C 5.19279200 1.84039000 -3.23905500

C 6.39113300 2.48834100 -2.81645500

C 6.52397800 -0.18841300 -3.66757500

C 7.72267000 0.59016800 -3.64632000

C 8.98964700 -0.06562700 -3.69881000

C 10.18683300 0.71011200 -3.64341200

C 10.33294200 -2.04269500 -3.10724300

C 11.52645300 -1.34656200 -3.46195900

C 12.79829200 -1.93803300 -3.17690700

C 13.99240100 -1.23576100 -3.51028200

C -20.35345800 -3.63788900 0.07116400

C -19.19586900 -3.48638400 -0.76213800

C -19.03773900 -3.02716900 2.02895900

C -17.85270400 -3.33128200 1.30495900

C -16.57328000 -3.06300300 1.89053200

C -15.37991100 -3.40018200 1.18064900

C -15.21511700 -1.61117100 3.33146500

C -14.02917900 -2.26724300 2.89582600

C -12.75349200 -1.74104300 3.27068000

C -11.56365600 -2.38376600 2.81021500

C -11.39247600 0.24460900 3.78030800

C -10.20504700 -0.54262000 3.71672600

C -8.92878600 0.09941400 3.77072000

C -7.73851400 -0.68569800 3.67957200

C -7.57558700 2.08438900 3.23521200

C -6.38557700 1.36643800 3.55533600

C -5.11409800 1.96281400 3.29264500

C -3.92239000 1.23507500 3.58524700

C -3.76576800 3.44937700 1.86704300

C -2.57486400 2.98728600 2.50029100

C -1.30240000 3.38349000 1.98265500

C -0.10988600 2.89807100 2.60083900

C 0.05093300 3.94444700 0.00745800

C 1.24253600 3.86461200 0.78820200

C 2.51546100 3.92425200 0.14157900

C 3.70944200 3.81125500 0.92101300

C 3.86116600 3.37132700 -1.84360100

C 5.05619300 3.69397700 -1.13516300

C 6.32264700 3.40986800 -1.72975200

C 7.51824300 3.70525900 -1.00842200

C 7.65601300 1.93885800 -3.18726800

C 8.85397400 2.57145800 -2.73805600

C 10.11965000 2.04072400 -3.13038600

C 11.31646600 2.65464500 -2.65328200

C 11.45482900 0.05567700 -3.71217000

C 12.65267500 0.82948800 -3.62532600

C 13.92033500 0.17719100 -3.71365400

C 15.11406700 0.94136600 -3.58586900

C 15.26004400 -1.83480800 -3.23671100

C 16.46453200 -1.12556200 -3.54437900

C 17.74057500 -1.75045000 -3.34769900

C 18.89037600 -0.99785600 -3.66196900

C -20.50481000 -2.49420300 -2.67535500

C -19.26477500 -2.92013900 -2.07930300

C -18.06414900 -2.41560600 -2.65405100

C -17.93012000 -3.54918500 -0.10973500

C -16.72701600 -3.44501000 -0.87598900

C -15.45575600 -3.57030700 -0.23225500

C -14.26356100 -3.45100100 -1.00211100

C -14.11157900 -3.16165200 1.78509000

C -12.91972400 -3.46188400 1.05895200

C -11.64707400 -3.24031800 1.67170100

C -10.45815400 -3.52109500 0.93858200

C -10.29014000 -1.87256400 3.20305800

C -9.10119000 -2.49825700 2.71938500

C -7.82302600 -2.00075000 3.12643900

C -6.63758300 -2.61448600 2.62708300

C -6.46602900 -0.04495900 3.75204900

C -5.27414300 -0.82533700 3.62391500

C -4.00199600 -0.18550000 3.72036500

C -2.81354600 -0.95595200 3.55376400

C -2.65484200 1.84302700 3.34772100

C -1.46184700 1.10102800 3.60503600

C -0.19184700 1.71792300 3.40144800

C 0.99500300 0.96563700 3.63124000

C 1.16036500 3.30696900 2.10036800

C 2.35140700 2.78760300 2.69631000

C 3.62750100 3.21216400 2.21128700

C 4.81468700 2.67712000 2.79637300

C 4.98047500 3.89439100 0.27637700

C 6.17139300 3.75209800 1.05018100

C 7.44283700 3.85526700 0.40757700

C 8.63371800 3.69156300 1.17680400

C 8.78515500 3.44809000 -1.61430900

C 9.97961400 3.71937000 -0.88339100

C 11.24675200 3.48649600 -1.49745400

C 12.44177700 3.73300000 -0.75534900

C 12.58480300 2.13882100 -3.06373700

C 13.78080800 2.73499900 -2.56382600

C 15.04721500 2.22895500 -2.98365200

C 16.24490100 2.80439100 -2.45518700

C 16.38702900 0.28889400 -3.68639300

C 17.57626400 1.05454700 -3.54995000

C 18.83729900 0.40216800 -3.70863900

C 20.01798200 1.21556900 -3.58046400

C -16.79399300 -2.84791100 -2.15920000

C -15.59310600 -2.34569200 -2.75160900

C -14.32958500 -2.80729300 -2.27578400

C -13.13072400 -2.28168900 -2.84293600

C -12.99511700 -3.58504900 -0.35832200

C -11.79888300 -3.43737500 -1.12456000

C -10.53338700 -3.59773300 -0.48551300

C -9.33852600 -3.42347100 -1.24350900

C -9.18622200 -3.31858000 1.55709800

C -7.99546000 -3.58176300 0.81577000

C -6.72412200 -3.40813600 1.44156500

C -5.53730600 -3.65286400 0.69175200

C -5.35776900 -2.12106200 3.03411100

C -4.16912300 -2.72043900 2.51361300

C -2.89685900 -2.22653700 2.91212500

C -1.70527100 -2.79994400 2.35282300

C -1.54193900 -0.31843700 3.67259400

C -0.35288800 -1.08213800 3.47953200

C 0.91121600 -0.45901800 3.63642500

C 2.10049600 -1.22389600 3.42097600

C 2.26680000 1.58541400 3.45043300

C 3.45611600 0.82165400 3.66785300

C 4.73005400 1.45198100 3.52368000

C 5.91942300 0.69071800 3.72909900

C 6.08915500 3.11354800 2.32316500

C 7.27814700 2.56297100 2.89204700

C 8.55222300 3.01561900 2.43040800

C 9.74175100 2.45363400 2.98388800

C 9.90439900 3.81955900 0.53843300

C 11.09485500 3.63364700 1.30143200

C 12.36629900 3.78391500 0.66672900

C 13.55861400 3.57740400 1.42391400

C 13.71084300 3.52656200 -1.37717000

C 14.90266100 3.74329300 -0.62721600

C 16.17448400 3.54820700 -1.25092900

C 17.37682800 3.74749100 -0.50253500

C 17.51755000 2.32518400 -2.89760000

C 18.71534400 2.90001100 -2.38618600

C 19.95755900 2.41548400 -2.93089000

C -11.86400000 -2.75355000 -2.37464600

C -10.66500400 -2.21049700 -2.92347000

C -9.40139800 -2.69602900 -2.46806000

C -8.19866700 -2.13197500 -2.99382200

C -8.07180300 -3.60905800 -0.60890500

C -6.87825600 -3.41376000 -1.35910900

C -5.61639600 -3.62556600 -0.72847300

C -4.41927100 -3.40030500 -1.46796700

C -4.25461700 -3.50330900 1.31593000

C -3.07908400 -3.75199200 0.56627200

C -1.78693900 -3.57938800 1.16505300

C -0.59620000 -3.89543000 0.44403400

C -0.43432900 -2.32850700 2.78144300

C 0.74690800 -2.91362700 2.24158800

C 2.01883700 -2.44528700 2.70974500

C 3.23696600 -2.98726300 2.16436900

C 3.37443100 -0.59673700 3.63813700

C 4.56985400 -1.34664000 3.44762900

C 5.83998300 -0.73164100 3.67560800

C 7.03742100 -1.47866600 3.45215300

C 7.19467200 1.32284100 3.59054600

C 8.38588700 0.55650000 3.77410000

C 9.65965400 1.19291200 3.64876100

C 10.85090600 0.42279000 3.80608400

C 11.01412800 2.92027000 2.53408700

C 12.20509200 2.34180700 3.06967400

C 13.47869000 2.82428100 2.63492200

C 14.66688600 2.23057400 3.14807700

C 14.82668900 3.74507500 0.79532700

C 16.02072800 3.50113900 1.54114300

C 17.29943700 3.70056400 0.92773700

C 18.48529900 3.49131100 1.68367500

C 18.64333600 3.61391700 -1.14298300

C 19.79994400 3.86582800 -0.33330200

C -6.93714800 -2.63744800 -2.55700800

C -5.73535200 -2.04943600 -3.04983200

C -4.47173200 -2.56935600 -2.62060100

C -3.16070100 -3.67449400 -0.86303400

C -1.97500300 -3.42506700 -1.58903500

C -0.68606200 -4.08050900 -1.11033900

C 0.58040300 -3.45692600 -1.69857000

C 0.64055400 -3.72018400 1.06671200

C 1.90157300 -4.23401500 0.36995800

C 3.19695400 -3.69526100 0.96303000

C 4.38036100 -3.81472000 0.20119900

C 4.50174600 -2.54223900 2.67961600

C 5.70199200 -3.07207000 2.13371100

C 6.96996600 -2.65280600 2.64976400

C 8.16626900 -3.14900800 2.05305600

C 8.30849000 -0.86464200 3.68248700

C 9.50387800 -1.60219600 3.42510700

C 10.77403100 -0.99535100 3.66904900

C 11.96904800 -1.72245100 3.37879900

C 12.12424000 1.06422200 3.69536200

C 13.31779000 0.28990700 3.82813900

C 14.58663800 0.93240700 3.73347600

C 15.78024300 0.15247100 3.82367900

C 15.94077200 2.71723800 2.71876400

C 17.14021500 2.11885300 3.22137100

C 18.40616800 2.65288800 2.85213100

C 19.55866400 2.01912800 3.41755300

C 19.72442700 3.79790000 1.03250500

C -2.00669200 -2.50378400 -2.63545200

C 0.48351400 -2.44215900 -2.70030000

C 1.81456400 -3.75876700 -1.12163900

C 3.00918000 -3.32489400 -1.77122500

C 4.29862600 -3.62044900 -1.21692700

C 5.47731500 -3.24639600 -1.90835700

C 5.64199600 -3.67161700 0.84529000

C 6.83655800 -3.76599400 0.07495100

C 8.10010400 -3.68659000 0.73237000

C 9.29301800 -3.75063900 -0.04363400

C 9.43371300 -2.74668500 2.57478400

C 10.62911800 -3.21438200 1.95264700

C 11.89703600 -2.83120700 2.48689500

C 13.09383800 -3.27648000 1.84557100

C 13.24102900 -1.12437700 3.64242200

C 14.43216900 -1.83712400 3.32368600

C 15.70334300 -1.24344400 3.59289500

C 16.90616900 -1.94751600 3.26028900

C 17.05978000 0.79457500 3.76323600

C 18.24563900 0.02143600 3.89066400

C 19.48178000 0.74191100 3.91126600

C 2.93635300 -2.33334200 -2.78973800

C 5.40038600 -2.24949400 -2.93570400

C 6.75770600 -3.52437900 -1.32593500

C 7.94644900 -3.15319200 -2.01794100

C 9.21716400 -3.45498200 -1.43831800

C 10.40908400 -3.07089300 -2.11951900

C 10.55981700 -3.70047200 0.61200300

C 11.75230500 -3.73748200 -0.16805200

C 13.02263800 -3.71277500 0.48742900

C 14.21304900 -3.72583000 -0.29088200

C 14.35899200 -2.91433300 2.38995000

C 15.55382000 -3.32237700 1.72415700

C 16.83165700 -2.99286600 2.28289800

C 18.01910400 -3.43759600 1.64166700

C 18.16964700 -1.39116400 3.60155600

C 19.32295100 -2.16388700 3.25991900

C 7.86837700 -2.14872300 -3.03082100

C 11.67801200 -3.39584200 -1.55000300

C 12.87259200 -2.99472400 -2.22122400

C 14.14058300 -3.34026900 -1.66583600

C 15.33178000 -2.91254000 -2.31579400

C 15.48084700 -3.71030800 0.36305900

C 16.68484800 -3.71153700 -0.41346800

C 17.94595200 -3.78587800 0.23889300

C 19.09629900 -3.82263000 -0.60415500

C 19.25110000 -3.14801800 2.30461600

C 16.61282100 -3.28000900 -1.77847700

C 17.80180600 -2.90149800 -2.45339200

C 19.02581200 -3.38759500 -1.90733000

H -21.51704700 -0.99938600 -3.80673700

H -20.39429200 1.27494100 -3.81709300

H -20.45670500 3.23699400 -2.92069700

H -20.68589900 4.08330700 1.44341100

H -20.57707000 4.27559800 -0.74193500

H -20.82653800 2.55725700 3.31368800

H -20.93770900 0.52341800 4.12274400

H -21.19308800 -3.39449900 2.00278500

H -21.08275000 -1.81866600 3.53174700

H -21.32695400 -3.80141200 -0.37992700

H 19.87103100 -1.46109300 -3.61319300

H -21.42855800 -2.99735500 -2.40728600

H 20.97853300 0.80481900 -3.88131000

H 20.87817900 2.95280200 -2.72561100

H 20.77329500 3.97630600 -0.80051500

H 20.53325900 2.48827300 3.32748500

H 20.63961000 3.85679600 1.61345600

H 20.39732400 0.23554300 4.20101500

H 20.29717200 -1.88110500 3.64584200

H 20.07013600 -4.05326200 -0.18388800

H 20.17030500 -3.61560500 1.96550800

H 19.94693200 -3.29120600 -2.47382000

C 1.99734200 -5.81973000 0.59203100

C -0.79891300 -5.59415500 -1.63319500

C -1.44015500 -5.83494200 -3.04414500

C -1.16389400 -7.26050200 -3.62529200

C -2.11336300 -7.65631900 -4.79150700

C 2.63271700 -6.33418000 1.93043900

C 2.33544000 -7.83992100 2.22893200

C 3.27451200 -8.46483100 3.29935600

F 0.43437400 -6.15882600 -1.76680100

F -1.52888500 -6.31731100 -0.74721200

F -0.92731400 -4.94439500 -3.93091700

F -2.78725300 -5.68178100 -2.99107300

F 0.09726200 -7.30672500 -4.10727200

F -1.30852200 -8.19373100 -2.65625300

F -1.63812600 -8.76165000 -5.38030100

F -3.34632500 -7.91098200 -4.34038600

F -2.17304200 -6.67532000 -5.70390700

F 0.75684500 -6.38343300 0.61411100

F 2.72058300 -6.36714400 -0.41700300

F 2.13059200 -5.62139500 2.97049300

F 3.98181600 -6.19252000 1.90671400

F 2.47213300 -8.57260200 1.09946200

F 1.07167500 -7.96000500 2.69095300

F 2.78007100 -9.65446300 3.66619400

F 4.50500800 -8.64829400 2.80861800

F 3.34620300 -7.67658800 4.38200100

**(6,5) SWNT-[(CH_2_)_3_CH_3_]_2_ (1,2-L_++_: L_87_)**

C -20.55325700 -1.78980500 -3.69706900

C -19.36763200 -1.00543700 -3.92379000

C -19.41143400 0.39010000 -4.04854600

C -18.25598900 1.16792100 -3.83133500

C -18.11033600 -1.64210900 -3.68977800

C -16.91640700 -0.90639400 -3.91794900

C -15.64724400 -1.55054300 -3.74204100

C -14.44861600 -0.81564000 -3.96077200

C -19.52934000 2.98347100 -2.61034800

C -18.30870800 2.42288700 -3.08907700

C -17.11751000 2.87396100 -2.46487300

C -16.98432200 0.51502600 -3.94879000

C -15.77444600 1.24814200 -3.72908400

C -14.51087100 0.61199000 -3.92667800

C -13.31200500 1.34002000 -3.67769900

C -13.18559300 -1.46185800 -3.79772200

C -11.98186000 -0.71187700 -3.97491300

C -10.71835300 -1.36392500 -3.83521000

C -9.51696100 -0.60802000 -3.97802900

C -19.76103600 3.25109000 1.59498900

C -19.59773800 3.57776800 -1.37155300

C -18.44861800 3.63488400 -0.52774000

C -17.18761100 3.47248000 -1.16414900

C -15.98433900 3.55835700 -0.39085500

C -15.83854700 2.43216100 -2.94929800

C -14.64444400 2.92907900 -2.35554300

C -13.37872100 2.50624900 -2.85949600

C -12.18091600 2.97847500 -2.23999800

C -12.04402400 0.70935300 -3.88820000

C -10.84533300 1.43279200 -3.61572000

C -9.57981500 0.81059900 -3.84299300

C -8.38035900 1.52581900 -3.54297200

C -8.25091800 -1.26391900 -3.85494900

C -7.04938900 -0.50256000 -3.96783300

C -5.78528000 -1.16078200 -3.85687100

C -4.58293200 -0.39443000 -3.93416700

C -3.25860600 -2.37455500 -3.29906700

C -0.79032900 -2.28588700 -3.32628800

C -19.83960500 2.38642600 2.65909700

C -18.69047200 1.65500300 3.09313500

C -18.52606000 3.45619700 0.90646400

C -17.34225600 3.08451600 1.59856000

C -16.06178800 3.33878800 1.00705200

C -14.87029500 3.00813500 1.72003100

C -14.71546900 3.48474900 -1.03963800

C -13.52632700 3.56059400 -0.26311900

C -12.25411000 3.49293300 -0.91352400

C -11.06221800 3.54837300 -0.13179200

C -10.91347400 2.57407500 -2.75962800

C -9.71980600 3.02825900 -2.12680400

C -8.45026100 2.63932600 -2.65571400

C -7.25642200 3.08067700 -2.01067200

C -7.11336200 0.91270400 -3.78908800

C -5.91743300 1.62033700 -3.46745500

C -4.64872200 1.01329500 -3.72374600

C -3.45329400 1.71587200 -3.38631400

C -3.31814200 -1.05301300 -3.82881400

C -2.12111300 -0.28805600 -3.88899800

C -0.85597300 -0.95454300 -3.80160000

C 0.34158100 -0.17263200 -3.87843600

C 1.67695400 -2.17677900 -3.40790600

C 4.14085200 -2.07944300 -3.53569300

C -20.01192200 -0.41956000 3.65923900

C -18.77301200 0.28789500 3.55017300

C -17.59046700 -0.50005400 3.51914300

C -17.42390900 2.16127700 2.69151000

C -16.22485700 1.49658500 3.10826400

C -14.95066900 2.04907200 2.77479500

C -13.76397000 1.37409200 3.17877500

C -13.60300000 3.29565000 1.13846300

C -12.40989700 2.92634000 1.83309700

C -11.13884100 3.23609200 1.26004000

C -9.94896700 2.84501500 1.94032200

C -9.79392800 3.50340000 -0.78332100

C -8.60077600 3.53230000 0.00001900

C -7.33090000 3.51443100 -0.65296000

C -6.13916600 3.52157900 0.13016900

C -5.98808500 2.71090000 -2.55055400

C -4.79404900 3.13475200 -1.89147400

C -3.52385700 2.78418800 -2.44304500

C -2.33062600 3.18863100 -1.77361200

C -2.18705100 1.11516300 -3.65517500

C -0.99165900 1.81142400 -3.30384000

C 0.27492700 1.21995500 -3.60238400

C 1.47290900 1.90698600 -3.24330600

C 1.60540800 -0.82707900 -3.85652700

C 2.80178500 -0.05170600 -3.91407500

C 4.06874100 -0.71097100 -3.92219500

C 5.26698000 0.06682800 -3.93728100

C 6.60314200 -1.97575900 -3.62063000

C 9.06891100 -1.86746200 -3.68683400

C -20.26786100 -3.81212400 1.18489600

C -20.09398300 -1.74800900 3.32789800

C -18.94423900 -2.45217900 2.84654300

C -17.67615700 -1.88095600 3.14570400

C -16.47909200 -2.54215800 2.72178700

C -16.30820600 0.13866900 3.50326000

C -15.11778400 -0.65148400 3.51005800

C -13.84683800 -0.00828000 3.52989500

C -12.65616700 -0.79776000 3.49125700

C -12.48943600 1.93218200 2.85025800

C -11.29822600 1.24003800 3.22903900

C -10.02754900 1.81103900 2.92216700

C -8.83565800 1.10390800 3.26852000

C -8.67754100 3.17299200 1.37717000

C -7.48502800 2.75984200 2.04425000

C -6.21570700 3.11255400 1.49512900

C -5.02241300 2.67287000 2.14372700

C -4.86851100 3.52565400 -0.52295900

C -3.67429700 3.50843300 0.26057000

C -2.40410200 3.53561700 -0.39191300

C -1.20888900 3.48330300 0.38914200

C -1.06060400 2.84951300 -2.33505700

C 0.13794100 3.22965200 -1.65376700

C 1.40552000 2.91092400 -2.23147900

C 2.60284600 3.25876400 -1.53792800

C 2.73624700 1.33047800 -3.57155500

C 3.93683300 1.99638700 -3.17837100

C 5.20128200 1.43348500 -3.53150100

C 6.40030500 2.08054400 -3.10834000

C 6.53243200 -0.59217000 -3.96461400

C 7.73144300 0.18446300 -3.94068500

C 8.99887900 -0.47377800 -3.98814800

C 10.19656200 0.30009400 -3.93479200

C 10.33758600 -2.45026400 -3.38564600

C 11.53257000 -1.75811700 -3.74381600

C 12.80311300 -2.35222600 -3.45884500

C 13.99808700 -1.65329400 -3.79521200

C -20.34508400 -4.04484200 -0.16255400

C -19.18837100 -3.89488800 -0.99744700

C -19.02707300 -3.43084300 1.79277200

C -17.84281500 -3.73628900 1.06793700

C -16.56274400 -3.46663500 1.65135000

C -15.37018100 -3.80532800 0.94062900

C -15.20301500 -2.01128600 3.08745200

C -14.01729000 -2.66768400 2.65107900

C -12.74151100 -2.14025300 3.02274500

C -11.55207000 -2.78316200 2.56079400

C -11.38055300 -0.15332600 3.52944300

C -10.19249000 -0.94012000 3.46368400

C -8.91757000 -0.29740000 3.51666700

C -7.72665400 -1.08277000 3.42075000

C -7.56309400 1.68847900 2.98390600

C -6.37231500 0.97045400 3.30228100

C -5.10094500 1.56562200 3.03753200

C -3.90795300 0.83368100 3.32330300

C -3.75099500 3.04890700 1.60885500

C -2.55825700 2.58485000 2.23906600

C -1.28665200 2.97533500 1.71694500

C -0.09272000 2.48113100 2.32971400

C 0.06461200 3.52745800 -0.26327700

C 1.25967900 3.44551900 0.51549700

C 2.53026000 3.50616000 -0.13469800

C 3.72398100 3.38955700 0.64099500

C 3.87017200 2.95544800 -2.12590600

C 5.06773300 3.27607000 -1.41808900

C 6.33374700 2.99701900 -2.01646300

C 7.53006100 3.28968700 -1.29575400

C 7.66557700 1.53220600 -3.48083000

C 8.86524200 2.16284700 -3.02994200

C 10.13072500 1.63179500 -3.42351000

C 11.32830100 2.24288000 -2.94542900

C 11.46312900 -0.35735800 -3.99938600

C 12.66266500 0.41470000 -3.91478100

C 13.92854800 -0.24046600 -4.00178000

C 15.12368500 0.52168500 -3.87568400

C 15.26467500 -2.25445300 -3.52191500

C 16.47041400 -1.54782600 -3.83180500

C 17.74527100 -2.17496200 -3.63595500

C 18.89641900 -1.42452700 -3.95108700

C -20.49901700 -2.90483500 -2.91057100

C -19.25851600 -3.33060900 -2.31530900

C -18.05828900 -2.82709000 -2.89193200

C -17.92188000 -3.95702500 -0.34629000

C -16.71964500 -3.85477200 -1.11409300

C -15.44769600 -3.97934300 -0.47157300

C -14.25623200 -3.86253300 -1.24324000

C -14.10097800 -3.56517100 1.54275100

C -12.91019600 -3.86788000 0.81601800

C -11.63679400 -3.64377100 1.42595900

C -10.44849100 -3.92855700 0.69217000

C -10.27805000 -2.27059300 2.95031500

C -9.09028600 -2.89661000 2.46569400

C -7.81269000 -2.39547200 2.86748300

C -6.62650000 -3.01024400 2.36618000

C -6.45383400 -0.44202400 3.49480000

C -5.26426100 -1.22202200 3.35973200

C -3.98922300 -0.58372400 3.45134000

C -2.80150500 -1.35492400 3.27505200

C -2.63781100 1.43874900 3.08520900

C -1.44758700 0.69174000 3.33355300

C -0.17399800 1.30725200 3.12706800

C 1.01359900 0.55263800 3.36610800

C 1.18139700 2.88978600 1.82678400

C 2.37001100 2.37360000 2.42415300

C 3.64441900 2.79226600 1.93290200

C 4.83340000 2.25828000 2.51695400

C 4.99499300 3.47170400 -0.00678000

C 6.18670300 3.33106800 0.76495600

C 7.45681400 3.43501000 0.12080900

C 8.64911800 3.27112400 0.88873000

C 8.79769700 3.03491500 -1.90348800

C 9.99301000 3.30450900 -1.17291700

C 11.25988100 3.07246300 -1.78804600

C 12.45607600 3.31660000 -1.04610300

C 12.59653500 1.72469800 -3.35557400

C 13.79343000 2.31906200 -2.85617400

C 15.05892200 1.81014000 -3.27557000

C 16.25789000 2.38411300 -2.74807900

C 16.39542900 -0.13341100 -3.97555200

C 17.58597800 0.63036000 -3.84088300

C 18.84583900 -0.02451500 -3.99894100

C 20.02798700 0.78720900 -3.87235100

C -16.78781500 -3.25952600 -2.39816500

C -15.58733700 -2.75823300 -2.99225500

C -14.32341600 -3.22050200 -2.51781700

C -13.12493300 -2.69603000 -3.08652500

C -12.98721000 -3.99608700 -0.60092600

C -11.79172600 -3.85115300 -1.36922500

C -10.52510200 -4.01163000 -0.73182900

C -9.33103800 -3.84219300 -1.49219600

C -9.17638500 -3.72344900 1.30712200

C -7.98687500 -3.99054700 0.56409700

C -6.71392500 -3.81189600 1.18493800

C -5.52986300 -4.06582300 0.43585700

C -5.35134800 -2.51378000 2.76712500

C -4.16221700 -3.11053800 2.23452400

C -2.88760500 -2.61962000 2.62508400

C -1.70323700 -3.21134400 2.07113500

C -1.52994800 -0.72569400 3.39460600

C -0.34087500 -1.49991200 3.19762500

C 0.93188000 -0.86869700 3.38325900

C 2.12074200 -1.62835400 3.20555100

C 2.28692600 1.17327500 3.19090300

C 3.47455800 0.41491400 3.41544900

C 4.75006000 1.04005000 3.25385000

C 5.93873200 0.27862700 3.46177300

C 6.10680900 2.69363300 2.03956900

C 7.29535100 2.14558800 2.60781100

C 8.56925900 2.59618700 2.14231300

C 9.75915000 2.03613700 2.69646900

C 9.91940300 3.40108500 0.24958100

C 11.11025900 3.21467000 1.01125700

C 12.38176600 3.36499800 0.37581500

C 13.57468900 3.15791200 1.13214000

C 13.72487800 3.11024700 -1.66899000

C 14.91735200 3.32490700 -0.91971400

C 16.18893000 3.12845700 -1.54425200

C 17.39197900 3.32652400 -0.79675700

C 17.52951600 1.90223400 -3.19052100

C 18.72840000 2.47576400 -2.68033300

C 19.96972900 1.98818300 -3.22466300

C -11.85797100 -3.16905400 -2.61989600

C -10.65869800 -2.62705200 -3.17000900

C -9.39517600 -3.11507100 -2.71772500

C -8.19214200 -2.55021700 -3.24188200

C -8.06350200 -4.02812200 -0.85928800

C -6.86811800 -3.84105200 -1.61318600

C -5.60626400 -4.05959100 -0.98787900

C -4.40428600 -3.83999700 -1.72676200

C -4.25068900 -3.89929500 1.05314700

C -3.06191700 -4.18943100 0.31839400

C -1.80054300 -4.03480900 0.93414900

C -0.58244700 -4.65968300 0.27937900

C -0.42092900 -2.74285100 2.52494800

C 0.78001000 -3.32040700 1.99183800

C 2.04666300 -2.85378400 2.48153700

C 3.24484100 -3.41021000 1.95860500

C 3.39292300 -1.00764200 3.40798100

C 4.58682400 -1.76554300 3.21272200

C 5.85871100 -1.14611100 3.42083400

C 7.05173900 -1.89405800 3.19202600

C 7.21252500 0.90819300 3.31252000

C 8.40365000 0.14180000 3.49619500

C 9.67750900 0.77726400 3.36579400

C 10.86752800 0.00731500 3.52393500

C 11.03071800 2.50135800 2.24426700

C 12.22210600 1.92311700 2.77955800

C 13.49560500 2.40463000 2.34328400

C 14.68369300 1.81075500 2.85591100

C 14.84244200 3.32529200 0.50288900

C 16.03708700 3.08025500 1.24776800

C 17.31558800 3.27932700 0.63363700

C 18.50183300 3.06893100 1.38851200

C 18.65794600 3.19108500 -1.43799300

C 19.81546500 3.44251300 -0.62926600

C -6.92957100 -3.05932600 -2.80895300

C -5.72788600 -2.46859500 -3.29425400

C -4.46338900 -2.99094400 -2.86670300

C -3.13818500 -4.13637400 -1.13275700

C -1.95220800 -3.88189300 -1.85206400

C -0.65363800 -4.52734000 -1.40337700

C 0.57227500 -3.82275000 -1.95399900

C 0.72358400 -4.10713300 0.82183900

C 1.90733000 -4.25948900 0.07027200

C 3.17676900 -4.07316700 0.70117000

C 4.37610500 -4.18981700 -0.06462000

C 4.51489600 -2.97170300 2.45787900

C 5.71039000 -3.49487700 1.88636200

C 6.97846100 -3.07143400 2.38938900

C 8.17444600 -3.56288100 1.78358000

C 8.32451000 -1.27907200 3.41148000

C 9.51849300 -2.01826700 3.15308500

C 10.78875700 -1.41174200 3.39130600

C 11.98252100 -2.14011700 3.09882000

C 12.14123000 0.64721300 3.40811400

C 13.33417500 -0.12783000 3.54125100

C 14.60314200 0.51320700 3.44303400

C 15.79610400 -0.26786000 3.53253400

C 15.95766600 2.29608400 2.42511400

C 17.15713500 1.69688700 2.92691400

C 18.42315000 2.22982500 2.55649200

C 19.57575600 1.59484100 3.12039300

C 19.74082200 3.37480000 0.73649600

C -1.99819400 -2.92423000 -2.88692200

C 0.48569000 -2.83287400 -2.95039800

C 1.83124500 -4.08611900 -1.37141600

C 3.02368800 -3.70070400 -2.05479600

C 4.30020200 -3.97556600 -1.47250500

C 5.48732700 -3.62273500 -2.17570300

C 5.64042500 -4.08364000 0.58535900

C 6.83380300 -4.16711500 -0.19025900

C 8.10235500 -4.09373900 0.46188500

C 9.29528700 -4.15770600 -0.31774900

C 9.44409100 -3.16323900 2.30256000

C 10.63690100 -3.63082800 1.67688200

C 11.90693700 -3.24911200 2.20802600

C 13.10203900 -3.69578400 1.56417200

C 13.25557900 -1.54265500 3.35873500

C 14.44495800 -2.25701000 3.03881600

C 15.71727900 -1.66399900 3.30427400

C 16.91869800 -2.36992100 2.97062500

C 17.07620700 0.37285200 3.46949900

C 18.26136100 -0.40142200 3.59605300

C 19.49828900 0.31774000 3.61432100

C 2.94516500 -2.73752900 -3.10013800

C 5.40979400 -2.64704200 -3.21848900

C 6.75804800 -3.91008700 -1.59182300

C 7.94981100 -3.54534200 -2.28665200

C 9.21982800 -3.85610000 -1.71152400

C 10.41150100 -3.47718100 -2.39548400

C 10.56330700 -4.11307800 0.33455900

C 11.75546100 -4.15014400 -0.44783500

C 13.02739700 -4.13007900 0.20541000

C 14.21666300 -4.14463800 -0.57470700

C 14.36848800 -3.33517500 2.10609100

C 15.56170800 -3.74464000 1.43819400

C 16.84102700 -3.41650900 1.99470300

C 18.02669800 -3.86343300 1.35198700

C 18.18333500 -1.81438400 3.30869400

C 19.33513300 -2.58887800 2.96595600

C 7.87392200 -2.54831600 -3.30405100

C 11.68053200 -3.80586500 -1.82857100

C 12.87566900 -3.40830400 -2.50233700

C 14.14331200 -3.75727200 -1.94931800

C 15.33490500 -3.33198000 -2.60090600

C 15.48574700 -4.13202100 0.07720700

C 16.68882700 -4.13534000 -0.70109400

C 17.95067300 -4.21234700 -0.05054800

C 19.09984100 -4.25138000 -0.89515000

C 19.26029800 -3.57435400 2.01225600

C 16.61593500 -3.70273600 -2.06575800

C 17.80486900 -3.32650000 -2.74211600

C 19.02854800 -3.81558500 -2.19798100

H -21.51191700 -1.41087200 -4.04249500

H -20.38866600 0.86304700 -4.05626100

H -20.45047900 2.82581800 -3.16297400

H -20.67750200 3.67852300 1.19999400

H -20.56990500 3.86818500 -0.98589800

H -20.81614800 2.15464500 3.07225100

H -20.92543600 0.12193900 3.88460000

H -21.18253000 -3.79790300 1.76953400

H -21.07045100 -2.22089800 3.29651200

H -21.31914400 -4.20878100 -0.61232500

H 19.87627500 -1.88946300 -3.90218000

H -21.42269900 -3.40730800 -2.64090400

H 20.98777400 0.37439200 -4.17287100

H 20.89130300 2.52431400 -3.02046900

H 20.78849900 3.55236900 -1.09732700

H 20.55072200 2.06294400 3.02882100

H 20.65637500 3.43337900 1.31692900

H 20.41377400 -0.18951500 3.90277600

H 20.31046000 -2.30646700 3.34940000

H 20.07374100 -4.48416600 -0.47617700

H 20.17838800 -4.04323000 1.67185500

H 19.94909500 -3.72089600 -2.76572000

C -0.60806200 -6.18195700 0.72242900

H -1.52715800 -6.63445500 0.33614900

H 0.23732100 -6.69317100 0.25193200

C -0.64514800 -5.96212600 -2.07862300

H -1.49882900 -6.52959600 -1.69573900

H 0.26718800 -6.48002400 -1.76579200

C -0.70494500 -5.99839700 -3.61339200

C -0.66365700 -7.43864000 -4.14672800

H 0.13404900 -5.43598400 -4.04089800

H -1.62070100 -5.51217300 -3.97080500

C -0.72513300 -7.50761300 -5.67606600

H 0.25329000 -7.93233600 -3.79472200

H -1.50030700 -8.01063700 -3.72085600

H -0.69383100 -8.54424700 -6.02998000

H -1.64781700 -7.05267600 -6.05633500

H 0.11829700 -6.97394900 -6.13079600

C -0.54499800 -6.46098300 2.23225700

C -0.60628400 -7.96686400 2.52992700

H -1.37395700 -5.96143800 2.74836200

H 0.37939300 -6.05105600 2.65694000

C -0.53331200 -8.28091900 4.02767200

H -1.53437300 -8.38381100 2.11368700

H 0.21703500 -8.47612000 2.00902400

H -0.58135200 -9.36024000 4.21106500

H 0.40110900 -7.90936300 4.46555300

H -1.36265800 -7.81283800 4.57173900

**(6,5) SWNT-[(CH_2_)_3_CH_3_]_2_ (1,2-L_+_: L_27_)**

C -20.54123400 -1.56179300 -3.52101900

C -19.35437300 -0.78013700 -3.74982700

C -19.39571900 0.61540700 -3.87636600

C -18.23921700 1.39149800 -3.65912600

C -18.09812500 -1.41847100 -3.51547300

C -16.90280000 -0.68470900 -3.74474600

C -15.63500800 -1.33066400 -3.56942400

C -14.43480200 -0.59721700 -3.78728300

C -19.51118900 3.20655400 -2.43658400

C -18.29090300 2.64582800 -2.91586500

C -17.09943800 3.09442700 -2.29031600

C -16.96818400 0.73694900 -3.77600000

C -15.75730900 1.46813000 -3.55545700

C -14.49432200 0.83014100 -3.75175300

C -13.29351500 1.55586000 -3.50100600

C -13.17278500 -1.24591800 -3.62614400

C -11.96739100 -0.49822300 -3.80094300

C -10.70561200 -1.15377400 -3.66275100

C -9.49918600 -0.40084600 -3.80074200

C -19.74388000 3.46197800 1.77055800

C -19.57999800 3.79724600 -1.19601100

C -18.43140900 3.85113600 -0.35153300

C -17.17012900 3.69012300 -0.98826500

C -15.96727700 3.77495800 -0.21492900

C -15.82061200 2.65197700 -2.77473400

C -14.62641300 3.14672400 -2.17970600

C -13.35999800 2.72247000 -2.68239300

C -12.16279600 3.19390800 -2.06246300

C -12.02657100 0.92353300 -3.70996900

C -10.82586100 1.64315800 -3.43206100

C -9.55997000 1.01914500 -3.65648600

C -8.36082100 1.72958100 -3.34974600

C -8.23815100 -1.06091500 -3.68234300

C -7.02988400 -0.30190300 -3.77302700

C -5.76768900 -0.96324600 -3.66375900

C -4.56748900 -0.20253100 -3.70661500

C -3.23601600 -2.20462800 -3.11205400

C -0.80335700 -2.14081700 -3.15246300

C -19.82162000 2.59711200 2.83472300

C -18.67198500 1.86676400 3.26906800

C -18.50918100 3.66897500 1.08227600

C -17.32514300 3.29701400 1.77391900

C -16.04503000 3.55334200 1.18285600

C -14.85385200 3.22261700 1.89542400

C -14.69842500 3.70207400 -0.86380800

C -13.50969500 3.77881000 -0.08701400

C -12.23745100 3.71208400 -0.73691100

C -11.04690100 3.77060900 0.04551500

C -10.89453200 2.78567600 -2.57818000

C -9.70124000 3.24250400 -1.94566700

C -8.43146000 2.84949400 -2.46867200

C -7.23912700 3.29610900 -1.82393700

C -7.09320100 1.11157100 -3.58557100

C -5.89725600 1.81914900 -3.26197700

C -4.63158800 1.20792200 -3.50429600

C -3.43674800 1.91095300 -3.16820500

C -3.30352500 -0.86509200 -3.58402000

C -2.10668600 -0.10489600 -3.65176500

C -0.85501500 -0.77203100 -3.58539500

C 0.34032800 -0.00300800 -3.65622300

C 1.68416600 -2.03617100 -3.29660500

C 4.15640300 -1.90614200 -3.42682200

C -19.99200900 -0.20699000 3.84081200

C -18.75345000 0.50027600 3.72809800

C -17.57086700 -0.28791900 3.69538000

C -17.40584500 2.37315300 2.86635800

C -16.20635000 1.70863300 3.28233200

C -14.93308500 2.26177900 2.94845300

C -13.74563400 1.58608800 3.35000700

C -13.58682900 3.51290100 1.31443800

C -12.39422600 3.14499000 2.00908000

C -11.12429600 3.45883400 1.43714400

C -9.93429400 3.06989600 2.11805500

C -9.77741400 3.72632100 -0.60466400

C -8.58638800 3.76306700 0.17953800

C -7.31570900 3.74335700 -0.47121800

C -6.12451900 3.76092400 0.31415700

C -5.96883800 2.92208900 -2.35661100

C -4.77790300 3.35388500 -1.70107400

C -3.50729800 2.99225800 -2.24290700

C -2.31310100 3.40536300 -1.57905900

C -2.17226700 1.30759200 -3.43196200

C -0.98113000 1.99570200 -3.09025600

C 0.27950200 1.38981500 -3.37790500

C 1.47941800 2.07633900 -3.02954200

C 1.60924900 -0.66458500 -3.67499200

C 2.80104600 0.11775500 -3.73291300

C 4.07565100 -0.52954800 -3.77316700

C 5.27267800 0.25604600 -3.77795000

C 6.62141600 -1.78203800 -3.49259000

C 9.08687000 -1.66044000 -3.54441200

C -20.25408500 -3.59288600 1.36202300

C -20.07482500 -1.53550300 3.50988500

C -18.92631700 -2.23928500 3.02521300

C -17.65733200 -1.66869600 3.32223300

C -16.46103200 -2.32998200 2.89566800

C -16.28852100 0.35042300 3.67720700

C -15.09807700 -0.43984800 3.68112700

C -13.82681700 0.20329300 3.69821300

C -12.63533300 -0.58574500 3.65537900

C -12.47216500 2.14679500 3.02316300

C -11.28084300 1.45395400 3.39713500

C -10.01094700 2.02982900 3.09334900

C -8.81839000 1.32389700 3.43435900

C -8.66399300 3.40571500 1.55834800

C -7.47255800 2.99544100 2.22589900

C -6.20222900 3.35650500 1.68084700

C -5.01009000 2.92454800 2.33272900

C -4.85323900 3.76319500 -0.33562800

C -3.66059300 3.75278000 0.44945400

C -2.38703700 3.77254000 -0.20078100

C -1.19373300 3.73084700 0.57957000

C -1.04690800 3.05619800 -2.13444400

C 0.14907600 3.43375700 -1.46007000

C 1.41525900 3.09470400 -2.03364200

C 2.61256300 3.45254400 -1.35128300

C 2.73828200 1.49878300 -3.36837300

C 3.93761600 2.16843200 -2.98545300

C 5.20471600 1.61468200 -3.35066200

C 6.40304500 2.26815900 -2.93445600

C 6.54324500 -0.39465000 -3.82182500

C 7.73946700 0.38618000 -3.78823000

C 9.01146400 -0.26681100 -3.84036500

C 10.20698000 0.51011400 -3.78257000

C 10.35535600 -2.23993300 -3.23875900

C 11.54880000 -1.54459400 -3.59298400

C 12.81972100 -2.13622200 -3.30394300

C 14.01358700 -1.43573000 -3.63868500

C -20.33376300 -3.82110800 0.01382700

C -19.17811700 -3.67099100 -0.82224000

C -19.01195000 -3.21536100 1.96931100

C -17.82908700 -3.52003400 1.24186200

C -16.54763700 -3.25319100 1.82412500

C -15.35682500 -3.59204200 1.11127200

C -15.18395500 -1.79955800 3.25851000

C -13.99861700 -2.45584100 2.81881100

C -12.72085200 -1.92861400 3.18663800

C -11.53241500 -2.57088600 2.72110200

C -11.36063200 0.05930400 3.69038900

C -10.17070200 -0.72625900 3.61507800

C -8.89608600 -0.08159900 3.66830300

C -7.70543100 -0.86176800 3.55733900

C -7.54911000 1.91605700 3.15589000

C -6.35609600 1.19882200 3.46683700

C -5.08766000 1.80755800 3.21874600

C -3.89631700 1.08036300 3.50114300

C -3.73873200 3.30312300 1.80072400

C -2.54504600 2.84467200 2.43688200

C -1.27137400 3.23640500 1.91930600

C -0.08062500 2.74891100 2.53463500

C 0.07751700 3.75824500 -0.07299100

C 1.27347200 3.68070400 0.70404700

C 2.54292100 3.72753000 0.04877700

C 3.73643900 3.62019100 0.82017700

C 3.87497900 3.13733900 -1.94022000

C 5.07456800 3.46885300 -1.24221300

C 6.33864600 3.18786100 -1.84393300

C 7.53566500 3.49014300 -1.13066900

C 7.67008700 1.72910300 -3.31552000

C 8.87002000 2.36351900 -2.86642600

C 10.13763000 1.83960400 -3.26521900

C 11.33456100 2.45292900 -2.78762700

C 11.47571200 -0.14409800 -3.84747700

C 12.67409200 0.63004500 -3.76029600

C 13.94140400 -0.02308500 -3.84661900

C 15.13525000 0.74057300 -3.71954800

C 15.28025500 -2.03467800 -3.36156000

C 16.48544600 -1.32701500 -3.67063900

C 17.76066600 -1.95226200 -3.47145400

C 18.91112100 -1.20110600 -3.78724500

C -20.48869400 -2.67624500 -2.73330300

C -19.24871500 -3.10459500 -2.13936000

C -18.04802100 -2.60301300 -2.71711800

C -17.91091700 -3.73676400 -0.17268300

C -16.70957200 -3.63563700 -0.94183600

C -15.43762400 -3.76391000 -0.30107700

C -14.24736900 -3.64844600 -1.07456800

C -14.08584500 -3.35314300 1.71130000

C -12.89649200 -3.65834900 0.98272300

C -11.62151100 -3.43472900 1.58899300

C -10.43512300 -3.72299700 0.85188300

C -10.25564600 -2.05498500 3.10176000

C -9.06672000 -2.68044700 2.61264400

C -7.78876700 -2.17165600 2.99938800

C -6.60031900 -2.77716400 2.48049700

C -6.43223300 -0.21643000 3.63397700

C -5.24047800 -0.98651700 3.48544200

C -3.97422600 -0.34010800 3.59793900

C -2.78253000 -1.10025700 3.41270800

C -2.62474800 1.69763400 3.28055000

C -1.43438200 0.96065600 3.54298300

C -0.16281000 1.57443400 3.34332600

C 1.02533600 0.81755700 3.57423500

C 1.19512400 3.14487300 2.02324500

C 2.38554800 2.63090800 2.62189200

C 3.65843900 3.04009600 2.12199900

C 4.84757900 2.50370300 2.70196400

C 5.00540100 3.68706800 0.16551600

C 6.19882800 3.55578600 0.93485700

C 7.46592700 3.65024300 0.28521000

C 8.65940400 3.49171000 1.05138000

C 8.80300300 3.23543200 -1.74034500

C 9.99934700 3.51238800 -1.01370200

C 11.26590900 3.28210800 -1.62981700

C 12.46278900 3.53001200 -0.88929700

C 12.60488000 1.93883600 -3.19937200

C 13.80117000 2.53534300 -2.70017900

C 15.06791100 2.02902100 -3.11977200

C 16.26627300 2.60473300 -2.59211000

C 16.40826000 0.08689600 -3.81750500

C 17.59760300 0.85233600 -3.68339600

C 18.85846000 0.19868500 -3.83911200

C 20.03941800 1.01244400 -3.71399300

C -16.77803500 -3.03844600 -2.22524900

C -15.57757600 -2.53915500 -2.82039900

C -14.31463600 -3.00466800 -2.34771100

C -13.11548700 -2.48243800 -2.91786600

C -12.97799200 -3.78666100 -0.43439200

C -11.78521700 -3.64541300 -1.20509400

C -10.51875000 -3.80954000 -0.56981000

C -9.32673400 -3.64796700 -1.33560900

C -9.15813300 -3.51760800 1.46102800

C -7.97351000 -3.79379600 0.71572800

C -6.69390300 -3.59625000 1.32149100

C -5.50830700 -3.85792600 0.56246300

C -5.31856200 -2.27222700 2.86591300

C -4.13405900 -2.84166600 2.30903900

C -2.85845000 -2.34000300 2.72053200

C -1.67030200 -2.89058100 2.16218200

C -1.51412900 -0.46551100 3.58125700

C -0.33087700 -1.22223200 3.39007600

C 0.94081500 -0.60450900 3.57500000

C 2.12820200 -1.36855000 3.36927200

C 2.30185300 1.43219200 3.39302500

C 3.48626500 0.66698300 3.60541500

C 4.76254500 1.28506800 3.43769700

C 5.94940000 0.51556400 3.63286700

C 6.12083700 2.93128600 2.21639600

C 7.30806600 2.38120500 2.78164300

C 8.58105400 2.82515200 2.30933500

C 9.77034000 2.26290900 2.86168000

C 9.92819000 3.61657000 0.40865500

C 11.11923400 3.43384600 1.16964100

C 12.38983400 3.58139000 0.53243900

C 13.58299100 3.37628800 1.28893600

C 13.73189900 3.32591100 -1.51251000

C 14.92433600 3.54254600 -0.76351900

C 16.19636300 3.34816800 -1.38791100

C 17.39925100 3.54720500 -0.64003800

C 17.53885800 2.12489200 -3.03448400

C 18.73680900 2.70008300 -2.52420500

C 19.97907500 2.21423600 -3.06818800

C -11.85098700 -2.96052500 -2.45476000

C -10.65092500 -2.41945600 -3.00515400

C -9.38897400 -2.91708000 -2.55996000

C -8.18578500 -2.35716200 -3.08550700

C -8.06223400 -3.84092900 -0.70788400

C -6.87343300 -3.66100600 -1.47193800

C -5.60778400 -3.89510000 -0.85647000

C -4.42348400 -3.74414700 -1.62379600

C -4.22159800 -3.63615700 1.12952500

C -3.04007000 -3.88426600 0.33425200

C -1.73447100 -3.64826300 0.94317000

C -0.56330800 -3.90316700 0.23496400

C -0.41173500 -2.43917000 2.64108300

C 0.77766300 -2.98459500 2.09070500

C 2.04867400 -2.57216100 2.60788800

C 3.24101100 -3.13415200 2.07632800

C 3.40070300 -0.75899800 3.57613800

C 4.58900800 -1.51819300 3.35638200

C 5.86466900 -0.90831100 3.57336300

C 7.05466400 -1.65843900 3.33899800

C 7.22352700 1.14152800 3.48462200

C 8.41192200 0.37027200 3.65950400

C 9.68710000 1.00357200 3.53003000

C 10.87508400 0.23141500 3.68530300

C 11.04081900 2.72485100 2.40574300

C 12.23135300 2.14538000 2.93993800

C 13.50452800 2.62515600 2.50152600

C 14.69190300 2.03128100 3.01438300

C 14.85012100 3.54313100 0.65924300

C 16.04480200 3.29859500 1.40426600

C 17.32315100 3.49820300 0.79033000

C 18.50937700 3.28776600 1.54521600

C 18.66538900 3.41410800 -1.28120100

C 19.82269500 3.66515100 -0.47201200

C -6.92833700 -2.87963800 -2.65928900

C -5.71150000 -2.29495500 -3.13861600

C -4.46815900 -2.84499700 -2.73773400

C -3.15451600 -4.04591600 -1.05075400

C -1.96538900 -4.12287900 -2.01882000

C -0.58850000 -4.42923400 -1.21100100

C 0.60459700 -3.75317200 -1.90135000

C 0.69796500 -3.69534700 0.86150900

C 1.92508700 -3.95056300 0.15539200

C 3.17247000 -3.80553500 0.81201500

C 4.38451600 -3.95119500 0.06230200

C 4.50997400 -2.71337300 2.58186300

C 5.71131800 -3.24481500 2.01571100

C 6.97799200 -2.83215900 2.52725900

C 8.17700400 -3.32727600 1.92689800

C 8.32866800 -1.04991600 3.56474600

C 9.52281800 -1.79064900 3.30488600

C 10.79368200 -1.18792700 3.54850000

C 11.98738800 -1.91666800 3.25610900

C 12.14918800 0.86988500 3.56920500

C 13.34140400 0.09383300 3.70183700

C 14.61047700 0.73433100 3.60346000

C 15.80288700 -0.04688800 3.69390900

C 15.96565800 2.51536300 2.58224500

C 17.16488800 1.91641200 3.08465700

C 18.43089400 2.44860700 2.71340400

C 19.58329700 1.81390400 3.27780200

C 19.74817200 3.59504100 0.89365200

C -1.97931800 -2.78273900 -2.77499300

C 0.49714900 -2.72956500 -2.84837900

C 1.87495900 -3.90991700 -1.27532100

C 3.05979100 -3.53951300 -1.96235700

C 4.32539500 -3.77402000 -1.34719000

C 5.51529000 -3.42749700 -2.05105600

C 5.64666700 -3.84370900 0.72037500

C 6.84424000 -3.94245700 -0.04770300

C 8.10977500 -3.86359400 0.60818700

C 9.30710900 -3.93480400 -0.16744100

C 9.44752800 -2.93283800 2.45116400

C 10.64208500 -3.40392800 1.83025000

C 11.91191500 -3.02453700 2.36411300

C 13.10899700 -3.47275900 1.72299400

C 13.26131300 -1.32083400 3.51884400

C 14.45072200 -2.03575400 3.20069700

C 15.72320300 -1.44327300 3.46741500

C 16.92492200 -2.14989700 3.13598100

C 17.08330200 0.59351300 3.62998800

C 18.26802700 -0.18069900 3.75882200

C 19.50526200 0.53791300 3.77477400

C 2.96587400 -2.58718200 -3.01622300

C 5.43225300 -2.46406900 -3.10130900

C 6.77832200 -3.70282100 -1.45430000

C 7.97059200 -3.33861600 -2.14614000

C 9.23784300 -3.64230200 -1.56294000

C 10.42949100 -3.26430700 -2.24539400

C 10.57237700 -3.88883600 0.48809100

C 11.76696800 -3.92822700 -0.29140600

C 13.03754100 -3.90742300 0.36433500

C 14.22840600 -3.92296300 -0.41371200

C 14.37485800 -3.11386700 2.26738600

C 15.56936800 -3.52366700 1.60145600

C 16.84812400 -3.19664800 2.16024700

C 18.03471600 -3.64384300 1.51939200

C 18.18936800 -1.59452600 3.47501200

C 19.34117900 -2.37017800 3.13532100

C 7.89319200 -2.34728300 -3.16746600

C 11.69623300 -3.58806300 -1.67310600

C 12.89213500 -3.19019300 -2.34540000

C 14.15817300 -3.53675200 -1.78900200

C 15.35029900 -3.11057800 -2.43877100

C 15.49616600 -3.91016000 0.24009900

C 16.70080900 -3.91282100 -0.53642600

C 17.96139900 -3.99051200 0.11603400

C 19.11223000 -4.02719400 -0.72639200

C 19.26726900 -3.35608600 2.18199900

C 16.63053300 -3.47952700 -1.90096700

C 17.82022200 -3.10233000 -2.57548000

C 19.04329200 -3.58995500 -2.02895100

H -21.49949500 -1.18105200 -3.86556800

H -20.37216600 1.08994900 -3.88426200

H -20.43215500 3.05109000 -2.99011300

H -20.66088100 3.88828600 1.37557400

H -20.55222000 4.08721800 -0.81012600

H -20.79804900 2.36428400 3.24755200

H -20.90490800 0.33459700 4.06847500

H -21.16796700 -3.57863100 1.94787700

H -21.05131200 -2.00851000 3.48097400

H -21.30885000 -3.98176500 -0.43492500

H 19.89161800 -1.66451500 -3.73640200

H -21.41333700 -3.17608500 -2.46202600

H 20.99985000 0.60069800 -4.01391600

H 20.89963900 2.75239600 -2.86478600

H 20.79564900 3.77638000 -0.93989600

H 20.55857500 2.28105400 3.18468400

H 20.66370100 3.65334000 1.47415600

H 20.42067700 0.03081700 4.06370300

H 20.31598500 -2.08832500 3.52050000

H 20.08555300 -4.25922700 -0.30568300

H 20.18553100 -3.82597800 1.84347500

H 19.96500200 -3.49362000 -2.59452800

C -2.14213500 -5.25727900 -3.12455900

H -1.15762800 -5.35209000 -3.59759700

H -2.34388900 -6.20453900 -2.62045700

C -0.42562100 -6.01188300 -1.10984000

H -1.41175800 -6.38462600 -0.80884000

H -0.23097800 -6.40515800 -2.10979900

C -3.15652100 -5.14886700 -4.27596000

C -2.97307600 -6.31702300 -5.25975100

H -3.03652700 -4.20756600 -4.82296500

H -4.18441100 -5.16928000 -3.90073700

C -3.96383700 -6.27029500 -6.42755400

H -1.94574300 -6.30686700 -5.65109200

H -3.08504500 -7.26994300 -4.72308400

H -3.81132700 -7.11146500 -7.11323100

H -5.00015900 -6.31416500 -6.07114200

H -3.85193000 -5.34465800 -7.00510400

C 0.58736200 -6.68804000 -0.16998500

C 0.40860600 -8.21577500 -0.20303200

H 0.46306500 -6.34312600 0.86234000

H 1.61579400 -6.45381400 -0.46147300

C 1.40082400 -8.94862400 0.70561900

H -0.61837300 -8.47059800 0.09546600

H 0.52386000 -8.57586300 -1.23536800

H 1.25190500 -10.03357800 0.66472200

H 2.43669500 -8.74277500 0.40975600

H 1.28726600 -8.63551900 1.75061100

**(6,5) SWNT-[(CH_2_)_3_CH_3_]_2_ (1,2-L–: L_–33_)**

C -20.53885500 -1.76323700 -3.71085000

C -19.35201500 -0.98082500 -3.93745600

C -19.39337500 0.41498800 -4.06174100

C -18.23691900 1.19078200 -3.84331500

C -18.09586900 -1.61944700 -3.70366200

C -16.90069500 -0.88516100 -3.93118000

C -15.63278000 -1.53135200 -3.75625800

C -14.43305900 -0.79781500 -3.97364400

C -19.50897500 3.00549700 -2.62038400

C -18.28858100 2.44439800 -3.09852200

C -17.09763100 2.89111900 -2.47057700

C -16.96602700 0.53633900 -3.96043100

C -15.75498800 1.26720800 -3.73800100

C -14.49232600 0.62978900 -3.93567400

C -13.29169000 1.35455000 -3.68179400

C -13.17133200 -1.44664300 -3.81232000

C -11.96578100 -0.69862200 -3.98797700

C -10.70470100 -1.35436800 -3.85194900

C -9.49962900 -0.60086900 -3.99002800

C -19.74829500 3.25433000 1.58593400

C -19.57897300 3.59478700 -1.37915100

C -18.43165200 3.64635600 -0.53296400

C -17.16961000 3.48464900 -1.16777200

C -15.96761100 3.56674600 -0.39228400

C -15.81831300 2.44901800 -2.95430700

C -14.62447200 2.94131400 -2.35657800

C -13.35792100 2.51720100 -2.85875100

C -12.16009800 2.98461900 -2.23504000

C -12.02431200 0.72268800 -3.89350900

C -10.82383900 1.44054900 -3.61301200

C -9.55842500 0.81589200 -3.84018200

C -8.35608900 1.52308100 -3.52857700

C -8.23607900 -1.26258600 -3.87573100

C -7.03007800 -0.50555200 -3.97479000

C -5.76774800 -1.17263400 -3.87287400

C -4.55560100 -0.41416900 -3.92039300

C -3.24203800 -2.42808700 -3.34964300

C -0.75059400 -2.33736600 -3.32818400

C -19.82874600 2.38734900 2.64826800

C -18.68035700 1.65607700 3.08395100

C -18.51212400 3.46193000 0.90062100

C -17.32975600 3.08804200 1.59376600

C -16.04812800 3.34372600 1.00519100

C -14.85865600 3.01216800 1.71989700

C -14.69783900 3.49327600 -1.03900100

C -13.51023000 3.56622100 -0.26001700

C -12.23646400 3.49794600 -0.90763800

C -11.04742700 3.55300100 -0.12306100

C -10.89211400 2.57753500 -2.75104600

C -9.69862500 3.02615000 -2.11240900

C -8.42758700 2.63342100 -2.63550200

C -7.23653300 3.07079700 -1.98470100

C -7.09065400 0.90624500 -3.77140000

C -5.89142200 1.60301700 -3.43091500

C -4.62264700 0.99299200 -3.68099900

C -3.43203000 1.69081900 -3.32685800

C -3.29645200 -1.08140500 -3.81743100

C -2.09416800 -0.30993500 -3.82818000

C -0.83025500 -0.96954000 -3.74668600

C 0.35573600 -0.19100400 -3.79826100

C 1.67827500 -2.21193100 -3.38531300

C 4.14997800 -2.10444100 -3.53925200

C -20.00174600 -0.41908600 3.64844000

C -18.76302800 0.28857100 3.54051900

C -17.58037500 -0.49928600 3.50990900

C -17.41330100 2.16293600 2.68493500

C -16.21490900 1.49801400 3.10335300

C -14.94073000 2.05149600 2.77349800

C -13.75493500 1.37616300 3.17874300

C -13.59043600 3.30038100 1.14105100

C -12.39927100 2.93220000 1.83897400

C -11.12806000 3.24347100 1.26957500

C -9.94045700 2.85507400 1.95472800

C -9.77711000 3.50443900 -0.77057900

C -8.58699700 3.53842200 0.01699000

C -7.31617400 3.51565000 -0.63027800

C -6.12790800 3.53149500 0.15915900

C -5.96592200 2.69278800 -2.51412100

C -4.77558500 3.12321900 -1.85195700

C -3.50646300 2.76700700 -2.39169900

C -2.31642200 3.18298300 -1.71984800

C -2.16462900 1.08773900 -3.58160100

C -0.97198000 1.79508900 -3.23405100

C 0.28825800 1.21366000 -3.52777400

C 1.48180900 1.89836000 -3.16635000

C 1.61572700 -0.84171500 -3.76790900

C 2.81328700 -0.06186100 -3.83177800

C 4.07739700 -0.71332500 -3.87673700

C 5.27215200 0.06593700 -3.88349600

C 6.62335600 -1.97592400 -3.61931600

C 9.08817900 -1.85304800 -3.67163900

C -20.25650700 -3.80411200 1.16713700

C -20.08323100 -1.74730500 3.31581000

C -18.93317600 -2.45030900 2.83371500

C -17.66530500 -1.87957500 3.13442100

C -16.46767300 -2.53936600 2.70907500

C -16.29812100 0.13943100 3.49642800

C -15.10760300 -0.65048600 3.50316500

C -13.83703500 -0.00697600 3.52597400

C -12.64534000 -0.79567800 3.48555000

C -12.48019300 1.93646800 2.85518900

C -11.29018400 1.24523000 3.23554800

C -10.02011800 1.81980200 2.93449900

C -8.82784300 1.11450400 3.28274500

C -8.66802700 3.18651500 1.39689800

C -7.47901700 2.77995600 2.07088500

C -6.20856900 3.13465500 1.52658800

C -5.01642600 2.70498100 2.18502100

C -4.85503500 3.53281300 -0.48715000

C -3.66607300 3.52888500 0.30160500

C -2.39392800 3.54928600 -0.34543300

C -1.19902000 3.51516800 0.44095300

C -1.04526100 2.84815000 -2.27504700

C 0.14705500 3.24152500 -1.59980200

C 1.41333600 2.91453800 -2.16825700

C 2.61218600 3.27188100 -1.47890600

C 2.74645100 1.31999700 -3.49106800

C 3.94371400 1.99172900 -3.11088300

C 5.20627000 1.42913200 -3.46532000

C 6.40680300 2.08139600 -3.04878900

C 6.54607500 -0.58517100 -3.93485000

C 7.73953700 0.19430700 -3.90308200

C 9.01232600 -0.45798700 -3.95739000

C 10.20854000 0.31913100 -3.89769000

C 10.35835600 -2.43517900 -3.37165600

C 11.55079600 -1.73713000 -3.72051400

C 12.82228400 -2.32972000 -3.43444600

C 14.01629000 -1.62662700 -3.76406400

C -20.33382200 -4.03056400 -0.18142500

C -19.17707400 -3.87747800 -1.01546800

C -19.01574600 -3.42561100 1.77669500

C -17.83143600 -3.72789900 1.05084900

C -16.55114200 -3.46019300 1.63548900

C -15.35844700 -3.79617000 0.92398500

C -15.19167500 -2.00936400 3.07654000

C -14.00527400 -2.66316500 2.63763900

C -12.72891100 -2.13609400 3.01084200

C -11.53842800 -2.77505900 2.54584000

C -11.37056500 -0.15050300 3.52877900

C -10.18148800 -0.93490400 3.45744900

C -8.90638900 -0.29026800 3.51788800

C -7.71470900 -1.06985800 3.41515500

C -7.55770700 1.70548200 3.00843500

C -6.36625100 0.98938200 3.32659100

C -5.09521500 1.59430100 3.07749300

C -3.90316200 0.86911100 3.36827900

C -3.74581800 3.08321400 1.65612500

C -2.55334700 2.62608100 2.29425500

C -1.27881500 3.01992500 1.77726700

C -0.08675800 2.53687500 2.39621600

C 0.07165300 3.55873800 -0.20683200

C 1.26560700 3.48182300 0.57177600

C 2.53841100 3.53561100 -0.07926000

C 3.73246300 3.42685800 0.69560900

C 3.87699600 2.96498400 -2.06616100

C 5.07407900 3.28951700 -1.36391800

C 6.34035900 3.00375600 -1.96202900

C 7.53668800 3.30289000 -1.24602800

C 7.67180800 1.54040000 -3.42935600

C 8.87063300 2.17192000 -2.97928900

C 10.13938300 1.64725500 -3.37735700

C 11.33540400 2.25974800 -2.89879400

C 11.47724600 -0.33431300 -3.96666200

C 12.67539100 0.43931700 -3.87540400

C 13.94359800 -0.21297900 -3.96441200

C 15.13694600 0.55037100 -3.83354000

C 15.28301200 -2.22665300 -3.48957400

C 16.48804600 -1.51681900 -3.79340600

C 17.76366200 -2.14253800 -3.59628500

C 18.91376600 -1.38960400 -3.90780500

C -20.48644400 -2.87940700 -2.92564300

C -19.24666200 -3.30856000 -2.33157900

C -18.04583900 -2.80536200 -2.90709600

C -17.91075000 -3.94278000 -0.36420600

C -16.70837600 -3.83877900 -1.13137700

C -15.43667700 -3.96602700 -0.48889100

C -14.24566300 -3.84829200 -1.26026700

C -14.08893800 -3.55688900 1.52659100

C -12.89775800 -3.85692900 0.79883100

C -11.62343900 -3.63274200 1.40819000

C -10.43576600 -3.91426000 0.67281000

C -10.26422500 -2.26119400 2.93516000

C -9.07386000 -2.88080400 2.44453400

C -7.79558800 -2.37854300 2.84624900

C -6.60879400 -2.98047900 2.33412200

C -6.44389100 -0.42513800 3.49889000

C -5.24982500 -1.19715900 3.35698100

C -3.98123700 -0.55428800 3.47464200

C -2.79059100 -1.31537400 3.29273100

C -2.63368100 1.48222700 3.14174700

C -1.43963600 0.74179300 3.40018000

C -0.16885200 1.35757600 3.20175800

C 1.01861200 0.60064100 3.42477000

C 1.18571000 2.93641600 1.88839600

C 2.37859300 2.41703000 2.48393100

C 3.65247200 2.83320300 1.99204200

C 4.83960000 2.29015200 2.56870300

C 5.00167500 3.49930900 0.04596300

C 6.19516200 3.35784200 0.81684600

C 7.46408000 3.45664100 0.17133800

C 8.65514600 3.28878500 0.93809700

C 8.80310500 3.04486700 -1.85316200

C 9.99939900 3.31688400 -1.12342200

C 11.26610900 3.08714000 -1.73848600

C 12.46143700 3.33007700 -0.99528900

C 12.60584600 1.74615000 -3.31004000

C 13.80201300 2.34096800 -2.80761300

C 15.06902600 1.83654900 -3.22802900

C 16.26660900 2.40960600 -2.69618100

C 16.41033500 -0.10230200 -3.93344700

C 17.59947300 0.66290000 -3.79468400

C 18.86060700 0.01059000 -3.95282800

C 20.04115400 0.82375900 -3.82212600

C -16.77603200 -3.24047100 -2.41405300

C -15.57520200 -2.74018300 -3.00777600

C -14.31250000 -3.20496400 -2.53401800

C -13.11365400 -2.68193300 -3.10330800

C -12.97649800 -3.98329100 -0.61801100

C -11.78215900 -3.84010200 -1.38712000

C -10.51598400 -4.00015500 -0.75016700

C -9.32365100 -3.83460100 -1.51377600

C -9.16081200 -3.70516800 1.28522100

C -7.97217800 -3.97198700 0.54122900

C -6.69695600 -3.78224700 1.15590500

C -5.51208100 -4.03227600 0.40053700

C -5.32854800 -2.48031100 2.73652500

C -4.14032000 -3.05677800 2.19724400

C -2.87004200 -2.56008200 2.60366600

C -1.67861400 -3.09883300 2.02235900

C -1.51954600 -0.68114300 3.44234700

C -0.33261200 -1.43858700 3.23798300

C 0.93381700 -0.82269200 3.41203500

C 2.11998000 -1.58675700 3.18176600

C 2.29431600 1.21373200 3.24182300

C 3.47932100 0.44279000 3.44652600

C 4.75327200 1.06129200 3.28926900

C 5.93863700 0.28641500 3.47692400

C 6.11424200 2.71995900 2.09017600

C 7.30041000 2.16148800 2.65315700

C 8.57370000 2.60932000 2.18967500

C 9.76155400 2.03942900 2.73879300

C 9.92563400 3.41455800 0.29867100

C 11.11509500 3.22458700 1.06085600

C 12.38620000 3.37425100 0.42692800

C 13.57794700 3.16232800 1.18413000

C 13.73158200 3.12696200 -1.61714100

C 14.92267000 3.33947800 -0.86525500

C 16.19528900 3.14716000 -1.48834500

C 17.39713400 3.34141700 -0.73730100

C 17.53991700 1.93202300 -3.13924900

C 18.73711900 2.50461700 -2.62452300

C 19.97993400 2.02202700 -3.16965200

C -11.84818400 -3.15846300 -2.63850600

C -10.64942700 -2.61900500 -3.19046300

C -9.38722300 -3.11185000 -2.74076800

C -8.18395900 -2.55424500 -3.27186700

C -8.05596800 -4.01927400 -0.88279400

C -6.86727600 -3.84023800 -1.64341400

C -5.60279100 -4.04970500 -1.01804000

C -4.41235900 -3.86706200 -1.78099400

C -4.22363300 -3.84424900 1.00247600

C -3.05341300 -4.08557500 0.24084800

C -1.75146800 -3.84129500 0.80913000

C -0.56881400 -4.10329200 0.07098000

C -0.41280900 -2.65274200 2.48255300

C 0.76697000 -3.19809600 1.91075900

C 2.03676900 -2.77937000 2.41343700

C 3.23253900 -3.34699100 1.87622700

C 3.39333000 -0.98126400 3.39910600

C 4.57768900 -1.74248400 3.17947000

C 5.85256100 -1.13554100 3.40576600

C 7.04500800 -1.88666600 3.17615700

C 7.21377600 0.91377200 3.34139000

C 8.40044000 0.14074100 3.51510300

C 9.67602900 0.77455600 3.39503100

C 10.86411700 0.00103900 3.55002600

C 11.03352800 2.50521900 2.29101100

C 12.22207800 1.92086900 2.82329100

C 13.49649900 2.40309200 2.39115500

C 14.68272000 1.80572700 2.90305100

C 14.84611400 3.33233300 0.55775800

C 16.03894500 3.08240000 1.30321700

C 17.31857500 3.28456100 0.69258800

C 18.50327800 3.06974900 1.44853200

C 18.66414100 3.21154900 -1.37717600

C 19.82007200 3.45693600 -0.56466400

C -6.92550700 -3.07246800 -2.84579300

C -5.71931500 -2.49117300 -3.34183700

C -4.45945500 -3.03461000 -2.93372200

C -3.15315000 -4.14417000 -1.18547600

C -1.95891900 -3.99349900 -1.94735300

C -0.68358700 -4.61582000 -1.37418000

C 0.59616300 -4.25593400 -2.30847500

C 0.67707300 -3.91498900 0.67333900

C 1.90714300 -4.15989400 -0.07090200

C 3.16776300 -4.01680300 0.61787900

C 4.38103600 -4.18988300 -0.10423400

C 4.49765600 -2.93653600 2.39908600

C 5.70342100 -3.47059600 1.84461600

C 6.97123300 -3.05963300 2.36612100

C 8.17109500 -3.55818300 1.77455700

C 8.31705900 -1.27967000 3.41208400

C 9.51263200 -2.01958500 3.15493700

C 10.78335100 -1.41738400 3.40642700

C 11.97795200 -2.14515300 3.11708700

C 12.13805400 0.64048000 3.44317900

C 13.33031500 -0.13613500 3.57485000

C 14.59977800 0.50527900 3.48433300

C 15.79204200 -0.27592500 3.57433400

C 15.95715100 2.29226200 2.47662300

C 17.15538800 1.69050600 2.97821800

C 18.42218000 2.22481900 2.61269800

C 19.57313400 1.58779000 3.17691300

C 19.74319500 3.37942200 0.80066300

C -1.98540700 -3.01215200 -2.94517200

C 0.49102400 -2.89600800 -3.01829100

C 1.87510000 -4.20119900 -1.46992600

C 3.07012300 -3.80439700 -2.13579800

C 4.32998000 -4.01716700 -1.51554900

C 5.52083100 -3.64330600 -2.20467800

C 5.64462600 -4.07825000 0.55948100

C 6.84649600 -4.17885500 -0.20416600

C 8.10857300 -4.09693600 0.45531900

C 9.30811000 -4.15825000 -0.31803700

C 9.44053600 -3.16099600 2.30183200

C 10.63808700 -3.63112100 1.68433700

C 11.90567200 -3.25139100 2.22217500

C 13.10491000 -3.69561500 1.58200500

C 13.25111500 -1.54968500 3.38546600

C 14.44210900 -2.26318000 3.06838000

C 15.71344400 -1.67144500 3.34113900

C 16.91649000 -2.37592100 3.00928700

C 17.07256400 0.36507000 3.51684600

C 18.25726300 -0.40935600 3.64535700

C 19.49408000 0.30943100 3.66795200

C 2.97555700 -2.79007000 -3.14131200

C 5.43467700 -2.66368700 -3.23217800

C 6.78477900 -3.92542600 -1.60821000

C 7.97388000 -3.54970400 -2.29308000

C 9.24135400 -3.85507300 -1.71022900

C 10.43315500 -3.46857200 -2.38792300

C 10.57249200 -4.11205200 0.34081800

C 11.76772200 -4.14480300 -0.43651700

C 13.03699600 -4.12431000 0.22163900

C 14.22924700 -4.13380700 -0.55452800

C 14.36937800 -3.33758600 2.13063500

C 15.56545600 -3.74321400 1.46528800

C 16.84274300 -3.41764500 2.02808000

C 18.03109600 -3.86045100 1.38718100

C 18.17986600 -1.82185600 3.35439100

C 19.33282300 -2.59503700 3.01330800

C 7.89494200 -2.54489000 -3.30358400

C 11.69892900 -3.79533900 -1.81654800

C 12.89492900 -3.39015900 -2.48349500

C 14.16083100 -3.73928800 -1.92750400

C 15.35300500 -3.30806000 -2.57277000

C 15.49545500 -4.12283200 0.10165700

C 16.70138800 -4.11929300 -0.67272000

C 17.96093400 -4.19917200 -0.01836600

C 19.11313800 -4.22967800 -0.85895900

C 19.26182800 -3.57560200 2.05414900

C 16.63283200 -3.67860400 -2.03490800

C 17.82317400 -3.29686600 -2.70564300

C 19.04580100 -3.78571200 -2.15945400

H -21.49701000 -1.38212200 -4.05527800

H -20.36984400 0.88949200 -4.06907200

H -20.42927700 2.85148700 -3.17542500

H -20.66429800 3.68163500 1.18974200

H -20.55147000 3.88521200 -0.99432400

H -20.80620400 2.15401600 3.05837800

H -20.91551200 0.12199900 3.87380200

H -21.17119100 -3.79225900 1.75179100

H -21.05955100 -2.22040800 3.28335000

H -21.30792900 -4.19226600 -0.63191700

H 19.89445300 -1.85275400 -3.85829900

H -21.41107900 -3.38025600 -2.65623200

H 21.00202100 0.41373500 -4.12301000

H 20.90025200 2.55899700 -2.96202100

H 20.79404200 3.56941500 -1.03012500

H 20.54860500 2.05542100 3.08828600

H 20.65797000 3.43332700 1.38276900

H 20.40893100 -0.19878100 3.95669900

H 20.30658500 -2.31466400 3.40217900

H 20.08614300 -4.46232300 -0.43783300

H 20.18124300 -4.04280100 1.71500200

H 19.96846300 -3.68502200 -2.72271100

C 0.69063700 -5.35282100 -3.45852300

H 0.95223100 -6.31129700 -3.00506700

H -0.32770800 -5.45386900 -3.85310600

C -0.79360200 -6.20102900 -1.27881200

H -1.06602600 -6.59091200 -2.26221300

H 0.22196000 -6.55595100 -1.06525200

C 1.61525700 -5.16968400 -4.67303100

C 1.40152200 -6.30430000 -5.68882500

H 2.66666400 -5.16844400 -4.36786700

H 1.42839400 -4.21160000 -5.17047900

C 2.31682100 -6.18729000 -6.91184800

H 1.57125500 -7.27307800 -5.19748300

H 0.35189000 -6.30852100 -6.01566800

H 2.14317900 -7.00614200 -7.61917400

H 2.14751400 -5.24415800 -7.44557000

H 3.37374500 -6.21671800 -6.62029900

C -1.71878600 -6.88443500 -0.25869500

C -1.54411300 -8.41164700 -0.30933600

H -2.76820500 -6.64787500 -0.46091100

H -1.50610300 -6.53926600 0.75925800

C -2.45203200 -9.14623700 0.68227700

H -1.75013800 -8.76999800 -1.32804200

H -0.49486600 -8.66704300 -0.10315500

H -2.30848100 -10.23119700 0.62531100

H -2.24529300 -8.83620600 1.71384300

H -3.50993600 -8.93873900 0.48055600

**(6,5) SWNT-[(CH_2_)_3_CH_3_]_2_ (1,4-L_++_: L_87_)**

C -20.54603800 -1.67438100 -3.35944300

C -19.35948400 -0.89205900 -3.58723300

C -19.40058700 0.50388900 -3.71155600

C -18.24342900 1.27947900 -3.49640800

C -18.10318000 -1.53118600 -3.35555300

C -16.90825700 -0.79728000 -3.58510800

C -15.64047100 -1.44388400 -3.41205900

C -14.44138200 -0.71078500 -3.63247800

C -19.51122000 3.09831700 -2.27517600

C -18.29243900 2.53465700 -2.75402700

C -17.09980100 2.98161200 -2.12900800

C -16.97312000 0.62428700 -3.61539800

C -15.76150300 1.35501600 -3.39687800

C -14.50000400 0.71677500 -3.59663900

C -13.29851400 1.44187800 -3.34727000

C -13.17960400 -1.35970700 -3.47275700

C -11.97537100 -0.61221200 -3.65403000

C -10.71472500 -1.26740000 -3.51898700

C -9.51095800 -0.51376500 -3.66741200

C -19.74445400 3.35194100 1.92954100

C -19.57829500 3.69035200 -1.03504000

C -18.42965300 3.74147800 -0.19067600

C -17.16866600 3.57715200 -0.82698800

C -15.96529000 3.65770600 -0.05326600

C -15.82193000 2.53808600 -2.61452800

C -14.62645500 3.02975500 -2.01934700

C -13.36125300 2.60488600 -2.52435300

C -12.16135300 3.07031400 -1.90310500

C -12.03284200 0.80978300 -3.56299700

C -10.83156800 1.52794600 -3.28764300

C -9.56764600 0.90361500 -3.52229600

C -8.36354600 1.61034000 -3.21800300

C -8.24860200 -1.17432900 -3.55729200

C -7.04541200 -0.41700900 -3.67434600

C -5.78323000 -1.08429300 -3.58374300

C -4.57018600 -0.33008000 -3.66635100

C -3.26956200 -2.33237000 -3.09038600

C -0.78382500 -2.43879200 -3.50996200

C -19.82553500 2.48388500 2.99095800

C -18.67808800 1.75012900 3.42470300

C -18.50865000 3.55765000 1.24301100

C -17.32612000 3.18122200 1.93448900

C -16.04452700 3.43468000 1.34443600

C -14.85486600 3.10051300 2.05740400

C -14.69648400 3.58161300 -0.70165700

C -13.50699300 3.65138200 0.07553700

C -12.23413800 3.58076800 -0.57391000

C -11.04334500 3.62974200 0.20864000

C -10.89535200 2.66279200 -2.42314400

C -9.69814900 3.10609700 -1.78573400

C -8.42977200 2.71472700 -2.31500700

C -7.23502900 3.13891000 -1.66204900

C -7.10152000 0.99404200 -3.47258800

C -5.89525900 1.68510300 -3.13382500

C -4.63212200 1.07799400 -3.40301100

C -3.43508500 1.75233500 -3.02682300

C -3.31989500 -1.00335300 -3.59567100

C -2.09210000 -0.24226500 -3.61434100

C -0.84384400 -0.89815400 -3.62233100

C 0.33625400 -0.13582800 -3.59026300

C 1.71263500 -2.14072700 -3.21784400

C 4.17064500 -2.00571700 -3.26760300

C -20.00184500 -0.32394600 3.98846900

C -18.76247200 0.38247700 3.88060500

C -17.58063400 -0.40664400 3.85045400

C -17.41049100 2.25554100 3.02507800

C -16.21286200 1.58912700 3.44270700

C -14.93814700 2.14074700 3.11162100

C -13.75327300 1.46420100 3.51727100

C -13.58617200 3.38540500 1.47671200

C -12.39507700 3.01509200 2.17324800

C -11.12336700 3.32087000 1.60121900

C -9.93583600 2.93050100 2.28601100

C -9.77315300 3.57775900 -0.44093400

C -8.58187700 3.60422900 0.34418500

C -7.31154000 3.57456800 -0.30508200

C -6.12172600 3.58319500 0.48299400

C -5.96523800 2.76114600 -2.19828500

C -4.77327500 3.17444600 -1.53292500

C -3.50577600 2.81102100 -2.07466400

C -2.31279800 3.21572000 -1.40013600

C -2.16683200 1.14872800 -3.29894200

C -0.97707600 1.83572900 -2.93625900

C 0.27963500 1.24987800 -3.23875100

C 1.47871300 1.93169700 -2.86818500

C 1.63128000 -0.77981800 -3.61130200

C 2.81464100 0.00769900 -3.62773900

C 4.09130400 -0.63715300 -3.64130700

C 5.28732800 0.14775300 -3.62664400

C 6.63102700 -1.89174600 -3.31365500

C 9.09388100 -1.77602700 -3.35383400

C -20.25896200 -3.71579400 1.51504000

C -20.08442400 -1.65265400 3.65773300

C -18.93491300 -2.35774500 3.17747500

C -17.66666500 -1.78735900 3.47689500

C -16.46957900 -2.44900600 3.05261700

C -16.29784300 0.23082100 3.83664200

C -15.10848700 -0.56039400 3.84443900

C -13.83748600 0.08194400 3.86688100

C -12.64704900 -0.70835700 3.82895800

C -12.47770400 2.02192600 3.19223900

C -11.28947500 1.32986600 3.57484600

C -10.01781900 1.90069900 3.27140600

C -8.82786700 1.19573200 3.62562500

C -8.66279300 3.25489200 1.72551900

C -7.47512700 2.84675300 2.40028200

C -6.20288900 3.19253100 1.85268500

C -5.01335400 2.76494700 2.51470900

C -4.85023600 3.57782100 -0.16422100

C -3.66192300 3.56613200 0.62374300

C -2.38919000 3.57996500 -0.02395500

C -1.19739400 3.54552400 0.76036400

C -1.04660200 2.88125400 -1.95848200

C 0.14394700 3.25689800 -1.28016900

C 1.41136600 2.92897100 -1.85413500

C 2.60781300 3.29014700 -1.16740100

C 2.74576700 1.38177000 -3.22819800

C 3.93879500 2.04528500 -2.82502800

C 5.21332200 1.50194700 -3.19080800

C 6.40829400 2.15080300 -2.75822400

C 6.55651700 -0.50612200 -3.65393400

C 7.75106600 0.27259600 -3.61043400

C 9.02193100 -0.38318400 -3.65237500

C 10.21693000 0.39247600 -3.58839700

C 10.35937800 -2.35826900 -3.04379500

C 11.55449700 -1.66449900 -3.39378400

C 12.82345400 -2.25827500 -3.10178900

C 14.01901000 -1.55834900 -3.43182500

C -20.33707500 -3.94428800 0.16686300

C -19.18100700 -3.79121100 -0.66806600

C -19.01796300 -3.33572900 2.12291600

C -17.83401400 -3.63947300 1.39685300

C -16.55334000 -3.37137000 1.98029700

C -15.36093500 -3.70806700 1.26837000

C -15.19353700 -1.91969300 3.41946300

C -14.00762700 -2.57493100 2.98096600

C -12.73134600 -2.04911800 3.35496100

C -11.54141700 -2.68876300 2.89024400

C -11.37236900 -0.06442200 3.87278900

C -10.18451000 -0.85102600 3.80505600

C -8.90898700 -0.20803800 3.86871500

C -7.71959600 -0.98987900 3.77182200

C -7.55652600 1.78141600 3.34725100

C -6.36684300 1.06541200 3.67430800

C -5.09446300 1.66474500 3.42160800

C -3.90533100 0.94025000 3.72275500

C -3.74208400 3.13129500 1.98066000

C -2.55069300 2.67800700 2.62494600

C -1.27674700 3.06290700 2.10425300

C -0.08747900 2.58456800 2.72668600

C 0.07100100 3.57684100 0.11055300

C 1.26525900 3.50315700 0.88832700

C 2.53537600 3.55586700 0.23446600

C 3.72724800 3.45486600 1.00762800

C 3.87158300 2.99422400 -1.75879900

C 5.06906300 3.32158900 -1.05358900

C 6.33784100 3.05585200 -1.65502400

C 7.53160400 3.35403500 -0.93517000

C 7.67806300 1.61472200 -3.13505500

C 8.87566300 2.24446800 -2.67400400

C 10.14529500 1.72125000 -3.06901400

C 11.33984500 2.33142900 -2.58373500

C 11.48427300 -0.26356700 -3.64762500

C 12.68248400 0.50932500 -3.55440000

C 13.94913300 -0.14512500 -3.63710700

C 15.14272300 0.61727200 -3.50453900

C 15.28395100 -2.15938500 -3.15301200

C 16.49073100 -1.45172600 -3.45628700

C 17.76475900 -2.07882300 -3.25580200

C 18.91685300 -1.32740600 -3.56472500

C -20.49255000 -2.79159400 -2.57570300

C -19.25206700 -3.22152600 -1.98379800

C -18.05191600 -2.71769100 -2.56000300

C -17.91411200 -3.85585700 -0.01784400

C -16.71212200 -3.75169600 -0.78592800

C -15.43976300 -3.87833400 -0.14449100

C -14.24890900 -3.75955600 -0.91652400

C -14.09108100 -3.46858300 1.87009700

C -12.89948600 -3.76809200 1.14170300

C -11.62528000 -3.54430300 1.75080700

C -10.43660400 -3.82195900 1.01418600

C -10.26787400 -2.17622000 3.28199600

C -9.07687200 -2.79560600 2.79161900

C -7.80103800 -2.29652100 3.20013800

C -6.61277700 -2.89561800 2.68593700

C -6.44697000 -0.34706100 3.85851900

C -5.25461400 -1.12193400 3.72783100

C -3.98573300 -0.48071900 3.84666900

C -2.79479800 -1.24683600 3.67882900

C -2.63263400 1.54748700 3.48976300

C -1.44121600 0.81105900 3.76475000

C -0.16983700 1.42074500 3.55394000

C 1.01869300 0.66786700 3.79271800

C 1.18625500 2.97570300 2.21051800

C 2.37782400 2.46962700 2.81453600

C 3.64898100 2.87906200 2.31266100

C 4.83813200 2.34930400 2.89647800

C 4.99666600 3.52771700 0.35564400

C 6.18985100 3.40022900 1.12795200

C 7.45809200 3.50295200 0.48266500

C 8.65043500 3.34521200 1.25137300

C 8.80297400 3.10753100 -1.54192400

C 9.99709200 3.38094600 -0.80934400

C 11.26640200 3.15561300 -1.42234400

C 12.46109900 3.40006900 -0.67696600

C 12.61160000 1.81710500 -2.99166700

C 13.80620500 2.41150200 -2.48671000

C 15.07395700 1.90495500 -2.90300700

C 16.27075500 2.47884500 -2.37042700

C 16.41537500 -0.03736700 -3.59938700

C 17.60473500 0.72695000 -3.45986200

C 18.86556800 0.07267800 -3.61270100

C 20.04661500 0.88531500 -3.48183100

C -16.78140500 -3.15302300 -2.06842700

C -15.58138600 -2.65253400 -2.66310000

C -14.31787600 -3.11652700 -2.19012300

C -13.11978900 -2.59364300 -2.76130200

C -12.97847400 -3.89346700 -0.27531400

C -11.78423700 -3.74838700 -1.04471400

C -10.51645200 -3.90489200 -0.40845100

C -9.32308500 -3.73582000 -1.17239600

C -9.16143000 -3.61361400 1.62738500

C -7.97139500 -3.87208300 0.88245500

C -6.69783200 -3.67994600 1.49906500

C -5.50704500 -3.91273200 0.74151800

C -5.33295300 -2.40479300 3.10372100

C -4.14558700 -2.97982500 2.56767800

C -2.87326500 -2.49295300 2.99385700

C -1.67997600 -3.04145800 2.43159800

C -1.52226800 -0.61362200 3.83279600

C -0.33668600 -1.37513700 3.64843000

C 0.93421800 -0.75495600 3.81329500

C 2.12239000 -1.52021200 3.59905100

C 2.29465100 1.28051700 3.59903600

C 3.47992600 0.51839300 3.81697900

C 4.75405100 1.13602400 3.64142000

C 5.94168200 0.36794300 3.83862600

C 6.11107300 2.77722500 2.41000500

C 7.29827800 2.23198000 2.97944800

C 8.57080400 2.67693200 2.50841600

C 9.76014500 2.11657900 3.06372100

C 9.92163400 3.47643800 0.61350200

C 11.11109200 3.29327000 1.37716900

C 12.38383800 3.44498900 0.74469400

C 13.57549400 3.23807700 1.50383900

C 13.73273000 3.19878100 -1.29704700

C 14.92289500 3.41294800 -0.54398900

C 16.19698700 3.22009900 -1.16515000

C 17.39774900 3.41728900 -0.41339100

C 17.54453900 1.99871900 -2.80943000

C 18.74102100 2.57267000 -2.29472800

C 19.98483600 2.08641500 -2.83478400

C -11.85345300 -3.06822400 -2.29708400

C -10.65636900 -2.52854200 -2.85182400

C -9.39209800 -3.01693500 -2.40156100

C -8.19247300 -2.46022800 -2.93900200

C -8.05341300 -3.91483800 -0.54247100

C -6.86394400 -3.72620200 -1.30213700

C -5.59339900 -3.91958600 -0.67665400

C -4.40170900 -3.71662100 -1.43475400

C -4.22536800 -3.73484500 1.35279700

C -3.04692800 -3.93939900 0.58462100

C -1.74730700 -3.75400800 1.19264200

C -0.57188900 -3.99827000 0.46117300

C -0.41753700 -2.59889700 2.90671100

C 0.76619900 -3.14104200 2.33764400

C 2.04035200 -2.72218100 2.83728800

C 3.23171200 -3.27396900 2.28419200

C 3.39491300 -0.90821600 3.79659600

C 4.58161600 -1.66640700 3.57078400

C 5.85715100 -1.05542400 3.78280700

C 7.04675200 -1.80472500 3.54389000

C 7.21453800 0.99456100 3.68737900

C 8.40256100 0.22429000 3.86348400

C 9.67707500 0.85789900 3.73294200

C 10.86471300 0.08579800 3.88924100

C 11.03082100 2.58049300 2.61139800

C 12.22061200 2.00103000 3.14739900

C 13.49463600 2.48249600 2.71347800

C 14.68101200 1.88753400 3.22765700

C 14.84450400 3.40836100 0.87870500

C 16.03736800 3.16142500 1.62606100

C 17.31752100 3.36392700 1.01658800

C 18.50171900 3.15109300 1.77393100

C 18.66576700 3.28546500 -1.05115300

C 19.82063500 3.53528900 -0.23818600

C -6.93233100 -2.96728800 -2.50813800

C -5.73156800 -2.38815700 -3.02099800

C -4.46925100 -2.91608200 -2.61269200

C -3.13517300 -3.92595700 -0.83202800

C -1.93429800 -3.72365300 -1.60900600

C -0.64224400 -4.30412700 -1.05251800

C 0.57332300 -3.66605900 -1.72006700

C 0.68066800 -3.82050900 1.08402700

C 1.90398700 -4.01532100 0.33970100

C 3.15880800 -3.90871800 1.00022400

C 4.36669700 -4.04045700 0.24525300

C 4.50019300 -2.85676300 2.78752500

C 5.70119800 -3.37703700 2.20735600

C 6.96891700 -2.97248000 2.72368200

C 8.16614500 -3.46293800 2.11821200

C 8.31949900 -1.19612200 3.76889700

C 9.51329100 -1.93592200 3.50495000

C 10.78352100 -1.33356200 3.75064300

C 11.97659900 -2.06161000 3.45687600

C 12.13810200 0.72450500 3.77470600

C 13.33003700 -0.05198700 3.90768800

C 14.59888000 0.58871700 3.81286200

C 15.79087100 -0.19292400 3.90340300

C 15.95561800 2.37330700 2.80059800

C 17.15383400 1.77244500 3.30367900

C 18.42058600 2.30651700 2.93812200

C 19.57177100 1.66930800 3.50204900

C 19.74219100 3.46140400 1.12705500

C -1.99853500 -2.90173200 -2.70992600

C 0.50906200 -2.82701500 -2.80775400

C 1.84682800 -3.88739100 -1.07588000

C 3.04464600 -3.59378200 -1.77398500

C 4.30822600 -3.85185700 -1.16198400

C 5.50734100 -3.52243200 -1.86554900

C 5.63384100 -3.95378300 0.90492900

C 6.83194700 -4.05547300 0.13762700

C 8.09789400 -3.98845400 0.79522800

C 9.29756700 -4.05898600 0.02141200

C 9.43746100 -3.07353200 2.64612400

C 10.63185600 -3.54290800 2.02421300

C 11.90123000 -3.16684600 2.56150600

C 13.09902500 -3.61353700 1.92076600

C 13.25010600 -1.46620700 3.72216000

C 14.43944600 -2.18081900 3.40430300

C 15.71137300 -1.58888200 3.67394400

C 16.91337500 -2.29518500 3.34325300

C 17.07135800 0.44745500 3.84368400

C 18.25574000 -0.32733200 3.97253400

C 19.49284900 0.39112400 3.99343000

C 2.97740600 -2.67391700 -2.86171400

C 5.43944400 -2.56479400 -2.92018400

C 6.76887000 -3.80890600 -1.26843200

C 7.96555000 -3.45023200 -1.95702000

C 9.23185300 -3.75995500 -1.37228800

C 10.42702500 -3.38453300 -2.05166000

C 10.56269300 -4.02084400 0.67932800

C 11.75908300 -4.05908000 -0.09764800

C 13.02910600 -4.04320300 0.56060900

C 14.22188600 -4.05778400 -0.21482400

C 14.36424800 -3.25713100 2.46869800

C 15.55958700 -3.66602200 1.80420800

C 16.83759200 -3.34068100 2.36613400

C 18.02506300 -3.78772500 1.72701700

C 18.17734600 -1.74063600 3.68535800

C 19.32939000 -2.51614800 3.34645200

C 7.89750900 -2.45952400 -2.97978200

C 11.69202900 -3.71306800 -1.47812100

C 12.89091100 -3.31513300 -2.14636900

C 14.15538200 -3.66598300 -1.58879600

C 15.34978500 -3.23893800 -2.23410700

C 15.48843700 -4.04890800 0.44168700

C 16.69494500 -4.05047800 -0.33212100

C 17.95406300 -4.13179900 0.32285400

C 19.10664700 -4.16765600 -0.51712100

C 19.25659500 -3.50113100 2.39199700

C 16.62840700 -3.61173800 -1.69501200

C 17.82027400 -3.23323200 -2.36510300

C 19.04128800 -3.72526500 -1.81812400

H -21.50470100 -1.29277600 -3.70191000

H -20.37693700 0.97868800 -3.71714300

H -20.43266800 2.94421100 -2.82830500

H -20.66002400 3.78143200 1.53472000

H -20.54977400 3.98273600 -0.64914500

H -20.80301500 2.25203300 3.40186900

H -20.91531600 0.21823200 4.21239400

H -21.17320700 -3.70346100 2.10038400

H -21.06125900 -2.12469100 3.62528900

H -21.31138400 -4.10707400 -0.28279100

H 19.89680000 -1.79174600 -3.51186700

H -21.41688100 -3.29253900 -2.30545100

H 21.00786200 0.47329400 -3.77875500

H 20.90512000 2.62347900 -2.62728800

H 20.79483200 3.64841100 -0.70302900

H 20.54726100 2.13674000 3.41270800

H 20.65595700 3.51869500 1.71042600

H 20.40778100 -0.11710000 4.28191500

H 20.30373200 -2.23503600 3.73334800

H 20.07862300 -4.40296000 -0.09510900

H 20.17528400 -3.97079300 2.05432900

H 19.96433800 -3.62830300 -2.38141100

C -0.88811300 -3.13306400 -4.92723800

H -1.87280200 -2.87138300 -5.33346400

H -0.90389500 -4.21705500 -4.75038900

C -0.54900000 -5.85627100 -1.34100700

H -0.54586200 -5.97793500 -2.43263600

H 0.43841000 -6.18185200 -0.99114800

C -1.61169800 -6.77662000 -0.72913300

C -1.30463100 -8.25751600 -1.00064600

H -2.60278900 -6.53809900 -1.13305900

H -1.66882200 -6.61553300 0.35536900

C -2.35815400 -9.20307200 -0.41476500

H -1.22895800 -8.42054000 -2.08520800

H -0.31754900 -8.50613200 -0.58592700

H -2.11364900 -10.25070000 -0.62376600

H -2.43205600 -9.08865300 0.67355600

H -3.35048900 -9.00157500 -0.83632300

C 0.17864400 -2.80303500 -5.97768700

C -0.13612700 -3.45138000 -7.33439800

H 1.16464800 -3.14391900 -5.64005600

H 0.25236100 -1.71547700 -6.10798500

C 0.92557500 -3.15427900 -8.39828100

H -0.23040700 -4.53904100 -7.20513700

H -1.11646400 -3.10094000 -7.68709200

H 0.67403700 -3.62630200 -9.35479000

H 1.02032600 -2.07568300 -8.57347100

H 1.91037400 -3.52703000 -8.09122300

**(6,5) SWNT-[(CH_2_)_3_CH_3_]_2_ (1,4-L_+_: L_27_)**

C -20.55184900 -1.21884400 -3.46912300

C -19.36514600 -0.43513100 -3.69261100

C -19.40756700 0.96057200 -3.81482500

C -18.25174200 1.73695000 -3.59410700

C -18.10873800 -1.07341000 -3.45775700

C -16.91374100 -0.33855700 -3.68283300

C -15.64550900 -0.98429400 -3.50606700

C -14.44551300 -0.25015500 -3.72132400

C -19.52547800 3.55173900 -2.37217300

C -18.30457200 2.99068100 -2.84993900

C -17.11385400 3.44032600 -2.22382900

C -16.98046400 1.08310900 -3.71088700

C -15.77042000 1.81460900 -3.48782600

C -14.50695400 1.17749900 -3.68439300

C -13.30814200 1.90415100 -3.43280900

C -13.18340500 -0.89787200 -3.55791400

C -11.97879600 -0.14852500 -3.73022200

C -10.71540400 -0.80169400 -3.58928700

C -9.51414800 -0.04638500 -3.72807800

C -19.76190000 3.81883900 1.83324300

C -19.59496900 4.14540000 -1.13320100

C -18.44674300 4.20150000 -0.28793500

C -17.18504000 4.03840800 -0.92289500

C -15.98276200 4.12323900 -0.14809600

C -15.83473600 2.99777500 -2.70688000

C -14.64105300 3.49346600 -2.11118500

C -13.37510200 3.07022900 -2.61398900

C -12.17818000 3.54169300 -1.99303300

C -12.04057000 1.27247900 -3.64167000

C -10.84202900 1.99549800 -3.36751100

C -9.57693800 1.37321500 -3.59394900

C -8.37868400 2.08902700 -3.29396100

C -8.24863200 -0.70229200 -3.60091700

C -7.04756400 0.06008800 -3.71492900

C -5.78472600 -0.59612100 -3.60214200

C -4.58433100 0.16975300 -3.68518000

C -3.25792400 -1.81288000 -3.05244700

C -0.80378700 -1.73269800 -3.10500900

C -19.84246300 2.95361200 2.89662900

C -18.69448800 2.22031600 3.33098300

C -18.52592200 4.02281500 1.14597700

C -17.34314900 3.64997600 1.83944100

C -16.06188700 3.90339800 1.24961500

C -14.87133900 3.57091600 1.96375200

C -14.71309500 4.04877800 -0.79536900

C -13.52473100 4.12384900 -0.01744100

C -12.25215700 4.05581900 -0.66651100

C -11.06082700 4.10964100 0.11653800

C -10.91043100 3.13694300 -2.51167900

C -9.71741800 3.59144500 -1.87839000

C -8.44834300 3.20288100 -2.40715700

C -7.25449400 3.64377300 -1.76145800

C -7.11167100 1.47624500 -3.54003700

C -5.91661800 2.18465000 -3.22090000

C -4.64935300 1.57752800 -3.47818800

C -3.45297100 2.27950300 -3.14271000

C -3.31964300 -0.48969600 -3.58808500

C -2.12515800 0.27210300 -3.65800800

C -0.86360800 -0.39582300 -3.57848800

C 0.33632800 0.38441100 -3.65256700

C 1.67781100 -1.62111800 -3.20590300

C 4.14371500 -1.51611600 -3.31850700

C -20.01888800 0.14638700 3.89185400

C -18.77903000 0.85271900 3.78545200

C -17.59718100 0.06349500 3.75434400

C -17.42687600 2.72590500 2.93153100

C -16.22899600 2.05944800 3.34864300

C -14.95379500 2.61095300 3.01700400

C -13.76777200 1.93405700 3.42126700

C -13.60286300 3.85804100 1.38399200

C -12.41092400 3.48680600 2.07924000

C -11.13868600 3.79559500 1.50742000

C -9.94914300 3.40238300 2.18794000

C -9.79182500 4.06560600 -0.53415100

C -8.59954700 4.09247000 0.24955400

C -7.32896900 4.07471700 -0.40328100

C -6.13696500 4.07955100 0.37970900

C -5.98619600 3.27577600 -2.30280900

C -4.79265200 3.69637100 -1.64356400

C -3.52230200 3.34462500 -2.19615600

C -2.32825300 3.74456100 -1.52679300

C -2.18849700 1.68012000 -3.41895500

C -0.99417000 2.36817400 -3.06526200

C 0.27197400 1.77398000 -3.36562600

C 1.47149500 2.45934100 -3.00341300

C 1.60488300 -0.26921900 -3.64326900

C 2.80063600 0.50793100 -3.69534300

C 4.07037100 -0.14871800 -3.70424600

C 5.26930500 0.63039100 -3.71108900

C 6.60666000 -1.41090200 -3.38979400

C 9.07234000 -1.30234600 -3.44767900

C -20.27563500 -3.24281800 1.41193100

C -20.10207700 -1.18128600 3.55791300

C -18.95255700 -1.88593300 3.07653900

C -17.68409300 -1.31658700 3.37820000

C -16.48735600 -1.97850500 2.95486300

C -16.31430500 0.70087400 3.74113600

C -15.12468400 -0.09061900 3.74736700

C -13.85270500 0.55122600 3.76976500

C -12.66300500 -0.23940200 3.73124700

C -12.49246100 2.49122100 3.09490600

C -11.30206100 1.79634300 3.47266500

C -10.02939700 2.36657900 3.16794700

C -8.83902500 1.65745500 3.51311800

C -8.67728900 3.73015200 1.62612400

C -7.48518500 3.31245800 2.29173200

C -6.21411300 3.66626000 1.74396800

C -5.02247000 3.22267200 2.38990100

C -4.86670600 4.08387500 -0.27348100

C -3.67266700 4.06004400 0.50991000

C -2.40074000 4.08737800 -0.14210400

C -1.20790500 4.03189400 0.63707700

C -1.06052900 3.40498100 -2.08947000

C 0.13697600 3.77601000 -1.40648600

C 1.40482500 3.45614800 -1.98515800

C 2.60098800 3.80170600 -1.29023100

C 2.73519400 1.88758400 -3.33816300

C 3.93511800 2.55092300 -2.93873700

C 5.20209100 1.99316300 -3.29530700

C 6.40009600 2.63884200 -2.86626000

C 6.53565700 -0.02770300 -3.73641900

C 7.73424400 0.74883700 -3.70560200

C 9.00255200 0.09063500 -3.74980800

C 10.19982200 0.86441200 -3.69199800

C 10.33940500 -1.88574600 -3.14330800

C 11.53522500 -1.19392200 -3.49886600

C 12.80494400 -1.78873300 -3.21167100

C 14.00056200 -1.08990500 -3.54541500

C -20.35121900 -3.47521200 0.06433800

C -19.19316000 -3.32658400 -0.76900800

C -19.03524600 -2.86322500 2.02171400

C -17.85027100 -3.16982400 1.29822900

C -16.57083200 -2.90204700 1.88342200

C -15.37787100 -3.24173000 1.17387000

C -15.21121300 -1.44925400 3.32246100

C -14.02546200 -2.10671900 2.88662800

C -12.74985000 -1.58146000 3.26053500

C -11.56105600 -2.22522800 2.79847700

C -11.38678600 0.40362200 3.77050900

C -10.19851000 -0.38520900 3.70458900

C -8.92335000 0.25553300 3.75907100

C -7.73369000 -0.53199200 3.65706500

C -7.56484500 2.23957100 3.22766700

C -6.37368900 1.51762100 3.54187200

C -5.10226600 2.11006400 3.27816900

C -3.91123000 1.37105800 3.55321000

C -3.75025900 3.59665100 1.85495100

C -2.55770000 3.12935900 2.48537100

C -1.28545500 3.52120100 1.96859400

C -0.09650400 3.02680700 2.58181500

C 0.06410400 4.07075700 -0.01443200

C 1.25800300 3.98355500 0.76490100

C 2.52827700 4.04484400 0.11474200

C 3.72005400 3.92845600 0.89018700

C 3.86805300 3.50196600 -1.87899000

C 5.06502300 3.82062300 -1.16862600

C 6.33200100 3.54770000 -1.76771800

C 7.52695400 3.83861600 -1.04458500

C 7.66679800 2.09421100 -3.23976100

C 8.86578900 2.72299400 -2.78286400

C 10.13251700 2.19461700 -3.17666500

C 11.32897000 2.80379300 -2.69365000

C 11.46635300 0.20676900 -3.75383500

C 12.66587000 0.97860100 -3.66512600

C 13.93164100 0.32325700 -3.75048500

C 15.12654400 1.08495000 -3.62056700

C 15.26634900 -1.69170700 -3.27047300

C 16.47287000 -0.98488000 -3.57724300

C 17.74712300 -1.61265200 -3.38026800

C 18.89905100 -0.86180400 -3.69172100

C -20.49990300 -2.33476100 -2.68376100

C -19.26074800 -2.76208000 -2.08675900

C -18.05903300 -2.25946700 -2.66140800

C -17.92758700 -3.39034800 -0.11611500

C -16.72421900 -3.28969000 -0.88235200

C -15.45346500 -3.41568900 -0.23818900

C -14.26042700 -3.30042700 -1.00856000

C -14.10912000 -3.00336800 1.77772400

C -12.91812600 -3.30808900 1.05259500

C -11.64613700 -3.08501600 1.66382200

C -10.45687100 -3.37220600 0.93057100

C -10.28618800 -1.71456000 3.18996200

C -9.09938000 -2.34281600 2.70688300

C -7.82333700 -1.84044800 3.10512400

C -6.63493000 -2.45505000 2.59614400

C -6.45615500 0.10519300 3.72785400

C -5.26946300 -0.67446200 3.57989500

C -3.99261900 -0.04227200 3.66830600

C -2.80208500 -0.82210200 3.49824800

C -2.63747300 1.98138500 3.32613000

C -1.45152200 1.24107800 3.59168700

C -0.17945900 1.85397500 3.38930300

C 1.00854300 1.10221900 3.63505800

C 1.17857100 3.42974400 2.07657600

C 2.36661100 2.91630600 2.67711800

C 3.63984700 3.33330500 2.18414400

C 4.82857500 2.80177100 2.76945600

C 4.99133400 4.01159800 0.24289000

C 6.18235600 3.87291900 1.01556400

C 7.45243900 3.97940100 0.37265500

C 8.64416000 3.81615600 1.14156100

C 8.79610200 3.58852700 -1.65185900

C 9.99034400 3.85680200 -0.91871500

C 11.25836500 3.62856400 -1.53292700

C 12.45357900 3.87097100 -0.78845500

C 12.59840200 2.28710300 -3.10302000

C 13.79429800 2.88013100 -2.59976400

C 15.06058600 2.37221100 -3.01813000

C 16.25868600 2.94500600 -2.48731000

C 16.39838800 0.42973200 -3.71896200

C 17.58870700 1.19311700 -3.58090600

C 18.84877400 0.53827800 -3.73747600

C 20.03077000 1.34976300 -3.60756500

C -16.78978500 -2.69379600 -2.16642300

C -15.58786500 -2.19313500 -2.75825100

C -14.32484700 -2.65753900 -2.28278000

C -13.12471500 -2.13426400 -2.84927800

C -12.99279200 -3.43632900 -0.36503700

C -11.79606700 -3.29260300 -1.13190500

C -10.53001700 -3.45625900 -0.49316600

C -9.33379200 -3.29248700 -1.25247900

C -9.18689600 -3.16984900 1.54651400

C -7.99965100 -3.43850000 0.80284900

C -6.72720300 -3.25847000 1.42424600

C -5.54129800 -3.54258900 0.69104800

C -5.35832400 -1.96088300 2.97905100

C -4.17234200 -2.56690600 2.43394700

C -2.87767600 -2.09019200 2.86381700

C -1.68616300 -2.68334900 2.34719200

C -1.53214900 -0.18453900 3.65730000

C -0.34682100 -0.94636200 3.49337900

C 0.92616900 -0.31932000 3.66842100

C 2.11726600 -1.08320100 3.49106400

C 2.28325300 1.72067600 3.45306300

C 3.46981900 0.96425900 3.68120400

C 4.74489700 1.58705400 3.51162500

C 5.93397500 0.82564700 3.71987100

C 6.10202500 3.23696000 2.29118300

C 7.28985400 2.69149200 2.86111400

C 8.56365400 3.14155800 2.39515900

C 9.75339000 2.58248700 2.95067000

C 9.91513600 3.94890800 0.50411500

C 11.10512000 3.76267100 1.26685900

C 12.37743800 3.91519700 0.63341100

C 13.56962900 3.70752700 1.39096100

C 13.72359200 3.66764500 -1.41020000

C 14.91490300 3.88066900 -0.65866000

C 16.18762500 3.68641300 -1.28186300

C 17.38955400 3.88311300 -0.53217200

C 17.53107400 2.46400900 -2.92864900

C 18.72905100 3.03662200 -2.41545800

C 19.97137700 2.54984000 -2.95837500

C -11.85920500 -2.60946600 -2.38155100

C -10.65667000 -2.06782400 -2.92842500

C -9.39517400 -2.55904600 -2.47700100

C -8.18949400 -1.98942100 -2.99045600

C -8.06991600 -3.48242600 -0.61801700

C -6.86351500 -3.29745000 -1.36704800

C -5.60990300 -3.53148400 -0.74543800

C -4.39644900 -3.27380900 -1.46240200

C -4.27667700 -3.38593500 1.30924500

C -3.06984800 -3.99758500 0.60805000

C -1.73686300 -3.50130600 1.16950000

C -0.57464300 -3.69015200 0.43115900

C -0.42440800 -2.19365600 2.80024100

C 0.77207700 -2.75090300 2.26900100

C 2.04297600 -2.30817100 2.76254100

C 3.23531600 -2.85697400 2.21759700

C 3.38812600 -0.46117500 3.68091000

C 4.58034200 -1.21820500 3.47334600

C 5.85350400 -0.59919200 3.67853500

C 7.04533400 -1.34635200 3.44610900

C 7.20692000 1.45484000 3.56867400

C 8.39764500 0.68841200 3.75133600

C 9.67158300 1.32387300 3.62057900

C 10.86104700 0.55393500 3.77870400

C 11.02480400 3.04818200 2.49943600

C 12.21577900 2.46993700 3.03536900

C 13.48957100 2.95221200 2.60080300

C 14.67712300 2.35800300 3.11396900

C 14.83811300 3.87714700 0.76390400

C 16.03194700 3.63090100 1.50981800

C 17.31124000 3.83216100 0.89800100

C 18.49654300 3.62031800 1.65391900

C 18.65649300 3.74957900 -1.17190800

C 19.81278800 3.99973600 -0.36100800

C -6.92600000 -2.49816500 -2.55488400

C -5.72629800 -1.90129900 -3.02870900

C -4.46151800 -2.41615000 -2.59295300

C -3.12848200 -3.59405700 -0.88754500

C -1.96346600 -3.32846400 -1.59773500

C -0.63702400 -3.90596700 -1.10231600

C 0.57771000 -3.22859500 -1.72433600

C 0.69709500 -3.46046900 1.04016200

C 1.90751800 -3.64340500 0.29589400

C 3.16344100 -3.50319600 0.94056200

C 4.36810700 -3.60983500 0.17270300

C 4.50522500 -2.42019800 2.71000900

C 5.70357000 -2.93827700 2.13043400

C 6.97039200 -2.52048300 2.63590000

C 8.16718700 -3.00737900 2.02626900

C 8.31786300 -0.73233200 3.66504100

C 9.51218500 -1.47102000 3.40395100

C 10.78193200 -0.86545300 3.64397900

C 11.97564600 -1.59290700 3.34937200

C 12.13455900 1.19372200 3.66322400

C 13.32740300 0.41841700 3.79611600

C 14.59618500 1.05954800 3.69945400

C 15.78896300 0.27830300 3.78847300

C 15.95142100 2.84414500 2.68534200

C 17.15045900 2.24410600 3.18735900

C 18.41675700 2.77803900 2.81960600

C 19.56885300 2.14173800 3.38300400

C 19.73628200 3.92858900 1.00445600

C -2.00287400 -2.37024700 -2.66448500

C 0.48448500 -2.27506100 -2.73891700

C 1.83967100 -3.47607300 -1.13149200

C 3.02905300 -3.11840500 -1.82541600

C 4.29906800 -3.38969000 -1.23209100

C 5.48947900 -3.04663300 -1.93858400

C 5.63353600 -3.51558500 0.82455800

C 6.82744700 -3.59821800 0.04930100

C 8.09575300 -3.53009200 0.70214000

C 9.29053400 -3.59407000 -0.07679600

C 9.43734100 -2.61215100 2.54841100

C 10.63019200 -3.07781400 1.92164100

C 11.90011500 -2.69890000 2.45503600

C 13.09586400 -3.14405700 1.81073900

C 13.24869700 -0.99612100 3.61131300

C 14.43798700 -1.70989800 3.29065000

C 15.71012000 -1.11745500 3.55797900

C 16.91171800 -1.82292200 3.22403100

C 17.06912100 0.91905600 3.72738900

C 18.25407700 0.14451100 3.85366200

C 19.49098200 0.86354100 3.87414900

C 2.94753300 -2.17480900 -2.88862300

C 5.41298800 -2.08030300 -2.98822800

C 6.75587500 -3.33792900 -1.35290400

C 7.94945300 -2.97621400 -2.04655100

C 9.21807400 -3.28971100 -1.46990800

C 10.41089600 -2.91262200 -2.15251900

C 10.55753900 -3.55441300 0.57683100

C 11.75101200 -3.59024600 -0.20426100

C 13.02242000 -3.57378700 0.45049700

C 14.21272200 -3.58738500 -0.32817200

C 14.36186600 -2.78588500 2.35507700

C 15.55566300 -3.19390500 1.68733500

C 16.83459500 -2.86766500 2.24609300

C 18.02075700 -3.31373400 1.60382500

C 18.17609600 -1.26812700 3.56410700

C 19.32800600 -2.04231100 3.22121900

C 7.87589100 -1.98221200 -3.06604100

C 11.67859500 -3.24293200 -1.58416700

C 12.87527800 -2.84563600 -2.25605600

C 14.14167100 -3.19660500 -1.70206800

C 15.33454100 -2.77071700 -2.35116400

C 15.48105900 -3.57753600 0.32523900

C 16.68526900 -3.57972400 -0.45158000

C 17.94618900 -3.65941600 0.20033900

C 19.09645900 -3.69708200 -0.64280500

C 19.25376900 -3.02625800 2.26585700

C 16.61461400 -3.14348000 -1.81516900

C 17.80472500 -2.76632500 -2.48899500

C 19.02729900 -3.25768400 -1.94454200

H -21.50957100 -0.83869300 -3.81581000

H -20.38435700 1.43441600 -3.82339800

H -20.44603400 3.39519900 -2.92608200

H -20.67742900 4.24776700 1.43768900

H -20.56737800 4.43637300 -0.74858000

H -20.81960800 2.72272500 3.30885900

H -20.93201300 0.68849300 4.11733100

H -21.19113200 -3.22763400 1.99524700

H -21.07901200 -1.65309100 3.52473600

H -21.32485400 -3.63785000 -0.38682700

H 19.87873200 -1.32699400 -3.64172500

H -21.42445000 -2.83677500 -2.41619000

H 20.99107500 0.93734900 -3.90697900

H 20.89258600 3.08568000 -2.75179400

H 20.78637700 4.11140600 -0.82748100

H 20.54393100 2.60993100 3.29314100

H 20.65095400 3.98638100 1.58635300

H 20.40626700 0.35565800 4.16213300

H 20.30306300 -1.76077200 3.60599600

H 20.06959100 -3.93180100 -0.22313100

H 20.17205800 -3.49479000 1.92552700

H 19.94866700 -3.16221300 -2.51082000

C -3.19191600 -5.55797200 0.84104300

H -2.31778500 -6.04064600 0.40078200

H -4.07055100 -5.90640600 0.28362100

C -0.53530800 -5.42772500 -1.52218600

H -1.41643000 -5.94753300 -1.14277800

H 0.33793900 -5.85254100 -1.01133000

C -0.43237600 -5.69991200 -3.02970500

C -0.42410300 -7.20284600 -3.34451300

H 0.47826600 -5.24313100 -3.43601200

H -1.27595300 -5.22703200 -3.55076100

C -0.30898700 -7.49853100 -4.84341200

H 0.40992500 -7.68126700 -2.81166300

H -1.34135200 -7.66301600 -2.95049400

H -0.30915100 -8.57698000 -5.03790300

H -1.14622200 -7.05915700 -5.39931800

H 0.61766500 -7.08479300 -5.25941100

C -3.29897900 -6.00820200 2.30478700

C -3.32878900 -7.53788700 2.43569100

H -4.20307100 -5.59160800 2.76545600

H -2.44879100 -5.61334800 2.87737000

C -3.45414400 -8.01091300 3.88764100

H -4.16758300 -7.93620000 1.84728100

H -2.41677300 -7.96049900 1.99071400

H -3.47125300 -9.10483000 3.95044500

H -2.61276000 -7.65499500 4.49467200

H -4.37618300 -7.63585100 4.34818900

**(6,5) SWNT-[(CH_2_)_3_CH_3_]_2_ (1,4-L–: L_–33_)**

C -20.56271200 -1.37989300 -3.44416400

C -19.37727100 -0.59514800 -3.67021700

C -19.42115200 0.80061800 -3.79342700

C -18.26565100 1.57820600 -3.57608600

C -18.11991800 -1.23199100 -3.43724600

C -16.92614800 -0.49595700 -3.66524600

C -15.65690300 -1.14010600 -3.49031900

C -14.45867600 -0.40497300 -3.70957100

C -19.53793000 3.39256300 -2.35228400

C -18.31776300 2.83234800 -2.83219600

C -17.12617700 3.28202700 -2.20757000

C -16.99407900 0.92543800 -3.69494700

C -15.78403500 1.65842100 -3.47547100

C -14.52082500 1.02263200 -3.67487500

C -13.32163500 1.75048400 -3.42594900

C -13.19549400 -1.05098100 -3.54707100

C -11.99205900 -0.30092300 -3.72635500

C -10.72882200 -0.95263400 -3.58745400

C -9.52730700 -0.19661700 -3.73360600

C -19.76897400 3.65310800 1.85332000

C -19.60573400 3.98492600 -1.11243100

C -18.45651000 4.03997300 -0.26882100

C -17.19569700 3.87789300 -0.90567100

C -15.99212500 3.96132700 -0.13239900

C -15.84748400 2.84115800 -2.69349700

C -14.65305700 3.33633000 -2.09914100

C -13.38761500 2.91448000 -2.60476400

C -12.18905500 3.38419400 -1.98435900

C -12.05387700 1.12044700 -3.63922300

C -10.85485100 1.84320800 -3.36668600

C -9.58947700 1.22144200 -3.59710600

C -8.38866100 1.93518800 -3.29630100

C -8.26126800 -0.85246800 -3.61334400

C -7.05958600 -0.09180800 -3.73046100

C -5.79518800 -0.75112500 -3.62350600

C -4.58942000 0.01334300 -3.70272300

C -3.26907400 -1.96903300 -3.07451800

C -0.78451900 -1.88680400 -3.08954400

C -19.84815700 2.78666900 2.91604700

C -18.69956900 2.05409700 3.34914500

C -18.53395300 3.85862200 1.16520900

C -17.35040100 3.48477000 1.85646000

C -16.06965700 3.73901200 1.26522200

C -14.87855100 3.40653500 1.97753000

C -14.72353700 3.88830700 -0.78150600

C -13.53411200 3.96078000 -0.00498800

C -12.26185800 3.89385000 -0.65576100

C -11.07009900 3.94559100 0.12581800

C -10.92219100 2.98118200 -2.50601400

C -9.72762400 3.43098900 -1.87114000

C -8.45807100 3.04381000 -2.40234900

C -7.26405800 3.47937500 -1.75472500

C -7.12242100 1.32265900 -3.54648100

C -5.92435600 2.02631200 -3.21969500

C -4.65498800 1.41993100 -3.48031500

C -3.45913800 2.11680900 -3.13472600

C -3.32585800 -0.64733700 -3.60106300

C -2.12354400 0.11589100 -3.64590600

C -0.85504900 -0.55176000 -3.55859000

C 0.34025900 0.23087200 -3.62704700

C 1.66906700 -1.77685100 -3.14441900

C 4.13349300 -1.67032800 -3.24749900

C -20.02192400 -0.02088600 3.91200900

C -18.78281300 0.68633500 3.80463400

C -17.60053900 -0.10188400 3.77347300

C -17.43271700 2.56017600 2.94817100

C -16.23404400 1.89465000 3.36471100

C -14.95962600 2.44675400 3.03196400

C -13.77360000 1.77113700 3.43618000

C -13.61106000 3.69322400 1.39597800

C -12.41817800 3.32246100 2.09038700

C -11.14725800 3.63121100 1.51751200

C -9.95804700 3.23862700 2.19787500

C -9.80181400 3.90016700 -0.52585400

C -8.60858300 3.92569600 0.25774700

C -7.33931300 3.90749400 -0.39477800

C -6.14833300 3.91084700 0.38937000

C -5.99558300 3.10951100 -2.29527700

C -4.80107600 3.52882300 -1.63290900

C -3.53168100 3.17966700 -2.18401000

C -2.33994300 3.58081700 -1.50927300

C -2.19182900 1.51457900 -3.40106900

C -0.99551500 2.21238800 -3.04337200

C 0.27002000 1.62857900 -3.34445900

C 1.46369500 2.31309300 -2.97617500

C 1.59934400 -0.42118300 -3.59465100

C 2.79564500 0.35583700 -3.63847500

C 4.06193000 -0.30259800 -3.64205600

C 5.25774700 0.47430100 -3.65755500

C 6.59388100 -1.56849300 -3.34203300

C 9.05955100 -1.45858700 -3.41388000

C -20.27704600 -3.41097100 1.43415300

C -20.10407100 -1.34904400 3.57924600

C -18.95420100 -2.05298500 3.09804500

C -17.68625300 -1.48234900 3.39858000

C -16.48900300 -2.14320200 2.97440600

C -16.31803800 0.53651200 3.75898100

C -15.12800600 -0.25391000 3.76633900

C -13.85705300 0.38895200 3.78744200

C -12.66647800 -0.40095800 3.74977700

C -12.49845600 2.32856300 3.10811200

C -11.30824500 1.63665200 3.48848900

C -10.03744000 2.20636300 3.18108300

C -8.84580400 1.49968800 3.53048100

C -8.68617900 3.56548700 1.63506500

C -7.49519900 3.15276800 2.30336900

C -6.22605200 3.50269000 1.75390000

C -5.03325200 3.06503400 2.40580700

C -4.87725900 3.91547500 -0.26263700

C -3.68547800 3.89840900 0.52223600

C -2.41591600 3.92498300 -0.12847600

C -1.22091800 3.87737000 0.65580800

C -1.06834900 3.24689800 -2.07037800

C 0.12697600 3.63163100 -1.38822400

C 1.39307700 3.31654500 -1.96176000

C 2.58991900 3.66422100 -1.26398600

C 2.72846000 1.73671300 -3.29803400

C 3.92770300 2.40510100 -2.90370300

C 5.19087000 1.84314400 -3.25450600

C 6.38914200 2.49020000 -2.82929900

C 6.52327700 -0.18468600 -3.68215900

C 7.72169500 0.59353900 -3.66033800

C 8.98855000 -0.06280500 -3.71007100

C 10.18569500 0.71268200 -3.65534100

C 10.33124900 -2.03924700 -3.11439700

C 11.52477100 -1.34424900 -3.47117800

C 12.79658500 -1.93594100 -3.18669600

C 13.99063600 -1.23412400 -3.52146300

C -20.35393700 -3.64137200 0.08626000

C -19.19718900 -3.48939600 -0.74812500

C -19.03653700 -3.03031400 2.04284800

C -17.85206800 -3.33415100 1.31774200

C -16.57211600 -3.06547900 1.90206600

C -15.37923800 -3.40228500 1.19103900

C -15.21313400 -1.61338200 3.34197000

C -14.02734500 -2.26897800 2.90502800

C -12.75154500 -1.74265500 3.27917000

C -11.56179700 -2.38448000 2.81708800

C -11.39084700 0.24294400 3.79024600

C -10.20339900 -0.54399000 3.72551200

C -8.92746800 0.09832900 3.78094800

C -7.73725900 -0.68640400 3.68887300

C -7.57389600 2.08328400 3.24646100

C -6.38393700 1.36578500 3.56812100

C -5.11189700 1.96099100 3.30456100

C -3.92027200 1.23349100 3.59963300

C -3.76333300 3.44037300 1.87244600

C -2.57193700 2.97870600 2.50588500

C -1.29982200 3.37154300 1.98513000

C -0.10685000 2.88676900 2.60301200

C 0.05059900 3.92928500 0.00659700

C 1.24295000 3.84938300 0.78585400

C 2.51464500 3.91043400 0.13739900

C 3.70921900 3.79785600 0.91539900

C 3.85840200 3.36514900 -1.85124000

C 5.05391200 3.68698900 -1.14253200

C 6.32063700 3.40729500 -1.73886300

C 7.51644900 3.70158600 -1.01714600

C 7.65456700 1.94214700 -3.20100100

C 8.85248700 2.57381100 -2.75012600

C 10.11840500 2.04347800 -3.14279500

C 11.31515700 2.65670300 -2.66501500

C 11.45336600 0.05772200 -3.72303100

C 12.65126000 0.83139800 -3.63701300

C 13.91870100 0.17879600 -3.72532800

C 15.11247500 0.94290800 -3.59830400

C 15.25836500 -1.83336300 -3.24890800

C 16.46274900 -1.12419600 -3.55751200

C 17.73890500 -1.74922800 -3.36212000

C 18.88859400 -0.99655100 -3.67670300

C -20.50812100 -2.49617500 -2.65941000

C -19.26749000 -2.92269600 -2.06502800

C -18.06746700 -2.41787000 -2.64076500

C -17.93078200 -3.55210600 -0.09689600

C -16.72838200 -3.44767000 -0.86426700

C -15.45641600 -3.57279200 -0.22175200

C -14.26494300 -3.45331600 -0.99276700

C -14.11031800 -3.16328000 1.79422300

C -12.91887800 -3.46295300 1.06693500

C -11.64560600 -3.24065400 1.67834300

C -10.45699700 -3.52040100 0.94393000

C -10.28829200 -1.87311600 3.20962700

C -9.09931800 -2.49710200 2.72388700

C -7.82150300 -1.99946400 3.13140100

C -6.63579400 -2.61034300 2.62927700

C -6.46490000 -0.04566800 3.76449800

C -5.27329400 -0.82577400 3.63673900

C -4.00076700 -0.18650900 3.73750000

C -2.81260400 -0.95796500 3.57432300

C -2.65152200 1.83863800 3.35926500

C -1.45859500 1.09639000 3.61894000

C -0.18788800 1.71068200 3.40971800

C 0.99925600 0.95809700 3.64049400

C 1.16266500 3.29374500 2.09903300

C 2.35419600 2.77554900 2.69422200

C 3.62911900 3.19893100 2.20591700

C 4.81714800 2.66548500 2.79046300

C 4.97946300 3.88303200 0.26963600

C 6.17109900 3.74143000 1.04246300

C 7.44195200 3.84721500 0.39940200

C 8.63350000 3.68388700 1.16790200

C 8.78360200 3.44734800 -1.62405300

C 9.97833500 3.71751000 -0.89284700

C 11.24551600 3.48661300 -1.50775100

C 12.44083300 3.73231000 -0.76572700

C 12.58347300 2.14079700 -3.07567100

C 13.77957200 2.73659300 -2.57571500

C 15.04581100 2.23048100 -2.99601400

C 16.24366200 2.80579400 -2.46791700

C 16.38530300 0.29031500 -3.69922300

C 17.57464000 1.05593600 -3.56329900

C 18.83558900 0.40352500 -3.72263000

C 20.01630300 1.21693900 -3.59476400

C -16.79677600 -2.85043400 -2.14737600

C -15.59652100 -2.34815700 -2.74095000

C -14.33248600 -2.80995500 -2.26657000

C -13.13427900 -2.28452400 -2.83518600

C -12.99567200 -3.58680500 -0.35024500

C -11.80019200 -3.43916700 -1.11781000

C -10.53373200 -3.59839600 -0.48004100

C -9.33966600 -3.42446600 -1.23962100

C -9.18438700 -3.31652500 1.56087200

C -7.99379900 -3.57789100 0.81810700

C -6.72168600 -3.40163500 1.44204500

C -5.53464800 -3.64338400 0.69090000

C -5.35691300 -2.11815800 3.03958700

C -4.16793000 -2.71437500 2.51724600

C -2.89685100 -2.22555100 2.92700500

C -1.70566800 -2.79618100 2.36874500

C -1.53920800 -0.32298700 3.69348800

C -0.35009900 -1.08915000 3.50198300

C 0.91557200 -0.46692100 3.65454600

C 2.10384300 -1.23338200 3.43714300

C 2.27086400 1.57595700 3.45328900

C 3.46042500 0.81242200 3.67060200

C 4.73392000 1.44217800 3.52095400

C 5.92357400 0.68194600 3.72710900

C 6.09058100 3.10241700 2.31540900

C 7.28020000 2.55369200 2.88412900

C 8.55349000 3.00719300 2.42120500

C 9.74389700 2.44707500 2.97462400

C 9.90385600 3.81438500 0.52928500

C 11.09491500 3.62888300 1.29152400

C 12.36604700 3.78093400 0.65645800

C 13.55903800 3.57476300 1.41283500

C 13.70984500 3.52714500 -1.38834800

C 14.90196500 3.74356000 -0.63885500

C 16.17359000 3.54914900 -1.26335200

C 17.37630800 3.74848900 -0.51559000

C 17.51615500 2.32659700 -2.91089100

C 18.71410600 2.90155200 -2.40005800

C 19.95610100 2.41683000 -2.94508400

C -11.86700100 -2.75648200 -2.36844200

C -10.66876700 -2.21380700 -2.91921000

C -9.40448800 -2.69924600 -2.46546400

C -8.20260600 -2.13607700 -2.99405600

C -8.07176800 -3.60803300 -0.60636800

C -6.87910100 -3.41403100 -1.35888400

C -5.61554800 -3.62295600 -0.72996100

C -4.41931800 -3.40317600 -1.47433200

C -4.25166500 -3.48906900 1.31404900

C -3.07443500 -3.73114200 0.56321000

C -1.78525800 -3.55397200 1.16862300

C -0.59278300 -3.85299700 0.44469900

C -0.43232000 -2.33469600 2.80326600

C 0.74824000 -2.91970800 2.25731400

C 2.02089600 -2.45556500 2.72452500

C 3.23796400 -3.00127500 2.17498600

C 3.37873000 -0.60638200 3.64626500

C 4.57409900 -1.35818000 3.45557600

C 5.84445700 -0.74092200 3.67834800

C 7.04234700 -1.48696900 3.45435900

C 7.19810000 1.31466000 3.58492400

C 8.39014200 0.54998900 3.76944300

C 9.66327600 1.18718500 3.64138200

C 10.85518600 0.41830300 3.79890000

C 11.01555900 2.91467100 2.52384300

C 12.20740400 2.33758300 3.05909700

C 13.48042500 2.82094900 2.62353200

C 14.66941500 2.22820200 3.13604700

C 14.82674600 3.74385200 0.78375400

C 16.02143200 3.50029300 1.52873400

C 17.29973800 3.70088300 0.91472600

C 18.48619300 3.49205600 1.66983600

C 18.64252900 3.61545300 -1.15681500

C 19.79947500 3.86790200 -0.34779400

C -6.94035700 -2.64159900 -2.55940300

C -5.73921400 -2.05530200 -3.05616800

C -4.47464900 -2.57613300 -2.63077300

C -3.15775900 -3.67147600 -0.87059800

C -1.96926900 -3.42721300 -1.59607100

C -0.68484700 -4.06619700 -1.09124800

C 0.57556500 -3.44463600 -1.69960400

C 0.64575700 -3.70855500 1.06700700

C 1.90041300 -4.21767000 0.35098500

C 3.19132300 -3.69884700 0.96578000

C 4.37764000 -3.81323500 0.20545200

C 4.50473200 -2.55648800 2.69027800

C 5.70502600 -3.08146100 2.13899700

C 6.97396500 -2.66087600 2.65169200

C 8.16963000 -3.15439600 2.05123900

C 8.31338800 -0.87136600 3.68094500

C 9.50888400 -1.60810200 3.42227500

C 10.77890800 -1.00003100 3.66328200

C 11.97383400 -1.72644400 3.37127100

C 12.12794500 1.06038300 3.68577700

C 13.32208500 0.28687700 3.81792100

C 14.59044400 0.92996200 3.72150100

C 15.78449700 0.15056100 3.81059300

C 15.94272600 2.71567600 2.70597800

C 17.14292900 2.11779000 3.20754700

C 18.40833400 2.65271900 2.83772600

C 19.56160000 2.01916100 3.40178500

C 19.72475300 3.79959500 1.01803400

C -2.00764900 -2.51394600 -2.65162800

C 0.48240800 -2.44344600 -2.71894600

C 1.81186700 -3.71775300 -1.11757700

C 3.00786500 -3.29897700 -1.77312200

C 4.29445800 -3.60016000 -1.21354800

C 5.47473300 -3.23194300 -1.90722800

C 5.64218800 -3.67586600 0.84733000

C 6.83592700 -3.76368000 0.07325500

C 8.10112300 -3.68704700 0.72847500

C 9.29317400 -3.74898400 -0.04958400

C 9.43783300 -2.75190000 2.57103600

C 10.63239600 -3.21793200 1.94607500

C 11.90098200 -2.83473700 2.47879400

C 13.09703800 -3.27891700 1.83536000

C 13.24581700 -1.12753500 3.63276400

C 14.43678000 -1.83976500 3.31248100

C 15.70797300 -1.24541700 3.57992800

C 16.91067600 -1.94895400 3.24578300

C 17.06368200 0.79321000 3.74889100

C 18.25001700 0.02053300 3.87503500

C 19.48580900 0.74156700 3.89474300

C 2.93689200 -2.32529500 -2.80689600

C 5.39941300 -2.24141100 -2.94086500

C 6.75535600 -3.51460700 -1.32685500

C 7.94426000 -3.14576100 -2.02059400

C 9.21571700 -3.45000600 -1.44343100

C 10.40742100 -3.06711600 -2.12621900

C 10.56112500 -3.70094000 0.60434200

C 11.75278000 -3.73698400 -0.17723300

C 13.02408700 -3.71349400 0.47672800

C 14.21371200 -3.72599500 -0.30290400

C 14.36277500 -2.91667200 2.37840200

C 15.55688300 -3.32400600 1.71097000

C 16.83531600 -2.99425800 2.26835200

C 18.02212500 -3.43862300 1.62572600

C 18.17433000 -1.39204600 3.58554400

C 19.32750700 -2.16424300 3.24232600

C 7.86679500 -2.14280500 -3.03510200

C 11.67699500 -3.39338200 -1.55864200

C 12.87124500 -2.99273600 -2.23115500

C 14.13983400 -3.33914400 -1.67747000

C 15.33057900 -2.91155900 -2.32853400

C 15.48231300 -3.71108600 0.34968300

C 16.68556400 -3.71197500 -0.42809000

C 17.94738700 -3.78670900 0.22296100

C 19.09684200 -3.82335200 -0.62128900

C 19.25482900 -3.14843000 2.28710300

C 16.61215800 -3.27963000 -1.79280000

C 17.80059500 -2.90097300 -2.46870900

C 19.02503800 -3.38762200 -1.92415700

H -21.52153300 -1.00056500 -3.78871100

H -20.39841500 1.27352600 -3.80000400

H -20.45933700 3.23595100 -2.90476700

H -20.68520500 4.08161500 1.45895400

H -20.57771800 4.27494800 -0.72602900

H -20.82491000 2.55472600 3.32861900

H -20.93553100 0.52060100 4.13701500

H -21.19181700 -3.39809600 2.01866400

H -21.08068000 -1.82153900 3.54641600

H -21.32785200 -3.80494100 -0.36395400

H 19.86928600 -1.45977600 -3.62828200

H -21.43169900 -2.99914300 -2.39032600

H 20.97679800 0.80612700 -3.89577400

H 20.87685900 2.95400200 -2.73993800

H 20.77250500 3.97896400 -0.81558000

H 20.53591600 2.48877000 3.31095900

H 20.64024700 3.85883300 1.59849700

H 20.40191900 0.23543200 4.18318800

H 20.30216600 -1.88083600 3.62673200

H 20.07112200 -4.05410000 -0.20206500

H 20.17388200 -3.61534600 1.94659600

H 19.94561400 -3.29088500 -2.49150800

C 2.00115300 -5.79169800 0.49702500

H 2.78150100 -6.13859100 -0.19234300

H 1.05922300 -6.23098300 0.17020600

C -0.80053200 -5.58327800 -1.53105200

H -1.57879300 -6.04861600 -0.91282900

H 0.14051800 -6.08105900 -1.30019100

C -1.10413000 -5.83085200 -3.01632300

C -1.04716700 -7.32416800 -3.37032900

H -0.38156000 -5.28483500 -3.63834200

H -2.09462300 -5.43927400 -3.27580200

C -1.36796500 -7.60129000 -4.84275200

H -0.04867800 -7.71772100 -3.13270600

H -1.75186900 -7.87602500 -2.73205500

H -1.31740400 -8.67276300 -5.06696700

H -2.37552900 -7.25360300 -5.10111600

H -0.66081200 -7.08789600 -5.50562800

C 2.28953900 -6.31861500 1.91087000

C 2.22337600 -7.85167800 1.97442800

H 1.56281200 -5.89651100 2.61855200

H 3.27903800 -5.98972800 2.24983900

C 2.52249500 -8.40518900 3.37133500

H 1.22712500 -8.18805800 1.65385600

H 2.93512600 -8.27618200 1.25209500

H 2.46675400 -9.49950500 3.38663100

H 3.52685000 -8.11766700 3.70545600

H 1.80667000 -8.02426600 4.10994500

**CF_3_(CF_2_)_3_-(6,5) SWNT-H (1,2-L_++_: L_87_)**

C -20.67431500 -0.67263900 -2.99446000

C -19.48822000 0.10801600 -3.23135700

C -19.53123300 1.50178100 -3.37509300

C -18.37543300 2.28196300 -3.16835900

C -18.23133100 -0.52605700 -2.98823800

C -17.03700000 0.20587300 -3.22608100

C -15.76826700 -0.43648400 -3.04098300

C -14.56924800 0.29467300 -3.26958000

C -19.64805900 4.11522900 -1.97323300

C -18.42762400 3.54704600 -2.44345700

C -17.23642800 4.00573700 -1.82477800

C -17.10413800 1.62677000 -3.27643300

C -15.89394400 2.36215300 -3.06639700

C -14.63072300 1.72264000 -3.25484500

C -13.43155900 2.45329200 -3.01532800

C -13.30667800 -0.34991500 -3.09746400

C -12.10263500 0.39686400 -3.28495900

C -10.83954300 -0.25379900 -3.13627300

C -9.63792100 0.49943200 -3.29015700

C -19.88225000 4.43783400 2.22755400

C -19.71665500 4.72616000 -0.74256100

C -18.56804100 4.79329700 0.10118200

C -17.30676300 4.62156800 -0.53216000

C -16.10390900 4.71672500 0.24068700

C -15.95754200 3.55659000 -2.30256500

C -14.76339600 4.06055500 -1.71478800

C -13.49774400 3.63020300 -2.21247800

C -12.29988300 4.10951500 -1.59853400

C -12.16399000 1.81912700 -3.21708300

C -10.96497300 2.54541000 -2.95401100

C -9.69992400 1.91966500 -3.17340900

C -8.50006700 2.63778300 -2.88248000

C -8.37229400 -0.15530000 -3.15852600

C -7.17049800 0.60374700 -3.28356100

C -5.90682400 -0.05373300 -3.16559300

C -4.70397800 0.71048900 -3.25497600

C -3.38083400 -1.26349800 -2.60029600

C -0.91147500 -1.18335200 -2.63886000

C -19.96214400 3.58719800 3.30279200

C -18.81370900 2.86116700 3.74753100

C -18.64659700 4.63326500 1.53746500

C -17.46351000 4.27002900 2.23520300

C -16.18243000 4.51571900 1.64131600

C -14.99162100 4.19390700 2.35943900

C -14.83467300 4.63349700 -0.40629000

C -13.64590300 4.71863800 0.36990400

C -12.37331000 4.64113700 -0.27880900

C -11.18177700 4.70575300 0.50284000

C -11.03254800 3.69735900 -2.11227200

C -9.83874400 4.15831200 -1.48463400

C -8.56937300 3.76148100 -2.00809200

C -7.37535300 4.20904800 -1.36786400

C -7.23359800 2.02102100 -3.12167200

C -6.03708200 2.73109200 -2.80813300

C -4.76894100 2.12022900 -3.05819000

C -3.57279800 2.82439300 -2.72743200

C -3.43962100 0.05198000 -3.14526800

C -2.24141400 0.81453000 -3.21454200

C -0.97688700 0.14731000 -3.12160900

C 0.22028000 0.92869500 -3.20287700

C 1.55406600 -1.07033600 -2.70608500

C 4.01893400 -0.97327400 -2.82599300

C -20.13649800 0.79496900 4.34104800

C -18.89724000 1.50046100 4.22311300

C -17.71499800 0.71169000 4.20385900

C -17.54657100 3.36147200 3.34020700

C -16.34818700 2.70205400 3.76697600

C -15.07344900 3.24931500 3.42699600

C -13.88745500 2.57947800 3.84132000

C -13.72367000 4.47265600 1.77489600

C -12.53130500 4.11212400 2.47535800

C -11.25954400 4.41269200 1.89882000

C -10.07046700 4.03038300 2.58547400

C -9.91309800 4.65054900 -0.14730900

C -8.72034500 4.68844100 0.63625700

C -7.45012700 4.65976900 -0.01568900

C -6.25889700 4.67578400 0.76798000

C -6.10723800 3.83119000 -1.90270200

C -4.91312900 4.26082600 -1.24799600

C -3.64323300 3.90203000 -1.79467300

C -2.45034200 4.31298500 -1.12923900

C -2.30698400 2.21998000 -2.99065700

C -1.11181800 2.91906500 -2.64538700

C 0.15416300 2.32418000 -2.93734000

C 1.35175500 3.01437000 -2.58363400

C 1.48385400 0.27467100 -3.16980000

C 2.68054300 1.04909900 -3.23301700

C 3.94745100 0.38984100 -3.23108000

C 5.14569700 1.16709000 -3.25423100

C 6.48129800 -0.87113300 -2.90841400

C 8.94687800 -0.76439800 -2.97394900

C -20.39194500 -2.63122200 1.91352700

C -20.21876200 -0.53790200 4.02795600

C -19.06893100 -1.24906100 3.55717600

C -17.80088900 -0.67416900 3.84928900

C -16.60383000 -1.34156100 3.43522100

C -16.43250900 1.34967100 4.18026600

C -15.24248200 0.55917800 4.19879800

C -13.97135200 1.20207100 4.21106400

C -12.78110900 0.41161000 4.18425900

C -12.61234500 3.13228200 3.50618400

C -11.42193100 2.44495700 3.89586000

C -10.15060300 3.01081600 3.58219500

C -8.95958300 2.30818300 3.94023200

C -8.79829500 4.34868200 2.01836500

C -7.60669100 3.94443200 2.69251000

C -6.33661600 4.28702100 2.13882100

C -5.14429300 3.85645600 2.79528300

C -4.98799000 4.66868100 0.11562100

C -3.79450600 4.66083000 0.90005800

C -2.52425800 4.67731800 0.24800500

C -1.32985000 4.63547700 1.03031800

C -1.18074100 3.96650500 -1.68612300

C 0.01707800 4.35439700 -1.00889700

C 1.28441000 4.02888300 -1.58230300

C 2.48125800 4.38541700 -0.89264900

C 2.61530800 2.43472600 -2.90498500

C 3.81551900 3.10517700 -2.51860600

C 5.08007300 2.53840400 -2.86450600

C 6.27883200 3.19043800 -2.44831600

C 6.41111000 0.50748200 -3.27135900

C 7.61012900 1.28411200 -3.25645900

C 8.87736600 0.62499600 -3.29412700

C 10.07510600 1.39925600 -3.25010500

C 10.21513600 -1.34327600 -2.66388700

C 11.41041600 -0.65643200 -3.03099800

C 12.68059100 -1.24700400 -2.73755100

C 13.87582900 -0.55295600 -3.08292100

C -20.46852600 -2.88168900 0.56925700

C -19.31134700 -2.74278300 -0.26691200

C -19.15136600 -2.24199100 2.51677000

C -17.96679000 -2.55740900 1.79672900

C -16.68699800 -2.28030500 2.37718600

C -15.49411000 -2.62865100 1.67163200

C -15.32788200 -0.80614200 3.79442100

C -14.14211900 -1.46880400 3.36773200

C -12.86655500 -0.93686700 3.73333100

C -11.67701400 -1.58608800 3.28068000

C -11.50536200 1.05594400 4.21523900

C -10.31771300 0.26778600 4.16148000

C -9.04263700 0.91056100 4.20785000

C -7.85214500 0.12341500 4.12416300

C -7.68630200 2.88766400 3.64852400

C -6.49638700 2.17388400 3.97943700

C -5.22423400 2.76395000 3.70702000

C -4.03203400 2.03620300 4.00678700

C -3.87222000 4.22208000 2.25522800

C -2.68035500 3.76746600 2.89390000

C -1.40835100 4.14797700 2.36590400

C -0.21495300 3.66397500 2.98741000

C -0.05656800 4.66977600 0.37788700

C 1.13790900 4.59915100 1.15800900

C 2.40838000 4.65108500 0.50723900

C 3.60180700 4.54650900 1.28468400

C 3.74861800 4.07589200 -1.47675500

C 4.94576700 4.40576500 -0.77270600

C 6.21196700 4.11993500 -1.36746000

C 7.40801600 4.42228500 -0.65037200

C 7.54421600 2.63751700 -2.81341700

C 8.74364600 3.27363900 -2.36986300

C 10.00924000 2.73741100 -2.75606800

C 11.20667800 3.35448300 -2.28553200

C 11.34146100 0.74075000 -3.30537400

C 12.54106000 1.51356300 -3.23033400

C 13.80674700 0.85701000 -3.30825200

C 15.00198200 1.62046800 -3.19176200

C 15.14210500 -1.15082700 -2.80133900

C 16.34810100 -0.44866800 -3.12025900

C 17.62268700 -1.07355200 -2.91578500

C 18.77411400 -0.32756600 -3.24046100

C -20.62085900 -1.77712700 -2.19320100

C -19.38076500 -2.19551900 -1.59191800

C -18.18007300 -1.70022200 -2.17461700

C -18.04518000 -2.79677700 0.38553000

C -16.84253400 -2.70496800 -0.38293300

C -15.57089900 -2.82129600 0.26179900

C -14.37897400 -2.71509000 -0.51077900

C -14.22519700 -2.38076700 2.27121300

C -13.03401600 -2.69310000 1.54911400

C -11.76097200 -2.46096200 2.15676200

C -10.57214600 -2.75493200 1.42731400

C -10.40331900 -1.06893700 3.66486600

C -9.21533100 -1.70114200 3.18913100

C -7.93824300 -1.19558200 3.58655000

C -6.75166500 -1.81607200 3.09353900

C -6.57901100 0.76436100 4.19226500

C -5.38973600 -0.01799900 4.07036500

C -4.11434900 0.62094300 4.15741600

C -2.92675400 -0.15366200 3.99745400

C -2.76102600 2.63626500 3.76000900

C -1.57121800 1.89339100 4.02313400

C -0.29686700 2.50418300 3.80559500

C 0.89104300 1.75417000 4.05837900

C 1.05922300 4.06401100 2.47790800

C 2.24780100 3.55829600 3.08388000

C 3.52208400 3.96927200 2.58573100

C 4.71124500 3.44508800 3.17796500

C 4.87275600 4.62019300 0.63591300

C 6.06442100 4.49139400 1.40966300

C 7.33453500 4.58677500 0.76416700

C 8.52680900 4.43427700 1.53445900

C 8.67580900 4.16001100 -1.25461600

C 9.87096500 4.43946600 -0.52746500

C 11.13799600 4.19934900 -1.13927300

C 12.33406000 4.45354200 -0.40049100

C 12.47495200 2.83076000 -2.68843300

C 13.67178200 3.43144500 -2.19659500

C 14.93728400 2.91674300 -2.60881800

C 16.13621600 3.49745000 -2.08876400

C 16.27354700 0.96377800 -3.28261900

C 17.46421100 1.72900100 -3.15767800

C 18.72392800 1.07173100 -3.30680600

C 19.90624500 1.88478200 -3.19058800

C -16.90999200 -2.12665200 -1.67469200

C -15.70908300 -1.63391700 -2.27493500

C -14.44550600 -2.09025200 -1.79385800

C -13.24665100 -1.57415600 -2.36934300

C -13.11022600 -2.84006600 0.13392500

C -11.91428700 -2.70566700 -0.63572100

C -10.64785100 -2.85694100 0.00451200

C -9.45332400 -2.69793700 -0.75762900

C -9.30049900 -2.54153000 2.04036900

C -8.11039800 -2.81696300 1.30137500

C -6.83812800 -2.62969300 1.92088400

C -5.65324500 -2.89055100 1.17558300

C -5.47691500 -1.31616500 3.49168900

C -4.28722600 -1.91909600 2.96764700

C -3.01342700 -1.42689300 3.36288000

C -1.82904600 -2.02107300 2.81677300

C -1.65442000 0.47675600 4.10875000

C -0.46460300 -0.30002100 3.92481900

C 0.80913800 0.33293300 4.09969700

C 1.99831200 -0.42922300 3.93138300

C 2.16455200 2.37140000 3.87147200

C 3.35248100 1.61674000 4.10721600

C 4.62795000 2.23880800 3.93421600

C 5.81659500 1.48016500 4.15218400

C 5.98453200 3.87324100 2.69373900

C 7.17316300 3.33390200 3.26995200

C 8.44697200 3.77765600 2.79777100

C 9.63686900 3.22548200 3.35973300

C 9.79715300 4.55554100 0.89360900

C 10.98796700 4.38006500 1.65800000

C 12.25954700 4.52163500 1.02063900

C 13.45239700 4.32514100 1.77995700

C 13.60300600 4.23864900 -1.02030200

C 14.79536900 4.46351300 -0.27384900

C 16.06705800 4.25832400 -0.89538100

C 17.26999900 4.46667500 -0.15055000

C 17.40784900 3.00940800 -2.52436100

C 18.60673100 3.58953600 -2.02172100

C 19.84808800 3.09433100 -2.55903700

C -11.97998800 -2.04107000 -1.89580100

C -10.78042100 -1.50740100 -2.45336500

C -9.51708500 -1.98897600 -1.99372100

C -8.31402900 -1.43242700 -2.52657300

C -8.18596800 -2.87338800 -0.12137100

C -6.99019600 -2.69601400 -0.87724000

C -5.72834700 -2.90122900 -0.24770900

C -4.52711500 -2.69296400 -0.99093000

C -4.37516200 -2.71599200 1.79158000

C -3.18594200 -3.01029700 1.05853200

C -1.92958200 -2.84248800 1.67833400

C -0.70407000 -3.39685700 1.00387900

C -0.54494600 -1.54897500 3.26274300

C 0.65778500 -2.12975500 2.73658200

C 1.92418700 -1.66299300 3.22062600

C 3.12058800 -2.22239800 2.69443900

C 3.27073100 0.19418700 4.12123000

C 4.46410900 -0.56645800 3.93146700

C 5.73625500 0.05499500 4.13039200

C 6.92882000 -0.69604300 3.90920900

C 7.09035300 2.10709100 3.99291300

C 8.28132000 1.34295300 4.18638200

C 9.55517800 1.97617500 4.04670300

C 10.74498200 1.20809100 4.21473300

C 10.90840400 3.68425400 2.90101900

C 12.09971400 3.11328600 3.44419900

C 13.37323700 3.58869100 3.00143600

C 14.56120200 3.00158200 3.52209000

C 14.72024700 4.48377400 1.14861200

C 15.91478600 4.24899600 1.89697100

C 17.19338200 4.43948800 1.28032400

C 18.37950000 4.23940500 2.03815700

C 18.53607700 4.32217000 -0.78955600

C 19.69349500 4.58487300 0.01572400

C -7.05139600 -1.93430500 -2.08552900

C -5.84999100 -1.35294600 -2.58323400

C -4.58536800 -1.86846600 -2.14883600

C -3.26097700 -2.97527400 -0.39232100

C -2.08294900 -2.75067700 -1.13723800

C -0.78167600 -3.35463000 -0.65276500

C 0.45350300 -2.70885800 -1.24673800

C 0.60578800 -2.91134600 1.56342300

C 1.78378100 -3.08478100 0.81127800

C 3.05256200 -2.89606900 1.44250200

C 4.25177300 -3.02682400 0.67985400

C 4.39134900 -1.78017900 3.18863500

C 5.58624600 -2.31042600 2.62226100

C 6.85475300 -1.88268000 3.12054400

C 8.05033600 -2.38246700 2.52074800

C 8.20180100 -0.07885900 4.12032600

C 9.39535000 -0.82162100 3.87063600

C 10.66580500 -0.21251300 4.10074700

C 11.85923800 -0.94491700 3.81743300

C 12.01872300 1.84604600 4.09005700

C 13.21144200 1.07254800 4.23327700

C 14.48048900 1.71195900 4.12647700

C 15.67325300 0.93186300 4.22617500

C 15.83523700 3.48098600 3.08495100

C 17.03456300 2.88832200 3.59480600

C 18.30065000 3.41611100 3.21737100

C 19.45316400 2.78832300 3.78946000

C 19.61863700 4.53617200 1.38225900

C -2.12062100 -1.81121000 -2.18897000

C 0.36271600 -1.72303200 -2.24980900

C 1.70580600 -2.94111700 -0.63328400

C 2.90041300 -2.56788200 -1.32029800

C 4.17572600 -2.83288200 -0.73050100

C 5.36370100 -2.49410500 -1.43900400

C 5.51596100 -2.91486500 1.32869500

C 6.70942300 -3.01082000 0.55461600

C 7.97795600 -2.93035000 1.20602600

C 9.17102400 -3.00625100 0.42757600

C 9.32026900 -1.97723700 3.03472000

C 10.51286100 -2.45354000 2.41516300

C 11.78311100 -2.06546800 2.94129900

C 12.97807300 -2.52125900 2.30362700

C 13.13246100 -0.34449800 4.06941600

C 14.32159700 -1.06325700 3.75878300

C 15.59408700 -0.46715400 4.01638900

C 16.79529600 -1.17773100 3.69212000

C 16.95345400 1.57150000 4.15473100

C 18.13843900 0.79867400 4.29128500

C 19.37551600 1.51779100 4.30003200

C 2.82268800 -1.62396100 -2.38212800

C 5.28729900 -1.53566900 -2.49710200

C 6.63405300 -2.77419400 -0.85041700

C 7.82636400 -2.42128900 -1.55035700

C 9.09604200 -2.72452700 -0.97035300

C 10.28819300 -2.35619700 -1.65938300

C 10.43907500 -2.95379200 1.07947000

C 11.63140100 -3.00249800 0.29787600

C 12.90332200 -2.97414100 0.95097800

C 14.09274100 -3.00002500 0.17130400

C 14.24467600 -2.15378000 2.84066200

C 15.43781700 -2.57281000 2.17861400

C 16.71724400 -2.23743900 2.73058100

C 17.90279800 -2.69356900 2.09417000

C 18.06008900 -0.61797100 4.02260400

C 19.21169100 -1.39722000 3.69022200

C 7.75149300 -1.43936900 -2.58229800

C 11.55693200 -2.67748300 -1.08756400

C 12.75241100 -2.28991000 -1.76661200

C 14.01980400 -2.63164700 -1.20854000

C 15.21168100 -2.21584200 -1.86578500

C 15.36178800 -2.97892100 0.82312500

C 16.56495500 -2.99349600 0.04508300

C 17.82670200 -3.06197500 0.69667400

C 18.97594000 -3.11332000 -0.14716300

C 19.13652000 -2.39573400 2.75023900

C 16.49246600 -2.57967400 -1.32540200

C 17.68165900 -2.21307400 -2.00662800

C 18.90504200 -2.69540800 -1.45585400

H -21.63265800 -0.29797200 -3.34534200

H -20.50819100 1.97512400 -3.38999400

H -20.56896800 3.95110900 -2.52434800

H -20.79816400 4.86045700 1.82619600

H -20.68876200 5.02277800 -0.36153900

H -20.93912800 3.36134200 3.71815800

H -21.04999200 1.33976900 4.55833700

H -21.30683500 -2.60927000 2.49756000

H -21.19534700 -1.01088500 4.00251700

H -21.44234300 -3.05153100 0.12118000

H 19.75383600 -0.79209700 -3.18541400

H -21.54483600 -2.27560100 -1.91727100

H 20.86601400 1.46780400 -3.48533500

H 20.76972400 3.63294200 -2.36182300

H 20.66658500 4.68818100 -0.45369700

H 20.42821600 3.25497400 3.69169900

H 20.53405300 4.60274400 1.96203700

H 20.29094400 1.01415000 4.59490800

H 20.18710500 -1.10981000 4.06969300

H 19.94964600 -3.34088700 0.27508800

H 20.05444200 -2.86947300 2.41622900

H 19.82566500 -2.60904500 -2.02478500

H -0.72057600 -4.47093500 1.21819400

C -0.80227200 -4.88286400 -1.04672300

C -0.98229400 -5.25042400 -2.55419900

C -0.62622400 -6.73098900 -2.89176400

C -1.15772600 -7.21547800 -4.26894700

F -1.82933600 -5.50477500 -0.39613100

F 0.35237900 -5.47827100 -0.62451500

F -2.27788000 -5.03878700 -2.89934700

F -0.19649800 -4.46845500 -3.33170200

F -1.13843600 -7.55812600 -1.95070700

F 0.71976000 -6.85642200 -2.90947200

F -2.48663300 -7.36317500 -4.23897900

F -0.60107100 -8.39986400 -4.55437800

F -0.83437800 -6.34363500 -5.23401900

**CF_3_(CF_2_)_3_-(6,5) SWNT-H (1,2-L_+_: L_27_)**

C -20.59380000 -0.67462300 -2.99780800

C -19.40662200 0.10605500 -3.22837000

C -19.44743400 1.50122500 -3.35926500

C -18.29066600 2.27765600 -3.14445400

C -18.15069800 -0.53195000 -2.99129600

C -16.95514100 0.20051700 -3.22273700

C -15.68769400 -0.44534700 -3.04455400

C -14.48733700 0.28666100 -3.26544100

C -19.56185600 4.09919000 -1.93050700

C -18.34180800 3.53486800 -2.40603800

C -17.15041900 3.98488200 -1.78120900

C -17.02000100 1.62208000 -3.25876300

C -15.80891800 2.35337800 -3.04023100

C -14.54637900 1.71413100 -3.23450200

C -13.34544200 2.43996300 -2.98556900

C -13.22565600 -0.36191700 -3.10162600

C -12.02030700 0.38448900 -3.28048800

C -10.75870700 -0.27083500 -3.14000300

C -9.55251900 0.48097900 -3.28359700

C -19.79883000 4.37410600 2.27459400

C -19.63103400 4.69579300 -0.69278100

C -18.48325200 4.75208300 0.15263600

C -17.22149200 4.58630200 -0.48183000

C -16.01931800 4.67285900 0.29253500

C -15.87170100 3.53948700 -2.26292300

C -14.67749200 4.03495400 -1.66838700

C -13.41130600 3.60807700 -2.16904100

C -12.21407400 4.07930700 -1.54907900

C -12.07907000 1.80646400 -3.19348400

C -10.87820400 2.52604200 -2.91719700

C -9.61302500 1.90115400 -3.14205300

C -8.41359800 2.61078000 -2.83611400

C -8.29144200 -0.17886200 -3.16428700

C -7.08307100 0.57900100 -3.26318200

C -5.82039000 -0.08306200 -3.16047500

C -4.62117500 0.68172100 -3.21287200

C -3.28833100 -1.31607900 -2.61766500

C -0.85267800 -1.22856700 -2.62486000

C -19.87909600 3.51335100 3.34185900

C -18.73084100 2.78347200 3.78057100

C -18.56292000 4.57668200 1.58713400

C -17.38021300 4.20650600 2.28195600

C -16.09891000 4.45828000 1.69131400

C -14.90897500 4.12973200 2.40711100

C -14.74982900 4.59496500 -0.35448100

C -13.56169000 4.67289400 0.42334000

C -12.28889300 4.60091100 -0.22487300

C -11.09886300 4.65989900 0.55854800

C -10.94623300 3.66878000 -2.06357000

C -9.75288900 4.12414400 -1.43057700

C -8.48375400 3.72928300 -1.95328300

C -7.29148900 4.17379000 -1.30790900

C -7.14664800 1.99263100 -3.07467100

C -5.95093900 2.70019400 -2.75155900

C -4.68560200 2.09186100 -3.00144800

C -3.49107300 2.79653200 -2.66409500

C -3.35556700 0.02231200 -3.10114900

C -2.15953700 0.78802100 -3.16406600

C -0.90433100 0.12782500 -3.08829600

C 0.29131000 0.89690200 -3.15243000

C 1.63257300 -1.12613100 -2.73561400

C 4.10170100 -1.01097400 -2.86155600

C -20.05343000 0.71222900 4.35610000

C -18.81425100 1.41851000 4.24382600

C -17.63205600 0.62961200 4.21634300

C -17.46358900 3.28747900 3.37821700

C -16.26536300 2.62398900 3.79919800

C -14.99096700 3.17471300 3.46520300

C -13.80483800 2.50036700 3.87274600

C -13.64060900 4.41461900 1.82612400

C -12.44921900 4.04908100 2.52449900

C -11.17783700 4.35631300 1.95193100

C -9.98899400 3.96970800 2.63683600

C -9.82903600 4.61010100 -0.09033800

C -8.63849100 4.64658800 0.69465800

C -7.36786800 4.62172100 0.04455300

C -6.17712300 4.63854200 0.83014000

C -6.02216700 3.79964000 -1.84181100

C -4.83133400 4.22961700 -1.18579200

C -3.56150800 3.87168000 -1.73073000

C -2.36765300 4.28397200 -1.06554900

C -2.22536000 2.19979400 -2.93527200

C -1.03435000 2.88952400 -2.58842500

C 0.22923400 2.28975300 -2.87690900

C 1.42879400 2.97520300 -2.52183400

C 1.55997100 0.23507700 -3.14708100

C 2.75274300 1.01302900 -3.20638700

C 4.02491500 0.36038300 -3.22880900

C 5.22303800 1.14261200 -3.23932100

C 6.56595600 -0.89677900 -2.93166700

C 9.03159800 -0.78167200 -2.98659900

C -20.31186800 -2.68578700 1.89193900

C -20.13613300 -0.61747700 4.02986300

C -18.98690900 -1.32372200 3.55048300

C -17.71837000 -0.75236600 3.84765300

C -16.52155100 -1.41549800 3.42553700

C -16.34942200 1.26734800 4.19896500

C -15.15954700 0.47652800 4.20839200

C -13.88810700 1.11918500 4.22671200

C -12.69724400 0.32930600 4.18997100

C -12.52998400 3.05797500 3.54527500

C -11.34002800 2.36658400 3.92652300

C -10.06839800 2.93822700 3.62099400

C -8.87712500 2.23355200 3.97057100

C -8.71726700 4.29761200 2.07552400

C -7.52683700 3.88942800 2.74693100

C -6.25544000 4.24179200 2.19900900

C -5.06394900 3.81149500 2.85380900

C -4.90639100 4.63737600 0.18008700

C -3.71415200 4.62694300 0.96487800

C -2.44145900 4.64654900 0.31406400

C -1.24842500 4.60583200 1.09440300

C -1.10083800 3.94195700 -1.62356700

C 0.09486500 4.31951400 -0.94654800

C 1.36289600 3.98790600 -1.52038900

C 2.55961000 4.34344700 -0.83394200

C 2.68947500 2.39692600 -2.85458300

C 3.88861600 3.06425900 -2.46734300

C 5.15584800 2.50524000 -2.82471200

C 6.35405800 3.15719300 -2.40697700

C 6.49141500 0.48780000 -3.27303900

C 7.68880200 1.26638300 -3.24449100

C 8.95912100 0.61039800 -3.29055500

C 10.15563100 1.38577900 -3.23642200

C 10.29919900 -1.36224800 -2.67937900

C 11.49390900 -0.67015800 -3.03655900

C 12.76409200 -1.26253300 -2.74581100

C 13.95901100 -0.56457900 -3.08275100

C -20.38964500 -2.92055500 0.54479000

C -19.23299400 -2.77362600 -0.29045600

C -19.07070800 -2.30460200 2.49887300

C -17.88672600 -2.61233000 1.77442400

C -16.60620100 -2.34257300 2.35716700

C -15.41423600 -2.68375300 1.64716600

C -15.24521600 -0.88424700 3.78918400

C -14.05934400 -1.54193300 3.35343100

C -12.78263100 -1.01426400 3.72332300

C -11.59363500 -1.65733900 3.26128900

C -11.42225700 0.97367000 4.22713700

C -10.23308200 0.18679700 4.15923700

C -8.95766700 0.83009200 4.21517000

C -7.76787600 0.04744700 4.11399300

C -7.60574000 2.81996300 3.68812000

C -6.41387100 2.10339200 4.00802600

C -5.14298700 2.70449600 3.75220100

C -3.95234800 1.97708800 4.04199400

C -3.79240700 4.18293100 2.31799800

C -2.59921000 3.72632600 2.95594000

C -1.32597100 4.11374300 2.43486200

C -0.13547100 3.62947500 3.05233400

C 0.02266700 4.63647800 0.44232700

C 1.21814800 4.56001600 1.22046000

C 2.48838100 4.61107100 0.56742200

C 3.68115900 4.50355200 1.34075600

C 3.82407600 4.03172500 -1.42104800

C 5.02273300 4.36024200 -0.71920000

C 6.28824200 4.07880200 -1.31818600

C 7.48427800 4.37883200 -0.60210600

C 7.62072200 2.61273800 -2.78154200

C 8.82056000 3.24660000 -2.33249900

C 10.08763600 2.71812400 -2.72638000

C 11.28453200 3.33142600 -2.24933000

C 11.42318200 0.72934700 -3.29708500

C 12.62240500 1.50239800 -3.21256700

C 13.88881500 0.84743800 -3.29549500

C 15.08341000 1.61029200 -3.17028700

C 15.22499800 -1.16411700 -2.80381700

C 16.43112100 -0.45858600 -3.11458400

C 17.70560800 -1.08460500 -2.91335000

C 18.85705700 -0.33539000 -3.23024700

C -20.54190400 -1.78649800 -2.20646600

C -19.30237800 -2.21267500 -1.60991000

C -18.10117600 -1.71353900 -2.18858300

C -17.96652100 -2.83582700 0.36084700

C -16.76423400 -2.73781800 -0.40734700

C -15.49284700 -2.86204600 0.23548300

C -14.30151100 -2.74976400 -0.53715600

C -14.14427900 -2.44197000 2.24796000

C -12.95361700 -2.74846700 1.52188900

C -11.67999200 -2.52181800 2.12942700

C -10.49202500 -2.80933100 1.39458500

C -10.31831900 -1.14197700 3.64629900

C -9.12884700 -1.76783300 3.16075500

C -7.85254600 -1.26247300 3.55623800

C -6.66379700 -1.86982100 3.04296500

C -6.49302100 0.69050000 4.19137400

C -5.30208500 -0.08405600 4.05484700

C -4.03236000 0.55816000 4.15929700

C -2.84097000 -0.20766400 3.98078300

C -2.67945000 2.58752600 3.81058600

C -1.48923600 1.85086300 4.07805700

C -0.21753800 2.46067700 3.86909900

C 0.97012400 1.70542800 4.10610600

C 1.13954900 4.02411600 2.53962900

C 2.32945500 3.51296100 3.14098700

C 3.60222200 3.92225400 2.64194900

C 4.79067000 3.38846100 3.22557100

C 4.95140800 4.57360400 0.68913700

C 6.14377400 4.44144400 1.46065700

C 7.41228900 4.53732100 0.81383500

C 8.60466000 4.37848400 1.58208000

C 8.75255300 4.12182000 -1.20906800

C 9.94795600 4.39778400 -0.48062900

C 11.21516500 4.16448400 -1.09438100

C 12.41122600 4.41256100 -0.35281300

C 12.55439100 2.81363300 -2.65726600

C 13.75066500 3.41042000 -2.15880100

C 15.01708600 2.90092400 -2.57515200

C 16.21546000 3.47710600 -2.04823800

C 16.35569700 0.95501000 -3.26534500

C 17.54573700 1.71967000 -3.13256800

C 18.80601000 1.06432400 -3.28557200

C 19.98775100 1.87711900 -3.16150400

C -16.83158300 -2.14685300 -1.69366200

C -15.63061900 -1.65047500 -2.29014000

C -14.36786000 -2.11334100 -1.81401000

C -13.16833900 -1.59443800 -2.38647700

C -13.03256600 -2.88306100 0.10520800

C -11.83860400 -2.74463000 -0.66475700

C -10.57235500 -2.90194800 -0.02691700

C -9.37899700 -2.74260600 -0.79201300

C -9.21713200 -2.60099000 2.00609300

C -8.03058500 -2.87276200 1.26283600

C -6.75480600 -2.67607800 1.87455500

C -5.56681600 -2.93163100 1.11901100

C -5.38310800 -1.37239700 3.44275700

C -4.19922000 -1.94872300 2.89537300

C -2.91893900 -1.45428000 3.30249400

C -1.72926800 -2.01313600 2.74705100

C -1.56950800 0.42545200 4.13470400

C -0.38629500 -0.33615900 3.95117200

C 0.88566400 0.28318900 4.12363500

C 2.07224400 -0.48420000 3.92476200

C 2.24584200 2.31864900 3.91877800

C 3.42978000 1.55546600 4.13815200

C 4.70544000 2.17339800 3.96723300

C 5.89227500 1.40632800 4.16995500

C 6.06430700 3.81647400 2.74187400

C 7.25097100 3.26885400 3.31099500

C 8.52460600 3.71279700 2.84042300

C 9.71338900 3.15268800 3.39644000

C 9.87471000 4.50313700 0.94153400

C 11.06475200 4.32084800 1.70441200

C 12.33641800 4.46701100 1.06871600

C 13.52865700 4.26300200 1.82702600

C 13.68082300 4.20517700 -0.97398400

C 14.87256400 4.42277100 -0.22434400

C 16.14502300 4.22496700 -0.84682500

C 17.34729100 4.42551200 -0.09851800

C 17.48786900 2.99428700 -2.48766900

C 18.68593500 3.56991500 -1.97813400

C 19.92817800 3.08088600 -2.51927800

C -11.90372800 -2.06885800 -1.91936500

C -10.70345200 -1.53166300 -2.47344300

C -9.44090300 -2.02402700 -2.02342800

C -8.23785600 -1.46931200 -2.55567700

C -8.11449100 -2.92506100 -0.16100000

C -6.92398300 -2.74642900 -0.92403500

C -5.65888700 -2.96271700 -0.30177700

C -4.47239500 -2.80705000 -1.06574700

C -4.28840000 -2.72710100 1.70820400

C -3.10541100 -2.96020800 0.92193400

C -1.79390500 -2.76820200 1.52898200

C -0.62018000 -3.05449700 0.82936700

C -0.46711200 -1.56379500 3.21839800

C 0.72058900 -2.12232200 2.67292600

C 1.99252100 -1.69926900 3.18047500

C 3.18528300 -2.26339000 2.65075400

C 3.34437200 0.12914100 4.12349400

C 4.53293400 -0.63138600 3.91216700

C 5.80801100 -0.01802200 4.12321900

C 6.99843700 -0.76899700 3.89527400

C 7.16609200 2.03234800 4.01952900

C 8.35461200 1.26297900 4.20116700

C 9.62968100 1.89606400 4.06982000

C 10.81771100 1.12496900 4.23040000

C 10.98462700 3.61398100 2.94164200

C 12.17465100 3.03625200 3.47923300

C 13.44855600 3.51485800 3.04135500

C 14.63537600 2.92230300 3.55730900

C 14.79668600 4.42771600 1.19832200

C 15.99050100 4.18477600 1.94524200

C 17.26962600 4.38181500 1.33192600

C 18.45498400 4.17331200 2.08864600

C 18.61394400 4.28877600 -0.73794800

C 19.77064000 4.54214100 0.07138700

C -6.97854800 -1.98556400 -2.12534700

C -5.76269600 -1.41275300 -2.62505000

C -4.51540000 -1.95899300 -2.22323200

C -3.21520700 -3.06242700 -0.45753200

C -2.02002300 -3.12620200 -1.38969000

C -0.66145800 -3.47968900 -0.66212700

C 0.54802200 -2.81002500 -1.32247800

C 0.64176200 -2.85224700 1.45124900

C 1.86745400 -3.08732100 0.73442400

C 3.11577300 -2.93725800 1.38680200

C 4.32647700 -3.06796600 0.63138800

C 4.45406600 -1.83436900 3.14954500

C 5.65566400 -2.36484000 2.58308600

C 6.92206200 -1.94777900 3.09087600

C 8.12066500 -2.44284200 2.49010300

C 8.27183800 -0.15786000 4.11627900

C 9.46614300 -0.89954400 3.86059900

C 10.73658900 -0.29506200 4.10085800

C 11.93024800 -1.02500900 3.81174200

C 12.09187300 1.76293900 4.11285800

C 13.28397000 0.98728200 4.24966600

C 14.55322500 1.62713400 4.15021500

C 15.74541300 0.84585000 4.24410000

C 15.90980200 3.40495700 3.12538400

C 17.10839500 2.80710700 3.63076700

C 18.37494800 3.33792200 3.25940600

C 19.52662400 2.70448200 3.82672200

C 19.69465400 4.47745900 1.43722100

C -2.03112600 -1.86312100 -2.24963900

C 0.44280800 -1.80299200 -2.28214300

C 1.81230900 -3.00004400 -0.69510700

C 2.99735000 -2.62981100 -1.38340300

C 4.26466400 -2.87716200 -0.77576900

C 5.45487900 -2.53366000 -1.48171800

C 5.58981900 -2.96215300 1.28726500

C 6.78622200 -3.05765500 0.51710700

C 8.05248500 -2.98005500 1.17189100

C 9.24924100 -3.05148800 0.39556500

C 9.39102700 -2.04563500 3.01213700

C 10.58514100 -2.51786600 2.39177100

C 11.85483000 -2.13635600 2.92411600

C 13.05163100 -2.58653100 2.28426500

C 13.20394100 -0.42807300 4.07220100

C 14.39323800 -1.14436900 3.75694700

C 15.66564600 -0.55123400 4.02199900

C 16.86725800 -1.25925000 3.69320200

C 17.02603100 1.48565100 4.17944900

C 18.21045200 0.71138600 4.31118200

C 19.44783200 1.42972800 4.32669500

C 2.90873700 -1.68059500 -2.44043900

C 5.37491300 -1.57170900 -2.53340300

C 6.71883200 -2.81438000 -0.88859800

C 7.91175800 -2.45323200 -1.58188000

C 9.17935900 -2.75926100 -0.99993300

C 10.37167200 -2.38422900 -1.68345600

C 10.51482200 -3.00550500 1.05065300

C 11.70924400 -3.04661100 0.27111300

C 12.97988800 -3.02506000 0.92687500

C 14.17078300 -3.04320900 0.14907400

C 14.31736400 -2.22581200 2.82754300

C 15.51176600 -2.63805500 2.16316500

C 16.79044500 -2.30928300 2.72102700

C 17.97700000 -2.75905600 2.08198300

C 18.13169000 -0.70315200 4.03093100

C 19.28344800 -1.47996300 3.69369200

C 7.83643700 -1.46394000 -2.60532000

C 11.63846300 -2.70883000 -1.11116200

C 12.83501600 -2.31400700 -1.78442400

C 14.10085100 -2.66062000 -1.22723700

C 15.29361300 -2.23752500 -1.87801500

C 15.43848000 -3.02914000 0.80314300

C 16.64324000 -3.03510700 0.02697600

C 17.90365700 -3.11121500 0.68000300

C 19.05457100 -3.15220200 -0.16209100

C 19.20953200 -2.46904300 2.74364400

C 16.57346600 -2.60627700 -1.33897800

C 17.76367800 -2.23234800 -2.01436300

C 18.98618500 -2.71962300 -1.46620400

H -21.55174500 -0.29502100 -3.34444100

H -20.42369100 1.97610900 -3.36933800

H -20.48245600 3.94245600 -2.48427300

H -20.71473400 4.80003700 1.87671500

H -20.60321900 4.98898000 -0.30928800

H -20.85633600 3.28322500 3.75426700

H -20.96661200 1.25502600 4.57964100

H -21.22643300 -2.66952400 2.47665100

H -21.11278600 -1.09008400 4.00057600

H -21.36399100 -3.08417800 0.09554100

H 19.83699200 -0.79977100 -3.17774000

H -21.46673100 -2.28548500 -1.93433400

H 20.94800600 1.46345200 -3.45931000

H 20.84917600 3.61854500 -2.31656400

H 20.74412900 4.65094200 -0.39594700

H 20.50206600 3.17126400 3.73357100

H 20.60960100 4.53743400 2.01845400

H 20.36280600 0.92316400 4.61792500

H 20.25825900 -1.19696400 4.07799400

H 20.02756600 -3.38391700 0.25953500

H 20.12779200 -2.94010300 2.40677600

H 19.90798400 -2.62643500 -2.03214400

H -2.23082200 -3.95162200 -2.07752000

C -0.54082000 -5.05389800 -0.68655200

C -0.41187300 -5.78106900 -2.07234300

C 0.21853200 -7.20331600 -1.98478600

C 0.01639300 -8.07322200 -3.25707100

F -1.65077200 -5.57879200 -0.09136400

F 0.53195400 -5.46010300 0.04978300

F -1.65252800 -5.92040500 -2.62241800

F 0.34471400 -5.04955800 -2.92394800

F -0.32795900 -7.87709300 -0.94615000

F 1.55005700 -7.07651000 -1.79265400

F 0.78596800 -9.16490100 -3.16092400

F -1.25930000 -8.45766000 -3.37360700

F 0.37093600 -7.39110900 -4.35535800

**CF_3_(CF_2_)_3_-(6,5) SWNT-H (1,2-L–: L_–33_)**

C -20.65919800 -0.58244600 -2.85165800

C -19.47156100 0.19574100 -3.08843200

C -19.51148200 1.58991400 -3.23057000

C -18.35404300 2.36721300 -3.02300000

C -18.21604700 -0.44125300 -2.84665800

C -17.02016500 0.28856800 -3.08414500

C -15.75284900 -0.35668800 -2.90091100

C -14.55248100 0.37240800 -3.12879100

C -19.62261600 4.20009400 -1.82338300

C -18.40349600 3.63082600 -2.29509200

C -17.21133200 4.08483500 -1.67468800

C -17.08406700 1.70962400 -3.13233600

C -15.87211600 2.44206900 -2.92115800

C -14.61046100 1.80044600 -3.11107100

C -13.40908900 2.52770000 -2.86921100

C -13.29124800 -0.27525700 -2.95856100

C -12.08529000 0.46901600 -3.14575400

C -10.82450000 -0.18521600 -2.99980900

C -9.61958700 0.56545200 -3.15146300

C -19.85710600 4.50777900 2.37887000

C -19.69031000 4.80680200 -0.59041500

C -18.54196200 4.86842400 0.25373600

C -17.28088000 4.69618500 -0.37999500

C -16.07792800 4.78667200 0.39321600

C -15.93320100 3.63471500 -2.15384600

C -14.73819900 4.13392100 -1.56427400

C -13.47297500 3.70190200 -2.06251900

C -12.27395100 4.17663300 -1.44680700

C -12.14277000 1.89160100 -3.07318200

C -10.94175100 2.61318000 -2.80601300

C -9.67746200 1.98456600 -3.02610700

C -8.47463000 2.69713700 -2.72972800

C -8.35610000 -0.09403500 -3.02581100

C -7.15105500 0.66148300 -3.14305300

C -5.88879100 -0.00234800 -3.02894500

C -4.67812000 0.75609800 -3.10282300

C -3.36412400 -1.23724400 -2.47876700

C -0.87707600 -1.15106600 -2.49096200

C -19.93837700 3.65481800 3.45237800

C -18.79113700 2.92673500 3.89628400

C -18.62117800 4.70351100 1.68970700

C -17.43884300 4.33635800 2.38654100

C -16.15722900 4.58175700 1.79352000

C -14.96753600 4.25677200 2.51107700

C -14.80901200 4.70291900 -0.25380400

C -13.62031500 4.78360600 0.52295400

C -12.34751200 4.70570200 -0.12544600

C -11.15711300 4.76731400 0.65679100

C -11.00743900 3.76222100 -1.95998100

C -9.81249600 4.21814800 -1.32940600

C -8.54306700 3.81915300 -1.85127000

C -7.34995700 4.26255300 -1.20828100

C -7.21037800 2.07704900 -2.96749000

C -6.01022000 2.78018000 -2.64385200

C -4.74272400 2.16750400 -2.89194800

C -3.54944900 2.86890500 -2.55307300

C -3.41924400 0.09232000 -2.99168900

C -2.21677100 0.86132200 -3.03671700

C -0.95413100 0.20043900 -2.95435200

C 0.23644800 0.97070700 -3.02538100

C 1.55368900 -1.05173700 -2.57959900

C 4.02591400 -0.95918700 -2.72093600

C -20.11656900 0.86160600 4.48778100

C -18.87643900 1.56538000 4.37036600

C -17.69540200 0.77487300 4.34957900

C -17.52332300 3.42572800 3.48966600

C -16.32590600 2.76391300 3.91568100

C -15.05069000 3.31002600 3.57707700

C -13.86591400 2.63765300 3.99023200

C -13.69921100 4.53474600 1.92722300

C -12.50778000 4.17214500 2.62792000

C -11.23637800 4.47282000 2.05279800

C -10.04850600 4.08881800 2.74024800

C -9.88785800 4.70985100 0.00771700

C -8.69608500 4.74829700 0.79270400

C -7.42633100 4.71714200 0.14307800

C -6.23628400 4.73492500 0.92965900

C -6.08100200 3.87926900 -1.73821400

C -4.88776500 4.31194000 -1.08248300

C -3.62001700 3.95009200 -1.62350800

C -2.42692000 4.36356500 -0.95573800

C -2.28353500 2.26213400 -2.80888800

C -1.08666100 2.96616300 -2.46885100

C 0.17236000 2.37760600 -2.76183800

C 1.36880900 3.05667900 -2.40005900

C 1.49424800 0.31263100 -2.99159200

C 2.69539600 1.08608800 -3.05481800

C 3.95757800 0.42705100 -3.08420400

C 5.15460400 1.20336200 -3.09270100

C 6.50039800 -0.83578400 -2.78866800

C 8.96563000 -0.71991100 -2.84074900

C -20.37829900 -2.55706800 2.05228800

C -20.20082600 -0.47068400 4.17246900

C -19.05229000 -1.18244900 3.69974700

C -17.78329100 -0.61013900 3.99255800

C -16.58719800 -1.27821900 3.57625800

C -16.41186400 1.41088800 4.32689300

C -15.22305700 0.61873000 4.34397400

C -13.95111200 1.25980000 4.35708700

C -12.76141700 0.46786400 4.32804300

C -12.58996700 3.19001500 3.65718900

C -11.40118900 2.50119800 4.04591900

C -10.12970100 3.06713800 3.73412200

C -8.93901400 2.36355100 4.09148300

C -8.77588100 4.40928100 2.17594000

C -7.58680400 4.00604000 2.85182700

C -6.31620700 4.34946100 2.30043700

C -5.12459700 3.92271300 2.96120100

C -4.96437100 4.72766100 0.28058500

C -3.77404600 4.72386300 1.06683000

C -2.50261700 4.73479200 0.41763800

C -1.30767100 4.70130400 1.20314100

C -1.15687200 4.02085400 -1.51110900

C 0.03720700 4.41031800 -0.83698300

C 1.30248000 4.07549500 -1.40362200

C 2.50137900 4.43199100 -0.71463800

C 2.63194100 2.47049300 -2.71997300

C 3.83040200 3.13935800 -2.33857700

C 5.09166700 2.57059900 -2.68663700

C 6.29280200 3.22372500 -2.27323400

C 6.42709400 0.54876500 -3.13005700

C 7.62220000 1.32603400 -3.10637200

C 8.89326800 0.67035100 -3.14933400

C 10.09080100 1.44588400 -3.09895900

C 10.23443500 -1.29965800 -2.53070800

C 11.42842700 -0.61002600 -2.89121600

C 12.69870500 -1.20070900 -2.59652700

C 13.89396400 -0.50485600 -2.93715000

C -20.45611200 -2.80227900 0.70705500

C -19.29924300 -2.66240100 -0.12919300

C -19.13680500 -2.17212300 2.65624400

C -17.95316000 -2.48661000 1.93445700

C -16.67242600 -2.21343400 2.51527200

C -15.48047600 -2.56124000 1.80817800

C -15.31023400 -0.74577400 3.93633700

C -14.12549100 -1.40845700 3.50682500

C -12.84830300 -0.87915500 3.87307800

C -11.65956700 -1.52790400 3.41788400

C -11.48508600 1.11047100 4.36104500

C -10.29831300 0.32177000 4.30275200

C -9.02139200 0.96350400 4.35201200

C -7.83228700 0.17820100 4.26432000

C -7.66733000 2.94521900 3.80477600

C -6.47786100 2.23024000 4.13303900

C -5.20548300 2.82581100 3.87045000

C -4.01574300 2.10178600 4.17288000

C -3.85389700 4.28976300 2.42514400

C -2.66282700 3.83684300 3.06765200

C -1.38826200 4.22003100 2.54437200

C -0.19800500 3.74358000 3.17054300

C -0.03741100 4.73481100 0.55443200

C 1.15563700 4.66192200 1.33395300

C 2.42801200 4.70683900 0.68280100

C 3.62108300 4.60583100 1.45949900

C 3.76540500 4.11830100 -1.29905000

C 4.96214200 4.44644200 -0.59837900

C 6.22776700 4.15470000 -1.19384900

C 7.42400600 4.46011200 -0.48057400

C 7.55690200 2.67726400 -2.64766500

C 8.75624200 3.31130400 -2.20288100

C 10.02396700 2.78024100 -2.59473400

C 11.22067000 3.39626200 -2.12282900

C 11.35793200 0.78909200 -3.15815600

C 12.55728300 1.56173700 -3.07679800

C 13.82404500 0.90601200 -3.15706400

C 15.01863100 1.66900300 -3.03634800

C 15.15961400 -1.10330200 -2.65491200

C 16.36579900 -0.39988600 -2.96927400

C 17.64030500 -1.02520800 -2.76443800

C 18.79166300 -0.27839000 -3.08621400

C -20.60777800 -1.68843200 -2.05217400

C -19.36834400 -2.11097700 -1.45268600

C -18.16700900 -1.61675200 -2.03485700

C -18.03289400 -2.72071500 0.52251000

C -16.83045100 -2.62857500 -0.24621500

C -15.55879500 -2.74919100 0.39759900

C -14.36749100 -2.64274000 -0.37528600

C -14.21072400 -2.31673800 2.40772900

C -13.02014000 -2.62835400 1.68388500

C -11.74586400 -2.39960300 2.29105900

C -10.55848700 -2.69208000 1.55955800

C -10.38466100 -1.01229800 3.80139600

C -9.19612300 -1.64293000 3.32133800

C -7.91705600 -1.14002500 3.71872800

C -6.73214100 -1.75518000 3.21892800

C -6.55931200 0.81982100 4.33443000

C -5.36788800 0.04038200 4.20907000

C -4.09714900 0.68112400 4.31024900

C -2.90870100 -0.08844400 4.14686600

C -2.74548200 2.70709200 3.93351500

C -1.55391600 1.96922700 4.20726700

C -0.28244200 2.57912800 3.99693900

C 0.90389800 1.82698900 4.23924800

C 1.07473800 4.13339200 2.65725800

C 2.26632300 3.62371900 3.26266400

C 3.54043800 4.03195500 2.76467400

C 4.72744100 3.50091400 3.35211200

C 4.89002500 4.67040800 0.80930600

C 6.08314700 4.53971400 1.58212700

C 7.35183900 4.63053200 0.93492200

C 8.54324800 4.47472800 1.70354900

C 8.68993400 4.19544500 -1.08544800

C 9.88648600 4.47518400 -0.35926800

C 11.15269300 4.23734300 -0.97236800

C 12.34862400 4.48912400 -0.23321600

C 12.49004300 2.87568900 -2.52799500

C 13.68706700 3.47489800 -2.03333600

C 14.95302600 2.96306600 -2.44772100

C 16.15165000 3.54140100 -1.92429700

C 16.29070400 1.01283000 -3.12797800

C 17.48113000 1.77781500 -2.99967500

C 18.74103600 1.12121900 -3.14964800

C 19.92300600 1.93408200 -3.03024400

C -16.89753800 -2.04681600 -1.53643800

C -15.69611500 -1.55557400 -2.13660600

C -14.43363600 -2.01532500 -1.65694400

C -13.23410100 -1.50070500 -2.23268100

C -13.09842300 -2.77136100 0.26858400

C -11.90341600 -2.63828600 -0.50181200

C -10.63741300 -2.79219000 0.13742100

C -9.44389900 -2.63470000 -0.62666300

C -9.28408100 -2.47984100 2.17124800

C -8.09525500 -2.75616300 1.43127000

C -6.82128900 -2.56656100 2.04772500

C -5.63567300 -2.82462600 1.29703900

C -5.45034300 -1.25580800 3.61705000

C -4.26381900 -1.84992100 3.09305100

C -2.99095000 -1.35170100 3.49113100

C -1.80019000 -1.91306100 2.92760500

C -1.63686200 0.54747200 4.28266400

C -0.45108100 -0.21688900 4.10120300

C 0.81673700 0.40382700 4.26113000

C 2.00473100 -0.36383700 4.05286800

C 2.18052700 2.43653900 4.04533600

C 3.36605400 1.67217500 4.27016300

C 4.64064100 2.28807000 4.09938400

C 5.82729300 1.51897300 4.30321600

C 6.00217600 3.92311200 2.86592300

C 7.18909700 3.37667700 3.43895500

C 8.46231800 3.81659700 2.96656500

C 9.65097800 3.25694300 3.52458300

C 9.81346200 4.59171900 1.06149400

C 11.00360800 4.41368600 1.82556200

C 12.27439900 4.55373400 1.18831500

C 13.46690600 4.35317500 1.94750500

C 13.61815900 4.27666700 -0.85337800

C 14.81006500 4.49872900 -0.10565900

C 16.08199600 4.29624200 -0.72712300

C 17.28471200 4.50053100 0.01970400

C 17.42393200 3.05580700 -2.36153500

C 18.62228900 3.63384400 -1.85575300

C 19.86404800 3.14143000 -2.39447700

C -11.96865900 -1.97165600 -1.76128800

C -10.76917700 -1.43984400 -2.31949300

C -9.50676600 -1.92582700 -1.86181100

C -8.30305300 -1.37462300 -2.39891800

C -8.17653500 -2.81305200 0.00759200

C -6.98576700 -2.63801800 -0.75145000

C -5.72212800 -2.83964000 -0.12115000

C -4.52836300 -2.65147000 -0.87802100

C -4.34876900 -2.64848500 1.90556800

C -3.17772700 -2.90552100 1.15140600

C -1.87624000 -2.68901300 1.73140000

C -0.69280000 -2.96663800 1.00124400

C -0.53423300 -1.45100800 3.37703100

C 0.64810800 -2.00233100 2.81599600

C 1.92064000 -1.57031800 3.30792000

C 3.11938000 -2.13764200 2.77622500

C 3.27933900 0.24747800 4.25711500

C 4.46552400 -0.51550100 4.05107600

C 5.74108900 0.09612100 4.26058000

C 6.93287400 -0.65807700 4.03572700

C 7.10271900 2.14331800 4.15243100

C 8.28991200 1.37347100 4.33781000

C 9.56575200 2.00404800 4.20335900

C 10.75369700 1.23204200 4.36762100

C 10.92283900 3.71471700 3.06749900

C 12.11198300 3.13864500 3.60774600

C 13.38630400 3.61302200 3.16641300

C 14.57281400 3.02235200 3.68567000

C 14.73470100 4.51299700 1.31736200

C 15.92822800 4.27368900 2.06527200

C 17.20742600 4.46563200 1.45033200

C 18.39276600 4.26118200 2.20811000

C 18.55103900 4.35970200 -0.61926600

C 19.70796000 4.61672400 0.18820400

C -7.04391600 -1.88310900 -1.96188200

C -5.83866200 -1.30731100 -2.46561300

C -4.57741000 -1.83569100 -2.04197100

C -3.27066500 -2.92051600 -0.27613200

C -2.08040800 -2.75497000 -1.03977600

C -0.79495700 -3.38258900 -0.48281700

C 0.47042500 -2.98598700 -1.35098300

C 0.55664700 -2.72875300 1.58669500

C 1.79093500 -2.93498200 0.84090400

C 3.05351500 -2.80064000 1.51732800

C 4.25721000 -2.96950000 0.78046200

C 4.38512100 -1.71900000 3.28660100

C 5.58727900 -2.25252200 2.72380500

C 6.85663900 -1.83817700 3.23639800

C 8.05300000 -2.34154800 2.64269700

C 8.20575000 -0.04827100 4.25745000

C 9.39970200 -0.79209000 4.00424000

C 10.67141800 -0.18806400 4.24385800

C 11.86406700 -0.92076700 3.95963000

C 12.02798600 1.86825100 4.24785500

C 13.21960900 1.09207300 4.38791000

C 14.48956800 1.73045500 4.28569100

C 15.68094700 0.94881500 4.38376700

C 15.84718300 3.50129200 3.25051800

C 17.04549900 2.90533300 3.75900900

C 18.31233600 3.43302100 3.38430600

C 19.46346700 2.80212400 3.95510700

C 19.63232300 4.56017600 1.55457700

C -2.10596800 -1.80388700 -2.06229600

C 0.36348700 -1.70233700 -2.17451300

C 1.75428600 -2.93477400 -0.54876400

C 2.93945100 -2.59472000 -1.25305500

C 4.20010500 -2.80915300 -0.63327800

C 5.39187300 -2.46328800 -1.33480800

C 5.52334900 -2.86034800 1.43881300

C 6.72141300 -2.97260200 0.67215400

C 7.98551800 -2.89089300 1.32806800

C 9.18306400 -2.96736900 0.55335800

C 9.32414500 -1.94183700 3.16278500

C 10.51878700 -2.42058400 2.54696800

C 11.78808500 -2.03730000 3.07795800

C 12.98497900 -2.49202800 2.44126500

C 13.13839700 -0.32370200 4.21735200

C 14.32746700 -1.04287700 3.90651600

C 15.60010300 -0.44952300 4.16901900

C 16.80134900 -1.16012500 3.84419000

C 16.96211600 1.58726600 4.31541100

C 18.14600300 0.81287700 4.45168000

C 19.38376900 1.53034100 4.46281500

C 2.84615600 -1.63283100 -2.31129800

C 5.30835700 -1.51127200 -2.38891100

C 6.65639200 -2.74114900 -0.73563600

C 7.84672700 -2.38581400 -1.42959100

C 9.11443100 -2.68606900 -0.84338900

C 10.30708100 -2.31597100 -1.52921800

C 10.44903000 -2.91759100 1.20949300

C 11.64301000 -2.96538000 0.43102300

C 12.91355100 -2.93927100 1.08705600

C 14.10478400 -2.96339400 0.30961300

C 14.25106300 -2.12928200 2.98309100

C 15.44520000 -2.54650300 2.32169400

C 16.72401900 -2.21532300 2.87783300

C 17.91049400 -2.66968300 2.24169300

C 18.06617000 -0.60324300 4.17935800

C 19.21731900 -1.38273800 3.84675100

C 7.77033600 -1.40142000 -2.45998500

C 11.57297600 -2.63719100 -0.95419300

C 12.76951900 -2.24626500 -1.62903400

C 14.03540700 -2.58976000 -1.06915700

C 15.22796400 -2.17131800 -1.72236900

C 15.37209000 -2.94584400 0.96388300

C 16.57698600 -2.95692100 0.18800200

C 17.83731200 -3.02977000 0.84168400

C 18.98823300 -3.07617900 0.00008500

C 19.14292700 -2.37702700 2.90209600

C 16.50763800 -2.53665300 -1.18056500

C 17.69807500 -2.16738800 -1.85818500

C 18.92022500 -2.65151800 -1.30676700

H -21.61697400 -0.20492300 -3.20102900

H -20.48742200 2.06538400 -3.24411700

H -20.54379400 4.03959600 -2.37511500

H -20.77251200 4.93201400 1.97806000

H -20.66196500 5.10389300 -0.20860100

H -20.91587800 3.42907300 3.86658600

H -21.02921700 1.40737000 4.70621200

H -21.29284300 -2.53564300 2.63687600

H -21.17811400 -0.94217200 4.14642700

H -21.43050600 -2.96875200 0.25896200

H 19.77152700 -0.74263500 -3.03111200

H -21.53281000 -2.18499100 -1.77632700

H 20.88308300 1.51837200 -3.32576600

H 20.78536700 3.67964600 -2.19474700

H 20.68151400 4.72149700 -0.27990500

H 20.43933700 3.26735400 3.85873200

H 20.54760400 4.62235500 2.13504700

H 20.29850700 1.02478300 4.75651300

H 20.19225900 -1.09832500 4.22967700

H 19.96115800 -3.30527700 0.42329500

H 20.06096300 -2.85052000 2.56804800

H 19.84225900 -2.56176100 -1.87288200

H 0.59196400 -3.79604200 -2.08132800

C -1.00096200 -4.95210100 -0.62711000

C 0.11969200 -5.94217600 -0.17568700

C -0.17140600 -7.44737600 -0.48400500

C 0.79650900 -8.43629900 0.22704200

F -1.24251300 -5.23329300 -1.94479600

F -2.09740200 -5.33355700 0.08368000

F 1.29091700 -5.66230800 -0.80588400

F 0.30286300 -5.81134400 1.16199000

F -0.04231900 -7.64181400 -1.81668000

F -1.42322500 -7.78881000 -0.10704600

F 0.59738800 -9.66255400 -0.27390400

F 0.55675300 -8.46647600 1.54279800

F 2.07478700 -8.09140500 0.02356000

**CF_3_(CF_2_)_3_-(6,5) SWNT-H (1,4-L_++_: L_87_)**

C -20.65491400 -0.85031400 -2.92855000

C -19.46774900 -0.07495400 -3.17627200

C -19.50750100 1.31769300 -3.33393400

C -18.34934600 2.09702900 -3.13829300

C -18.21188500 -0.70967500 -2.93041600

C -17.01642700 0.01724900 -3.17871800

C -15.74914600 -0.62638100 -2.99150500

C -14.54944500 0.09990800 -3.23050200

C -19.61414400 3.94543400 -1.95886800

C -18.39640000 3.36929300 -2.42549100

C -17.20274200 3.82943500 -1.81211600

C -17.07985600 1.43776200 -3.24287000

C -15.86727000 2.17214700 -3.04283900

C -14.60658000 1.52794100 -3.22838100

C -13.40410600 2.25737300 -2.99722300

C -13.28817100 -0.54636800 -3.05671500

C -12.08322800 0.19523100 -3.25662900

C -10.82301900 -0.45785400 -3.10738600

C -9.61843200 0.29067600 -3.27430000

C -19.84330200 4.29501600 2.23891900

C -19.67944600 4.56585700 -0.73258900

C -18.53002800 4.63477800 0.10943400

C -17.26980100 4.45476500 -0.52398700

C -16.06570600 4.55196900 0.24661300

C -15.92578400 3.37333600 -2.28837300

C -14.72932000 3.87747000 -1.70575800

C -13.46502300 3.43949300 -2.20177700

C -12.26425200 3.91818900 -1.59255800

C -12.13922900 1.61893500 -3.19875000

C -10.93697000 2.34199800 -2.94128000

C -9.67377400 1.71095800 -3.16170400

C -8.46888400 2.42342500 -2.87474800

C -8.35644300 -0.36833400 -3.14968600

C -7.15224400 0.38483600 -3.28467400

C -5.89023700 -0.28120500 -3.17904100

C -4.67632100 0.47058700 -3.27786100

C -3.37799900 -1.52267200 -2.66209700

C -0.88932600 -1.60955400 -3.01805900

C -19.92410300 3.45220100 3.32048200

C -18.77677000 2.72833100 3.77075700

C -18.60789400 4.48377300 1.54692300

C -17.42503400 4.12265900 2.24596300

C -16.14377600 4.36141400 1.64915500

C -14.95369000 4.04307200 2.36866800

C -14.79759600 4.45971200 -0.40106100

C -13.60741200 4.54662000 0.37330800

C -12.33531600 4.45978400 -0.27560600

C -11.14383800 4.52643900 0.50457400

C -10.99911500 3.49699700 -2.10373600

C -9.80120900 3.95461300 -1.47815000

C -8.53372500 3.54935200 -1.99877000

C -7.33840200 3.98951100 -1.35791100

C -7.20737700 1.80010900 -3.11475400

C -6.00085300 2.49880800 -2.79379300

C -4.73800700 1.88511400 -3.04863700

C -3.54186500 2.57269800 -2.69375600

C -3.42565200 -0.20136300 -3.19013000

C -2.20113400 0.56426700 -3.22095100

C -0.95292100 -0.08484600 -3.20072300

C 0.22884100 0.67050200 -3.20918200

C 1.60236300 -1.33284800 -2.79000000

C 4.06072200 -1.20206100 -2.84995100

C -20.10156600 0.66948700 4.38617400

C -18.86175800 1.37219400 4.25969500

C -17.68061100 0.58157100 4.24708600

C -17.50913900 3.22309100 3.35809700

C -16.31160200 2.56596700 3.79045900

C -15.03672600 3.10837400 3.44505400

C -13.85193100 2.44068500 3.86547500

C -13.68535000 4.31346800 1.78041200

C -12.49386200 3.95857600 2.48430600

C -11.22251900 4.24998800 1.90406300

C -10.03453000 3.87457900 2.59647800

C -9.87449400 4.45844500 -0.14497300

C -8.68250400 4.50291400 0.63815900

C -7.41308100 4.45774300 -0.01175100

C -6.22239400 4.48428100 0.77451800

C -6.06959100 3.59854300 -1.88617300

C -4.87709900 4.02980100 -1.23336700

C -3.61061600 3.65521200 -1.76881000

C -2.41607200 4.07571200 -1.10641000

C -2.27432100 1.96429000 -2.95163200

C -1.08294800 2.66016400 -2.61147400

C 0.17338600 2.06652200 -2.90172500

C 1.37389900 2.75370000 -2.54976700

C 1.51994200 0.01870900 -3.22077600

C 2.70484100 0.80395300 -3.26110100

C 3.98119100 0.15675800 -3.25813100

C 5.17839700 0.94058800 -3.26506900

C 6.52157600 -1.09146000 -2.90333800

C 8.98481700 -0.97844700 -2.95025400

C -20.36464200 -2.77834600 1.99266800

C -20.18561800 -0.66659700 4.08699000

C -19.03732500 -1.38373000 3.62186600

C -17.76825800 -0.80751900 3.90630800

C -16.57234300 -1.48006300 3.49628500

C -16.39730400 1.21747200 4.21656800

C -15.20861300 0.42560700 4.24167300

C -13.93700500 1.06709500 4.24740000

C -12.74734400 0.27504600 4.22667200

C -12.57619300 2.98933800 3.52603600

C -11.38804500 2.30534200 3.92339000

C -10.11615700 2.86757900 3.60522600

C -8.92632900 2.16973300 3.97396600

C -8.76192700 4.18498700 2.02717700

C -7.57367500 3.79120900 2.70951300

C -6.30196500 4.12364100 2.15250800

C -5.11173800 3.70952600 2.82172100

C -4.95191100 4.46430700 0.12578300

C -3.76224800 4.46939900 0.91178300

C -2.49039300 4.46890400 0.26193700

C -1.29735000 4.44942700 1.04491600

C -1.15048500 3.72867400 -1.65905200

C 0.04184500 4.11724800 -0.99091200

C 1.30843400 3.77431900 -1.55862100

C 2.50583500 4.14703600 -0.88048900

C 2.63857600 2.18868400 -2.89561500

C 3.83309000 2.85945500 -2.50909200

C 5.10638400 2.30517900 -2.86232300

C 6.30243300 2.96337400 -2.44717500

C 6.44726300 0.28525000 -3.27763500

C 7.64262800 1.06392600 -3.25501700

C 8.91316500 0.40665800 -3.28259200

C 10.10875700 1.18288600 -3.23884300

C 10.25056400 -1.55368300 -2.62808900

C 11.44562200 -0.86932600 -2.99650200

C 12.71474500 -1.45668100 -2.69236000

C 13.91019800 -0.76547700 -3.04072300

C -20.44452300 -3.03750900 0.65017900

C -19.28922600 -2.90475400 -0.18929600

C -19.12252800 -2.38571200 2.59018300

C -17.93972800 -2.70720100 1.86994500

C -16.65818300 -2.42702000 2.44568300

C -15.46697200 -2.78127700 1.74037100

C -15.29542600 -0.94324900 3.84890400

C -14.11071000 -1.60987500 3.42470800

C -12.83356100 -1.07644300 3.78454700

C -11.64487400 -1.72815400 3.33380100

C -11.47196700 0.91850600 4.25376200

C -10.28494100 0.12937800 4.20325500

C -9.00862100 0.77218000 4.25001100

C -7.82013400 -0.01309200 4.17039900

C -7.65468300 2.74732600 3.68020800

C -6.46514900 2.03752900 4.02140900

C -5.19251700 2.62930700 3.75253200

C -4.00361500 1.91016000 4.06739600

C -3.84099200 4.06298800 2.27760600

C -2.64909000 3.62224900 2.92934000

C -1.37561400 3.99443900 2.39849300

C -0.18614400 3.52823700 3.02953700

C -0.02949300 4.46569800 0.39314200

C 1.16546800 4.40647500 1.17107700

C 2.43493800 4.44338600 0.51529200

C 3.62716100 4.35712100 1.28943800

C 3.76826500 3.83413100 -1.46586300

C 4.96662400 4.17579200 -0.76930300

C 6.23418800 3.89450200 -1.36566700

C 7.42872800 4.20821700 -0.65393800

C 7.57126400 2.41717700 -2.81222000

C 8.76980900 3.05708400 -2.36774200

C 10.03858900 2.52371300 -2.75135200

C 11.23400200 3.14447300 -2.28177900

C 11.37573900 0.52505500 -3.28388300

C 12.57450300 1.29927000 -3.21043800

C 13.84071600 0.64251200 -3.27928700

C 15.03485000 1.40722900 -3.16593900

C 15.17526400 -1.36047100 -2.74966200

C 16.38195100 -0.66073600 -3.07127400

C 17.65594200 -1.28363000 -2.85786500

C 18.80793200 -0.54009700 -3.18592600

C -20.60179600 -1.94847300 -2.11834400

C -19.36116500 -2.36555200 -1.51770800

C -18.16105500 -1.87682300 -2.10687900

C -18.02167000 -2.95588100 0.46070800

C -16.82045300 -2.87083600 -0.31087700

C -15.54755100 -2.98407400 0.33189600

C -14.35745300 -2.88465700 -0.44405200

C -14.19627900 -2.52921900 2.33507900

C -13.00591700 -2.84685000 1.61247600

C -11.73092800 -2.61015900 2.21488900

C -10.54348500 -2.90635900 1.48365800

C -10.37043800 -1.20772800 3.71184600

C -9.18077700 -1.83999100 3.23519700

C -7.90399100 -1.33293600 3.63051300

C -6.71718900 -1.94618900 3.13005800

C -6.54655000 0.62991000 4.23933200

C -5.35527300 -0.14948900 4.12530200

C -4.08521100 0.49253100 4.22446800

C -2.89544400 -0.27905100 4.07153200

C -2.73092700 2.51056800 3.81826000

C -1.54009400 1.77885600 4.10713500

C -0.26863400 2.38226100 3.88107800

C 0.91916000 1.63398200 4.13639800

C 1.08726900 3.90683700 2.50406800

C 2.27860300 3.41259400 3.11800700

C 3.54953300 3.80992500 2.60665900

C 4.73854000 3.29248400 3.20151500

C 4.89580500 4.41408700 0.63493900

C 6.08929400 4.30272400 1.40894400

C 7.35675700 4.38982200 0.76021200

C 8.54954600 4.24924600 1.53133500

C 8.69905900 3.94662700 -1.25619400

C 9.89390500 4.23634100 -0.53123600

C 11.16231100 3.99588400 -1.14013900

C 12.35777500 4.25730300 -0.40184100

C 12.50497300 2.61994200 -2.67866700

C 13.70038400 3.22535200 -2.18897200

C 14.96739200 2.70853100 -2.59450000

C 16.16501600 3.29406700 -2.07669700

C 16.30706000 0.74999900 -3.24710900

C 17.49692300 1.51683700 -3.12663700

C 18.75724400 0.85857100 -3.26605300

C 19.93883300 1.67343000 -3.15507200

C -16.89050400 -2.30196600 -1.60673400

C -15.69056900 -1.81699000 -2.21428300

C -14.42706200 -2.27129300 -1.73210300

C -13.22898700 -1.76330900 -2.31664600

C -13.08654300 -3.00505200 0.19878500

C -11.89302600 -2.87936300 -0.57518000

C -10.62485000 -3.02242100 0.06337600

C -9.43215100 -2.87271900 -0.70560900

C -9.26764200 -2.68504200 2.09062000

C -8.07885800 -2.96238000 1.35077100

C -6.80480300 -2.75778900 1.96197400

C -5.61533600 -3.01020200 1.20936000

C -5.43622100 -1.44738000 3.53400000

C -4.25050500 -2.03781100 3.01096100

C -2.97619600 -1.54214700 3.41925200

C -1.78426300 -2.10740900 2.86864100

C -1.62221400 0.35621300 4.20694900

C -0.43770900 -0.41105200 4.04024800

C 0.83360400 0.21208200 4.19024600

C 2.02107000 -0.55924200 3.99723100

C 2.19519800 2.24135900 3.92885700

C 3.37991700 1.48399900 4.16494500

C 4.65429700 2.09708300 3.97479300

C 5.84203300 1.33427000 4.19083200

C 6.01121000 3.70896600 2.70482100

C 7.19857800 3.17680100 3.28592900

C 8.47078300 3.61063700 2.80377700

C 9.66049800 3.06359000 3.37121900

C 9.81997200 4.36511700 0.88914100

C 11.01000200 4.19975400 1.65584300

C 12.28203000 4.33618800 1.01846800

C 13.47436200 4.14751000 1.78118500

C 13.62856000 4.04067000 -1.01831700

C 14.81954300 4.27231800 -0.27181900

C 16.09284200 4.06400700 -0.88952000

C 17.29446500 4.27902800 -0.14415700

C 17.43807300 2.80323100 -2.50565500

C 18.63539400 3.38848800 -2.00582300

C 19.87835500 2.88910500 -2.53569300

C -11.96270000 -2.22847800 -1.84295900

C -10.76550500 -1.70324500 -2.41132800

C -9.50135100 -2.18254200 -1.95112000

C -8.30151600 -1.63977800 -2.50237800

C -8.16227700 -3.03820100 -0.07265600

C -6.97339800 -2.86849800 -0.83749700

C -5.70306600 -3.04791900 -0.20813100

C -4.51211700 -2.86372400 -0.97075300

C -4.33343900 -2.82205600 1.81611200

C -3.15644100 -3.04956800 1.05339400

C -1.85457400 -2.85909100 1.65395800

C -0.67785000 -3.14423700 0.93724500

C -0.52008000 -1.65329100 3.32972400

C 0.66347700 -2.21395800 2.77816300

C 1.93819700 -1.78176700 3.26871800

C 3.13089700 -2.34744300 2.73357500

C 3.29412000 0.05729500 4.18079200

C 4.48131000 -0.70632900 3.97623200

C 5.75724400 -0.09001900 4.17179100

C 6.94696200 -0.84490700 3.95091700

C 7.11499100 1.95694800 4.02365400

C 8.30329700 1.19146100 4.21799300

C 9.57784400 1.82159900 4.07090100

C 10.76575700 1.05366100 4.24468500

C 10.93079000 3.51676500 2.90692300

C 12.12109200 2.95032800 3.45545200

C 13.39469700 3.42142500 3.00889700

C 14.58160400 2.83890100 3.53588800

C 14.74268800 4.30246400 1.15065000

C 15.93630700 4.07373100 1.90249300

C 17.21579200 4.26106000 1.28675800

C 18.40078700 4.06675300 2.04772200

C 18.56174000 4.13121400 -0.77983700

C 19.71760300 4.40069600 0.02540000

C -7.04153600 -2.13814100 -2.06081500

C -5.84039300 -1.57300300 -2.58838300

C -4.57855100 -2.09319500 -2.16833300

C -3.24790600 -3.06418000 -0.36105500

C -2.05113700 -2.88687700 -1.14622300

C -0.75396400 -3.45606100 -0.57872200

C 0.47366300 -2.83178400 -1.25092400

C 0.57806800 -2.93528000 1.54737800

C 1.80400100 -3.13822600 0.81110600

C 3.05769000 -3.01196500 1.46564500

C 4.26404800 -3.15677900 0.71068200

C 4.39958100 -1.91692300 3.22493200

C 5.60029000 -2.45022500 2.65561000

C 6.86844300 -2.03254600 3.15971300

C 8.06487700 -2.53633000 2.56342200

C 8.22007500 -0.23080100 4.15886700

C 9.41375200 -0.97658200 3.91124700

C 10.68433900 -0.36854100 4.14037600

C 11.87708500 -1.10325000 3.86204300

C 12.03913900 1.68926900 4.11337800

C 13.23125900 0.91616600 4.26340300

C 14.50007300 1.55430600 4.15187900

C 15.69215100 0.77488200 4.25919500

C 15.85578100 3.31445000 3.09603200

C 17.05450200 2.72569400 3.61202900

C 18.32085900 3.25077000 3.23228000

C 19.47268200 2.62689900 3.80967500

C 19.64065600 4.36061900 1.39210300

C -2.11226600 -2.08764100 -2.26289100

C 0.40517500 -2.00313000 -2.34315700

C 1.74595800 -3.03572900 -0.60413000

C 2.93794300 -2.75097900 -1.31342000

C 4.20302800 -2.99637700 -0.69928700

C 5.40003100 -2.68452800 -1.41324600

C 5.53162100 -3.05535400 1.36613500

C 6.72795300 -3.17320300 0.59885300

C 7.99481500 -3.09171100 1.25279400

C 9.19298100 -3.18027100 0.47871000

C 9.33686700 -2.13431500 3.07982200

C 10.53022300 -2.61792500 2.46691500

C 11.80039700 -2.22921800 2.99309300

C 12.99713300 -2.69107500 2.36125900

C 13.15101000 -0.50195600 4.11133200

C 14.33987400 -1.22381700 3.80839900

C 15.61226900 -0.62602000 4.06238000

C 16.81373600 -1.33993100 3.74637000

C 16.97261900 1.41366000 4.18304000

C 18.15709200 0.64188400 4.32811700

C 19.39430500 1.36055100 4.33084600

C 2.86767900 -1.85781000 -2.42299800

C 5.33009900 -1.75317500 -2.49089700

C 6.66244400 -2.95839600 -0.81223700

C 7.85799500 -2.61716700 -1.51134200

C 9.12508900 -2.91405600 -0.92158300

C 10.31932400 -2.55579300 -1.61165100

C 10.45903500 -3.12728100 1.13371100

C 11.65415900 -3.18426500 0.35598700

C 12.92512500 -3.15313200 1.01188300

C 14.11671500 -3.18667800 0.23526300

C 14.26318100 -2.32175500 2.89854200

C 15.45749200 -2.74661400 2.24231000

C 16.73637300 -2.40802700 2.79427200

C 17.92283400 -2.87038800 2.16424900

C 18.07825600 -0.77765000 4.07349200

C 19.22980200 -1.56073200 3.75073100

C 7.78852400 -1.65159200 -2.55759100

C 11.58509900 -2.87127800 -1.03228500

C 12.78312500 -2.49033200 -1.71183600

C 14.04828100 -2.82820700 -1.14771300

C 15.24190800 -2.41770500 -1.80510400

C 15.38423000 -3.16220700 0.88955300

C 16.58955600 -3.18303400 0.11426300

C 17.84960600 -3.24862500 0.76906700

C 19.00090200 -3.30588500 -0.07146000

C 19.15546100 -2.56803500 2.82003700

C 16.52115800 -2.77786400 -1.25891100

C 17.71220400 -2.41636900 -1.93978200

C 18.93380500 -2.89568700 -1.38287300

H -21.61349800 -0.47604900 -3.27920800

H -20.48333700 1.79329200 -3.35061300

H -20.53622100 3.77987300 -2.50760800

H -20.75890200 4.71558000 1.83472100

H -20.65021900 4.86837300 -0.35281200

H -20.90139800 3.23067400 3.73745500

H -21.01429000 1.21750300 4.59858600

H -21.27815400 -2.75168800 2.57865800

H -21.16282300 -1.13855600 4.06725400

H -21.41949100 -3.20949500 0.20543000

H 19.78774500 -1.00359600 -3.12397900

H -21.52633900 -2.44188100 -1.83533200

H 20.89954900 1.25423600 -3.44352500

H 20.79912800 3.43032600 -2.34166300

H 20.69129900 4.50194900 -0.44320400

H 20.44808400 3.09202300 3.70826200

H 20.55503600 4.43191400 1.97295600

H 20.30956200 0.85904700 4.62985100

H 20.20475800 -1.27048300 4.12922100

H 19.97337700 -3.53125100 0.35477200

H 20.07359600 -3.04548600 2.49198200

H 19.85601800 -2.81318600 -1.94980800

H -0.90144700 -2.08365100 -4.01794600

C -0.65806100 -5.00343700 -0.92945200

C -1.77786800 -5.99131900 -0.48816300

C -1.38696700 -7.49833600 -0.62060800

C -2.60138000 -8.46819500 -0.58769400

F 0.49454200 -5.51380700 -0.40273300

F -0.58121400 -5.10983400 -2.28734500

F -2.09240100 -5.78520400 0.81408200

F -2.88337600 -5.78407000 -1.24969200

F -0.57771400 -7.83026400 0.40962700

F -0.73519900 -7.71431100 -1.78657300

F -3.41115600 -8.17947800 0.44095900

F -3.29778100 -8.39477000 -1.72736400

F -2.15152000 -9.72156300 -0.44174700

**CF_3_(CF_2_)_3_-(6,5) SWNT-H (1,4-L_+_: L_27_)**

C -20.57120700 -0.69486300 -3.00330700

C -19.38541300 0.08752700 -3.23609900

C -19.42959500 1.48147000 -3.37670400

C -18.27481500 2.26226200 -3.16622800

C -18.12830300 -0.54595500 -2.99200500

C -16.93420200 0.18733800 -3.22632200

C -15.66522900 -0.45423400 -3.03992900

C -14.46622400 0.27842400 -3.26501200

C -19.55065300 4.09207700 -1.96892800

C -18.32913200 3.52584500 -2.43901400

C -17.13896700 3.98529300 -1.81895000

C -17.00276500 1.60839300 -3.27393500

C -15.79375700 2.34451700 -3.06110000

C -14.52954900 1.70636500 -3.24871300

C -13.33184700 2.43838700 -3.00804000

C -13.20331300 -0.36493100 -3.09146900

C -12.00004700 0.38364700 -3.27500700

C -10.73576000 -0.26497000 -3.12349000

C -9.53620400 0.48997900 -3.27657700

C -19.78817600 4.41314500 2.23205600

C -19.62075400 4.70193500 -0.73787900

C -18.47269400 4.76983500 0.10677300

C -17.21076300 4.59987400 -0.52575800

C -16.00868400 4.69511600 0.24818700

C -15.85939200 3.53807800 -2.29624200

C -14.66629500 4.04301900 -1.70710100

C -13.39996100 3.61521600 -2.20484600

C -12.20345000 4.09570700 -1.58997500

C -12.06363900 1.80569800 -3.20841000

C -10.86608400 2.53462400 -2.94596900

C -9.60045000 1.91141000 -3.16524000

C -8.40273800 2.63308600 -2.87684700

C -8.26991000 -0.16136500 -3.13980400

C -7.07059200 0.60094700 -3.27256800

C -5.80735300 -0.05174100 -3.15393700

C -4.60614400 0.71194200 -3.25197400

C -3.27866400 -1.25736200 -2.58544500

C -0.82215700 -1.19244700 -2.64679000

C -19.86869200 3.56126000 3.30612200

C -18.72050800 2.83417700 3.75017200

C -18.55206600 4.60894200 1.54270400

C -17.36927000 4.24533600 2.24102000

C -16.08794500 4.49201200 1.64841100

C -14.89732500 4.16875500 2.36670900

C -14.73883400 4.61383800 -0.39794700

C -13.55052400 4.69867700 0.37917900

C -12.27781300 4.62413900 -0.26909200

C -11.08641500 4.68673300 0.51325000

C -10.93534800 3.68651500 -2.10433600

C -9.74239300 4.14906500 -1.47662800

C -8.47298400 3.75607900 -2.00186200

C -7.27883100 4.20293600 -1.36076800

C -7.13523400 2.01919700 -3.11757500

C -5.93954900 2.73110300 -2.80758400

C -4.67156400 2.12163700 -3.05949600

C -3.47429900 2.82460800 -2.72845500

C -3.34045300 0.05395900 -3.14706400

C -2.14379600 0.81133900 -3.22409200

C -0.88240100 0.14113700 -3.13355600

C 0.31599900 0.92007100 -3.20937200

C 1.65330100 -1.08485700 -2.72960600

C 4.12177600 -0.97698800 -2.84186400

C -20.04408700 0.76691100 4.33743800

C -18.80458700 1.47265800 4.22255000

C -17.62222200 0.68388500 4.20304000

C -17.45296800 3.33520400 3.34466600

C -16.25491100 2.67502800 3.77099900

C -14.97977500 3.22244700 3.43216400

C -13.79359600 2.55171900 3.84615000

C -13.62876800 4.44902100 1.78358100

C -12.43678800 4.08644000 2.48328800

C -11.16448700 4.38789800 1.90749300

C -9.97499100 4.00295600 2.59275000

C -9.81725000 4.63613700 -0.13705800

C -8.62497700 4.67065800 0.64617000

C -7.35416300 4.64569300 -0.00642200

C -6.16251600 4.65817200 0.77672500

C -6.00998700 3.82971700 -1.89851800

C -4.81648500 4.25498600 -1.24233400

C -3.54533100 3.89707200 -1.79011200

C -2.35217700 4.30328400 -1.12291400

C -2.20876100 2.22158900 -2.99687800

C -1.01539700 2.91217900 -2.64640600

C 0.25068800 2.31298400 -2.93637200

C 1.44871700 3.00151000 -2.57811700

C 1.58221700 0.26321600 -3.18292800

C 2.77794500 1.04040700 -3.24253200

C 4.04809200 0.38545700 -3.24362800

C 5.24635200 1.16556800 -3.26017200

C 6.58515400 -0.87009600 -2.91262100

C 9.05080900 -0.75954400 -2.96986600

C -20.29609100 -2.65580300 1.90301800

C -20.12609500 -0.56517100 4.02117200

C -18.97566000 -1.27551200 3.55045100

C -17.70792500 -0.70124200 3.84562700

C -16.51046400 -1.36815500 3.43253200

C -16.33976700 1.32191600 4.18196900

C -15.14975100 0.53129200 4.20025300

C -13.87817600 1.17406500 4.21449400

C -12.68822200 0.38345200 4.18877100

C -12.51833800 3.10442000 3.51208500

C -11.32792800 2.41530200 3.90018300

C -10.05528000 2.98083300 3.58704000

C -8.86510100 2.27707200 3.94337100

C -8.70319300 4.32326400 2.02662000

C -7.51148100 3.91393300 2.69768700

C -6.24048700 4.26057900 2.14558500

C -5.04961100 3.82668400 2.79861600

C -4.89197900 4.65511300 0.12413200

C -3.69895700 4.64038400 0.90879300

C -2.42649300 4.66063000 0.25794500

C -1.23483500 4.61702700 1.03935100

C -1.08372700 3.95798700 -1.68013900

C 0.11209500 4.33726100 -0.99941400

C 1.38048300 4.01069600 -1.57233400

C 2.57538300 4.36596800 -0.88084600

C 2.71221400 2.42468300 -2.90341600

C 3.91100200 3.09433900 -2.51215600

C 5.17819600 2.53317800 -2.86100900

C 6.37521500 3.18578900 -2.44017200

C 6.51325400 0.50841500 -3.27685100

C 7.71107700 1.28656000 -3.25540000

C 8.97995500 0.62916500 -3.29047400

C 10.17641200 1.40486200 -3.24217800

C 10.31825400 -1.33724400 -2.65652300

C 11.51356900 -0.64924200 -3.02105400

C 12.78368200 -1.23881200 -2.72519700

C 13.97873200 -0.54350300 -3.06815600

C -20.37024800 -2.90574200 0.55852000

C -19.21165200 -2.76630100 -0.27571100

C -19.05657500 -2.26689500 2.50867900

C -17.87070900 -2.58191800 1.79025200

C -16.59211400 -2.30567300 2.37312300

C -15.39821900 -2.65332600 1.66896200

C -15.23513500 -0.83314500 3.79420300

C -14.04867900 -1.49605400 3.36880800

C -12.77397400 -0.96523700 3.73728800

C -11.58445700 -1.61498500 3.28583500

C -11.41235200 1.02739200 4.21942200

C -10.22384000 0.23783200 4.16728000

C -8.94892300 0.87941300 4.21266400

C -7.75901500 0.09035200 4.12575100

C -7.59114800 2.85453300 3.64886600

C -6.40010700 2.13809100 3.97574100

C -5.12934400 2.72755100 3.70375700

C -3.93823400 1.99493200 3.99327100

C -3.77760300 4.19402800 2.25947000

C -2.58544100 3.73832700 2.89859500

C -1.31321700 4.12507200 2.37782500

C -0.12418300 3.64372700 3.00069700

C 0.03757600 4.64909000 0.38883600

C 1.23071800 4.57479600 1.17045700

C 2.50142100 4.62888600 0.52042200

C 3.69287900 4.52603800 1.29797400

C 3.84267800 4.05936200 -1.46507500

C 5.03874800 4.38926700 -0.75884800

C 6.30599500 4.10942400 -1.35405000

C 7.50033000 4.41220100 -0.63507400

C 7.64233400 2.63759500 -2.80636200

C 8.84045400 3.27382800 -2.35770300

C 10.10772000 2.74159800 -2.74429500

C 11.30342100 3.35860900 -2.26949100

C 11.44353200 0.74778500 -3.29489200

C 12.64224200 1.52188400 -3.21606900

C 13.90859400 0.86668300 -3.29236500

C 15.10273500 1.63117400 -3.17245700

C 15.24493900 -1.14034500 -2.78465700

C 16.45087800 -0.43667300 -3.10081500

C 17.72560300 -1.06058100 -2.89491900

C 18.87691300 -0.31299300 -3.21646800

C -20.51825300 -1.80056500 -2.20374900

C -19.27895500 -2.21839200 -1.60039100

C -18.07744500 -1.72147800 -2.18037200

C -17.94652600 -2.82039600 0.37877800

C -16.74261200 -2.72771100 -0.38766500

C -15.47220900 -2.84419200 0.25901200

C -14.27849000 -2.73640200 -0.51158600

C -14.13038000 -2.40670800 2.27121900

C -12.93835500 -2.71897100 1.55098700

C -11.66742100 -2.48801500 2.16129400

C -10.47684400 -2.78192700 1.43276300

C -10.31049900 -1.09903400 3.67277100

C -9.12288700 -1.73402000 3.20078800

C -7.84761800 -1.22710900 3.59550000

C -6.65800400 -1.85105400 3.09979100

C -6.48165800 0.72907800 4.18712800

C -5.29440900 -0.05295500 4.05737200

C -4.01862300 0.58348500 4.13406500

C -2.82589200 -0.19626800 3.97812800

C -2.66494100 2.60380000 3.75726500

C -1.47765800 1.87168200 4.03830600

C -0.20633400 2.48434500 3.82754900

C 0.98331600 1.73986900 4.08692200

C 1.15089300 4.04125900 2.49045800

C 2.33966600 3.54020800 3.10013100

C 3.61274500 3.95140100 2.60118000

C 4.80225400 3.43085400 3.19489400

C 4.96425900 4.60115000 0.64969000

C 6.15531700 4.47594700 1.42447400

C 7.42535700 4.57418100 0.77993600

C 8.61723000 4.42416700 1.55122900

C 8.76971000 4.15490800 -1.23881400

C 9.96353700 4.43527600 -0.50962500

C 11.23181300 4.19960900 -1.12055300

C 12.42671000 4.45423400 -0.37966900

C 12.57339900 2.83772900 -2.67166300

C 13.76862100 3.43884500 -2.17652600

C 15.03544600 2.92644500 -2.58768000

C 16.23293200 3.50771200 -2.06479000

C 16.37516200 0.97584000 -3.26174300

C 17.56473600 1.74214700 -3.13388600

C 18.82539400 1.08631200 -3.28139500

C 20.00664100 1.90057600 -3.16240300

C -16.80795600 -2.14768600 -1.67893200

C -15.60622400 -1.65284500 -2.27601400

C -14.34280800 -2.10904900 -1.79349500

C -13.14297100 -1.59112600 -2.36550400

C -13.01120600 -2.86281800 0.13473600

C -11.81366900 -2.72548900 -0.63228700

C -10.54792900 -2.87952100 0.00985300

C -9.35065500 -2.72010200 -0.74893300

C -9.20812100 -2.57312100 2.04906800

C -8.01905700 -2.84781800 1.31049500

C -6.74820000 -2.66627900 1.93544400

C -5.55973000 -2.95659700 1.20727400

C -5.38206000 -1.35282500 3.48298000

C -4.19560700 -1.96645000 2.94975300

C -2.89979100 -1.47314300 3.36245700

C -1.70414900 -2.06510400 2.85210700

C -1.55636000 0.44717900 4.12667100

C -0.36868500 -0.31321200 3.97609000

C 0.90305200 0.31912200 4.14136900

C 2.09542700 -0.44451800 3.97194700

C 2.25754400 2.35748400 3.89558500

C 3.44536100 1.60638500 4.13524800

C 4.71990000 2.22780400 3.95592600

C 5.90995200 1.47044600 4.17425400

C 6.07541400 3.86005300 2.70991800

C 7.26395200 3.32454700 3.28785400

C 8.53733900 3.76880200 2.81503700

C 9.72770100 3.21896300 3.37842000

C 9.88809000 4.54866500 0.91169600

C 11.07826300 4.37527300 1.67715700

C 12.35043300 4.51961400 1.04139600

C 13.54282200 4.32445400 1.80196600

C 13.69697200 4.24320700 -0.99839100

C 14.88802200 4.46846100 -0.25001100

C 16.16099100 4.26638800 -0.87024100

C 17.36267400 4.47529800 -0.12349700

C 17.50582900 3.02182000 -2.49922500

C 18.70321800 3.60271900 -1.99405400

C 19.94605900 3.10947700 -2.52989600

C -11.87695000 -2.05737500 -1.88997500

C -10.67501000 -1.52037700 -2.44264100

C -9.41274000 -2.00109000 -1.98208700

C -8.20871600 -1.43620400 -2.50406400

C -8.08710200 -2.89969600 -0.11036700

C -6.87994400 -2.71460000 -0.85831900

C -5.62625500 -2.93470200 -0.23148200

C -4.41981500 -2.66562700 -0.95444500

C -4.30018200 -2.79918400 1.83344800

C -3.08359600 -3.33303500 1.10414300

C -1.75052600 -2.88666200 1.67952200

C -0.59156000 -3.10050400 0.94765900

C -0.44382800 -1.56778900 3.29591100

C 0.75288900 -2.12383400 2.76351400

C 2.02297500 -1.67504300 3.25378700

C 3.21508500 -2.22809900 2.71485400

C 3.36553900 0.18099000 4.15425600

C 4.55837000 -0.57726100 3.95448900

C 5.83100900 0.04514000 4.15158000

C 7.02319000 -0.70400400 3.92744400

C 7.18230400 2.09838600 4.01354000

C 8.37374700 1.33539700 4.20596300

C 9.64711500 1.96992200 4.06608600

C 10.83718400 1.20309300 4.23416200

C 10.99865400 3.67921300 2.92031400

C 12.19018200 3.10965700 3.46425500

C 13.46351000 3.58683700 3.02276200

C 14.65156700 3.00086500 3.54414600

C 14.81115300 4.48602400 1.17244700

C 16.00519900 4.25189200 1.92191400

C 17.28431600 4.44538500 1.30724400

C 18.46977000 4.24567200 2.06617400

C 18.62980600 4.33365000 -0.76107000

C 19.78580800 4.59689800 0.04610900

C -6.94408000 -1.93284000 -2.05878800

C -5.74859400 -1.34376500 -2.55248400

C -4.48355100 -1.84424700 -2.10846900

C -3.15069500 -2.95154500 -0.37881200

C -1.98847300 -2.72445300 -1.09360400

C -0.67028000 -3.33164500 -0.58287000

C 0.55656300 -2.69535400 -1.24157600

C 0.68058100 -2.84681600 1.54414900

C 1.88878900 -3.03544300 0.80222700

C 3.14349400 -2.88705100 1.44516700

C 4.34790300 -3.00936700 0.68186800

C 4.48441700 -1.78731100 3.20416100

C 5.68257600 -2.31233700 2.63115100

C 6.94902500 -1.88805300 3.13168500

C 8.14589200 -2.38256100 2.52859100

C 8.29521400 -0.08636700 4.13815300

C 9.48992100 -0.82746900 3.88611000

C 10.75918300 -0.21795600 4.11809300

C 11.95335800 -0.94832900 3.83301400

C 12.11013200 1.84220900 4.10976600

C 13.30356100 1.06970200 4.25290600

C 14.57178700 1.71049600 4.14746200

C 15.76517800 0.93148200 4.24696000

C 15.92541500 3.48209900 3.10869900

C 17.12494800 2.89013100 3.61903400

C 18.39075200 3.41997300 3.24375900

C 19.54344000 2.79240600 3.81569300

C 19.70928400 4.54564600 1.41243600

C -2.02252300 -1.80885800 -2.19148000

C 0.45918300 -1.74118800 -2.26371800

C 1.81680400 -2.90076200 -0.62648200

C 3.00749700 -2.55370900 -1.32424800

C 4.27754900 -2.81350000 -0.72571600

C 5.46834300 -2.48352800 -1.43754600

C 5.61274400 -2.90694600 1.33313200

C 6.80663500 -3.00221200 0.55975800

C 8.07477200 -2.92428400 1.21203500

C 9.26975500 -2.99984500 0.43453000

C 9.41576700 -1.97977200 3.04560800

C 10.60890300 -2.45352200 2.42558600

C 11.87855600 -2.06645000 2.95386700

C 13.07469000 -2.52004900 2.31627600

C 13.22594600 -0.34720500 4.08702800

C 14.41576100 -1.06430100 3.77604600

C 15.68744600 -0.46734000 4.03542900

C 16.88959500 -1.17637500 3.71126400

C 17.04478700 1.57249000 4.17717100

C 18.23037000 0.80069100 4.31380900

C 19.46670000 1.52099100 4.32430300

C 2.92532900 -1.62953400 -2.40237800

C 5.39154700 -1.53471200 -2.50230400

C 6.73484400 -2.76476700 -0.84652500

C 7.92860900 -2.41367400 -1.54524900

C 9.19732800 -2.71719100 -0.96316400

C 10.39019300 -2.34959900 -1.65092400

C 10.53665700 -2.94984200 1.08789100

C 11.73037900 -2.99679700 0.30776700

C 13.00169400 -2.96977900 0.96251400

C 14.19219400 -2.99408000 0.18438100

C 14.34040600 -2.15324500 2.85560900

C 15.53461500 -2.57005700 2.19410100

C 16.81326900 -2.23483300 2.74820700

C 17.99983600 -2.68923800 2.11258700

C 18.15353400 -0.61586700 4.04360200

C 19.30608200 -1.39376700 3.71152100

C 7.85473000 -1.43523000 -2.57958800

C 11.65801200 -2.67028700 -1.07723000

C 12.85458100 -2.28196200 -1.75459200

C 14.12112300 -2.62363700 -1.19515000

C 15.31377900 -2.20622800 -1.85015500

C 15.46044100 -2.97362300 0.83778000

C 16.66477500 -2.98631300 0.06125700

C 17.92565900 -3.05545300 0.71433600

C 19.07608200 -3.10465400 -0.12799300

C 19.23261400 -2.39132500 2.77035300

C 16.59403700 -2.57006000 -1.30855600

C 17.78396700 -2.20158000 -1.98751000

C 19.00683100 -2.68423700 -1.43597200

H -21.52916500 -0.32067500 -3.35573000

H -20.40696500 1.95393000 -3.39246000

H -20.47098800 3.92745700 -2.52084200

H -20.70372000 4.83675500 1.83089900

H -20.59343600 4.99719500 -0.35728600

H -20.84582200 3.33525900 3.72105100

H -20.95777900 1.31129100 4.55490700

H -21.21202300 -2.63421700 2.48542500

H -21.10266900 -1.03805700 3.99356400

H -21.34327000 -3.07547100 0.10868900

H 19.85700900 -0.77658500 -3.16015000

H -21.44222600 -2.30059400 -1.93062900

H 20.96736200 1.48495100 -3.45596600

H 20.86674400 3.64897400 -2.33068300

H 20.75929800 4.70271400 -0.42192500

H 20.51812300 3.26009800 3.71925900

H 20.62381600 4.61279000 1.99354500

H 20.38245700 1.01781900 4.61896800

H 20.28089600 -1.10598500 4.09225600

H 20.04929700 -3.33257000 0.29520100

H 20.15125300 -2.86393600 2.43672400

H 19.92819100 -2.59633600 -2.00347700

H -3.12239100 -4.42960200 1.19323500

C -0.75390900 -4.85354900 -1.03803100

C 0.38828500 -5.85791400 -0.69772000

C 0.00165800 -7.35723400 -0.91217300

C 1.22249900 -8.31736300 -0.97986100

F -0.88166300 -4.87134100 -2.39318800

F -1.88354900 -5.42362000 -0.50949500

F 1.46710000 -5.59825800 -1.47860700

F 0.74125600 -5.73211400 0.60529400

F -0.68789300 -7.50490700 -2.06665200

F -0.77047900 -7.76046200 0.12208500

F 0.78443500 -9.58050000 -0.89477000

F 1.87850500 -8.16959400 -2.13565100

F 2.06504900 -8.08506200 0.03659000

**CF_3_(CF_2_)_3_-(6,5) SWNT-H (1,4-L–: L_–33_)**

C -20.61750900 -0.87634400 -2.84768600

C -19.43092400 -0.09915300 -3.09317500

C -19.47258400 1.29335200 -3.24999000

C -18.31574500 2.07417700 -3.05189900

C -18.17449300 -0.73225800 -2.84545300

C -16.97968700 -0.00382000 -3.09161800

C -15.71143900 -0.64571000 -2.90196700

C -14.51214200 0.08195800 -3.13928400

C -19.58463000 3.91991400 -1.87216500

C -18.36553100 3.34595900 -2.33845000

C -17.17309000 3.80834100 -1.72478700

C -17.04531900 1.41657600 -3.15536800

C -15.83399700 2.15258300 -2.95378700

C -14.57193800 1.51004800 -3.13816700

C -13.37148100 2.24142300 -2.90656300

C -13.25001200 -0.56219900 -2.96240700

C -12.04545600 0.18126200 -3.15991900

C -10.78323600 -0.46923500 -3.00695000

C -9.58062600 0.28104500 -3.17125700

C -19.81509900 4.27798100 2.32559200

C -19.65125800 4.54152800 -0.64665300

C -18.50194500 4.61393700 0.19550200

C -17.24142800 4.43491100 -0.43726400

C -16.03770800 4.53465600 0.33382000

C -15.89521500 3.35372900 -2.20012700

C -14.69988800 3.86072300 -1.61756100

C -13.43524700 3.42457000 -2.11293400

C -12.23585100 3.90669800 -1.50373900

C -12.10491900 1.60427600 -3.10510500

C -10.90464000 2.33095800 -2.84947600

C -9.64053500 1.70180100 -3.06585500

C -8.43858900 2.42002700 -2.78155200

C -8.31552200 -0.37405200 -3.03702200

C -7.11272700 0.38180500 -3.17205900

C -5.84928400 -0.27698400 -3.05103600

C -4.64233400 0.48299300 -3.14672400

C -3.32133100 -1.48948600 -2.47962900

C -0.84347700 -1.40805400 -2.51394100

C -19.89548000 3.43683000 3.40833400

C -18.74774200 2.71368200 3.85909600

C -18.57968900 4.46595100 1.63324900

C -17.39665000 4.10700700 2.33317100

C -16.11551700 4.34572000 1.73629700

C -14.92483000 4.02880000 2.45635700

C -14.76924400 4.44411200 -0.31340400

C -13.57966700 4.53335400 0.46120000

C -12.30752900 4.44847000 -0.18780800

C -11.11560300 4.51731100 0.59237100

C -10.96983000 3.48879300 -2.01550000

C -9.77449100 3.95137600 -1.39149600

C -8.50591300 3.54888800 -1.91322400

C -7.31113300 3.99766200 -1.27635000

C -7.17367700 1.79985600 -3.01839000

C -5.97471600 2.50842200 -2.70774600

C -4.70672900 1.89424000 -2.95515500

C -3.51060300 2.59840900 -2.62723300

C -3.37920000 -0.17726800 -3.03286000

C -2.17888900 0.58646900 -3.10193800

C -0.91245100 -0.08009100 -3.00711100

C 0.28305900 0.70343900 -3.09602400

C 1.61219300 -1.28612600 -2.56565800

C 4.07949300 -1.18153000 -2.66455700

C -20.07242200 0.65436100 4.47256200

C -18.83255000 1.35745300 4.34759400

C -17.65120600 0.56731400 4.33526400

C -17.48025500 3.20865000 3.44637200

C -16.28238100 2.55201500 3.87891400

C -15.00726800 3.09448700 3.53319700

C -13.82207800 2.42742800 3.95364100

C -13.65689400 4.29990200 1.86826200

C -12.46449000 3.94453100 2.57157100

C -11.19303000 4.23744800 1.99145900

C -10.00431800 3.86012900 2.68144800

C -9.84745600 4.45340400 -0.05803000

C -8.65401400 4.49622400 0.72473800

C -7.38495800 4.45914400 0.07274200

C -6.19371900 4.47967200 0.85641000

C -6.04385100 3.61245900 -1.80817200

C -4.84865800 4.04574000 -1.15650900

C -3.58074900 3.68214800 -1.70043900

C -2.38801500 4.09721900 -1.03667800

C -2.24515800 1.99033200 -2.88438100

C -1.04829500 2.69612700 -2.54524500

C 0.21572400 2.10641600 -2.83868200

C 1.41104500 2.79844800 -2.48671400

C 1.54356200 0.05535000 -3.05372100

C 2.74163200 0.83064700 -3.11530700

C 4.00882100 0.17276100 -3.10117400

C 5.20581500 0.94636000 -3.13131000

C 6.53749500 -1.09001100 -2.75146500

C 9.00240200 -0.99021900 -2.82740700

C -20.33244600 -2.79355400 2.07691200

C -20.15612500 -0.68136900 4.17196800

C -19.00724600 -1.39800900 3.70741400

C -17.73857100 -0.82166500 3.99357300

C -16.54232100 -1.49391500 3.58481700

C -16.36800400 1.20372800 4.30525900

C -15.17894600 0.41227100 4.33109100

C -13.90729400 1.05392300 4.33690300

C -12.71777600 0.26192100 4.31749500

C -12.54621000 2.97535100 3.61266200

C -11.35690700 2.29129200 4.00963400

C -10.08526100 2.85194500 3.68923700

C -8.89460400 2.15230400 4.05580900

C -8.73180100 4.17074200 2.11069700

C -7.54129100 3.77308300 2.78929800

C -6.27142200 4.10622900 2.23087600

C -5.07890900 3.68310700 2.89354800

C -4.92304100 4.46517000 0.20406600

C -3.73068100 4.46466400 0.98835900

C -2.46182500 4.47235900 0.33613300

C -1.26602500 4.44045800 1.11983400

C -1.11819100 3.74950800 -1.59242700

C 0.07847600 4.14740600 -0.92062600

C 1.34318600 3.81981600 -1.48993200

C 2.54155100 4.18080100 -0.80085900

C 2.67609100 2.21763900 -2.80062900

C 3.87691400 2.89296200 -2.41962400

C 5.14039000 2.32383400 -2.75897500

C 6.33984600 2.97709200 -2.34581500

C 6.46968400 0.28319800 -3.13280800

C 7.66970500 1.05845600 -3.12943200

C 8.93461700 0.39745600 -3.16005800

C 10.13342100 1.17097400 -3.12469000

C 10.27263300 -1.56719300 -2.51546800

C 11.46753800 -0.88420100 -2.89028600

C 12.73812700 -1.47237700 -2.59370000

C 13.93350200 -0.78124400 -2.94574900

C -20.41029600 -3.05523500 0.73483400

C -19.25374500 -2.92409300 -0.10335700

C -19.09113700 -2.40011100 2.67576100

C -17.90738500 -2.72236100 1.95748500

C -16.62686500 -2.44140300 2.53469400

C -15.43472200 -2.79637000 1.83126700

C -15.26575200 -0.95673400 3.93885700

C -14.08093600 -1.62397300 3.51718200

C -12.80452800 -1.09020300 3.87810200

C -11.61573000 -1.74395000 3.43066800

C -11.44146700 0.90502300 4.34320500

C -10.25511900 0.11541500 4.29668600

C -8.97850800 0.75723200 4.33819500

C -7.78947800 -0.03086800 4.26427800

C -7.62164300 2.72726000 3.75859900

C -6.43271800 2.01610200 4.09802700

C -5.15929000 2.60252900 3.82034800

C -3.96875800 1.88030900 4.13351700

C -3.80825200 4.04106600 2.34981600

C -2.61704700 3.59266500 2.99376600

C -1.34430900 3.96742200 2.46118400

C -0.15153500 3.49465400 3.08915500

C 0.00442000 4.47430200 0.46774700

C 1.19751600 4.40937500 1.24679900

C 2.46823100 4.45425200 0.59554600

C 3.66339100 4.35707300 1.37459400

C 3.80953000 3.87124900 -1.38379300

C 5.00605500 4.20587100 -0.68186400

C 6.27274600 3.91474200 -1.27293200

C 7.46928600 4.22126200 -0.55676400

C 7.60476500 2.41821200 -2.70389900

C 8.80383300 3.05665600 -2.26593600

C 10.06875600 2.51402500 -2.64499400

C 11.26670600 3.13487800 -2.18054000

C 11.39929400 0.51137400 -3.17607100

C 12.59882600 1.28406400 -3.10886200

C 13.86462700 0.62672900 -3.18218500

C 15.06003100 1.39099400 -3.07307300

C 15.20012000 -1.37716100 -2.66116000

C 16.40579000 -0.67764100 -2.98659600

C 17.68073600 -1.30098600 -2.77849700

C 18.83180500 -0.55767100 -3.11008500

C -20.56426000 -1.97366200 -2.03656800

C -19.32398200 -2.38796400 -1.43294200

C -18.12346300 -1.89882200 -2.02100800

C -17.98709100 -2.97340800 0.54843500

C -16.78494000 -2.88877400 -0.22170500

C -15.51278900 -3.00042800 0.42295300

C -14.32153300 -2.90123800 -0.35134700

C -14.16524900 -2.54441100 2.42795700

C -12.97451300 -2.86267500 1.70735100

C -11.70074200 -2.62673800 2.31248800

C -10.51275200 -2.92525600 1.58434400

C -10.34173400 -1.22480700 3.81074200

C -9.15372500 -1.86092200 3.33900800

C -7.87564200 -1.35543500 3.73515100

C -6.69095700 -1.97828000 3.24633900

C -6.51631600 0.60966800 4.32664200

C -5.32600000 -0.17489700 4.21683900

C -4.05233000 0.46408600 4.30471100

C -2.86577600 -0.31319400 4.15907700

C -2.69830900 2.47559900 3.87681100

C -1.50650000 1.73693100 4.15464600

C -0.23391100 2.34030600 3.92651400

C 0.95180600 1.58970000 4.17417900

C 1.11798900 3.88422600 2.57241100

C 2.30927500 3.37731600 3.17781300

C 3.58386900 3.78636000 2.67778700

C 4.77114700 3.26463000 3.27341100

C 4.93292500 4.42823300 0.72652600

C 6.12473300 4.30135900 1.50179400

C 7.39546800 4.39361500 0.85683600

C 8.58688300 4.24478500 1.62870700

C 8.73632000 3.95357900 -1.15827800

C 9.93152900 4.23613400 -0.43220500

C 11.19849300 3.99014300 -1.04185700

C 12.39419800 4.24906000 -0.30492000

C 12.53382400 2.60642500 -2.57858200

C 13.73111000 3.21057500 -2.09199100

C 14.99615500 2.69207300 -2.50040200

C 16.19518200 3.27649700 -1.98538200

C 16.33133900 0.73359300 -3.15963900

C 17.52228500 1.49965500 -3.04112700

C 18.78178300 0.84122300 -3.18649100

C 19.96424600 1.65476200 -3.07649700

C -16.85320500 -2.32175700 -1.51835900

C -15.65249800 -1.83573900 -2.12431400

C -14.38892100 -2.28836600 -1.64003300

C -13.19025200 -1.77870000 -2.22179400

C -13.05206100 -3.02084500 0.29357800

C -11.85675000 -2.89375400 -0.47811300

C -10.59001500 -3.03858000 0.16272900

C -9.39607200 -2.88548600 -0.60186800

C -9.23982200 -2.70715700 2.19543500

C -8.04985500 -2.98705100 1.45844500

C -6.77769800 -2.79548500 2.07704800

C -5.59161300 -3.05462800 1.33086400

C -5.41203500 -1.47862700 3.64585200

C -4.22432400 -2.08547800 3.13419300

C -2.95329200 -1.59133300 3.53462400

C -1.76373600 -2.17513800 2.98714600

C -1.59040600 0.32035400 4.26501300

C -0.40354000 -0.45425600 4.09307700

C 0.86518600 0.16577800 4.22589200

C 2.05223100 -0.61081900 4.02789700

C 2.22490000 2.19743500 3.96674900

C 3.41308400 1.43661300 4.20243700

C 4.68682800 2.05971700 4.03387400

C 5.87502500 1.30289100 4.25820500

C 6.04429000 3.68909100 2.78774700

C 7.23322400 3.15213300 3.36860700

C 8.50655600 3.59452000 2.89596100

C 9.69634500 3.04609200 3.46206800

C 9.85734900 4.36129600 0.98778500

C 11.04823300 4.19037700 1.75397800

C 12.31956700 4.32782800 1.11597000

C 13.51235400 4.13659200 1.87669600

C 13.66285400 4.02777600 -0.92279700

C 14.85537500 4.25833600 -0.17823100

C 16.12656800 4.04739000 -0.79824700

C 17.32970800 4.26158700 -0.05544200

C 17.46657700 2.78465900 -2.41732800

C 18.66575700 3.36850400 -1.91967400

C 19.90667000 2.86889200 -2.45354600

C -11.92338100 -2.24162000 -1.74494100

C -10.72462900 -1.71455900 -2.30963900

C -9.46070600 -2.19121300 -1.84552000

C -8.25830400 -1.64331900 -2.38887500

C -8.12785800 -3.05346900 0.03519200

C -6.93516100 -2.88024700 -0.72296100

C -5.67124100 -3.07118000 -0.08935600

C -4.47512300 -2.87306900 -0.84001000

C -4.30899800 -2.88279700 1.94680700

C -3.13305800 -3.14326100 1.20166800

C -1.84661900 -2.95564300 1.80400900

C -0.65316200 -3.28318400 1.09678100

C -0.48968900 -1.71338700 3.42010900

C 0.68906800 -2.31238000 2.88975100

C 1.96655900 -1.85014700 3.34649300

C 3.18277200 -2.42389100 2.81861800

C 3.32922800 0.01741800 4.21840000

C 4.52221300 -0.74336700 4.04733600

C 5.79364800 -0.12077900 4.24888400

C 6.99067500 -0.87359400 4.04326000

C 7.14995700 1.93049800 4.09905700

C 8.34105700 1.16989500 4.30295900

C 9.61460700 1.80258500 4.15869400

C 10.80580800 1.03684400 4.33484200

C 10.96834300 3.50282400 3.00138600

C 12.15964900 2.93697300 3.54966600

C 13.43310400 3.40956000 3.10367000

C 14.62158000 2.82723100 3.62930000

C 14.78028200 4.29044300 1.24403000

C 15.97476100 4.06206200 1.99413300

C 17.25325200 4.24716500 1.37556500

C 18.43955600 4.05404000 2.13497400

C 18.59560200 4.11140400 -0.69349400

C 19.75311700 4.38094300 0.10916700

C -6.99614300 -2.13989500 -1.94300800

C -5.79439300 -1.56838900 -2.45558200

C -4.52966800 -2.07960600 -2.01795200

C -3.21213900 -3.12677500 -0.23204500

C -2.02668800 -2.93785600 -0.98096700

C -0.72862100 -3.53168700 -0.43288200

C 0.52268400 -2.89345500 -1.06598800

C 0.58156500 -3.11227400 1.71097100

C 1.83325400 -3.58338100 0.98645100

C 3.13560700 -3.14899100 1.62283600

C 4.31653500 -3.26752400 0.85529500

C 4.45025200 -1.96609900 3.31894200

C 5.64942400 -2.50133200 2.77219600

C 6.91994300 -2.06761300 3.27087900

C 8.11422300 -2.57374000 2.67815200

C 8.26247400 -0.25317800 4.25180500

C 9.45702700 -0.99695600 4.01010200

C 10.72786000 -0.38422500 4.23435800

C 11.92164600 -1.11844600 3.95817900

C 12.07909100 1.67473600 4.20589300

C 13.27245100 0.90332900 4.35574400

C 14.54145800 1.54257700 4.24394400

C 15.73466100 0.76396900 4.35008400

C 15.89537200 3.30394700 3.18863900

C 17.09499800 2.71599800 3.70325200

C 18.36091600 3.24123700 3.32148100

C 19.51364800 2.61881000 3.89888000

C 19.67850000 4.34467600 1.47618600

C -2.05961700 -2.03548500 -2.05432100

C 0.42380400 -1.94186300 -2.12546500

C 1.75588700 -3.12019400 -0.47394700

C 2.95412800 -2.74586900 -1.13683800

C 4.23326100 -3.05571800 -0.56651000

C 5.41383700 -2.71013800 -1.27071000

C 5.58382000 -3.11962100 1.49151300

C 6.77592700 -3.21742900 0.71501300

C 8.04281600 -3.13147700 1.36604700

C 9.23360500 -3.20966500 0.58724700

C 9.38365800 -2.16052900 3.18644900

C 10.57669700 -2.64082100 2.56957500

C 11.84639500 -2.24695300 3.09168700

C 13.04097800 -2.70651500 2.45661400

C 13.19446700 -0.51488300 4.20396200

C 14.38423700 -1.23554200 3.89872900

C 15.65635900 -0.63678100 4.15120200

C 16.85783900 -1.34909100 3.83168900

C 17.01453300 1.40362600 4.27352800

C 18.20001900 0.63240300 4.41582500

C 19.43665700 1.35227700 4.41940000

C 2.88174800 -1.81714000 -2.20704800

C 5.34128200 -1.74651500 -2.33090600

C 6.69431600 -2.98980800 -0.68874300

C 7.88335700 -2.64018200 -1.39238900

C 9.15502500 -2.93746500 -0.81193900

C 10.34698400 -2.57284000 -1.50460500

C 10.50276400 -3.15137300 1.23828800

C 11.69337800 -3.20555700 0.45607200

C 12.96562300 -3.17059900 1.10793100

C 14.15435400 -3.20196400 0.32745500

C 14.30777800 -2.33337000 2.98969200

C 15.50054200 -2.75718700 2.33035000

C 16.78002400 -2.41623600 2.87848600

C 17.96549200 -2.87673100 2.24489400

C 18.12250200 -0.78611500 4.15734500

C 19.27444200 -1.56695600 3.82991800

C 7.80818200 -1.66030900 -2.42940000

C 11.61664500 -2.89057200 -0.93212100

C 12.81113500 -2.50776600 -1.61478600

C 14.07969600 -2.84496900 -1.05514000

C 15.27072300 -2.43479400 -1.71697700

C 15.42374700 -3.17474900 0.97826200

C 16.62613500 -3.19524600 0.19953200

C 17.88851900 -3.25728100 0.85075800

C 19.03695300 -3.31601000 0.00640900

C 19.19942300 -2.57269200 2.89763200

C 16.55212200 -2.79367900 -1.17451900

C 17.74068500 -2.43287100 -1.85994200

C 18.96464700 -2.91009600 -1.30600800

H -21.57589900 -0.50387500 -3.20078200

H -20.44906400 1.76758000 -3.26821200

H -20.50630300 3.75221600 -2.42093300

H -20.73077800 4.69790400 1.92089500

H -20.62263500 4.84269600 -0.26735400

H -20.87257200 3.21562800 3.82594600

H -20.98535400 1.20208000 4.68482100

H -21.24683500 -2.76596600 2.66149100

H -21.13323400 -1.15349100 4.15110300

H -21.38460400 -3.22807800 0.28897800

H 19.81153800 -1.02184600 -3.05236000

H -21.48845900 -2.46862300 -1.75512900

H 20.92384200 1.23537900 -3.36837700

H 20.82859700 3.40846400 -2.26038300

H 20.72620600 4.47955600 -0.36126700

H 20.48843700 3.08526700 3.79764100

H 20.59410700 4.41599700 2.05509200

H 20.35230900 0.85145200 4.71835100

H 20.24991300 -1.27582600 4.20638200

H 20.01114200 -3.53938900 0.42978200

H 20.11752200 -3.04813600 2.56655500

H 19.88472900 -2.82861400 -1.87652400

H 1.83363700 -4.68247700 1.01561900

C -0.68290700 -5.05365000 -0.89290700

C -1.75952400 -6.06275100 -0.38683300

C -1.37676500 -7.56139100 -0.60517800

C -2.57545900 -8.54373500 -0.48653700

F 0.51523600 -5.61422100 -0.53510300

F -0.74656200 -5.07566900 -2.25212700

F -1.95492500 -5.89873300 0.94498800

F -2.92924200 -5.84166400 -1.03734000

F -0.46985400 -7.92152900 0.33108100

F -0.83974600 -7.73013900 -1.83552800

F -3.26997600 -8.30658500 0.63530400

F -2.10390000 -9.79691400 -0.44375300

F -3.39111400 -8.43017500 -1.53997700

**CF_3_(CF_2_)_2_CH_2_-(6,5) SWNT-H (1,2-L_++_: L_87_)**

C -20.67786700 -0.66081900 -2.99783200

C -19.49176800 0.12042400 -3.23274800

C -19.53470800 1.51458000 -3.37262400

C -18.37880900 2.29408900 -3.16391900

C -18.23488200 -0.51442800 -2.99166200

C -17.04053500 0.21807500 -3.22764600

C -15.77181900 -0.42490300 -3.04460900

C -14.57279700 0.30682300 -3.27132800

C -19.65108000 4.12420100 -1.96363100

C -18.43076500 3.55722500 -2.43560800

C -17.23941900 4.01417300 -1.81592700

C -17.10757100 1.63911700 -3.27401700

C -15.89728100 2.37382800 -3.06215400

C -14.63414900 1.73475200 -3.25263800

C -13.43487000 2.46462400 -3.01131400

C -13.31024400 -0.33836000 -3.10128200

C -12.10619000 0.40885400 -3.28690700

C -10.84315300 -0.24239700 -3.14041300

C -9.64147500 0.51109300 -3.29232700

C -19.88449900 4.43568900 2.23802900

C -19.71942900 4.73189600 -0.73134800

C -18.57064800 4.79675400 0.11235300

C -17.30949000 4.62659600 -0.52167500

C -16.10648000 4.71960000 0.25121100

C -15.96064900 3.56622300 -2.29512900

C -14.76634600 4.06847700 -1.70620600

C -13.50080700 3.63935300 -2.20525900

C -12.30277300 4.11684100 -1.59020400

C -12.16740600 1.83093100 -3.21514100

C -10.96825200 2.55629200 -2.95016200

C -9.70331000 1.93100600 -3.17155300

C -8.50329600 2.64805300 -2.87862400

C -8.37595000 -0.14425300 -3.16299400

C -7.17403200 0.61490600 -3.28559400

C -5.91044200 -0.04321200 -3.16979700

C -4.70754700 0.72120200 -3.25663200

C -3.38449500 -1.25475200 -2.60747800

C -0.91506900 -1.17178600 -2.64295200

C -19.96427500 3.58211400 3.31094600

C -18.81582200 2.85473000 3.75343800

C -18.64895800 4.63291100 1.54823200

C -17.46577700 4.26769200 2.24477300

C -16.18478300 4.51488300 1.65131800

C -14.99388000 4.19102700 2.36835900

C -14.83735100 4.63798200 -0.39620000

C -13.64842800 4.72095300 0.38000500

C -12.37593400 4.64504400 -0.26910000

C -11.18425700 4.70745800 0.51251800

C -11.03555300 3.70593700 -2.10526000

C -9.84157100 4.16500800 -1.47654800

C -8.57232200 3.76944700 -2.00126800

C -7.37814600 4.21510700 -1.35997300

C -7.23693700 2.03172900 -3.11970300

C -6.04029100 2.74076100 -2.80429600

C -4.77228000 2.13044600 -3.05630000

C -3.57605500 2.83373600 -2.72383600

C -3.44326400 0.06211200 -3.14891900

C -2.24504000 0.82519300 -3.21583700

C -0.98020500 0.15826300 -3.12556600

C 0.21756500 0.93939800 -3.20559200

C 1.55234100 -1.06091400 -2.71757500

C 4.01687100 -0.96313900 -2.83635300

C -20.13866200 0.78697500 4.34132400

C -18.89937100 1.49268900 4.22519000

C -17.71719100 0.70388200 4.20354100

C -17.54871900 3.35607400 3.34728400

C -16.35031200 2.69535600 3.77198100

C -15.07560000 3.24350500 3.43333900

C -13.88958900 2.57242500 3.84559400

C -13.72599800 4.47125600 1.78435300

C -12.53354800 4.10875500 2.48365300

C -11.26184000 4.41079100 1.90774300

C -10.07268200 4.02660600 2.59322400

C -9.91566100 4.65379700 -0.13794300

C -8.72277200 4.68950800 0.64555200

C -7.45263100 4.66237300 -0.00663800

C -6.26124600 4.67622800 0.77689100

C -6.11014800 3.83854000 -1.89601300

C -4.91588100 4.26629700 -1.24031800

C -3.64611500 3.90889400 -1.78826700

C -2.45296500 4.31774500 -1.12192900

C -2.31032000 2.22999200 -2.98895800

C -1.11474000 2.92774300 -2.64186700

C 0.15144000 2.33393600 -2.93631300

C 1.34924500 3.02313300 -2.58083300

C 1.48165700 0.28531900 -3.17705900

C 2.67827800 1.06029000 -3.23735200

C 3.94531100 0.40113300 -3.23769400

C 5.14353100 1.17864500 -3.25789300

C 6.47923100 -0.86022600 -2.91771300

C 8.94475500 -0.75328500 -2.98257300

C -20.39475500 -2.63271800 1.90466100

C -20.22107800 -0.54503100 4.02461400

C -19.07137300 -1.25500500 3.55174600

C -17.80323700 -0.68100000 3.84520000

C -16.60627700 -1.34733800 3.42910900

C -16.43465900 1.34182600 4.18152000

C -15.24468100 0.55120400 4.19765400

C -13.97351300 1.19399300 4.21151700

C -12.78331700 0.40354200 4.18228700

C -12.61450900 3.12613000 3.51184200

C -11.42408500 2.43766000 3.89942700

C -10.15277100 3.00438300 3.58722100

C -8.96175800 2.30071300 3.94317800

C -8.80054700 4.34632700 2.02681700

C -7.60886400 3.94020500 2.69972600

C -6.33878800 4.28406600 2.14676500

C -5.14639700 3.85160800 2.80192200

C -4.99043000 4.67069100 0.12432500

C -3.79677900 4.66047500 0.90852400

C -2.52660700 4.67844200 0.25626600

C -1.33205400 4.63422700 1.03827100

C -1.18342300 3.97270900 -1.68005600

C 0.01461500 4.35841900 -1.00184700

C 1.28194100 4.03459400 -1.57644700

C 2.47887200 4.38923400 -0.88589700

C 2.61287200 2.44479000 -2.90446400

C 3.81309400 3.11403200 -2.51580700

C 5.07777500 2.54866700 -2.86372700

C 6.27653100 3.19951500 -2.44548100

C 6.40903500 0.51932800 -3.27704500

C 7.60800000 1.29594500 -3.25952500

C 8.87525900 0.63697700 -3.29898200

C 10.07298500 1.41108400 -3.25259300

C 10.21293800 -1.33309200 -2.67402700

C 11.40824300 -0.64526900 -3.03915500

C 12.67837300 -1.23670500 -2.74729700

C 13.87363800 -0.54173000 -3.09067500

C -20.47158900 -2.87961700 0.55974200

C -19.31455500 -2.73856600 -0.27626800

C -19.15405100 -2.24517200 2.50873600

C -17.96962000 -2.55874900 1.78764700

C -16.68969300 -2.28325900 2.36859900

C -15.49694300 -2.62980600 1.66191200

C -15.33022400 -0.81300000 3.78955900

C -14.14456300 -1.47454800 3.36082200

C -12.86889300 -0.94369600 3.72765400

C -11.67944900 -1.59169700 3.27296800

C -11.50754800 1.04774900 4.21489200

C -10.31993900 0.25969300 4.15868900

C -9.04487600 0.90233000 4.20680600

C -7.85443400 0.11541200 4.12065100

C -7.68845800 2.88096100 3.65298100

C -6.49855200 2.16625700 3.98192400

C -5.22635100 2.75694200 3.71103100

C -4.03419000 2.02831200 4.00887300

C -3.87430700 4.21833000 2.26259500

C -2.68236300 3.76185300 2.89990700

C -1.41039700 4.14358200 2.37267200

C -0.21698300 3.65768200 2.99272800

C -0.05885000 4.67024600 0.38567300

C 1.13571400 4.59745200 1.16548900

C 2.40611900 4.65112000 0.51471200

C 3.59962200 4.54458400 1.29176200

C 3.74621800 4.08156300 -1.47102700

C 4.94343000 4.40954500 -0.76612300

C 6.20965100 4.12567900 -1.36178400

C 7.40575200 4.42607100 -0.64389500

C 7.54201400 2.64799100 -2.81241400

C 8.74144100 3.28278300 -2.36687400

C 10.00708700 2.74781100 -2.75470300

C 11.20452500 3.36346800 -2.28231900

C 11.33932100 0.75268600 -3.30964000

C 12.53892700 1.52524900 -3.23236700

C 13.80459700 0.86886300 -3.31208000

C 14.99984300 1.63195100 -3.19344400

C 15.13988100 -1.14042400 -2.81072700

C 16.34590600 -0.43741700 -3.12764400

C 17.62047100 -1.06290400 -2.92490600

C 18.77193200 -0.31605600 -3.24744500

C -20.62434700 -1.76755700 -2.19967900

C -19.38417500 -2.18773100 -1.59979100

C -18.18356300 -1.69087900 -2.18132500

C -18.04827600 -2.79439000 0.37582700

C -16.84575500 -2.70059300 -0.39260000

C -15.57400400 -2.81873800 0.25159300

C -14.38221300 -2.71052000 -0.52091100

C -14.22788900 -2.38359400 2.26191000

C -13.03683800 -2.69406600 1.53876600

C -11.76363500 -2.46360400 2.14675800

C -10.57493900 -2.75567600 1.41627700

C -10.40565900 -1.07563800 3.65833800

C -9.21776100 -1.70647000 3.18052200

C -7.94063000 -1.20195600 3.57907600

C -6.75408700 -1.82092900 3.08397700

C -6.58129500 0.75614400 4.19076100

C -5.39212100 -0.02591400 4.06665100

C -4.11668000 0.61270700 4.15598900

C -2.92915900 -0.16163300 3.99454200

C -2.76302100 2.62867000 3.76343400

C -1.57322800 1.88489600 4.02463600

C -0.29884700 2.49619900 3.80844000

C 0.88907400 1.74532600 4.05899100

C 1.05715100 4.05902200 2.48402700

C 2.24579800 3.55182800 3.08860400

C 3.52002700 3.96410600 2.59136700

C 4.70926400 3.43849000 3.18214400

C 4.87052500 4.62008200 0.64307500

C 6.06227800 4.48935800 1.41639300

C 7.33235900 4.58657900 0.77109800

C 8.52470700 4.43203200 1.54089800

C 8.67358400 4.16575700 -1.24894600

C 9.86878900 4.44315800 -0.52103400

C 11.13584400 4.20494400 -1.13356300

C 12.33194900 4.45698200 -0.39408100

C 12.47281700 2.84091300 -2.68673500

C 13.66966200 3.44015700 -2.19317800

C 14.93516100 2.92660600 -2.60688300

C 16.13411100 3.50581800 -2.08520800

C 16.27139100 0.97547000 -3.28609400

C 17.46207500 1.74030100 -3.15903900

C 18.72178000 1.08341600 -3.30993700

C 19.90411400 1.89611700 -3.19151000

C -16.91341400 -2.11877600 -1.68279300

C -15.71258400 -1.62446700 -2.28189400

C -14.44895500 -2.08224200 -1.80230700

C -13.25017800 -1.56464300 -2.37657400

C -13.11333000 -2.83732500 0.12320700

C -11.91752200 -2.70095900 -0.64629700

C -10.65093300 -2.85407500 -0.00675400

C -9.45654300 -2.69309500 -0.76870700

C -9.30311000 -2.54391500 2.02958100

C -8.11312400 -2.81739200 1.28961000

C -6.84064700 -2.63164300 1.90926900

C -5.65580200 -2.89053000 1.16300500

C -5.47944200 -1.32227400 3.48383100

C -4.28974200 -1.92354200 2.95806100

C -3.01610300 -1.43317100 3.35666300

C -1.83181900 -2.02586600 2.80933200

C -1.65654000 0.46814000 4.10765800

C -0.46677200 -0.30849900 3.92131700

C 0.80712000 0.32402000 4.09729100

C 1.99632100 -0.43774300 3.92623300

C 2.16260300 2.36297300 3.87324900

C 3.35059400 1.60776000 4.10682200

C 4.62603700 2.23026100 3.93526300

C 5.81467900 1.47099500 4.15098200

C 5.98250600 3.86786900 2.69886500

C 7.17119300 3.32703700 3.27356500

C 8.44496600 3.77199600 2.80243100

C 9.63489400 3.21826700 3.36281200

C 9.79503900 4.55512800 0.90036400

C 10.98591100 4.37751500 1.66420000

C 12.25748500 4.52091300 1.02723900

C 13.45037500 4.32220000 1.78594600

C 13.60090500 4.24393800 -1.01453400

C 14.79330000 4.46659900 -0.26745900

C 16.06498400 4.26323000 -0.88961300

C 17.26795100 4.46936200 -0.14419400

C 17.40573500 3.01897100 -2.52219900

C 18.60463100 3.59766000 -2.01794700

C 19.84598000 3.10393400 -2.55665600

C -11.98345800 -2.03298300 -1.90457900

C -10.78399600 -1.49784700 -2.46090300

C -9.52059500 -1.98087700 -2.00294000

C -8.31763600 -1.42306700 -2.53454500

C -8.18895200 -2.87037800 -0.13327700

C -6.99334100 -2.69125800 -0.88909000

C -5.73105900 -2.89910900 -0.26077500

C -4.52988700 -2.68990700 -1.00399800

C -4.37775100 -2.71735100 1.77959000

C -3.18782300 -3.00820000 1.04542500

C -1.93243900 -2.84258200 1.66805700

C -0.70821000 -3.39097200 0.98543700

C -0.54732700 -1.55633500 3.25734000

C 0.65530800 -2.13585300 2.72850300

C 1.92198200 -1.66980200 3.21242700

C 3.11820400 -2.22702700 2.68290000

C 3.26880000 0.18518400 4.11697400

C 4.46208700 -0.57490600 3.92457300

C 5.73426900 0.04590700 4.12502800

C 6.92673400 -0.70454300 3.90138700

C 7.08842800 2.09828600 3.99323700

C 8.27935700 1.33352400 4.18439700

C 9.55321600 1.96706100 4.04632700

C 10.74296900 1.19840600 4.21208600

C 10.90641000 3.67821600 2.90526000

C 12.09772600 3.10567500 3.44678600

C 13.37124400 3.58223500 3.00529300

C 14.55920400 2.99359500 3.52424600

C 14.71822000 4.48264600 1.15505700

C 15.91277900 4.24562200 1.90269500

C 17.19137200 4.43789500 1.28659900

C 18.37750700 4.23552200 2.04381200

C 18.53401300 4.32671500 -0.78365500

C 19.69145300 4.58700400 0.02237900

C -7.05500300 -1.92672500 -2.09557000

C -5.85354000 -1.34422000 -2.59140700

C -4.58897300 -1.86215800 -2.15961700

C -3.26254100 -2.97623400 -0.40738200

C -2.08239700 -2.74422900 -1.14742300

C -0.78152400 -3.35352500 -0.66906300

C 0.44921000 -2.69455300 -1.25537600

C 0.60246400 -2.91404600 1.55315700

C 1.77980600 -3.08229600 0.79717100

C 3.04986000 -2.89702700 1.42874300

C 4.24887300 -3.02490000 0.66497600

C 4.38913900 -1.78631700 3.17796100

C 5.58385600 -2.31468400 2.60948600

C 6.85250300 -1.88870200 3.10904400

C 8.04796700 -2.38684600 2.50771100

C 8.19975900 -0.08809300 4.11424200

C 9.39320100 -0.83022800 3.86224400

C 10.66370700 -0.22187500 4.09407100

C 11.85705200 -0.95356400 3.80860700

C 12.01672000 1.83664000 4.08910700

C 13.20938700 1.06265400 4.23008700

C 14.47845400 1.70229100 4.12501400

C 15.67116900 0.92184600 4.22249300

C 15.83324200 3.47415200 3.08842500

C 17.03256000 2.87997300 3.59654600

C 18.29866200 3.40877100 3.22059700

C 19.45116000 2.77930300 3.79087100

C 19.61662900 4.53423200 1.38876600

C -2.12430400 -1.80272500 -2.19635300

C 0.36076500 -1.71401800 -2.26116300

C 1.70303000 -2.93670400 -0.64894900

C 2.89814900 -2.56145600 -1.33469500

C 4.17323200 -2.82788400 -0.74544600

C 5.36145800 -2.48688300 -1.45269000

C 5.51334900 -2.91514600 1.31411600

C 6.70686800 -3.00914600 0.53975600

C 7.97546300 -2.93086600 1.19138500

C 9.16858900 -3.00468700 0.41275100

C 9.31798600 -1.98335000 3.02291500

C 10.51052200 -2.45797500 2.40196300

C 11.78081900 -2.07159200 2.92924900

C 12.97574800 -2.52561200 2.29024200

C 13.13031800 -0.35393000 4.06226100

C 14.31939100 -1.07190400 3.74959300

C 15.59191500 -0.47658200 4.00883600

C 16.79307300 -1.18632900 3.68258200

C 16.95140200 1.56159500 4.15277400

C 18.13634800 0.78832900 4.28717600

C 19.37346200 1.50734700 4.29788300

C 2.82061900 -1.61612400 -2.39583700

C 5.28524900 -1.52608800 -2.50871700

C 6.63166000 -2.76864600 -0.86467800

C 7.82408100 -2.41396500 -1.56355200

C 9.09369700 -2.71897100 -0.98436300

C 10.28590100 -2.34879700 -1.67232100

C 10.43666400 -2.95430200 1.06479800

C 11.62901500 -3.00083300 0.28308400

C 12.90095200 -2.97450800 0.93626400

C 14.09038300 -2.99820000 0.15652500

C 14.24238500 -2.15981500 2.82836200

C 15.43549400 -2.57699300 2.16508700

C 16.71494700 -2.24332200 2.71804300

C 17.90047000 -2.69769500 2.08032100

C 18.05790200 -0.62756900 4.01462000

C 19.20944400 -1.40599700 3.68009500

C 7.74933800 -1.42930900 -2.59291900

C 11.55459900 -2.67180800 -1.10141300

C 12.75012200 -2.28235400 -1.77931800

C 14.01748800 -2.62579000 -1.22223900

C 15.20939800 -2.20811800 -1.87823900

C 15.35943500 -2.97912300 0.80840600

C 16.56261700 -2.99148700 0.03033200

C 17.82435700 -3.06198800 0.68174000

C 18.97360200 -3.11086700 -0.16223800

C 19.13420500 -2.40184000 2.73728100

C 16.49016200 -2.57360100 -1.33892200

C 17.67938100 -2.20505500 -2.01905000

C 18.90273400 -2.68905600 -1.46967800

H -21.63625300 -0.28508600 -3.34746400

H -20.51163800 1.98802900 -3.38593800

H -20.57210000 3.96156200 -2.51500500

H -20.80045100 4.85949300 1.83799700

H -20.69145500 5.02752800 -0.34934600

H -20.94120900 3.35519200 3.72585500

H -21.05208900 1.33126400 4.56018500

H -21.30955400 -2.61221900 2.48889500

H -21.19771600 -1.01784200 3.99796000

H -21.44550400 -3.04817400 0.11138400

H 19.75164000 -0.78075700 -3.19361600

H -21.54832100 -2.26671500 -1.92496100

H 20.86387200 1.47992500 -3.48740200

H 20.76762400 3.64199000 -2.35797000

H 20.66453200 4.69170800 -0.44675600

H 20.42623400 3.24617400 3.69438500

H 20.53206200 4.59908900 1.96871000

H 20.28887300 1.00284200 4.59133100

H 20.18487700 -1.11974400 4.06039100

H 19.94729600 -3.33972600 0.25934500

H 20.05209700 -2.87470700 2.40195000

H 19.82336900 -2.60103800 -2.03833700

H -0.72670600 -4.46998700 1.19061800

C -0.77384200 -4.87925900 -1.05310100

C -0.90262500 -5.25258100 -2.52722100

C -0.71993800 -6.77802300 -2.74888100

C -0.93226400 -7.29725200 -4.19300800

F -2.13342200 -4.92200100 -3.01924800

F 0.03149800 -4.62585500 -3.29815100

F -1.59855500 -7.44084100 -1.95023300

F 0.54195900 -7.11508600 -2.37530400

F -2.20122600 -7.11999200 -4.57830900

F -0.65398400 -8.60901400 -4.22656800

F -0.12208400 -6.66490400 -5.05027700

H -1.60407700 -5.37188300 -0.53798000

H 0.15970000 -5.31962400 -0.68820200

**CF_3_(CF_2_)_2_CH_2_-(6,5) SWNT-H (1,2-L_+_: L_27_)**

C -20.59236900 -0.68802200 -2.99140300

C -19.40539400 0.09247700 -3.22364400

C -19.44654400 1.48736600 -3.35743800

C -18.28995900 2.26453200 -3.14427000

C -18.14930200 -0.54475700 -2.98538200

C -16.95392900 0.18755300 -3.21829700

C -15.68632400 -0.45768100 -3.03897200

C -14.48614700 0.27419800 -3.26120200

C -19.56163400 4.08839500 -1.93435000

C -18.34142100 3.52334100 -2.40860800

C -17.15016800 3.97501600 -1.78471800

C -17.01912600 1.60902900 -3.25716700

C -15.80821700 2.34106500 -3.04006700

C -14.54551400 1.70171500 -3.23294100

C -13.34475800 2.42828800 -2.98532800

C -13.22432900 -0.37383400 -3.09632700

C -12.01915000 0.37253200 -3.27638000

C -10.75742600 -0.28235200 -3.13496500

C -9.55144400 0.46947100 -3.27947500

C -19.79880700 4.37139400 2.27004200

C -19.63102500 4.68758400 -0.69788800

C -18.48328800 4.74589400 0.14745300

C -17.22145500 4.57922400 -0.48663700

C -16.01932600 4.66783500 0.28756300

C -15.87130500 3.52886900 -2.26537000

C -14.67724200 4.02594300 -1.67185700

C -13.41091400 3.59820900 -2.17141900

C -12.21381300 4.07114300 -1.55244700

C -12.07823900 1.79463400 -3.19185400

C -10.87756000 2.51495000 -2.91680900

C -9.61229600 1.88982000 -3.14027600

C -8.41304900 2.60021000 -2.83554700

C -8.29023700 -0.19002100 -3.15924900

C -7.08209500 0.56784500 -3.25878100

C -5.81934900 -0.09390900 -3.15516700

C -4.62061700 0.67147800 -3.20912700

C -3.28667500 -1.32537000 -2.61293300

C -0.85230900 -1.23489100 -2.62206700

C -19.87875900 3.51263800 3.33894100

C -18.73020100 2.78411700 3.77912100

C -18.56294700 4.57324100 1.58227800

C -17.38011300 4.20489900 2.27784700

C -16.09889400 4.45611200 1.68677400

C -14.90884300 4.12948200 2.40324600

C -14.74977600 4.58898800 -0.35925100

C -13.56167800 4.66910000 0.41842800

C -12.28879800 4.59603200 -0.22954800

C -11.09877700 4.65732300 0.55378800

C -10.94584300 3.65963800 -2.06581200

C -9.75262100 4.11677100 -1.43381100

C -8.48339000 3.72080100 -1.95538800

C -7.29120500 4.16716300 -1.31106400

C -7.14607300 1.98180900 -3.07279400

C -5.95064100 2.69041600 -2.75122300

C -4.68533800 2.08200500 -3.00032400

C -3.49096600 2.78798600 -2.66501900

C -3.35502100 0.01270800 -3.09778700

C -2.15960100 0.77947000 -3.16354700

C -0.90422400 0.12014400 -3.08796000

C 0.29174600 0.88922000 -3.15398000

C 1.63438600 -1.13246000 -2.73647900

C 4.10258100 -1.01739700 -2.85929100

C -20.05190400 0.71352300 4.35908900

C -18.81302600 1.42006800 4.24515700

C -17.63052500 0.63156400 4.21915400

C -17.46315400 3.28788400 3.37581500

C -16.26465600 2.62568500 3.79803600

C -14.99049700 3.17636000 3.46305900

C -13.80409900 2.50324700 3.87178800

C -13.64056700 4.41384300 1.82177200

C -12.44905300 4.05025700 2.52094200

C -11.17771900 4.35695700 1.94787700

C -9.98875300 3.97236400 2.63370700

C -9.82886000 4.60626900 -0.09486900

C -8.63828000 4.64507500 0.69010800

C -7.36755000 4.61871500 0.04018800

C -6.17669800 4.63764500 0.82572900

C -6.02189100 3.79191000 -1.84404900

C -4.83100300 4.22361800 -1.18915500

C -3.56125700 3.86478000 -1.73360400

C -2.36721700 4.27842900 -1.06957800

C -2.22534100 2.19136200 -2.93619300

C -1.03416600 2.88192700 -2.59109400

C 0.22959800 2.28216500 -2.87948300

C 1.42932000 2.96826000 -2.52538500

C 1.56124400 0.22782300 -3.14938300

C 2.75393400 1.00607900 -3.20893800

C 4.02598500 0.35333100 -3.22957400

C 5.22405400 1.13542800 -3.24027700

C 6.56655900 -0.90345300 -2.92818500

C 9.03196900 -0.78915800 -2.98328800

C -20.30940500 -2.68970900 1.90218200

C -20.13412200 -0.61691500 4.03571800

C -18.98469300 -1.32373900 3.55767400

C -17.71632800 -0.75123500 3.85339400

C -16.51931100 -1.41481600 3.43250400

C -16.34814800 1.26976100 4.20034400

C -15.15796000 0.47941800 4.21112900

C -13.88679800 1.12262400 4.22809500

C -12.69563500 0.33317400 4.19255600

C -12.52950900 3.06091400 3.54343500

C -11.33928300 2.37069300 3.92586200

C -10.06791400 2.94257300 3.61967500

C -8.87643000 2.23903100 3.97051800

C -8.71701700 4.29952900 2.07182700

C -7.52642300 3.89332400 2.74429200

C -6.25493600 4.24454700 2.19564500

C -5.06329900 3.81603200 2.85160100

C -4.90588900 4.63461500 0.17571500

C -3.71342000 4.62589100 0.96039700

C -2.44076600 4.64365400 0.30935100

C -1.24755900 4.60457600 1.08959000

C -1.10051000 3.93535600 -1.62735000

C 0.09537300 4.31410400 -0.95123200

C 1.36343400 3.98186600 -1.52490100

C 2.56031300 4.33893500 -0.83928500

C 2.69031800 2.39012200 -2.85785300

C 3.88941600 3.05804500 -2.47123700

C 5.15679400 2.49868200 -2.82782900

C 6.35507700 3.15124400 -2.41105200

C 6.49232200 0.48043300 -3.27235500

C 7.68969900 1.25879900 -3.24496300

C 8.95979900 0.60240500 -3.28962400

C 10.15644800 1.37758200 -3.23695100

C 10.29939600 -1.36964900 -2.67533400

C 11.49420200 -0.67841900 -3.03370900

C 12.76424000 -1.27066200 -2.74218400

C 13.95928800 -0.57351200 -3.08032800

C -20.38729000 -2.92717200 0.55551500

C -19.23078000 -2.78153600 -0.28016000

C -19.06827900 -2.30690500 2.50818200

C -17.88429200 -2.61575500 1.78422300

C -16.60376400 -2.34430300 2.36620500

C -15.41177200 -2.68665500 1.65677800

C -15.24311300 -0.88225600 3.79474000

C -14.05704100 -1.54041000 3.36018100

C -12.78047500 -1.01136300 3.72858200

C -11.59126500 -1.65489700 3.26762500

C -11.42096700 0.97819200 4.22849300

C -10.23149600 0.19176500 4.16162100

C -8.95652100 0.83583500 4.21668100

C -7.76643600 0.05367500 4.11638000

C -7.60519800 2.82570000 3.68759000

C -6.41318800 2.11028800 4.00920600

C -5.14234700 2.71139300 3.75281000

C -3.95158000 1.98507500 4.04512900

C -3.79151900 4.18564100 2.31477400

C -2.59817800 3.73073100 2.95400300

C -1.32486700 4.11620200 2.43142300

C -0.13415700 3.63348900 3.05003100

C 0.02341200 4.63365900 0.43706600

C 1.21909000 4.55908000 1.21506100

C 2.48925500 4.60892000 0.56160100

C 3.68233400 4.50353300 1.33488800

C 3.82486000 4.02680900 -1.42612400

C 5.02366600 4.35670700 -0.72503100

C 6.28928000 4.07452700 -1.32369500

C 7.48549200 4.37584200 -0.60828400

C 7.62174600 2.60608300 -2.78466100

C 8.82167700 3.24046600 -2.33671400

C 10.08868700 2.71094100 -2.72955100

C 11.28572900 3.32483200 -2.25372300

C 11.42378400 0.72069600 -3.29651700

C 12.62316400 1.49359100 -3.21350000

C 13.88940400 0.83816700 -3.29539900

C 15.08417400 1.60095000 -3.17167900

C 15.22514600 -1.17293500 -2.80061100

C 16.43139900 -0.46817800 -3.11261100

C 17.70576500 -1.09418400 -2.91052100

C 18.85735200 -0.34575100 -3.22874100

C -20.54014700 -1.79832600 -2.19788000

C -19.30046300 -2.22310600 -1.60066200

C -18.09944000 -1.72479200 -2.18040900

C -17.96421200 -2.84217500 0.37111000

C -16.76204600 -2.74541900 -0.39741700

C -15.49053600 -2.86808700 0.24550200

C -14.29934000 -2.75718500 -0.52754100

C -14.14178100 -2.44307700 2.25681600

C -12.95111300 -2.75088400 1.53125900

C -11.67740000 -2.52226400 2.13796100

C -10.48943600 -2.81126800 1.40359300

C -10.31612200 -1.13800700 3.65108100

C -9.12645000 -1.76412700 3.16637000

C -7.85043400 -1.25692400 3.56021600

C -6.66139000 -1.86413100 3.04747500

C -6.49207100 0.69751100 4.19397400

C -5.30098700 -0.07661000 4.05895800

C -4.03163700 0.56642100 4.16519100

C -2.84015000 -0.19954600 3.99065100

C -2.67837700 2.59479600 3.81259000

C -1.48805300 1.85882200 4.08265400

C -0.21604400 2.46761100 3.87108200

C 0.97188400 1.71275300 4.10938900

C 1.14077500 4.02657600 2.53564800

C 2.33101800 3.51715200 3.13789600

C 3.60369100 3.92510600 2.63733100

C 4.79235200 3.39257300 3.22168200

C 4.95253400 4.57256100 0.68293800

C 6.14515000 4.44204900 1.45449500

C 7.41366200 4.53671900 0.80740000

C 8.60616100 4.37904500 1.57580300

C 8.75379500 4.11773100 -1.21486700

C 9.94936200 4.39475000 -0.48698800

C 11.21655500 4.16010000 -1.10035400

C 12.41273500 4.40913700 -0.35927600

C 12.55544800 2.80589000 -2.66068200

C 13.75190800 3.40329300 -2.16342400

C 15.01817700 2.89268200 -2.57889100

C 16.21672300 3.46951100 -2.05310900

C 16.35628900 0.94519200 -3.26570500

C 17.54652400 1.70980100 -3.13436400

C 18.80662600 1.05389200 -3.28636800

C 19.98856300 1.86661700 -3.16376800

C -16.82970000 -2.15693000 -1.68484400

C -15.62891300 -1.66142100 -2.28238600

C -14.36601200 -2.12324100 -1.80562000

C -13.16668000 -1.60516300 -2.37917200

C -13.03025100 -2.88887300 0.11491000

C -11.83642900 -2.75196200 -0.65558900

C -10.56998200 -2.90768800 -0.01766600

C -9.37679300 -2.75005500 -0.78341800

C -9.21441100 -2.60061600 2.01407000

C -8.02784200 -2.87404500 1.27121200

C -6.75181000 -2.67434300 1.88156100

C -5.56375600 -2.93162000 1.12641900

C -5.38145000 -1.36502100 3.44669400

C -4.19699200 -1.94096700 2.90017000

C -2.91809800 -1.44631300 3.31234100

C -1.72856300 -2.00384300 2.75855800

C -1.56848400 0.43360900 4.14448200

C -0.38502300 -0.32851900 3.96203400

C 0.88745300 0.29045700 4.13102100

C 2.07371400 -0.47784800 3.93093100

C 2.24761900 2.32483400 3.91866600

C 3.43168100 1.56171100 4.13851200

C 4.70725200 2.17884100 3.96547100

C 5.89377000 1.41154100 4.16866000

C 6.06589500 3.81936600 2.73679900

C 7.25258300 3.27240700 3.30662600

C 8.52619800 3.71529900 2.83518700

C 9.71489600 3.15568800 3.39191600

C 9.87623200 4.50241600 0.93500400

C 11.06633100 4.32107200 1.69812200

C 12.33801700 4.46593800 1.06217000

C 13.53026200 4.26278800 1.82075400

C 13.68228800 4.20028000 -0.98010100

C 14.87412900 4.41886100 -0.23089900

C 16.14651400 4.21958600 -0.85306600

C 17.34887400 4.42111200 -0.10516700

C 17.48899400 2.98557200 -2.49172600

C 18.68722700 3.56182100 -1.98330100

C 19.92931600 3.07151500 -2.52362700

C -11.90191200 -2.07866800 -1.91149800

C -10.70182000 -1.54226900 -2.46666700

C -9.43911800 -2.03388000 -2.01619400

C -8.23620100 -1.47980800 -2.54937100

C -8.11194400 -2.93095000 -0.15240000

C -6.92156800 -2.75471300 -0.91644000

C -5.65579600 -2.97037700 -0.29478300

C -4.46941800 -2.81935800 -1.06073100

C -4.28537300 -2.72172600 1.71396800

C -3.10168900 -2.95514700 0.92696200

C -1.79289600 -2.74863600 1.53490800

C -0.62079500 -3.01893500 0.82910200

C -0.46611800 -1.55633500 3.23039300

C 0.72087400 -2.11538200 2.68344900

C 1.99334600 -1.69354000 3.18739400

C 3.18490900 -2.26029000 2.65534200

C 3.34611400 0.13548400 4.12581000

C 4.53391000 -0.62609500 3.91385600

C 5.80910000 -0.01284000 4.12362000

C 6.99919300 -0.76461000 3.89677700

C 7.16763300 2.03694600 4.01691400

C 8.35588600 1.26748000 4.19952200

C 9.63101300 1.90005000 4.06709800

C 10.81882900 1.12888900 4.22886800

C 10.98618500 3.61600000 2.93637000

C 12.17608900 3.03875600 3.47477100

C 13.45007100 3.51643200 3.03617700

C 14.63677000 2.92437400 3.55301400

C 14.79831700 4.42619000 1.19176200

C 15.99210300 4.18411200 1.93902200

C 17.27125800 4.37984300 1.32534900

C 18.45659500 4.17227900 2.08235900

C 18.61546600 4.28291800 -0.74439800

C 19.77226000 4.53739000 0.06444500

C -6.97666300 -1.99570500 -2.11896500

C -5.76087700 -1.42329200 -2.61892900

C -4.51309800 -1.97016500 -2.21803600

C -3.20979100 -3.07471800 -0.45376300

C -2.01077500 -3.13451500 -1.38343800

C -0.65880900 -3.47492700 -0.64670800

C 0.54598100 -2.80791500 -1.31664200

C 0.64030200 -2.83692100 1.45656500

C 1.86376100 -3.09056200 0.73979700

C 3.11466200 -2.93842300 1.39332900

C 4.32526300 -3.07098800 0.63797600

C 4.45430600 -1.83023600 3.15276200

C 5.65551800 -2.36246600 2.58763300

C 6.92232200 -1.94484800 3.09478100

C 8.12070000 -2.44173000 2.49532300

C 8.27274400 -0.15348000 4.11690100

C 9.46674700 -0.89597100 3.86263800

C 10.73736700 -0.29131800 4.10180000

C 11.93081100 -1.02209900 3.81411300

C 12.09309100 1.76640300 4.11031200

C 13.28498700 0.99070400 4.24839900

C 14.55437400 1.63013300 4.14790600

C 15.74638500 0.84875000 4.24310600

C 15.91128300 3.40611000 3.12036100

C 17.10975500 2.80877100 3.62664800

C 18.37641100 3.33874400 3.25443700

C 19.52796300 2.70594600 3.82271500

C 19.69631700 4.47503300 1.43038900

C -2.02957600 -1.87113500 -2.24449100

C 0.44441100 -1.80701600 -2.28024900
[truncated: 246,820 more chars]
